# Multi-substituted trifluoromethyl alkene construction *via* gold-catalyzed fluoroarylation of *gem*-difluoroallenes†

**DOI:** 10.1039/d3sc06060h

**Published:** 2024-01-25

**Authors:** Zhi-Qiang Li, Hai-Jun Tang, Zaixin Wang, Cheng-Qiang Wang, Chao Feng

**Affiliations:** a Technical Institute of Fluorochemistry (TIF), Institute of Advanced Synthesis (IAS), School of Chemistry and Molecular Engineering, State Key Laboratory of Material-Oriented Chemical Engineering, Nanjing Tech University 30 South Puzhu Road Nanjing 211816 P. R. China cqwang08@njtech.edu.cn iamcfeng@njtech.edu.cn

## Abstract

An unprecedented fluoroarylation of 1,1-difluoroallenes with a cost-effective nucleophilic fluoride reagent and aryldiazonium salts is reported. This visible light promoted gold-catalyzed reaction allows a stereo- and regioselective incorporation of both the fluorine atom and aryl group, enabling a straightforward construction of multi-substituted trifluoromethyl alkenes. Under the mild reaction conditions, a nice tolerance of diverse functional groups is achieved. The high regioselectivity for fluorine-incorporation is rationalized by considering the thermodynamic driving force of trifluoromethyl group formation, whereas the counterintuitive stereoselectivity that aryl is installed on the side of the bulkier γ-substituent is interpreted by alleviating the increasing 1,3-allylic interaction in the gold-coordinated allene intermediate en route to the product.

## Abstract 2

A visible-light-promoted gold-catalyzed fluoroarylation of *gem*-difluoroallenes is developed. This reaction proceeds in a highly stereo- and regioselective manner and enables a facile construction of tri- and tetra-substituted trifluoromethyl alkenes.

## Graphical abstract

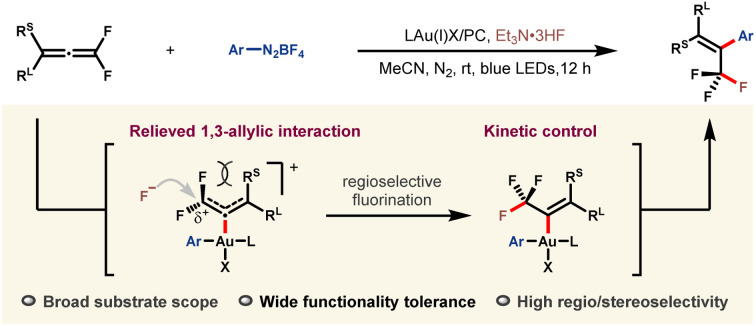

## Introduction

**Scheme 1:**
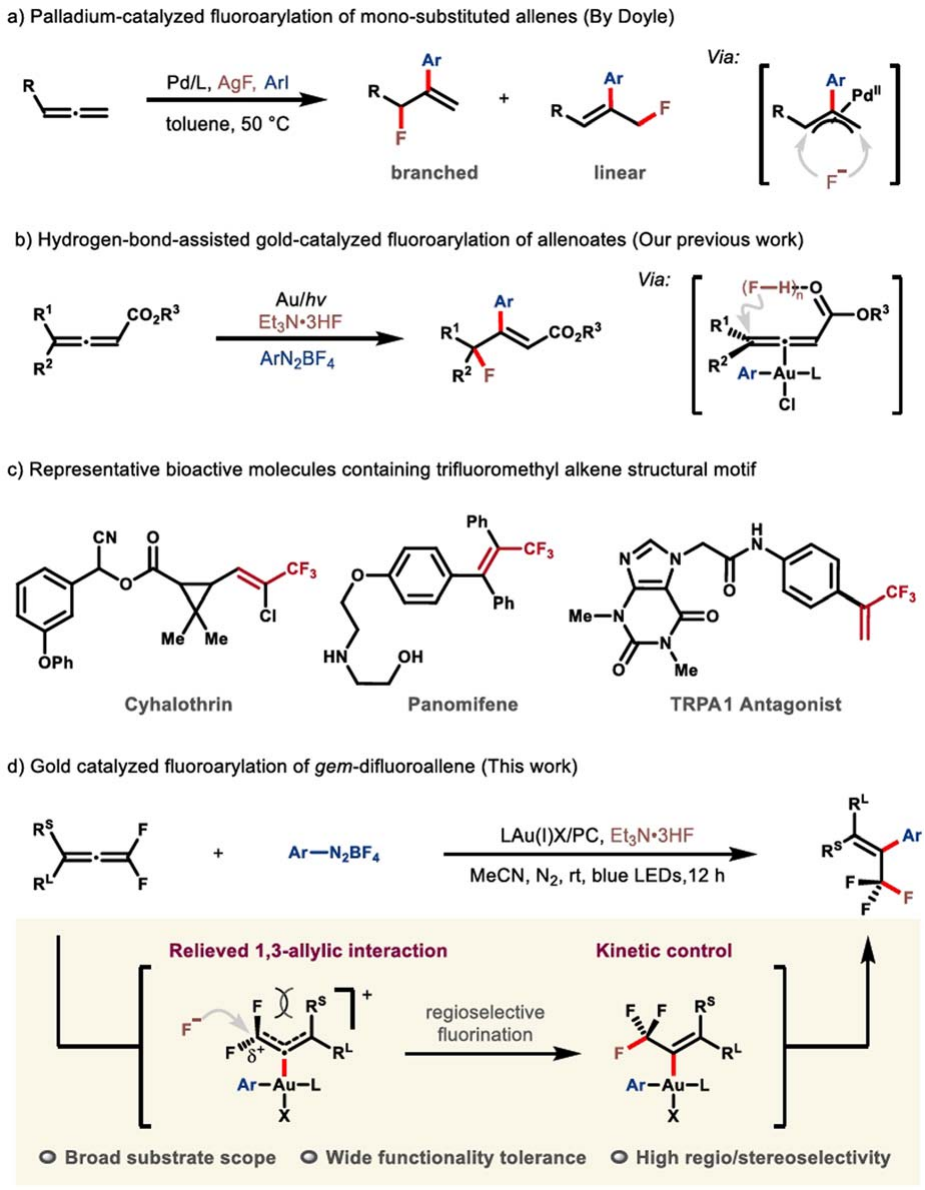
Intermolecular fluoroarylation of allenes and representative bioactive molecules containing the trifluoromethyl alkene skeleton.

Fluorine-decorated molecules, compared with their nonfluorinated analogues, often exhibit fundamentally differing physicochemical and biological properties because of the unique character of the fluorine element.^1,2^ The fact that nature is inadequate in producing fluorinated architectures has directly caused an increasing demand for synthetic techniques in both industrial and academic fields.^3–6^ To this end, diverse bespoke reagents and synthetic strategies have been successfully developed in recent decades.^7,8^ Among these protocols, the fluoroarylation of π systems, which permits the concomitant incorporation of a fluorine atom and an aryl group, proves to be a versatile platform for the rapid buildup of molecular complexity. Continuing endeavors from the synthetic community have thus been rewarded by a prominent advance in this area.^9,10^ However, the expensive electrophilic fluorination reagents, compromised substrate scope, and low atom-economy still remain as the conspicuous issues of concern.^11–14^ Consequently, the pursuit of more enabling protocols that employ readily available, cost-effective nucleophilic fluorination reagents is still in high demand. Recent advances from the groups of Loh and Feng,^15^ Ogoshi and Ohashi,^16^ Malcolmson^17^ and Zhang^18,19^ have demonstrated the feasibility of transition metal-catalyzed fluoroarylation of specific alkene derivatives, such as *gem*-difluoroalkenes and tetrafluoroethylene, though somewhat expensive silver fluoride is frequently required. By contrast, further extrapolation of this chemistry to accommodate allene counterparts is far less explored, probably due to the more complicated reactivity profile and potential selectivity issues. It is of note that Doyle and co-workers disclosed an elegant protocol leading to an expedient fluoroarylation of mono-substituted allene substrates, although the regioselectivity of fluorine-incorporation was not that encouraging (Scheme 1a).^20^ Very recently, by making use of readily available Et_3_N·3HF as the fluoride, our group had reported the first example of gold-catalyzed fluoroarylation of allenoates (Scheme 1b).^21,22^ Notwithstanding the advance in this vein, devising more efficient synthetic protocols for structurally diversified fluorinated frameworks is still of particular importance.

As a preeminent class of fluorinated scaffolds, trifluoromethyl alkenes widely occur in biologically active molecules, pharmaceuticals and functional materials (Scheme 1c),^23,24^ and find extensive applications in the preparation of more elaborate fluorine-containing compounds.^25–27^ As such, the development of efficacious synthetic strategies for expedient assembly of sophisticated trifluoromethyl alkenes, especially those that are not readily available by using the extant methods, has evoked enormous interest from multi-discipline.^27–29^ In the context, the Witting-type olefination^30–32^ and transition-metal-catalyzed cross-coupling reactions^26,27,33–35^ evolve to be the state of the art, despite the remaining issues such as strongly basic reaction conditions, volatile and expensive trifluoromethyl reagents, and poor stereoselectivities. With our continuing interest in the fluoroarylation of π systems,^15,21,36,37^ we would like to report herein our latest advancement in this territory (Scheme 1d). Notable features include: (i) the coordination of the allene motif by the *in situ* generated trivalent gold complex not only prompts the nucleophilic fluorination *via* substrate activation, but more importantly induces a cascade which eventually affords the trifluoromethyl alkene with high stereoselectivity; (ii) the fluoroarylation is subjected to a charge-controlled scenario, by which the nucleophilic attack of fluoride selectively targets the α-carbon atom, while the formation of the trifluoromethyl in turn permits a thermodynamic driving force for such a step.

## Results and discussion

We began our initial studies by using 1,1-difluoroallene 1a and aryldiazonium salt 2a as the model substrates. To our delight, when [Au(PPh_3_)]Cl and Et_3_N·3HF were used as the catalyst and fluoride source, reaction carried out in MeCN under 5 W blue LEDs afforded the desired product 3aa in 62% NMR yield (Table 1, entry 1).^38^ Further screening showed that the nucleophilic fluorides such as CsF, ^*n*^Bu_4_NF and pyridine·*x*HF were not effective (Table 1, entries 2–4). Low yields and stereoselectivity were obtained when DCE or DMF was employed as the solvent (Table 1, entries 5 and 6). Gold catalyst analysis indicated that [Au(PPh_3_)]NTf_2_ was also suitable, affording 3aa in 56% yield and better stereoselectivity (Table 1, entries 1, 7 and 8). Considering that the merger of gold and photoredox catalysis is the prevailing strategy to improve reaction turnover,^39–41^ the influence of photocatalysts in this reaction is further interrogated. Among a panel of photocatalysts, xanthone turned out to be optimal, resulting in a sharp increase of reaction efficiency and stereo-selectivity (Table 1, entries 9–12). Further control experiments verified the indispensability of the gold catalyst (Table 1, entry 13), whereas the photocatalyst and light irradiation were beneficial (Table 1, entries 8, 12 and 14).^42^

**Table 1:** Reaction condition optimization^a^

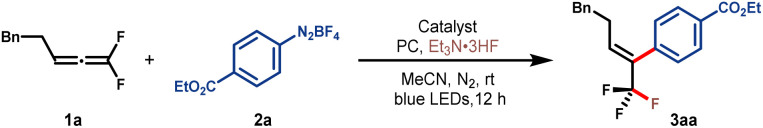				
Entry	Catalyst	PC	Yield (%)	*E*/*Z*
1	[Au1]	—	62	5.2/1
2	[Au1]	—	22^b^	15.9/1
3	[Au1]	—	33^c^	6.1/1
4	[Au1]	—	14^d^	*E* only
5	[Au1]	—	23^e^	3.9/1
6	[Au1]	—	Trace^f^	—
7	[Au2]	—	24	5.0/1
8	[Au3]	—	56	8.4/1
9	[Au3]	Ru(bpy)_3_(PF_6_)_2_	72	7.9/1
10	[Au3]	PTH	78	8.5/1
11	[Au3]	Thioxanthen-9-one	55	10.1/1
12	[Au3]	Xanthone	89(86)	16/1^g^
13		Xanthone	Trace	—
14	[Au3]	—	43^h^	>99/1

a Unless otherwise noted, all the experiments were conducted with 1a (0.1 mmol), 2a (2.0 equiv.), Et_3_N·3HF (10 equiv.), catalyst (10 mol%), and PC (5 mol%) in MeCN (1 mL) under 5 W blue LEDs for 12 h in a Schlenk tube under N_2_; yield was determined by crude ^19^F NMR with 1-iodo-4-(trifluoromethyl)benzene as the internal standard and the *E*/*Z* ratio was also determined by crude ^19^F NMR; isolated yield was indicated in the parentheses. [Au1] = [Au(PPh_3_)]Cl, [Au2] = [Au(SMe_2_)]Cl, [Au3] = [Au(PPh_3_)](NTf_2_).

b CsF was used as the fluoride source.

c Bu_4_NF was used as the fluoride source.

d Pyridine·*x*HF was used as the fluoride source.

e DCE (1,2-dichloroethane) was used as the solvent.

f DMF was used as the solvent.

g *E*/*Z* ratio was determined by ^19^F NMR of the isolated product.

h No blue LEDs. PC = photocatalyst, PTH = 10-phenyl-10*H*-phenothiazine.

With the optimal reaction conditions in hand, the substrate scope with respect to both 1,1-difluoroallene 1 and aryldiazonium salt 2 was subsequently examined, and the results are summarized in Table 2. A variety of functionalized monoalkyl substituted *gem*-difluoroallenes (1a–1r) were well accommodated, leading to the corresponding trifluoromethyl alkenes in moderate to high yields and good *E*/*Z*-selectivities. Functionalities such as phenyl (1a and 1b), halogen (1c and 1d), and ester (1e) on the tethered carbon chain proved to be well tolerated. Furthermore, 1,1-difluoroallenes substituted with electron-deficient arene (1f) or electron-rich furan (1g) also engaged in this reaction smoothly to afford the desired 3fa and 3ga in 54% and 56% yields, respectively. To our delight, hydroxycitronellal-derived allene 1h was also well tolerated, delivering 3ha in good yield and stereo-selectivity. To evaluate the influence of steric hindrance on the *E*/*Z* selectivity of this protocol, a series of *gem*-difluoroallenes containing secondary alkyl substitutes at the γ position were assessed. In general, the desired products 3ia–3na were readily obtained with high *E*/*Z* ratios (>15/1). Notably, substrates bearing an additional alkene motif did not show any interference with the desired fluoroarylation as demonstrated by the examples of 3la and 3na. Alicyclic 1,1-difluoroallenes also participated in this reaction without any issue (1m and 1n). Furthermore, sterically more hindered tertiary alkyl-substituted allenes were also proved to be applicable in this protocol (3oa–3ra). The generality with regard to aryldiazonium salt was also investigated, and substrates bearing a wide range of electron-withdrawing or electron-donating groups were compatible. Functional groups such as ketone (3ab–3ad), nitro (3ae), CF_3_ (3af), Ms (3ag), ester (3ah) and OMe (3ak) were well tolerated. When naphthyl diazonium salt 2j was employed, product 3aj was isolated in 57% yield with excellent stereo-selectivity. Furthermore, aryldiazonium salts with halogen substitutes underwent this fluoroarylation uneventfully, providing the potential handle for further synthetic elaboration through the well-developed cross-coupling reactions. In addition, aryldiazonium salts derived from (+)-menthol (2p) and coumarin 120 (2q) were amenable to this reaction, showcasing the synthetic potential of this protocol. γ,γ-Disubstituted *gem*-difluoroallenes were also competent to deliver the desired tetra-substituted trifluoromethyl alkenes in moderate to good yields (3sa–3afa). Consistent with the outcomes of monoalkyl-substituted *gem*-difluoroallenes, these reactions inclined to deliver the *E*-isomers by introducing the aryl group from the side of the bulkier substituent. It is a rational corollary that the stereoselectivity would deteriorate to a certain extent with a decrease of steric discrepancy between the two substituents, however, the reversion of *E*/*Z*-selectivity in the case of 3ta is still somewhat surprising. Allene substrates bearing a wide range of functionalities, such as aryl fluoride (1u), aryl chloride (1v), alkyl (1w), alkenyl (1y), alkyl chloride (1z), cycloalkyl (1aa, 1ab and 1ae), Boc-protected amine (1ac), and thioether (1ad), all uneventfully participated in this transformation with good yields and stereoselectivities. Of note, substrates derived from more complex molecules, such as those based on piperonyl acetone (1x) and dl-α-tocopherol (1af), were also well amenable to this reaction. It needs to be emphasized that the stereoselective construction of tetra-substituted trifluoromethyl alkenes represents an enduring challenge and the present reaction offers a straightforward avenue toward these entities.^43–46^

**Table 2:** Substrate scope^a^

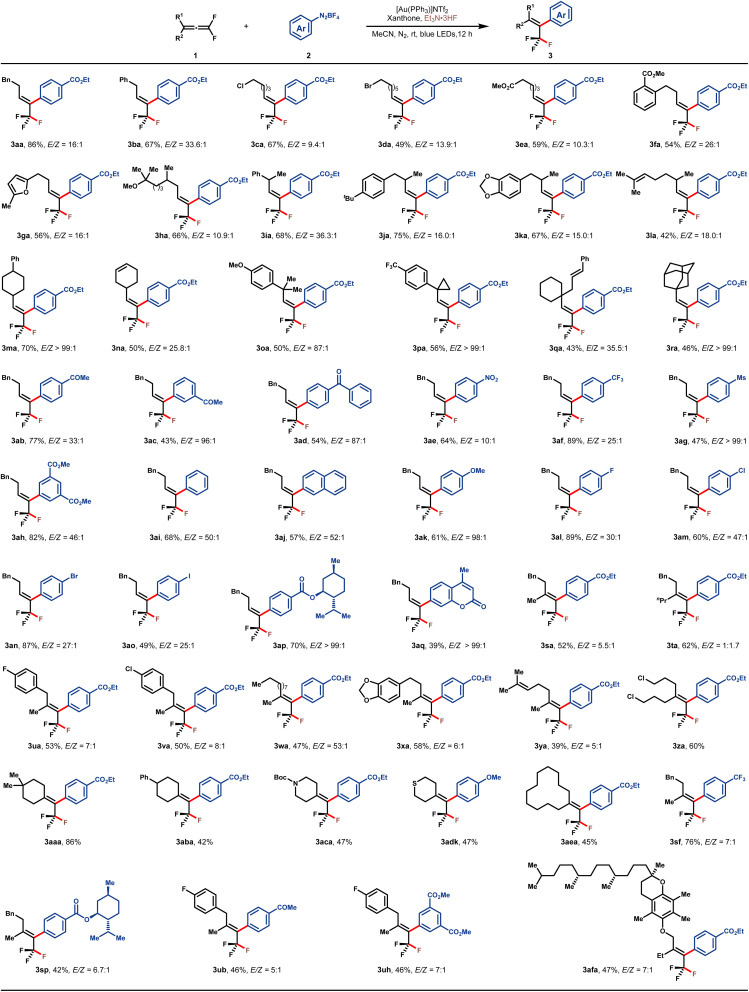

a See the ESI for reaction details.

**Scheme 2:**
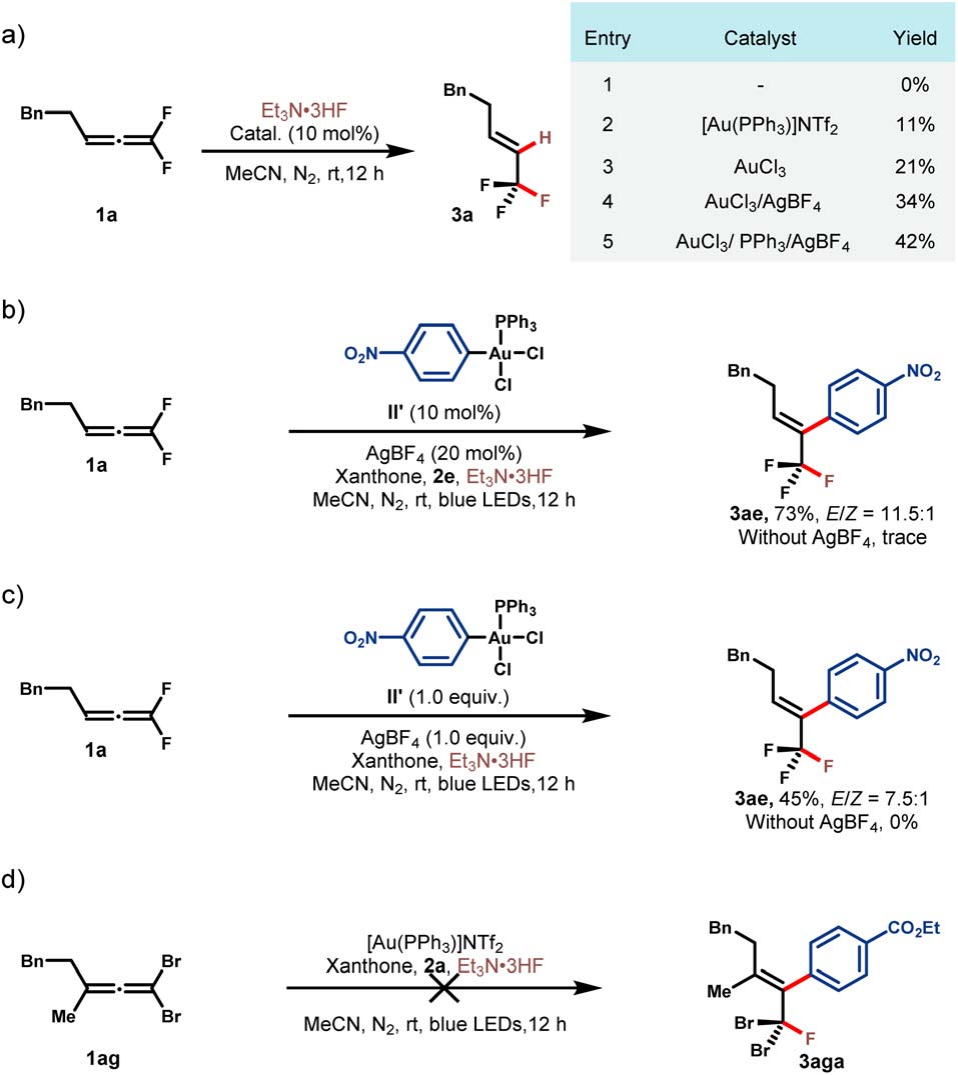
Mechanistic studies. See the ESI† for reaction details.

To shed more light on the reaction mechanism, a series of control experiments were conducted (Scheme 2). At the outset, we tried to figure out whether Au^I^ or Au^III^ activated *gem*-difluoroallene toward nucleophilic fluorination. Control experiments between allene 1a and Et_3_N·3HF indicated that no reaction occurred in the absence of the gold catalyst (Scheme 2a, entry (1)). The addition of either Au^I^ or Au^III^ salt to this reaction led to the formation of hydrofluorination product 3a, showing that both Au^I^ and Au^III^ catalysts could activate the allene substrate, and Au^III^ was superior probably because of its stronger Lewis acidity (Scheme 2a, entries 2 and 3). It was found that the addition of AgBF_4_ and PPh_3_ was beneficial, which demonstrated that cationic Au^III^ could serve as a more powerful π acidic catalyst (Scheme 2a, entries 4 and 5). To further distinguish the activation mode, Ar-Au^III^ species II′ was prepared and employed in the reaction of 1a and 2e. While no reaction occurred in the absence of the silver additive, fluoroarylation product 3ae was obtained in 73% yield with the addition of AgBF_4_ (Scheme 2b). A stoichiometric experiment between Ar–Au^III^ species II′ and 1a could also afford 3ae in modest yield and AgBF_4_ was proved to be necessary for productivity (Scheme 2c). These results further attested the amenability of the cationic high-valent gold species in catalyzing this transformation. Subsequently, a contrasting experiment between 1,1-dibromoallene 1ag and 2a under standard reaction conditions turned out to be unsuccessful, which underlines the key role of the *gem*-difluoro substituents in this reaction (Scheme 2d).^47,48^

**Scheme 3:**
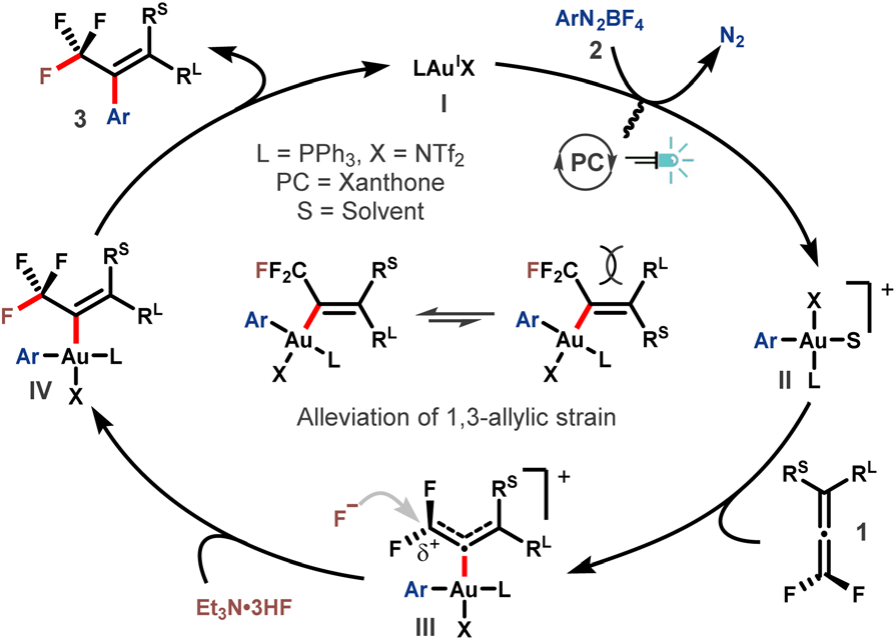
Proposed reaction mechanism.

On the basis of these results, a plausible mechanism was proposed (Scheme 3). The reaction started with oxidative addition of aryldiazonium salt 2 to the Au^I^ catalyst I by the assistance of photoredox catalysis, delivering the actively cationic Ar-Au^III^ species II.^49,50^ Then, the coordination of *gem*-difluoroallene 1 to the Au^III^ center affords intermediate III.^21,51,52^ The electron-withdrawing ability of the two fluorine atoms renders the α-carbon of intermediate III electron-deficient, thus making it susceptible to the ensuing nucleophilic attack by fluoride. Upon regioselective nucleophilic fluorination, trifluoromethyl vinyl gold complex IV is formed. Subsequent reductive elimination provided the desired product 3 accompanied by regeneration of the Au^I^ catalyst. The *E*-selectivity of this transformation might be ascribed to the alleviation of 1,3-allylic interaction in the transition state or intermediate IV.

## Conclusions

In summary, a novel synthetic protocol for the expedient assembly of multi-substituted trifluoromethyl alkenes through the fluoroarylation of *gem*-difluoroallene has been successfully developed. By visible-light-promoted gold catalysis, this reaction features high stereo- and regioselectivities, wide functional group tolerance and broad substrate scope. Furthermore, the fluorine substituent is demonstrated to be of vital importance for the success of this reaction, guaranteeing a charge-controlled nucleophilic fluorination on one hand, and providing extra thermodynamic driving force by the generation of the trifluoromethyl group on the other.

## Data availability

The ESI† contains method description, product characterization data, NMR spectra, and mechanism study details.

## Author contributions

Z.-Q. L. performed most of the experiments and mechanistic study. H.-J. T. did the initial study and examined some substrate scope. C.-Q. W. and Z. W. took part in the preparation of some substrates. C. F. conceived the study, and directed the project. C. F. and C.-Q. W. wrote the manuscript with the assistance of Z.-Q. L.

## Conflicts of interest

There are no conflicts to declare.

## Supplementary Material

SC-015-D3SC06060H-s001
