# Supplementary material for: Multi-substituted trifluoromethyl alkene construction via gold-catalyzed fluoroarylation of gem-difluoroallenes
Source: Chem Sci. 2024 Jan 25;15(10):3524–9. doi: 10.1039/d3sc06060h (PMC10915851; doi:10.1039/d3sc06060h)

Electronic Supplementary Material (ESI) for Chemical Science.
This journal is © The Royal Society of Chemistry 2024

Multi-Substituted Trifluoromethyl Alkene Construction via Gold-Catalyzed Fluoroarylation of *gem*-Difluoroallenes

Zhi-Qiang Li,[a] Hai-Jun Tang,[a] Zaixin Wang,[a] Cheng-Qiang Wang,*[a] Chao Feng*[a]

[a]Technical Institute of Fluorochemistry (TIF), Institute of Advanced Synthesis (IAS), School of Chemistry and Molecular Engineering, State Key Laboratory of Material-Oriented Chemical Engineering, Nanjing Tech University, 30 South Puzhu Road, Nanjing 211816, P. R. China.

iamcfeng@njtech.edu.cn; cqwang08@njtech.edu.cn

Table of Contents

1. General Information…………………………….……..……………………………3
2. Experimental Sections………………………………………………….…………………4
  2.1. Substrate synthesis ……………………..……………..…………………………..4
  2.2 General reaction procedure for the synthesis of substrates **1a-1r**……………………..…..5
  2.3 General reaction procedure for the synthesis of substrates **1s-1ae**……………………..…..6
  2.4 Reaction procedure for the synthesis of **1af**………………………………..……………..7
  2.5 Reaction procedure for the synthesis of **1ag**……………………………………...……..8
3. Preparation of Diazonium Salt **2**……………………………………………………………..9
  3.1 Preparation of aromatic amines……………………..……………………….………..9
  3.2 Preparation of diazonium salt………………………………..……………..…………9
4. Reaction Optimization…………………………………..………………….....………11
  4.1 Reaction optimization of the fluoroarylation of *gem*-difluoroallene……………......…11
5. General Procedure……………………………………………………..…………………16
  5.1 General Procedure for the fluoroarylation of *gem*-difluoroallenes………………..……...16
  5.2 Reaction apparatus……………………...……………………………………………16
6. Characterization of Structurally Novel Compounds……………………………………..….17
  6.1 Characterization of *gem*-difluoroallenes……………………….……….……….…..………17
  6.2 Characterization of the trifluoromethyl alkenes.........................…...25

7. Mechanistic Study……………………………………..………………………….….43
7.1 Control experiment……………………………………………………..………..….43
7.2 Study of **Au$^{(III)}$** intermediate……………………….……………………..………….51
7.2.1 Preparation of **Au$^{(III)}$** intermediate……………………..………………..……....51
7.2.2 Fluoroarylation process with prepared **Au$^{(III)}$** intermediate.……………………….…...51
7.3 TEMPO quenching experiments…………………………...……………………….53
7.4 Ultraviolet and Visible Spectrum………………………………..………………….55
7.5 Emission quenching experiments (Stern-Volmer studies)……………………….……56
7.6 DFT studies…………………………………………………………………….……58
8. Supplementary References…………………………………………………………….66
9. NMR Spectra……………………………………………………………………….67

1. General Information

Unless otherwise noted, all reactions were carried out under dry nitrogen atmosphere.

The following chemicals were purchased as follow: Xanthone (CAS: 90-47-1, Energy Chemical, >98%), Acetonitrile (CAS: 75-05-8, Energy Chemical and J&K, 99.5%, water<0.01%), Et_3N-3HF (CAS: 73602-61-6, J&K, >98%), $[Au(PPh_3)](NTf_2)$ (CAS: 866395-16-6, Sinocompound, >98%), $AgBF_4$ (CAS: 14104-20-2, Energy Chemical, >99%) and other commercial reagents were used directly without further purification. Organic solutions were concentrated under reduced pressure on an IKA RV 10 rotary evaporator. Flash chromatography was performed using Huang hai flash silica gel (200–300 mesh). Thin-layer chromatography (TLC) was performed on Silicycle 250 μm silica gel plates visualized under UV light (254 nm).

HRMS spectra were recorded on a Xevo G2-XS QTof (Waters Corporation). The NMR spectra were recorded using JEOL 400 MHz Fourier-transform NMR spectrometer. Chemical shifts were reported as δ in units of parts per million (ppm) downfield from $SiMe_4$ (δ 0.0). Multiplicities were given as: s (singlet); d (doublet); t (triplet); q (quartet); dd (doublet of doublets); dt (doublet of triplets); m (multiplet) and etc. Coupling constants are reported as a J value in Hz.

2. Experimental Sections

2.1. Substrate synthesis

Allenes 1 examined:

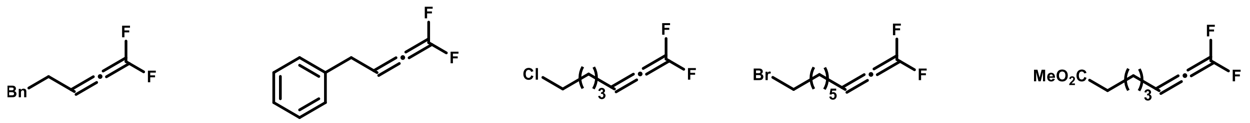

1a 1b 1c 1d 1e

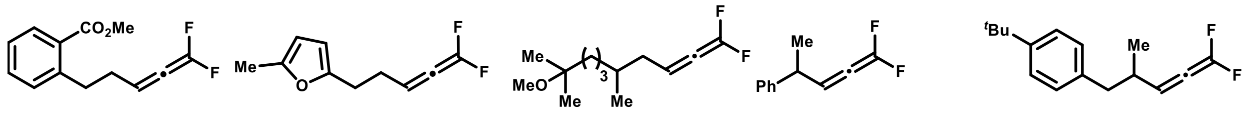

1f 1g 1h 1i 1j

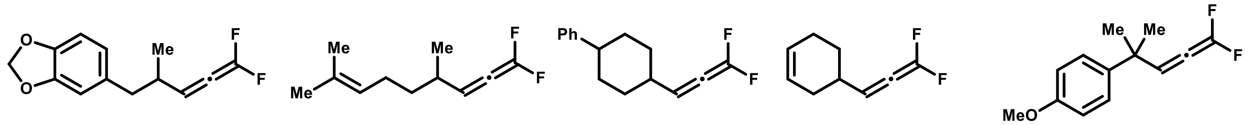

1k 1l 1m 1n 1o

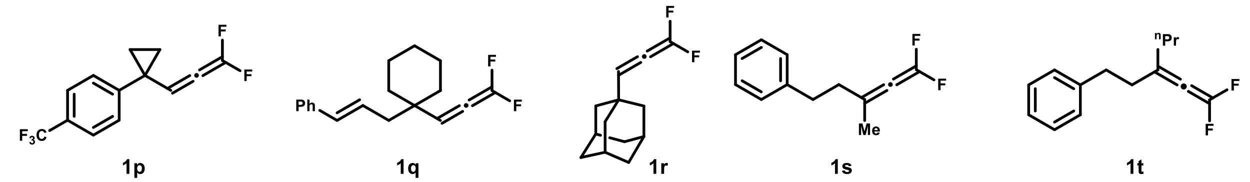

1p 1q 1r 1s 1t

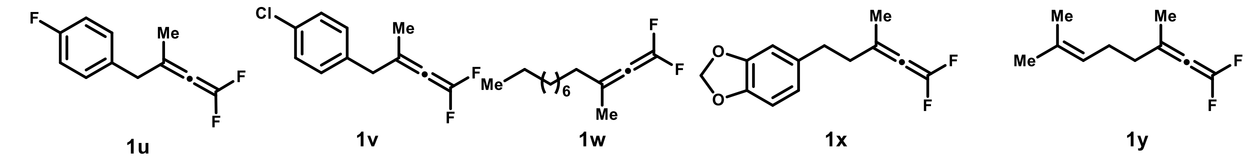

1u 1v 1w 1x 1y

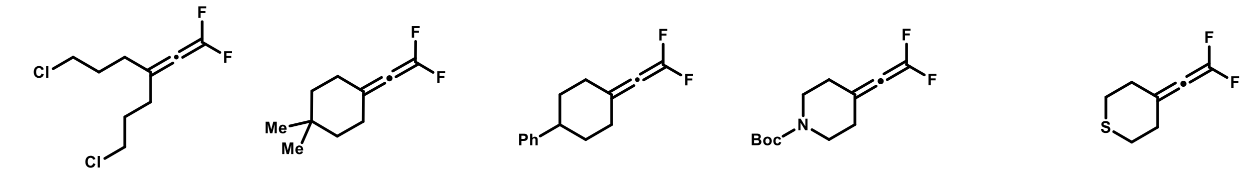

1z 1aa 1ab 1ac 1ad

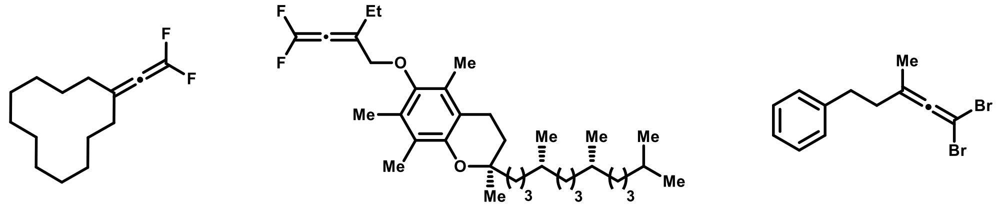

1ae 1af 1ag

1a[1]-**1ae**[1] were synthesized according to the previous literature. **1af**[2] and **1ag**[2] were synthesized according to the previous literature. All characterization data of these compounds are consistent with the literature.

2.2 General reaction procedure for the synthesis of substrates 1a-1r:

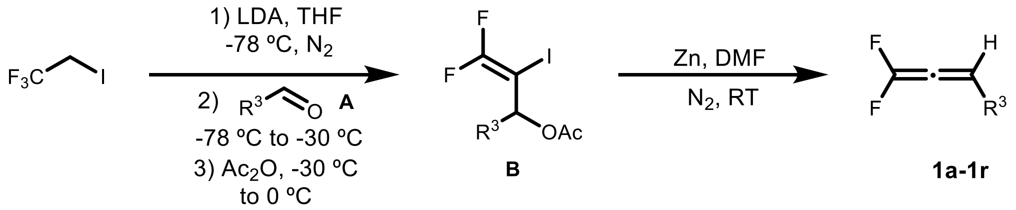

Synthetic procedure: To a solution of NH($^{i-}$Pr)$_2$ (2.1 equiv.) in THF (1.0 M) was added $^{n-}$BuLi (1.6 M or 2.5M in hexane, 2.1 equiv.) over 20 min at 0 ℃ under nitrogen. The resulting solution was allowed to stir for an additional 30 min.

The above reaction mixture was then cooled to -78 ℃ and to this cold LDA solution was added a solution of CF_3CH_2I (1.05 equiv.) in THF (0.5 M) over 20 min. After stirring for 30 min at the same temperature, a solution of aldehyde **A** (1.0 equiv.) in THF (2.0 M) was added over 10 min. The mixture was stirred for an additional 30 min, then slowly warmed to -30 ℃. After the addition of Ac_2O (1.2 equiv.), the mixture was allowed to warm to 0 ℃ over 2 h. The reaction was quenched with sat. aq. NH_4Cl (15 mL), diluted with DCM (30 mL), and filtered through diatomite, the filtrate was extracted with DCM (3 × 50 mL). The combined organic layers were washed with water, brine and dried over anhydrous Na_2SO_4. After removal of the solvent under reduced pressure, the residue was subjected to column chromatography on silica gel (PE/EtOAc) to deliver the corresponding ester **B**.

To a suspension of Zn powder (2.0 equiv.) in DMF (3.0 M) was added a solution of the corresponding ester **B** (3.0 M, 1.0 equiv.) at room temperature under nitrogen. After stirring for overnight, the resulting reaction mixture was diluted with water (50 mL) and petroleum ether (50 mL), filtered through diatomite to remove the excess Zn. The filtrate was extracted with petroleum ether (3 × 30 mL). The combined organic layers were washed with brine and dried over Na_2SO_4. After removal of the solvent under reduced pressure, the residue was purified by column chromatography to deliver the corresponding allene **1a-1r**.

1a-1r were synthetized from the corresponding commercial aldehydes following the above synthetic procedure.

2.3 General reaction procedure for the synthesis of substrates 1s-1ae:

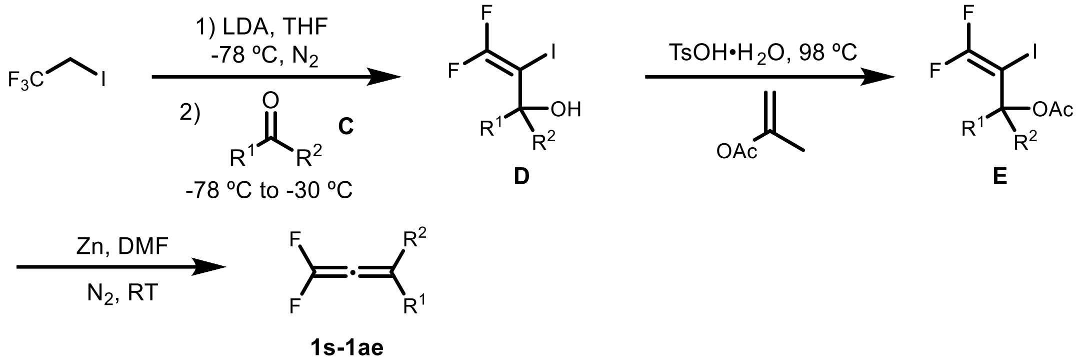

Synthetic procedure: To a solution of $NH(^{i}Pr)_2$ (2.1 equiv.) in THF (1.0 M) was added $^{n}BuLi$ (1.6 M in hexane, 2.1 equiv.) over 20 min at 0 ℃ under nitrogen. The resulting solution was allowed to stir for an additional 30 min.

The above reaction mixture was then cooled to -78 ℃ and to this cold LDA solution was added a solution of CF_3CH_2I (1.05 equiv.) in THF (0.5 M) over 20 min. After stirring for 30 min at the same temperature, a solution of ketone **C** (1.0 equiv.) in THF (2.0 M) was added over 10 min. The mixture was stirred for an additional 30 min, then slowly warmed to -30 ℃. The reaction was quenched with sat. aq. NH_4Cl (15 mL), diluted with EtOAc (30 mL), and filtered through diatomite, the filtrate was extracted with EtOAc (3 × 50 mL). The combined organic layers were washed with water, brine and dried over anhydrous Na_2SO_4. After removal of the solvent under reduced pressure, the residue was subjected to column chromatography on silica gel (PE/EA) to deliver the corresponding alcohol **D**.

To a solution of the alcohol **D** in isopropenyl acetate (2.0 M) was added 4-methylbenzenesulfonic acid monohydrate (0.05 equiv.). After refluxing 12 h and cooling to room temperature, the solvent was removed under reduced pressure and the residue was subjected to column chromatography on silica gel (PE/EA) to deliver the corresponding ester **E**.

To a suspension of Zn powder (2.0 equiv.) in DMF (3.0 M) was added a solution of the corresponding ester **E** (3.0 M, 1.0 equiv.) at room temperature under nitrogen. After stirring for overnight, the resulting reaction mixture was diluted with water (50 mL) and petroleum ether (50 mL), filtered through diatomite to remove the excess Zn. The filtrate was extracted with petroleum ether (3 × 30 mL). The combined organic layers were washed with brine and dried over Na_2SO_4. After removal of the solvent under reduced pressure, the residue was purified by column chromatography to deliver the corresponding allene **1s-1ae**.

1s-1ae were synthetized from the corresponding commercial aldehydes following the above synthetic procedure.

2.4 Reaction procedure for the synthesis of 1af:

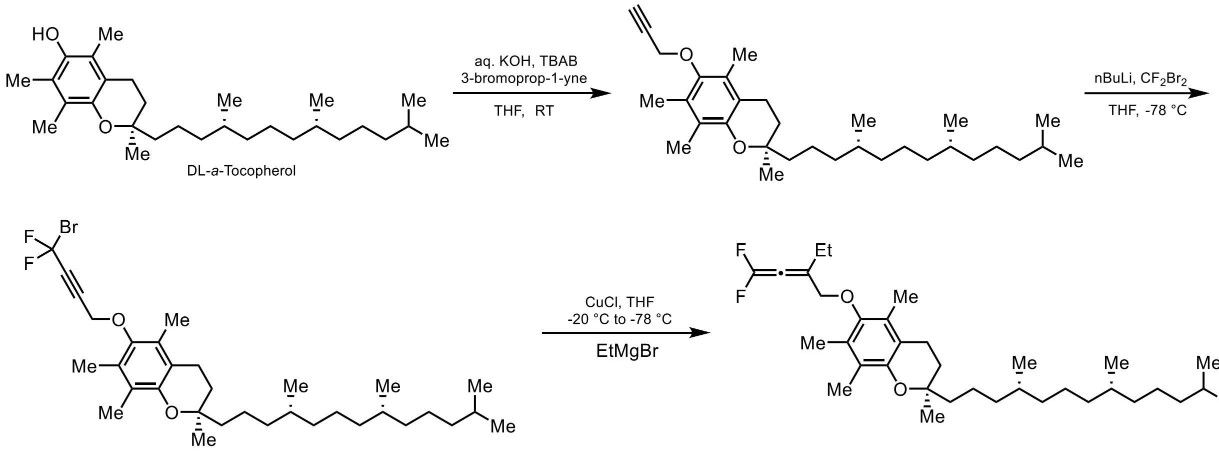

1af

Synthetic procedure: To a mixture of DL-*a*-Tocopherol (1 equiv.), 3-bromoprop-1-yne (1.3 equiv.) and TBAB (0.1 equiv.) in THF (1 M) at room temperature was slowly added aq. KOH (3 equiv., 50 % wt). After stirring 3 h, the solution was diluted with water, extracted with EtOAc, dried over Na_2SO_4, filtered, concentrated under reduced pressure and purified by column chromatography on silica gel to deliver the desired ether.

To a solution of the above obtained ether (1.0 equiv.) in dry THF (1 M), a solution of $^{n-}$BuLi (1.1 equiv., 2.5 M in hexane) was added dropwise at -78 ℃ under nitrogen atmosphere. After the reaction mixture was stirred for 30 min. at -78 ℃, dibromodifluoromethane (1.5 equiv., 5 M in THF) was slowly added to the reaction mixture. After stirring at -78 ℃ for 1 h, the mixture was allowed to warm to room temperature and stirred overnight. The reaction mixture was then quenched with sat. aq. NH_4Cl, extracted with DCM. The combined organic layers were dried over Na_2SO_4, filtered and concentrated. The resulting crude product was purified by flash chromatography to deliver the desired alkyne.

To a solution of CuCl (20 mmol, 4.0 equiv.) in dry THF (25 mL) at -20 ℃ under nitrogen atmosphere was added ethylmagnesium bromide in 5 min. After stirring 20 min, the resulting organo-copper reagent solution was cooled to -78 ℃ immediately and the above obtained alkyne (5 mmol, 5 M in THF, 1.0 equiv.) was added dropwise to the reaction mixture. After the consumption of the starting material, the mixture was quenched with sat. aq. NH_4Cl, extracted

with DCM, dried over Na_2SO_4, filtered, concentrated under reduced pressure and purified by column chromatography on silica gel to deliver the desired product **1af**.

2.5 Reaction procedure for the synthesis of 1ag:

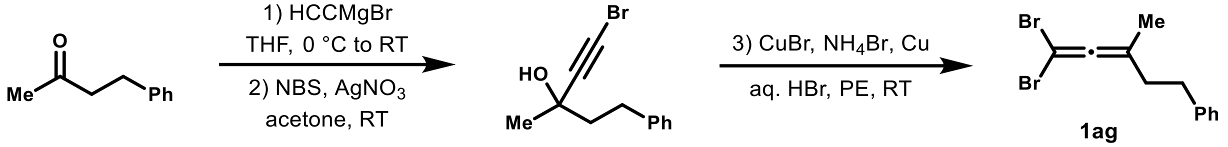

1ag

Synthetic procedure: 1) To a solution of 4-phenylbutan-2-one (1.0 equiv.) in THF (1.0 M) at 0 ℃ was slowly added ethynylmagnesium bromide (1.2 equiv.). The mixture was stirred 12 h at room temperature and then quenched with sat. aq. NH_4Cl, extracted with EA, dried over Na_2SO_4, filtered, and concentrated under reduced pressure. The residue was purified by flash column chromatography on silica gel (PE/EA) to deliver 3-methyl-5-phenylpent-1-yn-3-ol.

2) To a solution of 3-methyl-5-phenylpent-1-yn-3-ol (1.0 equiv.) in acetone (40 mL) was added *N*-bromosuccinimide (1.3 equiv.) and $AgNO_3$ (0.05 equiv.). The flask was wrapped into aluminum foil to prevent light exposure. After stirring for 6.0 h at room temperature, the reaction was concentrated under reduced pressure, diluted with sat. aq. NH_4Cl, extracted with EA, washed with brine, dried over Na_2SO_4, and filtered. Solvent removal and purification by column chromatography afforded 1-bromo-3-methyl-5-phenylpent-1-yn-3-ol.

3) The above 1-bromo-3-methyl-5-phenylpent-1-yn-3-ol (5.0 mmol, 1.0 equiv.) in light petroleum (40 mL) was added to a stirred suspension of copper(I) bromide (0.5 equiv.), ammonium bromide (0.5 equiv.), copper powder (0.3 equiv.), aq. hydrogen bromide (40% w/w, 2.5 equiv.) and the mixture was stirred for 12 h at room temperature. It was then filtered and the filtrate was extracted with light petroleum and dried over Na_2SO_4. Evaporation of the solvent and purification by column chromatography afforded the (5,5-dibromo-3-methylpenta-3,4-dien-1-yl)benzene **1ag**.

1ag[2] was synthesized according to the previous literature. All characterization data of this compound are consistent with the literature.

3. Preparation of Diazonium Salt 2

3.1 Preparation of aromatic amines

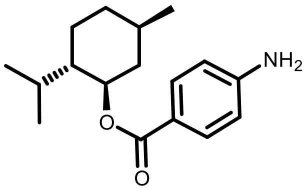

2p

Aromatic amine **2p** was prepared according to literatures.[3,4] Other aromatic amines are commercially available.

3.2 Preparation of diazonium salt

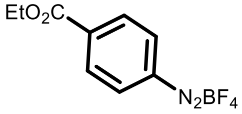

2a

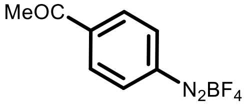

2b

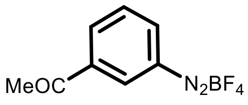

2c

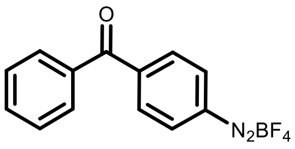

2d

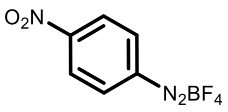

2e

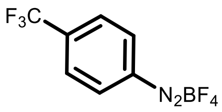

2f

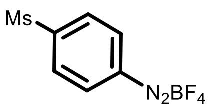

2g

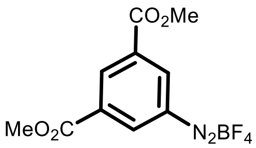

2h

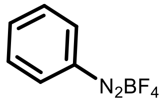

2i

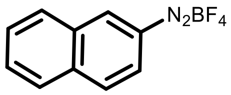

2j

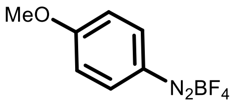

2k

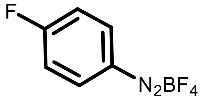

2l

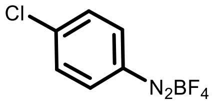

2m

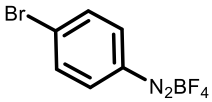

2n

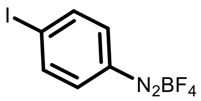

2o

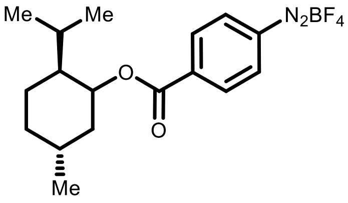

2p

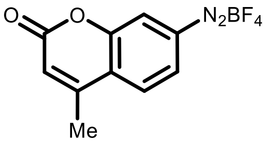

2q

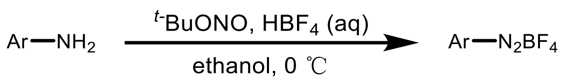

Following the reported method,[3] the aromatic aniline (1.0 equiv, 10 mmol) was dissolved in a mixture of EtOH (3.0 mL) and an aqueous solution of HBF_4 (48%, 2.0 equiv, 20 mmol, 3.7 g) in a 100 mL round-bottom flask with a stir bar, the mixture was stirred for several minutes, ^{t}BuONO (2.0 equiv, 20 mmol, 2.1 g) was added dropwise to the solution at 0 °C, the mixture was stirred for 1 h at room temperature. Finally, Et_2O (20 mL) was added to precipitate the aryldiazonium salt. The solid was filtered off and washed with Et_2O (3 × 10 mL). The aryldiazonium salt was dried in vacuo and directly used without further purification.

4. Reaction Optimization

4.1 Reaction optimization of the fluoroarylation of *gem*-difluoroallenes

Table S1. Screening ratio of 1a and 2a[a]

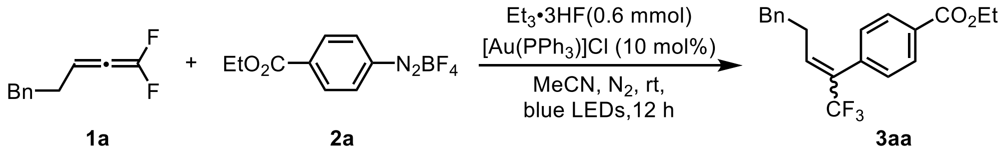

Entry	**1a**	**2a**	Conversion(**1a**)[b]	Yield of **3aa**[c]	*E*/*Z*[d]
1	0.1 mmol	0.1 mmol	84%	40%	7.1:1
2	0.1 mmol	0.15 mmol	92%	49%	6.3:1
3	0.1 mmol	0.2 mmol	100%	53%	8.4:1
4	0.1 mmol	0.3 mmol	95%	30%	9.1:1
5	0.2 mmol	0.1 mmol	100%	36%	8.2:1

[a] Reaction conditions: **1a**, **2a**, Et_3N•3HF (6.0 equiv., 0.6 mmol), $[Au(PPh_3)]Cl$ (10 mol %, 0.01 mmol) were dissolved in acetonitrile (1.0 mL) under nitrogen, then reacted at room temperature for 12 hours under 5 W blue light irradiation. [b] The conversion of substrate **1a** was determined by ^{19}F NMR with 1-iodine-4-trifluoromethyl benzene as internal standard. [c] The yield of compound **3aa** was determined by ^{19}F NMR with 1-iodine-4-trifluoromethylbenzene as internal standard. [d] The *Z*/*E* ratio of compound **3aa** was determined by ^{19}F NMR.

Note: The internal standard was added to the residue obtained after work-up of the reaction mixture to prepare the NMR sample for test.

Table S2. Screening of fluoride source[a]

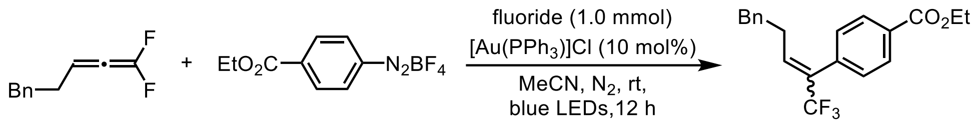

1a **2a** **3aa**

0.1 mmol, 1.0 equiv 0.2 mmol, 2.0 equiv

Entry	Fluoride source	Conversion(**1a**)[b]	Yield of **3aa**[c]	*E*/*Z*[d]
1	$Et_3N·3HF$ (0.6 mmol)	100%	53%	8.4:1
2	$Et_3N·3HF$ (1.0 mmol)	100%	62%	5.2:1
3	$Et_3N·3HF$ (1.5 mmol)	98%	44%	5.2:1
4	KF	100%	5%	*E* only
5	CsF	100%	22%	15.9:1
6	Deoxofluor	100%	11%	15.3:1
7	DAST	100%	33%	6.1:1
8	$^{n-}Bu_4NF$	100%	34%	32:1
9	DMPU·xHF	100%	0%	~
10	Pyridine·xHF	100%	14%	*E* only

[a] Reaction conditions: **1a** (1.0 equiv., 0.1 mmol), **2a** (2.0 equiv., 0.2 mmol), fluoride source, $[Au(PPh_3)]Cl$ (10 mol %, 0.01 mmol) were dissolved in acetonitrile (1.0 mL) under nitrogen, then reacted at room temperature for 12 hours under 5 W blue light irradiation. [b] The conversion of substrate **1a** was determined by ^{19}F NMR with 1-iodine-4-trifluoromethyl benzene as internal standard. [c] The yield of compound **3aa** was determined by ^{19}F NMR with 1-iodine-4-trifluoromethylbenzene as internal standard. [d] The *E*/*Z* ratio of compound **3aa** was determined by ^{19}F NMR.

Table S3. Screening of solvent[a]

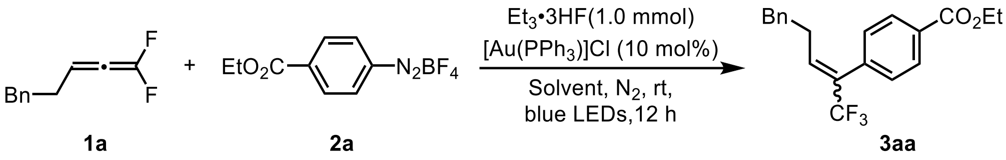

0.1 mmol, 1.0 equiv 0.2 mmol, 2.0 equiv

Entry	Solvent	Conversion(**1a**)[b]	Yield of **3aa**[c]	*E*/*Z*[d]
1	MeCN	100%	62%	5.2:1
2	DCE	91%	23%	3.9:1
3	DCM	91%	24%	3.8:1
4	Dioxane	47%	13%	12.0:1
5	DMF	46%	trace	~
6	THF	100%	NDP	~
7	Acetone	80%	7%	2.5:1

[a] Reaction conditions: **1a** (1.0 equiv., 0.1 mmol), **2a** (2.0 equiv., 0.2 mmol), Et_3N•3HF (10.0 equiv., 1.0 mmol), [Au(PPh_3)]Cl (10 mol %, 0.01 mmol) were dissolved in the corresponding solvent (1.0 mL) under nitrogen, then reacted at room temperature for 12 hours under 5 W blue light irradiation. [b] The conversion of substrate **1a** was determined by ^{19}F NMR with 1-iodine-4-trifluoromethyl benzene as internal standard. [c] The yield of compound **3aa** was determined by ^{19}F NMR with 1-iodine-4-trifluoromethylbenzene as internal standard. [d] The *E*/*Z* ratio of compound **3aa** was determined by ^{19}F NMR.

Table S4. Screening of catalyst[a]

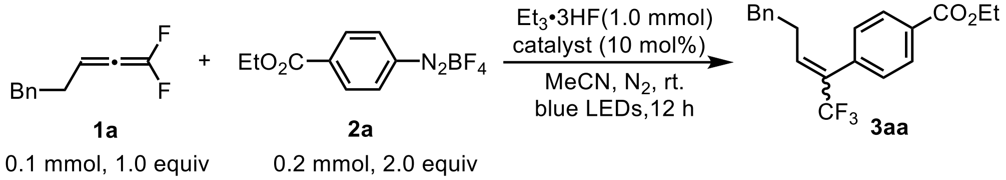

Entry	Catalyst	Conversion(**1a**)[b]	Yield of **3aa**[c]	*E*/*Z*[d]
1	$[Au(PPh_3)]Cl$	100%	62%	5.2:1
2	$[Au(PPh_3)]NTf_2$	100%	56%	8.4:1
3	$[Au(SMe_2)]Cl$	100%	24%	5.0:1
4	Ph_3P+AuCl	95%	9%	4.4:1
5	IPr-AuCl	100%	trace	~
6	[Au(XPhos)]Cl	95%	trace	~
7	Au{P[(2,4-di-^{t}Bu-Ph)O]$_3$}Cl	83%	4%	3:1

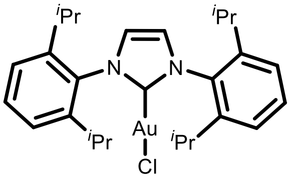

IPr-AuCl

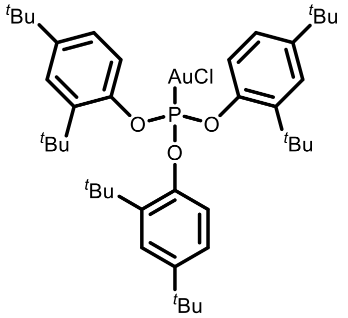

Au{P[(2,4-di-tBu-Ph)O]$_3$}Cl

[a] Reaction conditions: **1a** (1.0 equiv., 0.1 mmol), **2a** (2.0 equiv, 0.2 mmol), Et_3N•3HF (10.0 equiv, 1.0 mmol), gold catalyst (10 mol %, 0.01 mmol) were dissolved in acetonitrile (1.0 mL) under nitrogen, then reacted at room temperature for 12 hours under 5 W blue light irradiation. [b] The conversion of substrate **1a** was determined by ^{19}F NMR with 1-iodine-4-trifluoromethyl benzene as internal standard. [c] The yield of compound **3aa** was determined by ^{19}F NMR with 1-iodine-4-trifluoromethylbenzene as internal standard. [d] The *E*/*Z* ratio of compound **3aa** was determined by ^{19}F NMR.

Table S5. Screening of photocatalyst[a]

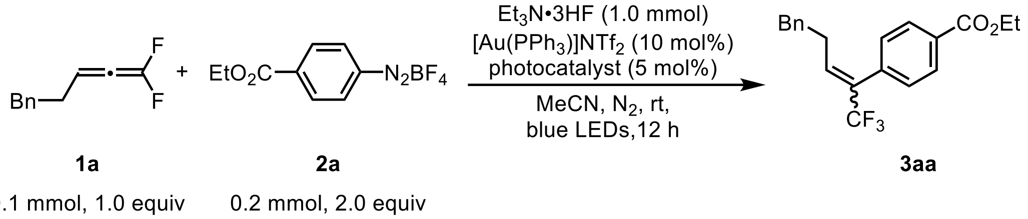

Entry	Photocatalyst	Conversion(**1a**)[b]	Yield of **3aa**[c]	*E*/*Z*[d]
1	$Ir(ppy)_3$	100%	64%	7.6:1
2	$Ru(bpy)_3(PF_6)_2$	100%	72%	7.9:1
3	$[Ir(dF(CF_3)ppy)_2(5,5'\text{-}dCF_3bpy)](PF_6)$	100%	63%	9.8:1
4	Acr^+-Mes	100%	61%	8.3:1
5	EosinY	100%	60%	8.8:1
6	DCA	100%	66%	8.3:1
7	2-CzPN	100%	63%	8.1:1
8	PTH	100%	78%	8.5:1
9	Xanthone	100%	86%	16.1:1
10	Thioxanthen-9-one	100%	55%	10.1:1

[a] Reaction conditions: **1a** (1.0 equiv., 0.1 mmol), **2a** (2.0 equiv, 0.2 mmol), $Et_3N•3HF$ (10.0 equiv, 1.0 mmol), $[Au(PPh_3)]NTf_2$ (10 mol %, 0.01 mmol), Photocatalyst (5 mol %, 0.005 mmol) was dissolved in acetonitrile (1.0 mL) and reacted at room temperature under 5 W blue light for 12 hours. [b] The conversion of substrate **1a** was determined by ^{19}F NMR with 1-iodine-4-trifluoromethyl benzene as internal standard. [c] The yield of compound **3aa** was determined by ^{19}F NMR with 1-iodine-4-trifluoromethylbenzene as internal standard. [d] The *E*/*Z* ratio of compound **3aa** was determined by ^{19}F NMR.

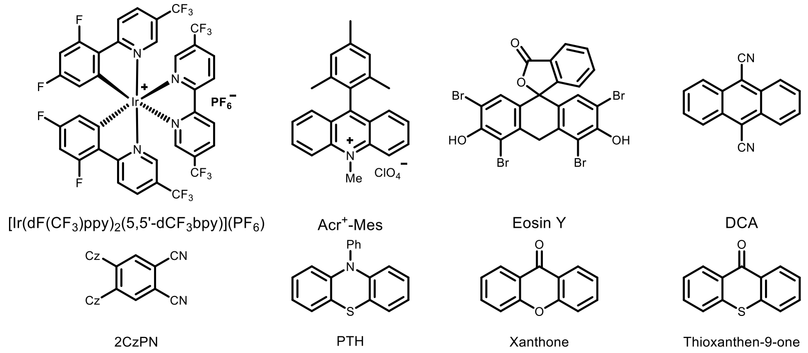

5. General Procedure

5.1 General procedure for the fluoroarylation of *gem*-difluoroallenes

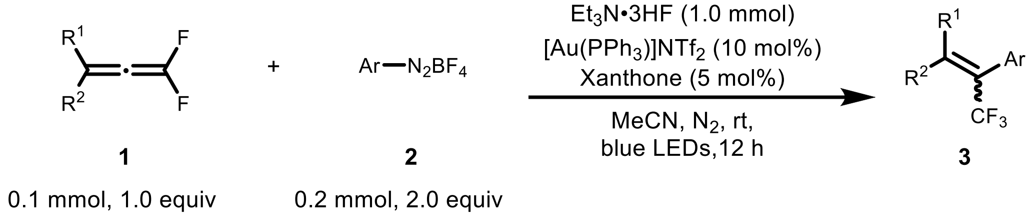

To an oven-dried 10 mL Schlenk Tube equipped with a stir bar add $[Au(PPh_3)]NTf_2$ (10 mol%, 0.01 mmol) in the glove box, then remove the reaction tube out of the glove box, under the atmosphere of nitrogen, Xanthone (5 mol%, 0.005 mmol), *gem*-difluoroallene **1** (1.0 equiv, 0.1 mmol), aryl diazonium **2** (2.0 equiv, 0.2 mmol), MeCN (1.0 mL) and Et_3N-3HF (10.0 equiv, 1.0 mmol) were added. After stirring 12 hours at room temperature under 5 W blue light irradiation, use TLC to monitor the reaction, then the reaction mixture was passed through a short pad of celite and rinsed with EtOAc. The filtrate was evaporated under reduced pressure and the crude residue was purified by column chromatography on silica gel (EtOAc/PE = 1:20 to 1:10) to afford the desired fluoroarylation product **3**.

5.2 Reaction apparatus

Irradiation of visible light was performed with a 5 W Blue LED lamp. With the help of fan, the heating effect from LED irradiation conditions above was minimal. With 3 hours irradiation, the increase of temperature was less than 5 °C. The distance between vial and lamp was approximately 2 cm.

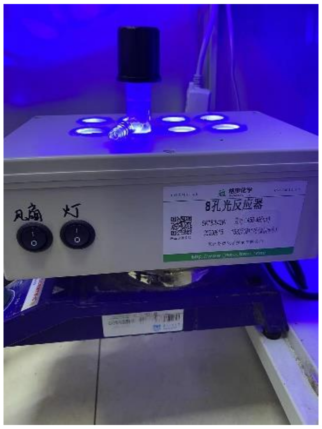
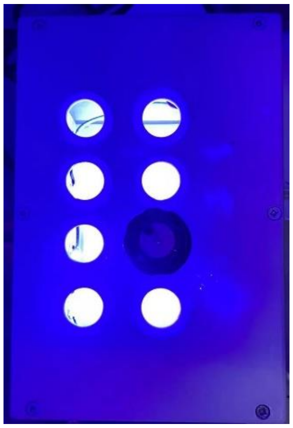
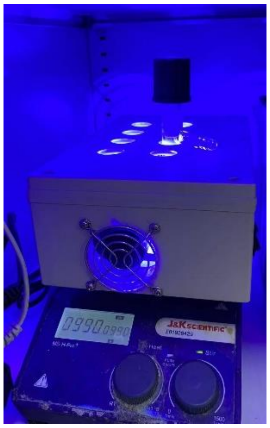

6. Characterization of Structurally Novel Compounds

6.1 Characterization of *gem*-difluoroallenes

(5,5-difluoropenta-3,4-dien-1-yl)benzene

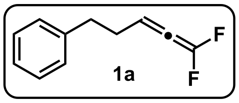

^{1}H NMR (400 MHz, CDCl$_3$): δ 7.35 – 7.30 (m, 2H), 7.25 – 7.20 (m, 3H), 6.49 (tt, *J* = 6.1, 2.4 Hz, 1H), 2.85 – 2.80 (t, 2H), 2.63 – 2.54 (m, 2H);**^{19}F NMR (376 MHz, CDCl$_3$):** δ -101.26(s, 2F); **^{13}C NMR (101 MHz, CDCl$_3$):** δ 170.19(t, *J* = 36.1 Hz), 155.49, 152.89, 150.30 (t, *J* = 260.0 Hz), 140.75, 128.61, 128.51, 126.33, 121.59, 121.53, 121.47 (t, *J* = 5.6 Hz), 33.87. **HRMS (ESI, m/z):** calculated for $C_{11}H_{10}F_2$ [M]$^+$: 180.0751, found: 180.0750.

(4,4-difluorobuta-2,3-dien-1-yl)benzene

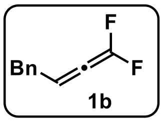

^{1}H NMR (400 MHz, CDCl$_3$): δ 7.39–7.35 (m, 2H), 7.32–7.27 (m, 1H), 7.27–7.22 (m, 2H), 6.56 (tt, *J* = 6.9, 2.3 Hz, 1H), 3.60 (dt, *J* = 6.9, 4.7 Hz, 2H); **^{19}F NMR (376 MHz, CDCl$_3$):** δ -101.6 (s, 2F); **^{13}C NMR (101 MHz, CDCl$_3$):** δ 171.0 (t, *J* = 36.4 Hz), 152.4 (t, *J* = 262.0 Hz), 137.3, 128.7, 126.8, 120.6 (t, *J* = 5.8 Hz), 38.9; **HRMS (ESI, m/z):** calculated for $C_{10}H_9F_2$ [M+H]$^+$: 167.0672, found: 167.0676.

7-chloro-1,1-difluorohepta-1,2-diene

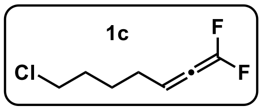

^{1}H NMR (400 MHz, CDCl$_3$): δ 6.44 (tt, *J* = 6.2, 2.4 Hz, 1H), 3.54 (t, *J* = 6.5 Hz, 2H), 2.32–2.24 (m, 2H), 1.86–1.77 (m, 2H), 1.70–1.62 (m, 2H); **^{19}F NMR (376 MHz, CDCl$_3$):** δ -102.0 (t, *J* = 5.6 Hz, 2F); **^{13}C NMR (101 MHz, CDCl$_3$):** δ 169.7 (t, *J* = 36.1 Hz), 152.6 (t, *J* = 260.5 Hz), 121.6 (t, *J* = 5.5 Hz), 44.5, 31.6, 31.3, 24.6; **HRMS (ESI, m/z):** calculated for $C_7H_{10}ClF_2$ [M+H]$^+$: 167.0439, found: 167.0434.

9-bromo-1,1-difluoronona-1,2-diene

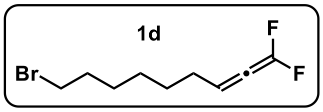

^{1}H NMR (400 MHz, CDCl$_3$): δ 6.43 (tt, *J* = 6.3, 2.4 Hz, 1H), 3.40 (t, *J* = 6.8 Hz, 2H), 2.29–2.20 (m, 2H), 1.90–1.81 (m, 2H), 1.55–1.34 (m, 6H); **^{19}F NMR (376 MHz, CDCl$_3$):** δ -102.1 (s, 2F); **^{13}C NMR (101 MHz, CDCl$_3$):** δ 169.4 (t, *J* = 35.7 Hz), 152.5 (t, *J* = 260.0 Hz), 122.2 (t, *J* = 5.6 Hz), 33.8, 32.6, 32.0, 28.0, 27.8, 27.2; **HRMS (ESI, m/z):** calculated for $C_9H_{14}BrF_2$ [M+H]$^+$: 239.0247, found: 239.0245.

methyl 8,8-difluoroocta-6,7-dienoate

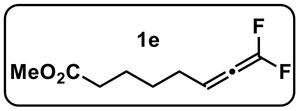

^{1}H NMR (400 MHz, CDCl$_3$): δ 6.41 (tt, *J* = 6.2, 2.3 Hz, 1H), 3.64 (s, 3H), 2.31 (t, *J* = 7.3 Hz, 2H), 2.28–2.20 (m, 2H), 1.70–1.60 (m, 2H), 1.55–1.45 (m, 2H); **^{19}F NMR (376 MHz, CDCl$_3$):** δ -102.1 (t, *J* = 5.6 Hz, 2F); **^{13}C NMR (101**

MHz, $CDCl_3$): δ 173.8, 169.6 (t, *J* = 36.2 Hz), 152.5 (t, *J* = 260.5 Hz), 121.8 (t, *J* = 5.5 Hz), 51.4, 33.6, 31.8, 26.9, 24.1; **HRMS (ESI, m/z):** calculated for $C_9H_{13}F_2O_2$ $[M+H]^+$: 191.0884, found: 191.0885.

methyl 2-(5,5-difluoropenta-3,4-dien-1-yl)benzoate

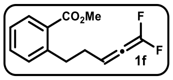

1f[1] were synthesized according to the previous literature. All characterization data of these compounds are consistent with the literature.

2-(5,5-difluoropenta-3,4-dien-1-yl)-5-methylfuran

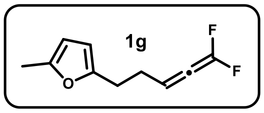

^{1}H NMR (400 MHz, $CDCl_3$): δ 6.48 (ddd, *J* = 8.5, 6.0, 2.4 Hz, 1H), 5.89 (d, *J* = 3.1 Hz, 1H), 5.85 (d, *J* = 1.8 Hz, 1H), 2.77 (t, *J* = 7.4 Hz, 2H), 2.62 – 2.53 (m, 2H), 2.25 (s, 3H). **^{19}F NMR (376 MHz, $CDCl_3$):** δ -101.64 (t, *J* = 6.1 Hz). **^{13}C NMR (101 MHz, $CDCl_3$):** δ 170.24 (t, *J* = 35.5 Hz), 155.57, 152.97, 150.80 (t, *J* = 259.9 Hz), 152.46, 121.20, 121.15, 121.09 (t, *J* = 5.6 Hz), 106.25, 105.96, 30.82, 26.18, 13.58. **HRMS (ESI, m/z):** calculated for $C_{10}H_{10}F_2O$ $[M+H]^+$: 185.0778, found: 185.0789.

1,1-difluoro-9-methoxy-5,9-dimethyldeca-1,2-diene

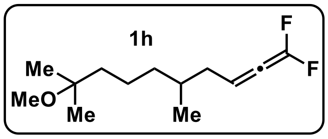

^{1}H NMR (400 MHz, $CDCl_3$): δ 6.34 (tt, *J* = 7.1, 2.4 Hz, 1H), 3.13 (s, 3H), 2.28–2.17 (m, 1H), 2.10–1.99 (m, 1H), 1.72–1.60 (m, 1H), 1.43–1.23 (m, 5H), 1.21–1.13 (m, 1H), 1.10 (s, 6H), 0.91 (d, *J* = 6.7 Hz, 3H); **^{19}F NMR (376 MHz, $CDCl_3$):** δ -102.7 (s, 2F); **^{13}C NMR (101 MHz, $CDCl_3$):** δ 170.2 (t, *J* = 35.8 Hz), 152.1 (t, *J* = 260.0 Hz), 121.0 (t, *J* = 5.6 Hz), 74.4, 49.0, 39.9, 39.6, 36.8, 32.2, 24.9, 21.1, 19.3; **HRMS (ESI, m/z):** calculated for $C_{13}H_{23}F_2O$ $[M+H]^+$: 233.1717, found: 233.1719.

(5,5-difluoropenta-3,4-dien-2-yl)benzene

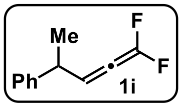

^{1}H NMR (400 MHz, $CDCl_3$): δ 7.44–7.38 (m, 2H), 7.35–7.27 (m, 3H), 6.66–6.64 (m, 1H), 3.78–3.68 (m, 1H), 1.48 (d, *J* = 6.9 Hz, 3H); **^{19}F NMR (376 MHz, $CDCl_3$):** δ -100.6 (d, *J* = 120.0 Hz, 1F), -101.2 (d, *J* = 120.0 Hz, 1F); **^{13}C NMR (101 MHz, $CDCl_3$):** δ 169.1 (t, *J* = 36.3 Hz), 153.5 (t, *J* = 260.6 Hz), 143.0, 128.7, 127.3, 126.9, 126.2 (t, *J* = 5.5 Hz), 42.5, 20.3; **HRMS (ESI, m/z):** calculated for $C_{11}H_{11}F_2$ $[M+H]^+$: 181.0829, found: 181.0832.

1-(tert-butyl)-4-(5,5-difluoro-2-methylpenta-3,4-dien-1-yl)benzene

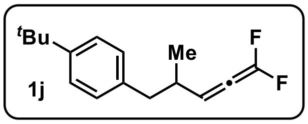

^{1}H NMR (400 MHz, $CDCl_3$): δ 7.39 (d, *J* = 8.4 Hz, 2H), 7.17 (d, *J* = 8.4 Hz, 2H), 6.52–6.49 (m, 1H), 2.86–2.81 (m, 1H), 2.77–2.71 (m, 1H), 2.68– 2.63 (m, 1H), 1.39 (s, 9H), 1.14 (d, *J* = 6.5 Hz, 3H); **^{19}F NMR (376 MHz, $CDCl_3$):** δ -101.5 (s, 2F); **^{13}C NMR (101 MHz, $CDCl_3$):** δ 168.6 (t, *J* = 36.1 Hz),

153.4 (t, J = 260.8 Hz), 149.1, 136.4, 128.8, 127.1 (t, J = 5.6 Hz), 125.2, 41.6, 38.3, 34.4, 31.4, 18.5; **HRMS (ESI, m/z):** calculated for $C_{16}H_{21}F_2$ $[M+H]^+$: 251.1611, found: 251.1605.

5-(5,5-difluoro-2-methylpenta-3,4-dien-1-yl)benzo[d][1,3]dioxole

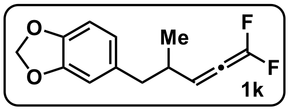

^{1}H NMR (400 MHz, CDCl$_3$): δ 6.75 (d, J = 7.9 Hz, 1H), 6.67 (d, J = 1.6 Hz, 1H), 6.62 (dd, J = 7.9, 1.7 Hz, 1H), 6.45–6.43 (m, 1H), 5.94 (s, 2H), 2.75–2.71 (m, 1H), 2.67–2.59 (m, 1H), 2.57–2.52 (m, 1H), 1.07 (d, J = 6.5 Hz, 3H); **^{19}F NMR (376 MHz, CDCl$_3$):** δ -101.5 (s, 2F); **^{13}C NMR (101 MHz, CDCl$_3$):** δ 168.8 (t, J = 36.3 Hz), 153.5 (t, J = 261.5 Hz), 147.7, 146.1, 133.4, 127.0 (t, J = 5.6 Hz), 122.1, 109.4, 108.2, 100.9, 41.9, 38.6, 18.5; **HRMS (ESI, m/z):** calculated for $C_{13}H_{13}F_2O_2$ $[M+H]^+$: 239.0884, found: 239.0886.

1,1-difluoro-4,8-dimethylnona-1,2,7-triene

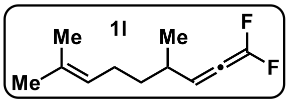

^{1}H NMR (400 MHz, CDCl$_3$): δ 6.40 (dt, J = 6.3, 2.5 Hz, 1H), 5.13–5.08 (m, 1H), 2.44–2.32 (m, 1H), 2.05 (q, J = 7.5 Hz, 2H), 1.71 (s, 3H), 1.62 (s, 3H), 1.58–1.36 (m, 2H), 1.09 (d, J = 6.8 Hz, 3H); **^{19}F NMR (376 MHz, CDCl$_3$):** δ -101.9 (s, 2F); **^{13}C NMR (101 MHz, CDCl$_3$):** δ 168.2 (t, J = 35.8 Hz), 153.0 (t, J = 260.2 Hz), 132.0, 127.6 (t, J = 5.5 Hz), 123.9, 36.3, 35.8, 25.6, 25.5, 18.9, 17.6; **HRMS (ESI, m/z):** calculated for $C_{11}H_{17}F_2$ $[M+H]^+$: 187.1298, found: 187.1297.

(4-(3,3-difluoropropa-1,2-dien-1-yl)cyclohexyl)benzene

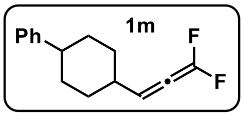

^{1}H NMR (400 MHz, CDCl$_3$): δ 7.39–7.32 (m, 2H), 7.29–7.22 (m, 3H), 6.50 (dt, J = 5.6, 2.3 Hz, 1H), 2.55 (m, 1H), 2.39– 2.23 (m, 1H), 2.08– 1.99 (m, 4H), 1.88–1.52 (m, 3H), 1.44–1.34 (m, 1H); **^{19}F NMR (376 MHz, CDCl$_3$):** δ -101.6 (s, 2F); **^{13}C NMR (101 MHz, CDCl$_3$):** δ 168.1 (t, J = 35.8 Hz), 153.2 (t, J = 259.8 Hz), 146.9, 128.4, 127.0 (t, J = 5.3 Hz), 126.8, 126.0, 43.7, 40.3, 33.6, 31.7; **HRMS (ESI, m/z):** calculated for $C_{15}H_{17}F_2$ $[M+H]^+$: 235.1298, found: 235.1295.

4-(3,3-difluoropropa-1,2-dien-1-yl)cyclohex-1-ene

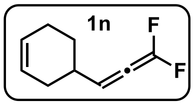

^{1}H NMR (400 MHz, CDCl$_3$): δ 6.48 (dt, J = 5.3, 2.5 Hz, 1H), 5.76–5.63 (m, 2H), 2.58–2.44 (m, 1H), 2.28–2.17 (m, 1H), 2.14–2.09 (m, 2H), 2.03–1.87 (m, 2H), 1.56–1.41 (m, 1H); **^{19}F NMR (376 MHz, CDCl$_3$):** δ -101.4 (t, J = 122.1 Hz, 1F), -101.9 (t, J = 122.1 Hz, 1F); **^{13}C NMR (101 MHz, CDCl$_3$):** δ 168.5 (t, J = 36.2 Hz), 153.2 (t, J = 259.9 Hz), 126.8, 126.6 (t, J = 5.4 Hz), 125.2, 36.6, 29.8, 27.3, 24.5; **HRMS (ESI, m/z):** calculated for $C_9H_{11}F_2$ $[M+H]^+$: 157.0829, found: 157.0827.

1-(5,5-difluoro-2-methylpenta-3,4-dien-2-yl)-4-methoxybenzene

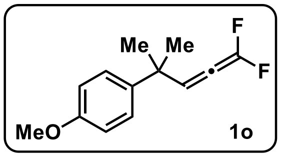

^{1}H NMR (400 MHz, CDCl$_3$): δ 7.32 (d, *J* = 8.9 Hz, 2H), 6.93 (d, *J* = 8.9 Hz, 2H), 6.57 (t, *J* = 2.5 Hz, 1H), 3.84 (s, 3H), 1.51 (s, 6H); **^{19}F NMR (376 MHz, CDCl$_3$):** δ -100.5 (s, 2F); **^{13}C NMR (101 MHz, CDCl$_3$):** δ 166.6 (t, *J* = 36.3 Hz), 158.1, 153.2 (t, *J* = 261.4 Hz), 138.2, 131.3 (t, *J* = 5.5 Hz), 127.0, 113.7, 55.1, 41.7, 28.1; **HRMS (ESI, m/z):** calculated for $C_{13}H_{15}F_2O$ [M+H]$^+$: 225.1091, found: 225.1096.

1-(1-(3,3-difluoropropa-1,2-dien-1-yl)cyclopropyl)-4-(trifluoromethyl)benzene

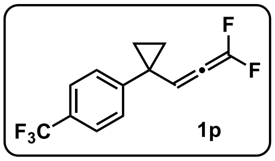

^{1}H NMR (400 MHz, CDCl$_3$): δ 7.60 (d, *J* = 8.6 Hz, 2H), 7.44 (d, *J* = 8.1 Hz, 2H), 6.37 (t, *J* = 2.2 Hz, 1H), 1.36–1.27 (m, 4H); **^{19}F NMR (376 MHz, CDCl$_3$):** δ -62.4 (s, 3F), -98.8 (s, 2F); **^{13}C NMR (101 MHz, CDCl$_3$):** δ 167.2 (t, *J* = 36.4 Hz), 152.3 (t, *J* = 262.3 Hz), 145.6, 129.4, 129.3 (q, *J* = 32.7 Hz), 128.6 (t, *J* = 5.3 Hz), 125.3 (q, *J* = 3.7 Hz), 124.2 (q, *J* = 272.0 Hz), 28.3, 16.5; **HRMS (ESI, m/z):** calculated for $C_{13}H_{10}F_5$ [M+H]$^+$: 261.0703, found: 261.0704.

(*E*)-(3-(1-(3,3-difluoropropa-1,2-dien-1-yl)cyclohexyl)prop-1-en-1-yl)benzene

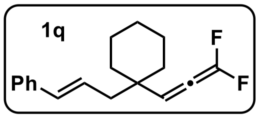

^{1}H NMR (400 MHz, CDCl$_3$): δ 7.45–7.35 (m, 4H), 7.30–7.25 (m, 1H), 6.46 (d, *J* = 15.7 Hz, 1H), 6.38 (s, 1H), 6.24 (dt, *J* = 15.4, 7.5 Hz, 1H), 2.37 (d, *J* = 7.5 Hz, 2H), 1.77–1.58 (m, 5H), 1.56–1.35 (m, 5H); **^{19}F NMR (376 MHz, CDCl$_3$):** δ -101.8 (s, 2F); **^{13}C NMR (101 MHz, CDCl$_3$):** δ 169.1 (t, *J* = 35.8 Hz), 153.9 (t, *J* = 260.9 Hz), 137.4, 133.0, 129.7 (t, *J* = 5.5 Hz), 128.5, 127.1, 126.0, 125.4, 44.5, 42.5, 35.4, 26.0, 22.2; **HRMS (ESI, m/z):** calculated for $C_{18}H_{21}F_2$ [M+H]$^+$: 275.1611, found: 275.1617.

1-(3,3-difluoropropa-1,2-dien-1-yl)adamantane

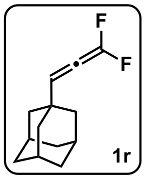

^{1}H NMR (400 MHz, CDCl$_3$): δ 6.25 (t, *J* = 2.5 Hz, 1H), 2.03 (s, 3H), 1.79–1.62 (m, 12H); **^{19}F NMR (376 MHz, CDCl$_3$):** δ -101.3 (s, 2F); **^{13}C NMR (101 MHz, CDCl$_3$):** δ 166.2 (t, *J* = 36.4 Hz), 153.5 (t, *J* = 259.8 Hz), 132.3 (t, *J* = 5.3 Hz), 41.2, 37.3, 36.5, 28.2; **HRMS (ESI, m/z):** calculated for $C_{13}H_{17}F_2$ [M+H]$^+$: 211.1298, found: 211.1295.

(5,5-difluoro-3-methylpenta-3,4-dien-1-yl)benzene

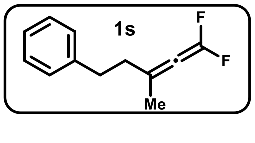

^{1}H NMR (400 MHz, CDCl$_3$): δ 7.38–7.33 (m, 2H), 7.30–7.23 (m, 3H), 2.85 (t, *J* = 7.5 Hz, 2H), 2.59–2.52 (m, 2H), 2.02 (t, *J* = 5.0 Hz, 3H); **^{19}F NMR (376 MHz, CDCl$_3$):** δ -100.3 (s, 2F); **^{13}C NMR (101 MHz, CDCl$_3$):** δ 163.0

(t, J = 34.4 Hz), 150.4 (t, J = 259.0 Hz), 141.0, 132.3 (t, J = 6.1 Hz), 128.4, 128.3, 126.1, 38.7, 33.4, 22.8; **HRMS (ESI, m/z):** calculated for $C_{12}H_{13}F_2$ $[M+H]^+$: 195.0985, found: 195.0977.

(3-(2,2-difluorovinylidene)hexyl)benzene

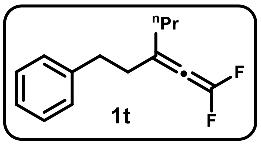

^{1}H NMR (400 MHz, CDCl$_3$): δ 7.33 (t, J = 7.2 Hz, 2H), 7.26 – 7.20 (m, 3H), 2.85 – 2.79 (m, 2H), 2.57 – 2.49 (m, 2H), 2.26 – 2.17 (m, 2H), 1.60 – 1.51 (m, 2H), 0.96 (t, J = 7.4 Hz, 3H). **^{19}F NMR (376 MHz, CDCl$_3$):** δ -100.01 (p, J = 5.8 Hz). **^{13}C NMR (101 MHz, CDCl$_3$):** δ 162.71, 162.34, 162.02 (t, J = 34.67 Hz), 154.71, 151.93, 149.75 (t, J = 259.12 Hz), 141.27, 137.30, 137.24, 137.19 (t, J = 6.03 Hz), 128.53, 128.43, 126.17, 38.41, 37.77, 33.56, 20.45, 13.63. **HRMS (ESI, m/z):** calculated for $C_{14}H_{16}F_2$ $[M]^+$: 222.1220, found: 222.1204.

1-(4,4-difluoro-2-methylbuta-2,3-dien-1-yl)-4-fluorobenzene

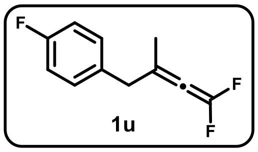

^{1}H NMR (400 MHz, CDCl$_3$): δ 7.17 – 7.10 (m, 2H), 7.04 – 6.97 (m, 2H), 3.46 (t, J = 4.6 Hz, 2H), 1.92 (t, J = 5.0 Hz, 3H). **^{19}F NMR (376 MHz, CDCl$_3$):** δ -100.80 (s, 2F), -116.01 (s, 1F). **^{13}C NMR (101 MHz, CDCl$_3$):** δ 165.57, 165.22, 164.87 (t, J = 36.29Hz), 152.66, 150.08, 147.50 (t, J = 261.04 Hz), 131.5, 131.45, 131.38 (t, J = 6.44 Hz), 130.60, 130.52, 115.55, 115.34, 43.26, 22.07. **HRMS (ESI, m/z):** calculated for $C_{11}H_9F_3$ $[M+H]^+$: 199.0735, found: 199.0739.

1-chloro-4-(4,4-difluoro-2-methylbuta-2,3-dien-1-yl)benzene

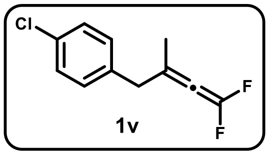

^{1}H NMR (400 MHz, CDCl$_3$): δ 7.31 – 7.27 (m, 2H), 7.14 – 7.09 (m, 2H), 3.46 (t, J = 4.5 Hz, 2H), 1.93 (t, J = 5.0 Hz, 3H). **^{19}F NMR (376 MHz, CDCl$_3$):** δ -100.63 (h, J = 4.5 Hz). **^{13}C NMR (101 MHz, CDCl$_3$):** δ 165.8, 165.4, 165.1 (t, J = 34.34 Hz), 157.7, 150.08, 147.5 (t, J = 258.58 Hz), 135.78, 132.76, 131.1, 131.02, 130.96 (t, J = 5.77 Hz), 130.44, 128.76, 43.39, 22.13. **HRMS (ESI, m/z):** calculated for $C_{11}H_9ClF_2$ $[M+H]^+$: 215.0439, found: 215.0446.

1,1-difluoro-3-methyldodeca-1,2-diene

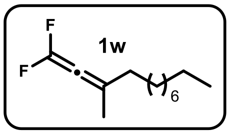

^{1}H NMR (400 MHz, CDCl$_3$): δ 2.17 (tt, J = 7.6, 5.6 Hz, 2H), 1.93 (t, J = 5.0 Hz, 3H), 1.46 (t, J = 7.3 Hz, 2H), 1.31 – 1.24 (m, 12H), 0.90 – 0.86 (m, 3H). **^{19}F NMR (376 MHz, CDCl$_3$):** δ -101.13 (p, J = 6.3 Hz). **^{13}C NMR (101 MHz, CDCl$_3$):** δ 162.83, 162.49, 162.14 (t, J = 34.05 Hz), 152.69, 150.12, 147.56 (t, J = 258.14 Hz), 133.39, 133.34, 133.27 (t, J = 6.24 Hz), 37.23, 31.97, 29.60, 29.51, 29.38, 29.08, 27.09, 22.78, 14.20. **HRMS (ESI, m/z):** calculated for $C_{13}H_{22}F_2$ $[M]^+$: 216.1690, found: 216.1694.

5-(5,5-difluoro-3-methylpenta-3,4-dien-1-yl)benzo[d][1,3]dioxole

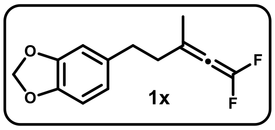

^{1}H NMR (400 MHz, CDCl$_3$): δ 6.76 – 6.61 (m, 3H), 5.93 (s, 2H), 2.71 (t, *J* = 7.9 Hz, 2H), 2.46 (dt, *J* = 12.8, 6.5 Hz, 2H), 1.97 (t, *J* = 5.0 Hz, 3H). **^{19}F NMR (376 MHz, CDCl$_3$):** δ -100.26 (h, *J* = 4.6 Hz). **^{13}C NMR (101 MHz, CDCl$_3$):** 163.5, 163.16, 162.8 (t, *J* = 35.04 Hz), 153.00, 150.43, 147.9(t, *J* = 258.55 Hz), 147.68, 145.88, 134.94, 132.37, 132.30, 132.25(t, *J* = 6.19 Hz), 121.20, 108.83, 108.30, 100.93, 39.09, 33.22, 23.10. **HRMS (ESI, m/z):** calculated for $C_{13}H_{13}F_2O_2$ [M]$^+$: 238.0805, found: 238.0792.

1,1-difluoro-3,7-dimethylocta-1,2,6-triene

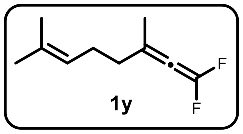

^{1}H NMR (400 MHz, CDCl$_3$): δ 5.07 (tt, *J* = 6.8, 1.4 Hz, 1H), 2.17 (tt, *J* = 11.2, 5.1 Hz, 4H), 1.94 (t, *J* = 5.0 Hz, 3H), 1.68 (s, 3H), 1.61 (s, 3H). **^{19}F NMR (376 MHz, CDCl$_3$):** δ -100.82 (dt, *J* = 8.8, 4.5 Hz). **^{13}C NMR (101 MHz, CDCl$_3$):** δ 163.2, 162.9, 162.5 (t, *J* = 34.91 Hz), 152.9, 150.35, 147.77 (t, *J* = 259.5 Hz), 132.9, 132.85, 132.79 (t, *J* = 6.70 Hz), 132.71, 123.09, 123.02, 37.25, 25.78, 22.94, 17.77. **HRMS (ESI, m/z):** calculated for $C_{10}H_{14}F_2$ [M]$^+$: 172.1064, found: 172.1080.

1,7-Dichloro-4-(2,2-difluorovinylidene)heptane

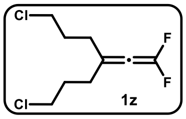

^{1}H NMR (400 MHz, CDCl$_3$): δ 3.56 (t, *J* = 6.3 Hz, 4H), 2.39 (ddd, *J* = 13.3, 7.4, 5.6 Hz, 4H), 1.95 (dq, *J* = 8.3, 6.5 Hz, 4H), **^{19}F NMR (376 MHz, CDCl$_3$):** δ -98.85 (p, *J* = 5.6 Hz), **^{13}C NMR (100 MHz, CDCl$_3$):** δ 162.4 (t, *J* = 35.0 Hz), 152.5 (t, *J* = 261.1 Hz), 135.2 (t, *J* = 5.9 Hz), 43.9, 33.1, 29.8, **HRMS (ESI, m/z):** calculated for [M+H]$^+$: 229.0362, found: 229.0368.

4-(2,2-difluorovinylidene)-1,1-dimethylcyclohexane

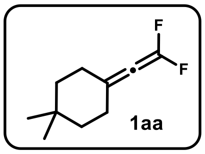

^{1}H NMR (400 MHz, CDCl$_3$): δ 2.35 – 2.29 (m, 4H), 1.51 – 1.47 (m, 4H), 1.00 (s, 6H). **^{19}F NMR (376 MHz, CDCl$_3$):** δ -101.26. **^{13}C NMR (101 MHz, CDCl$_3$):** δ 159.90 (t, *J* = 34.3 Hz), 150.86, 148.30, 145.71 (t, *J* = 260 Hz), 135.74, 135.68, 135.62 (t, *J* = 6.08 Hz), 39.67, 30.70, 30.01, 27.96. **HRMS (ESI, m/z):** calculated for $C_{10}H_{14}F_2$ [M]$^+$:172.1064, found: 172.1084.

(4-(2,2-difluorovinylidene)cyclohexyl)benzene

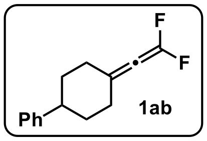

^{1}H NMR (400 MHz, CDCl$_3$): δ 7.44–7.38 (m, 2H), 7.33–7.29 (m, 3H), 2.86 (tt, *J* = 12.2, 3.4 Hz, 1H), 2.66 (d, *J* = 13.8 Hz, 2H), 2.53–2.39 (m, 2H), 2.20–2.15 (m, 2H), 1.81 (qd, *J* = 13.0, 3.8 Hz, 2H); **^{19}F NMR (376 MHz, CDCl$_3$):** δ -100.0

(d, J = 125.2 Hz, 1F), -101.7 (d, J = 125.2 Hz, 1F); **^{13}C NMR (101 MHz, $CDCl_3$):** δ 160.6 (t, J = 34.6 Hz), 148.5 (t, J = 259.0 Hz), 145.7, 134.2 (t, J = 6.1 Hz), 128.5, 126.7, 126.3, 43.4, 34.4; **HRMS (ESI, m/z):** calculated for $C_{14}H_{15}F_2$ $[M+H]^+$: 221.1142, found: 221.1137.

***tert*-butyl 4-(2,2-difluorovinylidene)piperidine-1-carboxylate**

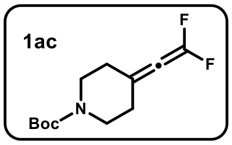

^{1}H NMR (400 MHz, $CDCl_3$): δ 3.53 (t, J = 5.9 Hz, 4H), 2.33 (t, J = 5.6 Hz, 4H), 1.46 (s, 9H). **^{19}F NMR (376 MHz, $CDCl_3$):** δ -100.10 (p, J = 3.7, 3.0 Hz). **^{13}C NMR (101 MHz, $CDCl_3$):** δ 163.00, 162.59, 162.20 (t, J = 34.3 Hz), 154.59, 151.45, 148.86, 146.32 (t, J = 260 Hz), 130.93, 130.87, 130.81 (t, J = 5.87 Hz), 80.21, 69.89, 33.90, 28.52. **HRMS (ESI, m/z):** calculated for $C_{12}H_{17}F_2NO_2$ $[M+H]^+$: 246.1306, found: 246.1313.

4-(2,2-difluorovinylidene)tetrahydro-2*H*-thiopyran

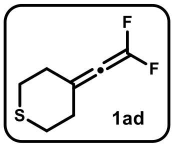

^{1}H NMR (400 MHz, $CDCl_3$): δ 2.89 – 2.74 (m, 4H), 2.72 – 2.57 (m, 4H). **^{19}F NMR (376 MHz, $CDCl_3$):** δ -100.66 (p, J = 3.8 Hz). **^{13}C NMR (101 MHz, $CDCl_3$):** δ 163.59, 163.25, 162.89 (t, J = 35.47 Hz), 151.08, 148.49, 145.90 (t, J = 260.54 Hz), 132.42, 132.36, 132.30 (t, J = 6.05 Hz), 36.10, 36.07, 36.05 (t, J = 2.41 Hz), 30.07, 30.04, 30.01 (t, J = 3.03 Hz). **HRMS (ESI, m/z):** calculated for $C_7H_9F_2S$ $[M+H]^+$: 163.0393, found: 163.0390.

(2,2-difluorovinylidene)cyclododecane

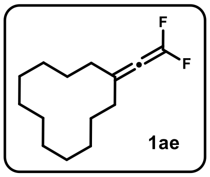

^{1}H NMR (400 MHz, $CDCl_3$): δ 2.27 (p, J = 5.0 Hz, 4H), 1.56 (p, J = 6.5 Hz, 4H), 1.30 (tq, J = 11.3, 5.8, 5.0 Hz, 14H). **^{19}F NMR (376 MHz, $CDCl_3$): -**101.63 (p, J = 5.3, 4.8 Hz). **^{13}C NMR (101 MHz, $CDCl_3$):** δ 163.94 (t, J = 34.3 Hz), 154.10, 151.53, 148.97 (t, J = 260 Hz), 135.80, 135.74, 135.68 (t, J = 5.87 Hz), 33.19, 24.50, 24.42, 23.96, 22.81, 22.03. **HRMS (ESI, m/z):** calculated for $C_{14}H_{22}F_2$ $[M]^+$: 228.1690, found: 228.1701.

(*R*)-6-((2-ethyl-4,4-difluorobuta-2,3-dien-1-yl)oxy)-2,5,7,8-tetramethyl-2-((4*R*,8*R*)-4,8,12-trimethyltridecyl)chromane

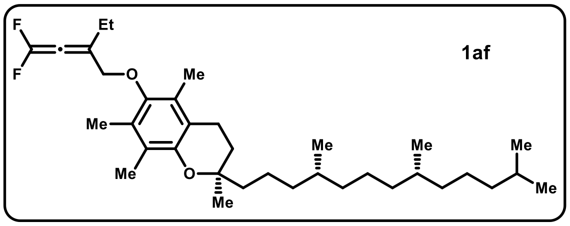

^{1}H NMR (400 MHz, $CDCl_3$): δ 4.37 (t, J = 4.8 Hz, 2H), 2.70 (t, J = 6.5 Hz, 2H), 2.57 – 2.48 (m, 2H), 2.30 (s, 3H), 2.26 (s, 3H), 2.24 (s, 3H), 1.91 (ddt, J = 19.4, 12.6, 6.3 Hz, 2H), 1.70 (dq, J = 20.1, 6.9, 5.8 Hz, 3H), 1.60 – 1.52 (m, 3H), 1.49 – 1.40 (m,

7H), 1.38 (s, 3H), 1.34 – 1.19 (m, 10H), 1.05 – 1.00 (m, 13H). **^{19}F NMR (376 MHz, $CDCl_3$):** δ -99.00. **^{13}C NMR (101 MHz, $CDCl_3$):** δ 164.41, 164.07,163.71 (t, *J* = 35.95 Hz), 155.95, 153.35,150.74 (t, *J* = 260.79 Hz), 148.05, 147.86, 134.85, 134.80, 134.74 (t, *J* = 5.82 Hz), 127.65, 125.70, 122.98, 117.52, 74.71, 73.52, 39.96, 39.90, 39.41, 37.58, 37.50, 37.44, 37.33, 32.82, 32.68, 31.31, 31.26, 28.00, 25.68, 24.87, 24.48, 23.76, 22.71, 22.61, 21.03, 20.64, 19.72, 19.65, 19.60, 19.57, 12.60, 11.72, 11.35. **HRMS (ESI, m/z):** calculated for $C_{35}H_{57}F_2O_2$ $[M+H]^+$: 547.4327, found: 547.4307.

(5,5-dibromo-3-methylpenta-3,4-dien-1-yl)benzene

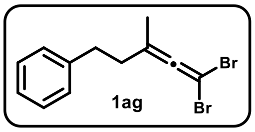

^{1}H NMR (400 MHz, $CDCl_3$): δ 7.30 – 7.24 (m, 2H), 7.20 – 7.14 (m, 3H), 2.78 (t, *J* = 7.8 Hz, 2H), 2.47 (t, *J* = 7.8 Hz, 2H), 1.88 (s, 3H). **^{13}C NMR (100 MHz, $CDCl_3$):** δ 199.7, 140.7, 128.4, 128.2, 126.1, 113.3, 52.5, 35.5, 33.1, 19.1. **HRMS (ESI, m/z):** calculated for $C_{12}H_{13}Br_2$ $[M+H]^+$: 314.9384, found: 314.9370.

6.2 Characterization of the trifluoromethyl alkenes

ethyl-4-(1,1,1-trifluoro-5-phenylpent-2-en-2-yl)benzoate

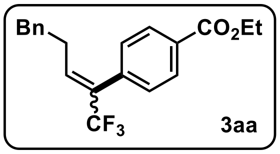

3aa was obtained as a colorless oil (30.0 mg, 0.0860 mmol, yield: 86%, *E/Z* = 16:1), **^{1}H NMR (400 MHz, $CDCl_3$)**: δ 8.01 (d, *J* = 8.2 Hz, 2H), 7.30–7.19 (m, 3H), 7.10 (d, *J* = 8.1 Hz, 2H), 7.06 (d, *J* = 7.1 Hz, 2H), 6.49 (t, *J* = 7.1 Hz, 1H), 4.39 (q, *J* = 7.1 Hz, 2H), 2.71 (t, *J* = 7.5 Hz, 2H), 2.35–2.24 (m, 2H), 1.40 (t, *J* = 7.1 Hz, 3H); **^{19}F NMR (376 MHz, $CDCl_3$):** δ -65.6 (s, 3F); **^{13}C NMR (101 MHz, $CDCl_3$):** δ 166.2, 140.2, 136.7, 136.2 (q, *J* = 5.3 Hz), 131.2 (q, *J* = 29.8 Hz), 130.5, 129.7, 129.5, 128.5, 128.4, 126.3, 123.1 (q, *J* = 273.1 Hz), 61.1, 34.8, 30.2, 14.3; **HRMS (ESI, m/z):** calculated for $C_{20}H_{20}F_3O_2$ $[M+H]^+$: 349.1415, found: 349.1420. Rf = 0.4 (PE/EA=20:1).

ethyl-4-(1,1,1-trifluoro-4-phenylbut-2-en-2-yl)benzoate

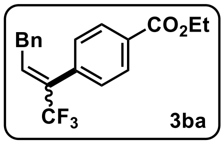

3ba was obtained as a colorless oil (22.4 mg, 0.067mmol, yield: 67%, *E/Z* = 33.6:1), **^{1}H NMR (400 MHz, $CDCl_3$)**: δ 8.11 (d, *J* = 8.5 Hz, 2H), 7.40 (d, *J* = 8.1 Hz, 2H), 7.33–7.29 (m, 2H), 7.26–7.21 (m, 1H), 7.11–7.08 (m, 2H), 6.65 (tq, *J* = 7.7, 1.6 Hz, 1H), 4.41 (q, *J* = 7.1 Hz, 2H), 3.33 (dq, *J* = 7.5, 2.4 Hz, 2H), 1.41 (t, *J* = 7.1 Hz, 3H); **^{19}F NMR (376 MHz, $CDCl_3$):** δ -65.4 (s, 3F); **^{13}C NMR (101 MHz, $CDCl_3$):** δ 166.1, 137.9, 136.5, 135.5 (q, *J* = 5.5 Hz), 131.1 (q, *J* = 30.1 Hz), 130.8, 129.8, 129.7, 128.8, 128.3, 126.7, 123.1 (q, *J* = 273.5 Hz), 61.2, 34.4, 14.3; **HRMS (ESI, m/z):** calculated for $C_{19}H_{18}F_3O_2$ $[M+H]^+$: 335.1259, found: 335.1257. Rf = 0.4 (PE/EA=20:1).

ethyl-4-(7-chloro-1,1,1-trifluorohept-2-en-2-yl)benzoate

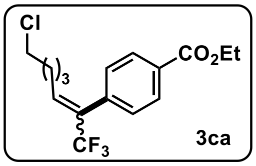

3ca was obtained as a colorless oil (22.4 mg, 0.067 mmol, yield: 67%, *E/Z* = 9.4:1), **^{1}H NMR (400 MHz, $CDCl_3$)**: δ 8.08 (d, *J* = 8.4 Hz, 2H), 7.31 (d, *J* = 8.2 Hz, 2H), 6.45 (tq, *J* = 7.7, 1.7 Hz, 1H), 4.40 (q, *J* = 7.1 Hz, 2H), 3.46 (t, *J* = 6.4 Hz, 2H), 2.07–1.98 (m, 2H), 1.75–1.66 (m, 2H), 1.59–1.52 (m, 2H), 1.40 (t, *J* = 7.1 Hz, 3H); **^{19}F NMR (376 MHz, $CDCl_3$):** δ -65.4 (s, 3F); **^{13}C NMR (101 MHz, $CDCl_3$):** δ 166.1, 136.7, 136.5 (q, *J* = 5.4 Hz), 131.1 (q, *J* = 29.7 Hz), 130.6, 129.7, 129.6, 123.1 (q, *J* = 272.8 Hz), 61.2, 44.4, 31.8, 27.5, 25.8, 14.3; **HRMS (ESI, m/z):** calculated for $C_{16}H_{18}ClF_3NaO_2$ $[M+Na]^+$: 357.0845, found: 357.0843. Rf = 0.4 (PE/EA=20:1).

ethyl-4-(9-bromo-1,1,1-trifluoronon-2-en-2-yl)benzoate

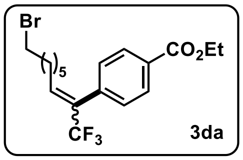

3da was obtained as a colorless oil (19.9 mg, 0.049 mmol, yield: 49%, *E/Z* = 13.9:1), **^{1}H NMR (400 MHz, CDCl$_3$)**: δ 8.07 (d, *J* = 8.1 Hz, 2H), 7.31 (d, *J* = 8.3 Hz, 2H), 6.45 (tq, *J* = 7.9, 1.7 Hz, 1H), 4.39 (q, *J* = 7.1 Hz, 2H), 3.36 (t, *J* = 6.8 Hz, 2H), 2.02–1.95 (m, 2H), 1.84–1.77 (m, 2H), 1.43–1.32 (m, 7H), 1.29–1.21 (m, 2H); **^{19}F NMR (376 MHz, CDCl$_3$):** δ -65.3 (s, 3F); **^{13}C NMR (101 MHz, CDCl$_3$):** δ 166.1, 137.2 (q, *J* = 5.2 Hz), 136.9, 130.5 (q, *J* = 29.6 Hz), 130.5, 129.8, 129.6, 123.2 (q, *J* = 272.6 Hz), 61.1, 33.8, 32.5, 28.4, 28.2, 28.2, 27.8, 14.3; **HRMS (ESI, m/z):** calculated for $C_{18}H_{22}BrF_3NaO_2$ [M+Na]$^+$: 429.0653, found: 429.0656. Rf = 0.4 (PE/EA=20:1).

ethyl-4-(1,1,1-trifluoro-8-methoxy-8-oxooct-2-en-2-yl)benzoate

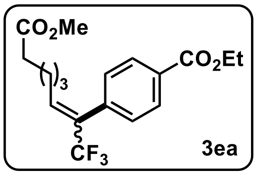

3ea was obtained as an orange oil (21.1 mg, 0.059 mmol, yield: 59%, *E/Z* = 10.3:1), **^{1}H NMR (400 MHz, CDCl$_3$)**: δ 8.07 (d, *J* = 8.6 Hz, 2H), 7.30 (d, *J* = 8.2 Hz, 2H), 6.45 (tq, *J* = 7.6, 1.6 Hz, 1H), 4.39 (q, *J* = 7.1 Hz, 2H), 3.65 (s, 3H), 2.24 (t, *J* = 7.3 Hz, 2H), 2.04–1.97 (m, 2H), 1.60–1.52 (m, 2H), 1.47–1.38 (m, 5H); **^{19}F NMR (376 MHz, CDCl$_3$):** δ -65.4 (s, 3F); **^{13}C NMR (101 MHz, CDCl$_3$):** δ 173.7, 166.1, 136.8, 136.8 (q, *J* = 4.9 Hz), 130.8 (q, *J* = 29.8 Hz), 130.5, 129.7, 129.6, 123.1 (q, *J* = 272.8 Hz), 61.1, 51.6, 33.6, 28.1, 28.0, 24.3, 14.3; **HRMS (ESI, m/z):** calculated for $C_{18}H_{21}F_3NaO_4$ [M+Na]$^+$: 381.1290, found: 381.1288. Rf = 0.6 (PE/EA=10:1).

methyl-2-(4-(4-(ethoxycarbonyl)phenyl)-5,5,5-trifluoropent-3-en-1-yl)benzoate

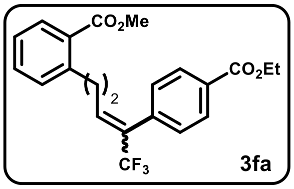

3fa was obtained as a colorless oil (22.07 mg, 0.0543 mmol, yield: 54.3%, *Z/E* = 26:1), **^{1}H NMR (400 MHz, CDCl$_3$):** δ 8.02 – 7.95 (m, 2H), 7.89 (dd, *J* = 7.8, 1.5 Hz, 1H), 7.41 (td, *J* = 7.5, 1.5 Hz, 1H), 7.28 (d, *J* = 1.3 Hz, 1H), 7.09 (dd, *J* = 7.6, 5.3 Hz, 3H), 6.53 (tq, *J* = 7.8, 1.7 Hz, 1H), 4.38 (q, *J* = 7.1 Hz, 2H), 3.81 (s, 3H), 3.10 (t, *J* = 7.5 Hz, 2H), 2.30 (qd, *J* = 7.6, 2.1 Hz, 2H), 1.39 (t, *J* = 7.1 Hz, 3H). **^{19}F NMR (376 MHz, CDCl$_3$):** δ -65.51 (s, 3F). **^{13}C NMR (101 MHz, CDCl$_3$):** δ 167.66, 166.31, 142.52, 136.83, 136.63, 136.58, 136.53, 136.47 (q, *J* = 5.25 Hz), 132.29, 131.33, 131.05, 130.87, 130.47, 129.85, 129.55, 129.29, 127.21, 126.54, 124.58, 12186, 119.15, (q, *J* = 273.44 Hz), 61.17, 52.01, 33.20, 30.25, 14.42. **HRMS (ESI, m/z)**: calculated for $C_{22}H_{21}F_3Na\ O_4$ [M+Na]$^+$: 429.1290, found: 429.1289. Rf = 0.6 (PE/EA=10:1).

ethyl-4-(1,1,1-trifluoro-5-(5-methylfuran-2-yl)pent-2-en-2-yl)benzoate

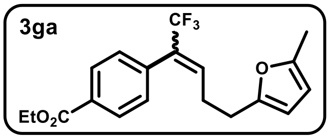

3ga was obtained as a colorless oil (19.7 mg, 0.056 mmol, yield: 56%, *E/Z* = 16:1), **^{1}H NMR (400 MHz, CDCl$_3$):** δ 8.06 – 8.03 (m, 2H), 7.21 (d, *J* = 8.1 Hz, 2H), 6.48 (td, *J* = 7.6, 1.7 Hz, 1H), 5.84 (dd, *J* = 3.0, 1.1 Hz, 1H), 5.82 (d, *J* = 3.1 Hz, 1H), 4.39 (q, *J* = 7.1 Hz, 2H), 2.66 (t, *J* = 7.2 Hz, 2H), 2.31 (td, *J* = 7.3, 2.2 Hz, 2H), 2.22 (s, 3H), 1.40 (t, *J* = 7.1 Hz, 3H). **^{19}F NMR (376 MHz, CDCl$_3$):** δ -65.51 (s, 3F). **^{13}C NMR (101 MHz, CDCl$_3$):** δ 166.28, 152.08, 150.89, 136.84, 136.30, 136.24, 136.19, 136.14 (q, J =5.36 Hz), 131.48, 131.19, 130.64, 129.84, 129.68, 127.30, 124.57, 121.85, 119.13 (q, J =273.6 Hz), 106.49, 106.02, 61.25, 27.34, 14.43, 13.59. **HRMS (ESI, m/z)**: calculated for $C_{19}H_{20}F_3O_3$ [M+H]$^+$: 353.1364, found: 353.1365. Rf = 0.4 (PE/EA=20:1).

ethyl-4-(1,1,1-trifluoro-9-methoxy-5,9-dimethyldec-2-en-2-yl)benzoate

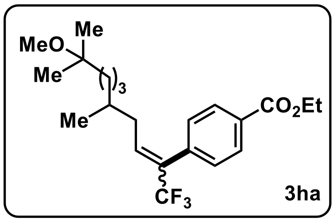

3ha was obtained as an orange oil (26.4 mg, 0.066 mmol, yield: 66%, *E/Z* = 10.9:1), **^{1}H NMR (400 MHz, CDCl$_3$)**: δ 8.07 (d, *J* = 8.3 Hz, 2H), 7.30 (d, *J* = 8.2 Hz, 2H), 6.48 (tq, *J* = 7.6, 1.5 Hz, 1H), 4.41 (q, *J* = 7.1 Hz, 2H), 3.15 (s, 3H), 2.04–1.95 (m, 1H), 1.86–1.77 (m, 1H), 1.61–1.53 (m, 1H), 1.45–1.34 (m, 6H), 1.29–1.16 (m, 3H), 1.10 (s, 6H), 0.84 (d, *J* = 6.7 Hz, 3H); **^{19}F NMR (376 MHz, CDCl$_3$):** δ -65.3 (s, 3F); **^{13}C NMR (101 MHz, CDCl$_3$):** δ 166.2, 137.1, 136.5 (q, *J* = 5.1 Hz), 131.0 (q, *J* = 29.4 Hz), 130.1, 129.9, 129.6, 123.2 (q, *J* = 272.9 Hz), 74.4, 61.1, 49.1, 40.0, 37.0, 35.4, 32.9, 24.9, 21.2, 19.5, 14.3; **HRMS (ESI, m/z):** calculated for $C_{22}H_{32}F_3O_3$ [M+H]$^+$: 401.2304, found: 401.2305. Rf = 0.4 (PE/EA=20:1).

ethyl-4-(1,1,1-trifluoro-4-phenylpent-2-en-2-yl)benzoate

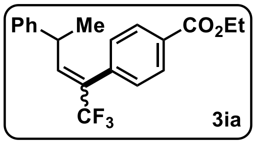

3ia was obtained as an orange oil (23.7 mg, 0.068 mmol, yield: 68%, *E/Z* = 36.3:1), **^{1}H NMR (400 MHz, CDCl$_3$)**: δ 8.11 (d, *J* = 8.5 Hz, 2H), 7.36–7.29 (m, 4H), 7.26–7.20 (m, 1H), 7.13–7.08 (m, 2H), 6.57 (dq, *J* = 10.7, 1.5 Hz, 1H), 4.41 (q, *J* = 7.1 Hz, 2H), 3.44–3.36 (m, 1H), 1.42 (t, *J* = 7.1 Hz, 3H), 1.35 (d, *J* = 6.9 Hz, 3H); **^{19}F NMR (376 MHz, CDCl$_3$):** δ -65.6 (s, 3F); **^{13}C NMR (101 MHz, CDCl$_3$):** δ 166.1, 143.4, 141.0 (q, *J* = 5.1 Hz), 136.7, 130.7, 129.8, 129.7, 128.9 (q, *J* = 30.0 Hz), 128.8, 126.8, 126.7, 123.1 (q, *J* = 273.0 Hz), 61.2, 38.3, 21.4, 14.3; **HRMS (ESI, m/z):** calculated for $C_{20}H_{19}F_3NaO_2$ [M+Na]$^+$: 371.1235, found: 371.1231. Rf = 0.4 (PE/EA=20:1).

ethyl -4-(5-(4-(tert-butyl)phenyl)-1,1,1-trifluoro-4-methylpent-2-en-2-yl)benzoate

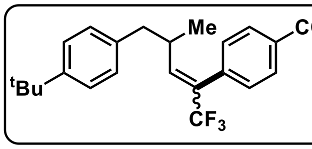

3ja

3ja was obtained as a colorless oil (31.4 mg, 0.075 mmol, yield: 75%, *E/Z* = 16.0:1), **^{1}H NMR (400 MHz, CDCl$_3$)**: δ 7.91 (d, *J* = 8.0 Hz, 2H), 7.28 (d, *J* = 8.1 Hz, 2H), 6.89 (d, *J* = 8.1 Hz, 2H), 6.78 (d, *J* = 8.1 Hz, 2H), 6.25 (d, *J* = 10.8 Hz, 1H), 4.39 (q, *J* = 7.1 Hz, 2H), 2.64–2.47 (m, 2H), 2.26–2.24 (m, 1H), 1.40 (t, *J* = 7.2 Hz, 3H), 1.34 (s, 9H), 1.03 (d, *J* = 6.6 Hz, 3H); **^{19}F NMR (376 MHz, CDCl$_3$):** δ -65.8 (s, 3F); **^{13}C NMR (101 MHz, CDCl$_3$):** δ 166.2, 149.2, 141.7 (q, *J* = 5.3 Hz), 136.8, 136.2, 130.3, 129.7, 129.6 (q, *J* = 29.9 Hz), 129.2, 128.8, 125.1, 123.1 (q, *J* = 272.6 Hz), 61.1, 42.4, 35.7, 34.4, 31.4, 20.1, 14.3; **HRMS (ESI, m/z):** calculated for $C_{25}H_{30}F_3O_2$ $[M+H]^+$: 419.2198, found: 419.2204. Rf = 0.5(PE/EA=20:1).

ethyl-4-(5-(benzo[*d*][1,3]dioxol-5-yl)-1,1,1-trifluoro-4-methylpent-2-en-2-yl)benzoate

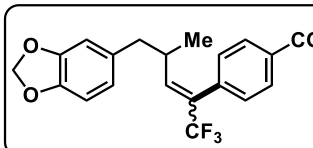

3ka

3ka was obtained as a colorless oil (27.2 mg, 0.067 mmol, yield: 67%, *E/Z* = 15:1), **^{1}H NMR (400 MHz, CDCl$_3$)**: δ 7.98 (d, *J* = 8.2 Hz, 2H), 6.97 (d, *J* = 8.2 Hz, 2H), 6.70 (d, *J* = 7.8 Hz, 1H), 6.45–6.39 (m, 2H), 6.23 (dq, *J* = 10.8, 1.5 Hz, 1H), 5.96–5.92 (m, 2H), 4.39 (q, *J* = 7.1 Hz, 2H), 2.56–2.44 (m, 2H), 2.40–2.26 (m, 1H), 1.40 (t, *J* = 7.2 Hz, 3H), 1.00 (d, *J* = 6.6 Hz, 3H); **^{19}F NMR (376 MHz, CDCl$_3$):** δ -65.7 (s, 3F); **^{13}C NMR (101 MHz, CDCl$_3$):** δ 166.2, 147.4, 145.9, 141.6 (q, *J* = 5.1 Hz), 136.8, 132.9, 130.4, 129.7 (q, *J* = 29.9 Hz), 129.6, 129.4, 123.0 (q, *J* = 273.0 Hz), 122.0, 109.4, 108.0, 100.8, 61.1, 42.6, 35.4, 19.9, 14.3; **HRMS (ESI, m/z):** calculated for $C_{22}H_{22}F_3O_4$ $[M+H]^+$: 407.1470, found: 407.1472. Rf = 0.4 (PE/EA=20:1).

ethyl-4-(1,1,1-trifluoro-4,8-dimethylnona-2,7-dien-2-yl)benzoate

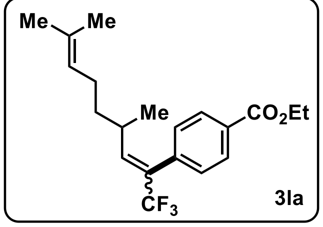

3la

3la was obtained as a colorless oil (14.9 mg, 0.042mmol, yield: 42%, *E/Z* = 18:1), **^{1}H NMR (400 MHz, CDCl$_3$)**: δ 8.06 (d, *J* = 8.5 Hz, 2H), 7.30 (d, *J* = 8.2 Hz, 2H), 6.23 (dq, *J* = 10.8, 1.5 Hz, 1H), 4.95–4.90 (m, 1H), 4.39 (q, *J* = 7.1 Hz, 2H), 2.20–2.07 (m, 1H), 1.93–1.76 (m, 2H), 1.62 (s, 3H), 1.54 (s, 3H), 1.40 (t, *J* = 7.1 Hz, 3H), 1.36–1.28 (m, 2H), 0.97 (d, *J* = 6.7 Hz, 3H); **^{19}F NMR (376 MHz, CDCl$_3$):** δ -65.4 (s, 3F); **^{13}C NMR (101 MHz, CDCl$_3$):** δ 166.2, 142.8 (q, *J* = 5.0 Hz), 137.2, 132.0, 130.4, 129.8, 129.5, 129.3 (q, *J* = 29.7 Hz), 123.7, 123.2 (q, *J* = 273.0 Hz), 61.1, 36.6, 32.6, 25.7, 25.6, 20.2, 17.6, 14.3; **HRMS (ESI, m/z):** calculated for $C_{20}H_{26}F_3O_2$ $[M+H]^+$: 355.1885, found: 355.1877. Rf = 0.4 (PE/EA=20:1).

ethyl-4-(3,3,3-trifluoro-1-(4-phenylcyclohexyl)prop-1-en-2-yl)benzoate

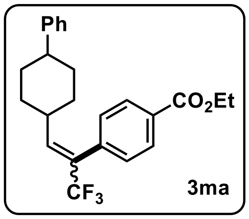

3ma was obtained as a colorless oil (28.1 mg, 0.070 mmol, yield: 70%, *E/Z* > 99:1), **^{1}H NMR (400 MHz, CDCl$_3$)**: δ 8.10 (d, *J* = 8.5 Hz, 2H), 7.35 (d, *J* = 8.2 Hz, 2H), 7.28–7.24 (m, 2H), 7.20–7.11 (m, 3H), 6.30 (dq, *J* = 10.4, 1.5 Hz, 1H), 4.41 (q, *J* = 7.1 Hz, 2H), 2.49–2.43 (m, 1H), 2.08–1.95 (m, 1H), 1.90–1.82 (m, 2H), 1.79–1.74 (m, 2H), 1.42 (t, *J* = 7.1 Hz, 3H), 1.39–1.26 (m, 4H); **^{19}F NMR (376 MHz, CDCl$_3$):** δ -65.5 (s, 3F); **^{13}C NMR (101 MHz, CDCl$_3$):** δ 166.2, 146.9, 142.0 (q, *J* = 5.2 Hz), 137.2, 130.6, 129.7, 129.6, 129.2 (q, *J* = 29.8 Hz), 128.3, 126.7, 126.0, 123.2 (q, *J* = 272.6 Hz), 61.2, 43.5, 37.0, 32.9, 32.3, 14.3; **HRMS (ESI, m/z):** calculated for $C_{24}H_{26}F_3O_2$ [M+H]$^+$: 403.1885, found: 403.1882. Rf = 0.4 (PE/EA=20:1).

ethyl-4-(1-(cyclohex-3-en-1-yl)-3,3,3-trifluoroprop-1-en-2-yl)benzoate

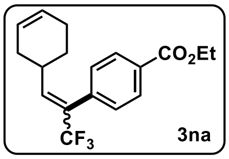

3na was obtained as an orange oil (16.2 mg, 0.050 mmol, yield: 50%, *E/Z* = 25.8:1), **^{1}H NMR (400 MHz, CDCl$_3$)**: δ 8.07 (d, *J* = 8.5 Hz, 2H), 7.32 (d, *J* = 8.2 Hz, 2H), 6.35 (dq, *J* = 10.4, 1.6 Hz, 1H), 5.68–5.55 (m, 2H), 4.39 (q, *J* = 7.1 Hz, 2H), 2.30–2.18 (m, 1H), 2.05–1.84 (m, 4H), 1.70–1.61 (m, 1H), 1.53–1.43 (m, 1H), 1.40 (t, *J* = 7.1 Hz, 3H); **^{19}F NMR (376 MHz, CDCl$_3$):** δ -65.5 (s, 3F); **^{13}C NMR (101 MHz, CDCl$_3$):** δ 166.1, 141.5 (q, *J* = 5.0 Hz), 137.0, 130.6, 129.7, 129.6, 129.6 (q, *J* = 29.6 Hz), 126.9, 124.8, 123.2 (q, *J* = 272.9 Hz), 61.1, 33.0, 30.4, 27.8, 23.7, 14.3; **HRMS (ESI, m/z):** calculated for $C_{18}H_{19}F_3NaO_2$ [M+Na]$^+$: 347.1235, found: 347.1232. Rf = 0.4 (PE/EA=20:1).

ethyl-4-(1,1,1-trifluoro-4-(4-methoxyphenyl)-4-methylpent-2-en-2-yl)benzoate

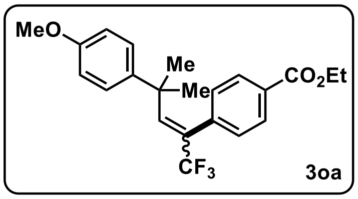

3oa was obtained as an orange oil (19.6mg, 0.050 mmol, yield: 50%, *E/Z* = 87:1), **^{1}H NMR (400 MHz, CDCl$_3$)**: δ 7.86 (d, *J* = 8.4 Hz, 2H), 7.05–6.99 (m, 4H), 6.74–6.70 (m, 2H), 6.69 (q, *J* = 1.6 Hz, 1H), 4.37 (q, *J* = 7.1 Hz, 2H), 3.77 (s, 3H), 1.39 (t, *J* = 7.1 Hz, 3H), 1.28 (s, 6H); **^{19}F NMR (376 MHz, CDCl$_3$):** δ -66.1 (s, 3F); **^{13}C NMR (101 MHz, CDCl$_3$):** δ 166.2, 157.8, 145.3 (q, *J* = 5.0 Hz), 139.9, 136.9, 130.4, 130.0, 128.9 (q, *J* = 29.4 Hz), 128.7, 126.9, 123.3 (q, *J* = 273.7 Hz), 113.5, 61.0, 55.2, 39.8, 30.1, 14.3; **HRMS (ESI, m/z):** calculated for $C_{22}H_{23}F_3NaO_3$ [M+Na]$^+$: 415.1497, found: 415.1499. Rf = 0.4 (PE/EA=20:1).

ethyl-4-(3,3,3-trifluoro-1-(1-(4-(trifluoromethyl)phenyl)cyclopropyl)prop-1-en-2-yl)benzoate

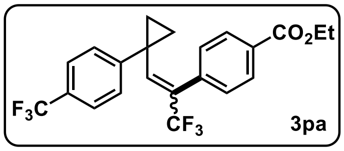

3pa was obtained as a colorless oil (23.9 mg, 0.056 mmol, yield: 56%, *E/Z* > 99:1), **^{1}H NMR (400 MHz, CDCl$_3$)**: δ 7.81 (d, *J* = 8.3 Hz, 2H), 7.32 (d, *J* = 8.1 Hz, 2H), 7.09 (d, *J* = 8.1 Hz, 2H), 7.06 (d, *J* = 8.2 Hz, 2H), 6.54 (q, *J* = 1.7 Hz, 1H), 4.36 (q, *J* = 7.1 Hz, 2H), 1.38 (t, *J* = 7.1 Hz, 3H), 1.15–1.02 (m, 4H); **^{19}F NMR (376 MHz, CDCl$_3$):** δ -62.5 (s, 3F), -65.2 (s, 3F); **^{13}C NMR (101 MHz, CDCl$_3$):** δ 165.9, 146.6, 139.8 (q, *J* = 5.5 Hz), 136.2, 131.3 (q, *J* = 29.5 Hz), 130.3, 129.9, 129.0, 128.4 (q, *J* = 32.4 Hz), 128.3, 125.0 (q, *J* = 3.7 Hz), 123.9 (q, *J* = 272.1 Hz), 123.9 (q, *J* = 273.5 Hz), 61.1, 24.8, 18.0, 14.2; **HRMS (ESI, m/z):** calculated for $C_{22}H_{18}F_6NaO_2$ [M+Na]$^+$: 451.1109, found: 451.1106. Rf = 0.4 (PE/EA=20:1).

ethyl 4-(-1-(1-cinnamylcyclohexyl)-3,3,3-trifluoroprop-1-en-2-yl)benzoate

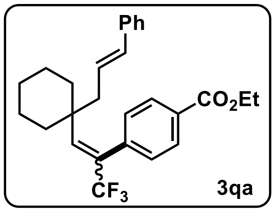

3qa was obtained as a colorless oil (19.0 mg, 0.043 mmol, yield: 43%, *E/Z* > 99:1), **^{1}H NMR (400 MHz, CDCl$_3$)**: δ 8.02 (d, *J* = 8.4 Hz, 2H), 7.38–7.31 (m, 7H), 6.37 (d, J = 15.7 Hz, 1H), 6.33 (q, *J* = 1.5 Hz, 1H), 6.14 (dt, *J* = 15.7, 7.8 Hz, 1H), 4.39 (q, *J* = 7.1 Hz, 2H), 2.23 (d, *J* = 7.4 Hz, 2H), 1.44–1.25 (m, 13H); **^{19}F NMR (376 MHz, CDCl$_3$):** δ -65.8 (s, 3F); **^{13}C NMR (101 MHz, CDCl$_3$):** δ 166.2, 143.6 (q, *J* = 5.1 Hz), 137.6, 137.3, 133.1, 130.6, 130.2, 129.7 (q, *J* = 28.9 Hz), 129.1, 128.6, 127.2, 126.0, 125.5, 123.4 (q, *J* = 273.9 Hz), 61.2, 44.6, 41.1, 36.4, 25.7, 22.5, 14.3; **HRMS (ESI, m/z):** calculated for $C_{27}H_{30}F_3O_2$ [M+H]$^+$: 443.2198, found: 443.2200. Rf = 0.45 (PE/EA=20:1).

ethyl 4-(-1-(adamantan-1-yl)-3,3,3-trifluoroprop-1-en-2-yl)benzoate

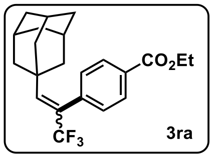

3ra was obtained as an orange oil (17.4 mg, 0.046 mmol, yield: 46%, *E/Z* > 99:1), **^{1}H NMR (400 MHz, CDCl$_3$)**: δ 8.02 (d, *J* = 8.3 Hz, 2H), 7.32 (d, *J* = 8.2 Hz, 2H), 6.13 (q, *J* = 1.4 Hz, 1H), 4.40 (q, *J* = 7.1 Hz, 2H), 1.86–1.81 (m, 3H), 1.61–1.57 (m, 2H), 1.53–1.45 (m, 10H), 1.41 (t, *J* = 7.1 Hz, 3H); **^{19}F NMR (376 MHz, CDCl$_3$):** δ -66.3 (s, 3F); **^{13}C NMR (101 MHz, CDCl$_3$):** δ 166.3, 145.2 (q, *J* = 4.9 Hz), 137.9, 130.9, 130.4, 128.8, 127.2 (q, *J* = 28.8 Hz), 123.5 (q, *J* = 273.3 Hz), 61.1, 42.2, 36.2, 36.1, 28.0, 14.3; **HRMS (ESI, m/z):** calculated for $C_{22}H_{26}F_3O_2$ [M+H]$^+$: 379.1885, found: 379.1890. Rf = 0.4 (PE/EA=20:1).

1-(4-(1,1,1-trifluoro-5-phenylpent-2-en-2-yl)phenyl)ethan-1-one

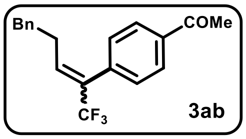

3ab was obtained as a colorless oil (24.5 mg, 0.077 mmol, yield: 77%, *E/Z* = 33:1), **^{1}H NMR (400 MHz, $CDCl_3$):** δ 7.92 – 7.88 (m, 2H), 7.29 – 7.26 (m, 1H), 7.25 – 7.19 (m, 2H), 7.11 (d, *J* = 8.2 Hz, 2H), 7.05 (d, *J* = 7.0 Hz, 2H), 6.49 (tq, *J* = 7.8, 1.8 Hz, 1H), 2.70 (t, *J* = 7.5 Hz, 2H), 2.60 (s, 3H), 2.29 (qd, *J* = 7.6, 2.1 Hz, 2H). **^{19}F NMR (376 MHz, $CDCl_3$):** δ -65.49 (s, 3F). **^{13}C NMR (101 MHz, $CDCl_3$):** δ 197.78, 140.32, 137.09, 136.97, 136.51, 136.45, 136.35 (q, *J* = 5.46 Hz), 136.40, 136.35, 135.99, 131.60, 131.29, 131.00, 130.07 (q, *J* = 29.79 Hz), 128.60, 128.56, 128.39, 127.24, 126.43, 124.53, 121.80, 119.12 (q, *J* = 273.45 Hz), 34.86, 30.37, 26.79. **HRMS (ESI, m/z)**: calculated for $C_{19}H_{18}F_3O$ $[M+H]^+$: 319.1310, found: 319.1311. Rf = 0.25 (PE/EA=20:1).

1-(3-(1,1,1-trifluoro-5-phenylpent-2-en-2-yl)phenyl)ethan-1-one

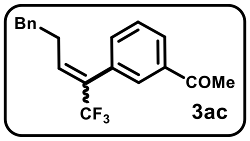

3ac was obtained as a colorless oil (13.7 mg, 0.043 mmol, yield: 43.3%, *E/Z* = 96:1), **^{1}H NMR (400 MHz, $CDCl_3$):** ^{1}H NMR (400 MHz,) δ 7.94 (dt, *J* = 7.8, 1.2 Hz, 1H), 7.68 (s, 1H), 7.44 (t, *J* = 7.7 Hz, 1H), 7.28 (t, *J* = 1.4 Hz, 1H), 7.24 (s, 1H), 7.23 – 7.18 (m, 2H), 7.06 (d, *J* = 7.0 Hz, 2H), 6.55 – 6.48 (m, 1H), 2.71 (t, *J* = 7.5 Hz, 2H), 2.59 (s, 3H), 2.29 (qd, *J* = 7.6, 2.1 Hz, 2H). **^{19}F NMR (376 MHz, $CDCl_3$):** δ -65.75 (s, 3F). **^{13}C NMR (101 MHz, $CDCl_3$):** δ 197.75, 140.37, 137.30, 136.53, 136.47, 136.42, 136.37 (q, *J* = 5.41 Hz), 134.40, 132.72, 131.26, 130.96, 130.66 (q, *J* = 29.70 Hz), 129.57, 128.85, 128.57, 128.54, 128.45, 127.33, 126.39, 124.62, 121.91, 118.09 (q, *J* = 273.94 Hz), 34.86, 30.32, 26.80. **HRMS (ESI, m/z)**: calculated for $C_{19}H_{18}F_3O$ $[M+H]^+$: 319.1310, found: 319.1309. Rf = 0.25 (PE/EA=20:1).

Phenyl(4-(1,1,1-trifluoro-5-phenylpent-2-en-2-yl)phenyl)methanone

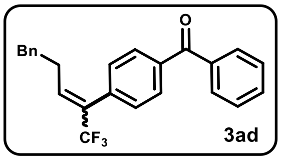

3ad was obtained as an orange oil (20.5 mg, 0.054 mmol, yield: 54%, *E/Z* = 87:1), **^{1}H NMR (400 MHz, $CDCl_3$):** δ 7.80 (dd, *J* = 21.2, 8.2 Hz, 4H), 7.62 (t, *J* = 7.4 Hz, 1H), 7.50 (t, *J* = 7.6 Hz, 2H), 7.29 (d, *J* = 6.9 Hz, 1H), 7.21 (t, *J* = 7.3 Hz, 1H), 7.11 (dd, *J* = 26.7, 7.4 Hz, 4H), 6.52 (tq, *J* = 7.7, 1.7 Hz, 1H), 2.73 (t, *J* = 7.4 Hz, 2H), 2.37 – 2.30 (m, 2H). **^{19}F NMR (376 MHz, $CDCl_3$):** δ -65.45 (s, 3F). **^{13}C NMR (101 MHz, $CDCl_3$):** δ 196.37, 140.36, 137.56, 137.35, 136.55, 136.50, 136.43(q, J = 5.25 Hz), 136.39, 132.77, 131.35, 131.05, 130.76(t, J = 30.12 Hz), 130.20, 130.12, 129.78, 128.62, 128.60, 128.48, 127.29, 126.44, 124.60, 121.88, 119.16 (q, J = 273.94 Hz), 34.89, 30.38. **HRMS (ESI, m/z)**: calculated for $C_{24}H_{20}F_3O$ $[M+H]^+$: 381.1466, found: 381.1464. Rf = 0.4 (PE/EA=20:1).

1-nitro-4-(1,1,1-trifluoro-5-phenylpent-2-en-2-yl)benzene

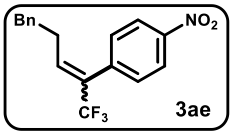

3ae was obtained as an orange oil (20.6 mg, 0.064 mmol, yield: 64%, *E/Z* = 10:1), **^{1}H NMR (400 MHz, $CDCl_3$):** δ 8.20 – 8.14 (m, 2H), 7.32 – 7.21 (m, 3H), 7.13 – 7.09 (m, 2H), 7.07 – 7.03 (m, 2H), 6.56 (tq, *J* = 7.8, 1.7 Hz, 1H), 2.74 (t, *J* = 7.3 Hz, 2H), 2.29 (dtt, *J* = 9.5, 7.4, 2.1 Hz, 2H).**^{19}F NMR (376 MHz, $CDCl_3$):** δ -65.46 (s, 3F). **^{13}C NMR (101 MHz, $CDCl_3$)** δ 147.94, 140.05, 138.87, 137.33, 137.28, 137.22, 137.17 (q, *J* = 5.51Hz), 130.92, 130.83, 130.53, 130.22, 129.92 (q, *J* = 30.17 Hz), 129.12, 128.69, 128.64, 128.50, 128.41, 126.97, 126.58, 124.26, 123.68, 123.61, 121.54, 118.82 (q, *J* = 273.39 Hz), 34.70, 30.49. **HRMS (ESI, m/z)**: calculated for $C_{17}H_{15}F_3O_2$ $[M+H]^+$:322.1055, found: 322.1059. Rf = 0.3 (PE/EA=20:1).

1-(1,1,1-trifluoro-5-phenylpent-2-en-2-yl)-4-(trifluoromethyl) benzene

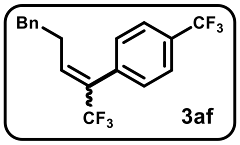

3af was obtained as a colorless oil (30.6 mg, 0.089 mmol, yield: 89%, *E/Z* = 25:1), **^{1}H NMR (400 MHz, $CDCl_3$):** δ 7.59 (d, *J* = 8.0 Hz, 2H), 7.31 – 7.26 (m, 2H), 7.25 – 7.19 (m, 1H), 7.14 – 7.04 (m, 4H), 6.52 (tq, *J* = 7.7, 1.7 Hz, 1H), 2.72 (t, *J* = 7.4 Hz, 2H), 2.34 – 2.25 (m, 2H). **^{19}F NMR (376 MHz, $CDCl_3$):** δ -62.64 (s, 3F), -65.68 (s, 3F). **^{13}C NMR (101 MHz, $CDCl_3$):** δ 139.21, 135.65, 135.59, 135.54, 135.49 (q, *J* = 5.46 Hz), 134.82, 129.97, 129.79, 129.67, 129.47, 129.16, 127.57, 127.54, 127.02, 126.16, 124.32, 123.43, 121.59, 120.72, 118.91, 117.99(q, *J* = 273.25 Hz) (q, *J* = 272.31 Hz), 125.40, 124.41, 124.37, 124.33, 124.30 (q, *J* = 3.75 Hz), 33.75, 29.25. **HRMS (ESI, m/z)**: calculated for $C_{18}H_{14}F_6$ $[M]^+$: 344.1000, found: 344.0991. Rf = 0.6 (PE).

1-(methylsulfonyl)-4-(1,1,1-trifluoro-5-phenylpent-2-en-2-yl)benzene

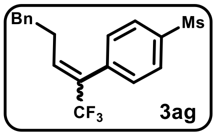

3ag was obtained as a colorless oil (17.85 mg, 0.0451 mmol, yield: 45.1%, *E/Z* > 99:1), **^{1}H NMR (400 MHz, $CDCl_3$):** δ 7.91 – 7.87 (m, 2H), 7.29 (ddd, *J* = 7.6, 6.3, 1.7 Hz, 2H), 7.25 – 7.21 (m, 1H), 7.14 (d, *J* = 8.2 Hz, 2H), 7.05 (d, *J* = 6.7 Hz, 2H), 6.54 (tq, *J* = 7.6, 1.7 Hz, 1H), 3.08 (s, 3H), 2.73 (t, *J* = 7.3 Hz, 2H), 2.28 (dtd, *J* = 9.6, 7.3, 3.1 Hz, 2H). **^{19}F NMR (376 MHz, $CDCl_3$):** δ -65.45 (s, 3F), **^{13}C NMR (101 MHz, $CDCl_3$)** δ 140.59, 140.10, 137.95, 137.26, 137.21, 137.15, 137.10 (q, *J* = 5.31Hz), 130.88, 130.93, 130.63, 130.33 (q, *J* =30.2Hz), 128.68, 128.65, 127.06, 126.55, 124.34, 121.63, 118.91 (q, *J* = 272.94Hz), 44.54, 34.72, 30.41. **HRMS (ESI, m/z)**: calculated for $C_{18}H_{18}F_3O_2S$ $[M+H]^+$:355.0980, found: 355.0970. Rf = 0. (PE/EA=10:1).

dimethyl (E)-5-(1,1,1-trifluoro-5-phenylpent-2-en-2-yl)isophthalate

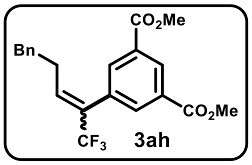

3ah was obtained as a colorless oil (31 mg, 0.082 mmol, yield: 82%, *E/Z* = 46:1).**^{1}H NMR (400 MHz, $CDCl_3$):** δ 8.68 (t, *J* = 1.6 Hz, 1H), 7.95 (d, *J* = 1.6 Hz, 2H), 7.26 – 7.16 (m, 3H), 7.06 – 7.02 (m, 2H), 6.55 (tq, *J* = 7.6, 1.6 Hz, 1H), 3.96 (s, 6H), 2.71 (t, *J* = 7.5 Hz, 2H), 2.28 (qd, *J* = 7.6, 2.1 Hz, 2H). **^{19}F NMR (376 MHz, $CDCl_3$):** δ -65.64 (s, 3F). **^{13}C NMR (101 MHz, $CDCl_3$)** δ 165.83, 140.15, 137.26, 137.21, 137.16, 137.11 (q, *J* = 5.22 Hz), 135.03, 133.94, 133.04, 131.08, 130.88, 128.60, 128.47, 127.14, 126.43, 124.44, 121.72, 119.00 (q, *J* = 273.06 Hz), 52.66, 34.76, 30.33. **HRMS (ESI, m/z)**: calculated for $C_{21}H_{20}F_3O_4$ $[M+H]^+$:393.1314, found: 393.1308. Rf = 0.5 (PE/EA=10:1).

(5,5,5-trifluoropent-3-ene-1,4-diyl)dibenzene

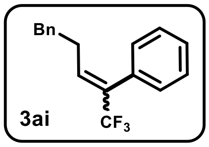

3ai was obtained as a colorless oil (18.8 mg, 0.068 mmol, yield: 68%, *E/Z* = 50:1), **^{1}H NMR (400 MHz, $CDCl_3$):** δ 7.37 – 7.33 (m, 3H), 7.30 – 7.26 (m, 1H), 7.25 (s, 1H), 7.23 – 7.18 (m, 1H), 7.11 – 7.05 (m, 4H), 6.45 (td, J = 7.6, 1.7 Hz, 1H), 2.70 (t, J = 7.6 Hz, 2H), 2.31 (qd, J = 7.6, 2.1 Hz, 2H). **^{19}F NMR (376 MHz, $CDCl_3$):** δ -65.78 (s, 3F). **^{13}C NMR (101 MHz, $CDCl_3$):** δ 140.67, 135.64, 135.59, 135.54, 135.48(q, J = 5.55 Hz), 132.24, 132.06, 131.76, 129.71, 128.54, 128.49, 128.44, 127.58, 126.30, 124.87, 119.42 (q, J = 274.66 Hz), 122.15, 30.25. **HRMS (ESI, m/z)**: calculated for $C_{17}H_{16}F_3$ $[M+H]^+$: 277.1204, found: 277.1216. Rf = 0.6 (PE).

2-(1,1,1-trifluoro-5-phenylpent-2-en-2-yl)naphthalene

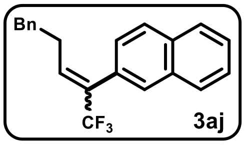

3aj was obtained as a colorless oil (18.6 mg, 0.057 mmol, yield: 57%, *E/Z* = 52:1), **^{1}H NMR (400 MHz, $CDCl_3$):** δ 7.82 (td, *J* = 13.8, 9.5 Hz, 3H), 7.54 – 7.48 (m, 2H), 7.45 (s, 1H), 7.30 – 7.26 (m, 1H), 7.26 – 7.21 (m, 2H), 7.17 (d, *J* = 6.7 Hz, 1H), 7.09 – 7.04 (m, 2H), 6.53 (tq, *J* = 7.7, 1.7 Hz, 1H), 2.73 (t, *J* = 7.5 Hz, 2H), 2.35 (qd, *J* = 7.5, 2.1 Hz, 2H). **^{19}F NMR (376 MHz, $CDCl_3$):** δ -65.51 (s, 3F). **^{13}C NMR (101 MHz, $CDCl_3$):** δ 140.60, 136.01, 135.96, 135.90, 135.85 (q, *J* = 5.60 Hz), 133.03, 129.65, 129.17, 128.64, 128.57, 128.28, 128.10, 127.77, 127.66, 127.22, 126.64, 126.42, 126.32, 124.94, 122.22, 119.60 (q, *J* = 272.74 Hz), 35.01, 30.39. **HRMS (ESI, m/z)**: calculated for $C_{21}H_{18}F_3$ $[M+H]^+$:327.1361, found: 327.1373. Rf = 0.6 (PE).

1-methoxy-4-(1,1,1-trifluoro-5-phenylpent-2-en-2-yl)benzene

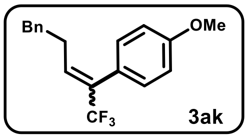

3ak was obtained as a colorless oil (18.7 mg, 0.061 mmol, yield: 61%, *E/Z* = 98:1), **^{1}H NMR (400 MHz, CDCl$_3$):** δ 7.30 – 7.26 (m, 1H), 7.26 (s, 1H), 7.23 – 7.17 (m, 1H), 7.09 (d, *J* = 7.3 Hz, 2H), 7.00 (d, *J* = 9.0 Hz, 2H), 6.90 – 6.84 (m, 2H), 6.42 (tq, *J* = 7.5, 1.7 Hz, 1H), 3.82 (s, 3H), 2.70 (t, *J* = 7.6 Hz, 2H), 2.36 – 2.28 (m, 2H). **^{19}F NMR (376 MHz, CDCl$_3$):** δ -65.94 (s, 3F). **^{13}C NMR (101 MHz, CDCl$_3$):** δ 159.62, 140.75, 135.52，135.47, 135.42, 135.36(q, J = 5.67 Hz), 131.89, 131.60, 131.31, 131.01(q, J = 29.46 Hz), 130.93, 128.54, 127.66, 126.27, 124.96, 124.37, 122.25, 119.51(q, J = 273.31 Hz), 113.84, 55.32, 35.04, 30.26. **HRMS (ESI, m/z)**: calculated for $C_{18}H_{17}F_3NaO$ [M+Na]$^+$: 329.1129, found: 329.1123. Rf = 0.5 (PE).

1-fluoro-4-(1,1,1-trifluoro-5-phenylpent-2-en-2-yl)benzene

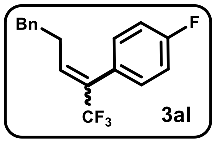

3al was obtained as a colorless oil (26.2 mg, 0.089 mmol, yield: 89%, *E/Z* = 30:1), **^{1}H NMR (400 MHz, CDCl$_3$):** δ 7.31 – 7.26 (m, 2H), 7.21 (t, *J* = 7.3 Hz, 1H), 7.09 – 6.96 (m, 6H), 6.46 (tq, *J* = 7.6, 1.7 Hz, 1H), 2.71 (t, *J* = 7.5 Hz, 2H), 2.29 (dddd, *J* = 9.6, 7.4, 5.3, 2.1 Hz, 2H). **^{19}F NMR (376 MHz, CDCl$_3$):** δ -66.04 (s, 3F), -113.11 (p, *J* = 7.8 Hz, 1F). **^{13}C NMR (101 MHz, CDCl$_3$):** δ 164.04, 161.57, 140.51, 136.16, 136.10, 136.06, 136.00 (q, *J* = 5.44 Hz), 131.61, 131.53, 128.57, 127.43, 126.37, 124.70, 122.00, 119.27 (q, *J* = 272.69 Hz) 115.62, 115.41, 34.90, 30.29. **HRMS (ESI, m/z)**: calculated for $C_{17}H_{15}F_4$ [M+H]$^+$: 295.1110, found: 295.1114. Rf = 0.5 (PE).

1-chloro-4-(1,1,1-trifluoro-5-phenylpent-2-en-2-yl)benzene

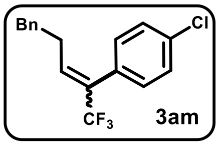

3am was obtained as a colorless oil (18.5 mg, 0.060 mmol, yield: 60%, *E/Z* = 47:1), **^{1}H NMR (400 MHz, CDCl$_3$):** δ 7.34 – 7.27 (m, 4H), 7.24 – 7.19 (m, 1H), 7.07 (d, *J* = 6.7 Hz, 2H), 6.94 (d, *J* = 8.4 Hz, 2H), 6.46 (tq, *J* = 7.5, 1.6 Hz, 1H), 2.71 (t, *J* = 7.5 Hz, 2H), 2.29 (qd, *J* = 7.5, 2.1 Hz, 2H). **^{19}F NMR (376 MHz, CDCl$_3$):** δ -65.88 (s, 3F). **^{13}C NMR (101 MHz, CDCl$_3$):** δ 140.42, 136.28, 136.22, 136.17, 136.12 (q, *J* = 5.56 Hz), 134.65, 131.12, 131.05, 130.74, 130.56, 128.59, 127.32, 126.40, 124.61, 121.90, 119.19 (q, *J* = 273.62 Hz), 34.87, 30.31. **HRMS (ESI, m/z)**: calculated for $C_{17}H_{14}ClF_3Na$ [M+Na]$^+$: 333.0634, found: 333.0630. Rf = 0.5 (PE).

1-bromo-4-(1,1,1-trifluoro-5-phenylpent-2-en-2-yl)benzene

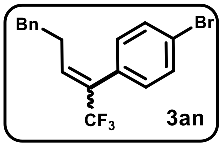

3an was obtained as an orange oil (30.8 mg, 0.087 mmol, yield: 87%, *E/Z* = 27:1), **^{1}H NMR (400 MHz, CDCl$_3$):** δ 7.47 (d, *J* = 8.6 Hz, 2H), 7.29 (t, *J* = 7.1 Hz, 2H), 7.22 (t, *J* = 7.3 Hz, 1H), 7.07 (d, *J* = 7.2 Hz, 2H), 6.88 (d, *J* = 8.3 Hz, 2H), 6.47 (tq, *J* = 7.6, 1.7 Hz, 1H), 2.71 (t, *J* = 7.5 Hz, 2H), 2.30 (dddd, *J* = 9.6, 7.5, 5.3, 2.1 Hz, 2H). **^{19}F NMR (376 MHz, CDCl$_3$):** δ -65.84 (s, 3F). **^{13}C NMR (101 MHz, CDCl$_3$):** δ 140.40, 136.26, 136.21, 136.16, 136.11 (q, *J* = 5.30 Hz), 131.68, 131.41, 131.04, 129.91, 128.59, 127.26, 126.40, 124.54, 122.89, 121.83, 119.11 (q, *J* = 272.96 Hz), 34.88, 30.33. **HRMS (ESI, m/z)**: calculated for $C_{17}H_{15}BrF_3$ [M+H]$^+$: 355.0309, found: 355.0306. Rf = 0.5 (PE).

1-iodo-4-(1,1,1-trifluoro-5-phenylpent-2-en-2-yl)benzene

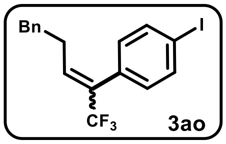

3ao was obtained as an orange oil (19.6 mg, 0.049 mmol, yield: 49%, *E/Z* = 25:1), **^{1}H NMR (400 MHz, CDCl$_3$):** δ 7.69 – 7.65 (m, 2H), 7.29 (d, *J* = 6.9 Hz, 2H), 7.22 (d, *J* = 7.2 Hz, 1H), 7.07 (d, *J* = 6.8 Hz, 2H), 6.75 (d, *J* = 8.3 Hz, 2H), 6.46 (td, *J* = 7.6, 1.7 Hz, 1H), 2.70 (t, *J* = 7.5 Hz, 2H), 2.29 (qd, *J* = 7.5, 2.1 Hz, 2H). **^{19}F NMR (376 MHz, CDCl$_3$):** δ -65.81 (s, 3F). **^{13}C NMR (101 MHz, CDCl$_3$):** δ 140.39, 137.62, 136.21, 136.16, 136.11, 136.05 (q, *J* = 5.47 Hz), 131.56, 131.42, 131.13, 130.64 (t, *J* = 29.57 Hz), 128.58, 127.24, 126.39, 124.51, 121.80, 119.09 (q, *J* = 273.83 Hz), 94.69, 34.88, 30.32. **HRMS (ESI, m/z)**: calculated for $C_{17}H_{14}IF_3Na$ [M+Na]$^+$: 424.9990, found: 424.9999. Rf = 0.5 (PE).

2-isopropyl-5-methylcyclohexyl-4-(1, 1, 1-trifluoro-5-phenylpent-2-en-2-yl)benzoate

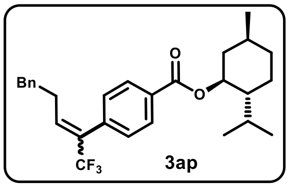

3ap was obtained as an orange oil (32.08 mg, 0.070 mmol, yield: 70%, *E/Z* > 99:1), **^{1}H NMR (400 MHz, CDCl$_3$):** δ 8.03 – 7.98 (m, 2H), 7.30 – 7.27 (m, 1H), 7.26 (s, 1H), 7.24 – 7.18 (m, 1H), 7.13 – 7.05 (m, 4H), 6.49 (td, *J* = 7.6, 1.7 Hz, 1H), 4.94 (td, *J* = 10.9, 4.4 Hz, 1H), 2.71 (t, *J* = 7.5 Hz, 2H), 2.30 (qd, *J* = 7.6, 2.1 Hz, 2H), 2.12 (dd, *J* = 11.1, 4.1 Hz, 1H), 1.96 (pd, *J* = 6.9, 2.7 Hz, 1H), 1.76 – 1.70 (m, 2H), 1.58 (s, 2H), 1.56-1.51 (d, *J* = 12.9 Hz, 1H), 1.16 – 1.05 (m, 2H), 0.93 (dd, *J* = 6.8, 3.1 Hz, 6H), 0.80 (d, *J* = 6.9 Hz, 3H).**^{19}F NMR (376 MHz, CDCl$_3$):** δ -65.55 (s, 3F). **^{13}C NMR (101 MHz, CDCl$_3$):** δ 165.74, 140.37, 136.70, 136.33, 136.22 (t, J = 5.64 Hz), 136.27, 136.22, 130.95, 129.79, 129.66, 128.60, 128.55, 127.30, 126.41, 124.57, 121.84, 119.15 (q, J = 274.26 Hz), 75.12, 47.30, 41.02, 34.88, 34.37, 31.53, 30.30, 26.52, 23.61, 22.16, 20.90, 16.54. **HRMS (ESI, m/z)**: calculated for $C_{28}H_{33}F_3NaO_2$ [M+Na]$^+$: 481.2330, found: 481.2337. Rf = 0.45 (PE/EA=20:1).

4-methyl-7-(1,1,1-trifluoro-5-phenylpent-2-en-2-yl)-2*H*-chromen-2-one

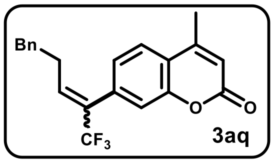

3aq was obtained as a colorless oil (15.4 mg, 0.043 mmol, yield: 39%, *E/Z* > 99:1), **^{1}H NMR (400 MHz, CDCl$_3$):** δ 7.56 (d, *J* = 8.2 Hz, 1H), 7.30 – 7.19 (m, 3H), 7.08 – 7.02 (m, 3H), 6.96 (dd, *J* = 8.1, 1.6 Hz, 1H), 6.53 (tq, *J* = 7.6, 1.6 Hz, 1H), 6.33 (q, *J* = 1.3 Hz, 1H), 2.73 (t, *J* = 7.5 Hz, 2H), 2.44 (d, *J* = 1.4 Hz, 3H), 2.33 (qd, *J* = 7.6, 2.1 Hz, 2H).**^{19}F NMR (376 MHz, CDCl$_3$) :** δ -65.46 (s, 3F). **^{13}C NMR (101 MHz, CDCl$_3$) :** δ 160.54, 153.28, 151.96, 140.19, 137.03, 136.97, 136.92, 136.87, 135.90, 128.64, 128.51, 127.16, 126.52, 125.63, 124.68, 124.46, 121.72, 120.04, 119.00 (q, *J* = 273.44 Hz), 118.42, 115.78, 34.80, 30.39, 18.70. **HRMS (ESI, m/z)**: calculated for $C_{21}H_{18}F_3O_2$ [M+H]$^+$: 359.1259, found: 359.1263. Rf = 0.45 (PE/EA=20:1).

ethyl -4-(1,1,1-trifluoro-3-methyl-5-phenylpent-2-en-2-yl)benzoate

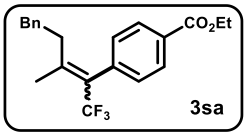

3sa was obtained as a colorless oil (18.8 mg, 0.052 mmol, yield: 52%, *E/Z* = 5.5:1), **^{1}H NMR (400 MHz, CDCl$_3$)**: δ 7.98 (d, *J* = 8.5 Hz, 2H), 7.25–7.16 (m, 3H), 6.99 (d, *J* = 8.3 Hz, 2H), 6.95–6.90 (m, 2H), 4.39 (q, *J* = 7.1 Hz, 2H), 2.66 (t, *J* = 7.6 Hz, 2H), 2.19–2.12 (m, 5H), 1.40 (t, *J* = 7.1 Hz, 3H); **^{19}F NMR (376 MHz, CDCl$_3$):** δ -56.1 (s, 3F); **^{13}C NMR (101 MHz, CDCl$_3$):** δ 166.3, 146.5 (q, *J* = 2.7 Hz), 140.5, 139.9, 129.9, 129.8, 129.4, 128.4, 128.3, 127.6 (q, *J* = 30.4 Hz), 126.2, 123.7 (q, *J* = 275.2 Hz), 61.1, 38.7, 33.7, 18.8, 14.3; **HRMS (ESI, m/z):** calculated for $C_{21}H_{22}F_3O_2$ [M+H]$^+$: 363.1572, found: 363.1570. Rf = 0.4 (PE/EA=20:1).

ethyl-4-(1,1,1-trifluoro-3-phenethylhex-2-en-2-yl)benzoate

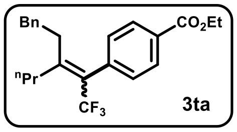

3ta was obtained as a colorless oil (24.1 mg, 0.0618mmol, yield: 61.8%, *E/Z* = 1:1.7), **^{1}H NMR (400 MHz, CDCl$_3$):**δ 8.08 – 7.98 (m, 3H), 7.37 – 7.26 (m, 3H), 7.26 – 7.15 (m, 6H), 7.11 – 7.05 (m, 2H), 6.88 (dd, *J* = 7.1, 2.4 Hz, 2H), 4.40 (q, *J* = 7.2 Hz, 4H), 2.88 – 2.79 (m, 1H), 2.72 – 2.64 (m, 1H), 2.62 – 2.55 (m, 2H), 2.45 (td, *J* = 8.5, 7.7, 2.8 Hz, 2H), 2.22 – 2.13 (m, 2H), 1.95 – 1.85 (m, 1H), 1.67 – 1.56 (m, 3H), 1.41 (t, *J* = 7.1 Hz, 6H), 1.03 (t, *J* = 7.3 Hz, 3H), 0.74 (t, *J* = 7.3 Hz, 2H). **^{19}F NMR (376 MHz, CDCl$_3$):** δ -55.35, -55.55 (s, 3F). **^{13}C NMR (101 MHz, CDCl$_3$)** δ 166.42, 151.21, 141.39, 140.75, 140.36, 140.23, 130.15, 130.06, 130.00, 129.66, 129.63, 129.53, 128.65, 128.53, 128.42, 128.31, 127.68, 126.35, 126.27,124.94, 122.21, 119.47 (q, *J* = 274.29 Hz), 61.21, 35.98, 35.75, 35.44, 34.24, 33.92, 22.25, 21.41, 14.44, 14.38, 14.15. **HRMS (ESI, m/z)**: calculated for $C_{23}H_{26}F_3O_2$ [M+H]$^+$: 391.1885, found: 391.1888. Rf = 0.4 (PE/EA=20:1).

ethyl -4-(1,1,1-trifluoro-4-(4-fluorophenyl)-3-methylbut-2-en-2-yl)benzoate

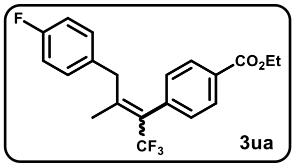

3ua was obtained as a colorless oil (19.5 mg, 0.0533 mmol, yield: 53.3%, *E/Z* = 7:1), **^{1}H NMR (400 MHz, $CDCl_3$):** δ 8.06 (dd, *J* = 8.3, 1.8 Hz, 2H), 7.31 (d, *J* = 8.2 Hz, 2H), 7.06 – 6.90 (m, 4H), 4.38 (q, *J* = 7.1 Hz, 2H), 3.22 (d, *J* = 2.0 Hz, 2H), 1.96 (s, 3H), 1.39 (t, *J* = 7.1 Hz, 3H). **^{19}F NMR (376 MHz, $CDCl_3$):** δ -55.80 (s, 3F), -116.11 (p, *J* = 7.3 Hz, 1F). **^{13}C NMR (101 MHz, $CDCl_3$)** δ 166.26, 145.86, 139.91, 133.41, 130.08, 130.00, 129.87, 129.83, 125.13, 122.40, 119.64 (q, *J* = 274.31 Hz), 115.70, 115.49, 61.26, 41.38, 18.70, 14.43. **HRMS (ESI, m/z)**: calculated for $C_{20}H_{19}F_4O_2$ [M+H]$^+$:367.1321, found: 393.67.1317. Rf = 0.4 (PE/EA=20:1).

ethyl -4-(4-(4-chlorophenyl)-1,1,1-trifluoro-3-methylbut-2-en-2-yl)benzoate

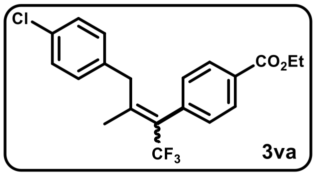

3va was obtained as a colorless oil (16.8 mg, 0.0502 mmol, yield: 502%, *E/Z* = 8:1), **^{1}H NMR (400 MHz, $CDCl_3$):** δ 8.08 – 8.04 (m, 2H), 7.33 – 7.28 (m, 2H), 7.25 (d, *J* = 1.9 Hz, 2H), 6.96 (d, *J* = 8.4 Hz, 2H), 4.38 (q, *J* = 7.1 Hz, 2H), 3.21 (s, 2H), 1.97 (q, *J* = 2.6 Hz, 3H), 1.39 (t, *J* = 7.1 Hz, 3H). **^{19}F NMR (376 MHz, $CDCl_3$):** δ -55.84 (s, 3F), **^{13}C NMR (101 MHz, $CDCl_3$)** δ 166.24, 145.53, 145.50, 145.47, 145.44 (q, *J* =3.2 Hz), 139.83, 136.23, 132.59, 130.39, 130.25, 130.03, 129.93, 129.88, 128.88, 127.84, 125.10, 122.36, 119.60 (q, *J* =275.10 Hz), 61.26, 41.55, 18.78, 18.76, 18.74, 18.71 (q, *J* =2.18 Hz), 14.42. **HRMS (ESI, m/z)**: calculated for $C_{20}H_{18}ClF_3O_2$ [M]$^+$: 382.0947, found: 382.0951. Rf = 0.4 (PE/EA=20:1).

ethyl -4-(1,1,1-trifluoro-3-methyldodec-2-en-2-yl)benzoate

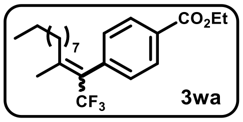

3wa was obtained as a colorless oil (14.6 mg, 0.047mmol, yield: 47%, *E/Z* = 53:1), **^{1}H NMR (400 MHz, $CDCl_3$):** δ 8.07 – 8.01 (m, 2H), 7.22 (d, *J* = 8.2 Hz, 2H), 4.39 (q, *J* = 7.2 Hz, 2H), 2.04 (q, *J* = 2.6 Hz, 3H), 1.88 – 1.80 (m, 2H), 1.40 (t, *J* = 7.1 Hz, 3H), 1.28 – 1.02 (m, 14H), 0.86 (t, *J* = 7.0 Hz, 3H). **^{19}F NMR (376 MHz, $CDCl_3$):** δ -55.73 (s, 3F), **^{13}C NMR (101 MHz, $CDCl_3$):** δ 166.43, 148.39, 148.36,148.33 (q, J =3.03 Hz), 148.30, 140.45, 130.13, 129.95, 129.59, 128.05, 126.70, 125.18, 122.58, 119.87 (q, J =273.49 Hz), 61.16, 36.67, 31.92, 29.80, 29.43, 29.39, 29.32, 27.63, 22.74, 18.72, 14.43, 14.19. **HRMS (ESI, m/z)**: calculated for $C_{22}H_{32}F_3O_2$ [M+ H]$^+$: 385.2354, found: 385.2345. Rf = 0.4 (PE/EA=20:1).

ethyl -4-(5-(benzo[d][1,3]dioxol-5-yl)-1,1,1-trifluoro-3-methylpent-2-en-2-yl)benzoate

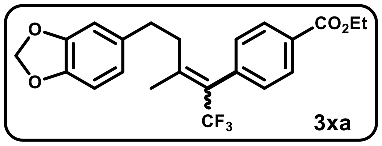

3xa was obtained as a colorless oil (23.6 mg, 0.058 mmol, yield: 58%, *E/Z* = 6:1), **^{1}H NMR (400 MHz, $CDCl_3$):** δ 8.02 – 7.98 (m, 2H), 7.05 (d, *J* = 8.3 Hz, 2H), 6.67 (d, *J* = 7.8 Hz, 1H), 6.40 – 6.36 (m, 2H), 5.91 (s, 2H), 4.39 (q, *J* = 7.1 Hz, 2H), 2.60 – 2.54 (t, *J* = 7.6 Hz 2H), 2.11 (q, *J* = 2.7 Hz, 3H), 1.62 (q, *J* = 2.3 Hz, 2H), 1.40 (t, *J* = 7.2, 1.4 Hz, 3H). **^{19}F NMR (376 MHz, $CDCl_3$):** δ -55.97 (s, 3F), **^{13}C NMR (101 MHz, $CDCl_3$)** δ 166.37, 147.65, 146.64, 146.61, 146.58, 146.55 (q, J =3.2 Hz), 145.97, 139.97, 134.97, 134.41, 130.03, 129.96, 129.75, 129.56, 126.85, 124.04, 121.21 (q, *J* =282.62 Hz), 121.15, 118.11, 108.79, 108.27, 100.95, 61.20, 39.00, 33.59, 29.81, 18.94, 14.44. **HRMS (ESI, m/z)**: calculated for $C_{23}H_{24}F_3O_4$ $[M+H]^+$: 421.1627, found: 421.1630. Rf = 0.4 (PE/EA=20:1).

ethyl -4-(1,1,1-trifluoro-3,7-dimethylocta-2,6-dien-2-yl)benzoate

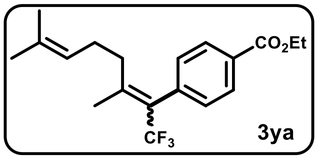

3ya was obtained as an colorless oil (13.3 mg, 0.039 mmol, yield: 39%, *E/Z* = 5:1), **^{1}H NMR (400 MHz, $CDCl_3$):** δ 8.04 (d, *J* = 2.4 Hz, 2H), 7.22 (d, *J* = 8.0 Hz, 2H), 4.93 – 4.81 (m, 1H), 4.39 (q, *J* = 7.1 Hz, 2H), 2.08 – 2.04 (m, 3H), 2.01 (m, 2H), 1.88 (m, 2H), 1.62 (s, 3H), 1.46 (s, 3H), 1.40 (t, *J* = 7.1 Hz, 3H). **^{19}F NMR (376 MHz, $CDCl_3$) :** δ -55.86 (s, 3F). **^{13}C NMR (101 MHz, $CDCl_3$) :** δ 166.43, 147.66, 140.37, 132.85, 130.18, 129.96, 129.70, 129.56, 128.03, 125.29, 122.56, 122.77, 119.83 (q, *J* = 274.74 Hz), 61.17, 36.74, 26.15, 25.72, 18.79, 17.55, 14.43. **HRMS (ESI, m/z)**: calculated for $C_{19}H_{24}F_3O_2$ $[M+H]^+$: 341.1728, found: 341.1734. Rf = 0.4 (PE/EA=20:1).

ethyl 4-(6-chloro-3-(3-chloropropyl)-1,1,1-trifluorohex-2-en-2-yl)benzoate

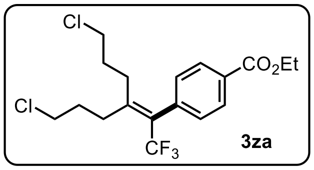

3za was obtained as an orange oil (23.8 mg, 0.060 mmol, yield: 60%), **^{1}H NMR (400 MHz, $CDCl_3$)**: δ 8.06 (d, *J* = 8.5 Hz, 2H), 7.22 (d, *J* = 8.2 Hz, 2H), 4.39 (q, *J* = 7.1 Hz, 2H), 3.62 (t, *J* = 6.4 Hz, 2H), 3.31 (t, *J* = 6.4 Hz, 2H), 2.60–2.51 (m, 2H), 2.08–1.98 (m, 4H), 1.81–1.71 (m, 2H), 1.40 (t, *J* = 7.1 Hz, 3H); **^{19}F NMR (376 MHz, $CDCl_3$):** δ -55.8 (s, 3F); **^{13}C NMR (101 MHz, $CDCl_3$):** δ 166.1, 149.2 (q, *J* = 2.8 Hz), 139.4, 130.3, 129.7, 129.5, 128.8 (q, *J* = 29.9 Hz), 123.4 (q, *J* = 275.0 Hz), 61.2, 44.6, 44.2, 31.6, 31.2, 30.8, 29.5, 14.3; **HRMS (ESI, m/z):** calculated for $C_{18}H_{22}Cl_2F_3O_2$ $[M+H]^+$: 397.0949, found: 397.0943. Rf = 0.4 (PE/EA=20:1).

ethyl 4-(1-(4,4-dimethylcyclohexylidene)-2,2,2-trifluoroethyl)benzoate

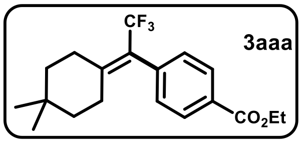

3aaa was obtained as a colorless oil (29.3 mg, 0.0861 mmol, yield: 86.1%), **^{1}H NMR (400 MHz, CDCl$_3$):** δ 8.05 – 8.02 (m, 2H), 7.24 – 7.20 (m, 2H), 4.38 (q, *J* = 7.1 Hz, 2H), 2.54 (td, *J* = 6.3, 1.6 Hz, 2H), 1.96 (td, *J* = 6.3, 1.7 Hz, 2H), 1.54 – 1.50 (m, 2H), 1.39 (t, *J* = 7.2 Hz, 3H), 1.32 – 1.28 (m, 2H), 0.98 (s, 6H). **^{19}F NMR (376 MHz, CDCl$_3$):** δ -54.10 (s, 3F), **^{13}C NMR (101 MHz, CDCl$_3$)** δ 166.42, 152.62, 152.59, 152.55, 152.52 (q, J =3.1 Hz), 140.24, 130.16, 129.90, 129.60, 129.56, 127.95, 125.22, 122.49, 119.76 (q, J =275.73 Hz), 61.16, 40.59, 30.17, 29.03, 28.07, 27.44, 14.44. **HRMS (ESI, m/z)**: calculated for $C_{19}H_{24}F_3O_4$ [M+H]$^+$: 341.1728, found: 341.1731. Rf = 0.4 (PE/EA=20:1).

ethyl 4-(2,2,2-trifluoro-1-(4-phenylcyclohexylidene)ethyl)benzoate

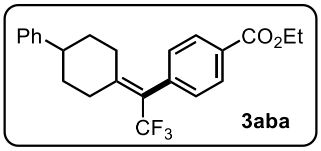

3aba was obtained as an orange oil (16.3 mg, 0.042 mmol, yield: 42%), **^{1}H NMR (400 MHz, CDCl$_3$)**: δ 8.07 (d, *J* = 8.6 Hz, 2H), 7.33–7.20 (m, 7H), 4.39 (q, *J* = 7.1 Hz, 2H), 3.22–3.15 (m, 1H), 2.80 (tt, *J* = 12.0, 3.4 Hz, 1H), 2.32–2.12 (m, 3H), 2.02–1.90 (m, 2H), 1.83–1.69 (m, 1H), 1.59–1.46 (m, 1H), 1.40 (t, *J* = 7.1 Hz, 3H); **^{19}F NMR (376 MHz, CDCl$_3$):** δ -54.1 (s, 3F); **^{13}C NMR (101 MHz, CDCl$_3$):** δ 166.2, 151.0 (q, *J* = 2.7 Hz), 145.5, 139.9, 130.5, 130.0, 129.6, 128.9 (q, *J* = 28.6 Hz), 128.4, 126.7, 126.3, 123.7 (q, *J* = 275.7 Hz), 61.1, 43.9, 35.3, 35.1, 32.8, 31.3, 14.3; **HRMS (ESI, m/z):** calculated for $C_{23}H_{23}F_3NaO_2$ [M+Na]$^+$: 411.1548, found: 411.1537. Rf = 0.4 (PE/EA=20:1).

***tert*-butyl-4-(1-(4-(ethoxycarbonyl)phenyl)-2,2,2-trifluoroethylidene)piperidine-1-carboxylate**

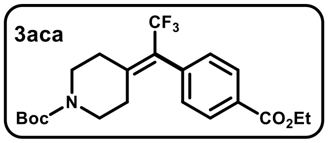

3aca was obtained as a pale yellow solid (19.4 mg, 0.047 mmol, yield: 47%), **^{1}H NMR (400 MHz, CDCl$_3$):** δ 8.08 – 8.03 (m, 2H), 7.24 – 7.19 (m, 2H), 4.39 (q, *J* = 7.1 Hz, 2H), 3.57 (t, *J* = 5.9 Hz, 2H), 3.37 (t, *J* = 5.9 Hz, 2H), 2.65 (t, *J* = 6.0 Hz, 2H), 2.06 (t, *J* = 6.4 Hz, 2H), 1.45 (s, 9H), 1.39 (t, *J* = 7.1 Hz, 3H). **^{19}F NMR (376 MHz, CDCl$_3$) :** δ -54.53 (s, 3F). **^{13}C NMR (101 MHz, CDCl$_3$) :** δ 166.22, 154.64, 147.48, 147.44, 147.42, 147.39 (q, *J* = 2.68 Hz), 139.15, 130.35, 130.05, 129.80, 127.62, 124.90, 122.17, 119.41 (q, *J* = 275.20 Hz), 80.11, 61.25, 32.10, 30.88, 28.47, 14.43. **HRMS (ESI, m/z)**: calculated for $C_{21}H_{27}F_3NO_4$ [M+H]$^+$: 414.1892, found: 414.1891. Rf = 0.5 (PE/EA=10:1).

4-(2,2,2-trifluoro-1-(4-methoxyphenyl)ethylidene)tetrahydro-2H-thiopyran

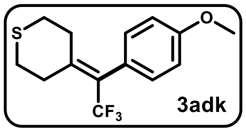

3adk was obtained as an orange oil (16.3 mg, 0.042 mmol, yield: 42%), **^{1}H NMR (400 MHz, CDCl$_3$)** δ 7.06 – 6.99 (m, 2H), 6.91 – 6.86 (m, 2H), 3.81 (s, 3H), 2.88 – 2.81 (m, 4H), 2.66 – 2.61 (m, 2H), 2.34 (ddt, *J* = 8.3, 3.4, 1.5 Hz, 2H). **^{19}F NMR (376 MHz, CDCl$_3$):** δ -54.51 (s, 3F). **^{13}C NMR (101 MHz, CDCl$_3$)** δ 159.31, 148.44, 130.92, 128.04, 125.29, 122.52, 119.79 (q, *J* = 273.69 Hz), 113.93, 55.32, 34.81, 33.62, 30.89, 30.76. **HRMS (ESI, m/z)**: calculated for $C_{14}H_{16}F_3OS$ $[M+H]^+$:289.0874, found: 289.0875. Rf = 0.5 (PE).

ethyl 4-(1-cyclododecylidene-2,2,2-trifluoroethyl)benzoate

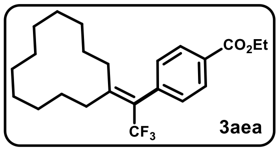

3aea was obtained as a white solid (17.85 mg, 0.0451 mmol, yield: 45.1%), **^{1}H NMR (400 MHz, CDCl$_3$):** δ 8.04 (d, *J* = 8.3 Hz, 2H), 7.23 (d, *J* = 8.2 Hz, 2H), 4.39 (q, *J* = 7.1 Hz, 2H), 2.36 (d, *J* = 7.5 Hz, 2H), 1.87 – 1.78 (m, 2H), 1.67 – 1.56 (m, 4H), 1.44 – 1.36 (m, 8H), 1.32 (s, 9H). **^{19}F NMR (376 MHz, CDCl$_3$):** δ -55.09 (s, 3F), **^{13}C NMR (101 MHz, CDCl$_3$)** δ 166.47, 153.15, 147.18, 140.68, 130.12, 129.94, 129.58, 127.94, 125.23, 122.52, 119.72 (q, *J* = 273.04 Hz), 29.81, 27.52, 26.47, 26.05, 25.83, 24.02, 23.61, 22.39, 22.23, 21.75, 21.65, 14.45. **HRMS (ESI, m/z)**: calculated for $C_{23}H_{32}F_3O_2$ $[M+H]^+$:397.2354, found: 397.2359. Rf = 0.5 (PE/EA=20:1).

1-(1,1,1-trifluoro-3-methyl-5-phenylpent-2-en-2-yl)-4-(trifluoromethyl)benzene

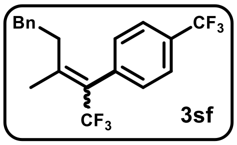

3sf was obtained as a colorless oil (27.2 mg, 0.076 mmol, yield: 76%, *E/Z* = 7:1), **^{1}H NMR (400 MHz, CDCl$_3$):** δ 7.54 (d, *J* = 7.8 Hz, 2H), 7.25 – 7.17 (m, 3H), 6.99 (d, *J* = 8.0 Hz, 2H), 6.95 – 6.89 (m, 2H), 2.70 – 2.65 (t, *J* = 7.63 Hz 2H), 2.21 – 2.16 (dq, *J* = 1.5 Hz, *J* = 8.0 Hz 2H), 2.16-2.12 (q, *J* = 2.67Hz, 3H). **^{19}F NMR (376 MHz, CDCl$_3$):** δ -56.10 (s, 3F), -62.52 (s, 3F). **^{13}C NMR (101 MHz, CDCl$_3$)** δ 146.99, 146.96, 146.96, 146.90 (q, *J* =3.03 Hz), 140.51, 138.98, 130.43, 130.37, 128.66, 128.59, 128.47, 128.21, 127.84, 125.51, 125.12, 122.77, 122.39, 120.07 (q, J =272.33 Hz), 119.67 (q, J =274.29 Hz), 126.39, 125.51, 125.33, 125.30, 125.26, 125.22, 122.77, 38.74, 33.68, 18.87. **HRMS (ESI, m/z)**: calculated for $C_{19}H_{16}F_6Na$ $[M+ Na]^+$: 381.1054, found: 381.1053. Rf = 0.5 (PE).

2-isopropyl-5-methylcyclohexyl-4-(1,1,1-trifluoro-3-methyl-5-phenylpent-2-en-2-yl)benzoate

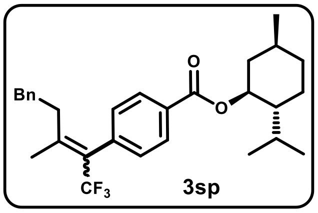

3sp was obtained as an orange oil (19.8 mg, 0.042mmol, yield: 42%, *E/Z* = 6.7:1), **^{1}H NMR (400 MHz, $CDCl_3$):** δ 7.97 (d, *J* = 8.0 Hz, 2H), 7.25 – 7.16 (m, 3H), 7.00 (d, *J* = 8.0 Hz, 2H), 6.97 – 6.90 (m, 2H), 4.94 (td, *J* = 10.9, 4.4 Hz, 1H), 2.71 – 2.63 (t, *J* = 8.1 Hz, 2H), 2.18 (t, *J* = 8.1 Hz, 2H), 2.13 (q, *J* = 2.7 Hz, 3H), 1.97 (td, *J* = 7.0, 2.8 Hz, 1H), 1.78 – 1.69 (m, 2H), 1.64 (q, *J* = 2.4 Hz, 1H), 1.57 (s, 2H), 1.53 (t, *J* = 3.0 Hz, 1H), 1.16 – 1.06 (m, 2H), 0.93 (dd, *J* = 6.7, 1.9 Hz, 6H), 0.81 (d, *J* = 6.9 Hz, 3H). **^{19}F NMR (376 MHz, $CDCl_3$) :** δ -56.06 (s, 3F). **^{13}C NMR (101 MHz, $CDCl_3$) :** δ 165.86, 140.63, 139.88, 129.95, 129.58, 128.54, 128.42, 127.93, 126.32, 125.21, 122.47, 119.72 (q, *J* = 275.87 Hz), 75.06, 47.33, 41.04, 38.73, 34.38, 33.80, 31.53, 29.80, 26.54, 23.67, 22.15, 20.88, 16.59. **HRMS (ESI, m/z)**: calculated for $C_{29}H_{36}F_3O_2$ $[M+H]^+$: 473.2667, found: 473.2671. Rf = 0.4 (PE/EA=20:1).

1-(4-(1,1,1-trifluoro-4-(4-fluorophenyl)-3-methylbut-2-en-2-yl)phenyl)ethan-1-one

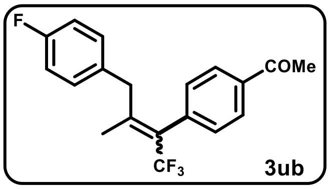

3ub was obtained as a colorless oil (15.5 mg, 0.046 mmol, yield:46%, *E/Z* = 5:1), **^{1}H NMR (400 MHz, $CDCl_3$):** δ 7.98 (d, *J* = 8.3 Hz, 2H), 7.34 (d, *J* = 8.2 Hz, 2H), 7.00 – 6.93 (m, 4H), 3.22 (s, 2H), 2.61 (s, 3H), 1.97 (q, *J* = 2.6 Hz, 3H). **^{19}F NMR (376 MHz, $CDCl_3$):** δ -55.81 (s, 3F), -116.04 (s, 1F). **^{13}C NMR (101 MHz, $CDCl_3$):** δ 197.66, 162.99, 160.55, 145.96, 140.21, 136.78, 133.35, 130.33, 130.27, 130.06, 129.99, 128.64, 128.58, 127.85, 125.11, 122.29, 119.67 (q, *J* =275.31 Hz), 115.71, 115.50, 41.41, 26.75, 18.74, 18.72, 18.69, 18.67. **HRMS (ESI, m/z)**: calculated for $C_{19}H_{16}F_4NaO$ $[M+ Na]^+$: 359.1035, found: 359.1039. Rf = 0.35 (PE/EA=20:1).

dimethyl -5-(1,1,1-trifluoro-4-(4-fluorophenyl)-3-methylbut-2-en-2-yl)isophthalate

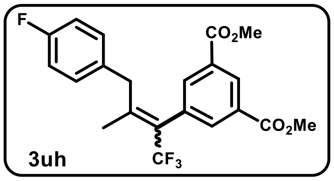

3uh was obtained as a colorless oil (18.9 mg, 0.046 mmol, yield: 46%, *E/Z* = 7:1), **^{1}H NMR (400 MHz, $CDCl_3$):** δ 8.67 (t, *J* = 1.6 Hz, 1H), 8.09 (d, *J* = 1.6 Hz, 2H), 6.98 – 6.95 (m, 4H), 3.95 (s, 6H), 3.21 (s, 2H), 1.99 (q, *J* = 2.5 Hz, 3H). **^{19}F NMR (376 MHz, $CDCl_3$):** δ -55.79 (s, 3F), -116.03 (p, *J* = 7.0, 6.4 Hz, 1F). **^{13}C NMR (101 MHz, $CDCl_3$):** δ 165.85, 147.08, 135.35, 134.08, 133.10, 131.18, 130.51, 130.45, 130.09, 130.01, 127.77, 125.02, 122.29 (q, *J* =276.10 Hz), 115.77, 115.56, 52.66, 41.51, 18.88. **HRMS (ESI, m/z)**: calculated for $C_{21}H_{19}F_4O_4$ $[M+ H]^+$: 411.1219, found: 411.1230. Rf = 0.4 (PE/EA=10:1).

ethyl-4-(-1,1,1-trifluoro-3-((((R)-2,5,7,8-tetramethyl-2-((4R,8R)-4,8,12-trimethyltridecyl)chroman-6-yl)oxy)methyl)pent-2-en-2-yl)benzoate

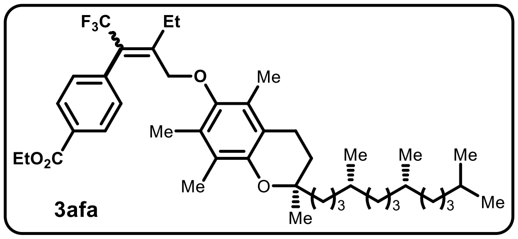

3afa was obtained as a colorless oil (33.6 mg, 0.047 mmol, yield: 47%, *E/Z* = 7:1), **^{1}H NMR (400 MHz, $CDCl_3$):** δ 7.97 (d, *J* = 8.1 Hz, 2H), 7.14 (d, *J* = 8.0 Hz, 2H), 4.38 (q, *J* = 7.1 Hz, 2H), 3.97 (d, *J* = 1.7 Hz, 2H), 2.87 – 2.74 (m, 2H), 2.48 (t, *J* = 6.8 Hz, 2H), 2.20 (d, *J* = 17.3 Hz, 1H), 2.14 – 2.07 (m, 1H), 2.00 (s, 3H), 1.95 (s, 3H), 1.90 (s, 3H), 1.75 (dp, *J* = 20.0, 6.9 Hz, 2H), 1.59 (s, 2H), 1.56 – 1.48 (m, 3H), 1.40 (t, *J* = 7.2 Hz, 3H), 1.31 (t, *J* = 7.4 Hz, 3H), 1.24 (d, *J* = 8.4 Hz, 10H), 1.19 (s, 3H), , 0.85 (dd, *J* = 12.2, 6.6 Hz, 16H). **^{19}F NMR (376 MHz, $CDCl_3$):** δ -56.44 (s, 3F). **^{13}C NMR (101 MHz, $CDCl_3$):** δ 166.24, 149.43, 148.10, 147.37, 138.95, 130.29, 129.88, 129.77, 129.58, 127.73, 125.79, 124.98, 122.24, 119.50 (q, *J* =275.80 Hz), 123.09, 117.66, 70.97, 61.23, 40.17, 40.10, 39.45, 37.63, 37.53, 37.47, 37.36, 32.87, 32.77, 31.24, 31.18, 29.81, 28.08, 24.91, 24.53, 23.87, 23.14, 22.83, 22.73, 21.11, 20.67, 19.84, 19.77, 19.72, 19.71, 19.67, 14.42, 13.75, 12.78, 11.91, 11.83. **HRMS (ESI, m/z)**: calculated for $C_{44}H_{66}F_3O4$ $[M+H]^+$: 715.4913, found: 715.4929. Rf = 0.5 (PE/EA=20:1).

(*E*)-(5,5,5-trifluoropent-3-en-1-yl)benzene

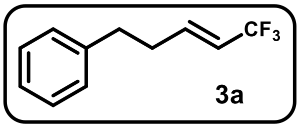

3a was obtained as a colorless oil (8.8 mg, 0.044 mmol, yield: 44%), **^{1}H NMR (400 MHz, $CDCl_3$):** δ 7.41 – 7.32 (m, 2H), 7.30 – 7.20 (m, 3H), 6.47 (dddt, *J* = 15.9, 9.1, 4.5, 2.3 Hz, 1H), 5.68 (dddt, *J* = 12.7, 6.4, 3.2, 1.7 Hz, 1H), 2.80 (dd, *J* = 9.1, 6.6 Hz, 2H), 2.51 (dtq, *J* = 9.3, 6.8, 2.8, 2.3 Hz, 2H). **^{19}F NMR (376 MHz, $CDCl_3$):** δ -63.98, -63.96 (d, *J* = 6.63 Hz, 3F). **^{13}C NMR (101 MHz, $CDCl_3$)** δ 140.60, 139.84, 139.77, 139.71, 139.64 (q, *J* = 6.36 Hz), 128.63, 128.45, 127.10, 126.38, 124.42, 121.75, 119.58, 119.25, 119.07 (q, *J* = 269.44 Hz), 118.92, 118.59 (q, *J* = 33.40 Hz), 34.42, 33.29. **HRMS (ESI, m/z)**: calculated for $C_{11}H_{11}F_3$ $[M]^+$: 200.0813, found: 200.0826. Rf = 0. 6 (PE).

7. Mechanistic study

7.1 Control experiment

Control experiments with respect to the reaction scope when reactions were carried out without photocatalyst or with no photocatalyst and no light irradiation

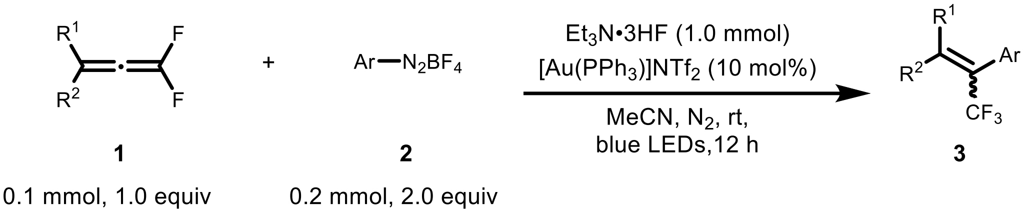

$[Au(PPh_3)]NTf_2$ (10 mol%, 0.01 mmol) was added to an oven-dried 10 mL Schlenk Tube equipped with a stir bar in the glove box, then the reaction tube was removed out of the glove box. Under the atmosphere of nitrogen, *gem*-difluoroallene **1** (1.0 equiv, 0.1 mmol), aryl diazonium **2** (2.0 equiv, 0.2 mmol), MeCN (1.0 mL) and Et_3N-3HF (10.0 equiv, 1.0 mmol) were added to the reaction tube. After stirring 12 hours at room temperature under 5 W blue light irradiation, the reaction mixture was passed through a short pad of celite and rinsed with EtOAc. The filtrate was evaporated under reduced pressure and the yield and *E*/*Z* ratio of product **3** was determined by ^{19}F NMR with 1-iodine-4-trifluoromethylbenzene as internal standard.

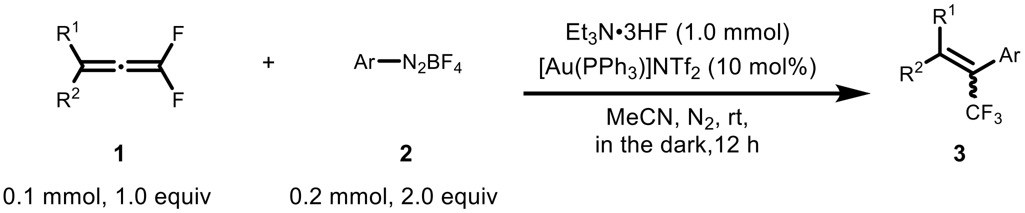

$[Au(PPh_3)]NTf_2$ (10 mol%, 0.01 mmol) was added to an oven-dried 10 mL Schlenk Tube equipped with a stir bar in the glove box, then the reaction tube was removed out of the glove box. Under the atmosphere of nitrogen, *gem*-difluoroallene **1** (1.0 equiv, 0.1 mmol), aryl diazonium **2** (2.0 equiv, 0.2 mmol), MeCN (1.0 mL) and Et_3N-3HF (10.0 equiv, 1.0 mmol) were added to the reaction tube. After stirring 12 hours in the dark at room temperature, the reaction mixture was passed through a short pad of celite and rinsed with EtOAc. The filtrate was evaporated under reduced pressure and the yield and *E*/*Z* ratio of product **3** was determined by ^{19}F NMR with 1-iodine-4-trifluoromethylbenzene as internal standard.

The results of these control experiments were summarized in the following form:

Substrate	Reaction conditions	*E*/*Z* ratio of product **3**	Yield of product **3** (%)
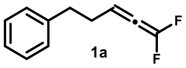 1a	Standard condition	16:1[a]	86[b]
	No photocatalyst	24:1	57
	No photocatalyst and no light	>99:1	43
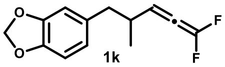 1k	Standard condition	15:1[a]	67[b]
	No photocatalyst	34:1	38
	No photocatalyst and no light	43:1	26
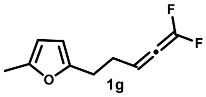 1g	Standard condition	16:1[a]	56[b]
	No photocatalyst	22:1	1
	No photocatalyst and no light	>99:1	trace
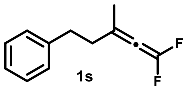 1s	Standard condition	5.5:1[a]	52[b]
	No photocatalyst	7:1	5
	No photocatalyst and no light	9:1	5
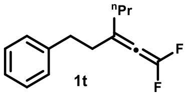 1t	Standard condition	1:1.7[a]	62[b]
	No photocatalyst	1:1.9	30
	No photocatalyst and no light	1:1.7	24
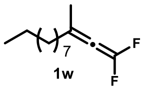 1w	Standard condition	16:1[a]	56[b]
	No photocatalyst	22:1	1
	No photocatalyst and no light	>99:1	trace

(Note: [a]The *E*/*Z* ratio of product **3** was determined by the analysis of ^{19}F NMR of isolated product; [b]The yield of product **3** was isolated yield.)

Standard conditions and thermal control experiments:

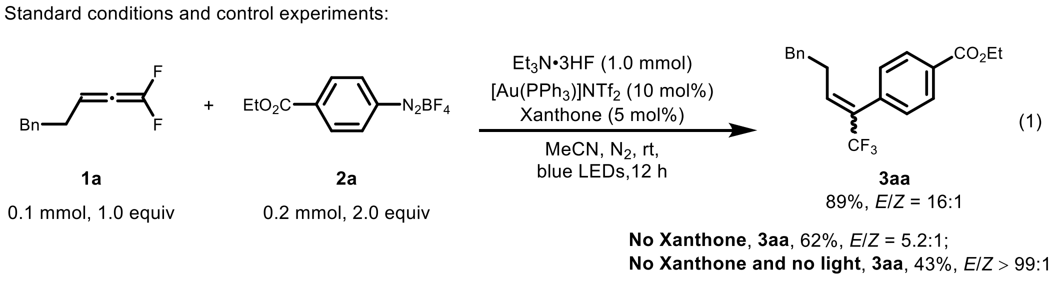

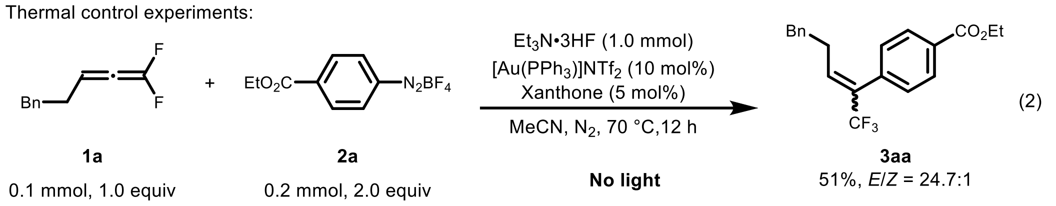

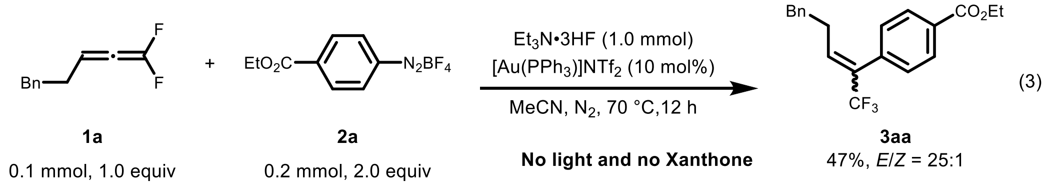

Procedure for the thermal control experiments:

$[Au(PPh_3)]NTf_2$ (10 mol%, 0.01 mmol) was added to an oven-dried 10 mL Schlenk Tube equipped with a stir bar in the glove box, then the reaction tube was removed out of the glove box. Under the atmosphere of nitrogen, *gem*-difluoroallene **1a** (1.0 equiv, 0.1 mmol), aryl diazonium **2a** (2.0 equiv, 0.2 mmol), xanthone (0.005 mmol) or without xanthone, MeCN (1.0 mL) and Et_3N-3HF (10.0 equiv, 1.0 mmol) were added to the reaction tube. After stirring 12 hours in the dark at 70 ℃, the reaction mixture was passed through a short pad of celite and rinsed with EtOAc. The filtrate was evaporated under reduced pressure and the yield and *E*/*Z* ratio of product **3aa** was determined by ^{19}F NMR with 1-iodine-4-trifluoromethylbenzene as internal standard.

The *E*/*Z* ratio of product 3aa monitoring experiments:

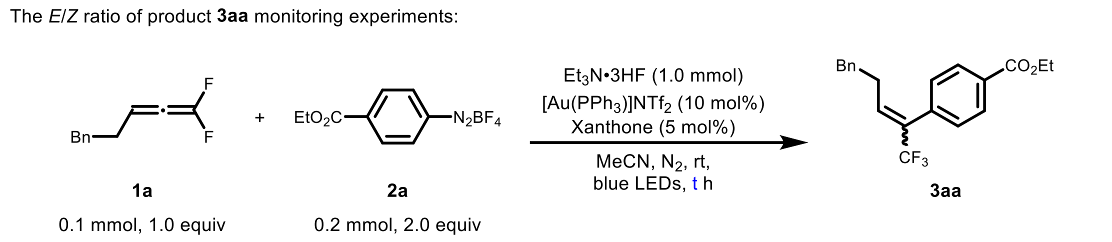

t (h)	*E*/*Z* ratio of product **3aa**	t (h)	*E*/*Z* ratio of product **3aa**	t (h)	*E*/*Z* ratio of product **3aa**
1	35:1	5	40:1	9	41:1
2	38:1	6	39:1	10	38:1
3	37:1	7	36:1	11	38:1
4	38:1	8	37:1	12	37:1

Experiment procedure: $[Au(PPh_3)]NTf_2$ (10 mol%, 0.01 mmol) was added to an oven-dried 10 mL Schlenk Tube equipped with a stir bar in the glove box, then the reaction tube was removed out of the glove box. Under the atmosphere of nitrogen, *gem*-difluoroallene **1a** (1.0 equiv, 0.1 mmol), aryl diazonium **2a** (2.0 equiv, 0.2 mmol), xanthone (0.005 mmol), MeCN (1.0 mL) and Et_3N-3HF (10.0 equiv, 1.0 mmol) were added to the reaction tube. The resulting reaction mixture was stirred at RT under 5 W blue light irradiation. After the indicated time, 30 μL reaction mixture was taken out from the tube and diluted with 0.5 mL $CDCl_3$ in NMR tube. And the *E*/*Z* ratio of product **3aa** was immediately determined by ^{19}F NMR of the reaction mixture when reaction proceeded 1 h, 2 h, 3 h, 4 h, 5 h, 6 h, 7 h, 8 h, 9 h, 10 h, 11 h, 12 h.

E/*Z*-isomerisation experiments of product 3aa

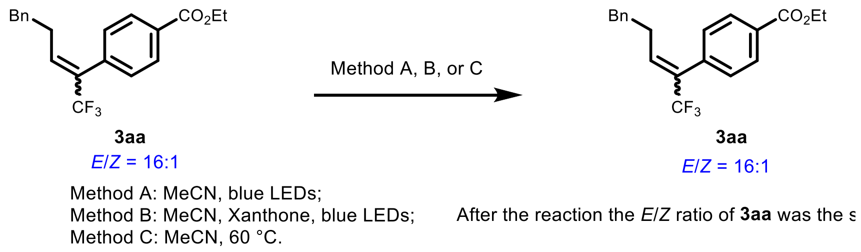

Experiment procedure:

Method A: Product **3aa** (0.1 mmol) was added to an oven-dried 10 mL Schlenk Tube equipped with a stir bar, then MeCN (1.0 mL) was added to the reaction tube under the atmosphere of nitrogen. The resulting reaction mixture was stirred at RT under 5 W blue light irradiation for 12

h. Solvent was removed under reduced pressure and the *E*/*Z* ratio of the residue was determined by ^{19}F NMR.

Method B: Product **3aa** (0.1 mmol) and xanthone (0.005 mmol) were added to an oven-dried 10 mL Schlenk Tube equipped with a stir bar, then MeCN (1.0 mL) was added to the reaction tube under the atmosphere of nitrogen. The resulting reaction mixture was stirred at RT under 5 W blue light irradiation for 12 h. Solvent was removed under reduced pressure and the *E*/*Z* ratio of the residue was determined by ^{19}F NMR.

Method C: Product **3aa** (0.1 mmol) was added to an oven-dried 10 mL Schlenk Tube equipped with a stir bar, then MeCN (1.0 mL) was added to the reaction tube under the atmosphere of nitrogen. The resulting reaction mixture was stirred at 60 ℃ for 12 h in the dark. Solvent was removed under reduced pressure and the *E*/*Z* ratio of the residue was determined by ^{19}F NMR.

The analysis of ^{19}F NMR indicted that no *E*/*Z*-isomerisation occurred during the above reactions.

Control experiment to test the stability of stereoisomer 3ai to isomerisation

Stereoisomer isomerisation experiment:

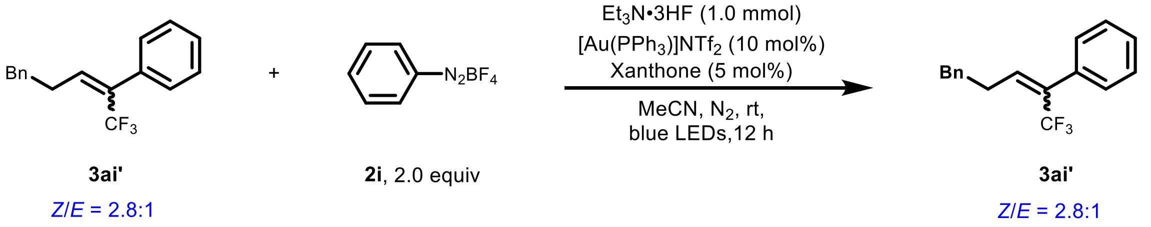

After the reaction the *Z*/*E* ratio of **3ai'** was the same as before.

Experiment procedure: [Au(PPh$_3$)]NTf$_2$ (10 mol%, 0.01 mmol) was added to an oven-dried 10 mL Schlenk Tube equipped with a stir bar in the glove box, then the reaction tube was removed out of the glove box. Under the atmosphere of nitrogen, stereoisomer **3ai'**[6] (1.0 equiv, 0.1 mmol), aryl diazonium **2i** (2.0 equiv, 0.2 mmol), xanthone (0.005 mmol), MeCN (1.0 mL) and Et$_3$N-3HF (10.0 equiv, 1.0 mmol) were added to the reaction tube. The resulting reaction mixture was stirred at RT under 5 W blue light irradiation. After the indicated time, the *Z*/*E* ratio of the obtained **3ai'** after the reaction was immediately determined by ^{19}F NMR.

The investigation of counteranions of aryldiazonium salt and additive

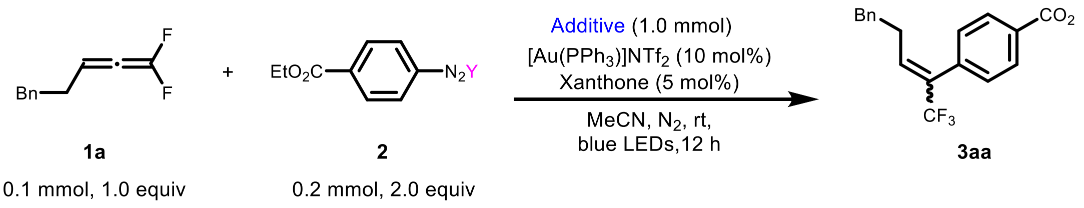

Entry	Y	Additive	NMR yield of **3aa**	*E*/*Z* ratio of **3aa**
1	BF_4	Et_3N•3HF	86%[a]	16:1[b]
2	BPh_4	Et_3N•3HF	5%	*Z* only
3	Cl	Et_3N•3HF	3%	4:1
4	HSO_4	Et_3N•3HF	trace	-
5	OTf	Et_3N•3HF	52%	> 99:1
6	BF_4	-	29%	> 99:1
7	BF_4	HBF_4•Et_2O	NDP	-
8	BF_4	HCl (1 M in 1,4-dioxane)	NDP	-

To an oven-dried 10 mL Schlenk Tube equipped with a stir bar add $[Au(PPh_3)]NTf_2$ (10 mol%, 0.01 mmol) in the glove box, then remove the reaction tube out of the glove box, under the atmosphere of nitrogen, Xanthone (5 mol%, 0.005 mmol), *gem*-difluoroallene **1a** (1.0 equiv, 0.1 mmol), aryl diazonium **2** (2.0 equiv, 0.2 mmol), MeCN (1.0 mL) and additive (10.0 equiv, 1.0 mmol) were added. After stirring 12 hours at room temperature under 5 W blue light irradiation, use TLC to monitor the reaction, then the reaction mixture was passed through a short pad of celite and rinsed with EtOAc. The filtrate was evaporated under reduced pressure and the yield and *E*/*Z* ratio of product **3aa** was determined by ^{19}F NMR with 1-iodine-4-trifluoromethylbenzene as internal standard.

(Note: [a]The *E*/*Z* ratio of product **3aa** was determined by the analysis of ^{19}F NMR of isolated product; [b]The yield of product **3aa** was isolated yield.)

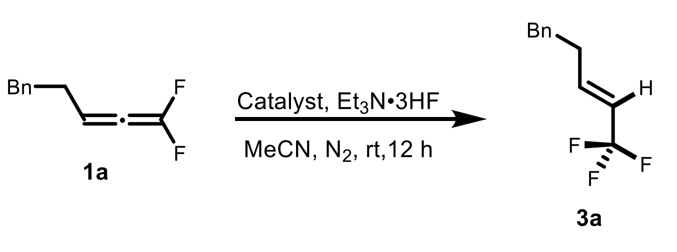

Entry	Catalyst	Yield
1	-	0%
2	$[Au(PPh_3)]NTf_2$	11%
3	$AuCl_3$	21%
4	$AuCl_3/AgBF_4$	34%
5	$AuCl_3/PPh_3/AgBF_4$	42%

To an oven-dried 10 mL Schlenk Tube equipped with a stir bar add catalyst in the glove box, then remove the reaction tube out of the glove box, under the atmosphere of nitrogen, Xanthone (5 mol%, 0.005 mmol), difluoroallene **1a** (1.0 equiv., 0.1 mmol), Et_3N-3HF (10.0 equiv., 1.0 mmol) and MeCN (1.0 mL) were added. After stirring 12 hours at room temperature under 5 W blue light irradiation, use TLC to monitor the reaction, then the reaction mixture was passed through a short pad of celite and rinsed with EtOAc. The filtrate was evaporated under reduced pressure and the crude residue was purified by column chromatography on silica gel (EtOAc/PE =0 to 2:100) to afford the desired fluoroarylation product **3a**. [Note: entry 2 $[Au(PPh_3)]NTf_2$ (10 mol%, 0.01 mmol); entry 3 $AuCl_3$ (10 mol%, 0.01 mmol); entry 4 $AuCl_3$ (10 mol%, 0.01 mmol), $AgBF_4$ (60 mol%, 0.06 mmol); entry 5 $AuCl_3$ (10 mol%, 0.01 mmol), PPh_3 (10 mol%, 0.01 mmol), $AgBF_4$ (60 mol%, 0.06 mmol)]

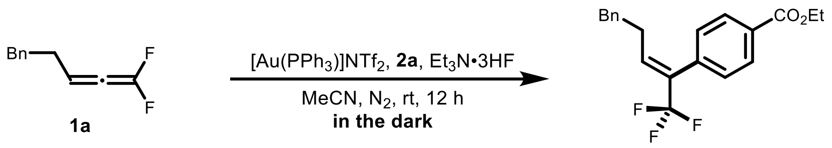

3aa, 43%, *E/Z* > 99:1

To an oven-dried 10 mL Schlenk Tube equipped with a stir bar add $[Au(PPh_3)]NTf_2$ (10 mol%, 0.01 mmol) in the glove box, then remove the reaction tube out of the glove box, under the atmosphere of nitrogen, difluoroallene **1a** (1.0 equiv., 0.1 mmol), aryl diazonium **2a** (2.0 equiv., 0.2 mmol), Et_3N-3HF (10.0 equiv., 1.0 mmol) and MeCN (1.0 mL) were added. After stirring 12 hours at room temperature in the dark, use TLC to monitor the reaction, then the reaction mixture was passed through a short pad of celite and rinsed with EtOAc. The filtrate was evaporated under reduced pressure and the crude residue was purified by column chromatography on silica gel (EtOAc/PE =1:20 to 1:10) to afford the desired fluoroarylation product **3aa**.

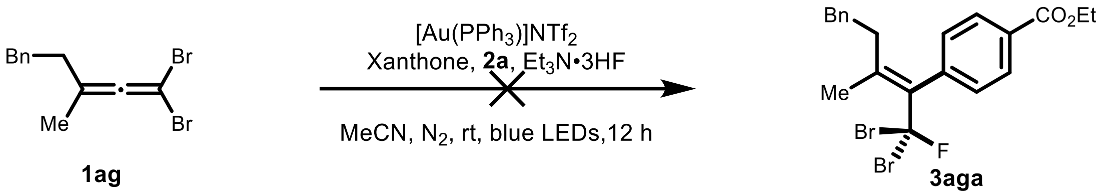

To an oven-dried 10 mL Schlenk Tube equipped with a stir bar add [Au(PPh$_3$)]NTf$_2$ (10 mol%, 0.01 mmol) in the glove box, then remove the reaction tube out of the glove box, under the atmosphere of nitrogen, Xanthone (5 mol%, 0.005 mmol), *gem*-dibromoallene **1ag** (1.0 equiv., 0.1 mmol), aryl diazonium **2a** (2.0 equiv., 0.2 mmol), Et$_3$N-3HF (10.0 equiv., 1.0 mmol) and MeCN (1.0 mL) were added. After stirring 12 hours at room temperature under 5 W blue light irradiation, use TLC to monitor the reaction. No reaction was occurred via the TLC and crude NMR analysis.

7.2 Study of Au(III) intermediate

7.2.1 Preparation of Au(III) intermediate

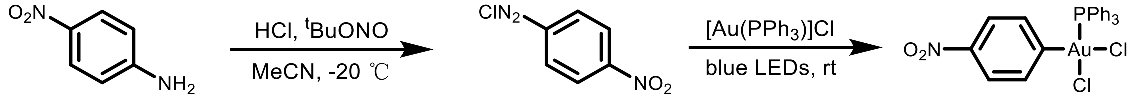

Following the literature procedure:[5]

Into a pear-shaped bottle equipped with a stir bar, a solution of 4-nitroaniline (0.5 mmol, 1.0 equiv.) in 2.0 mL MeCN was added under N_2 atmosphere, Then the mixture was cooled to -20 °C and 0.25 mL HCl solution (4 mol/L in dioxane) was added slowly and stirred for 5 min, next $^{t-}$BuONO (0.6 mmol, 1.2 equiv.) was added dropwise at the same temperature. The reaction was stirred for another 15 min. Then [Au(PPh_3)]Cl (0.5 mmol, 1.0 equiv.) was added under N_2 atmosphere. The bottle was sealed with screw cap and the mixture was stirring for 12 h upon the irradiation of 5 W Blue LEDs. The product was precipitated by the addition of ether. After separation, the Au(III) complex was obtained as white solid. The spectra data was in agreement with the literature.[5]

7.2.2 Fluoroarylation process with prepared Au(III) intermediate

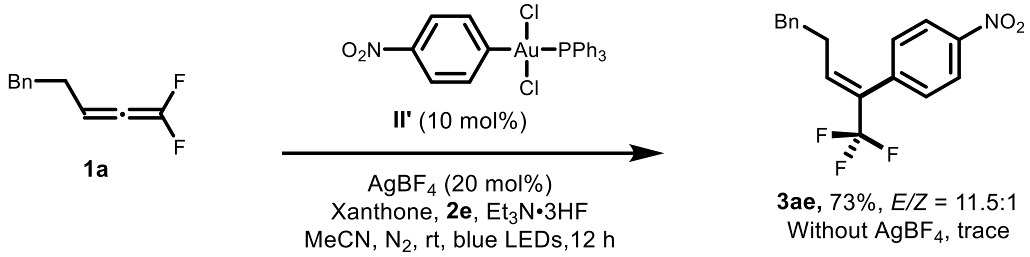

An oven-dried 10 mL Schlenk Tube containing agitator in the glove box, add gold(III) complex **II'** (10 mol%, 0.01 mmol) and silver tetrafluoroborate (20 mol%, 0.02 mmol) then remove the reaction tube out of the glove box, under the protection of nitrogen, add Xanthone (5 mol%, 0.005 mmol), *gem*-difluoroallene **1a** (1.0 equiv., 0.1 mmol), aryl diazonium **2e** (2.0 equiv., 0.2 mmol), and Et_3N-3HF (10.0 equiv., 1.0 mmol), followed by the reaction solvent MeCN (1.0 mL), seal the reaction tube under nitrogen protection. Reaction at room temperature under 5 W blue light irradiation reaction after 12 hours, TLC test reaction conversion, then use a small amount of silica gel filtration reaction liquid and EtOAc flushing for many times, to get the solution of the stress relief after removing the solvent residual organic matter by column chromatography (EtOAc / PE = 1:20 to 1:10) purified fluorine aromatization product **3ae**.

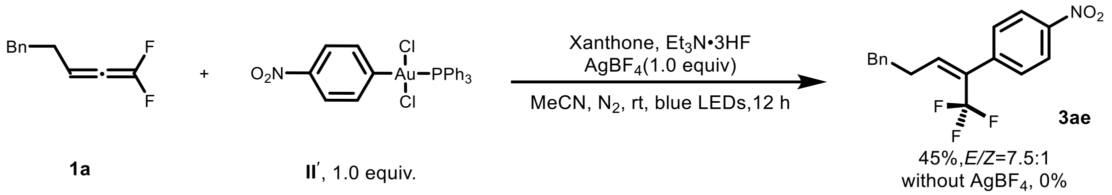

An oven-dried 10 mL Schlenk Tube containing agitator in the glove box, add gold(III) complex **II'** (1.0 equiv., 0.05 mmol) and silver tetrafluoroborate (1.0 equiv., 0.05 mmol) then remove the reaction tube out of the glove box, under the protection of nitrogen, add Xanthone (5 mol%, 0.0025 mmol), *gem*-difluoroallene **1a** (1.0 equiv,0.05 mmol), and Et_3N-3HF (10.0 equiv., 0.5 mmol), followed by the reaction solvent MeCN (1.0 mL), seal the reaction tube under nitrogen protection. Reaction at room temperature under 5 W blue light irradiation reaction after 12 hours, TLC test reaction conversion, then use a small amount of silica gel filtration reaction liquid and EtOAc flushing for many times, to get the solution of the stress relief after removing the solvent residual organic matter by column chromatography (EtOAc / PE = 1:20 to 1:10) purified fluorine aromatization product **3ae**.

Thermal control experiments between allene 1a and Ar-AuIII species II':

Thermal control experiments between allene **1a** and Ar-Au^{III} species **II'**:

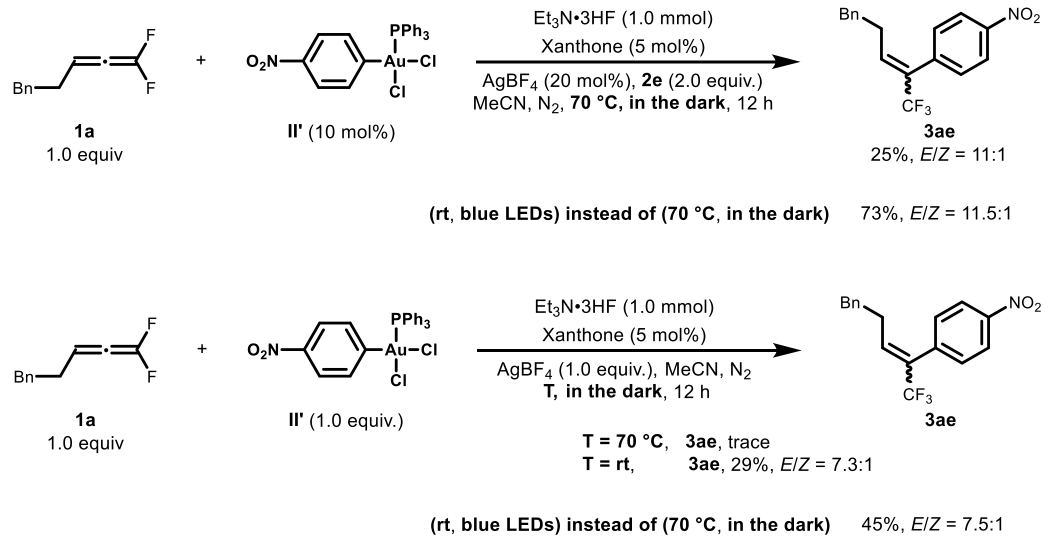

Procedure A: An oven-dried 10 mL Schlenk Tube containing agitator in the glove box, add gold(III) complex **II'** (10 mol%, 0.01 mmol) and silver tetrafluoroborate (20 mol%, 0.02 mmol) then remove the reaction tube out of the glove box, under the protection of nitrogen, add Xanthone (5 mol%, 0.005 mmol), *gem*-difluoroallene **1a** (1.0 equiv., 0.1 mmol), aryl diazonium **2e** (2.0 equiv., 0.2 mmol), and Et_3N-3HF (10.0 equiv., 1.0 mmol), followed by the reaction solvent MeCN

(1.0 mL), seal the reaction tube under nitrogen protection. After stirring 12 hours at 70 ℃ in the dark, use TLC to monitor the reaction, then the reaction mixture was passed through a short pad of celite and rinsed with EtOAc. The filtrate was evaporated under reduced pressure and the yield and *E*/*Z* ratio of product **3ae** was determined by ^{19}F NMR with 1-iodine-4-trifluoromethylbenzene as internal standard.

Procedure B: An oven-dried 10 mL Schlenk Tube containing agitator in the glove box, add gold(III) complex **II'** (1.0 equiv., 0.05 mmol) and silver tetrafluoroborate (1.0 equiv., 0.05 mmol) then remove the reaction tube out of the glove box, under the protection of nitrogen, add Xanthone (5 mol%, 0.0025 mmol), *gem*-difluoroallene **1a** (1.0 equiv,0.05 mmol), and Et_3N-3HF (10.0 equiv., 0.5 mmol), followed by the reaction solvent MeCN (1.0 mL), seal the reaction tube under nitrogen protection. After stirring 12 hours at 70 ℃ or room temperature in the dark, use TLC to monitor the reaction, then the reaction mixture was passed through a short pad of celite and rinsed with EtOAc. The filtrate was evaporated under reduced pressure and the yield and *E*/*Z* ratio of product **3ae** was determined by ^{19}F NMR with 1-iodine-4-trifluoromethylbenzene as internal standard.

7.3 TEMPO quenching experiments

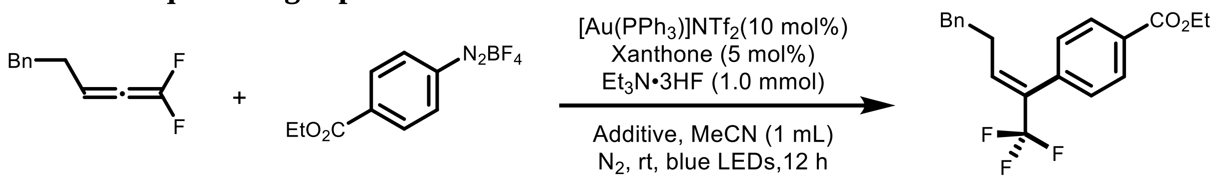

1a, 0.1 mmol **2a**, 0.2 mmol **3aa**

Entry	Additive	Yield of **3aa**[c]	*E*/*Z* of **3aa**[d]
1	None	86%	16/1
2	0. 1 mmol TEMPO	47%	16/1
3	0. 2 mmol TEMPO	2.8%	10.6/1
4	0. 3 mmol TEMPO	ND	-

To an oven-dried 10 mL Schlenk Tube equipped with a stir bar add $[Au(PPh_3)]NTf_2$ (10 mol%, 0.01 mmol) in the glove box, then remove the reaction tube out of the glove box, under the atmosphere of nitrogen, Xanthone (5 mol%, 0.005 mmol), *gem*-difluoroallene **1a** (1.0 equiv., 0.1 mmol), aryl diazonium **2a** (2.0 equiv., 0.2 mmol), TEMPO, MeCN (1.0 mL) and Et_3N-3HF (10.0 equiv., 1.0 mmol) were added. After stirring 12 hours at room temperature under 5 W blue light

irradiation, use TLC to monitor the reaction, then the reaction mixture was passed through a short pad of celite and rinsed with EtOAc. The filtrate was evaporated under reduced pressure and the crude residue was purified by column chromatography on silica gel (EtOAc/PE =1:20 to 1:10) to afford the desired fluoroarylation product **3aa**. [c] The yield of compound **3aa** was determined by ^{19}F NMR with 1-iodine-4-trifluoromethylbenzene as internal standard. [d] The *E*/*Z* ratio of compound **3aa** was determined by ^{19}F NMR.

7.4 Ultraviolet and Visible Spectrum

Figure S1. Ultraviolet and visible spectrum of $[Au(PPh_3)]N(Tf)_2$

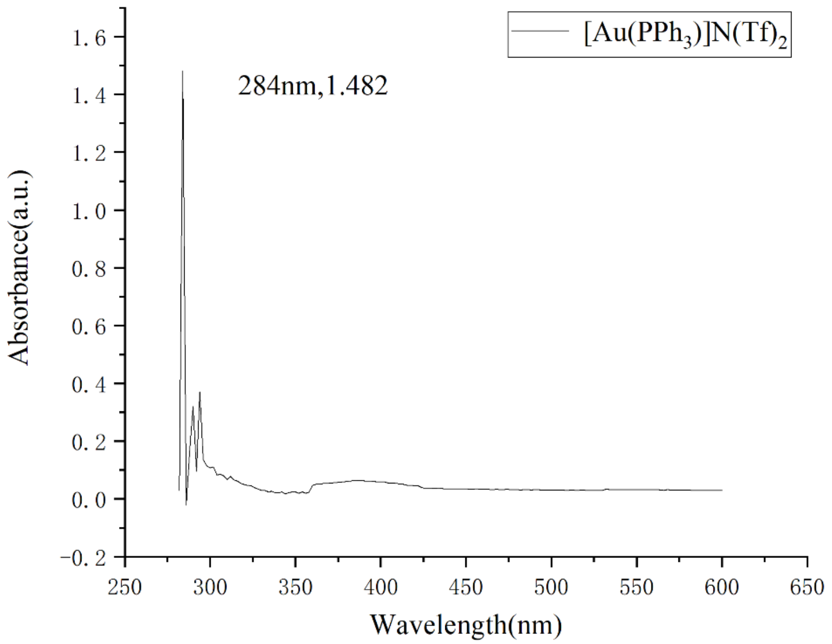

.

Figure S2. Ultraviolet and visible spectrum of Xanthone.

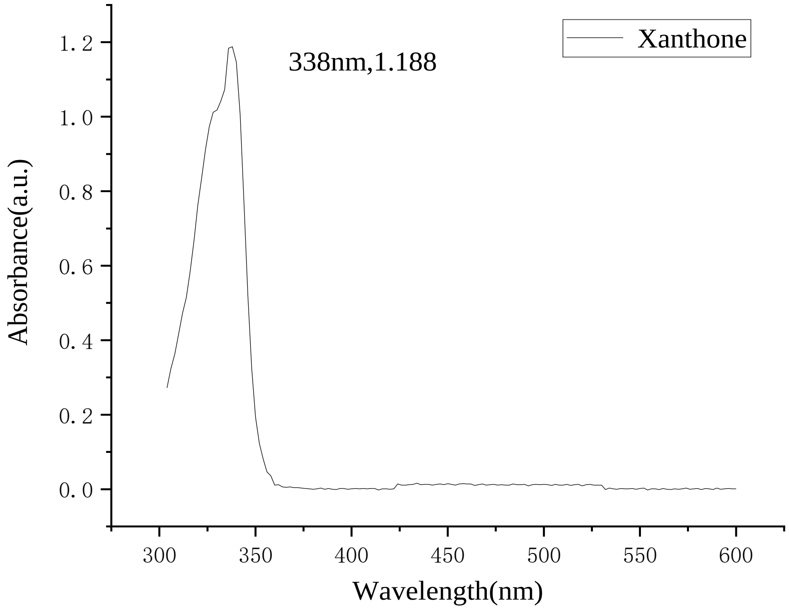

7.5 Emission quenching experiments (Stern-Volmer studies)

Emission intensities were recorded using PerkinElemer LS 55 Fluorescence Spectrometer for all experiments. All Xanthone solutions (0.015 mM) were excited at 283 nm and the emission intensity at 338 nm was collected at room temperature under an inert atmosphere. Samples were prepared by adding solutions of photocatalyst, quencher, and MeCN to obtain a total volume of 3.0 mL in glove box. The sample was shaken for 1 min and then the emission of the sample was collected. The data show that $[Au(PPh_3)]NTf_2$ and aryl diazonium **2a** was competent at quenching the excited state of the photocatalyst.

Table S13. Constant Xanthone, Varied [Au].

Species	Concentration (mM)
Xanthone	0.015
$[Au(PPh_3)]NTf_2$	Varied

[Au] (mM)	Scan 1	Scan 2	Scan 3	Average	I_0/I
0	608.83	605.63	609.60	608.02	1
0.96	477.77	480.83	479.96	479.30	1.27
1.92	411.67	409.45	409.45	410.19	1.4^{8}
2.88	357.78	354.92	355.81	356.17	1.71
3.84	304.81	306.27	303.57	304.88	1.99

Figure S4. Fluorescence spectra of photocatalyst with different concentration of $[Au(PPh_3)]NTf_2$.

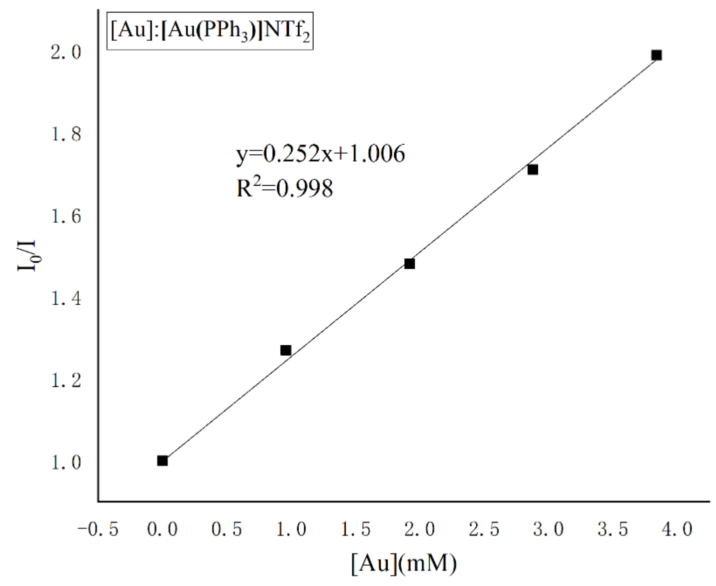

Table S14. Constant Xanthone, Varied 2a.

Species	Concentration (mM)
Xanthone	0.015
**2a**	Varied

[**2a**] (mM)	Scan 1	Scan 2	Scan 3	Average	I_0/I
0	608.83	601.57	599.58	603.32	1
0.96	427.22	436.34	443.16	435.57	1.39
1.92	255.82	256.17	254.89	255.63	2.36
2.88	155.47	153.31	151.63	153.47	3.93

Figure S5. Fluorescence spectra of photocatalyst with different concentration of 2a.

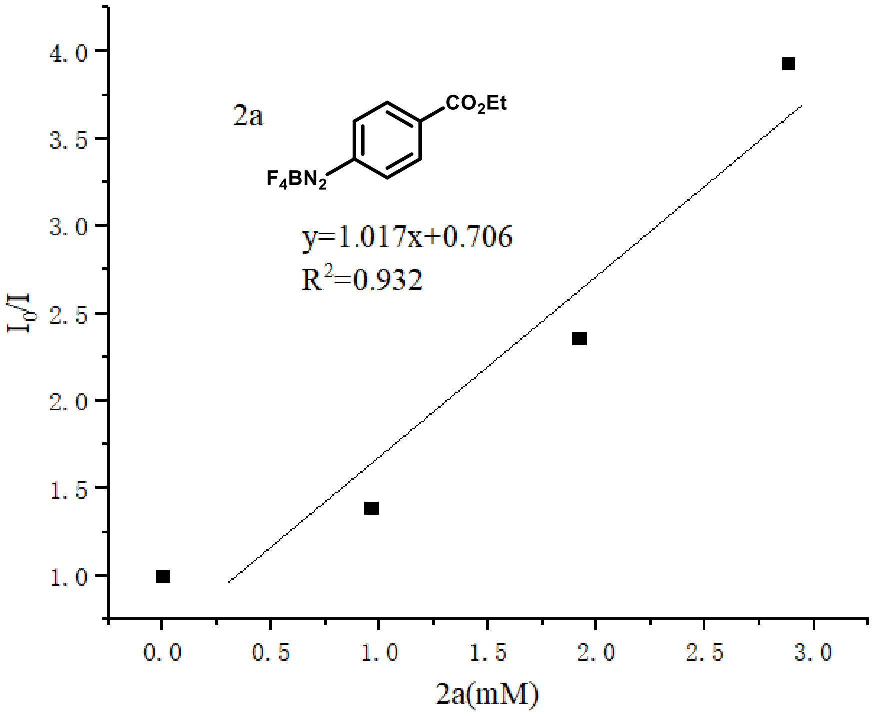

7.6 DFT studies

The DFT calculations were performed with the Gaussian 09 program[7]. Geometries of the minimum energy structures were optimized at the B3-LYP level of theory with the 6-31G (d, p) basis set[8]. Harmonic vibrational frequency calculations were performed for the stationary points to confirm that they were local minima. The single-point energies were obtained using the M06-2X functional with the 6-311G (d, p) basis set with more accurate energy information[9].

(*E*)-**3aa** single-point energy = -1223.40849575 a.u.

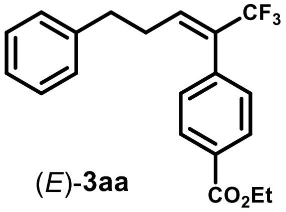

(*E*)-**3aa**

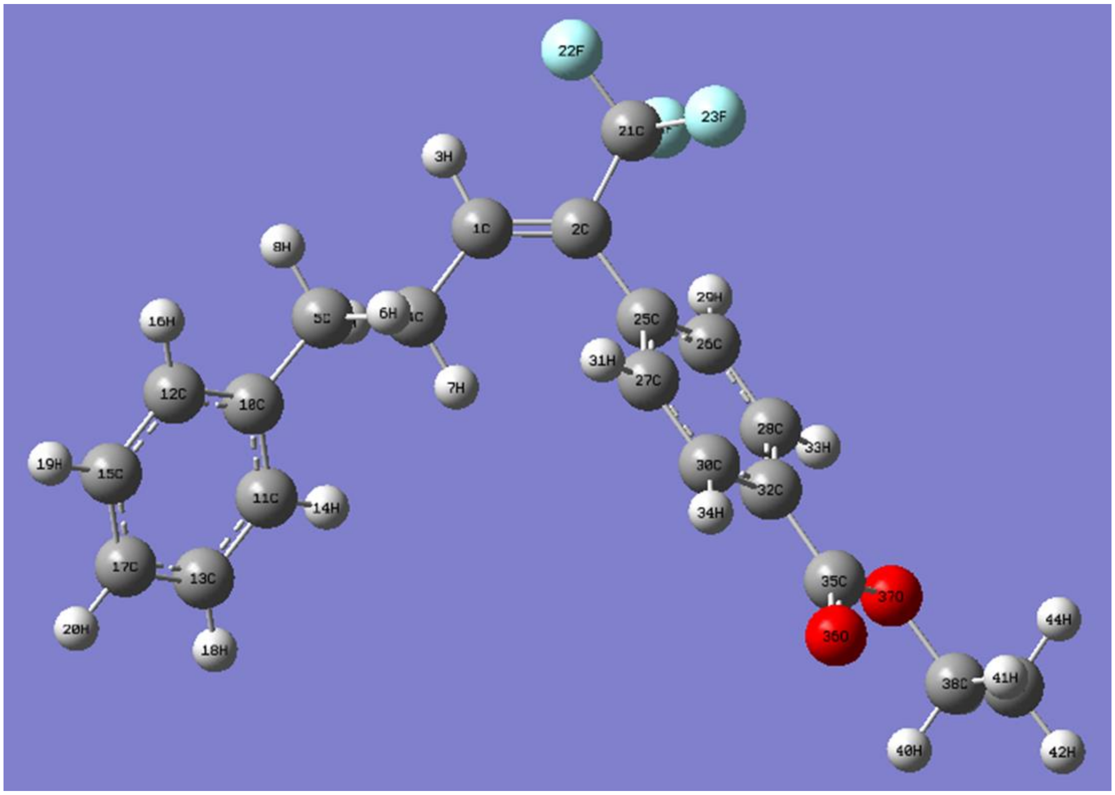

(*E*)-**3aa**:

0 1

C	1.85682700	1.88699200	-0.24519900
C	0.55715200	2.20692700	-0.13154600
H	2.57418600	2.70121100	-0.16851900
C	2.45671600	0.52238000	-0.41714200

C	3.55632400	0.24296100	0.63921200
H	2.91450100	0.45058000	-1.41396700
H	1.69283700	-0.25756500	-0.36694600
H	4.31372100	1.03440600	0.58463800
H	3.10934400	0.30452400	1.63799100
C	4.21096400	-1.10807300	0.44879100
C	3.67483100	-2.25528100	1.04882300
C	5.34630700	-1.24810600	-0.36027600
C	4.25492600	-3.50768600	0.84599900
H	2.79732000	-2.16446800	1.68473400
C	5.93031000	-2.49847200	-0.56578400
H	5.78063400	-0.36751500	-0.82812300
C	5.38518500	-3.63321900	0.03671300
H	3.82661500	-4.38449500	1.32323700
H	6.81342000	-2.58539200	-1.19239400
H	5.84007600	-4.60691300	-0.11955400
C	0.18434300	3.64899200	0.12528200
F	1.23091800	4.49389000	0.00991600
F	-0.76946200	4.07368300	-0.73132900
F	-0.31487100	3.82336200	1.37953400
C	-0.58030500	1.25129800	-0.25185900
C	-1.57123100	1.16149000	0.74100900
C	-0.68607000	0.42170700	-1.38144400
C	-2.62361800	0.26008500	0.61597300
H	-1.51252700	1.79989100	1.61442600
C	-1.73561000	-0.48129400	-1.50739100
H	0.05296700	0.50251100	-2.17184400
C	-2.71243500	-0.57051100	-0.50859500
H	-3.37949900	0.19494200	1.38980500
H	-1.82230100	-1.12220900	-2.37797600
C	-3.81750200	-1.55403800	-0.69693800
O	-3.91902300	-2.29396600	-1.65674600
O	-4.70425000	-1.53597500	0.32272800

C	-5.80965500	-2.46161700	0.21159400
C	-6.68451900	-2.27571400	1.43583500
H	-5.41141900	-3.47852800	0.13929600
H	-6.35003900	-2.25465400	-0.71728200
H	-7.53599300	-2.96160400	1.39035900
H	-6.12505800	-2.48307000	2.35249400
H	-7.06950800	-1.25346700	1.49033800

(*Z*)-**3aa** single-point energy = -1223.40456928 a.u.

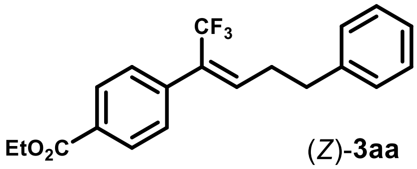

(*Z*)-**3aa**

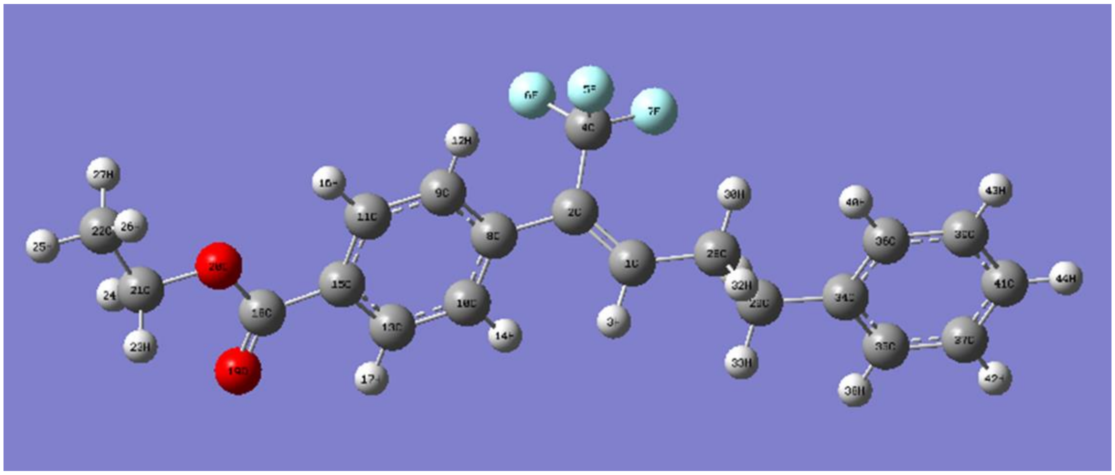

(*Z*)-**3aa**:

0 1

C	1.35251000	-0.27610000	-0.41328200
C	0.36381500	0.63550800	-0.46418900
H	1.02223200	-1.31237500	-0.33926600
C	0.61696600	2.12631800	-0.51934000
F	0.59523300	2.68084700	0.72303500
F	-0.32829100	2.76181700	-1.24397900
F	1.80735800	2.45073700	-1.06922200

C	-1.06628600	0.22051300	-0.41245600
C	-1.99807200	0.91035800	0.38347800
C	-1.50949800	-0.89311400	-1.14620600
C	-3.32145700	0.49007400	0.45683800
H	-1.67985900	1.77277300	0.95819800
C	-2.83056500	-1.31765200	-1.07100300
H	-0.81419600	-1.41406400	-1.79688500
C	-3.74866100	-0.63041000	-0.26792700
H	-4.02849500	1.02526800	1.07975000
H	-3.17660500	-2.17436800	-1.63898600
C	-5.15334700	-1.12666700	-0.23050000
O	-5.55402400	-2.09560800	-0.84717400
O	-5.94509900	-0.37899900	0.57052500
C	-7.32615200	-0.79811700	0.65650200
C	-8.03753900	0.16327600	1.58841800
H	-7.35959700	-1.82879000	1.02325900
H	-7.75845300	-0.79423300	-0.34892900
H	-9.09080700	-0.11959000	1.67887600
H	-7.59015200	0.14500300	2.58630600
H	-7.98839500	1.18711000	1.20692600
C	2.84756200	-0.11364800	-0.41709100
C	3.52877800	-1.07907000	0.58248900
H	3.14948100	0.91134800	-0.20282300
H	3.22305400	-0.33898400	-1.42618300
H	3.16785000	-0.85657300	1.59337500
H	3.21774400	-2.10706300	0.35810400
C	5.03846600	-0.98124200	0.54207100
C	5.78465200	-1.77057600	-0.34321500
C	5.72136700	-0.07311000	1.36181700
C	7.17337700	-1.65548900	-0.41040800
H	5.27275200	-2.48686500	-0.98191500
C	7.11004400	0.04571700	1.29824500
H	5.15939600	0.54312400	2.05976200

C	7.84089300	-0.74529200	0.41047800
H	7.73397500	-2.27991900	-1.10026300
H	7.62107700	0.75318900	1.94503900
H	8.92216800	-0.65639700	0.36202900

(*E*)-**3ai** single-point energy = -956.24235560 a.u.

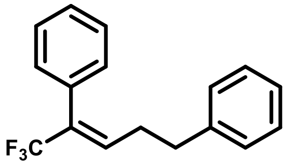

(*E*)-**3ai**

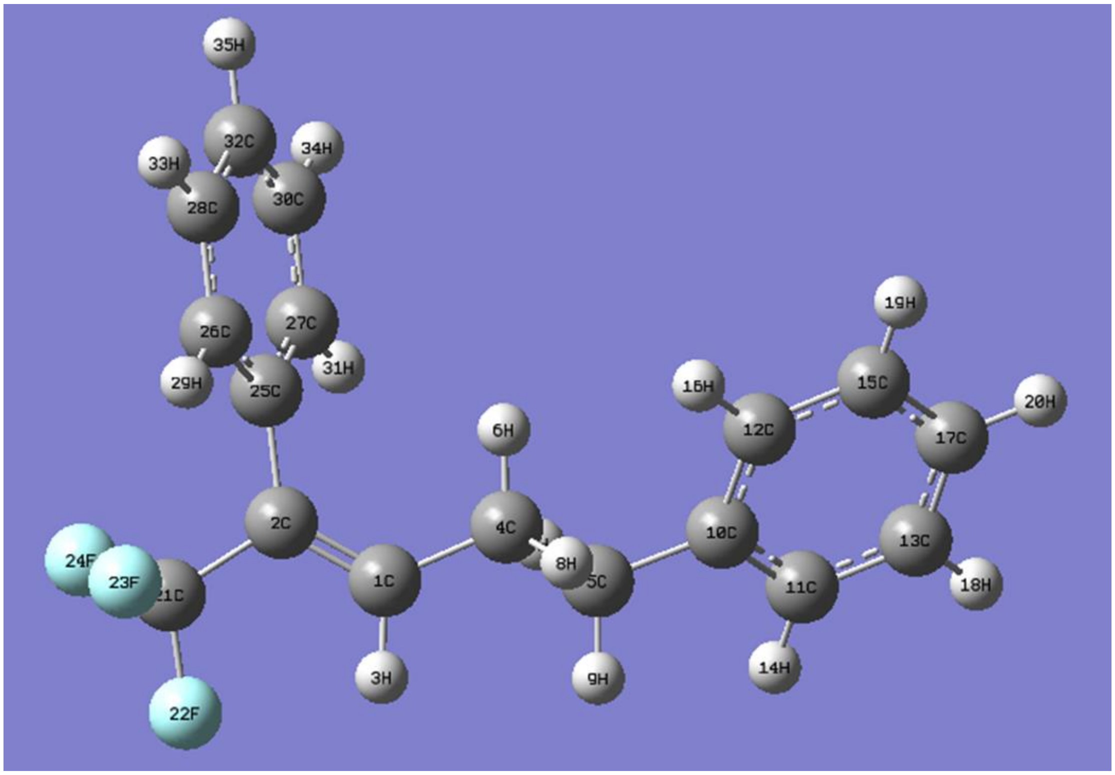

(*E*)-**3ai**:

0 1

C	0.48109300	-1.33340900	0.32804600
C	1.67826100	-0.75512600	0.14157100
H	0.45045100	-2.42075100	0.33817600
C	-0.84748300	-0.65339900	0.48387100

C	-1.89293600	-1.19584000	-0.52367800
H	-0.75385500	0.42949300	0.36932900
H	-1.22769500	-0.83087300	1.49992900
H	-1.53116100	-1.01000200	-1.54131400
H	-1.96739300	-2.28441200	-0.41085300
C	-3.25677600	-0.56698400	-0.33742800
C	-4.19375800	-1.13115200	0.53861700
C	-3.60035600	0.61370300	-1.00922600
C	-5.43834700	-0.53362600	0.73985200
H	-3.94760200	-2.05184200	1.06279200
C	-4.84362000	1.21504600	-0.81139300
H	-2.88782200	1.06251100	-1.69729000
C	-5.76705600	0.64310500	0.06512500
H	-6.15274100	-0.98990100	1.41926300
H	-5.09259000	2.12783400	-1.34521400
H	-6.73667300	1.10800800	0.21748100
C	2.88840600	-1.63663900	-0.05937600
F	2.62448400	-2.94962900	0.11794800
F	3.39939200	-1.51342500	-1.31434200
F	3.88327800	-1.31090000	0.79485900
C	1.93647400	0.71427300	0.14045300
C	2.54988200	1.34867000	-0.95244100
C	1.57058800	1.49348100	1.24954400
C	2.77429900	2.72449600	-0.93852800
H	2.84753200	0.76157700	-1.81352300
C	1.79565900	2.86966100	1.26133700
H	1.12114500	1.01122400	2.11213100
C	2.39694400	3.49004000	0.16588200
H	3.24480300	3.19911100	-1.79468600
H	1.50714500	3.45440900	2.12988800
H	2.57442500	4.56140600	0.17434400

(*Z*)-**3ai** single-point energy = -956.23798956 a.u.

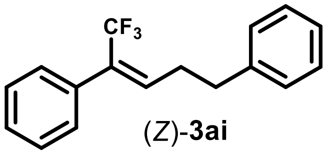

(*Z*)-**3ai**

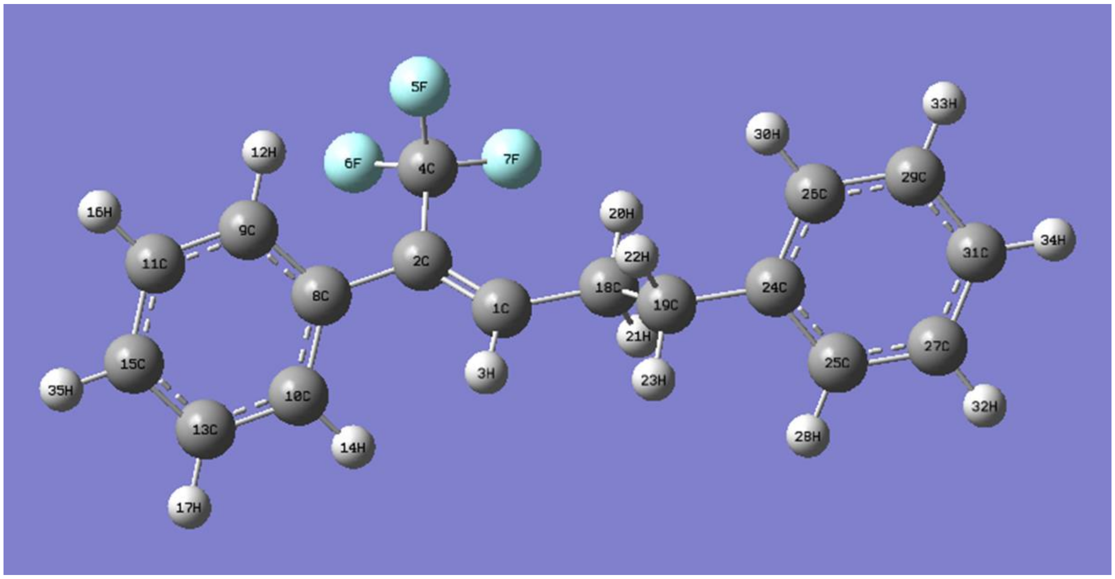

(*Z*)-**3ai**:

0 1

C	-0.33719400	-0.57174400	-0.23144500
C	-1.44406000	0.19311900	-0.23630100
H	-0.51052600	-1.63335600	-0.05382400
C	-1.40874800	1.69486100	-0.41665700
F	-1.39877900	2.33966100	0.78213000
F	-2.49361600	2.13930700	-1.08592000
F	-0.32848300	2.13333900	-1.10092900
C	-2.79322600	-0.40058900	-0.00956500
C	-3.72211400	0.20785700	0.85186400
C	-3.15430300	-1.60177600	-0.63978700
C	-4.96361000	-0.37930300	1.08612300
H	-3.46491200	1.13702100	1.34836700
C	-4.39574200	-2.18972500	-0.40139200
H	-2.46326500	-2.06499700	-1.33757800

C	-5.30540000	-1.58122400	0.46378700
H	-5.66479900	0.10324700	1.76072200
H	-4.65622700	-3.11714200	-0.90316200
C	1.11307900	-0.21211700	-0.40710400
C	2.01635600	-0.99252800	0.57780500
H	1.28559500	0.85784400	-0.29379800
H	1.42031000	-0.46478000	-1.43273700
H	1.71509500	-0.74995000	1.60358100
H	1.84534100	-2.06848400	0.44758100
C	3.48491300	-0.68106000	0.38644200
C	4.26603100	-1.42447600	-0.50829300
C	4.08771900	0.38215200	1.07176000
C	5.61100700	-1.11573600	-0.71396200
H	3.81669500	-2.25704800	-1.04485800
C	5.43219300	0.69496400	0.86948800
H	3.49789000	0.96715800	1.77354200
C	6.19855800	-0.05345300	-0.02531600
H	6.20074700	-1.70751300	-1.40833300
H	5.88190200	1.52057200	1.41373800
H	7.24612600	0.18663800	-0.18161200
H	-6.27482500	-2.03530400	0.64616800

The DFT calculations indicated that (*E*)-**3aa** and (*E*)-**3ai** were more thermodynamically stable than their stereoisomers.

8. Supplementary References

1. Oh K, Fuchibe K, Ichikawa J. *Synthesis* **2011**, 6: 881–886.
2. Wang CQ, Li Y, Feng C. *Cell Rep. Phys. Sci.,* **2021**, 2: 100461.
3. Xing B, Ni C, Hu J. *Angew. Chem. Int. Ed.,* **2018**, 57: 9896–9900.
4. Radu II, Poirier D, Provencher L. *Tetrahedron Lett.,* **2002**, 43: 7617–7619.
5. Asomoza-Solís EO, Rojas-Ocampo J, Toscano RA, Porcel S. *Chem. Commun.,* **2016**, 52: 7295–7298.
6. Chen L, Luo ZF, Ye P, Mao YJ, Xu ZY, Xu DQ, Lou SJ. *Org. Biomol. Chem.,* **2023**, 21: 8979–8983.
7. Frisch MJ, Trucks G.W, Schlegel HB, et al., Gaussian 09, Revision D. 01, Gaussian, Inc, Wallingford, CT, 2013.
8. (a) Becke AD, *J. Chem. Phys.,* **1993**, 98: 5648–5652; (b) Lee C, Yang W, Parr RG, *Phys. Rev. B., 1988*, 37: 785–789; (c) Peverati R, Truhlar DG, *J. Phys. Chem. Lett.,* **2011**, 2: 2810–2817.
9. Zhao Y, Truhlar DG, *Theor. Chem. Acc.,* **2008**, 120: 215–241.

9. NMR Spectra

^{1}H NMR Spectrum of **1a** ($CDCl_3$, 400 MHz)

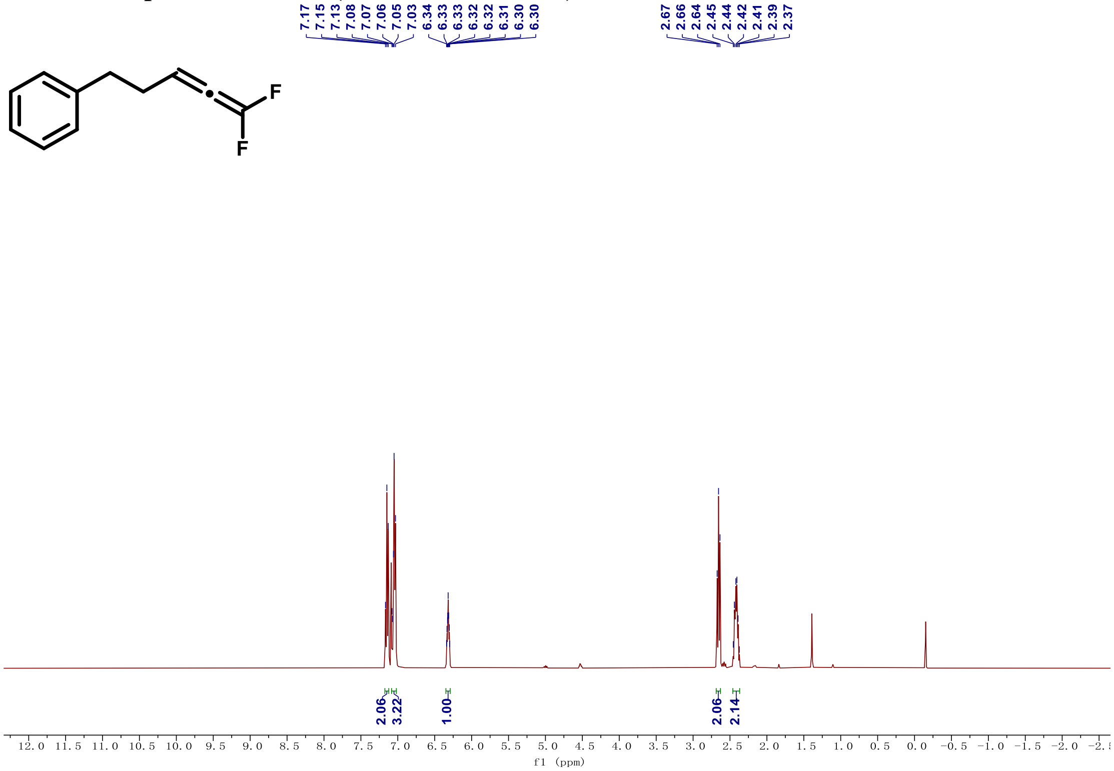

^{19}F NMR Spectrum of **1a** ($CDCl_3$, 376 MHz)

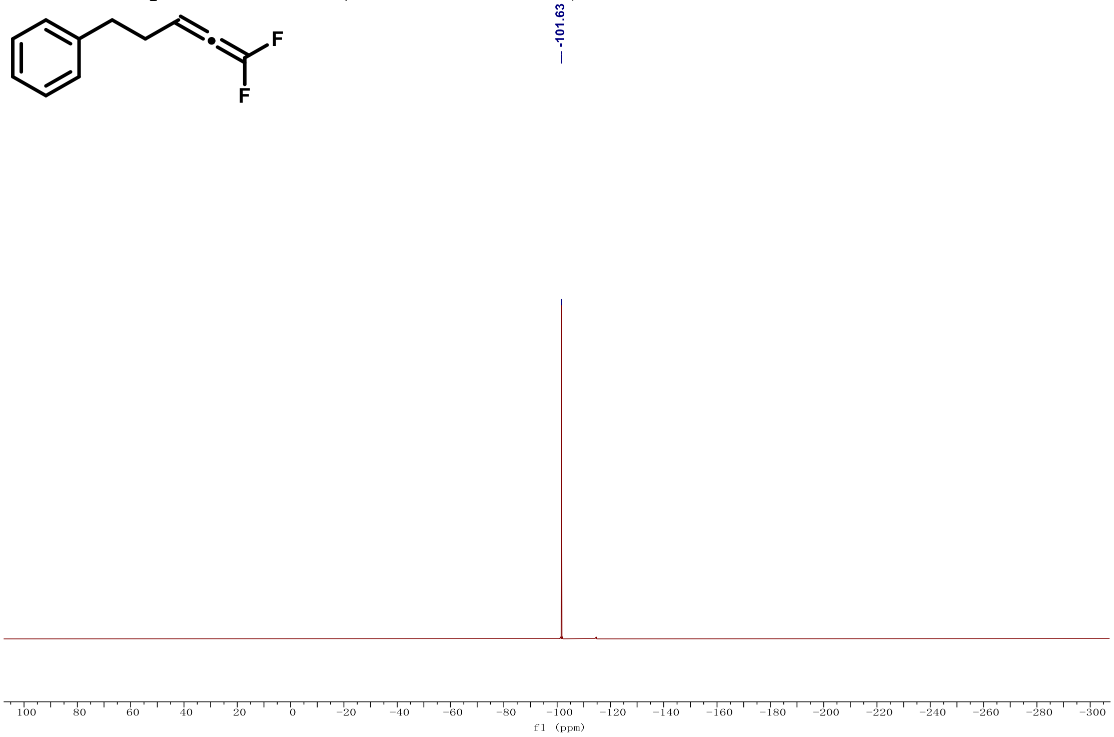

^{13}C NMR Spectrum of **1a** ($CDCl_3$, 101 MHz)

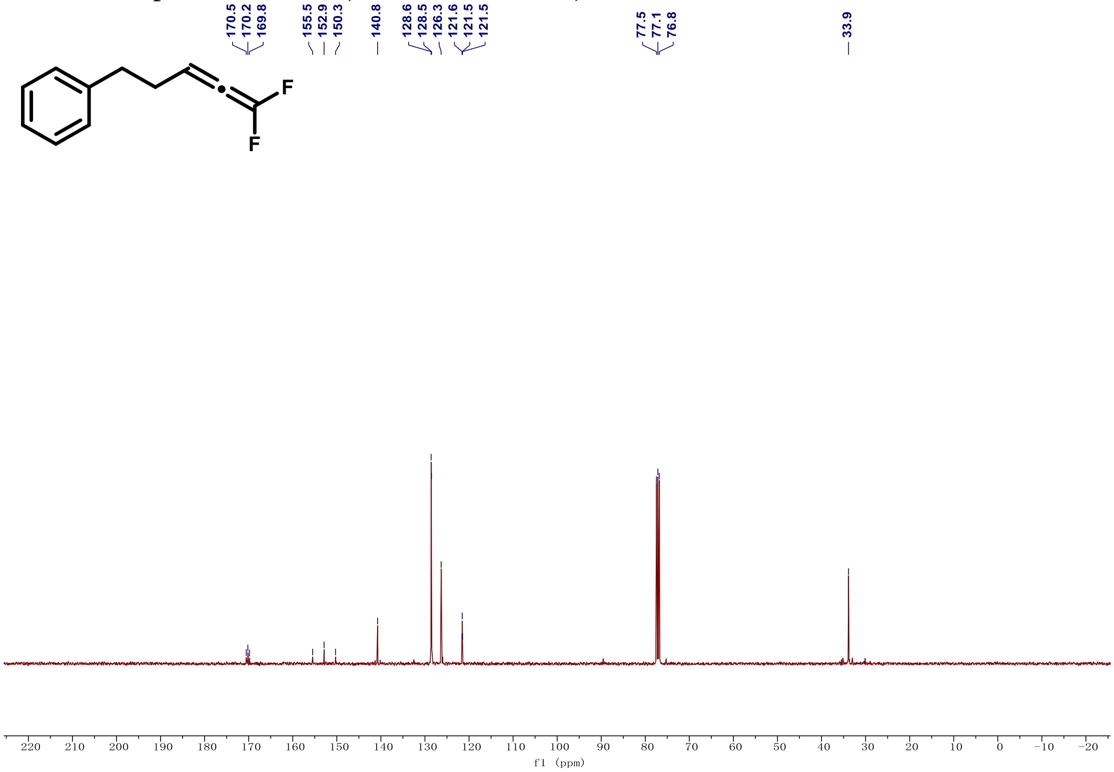

^{1}H NMR Spectrum of **1b** ($CDCl_3$, 400 MHz)

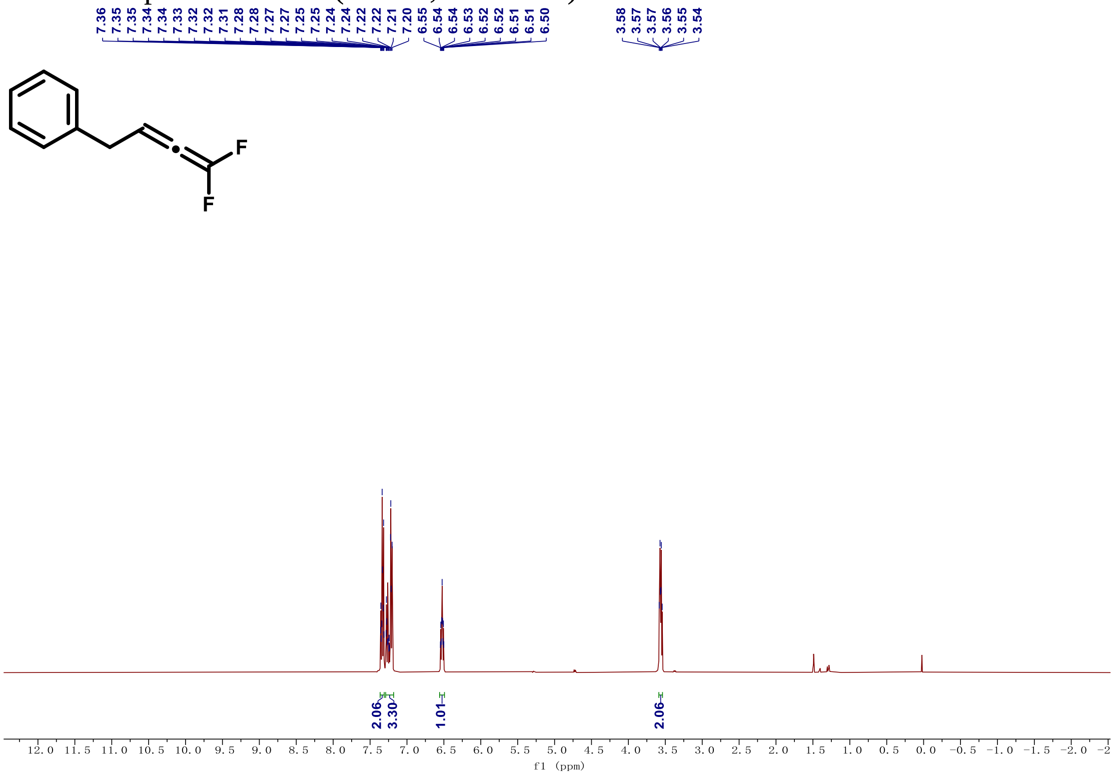

^{19}F NMR Spectrum of **1b** ($CDCl_3$, 376 MHz)

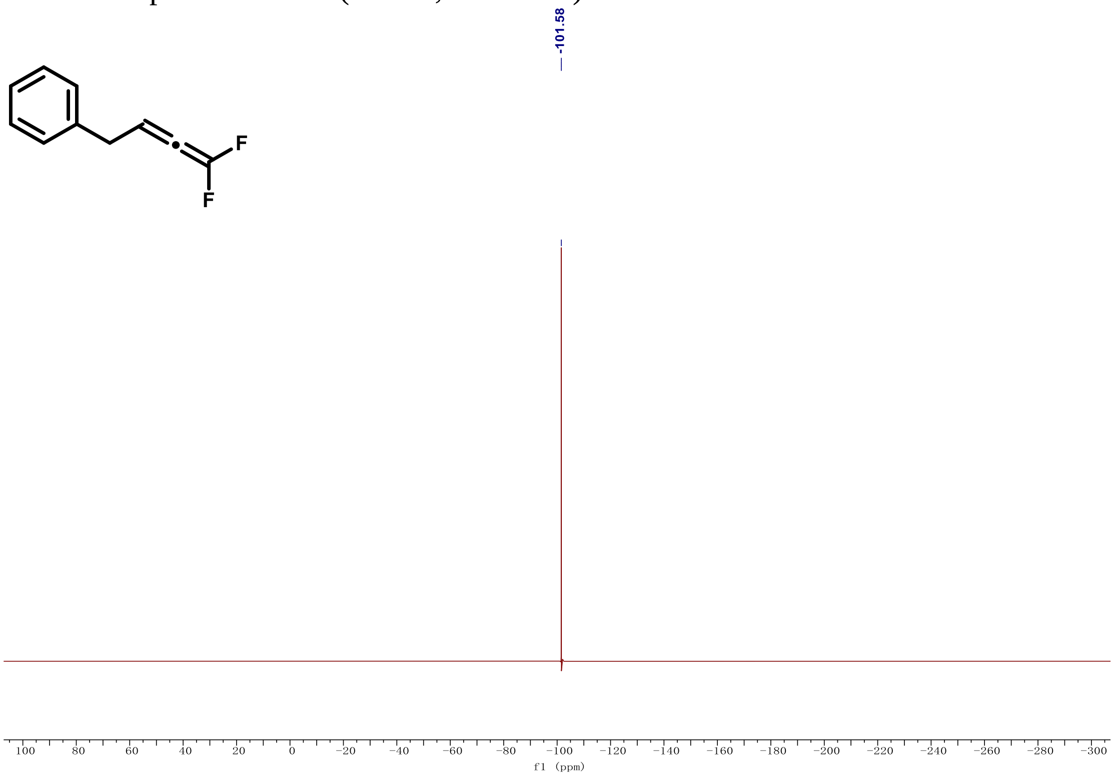

^{13}C NMR Spectrum of **1b** ($CDCl_3$, 101 MHz)

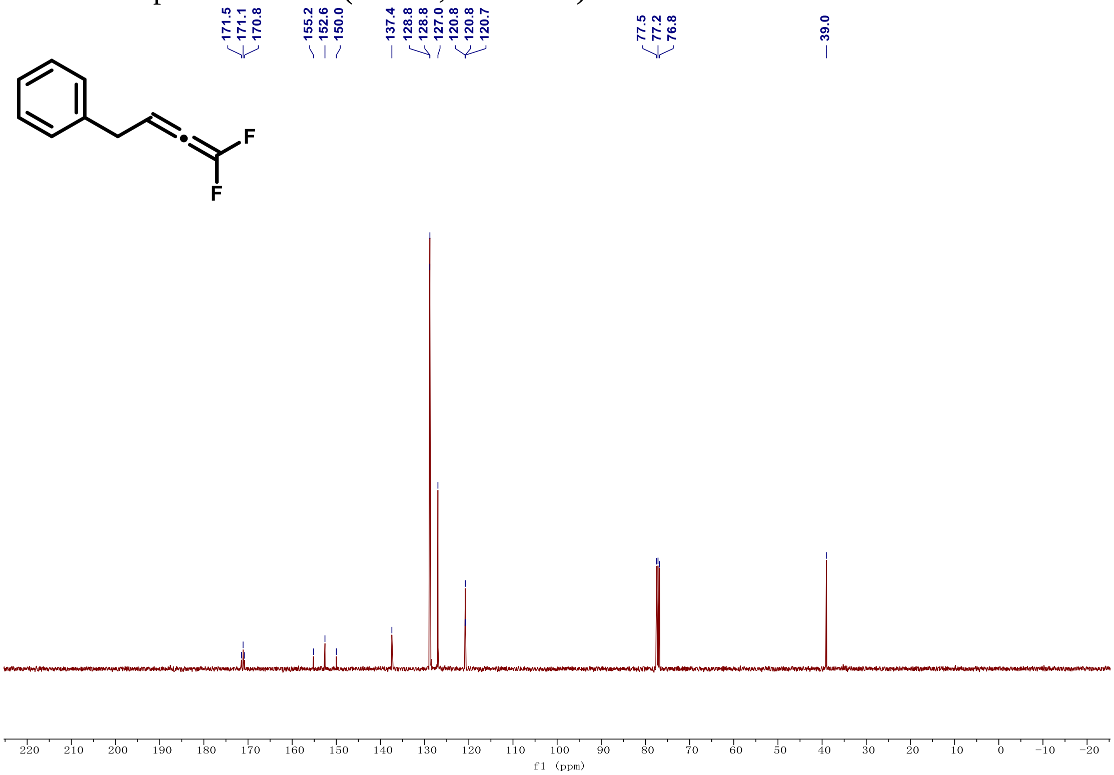

^{1}H NMR Spectrum of **1c** ($CDCl_3$, 400 MHz)

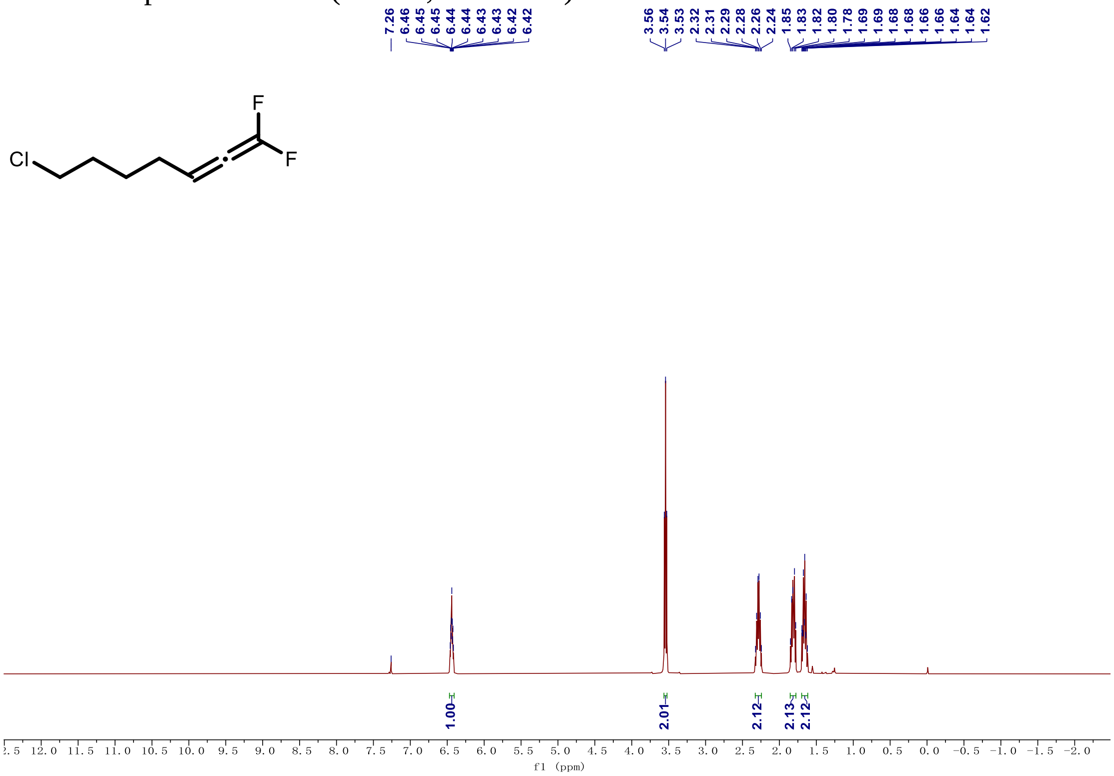

^{19}F NMR Spectrum of **1c** ($CDCl_3$, 376 MHz)

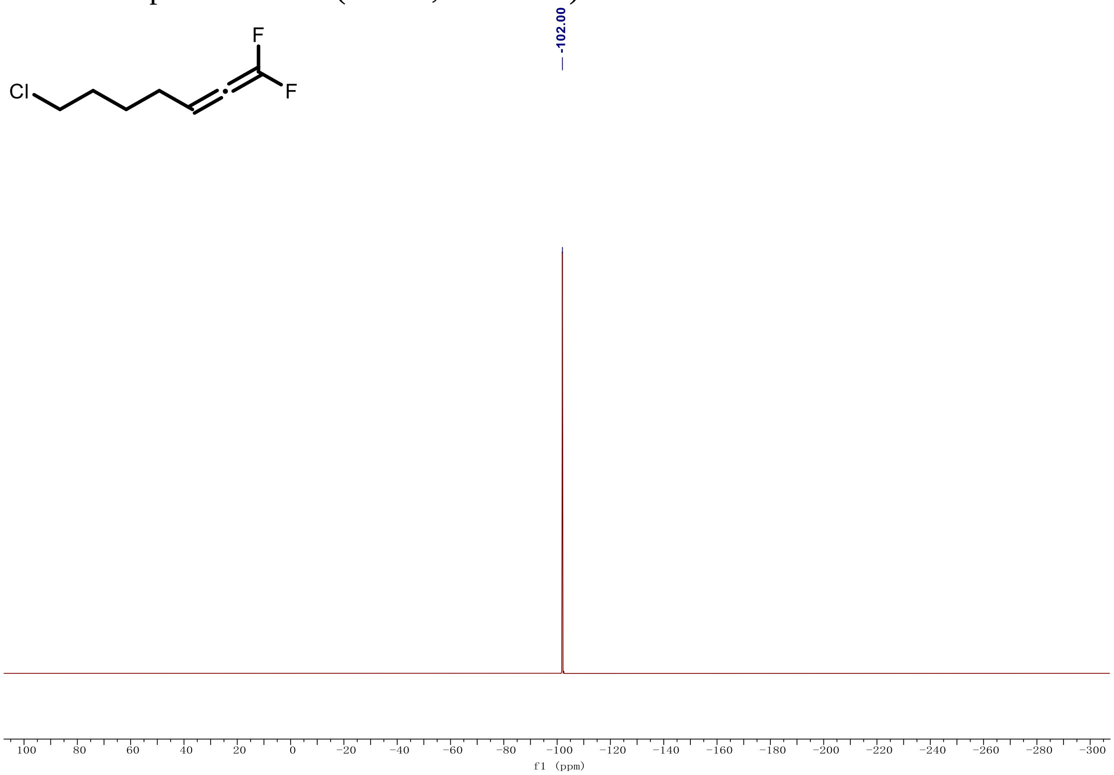

^{13}C NMR Spectrum of **1c** ($CDCl_3$, 101 MHz)

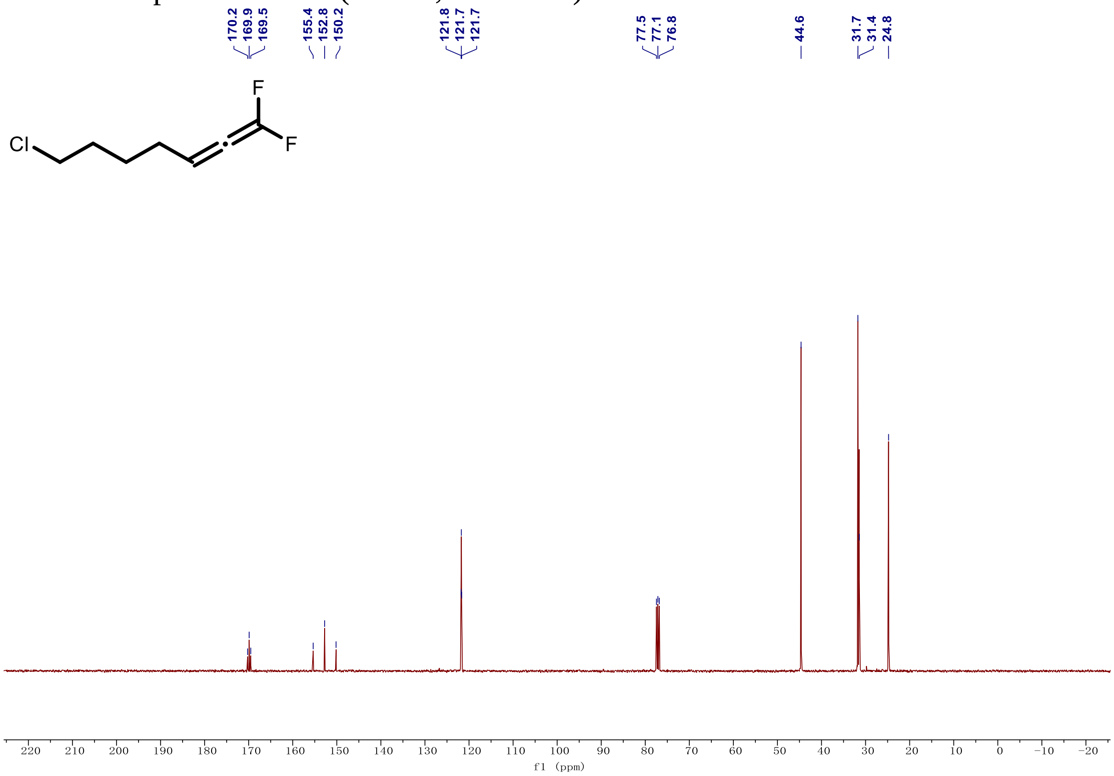

^{1}H NMR Spectrum of **1d** ($CDCl_3$, 400 MHz)

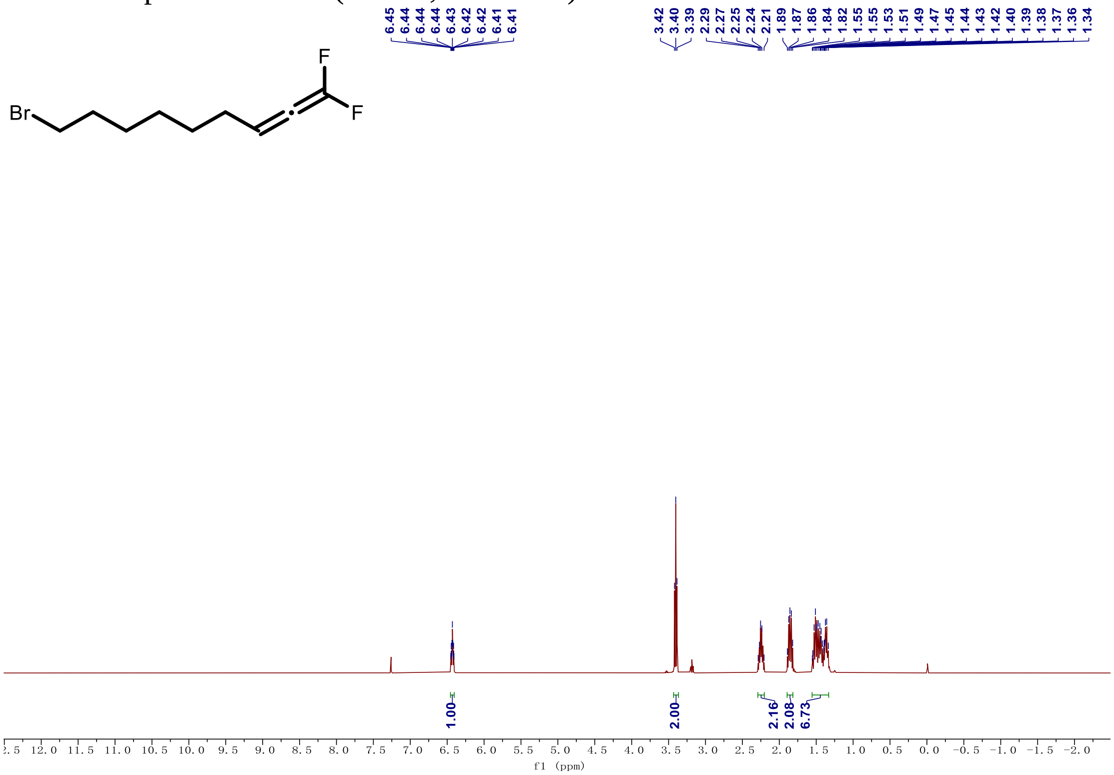

^{19}F NMR Spectrum of **1d** (CDCl$_3$, 376 MHz)

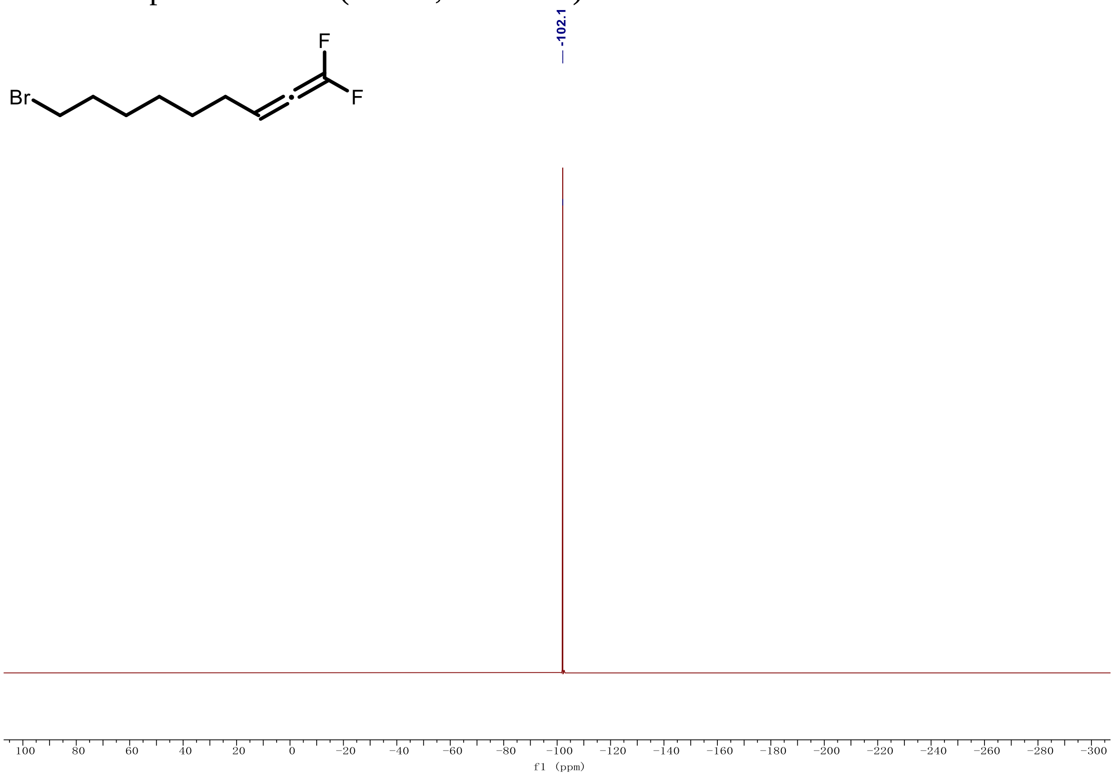

^{13}C NMR Spectrum of **1d** (CDCl$_3$, 101 MHz)

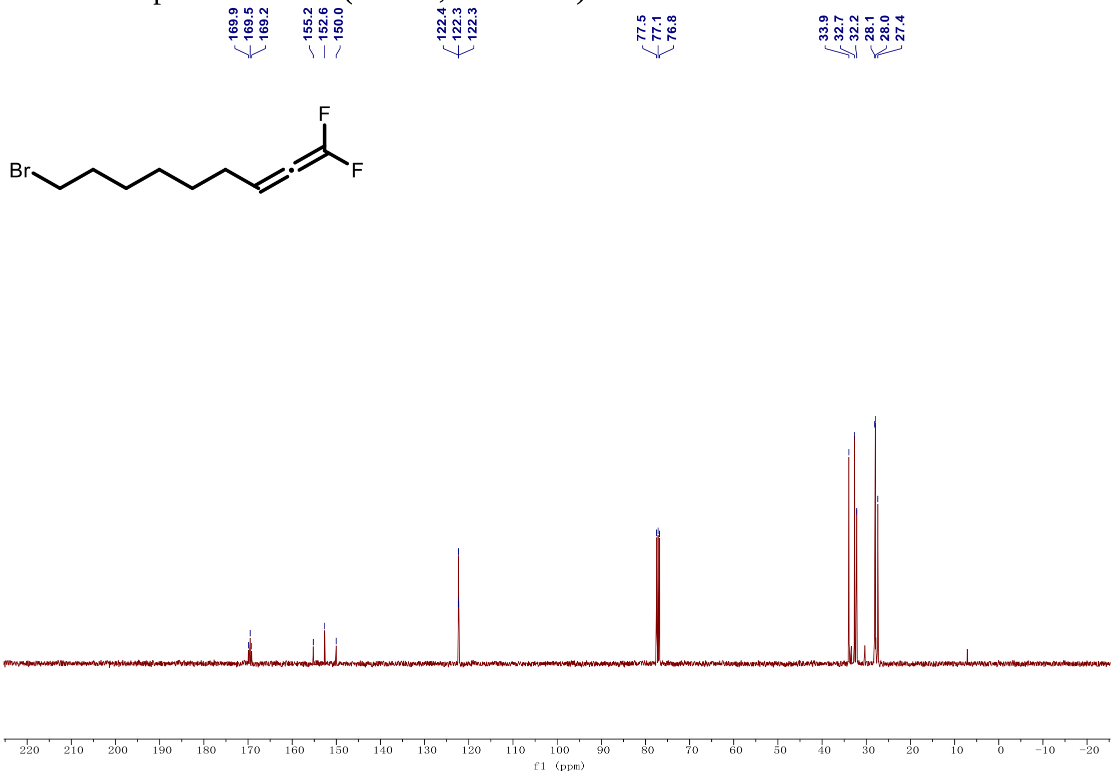

^{1}H NMR Spectrum of **1e** ($CDCl_3$, 400 MHz)

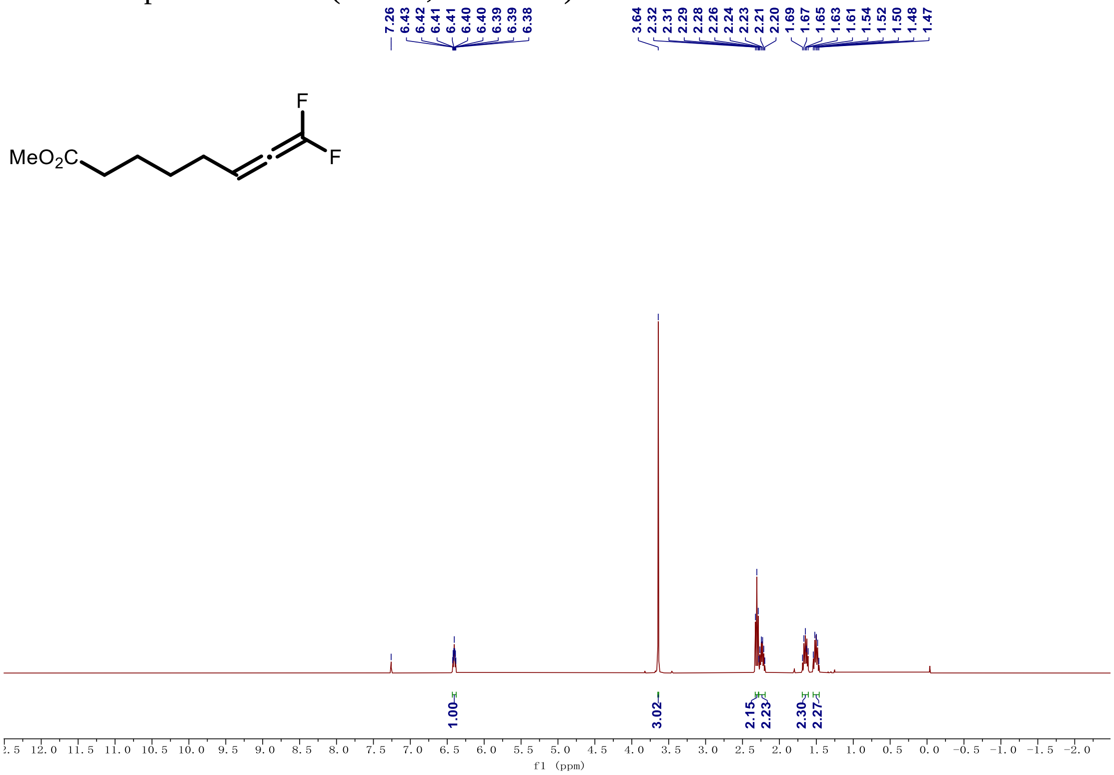

^{19}F NMR Spectrum of **1e** ($CDCl_3$, 376 MHz)

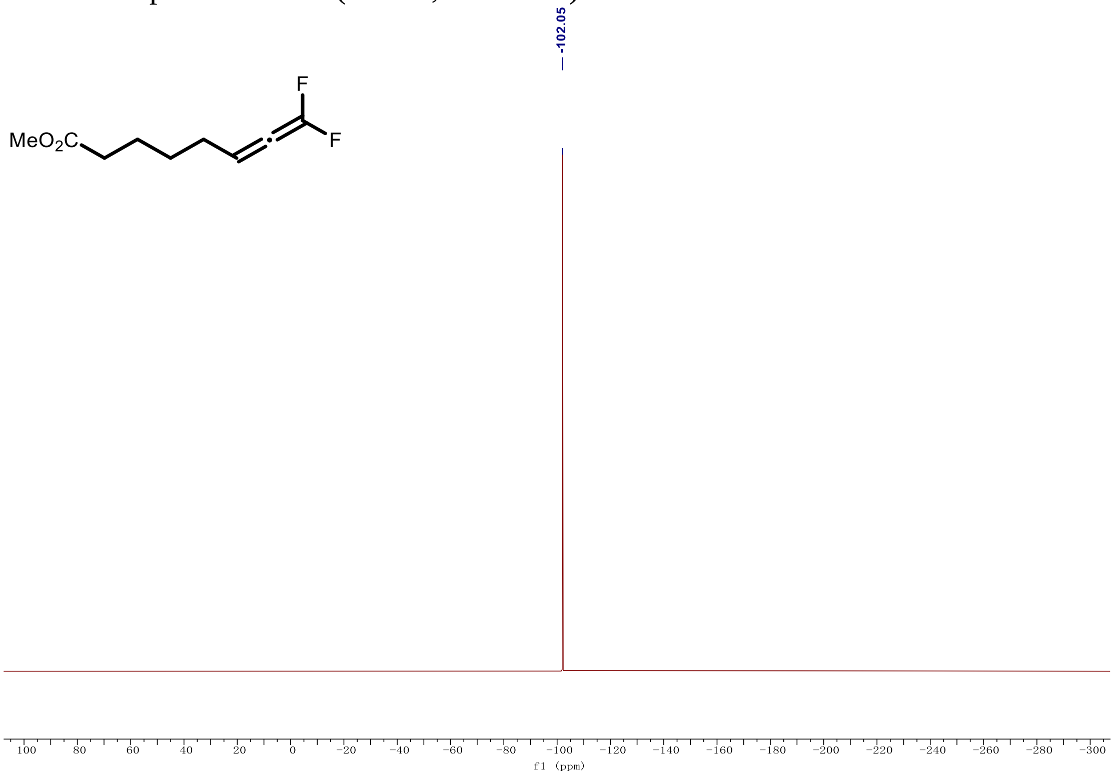

^{13}C NMR Spectrum of **1e** ($CDCl_3$, 101 MHz)

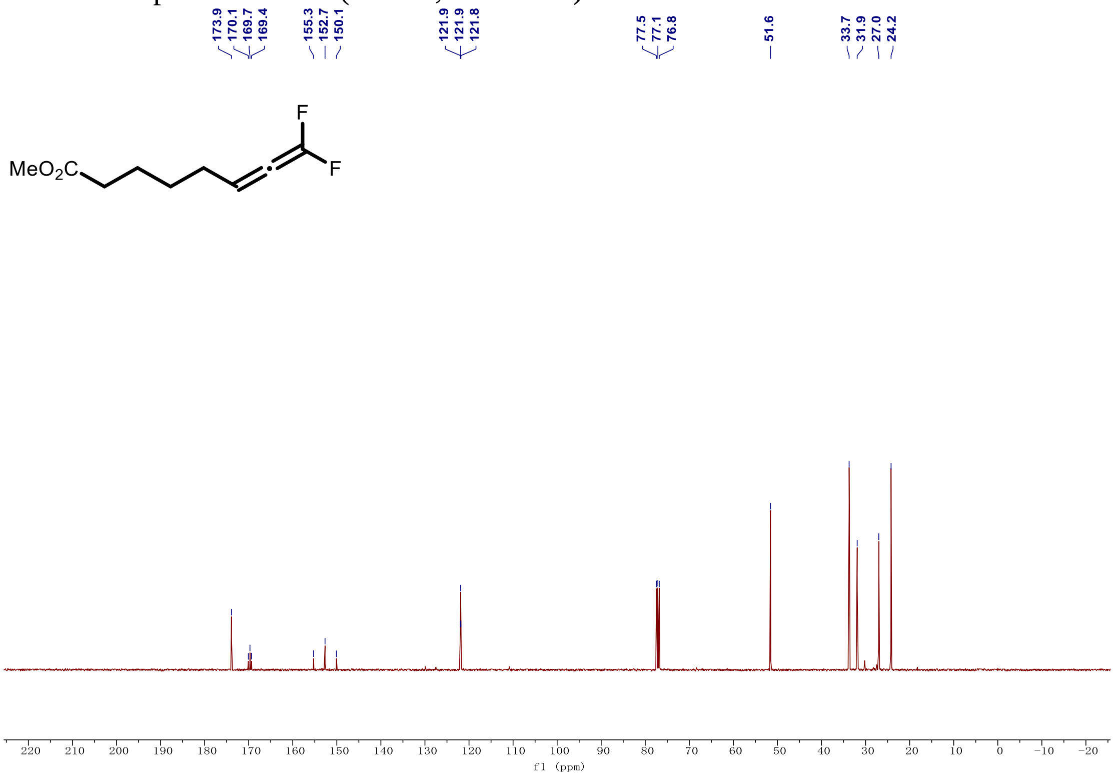

^{1}H NMR Spectrum of **1g** ($CDCl_3$, 400 MHz)

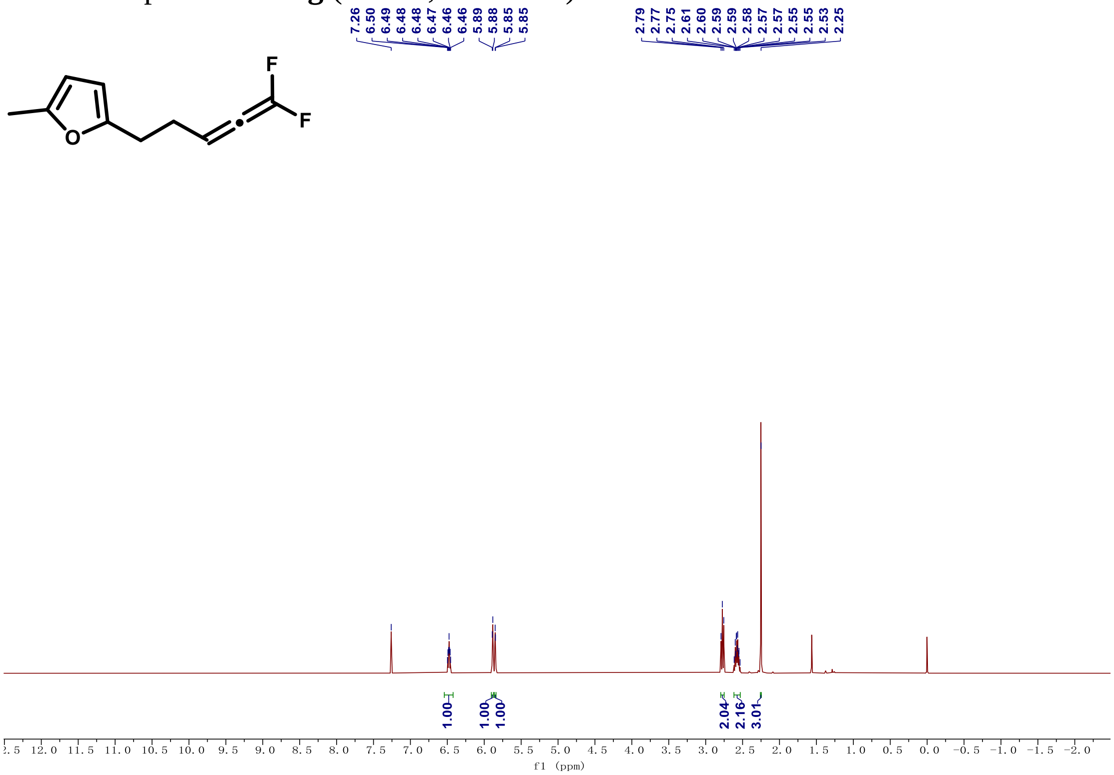

^{19}F NMR Spectrum of **1g** ($CDCl_3$, 376 MHz)

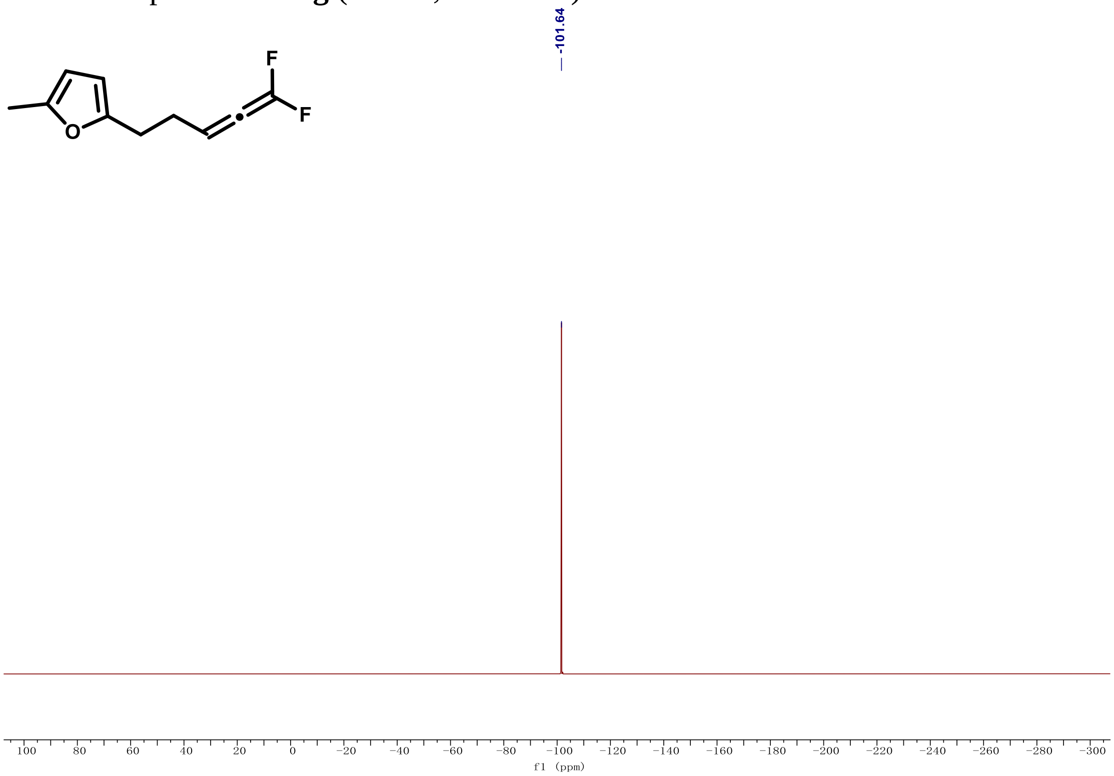

^{13}C NMR Spectrum of **1g** ($CDCl_3$, 101 MHz)

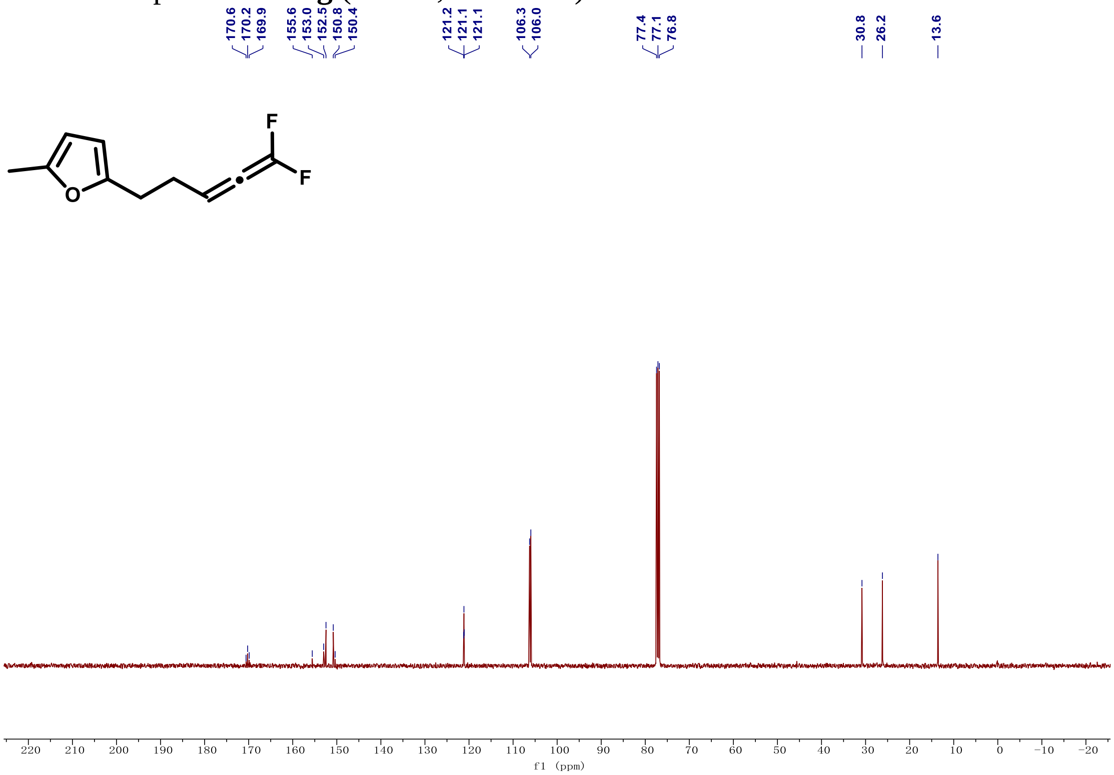

^{1}H NMR Spectrum of **1h** ($CDCl_3$, 400 MHz)

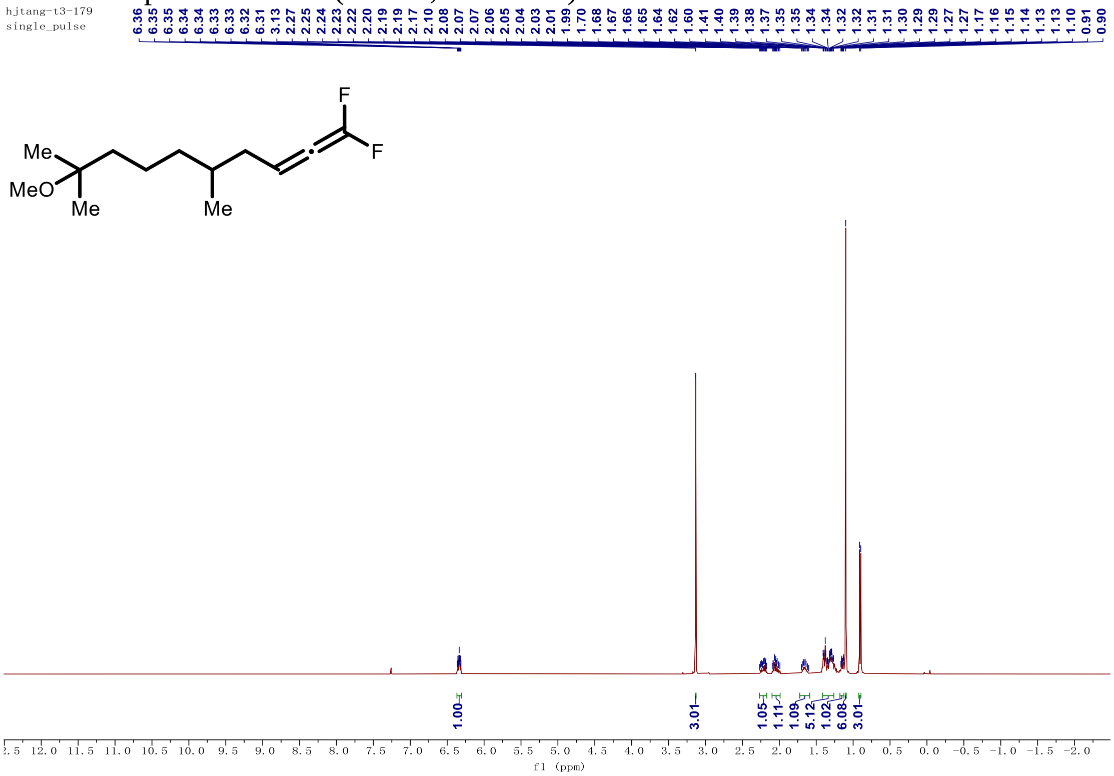

^{19}F NMR Spectrum of **1h** ($CDCl_3$, 376 MHz)

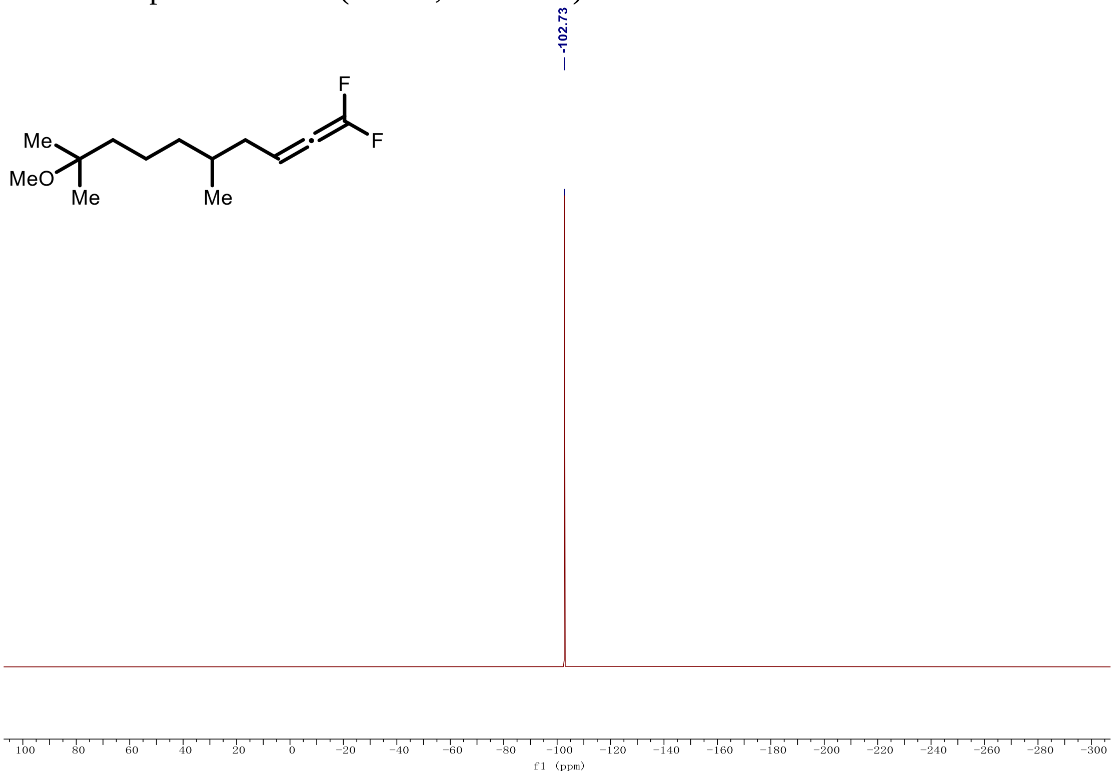

^{13}C NMR Spectrum of **1h** ($CDCl_3$, 101 MHz)

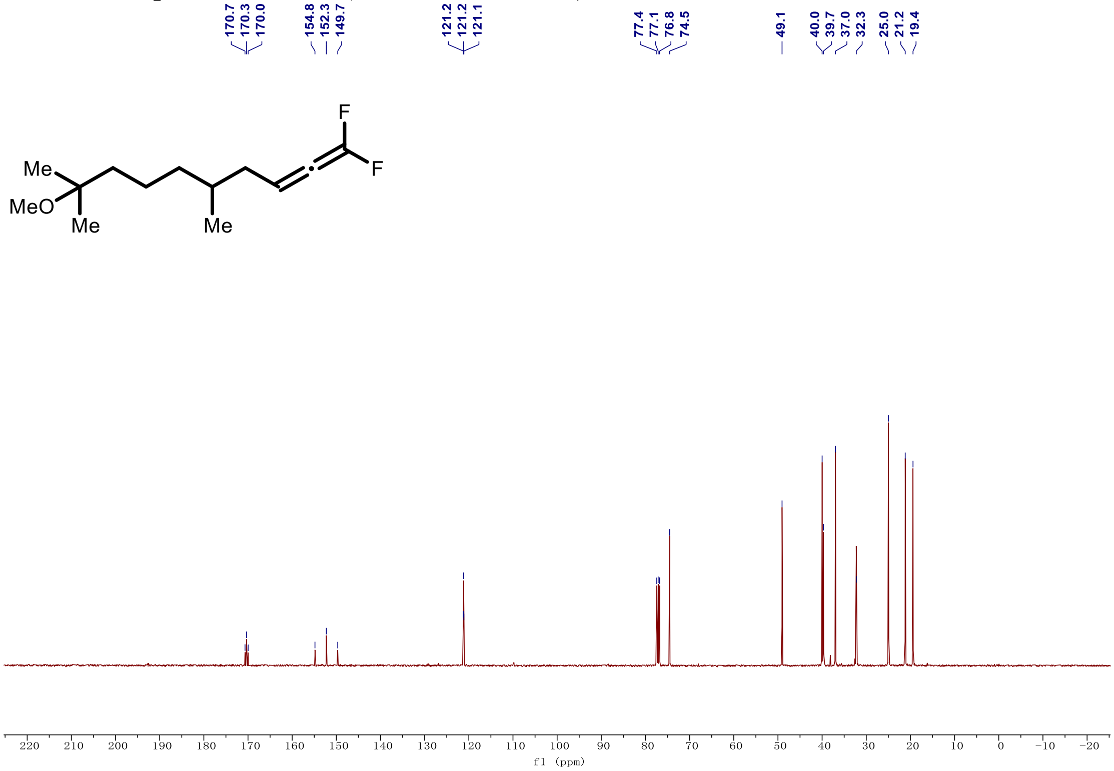

^{1}H NMR Spectrum of **1i** ($CDCl_3$, 400 MHz)

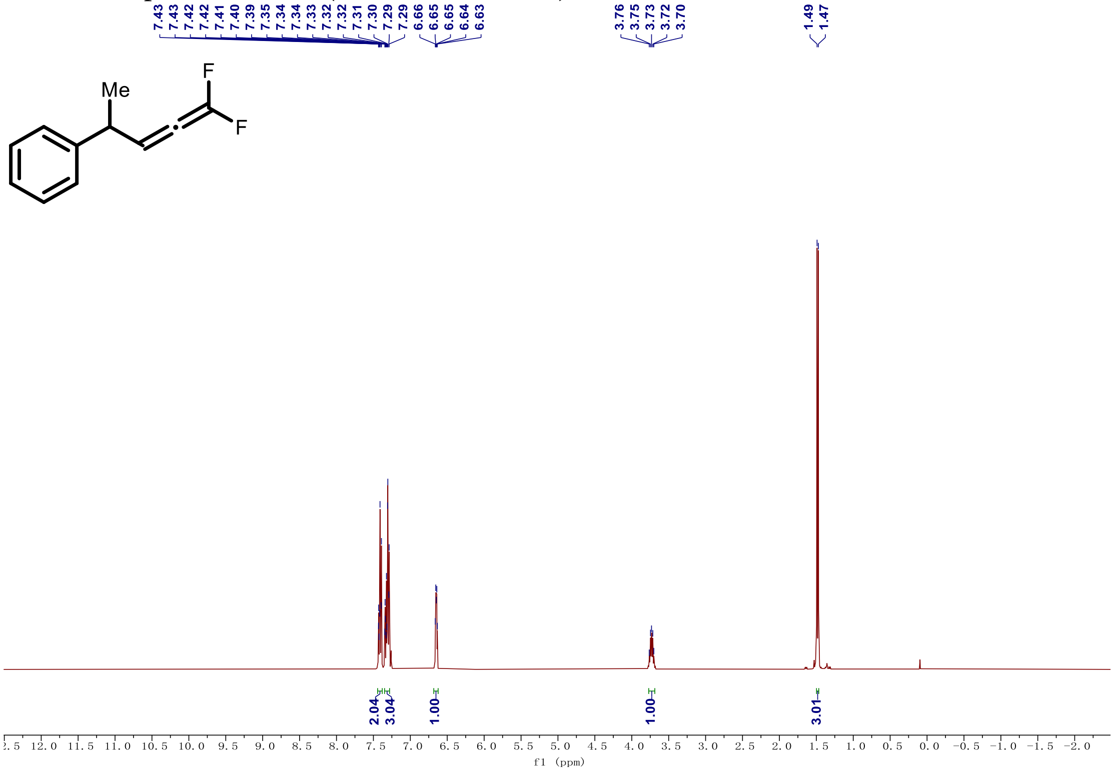

^{19}F NMR Spectrum of **1i** ($CDCl_3$, 376 MHz)

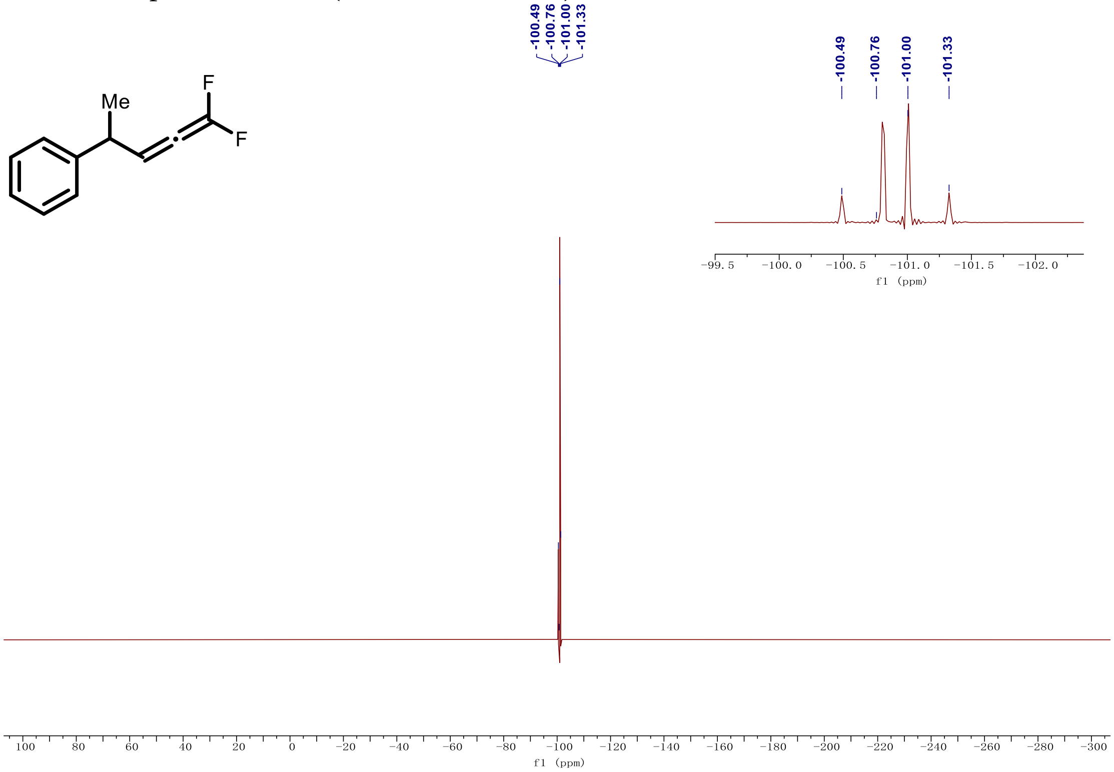

^{13}C NMR Spectrum of **1i** ($CDCl_3$, 101 MHz)

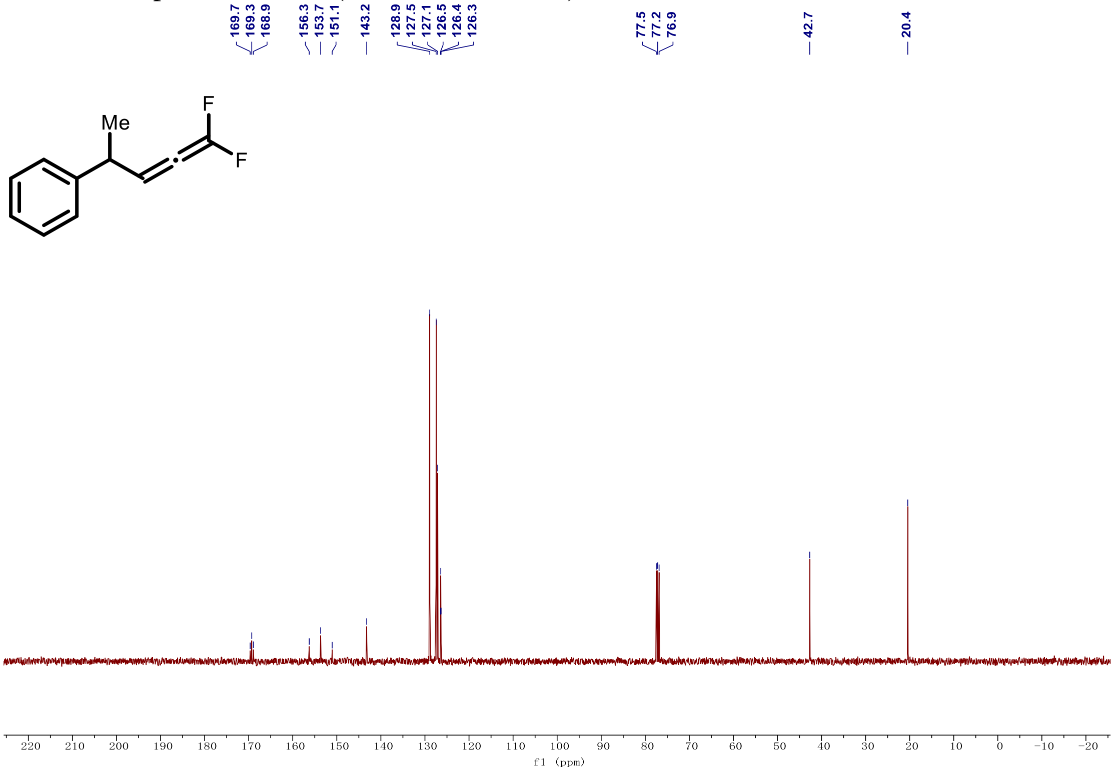

^{1}H NMR Spectrum of **1j** ($CDCl_3$, 400 MHz)

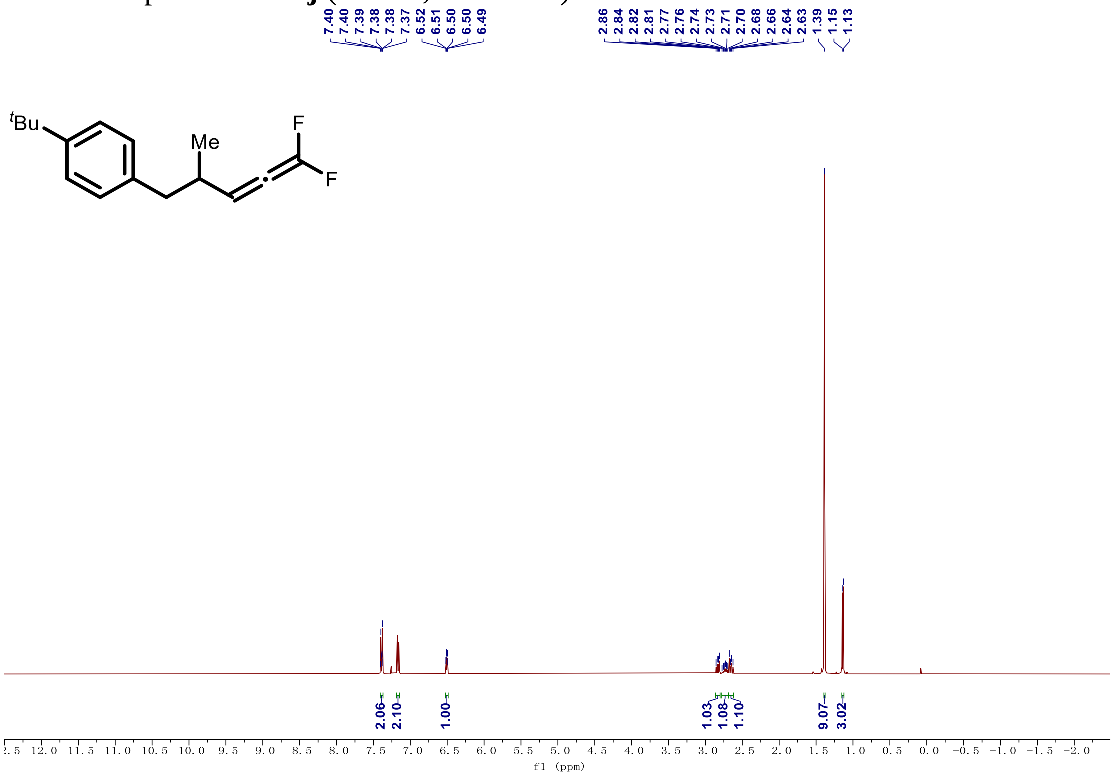

^{19}F NMR Spectrum of **1j** ($CDCl_3$, 376 MHz)

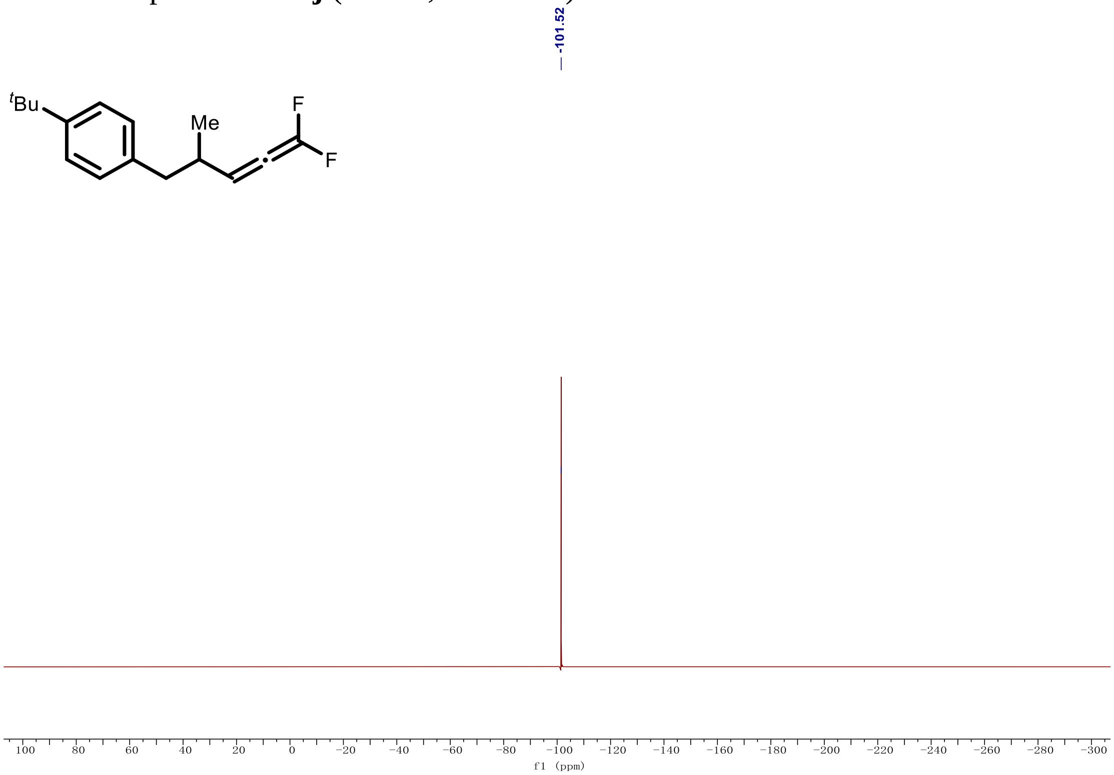

^{13}C NMR Spectrum of **1j** ($CDCl_3$, 101 MHz)

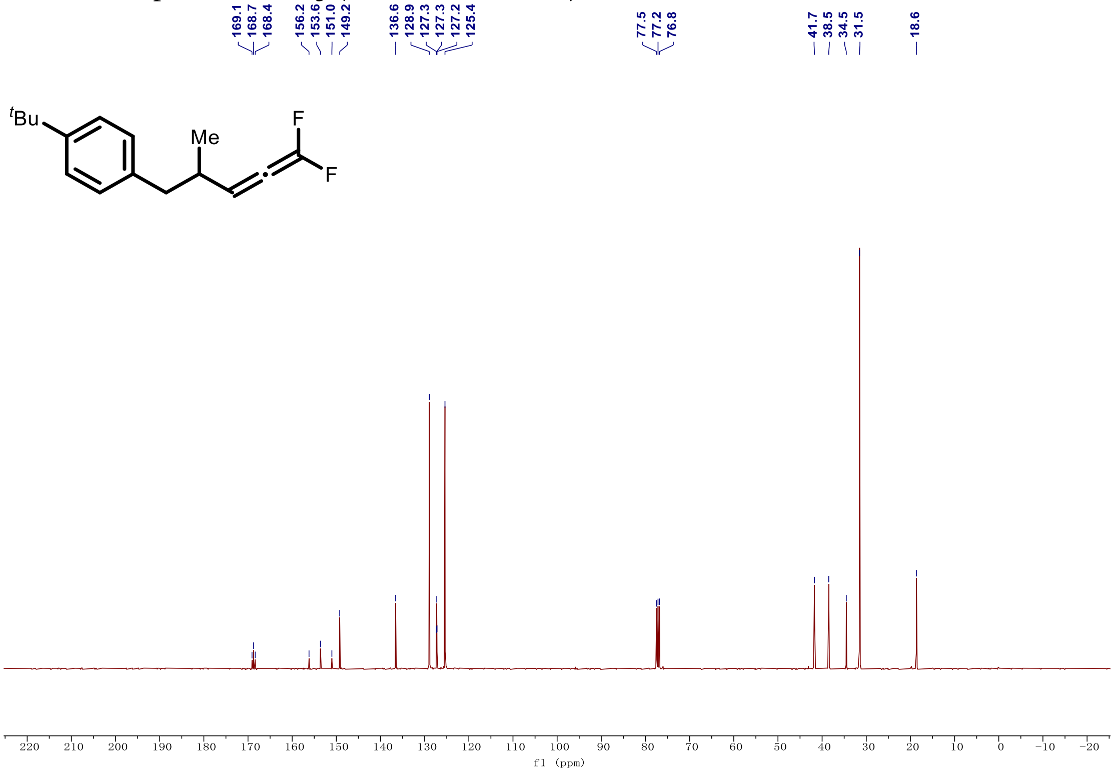

^{1}H NMR Spectrum of **1k** ($CDCl_3$, 400 MHz)

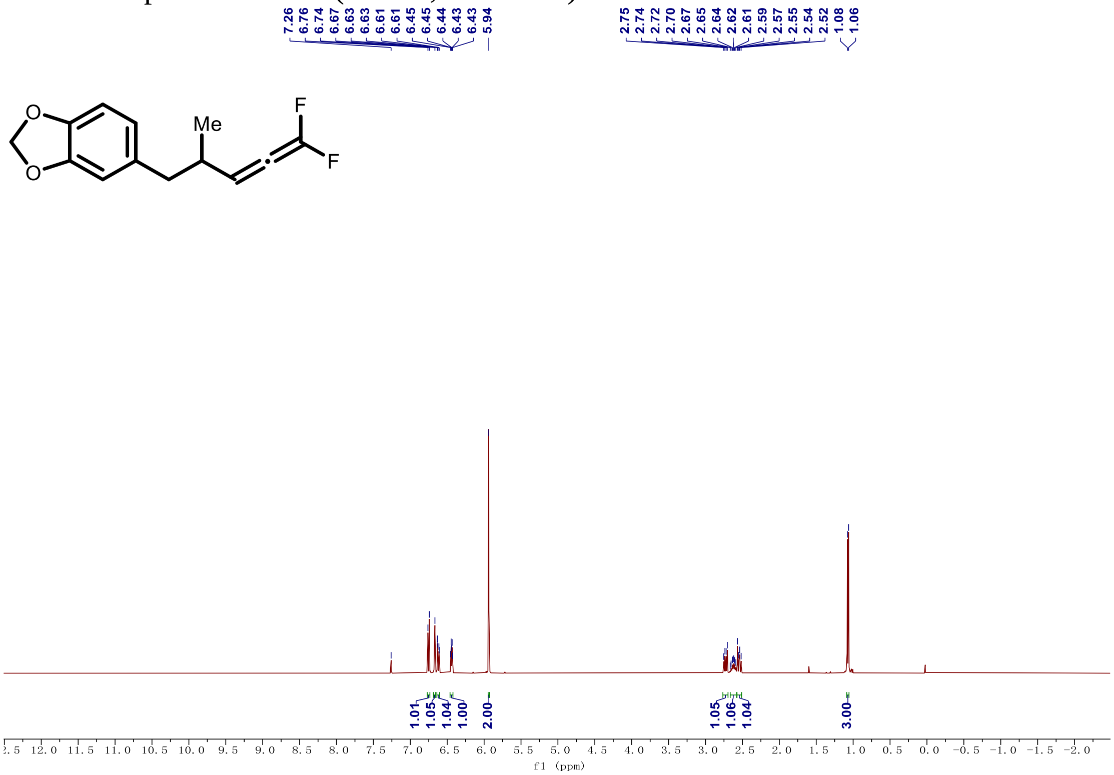

^{19}F NMR Spectrum of **1k** ($CDCl_3$, 376 MHz)

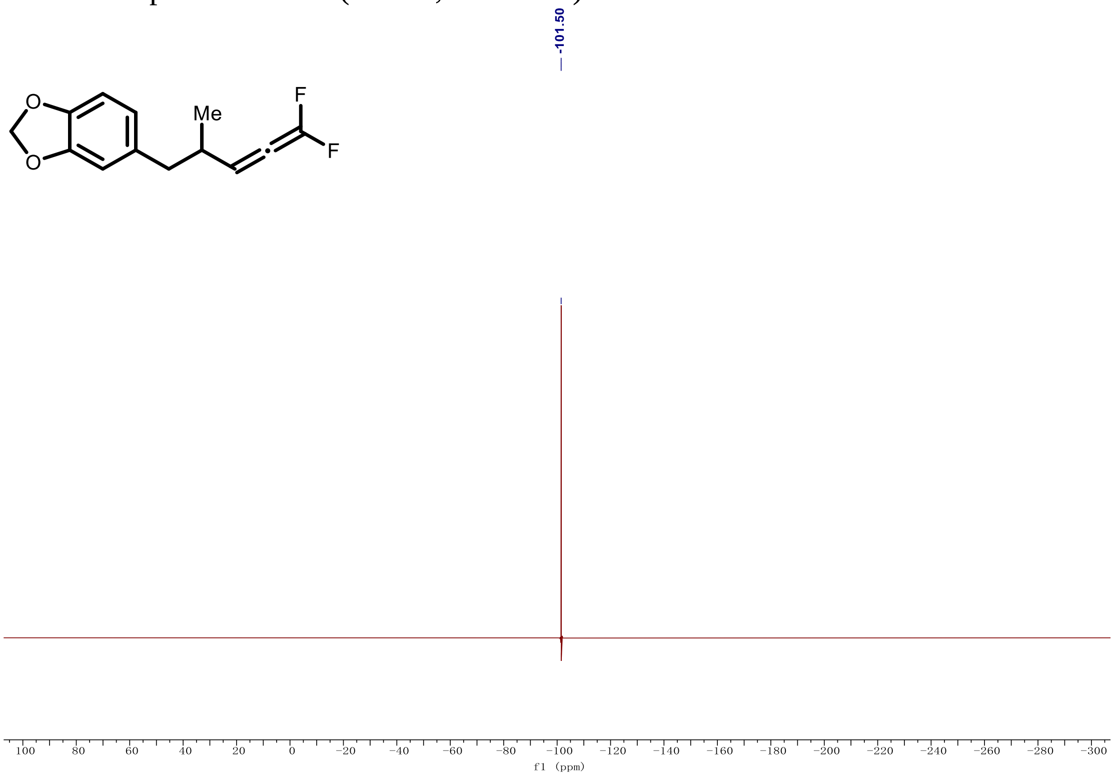

^{13}C NMR Spectrum of **1k** ($CDCl_3$, 101 MHz)

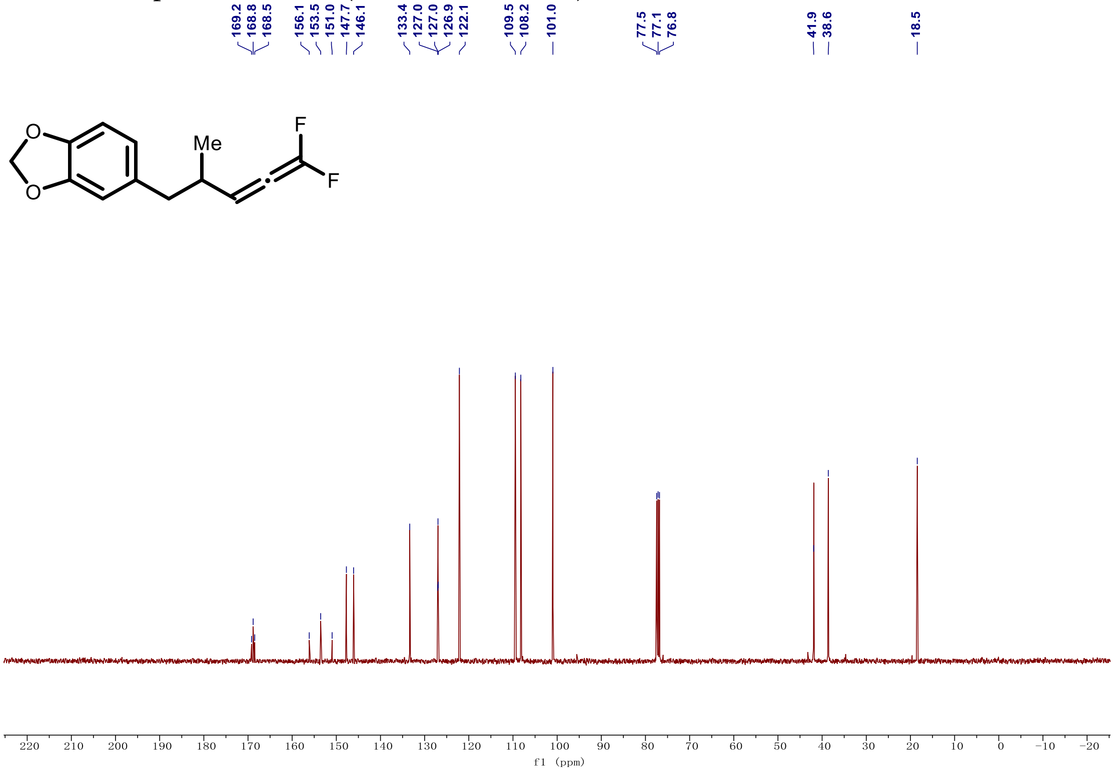

^{1}H NMR Spectrum of **1l** ($CDCl_3$, 400 MHz)

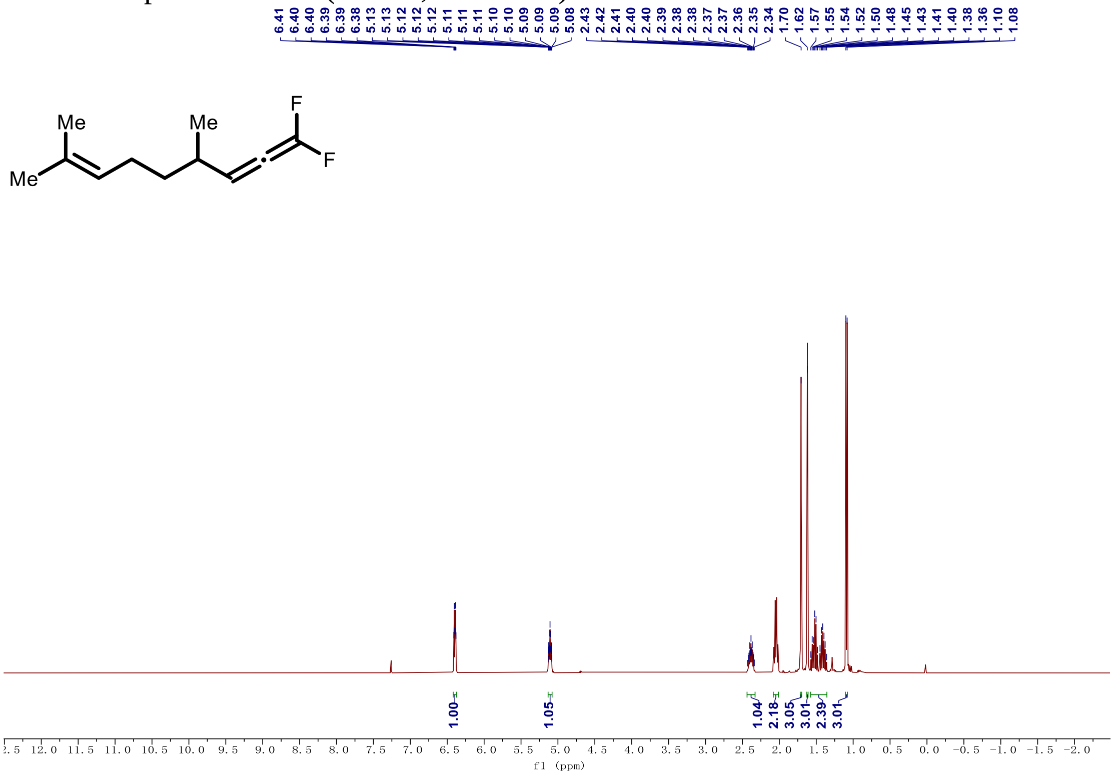

^{19}F NMR Spectrum of **1l** ($CDCl_3$, 376 MHz)

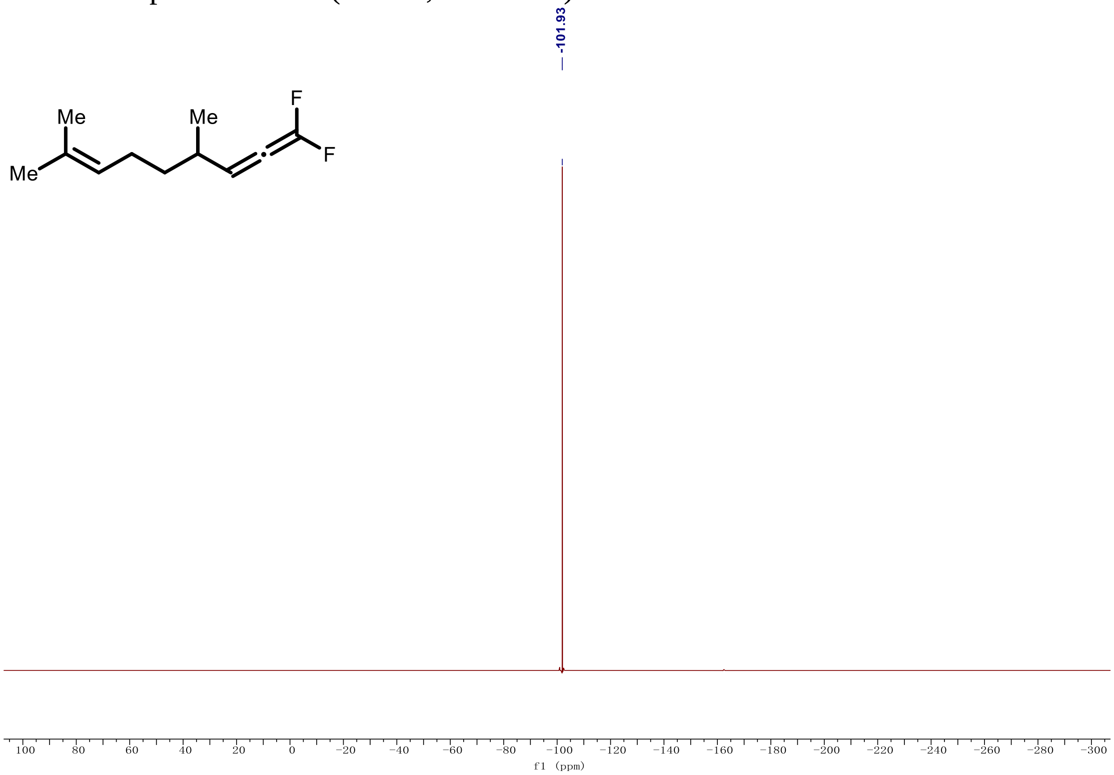

^{13}C NMR Spectrum of **1l** ($CDCl_3$, 101 MHz)

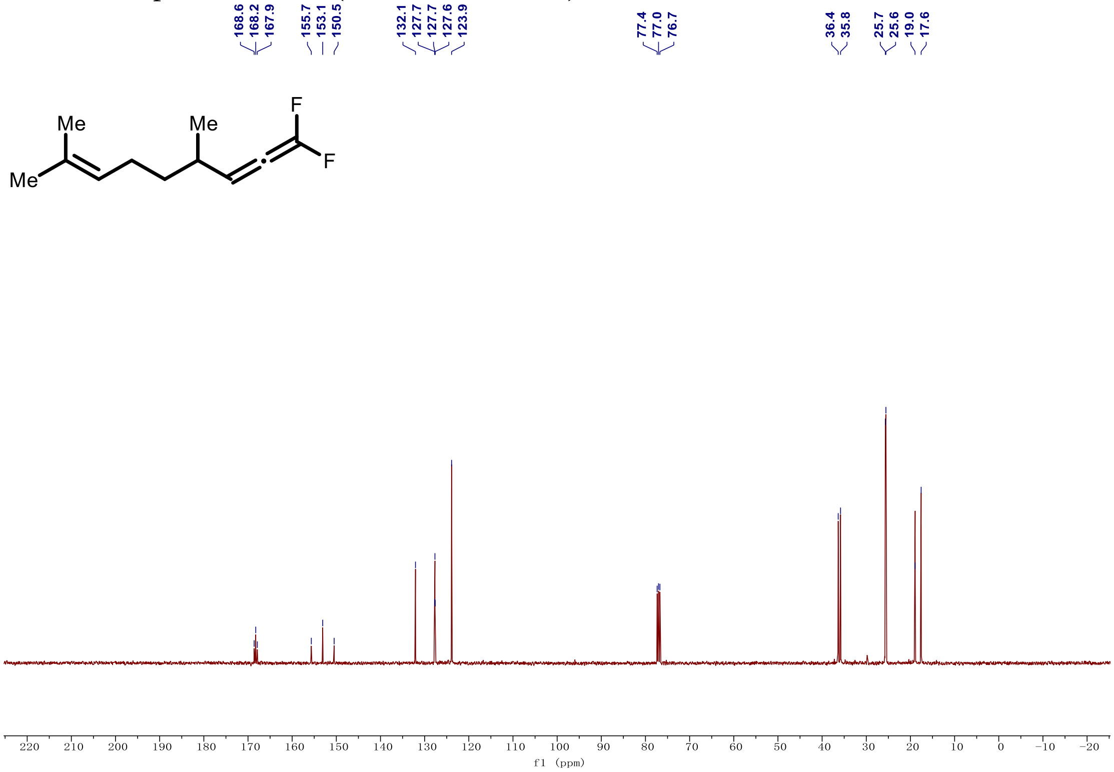

^{1}H NMR Spectrum of **1m** ($CDCl_3$, 400 MHz)

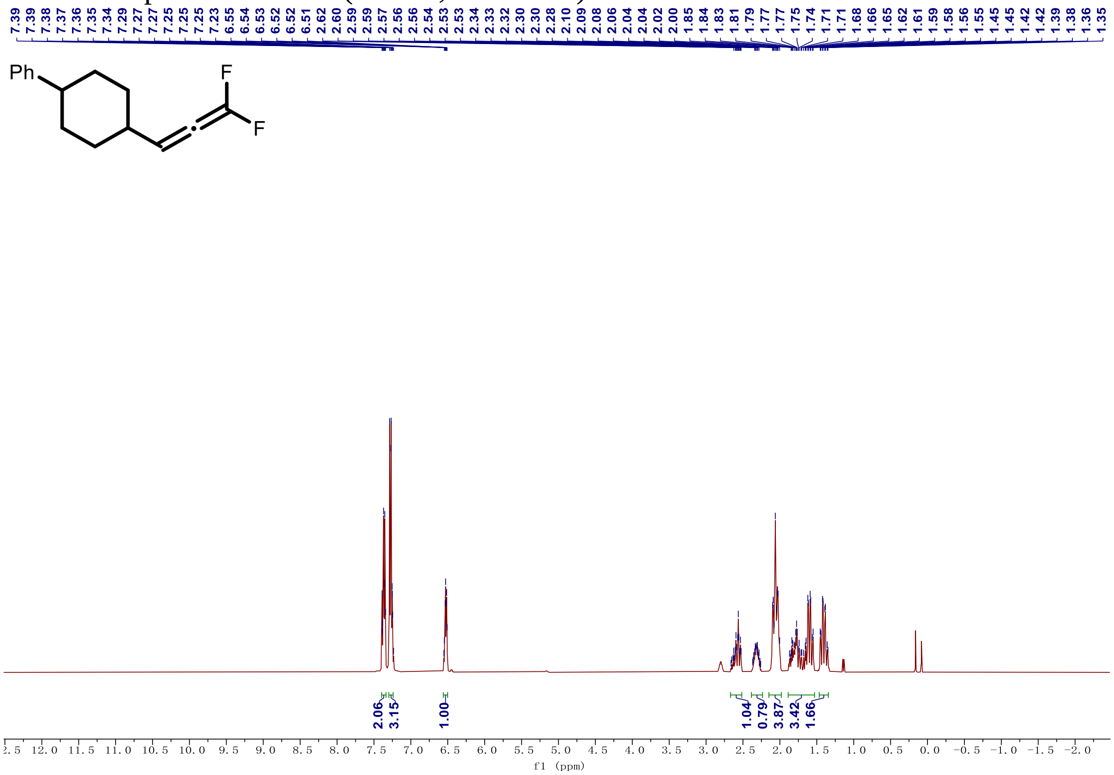

^{19}F NMR Spectrum of **1m** ($CDCl_3$, 376 MHz)

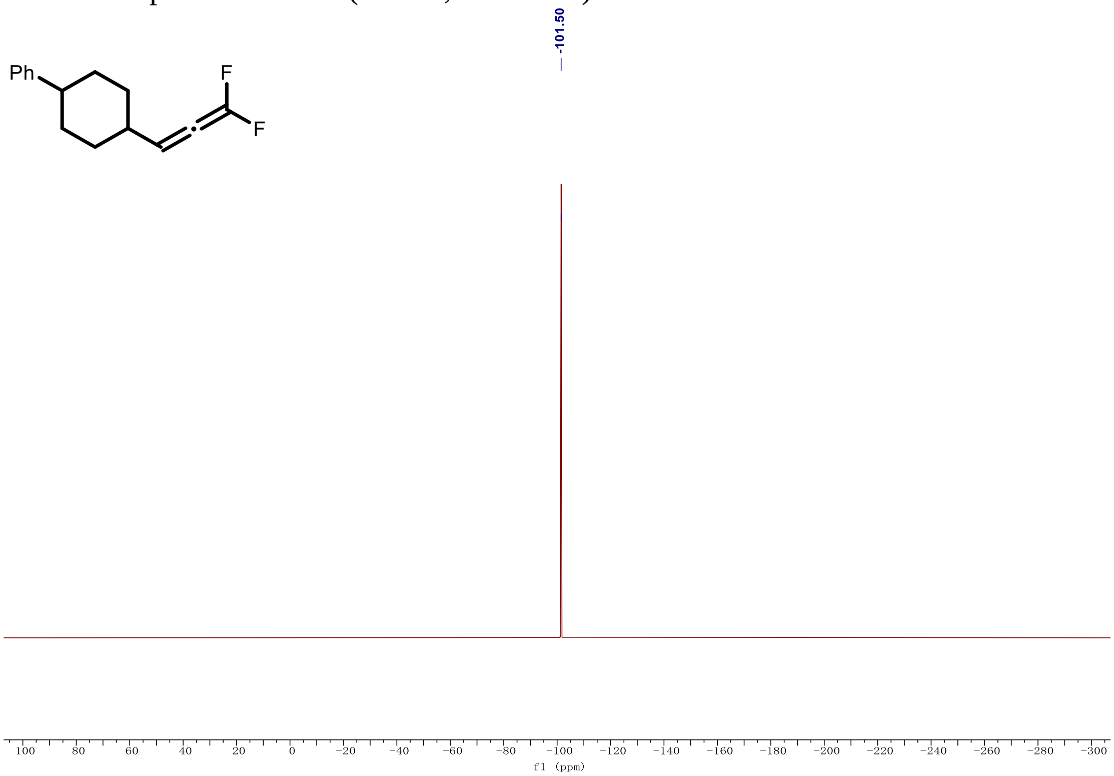

^{13}C NMR Spectrum of **1m** ($CDCl_3$, 101 MHz)

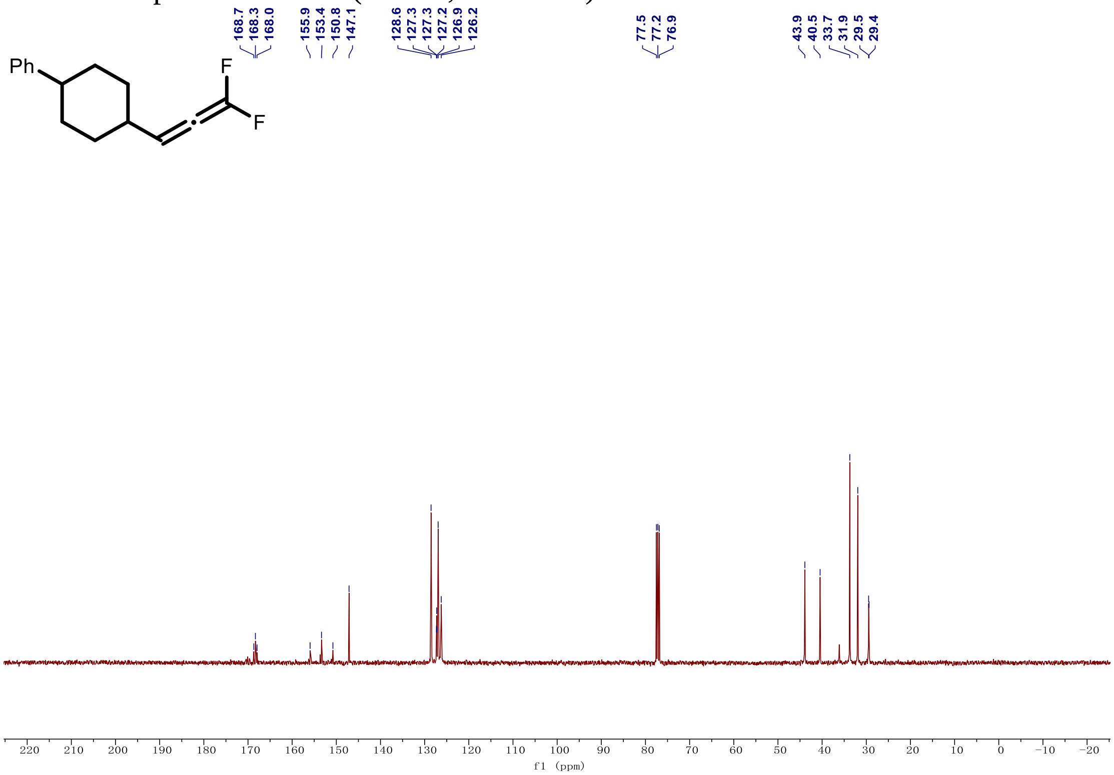

^{1}H NMR Spectrum of **1n** ($CDCl_3$, 400 MHz)

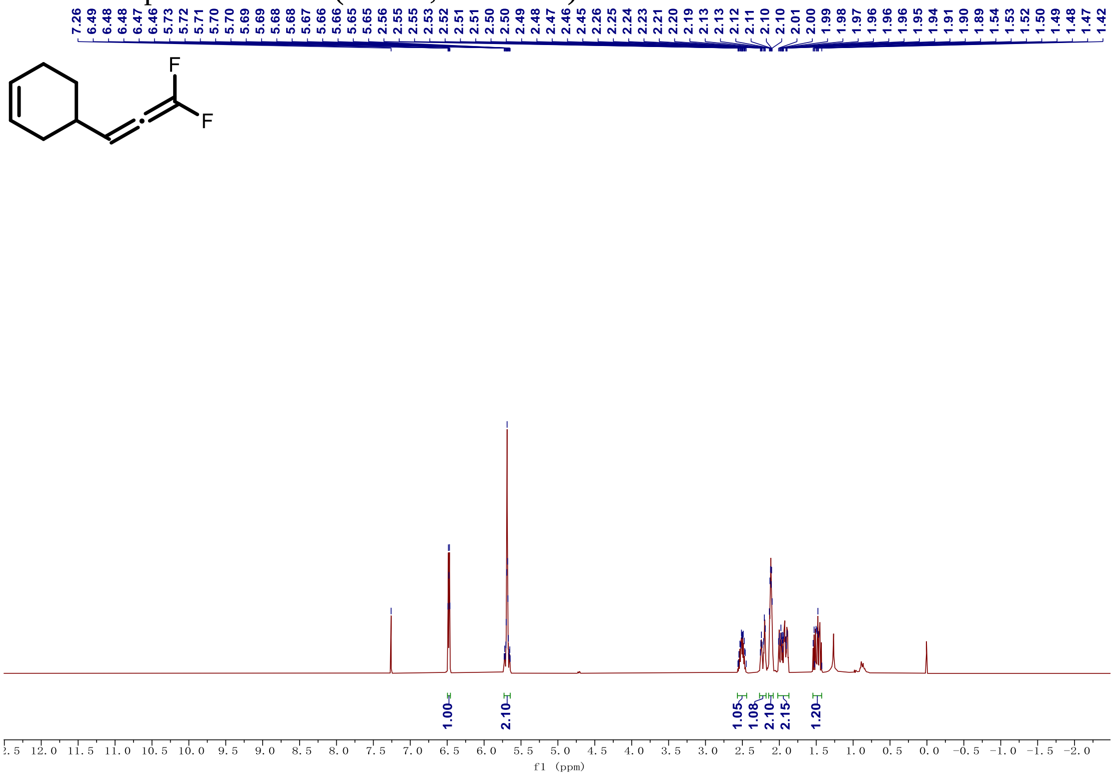

^{19}F NMR Spectrum of **1n** ($CDCl_3$, 376 MHz)

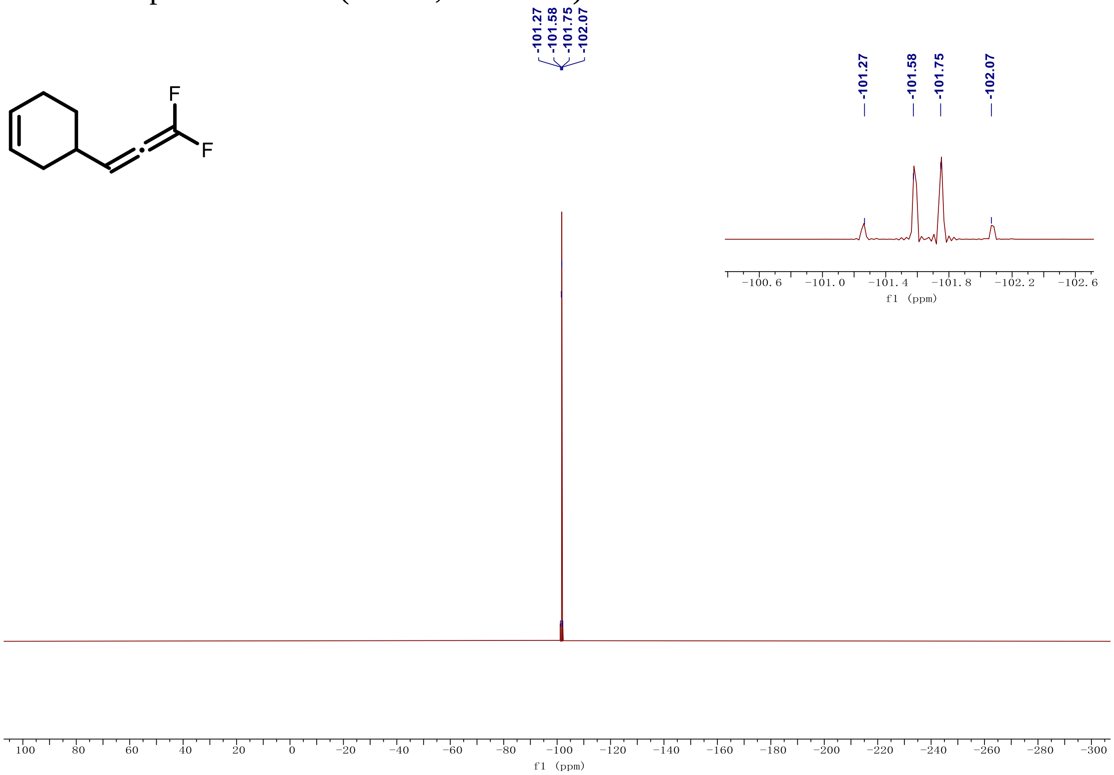

^{13}C NMR Spectrum of **1n** ($CDCl_3$, 101 MHz)

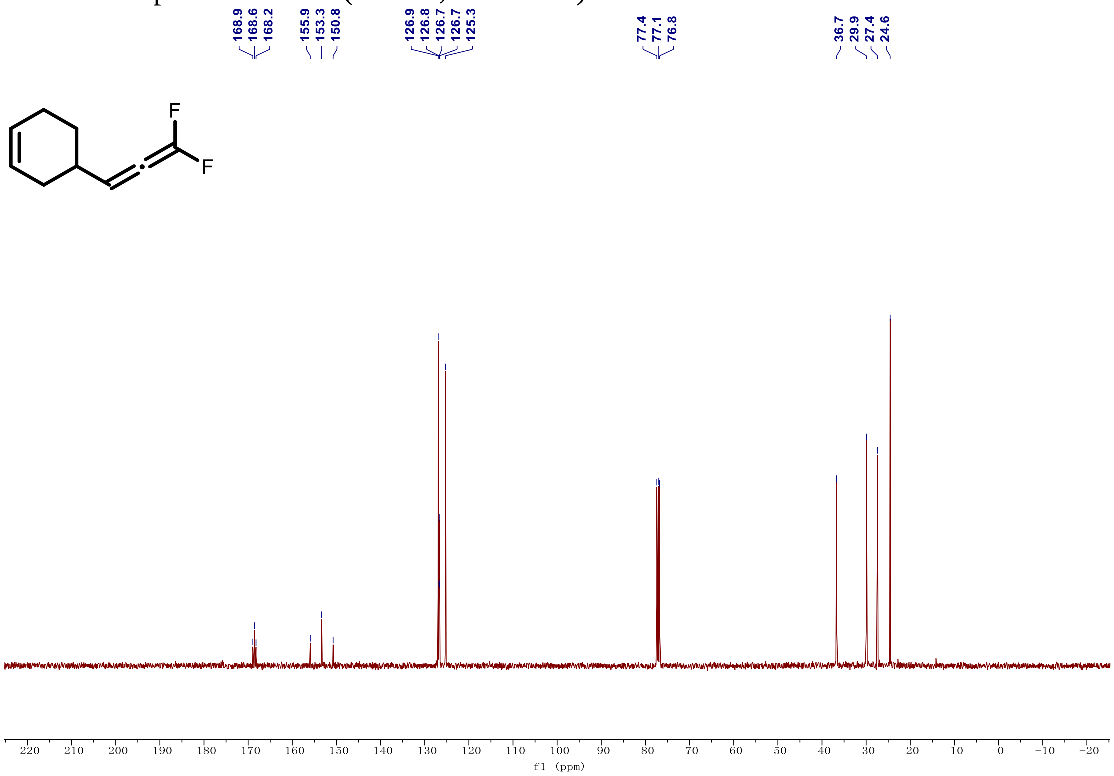

^{1}H NMR Spectrum of **1o** ($CDCl_3$, 400 MHz)

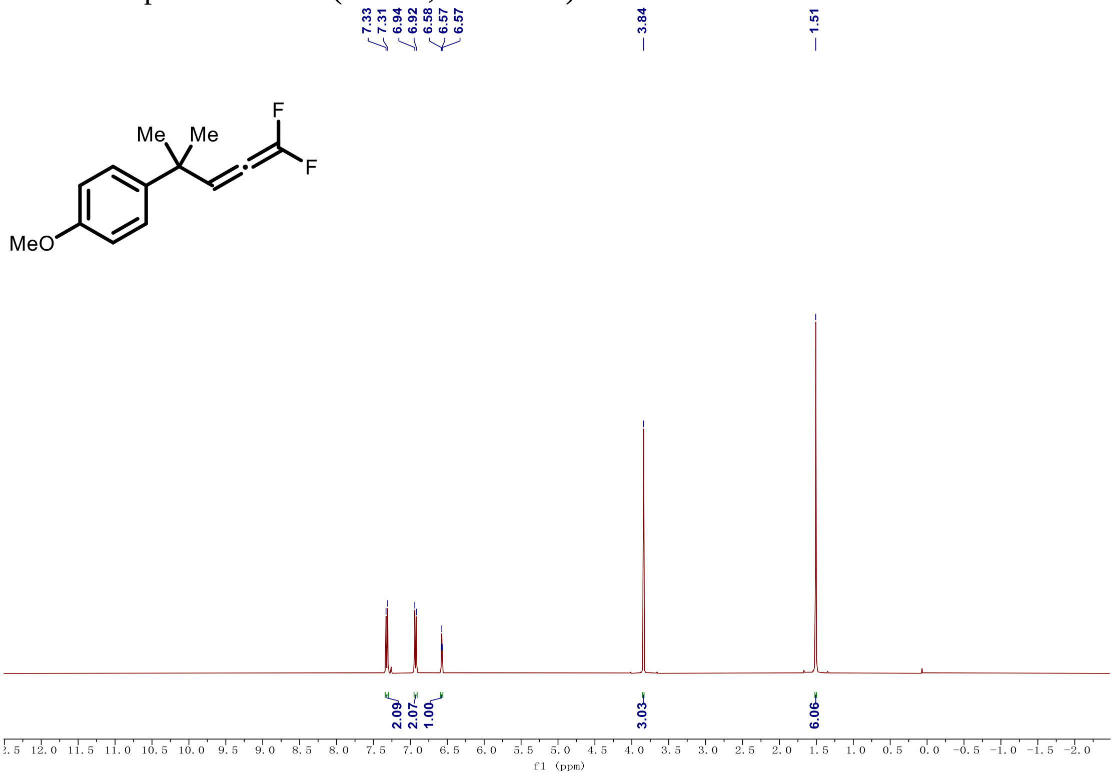

^{19}F NMR Spectrum of **1o** ($CDCl_3$, 376 MHz)

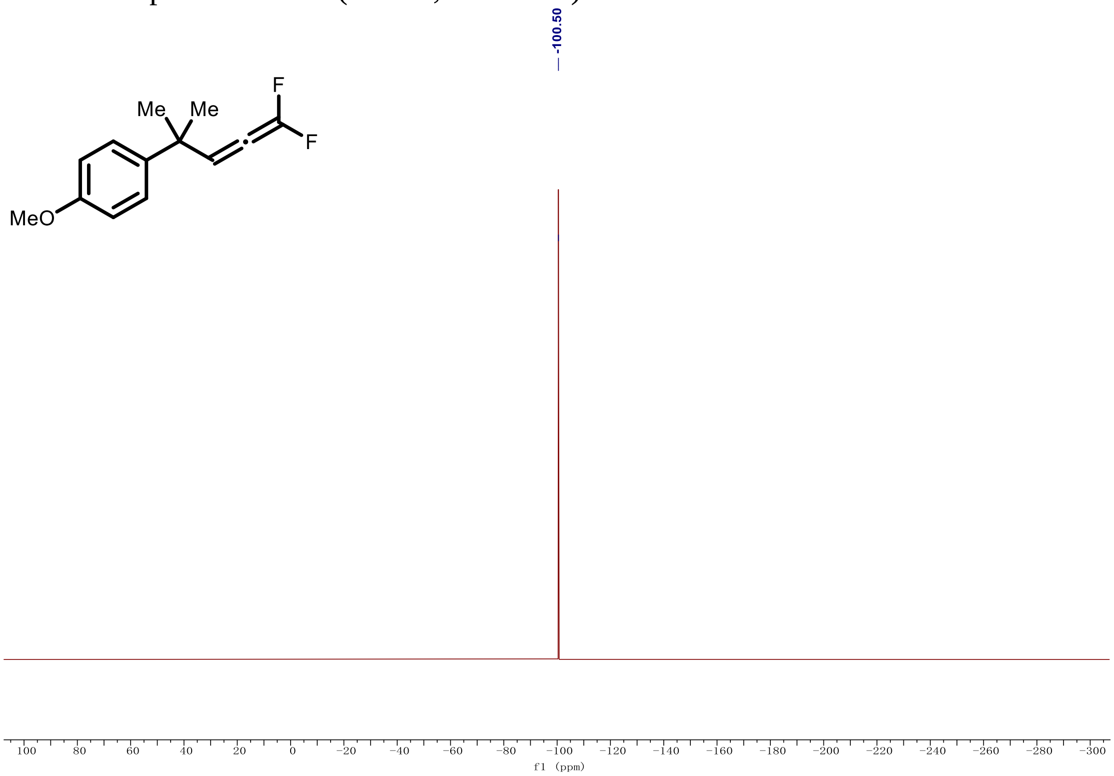

^{13}C NMR Spectrum of **1o** ($CDCl_3$, 101 MHz)

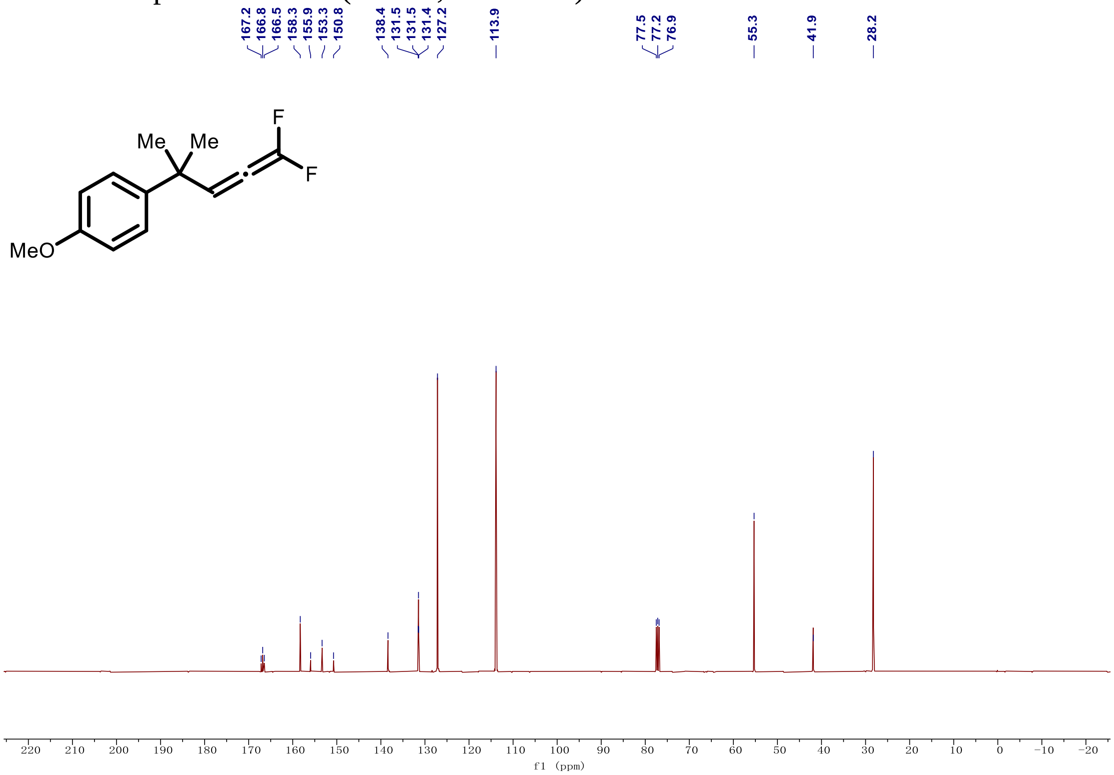

^{1}H NMR Spectrum of **1p** ($CDCl_3$, 400 MHz)

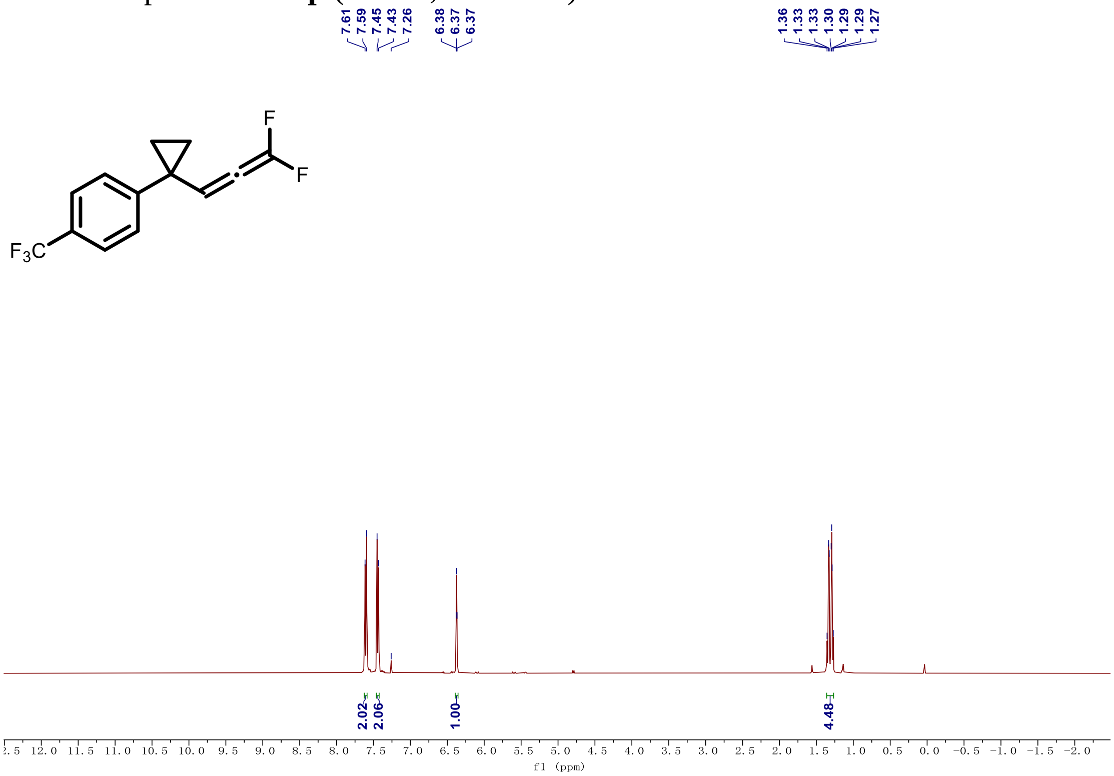

^{19}F NMR Spectrum of **1p** ($CDCl_3$, 376 MHz)

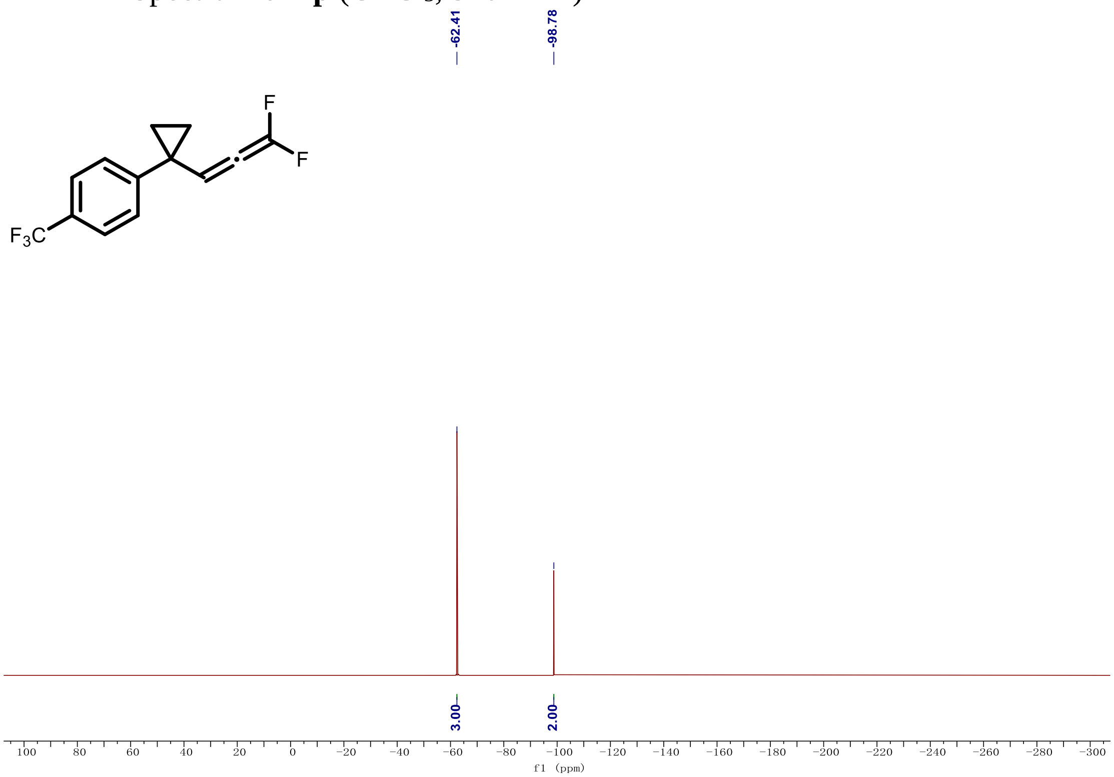

^{13}C NMR Spectrum of **1p** ($CDCl_3$, 101 MHz)

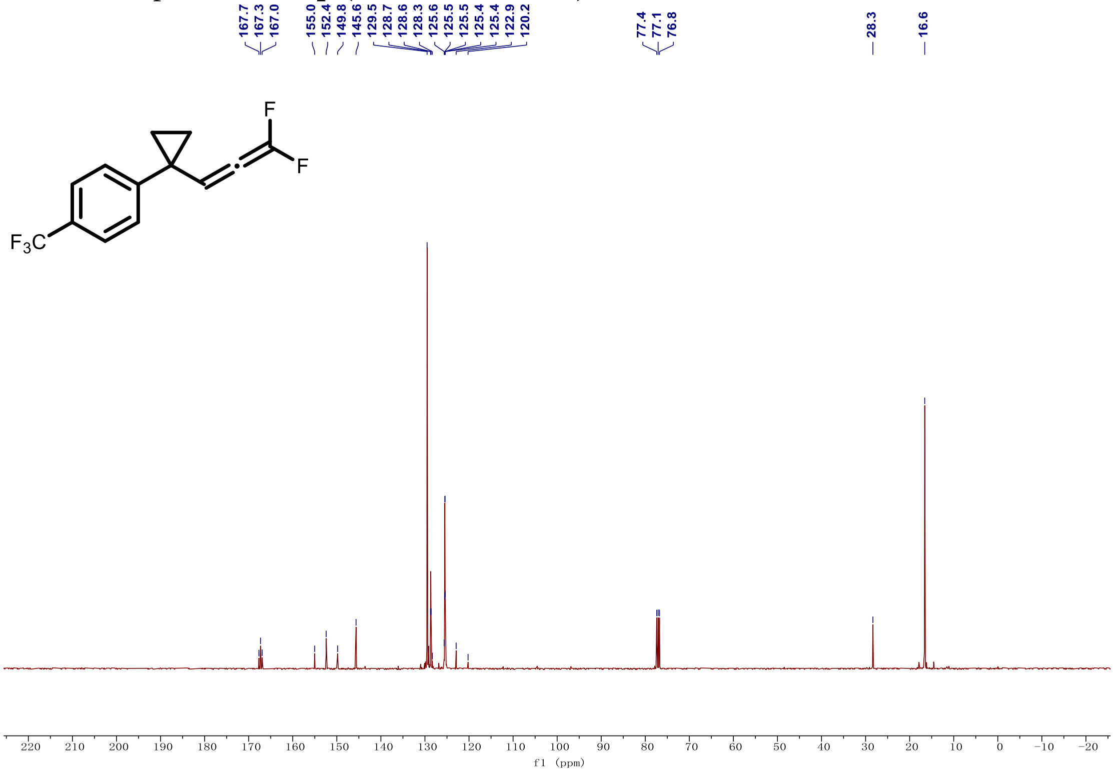

^{1}H NMR Spectrum of **1q** ($CDCl_3$, 400 MHz)

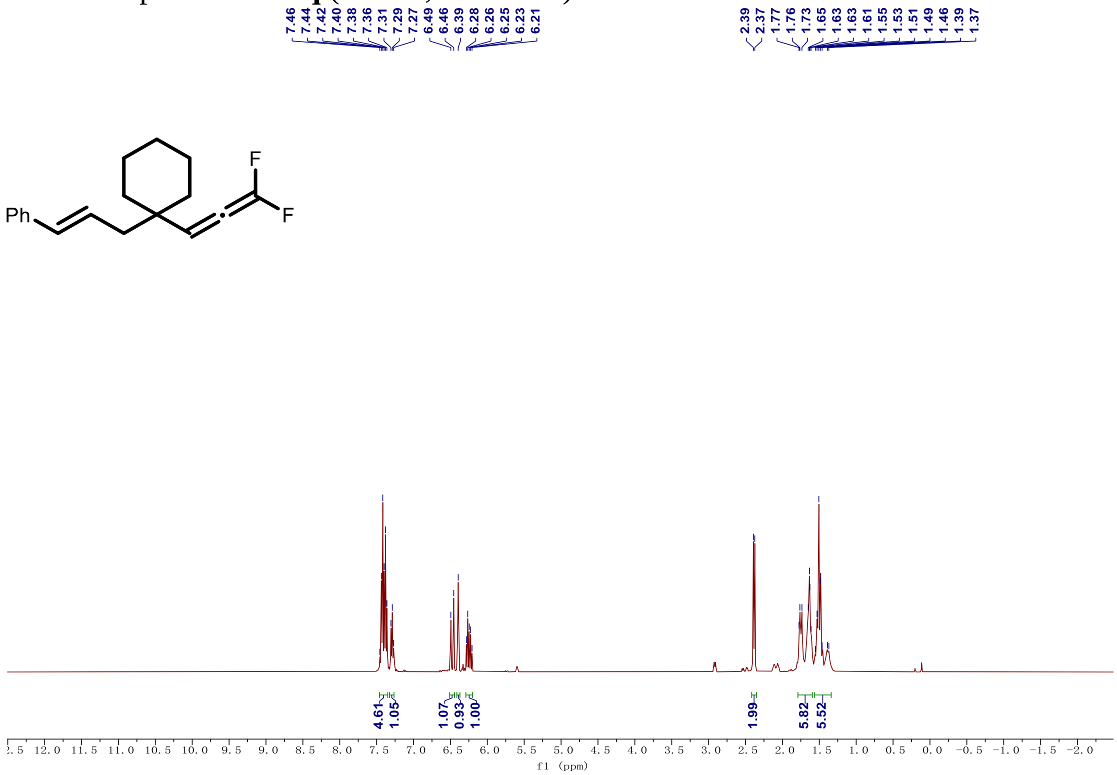

^{19}F NMR Spectrum of **1q** ($CDCl_3$, 376 MHz)

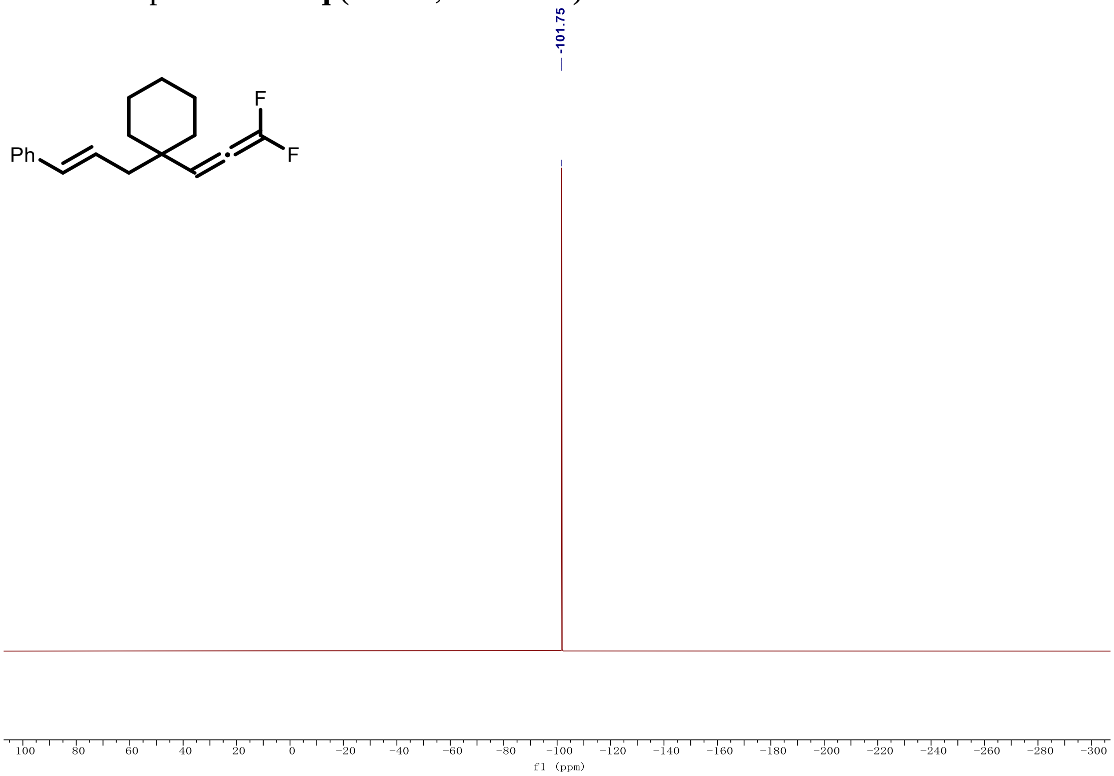

^{13}C NMR Spectrum of **1q** ($CDCl_3$, 101 MHz)

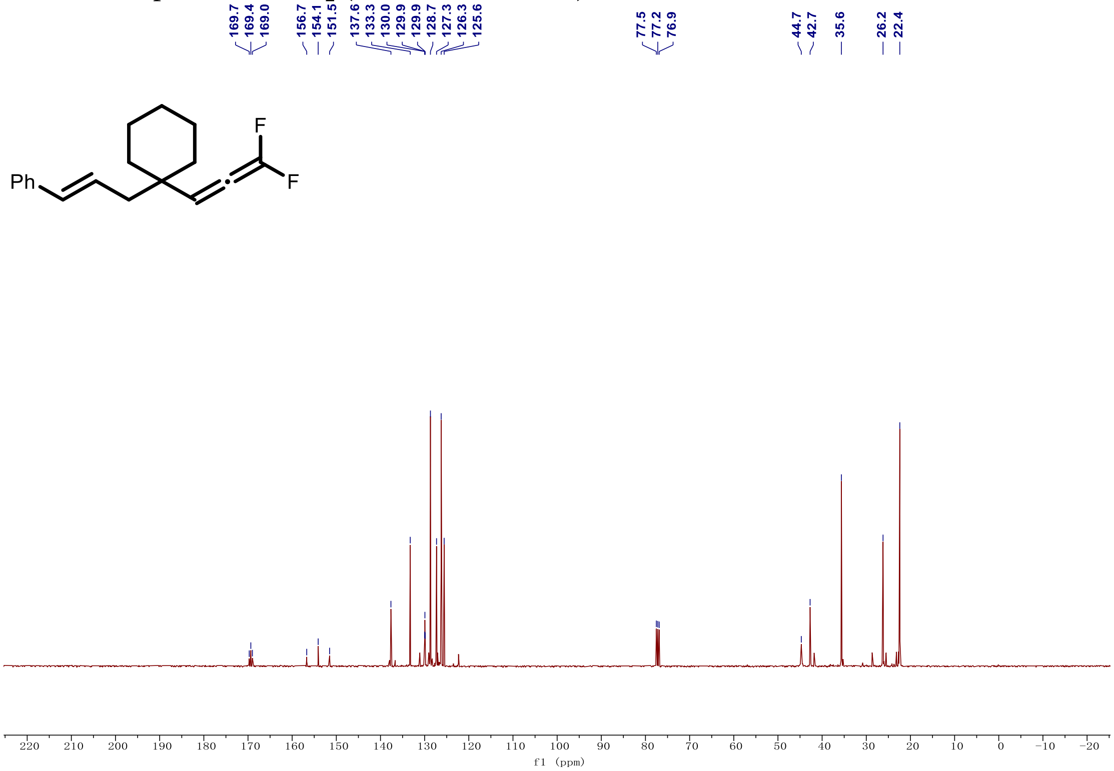

^{1}H NMR Spectrum of **1r** ($CDCl_3$, 400 MHz)

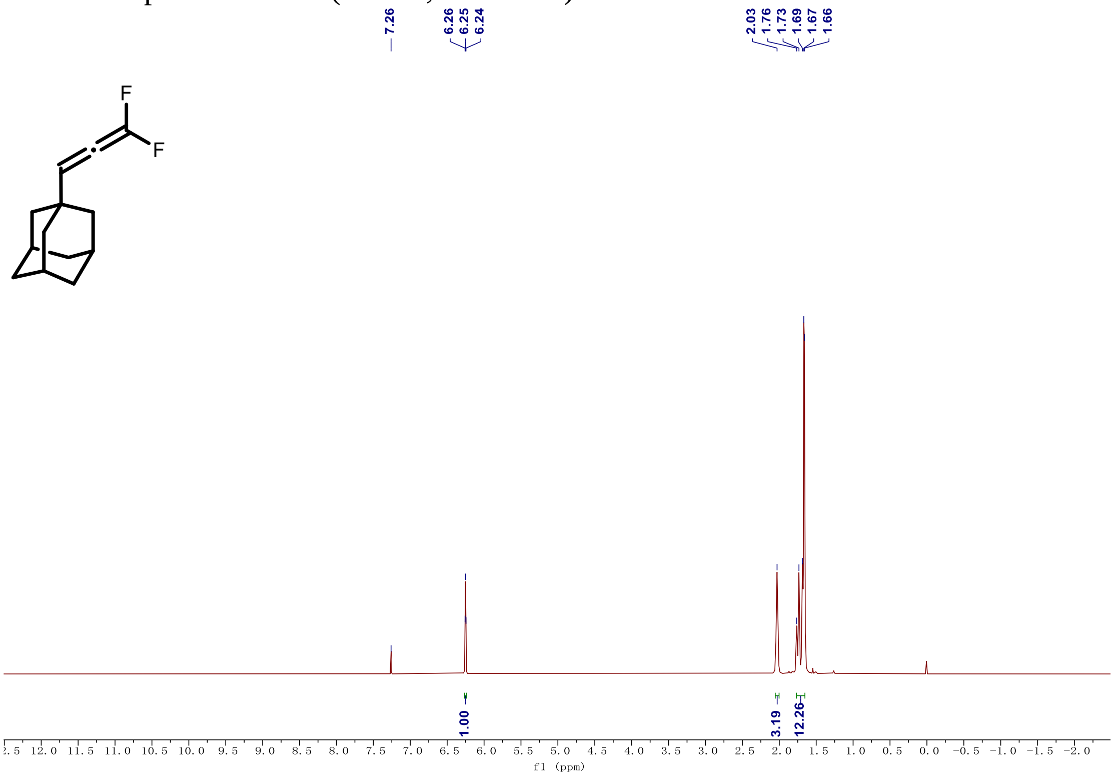

^{19}F NMR Spectrum of **1r** ($CDCl_3$, 376 MHz)

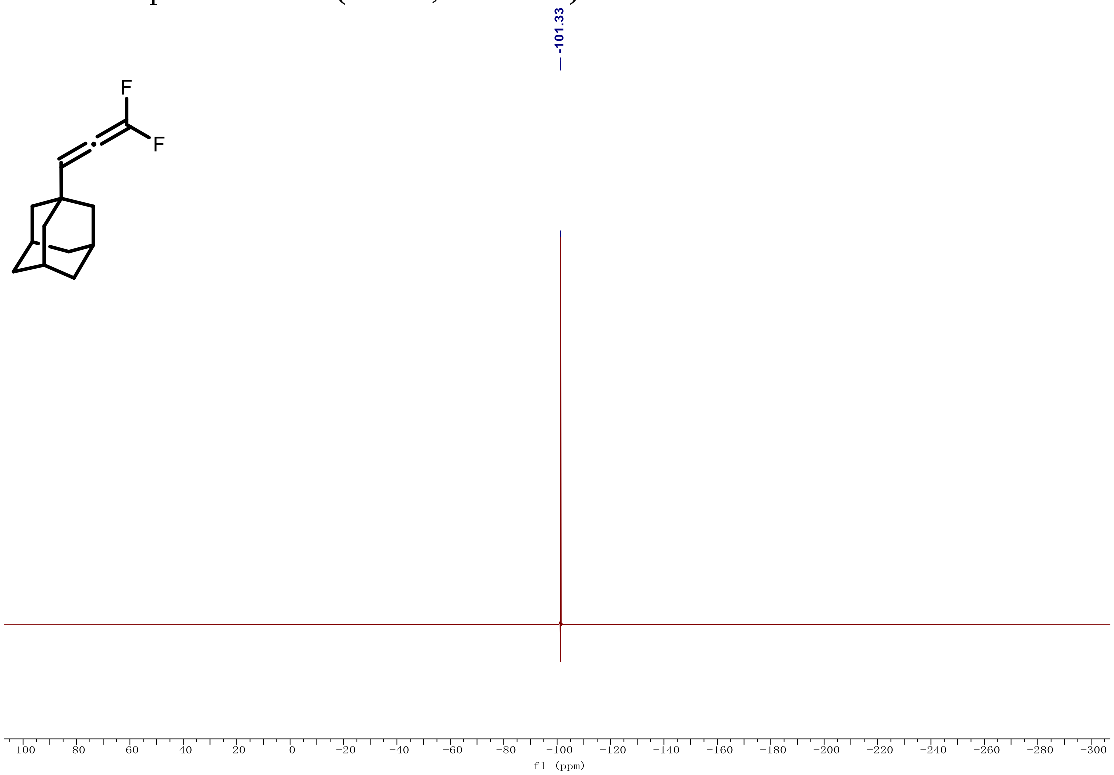

^{13}C NMR Spectrum of **1r** ($CDCl_3$, 101 MHz)

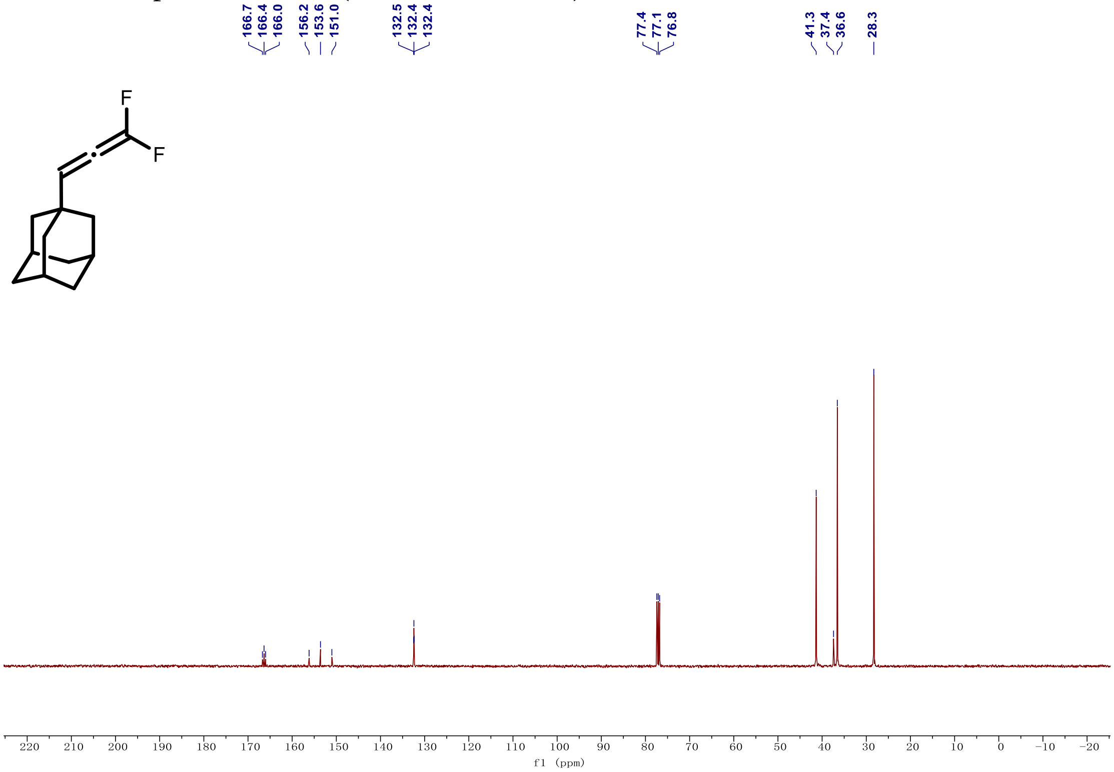

^{1}H NMR Spectrum of **1s** ($CDCl_3$, 400 MHz)

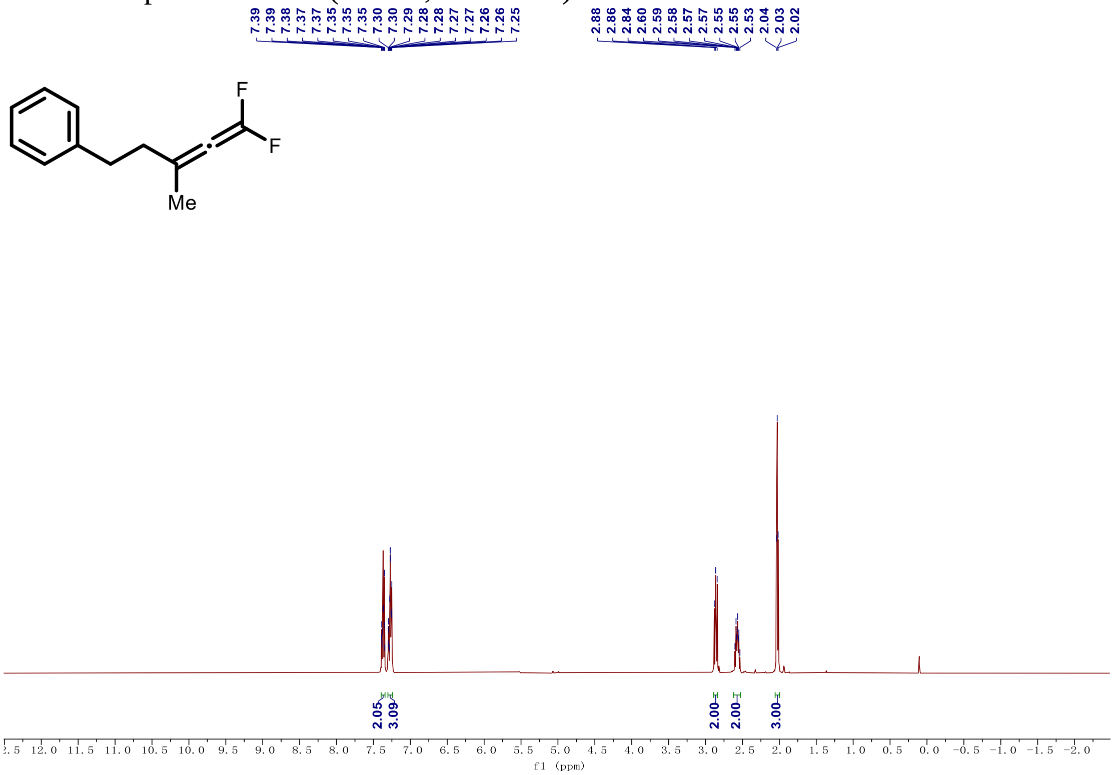

^{19}F NMR Spectrum of **1s** ($CDCl_3$, 376 MHz)

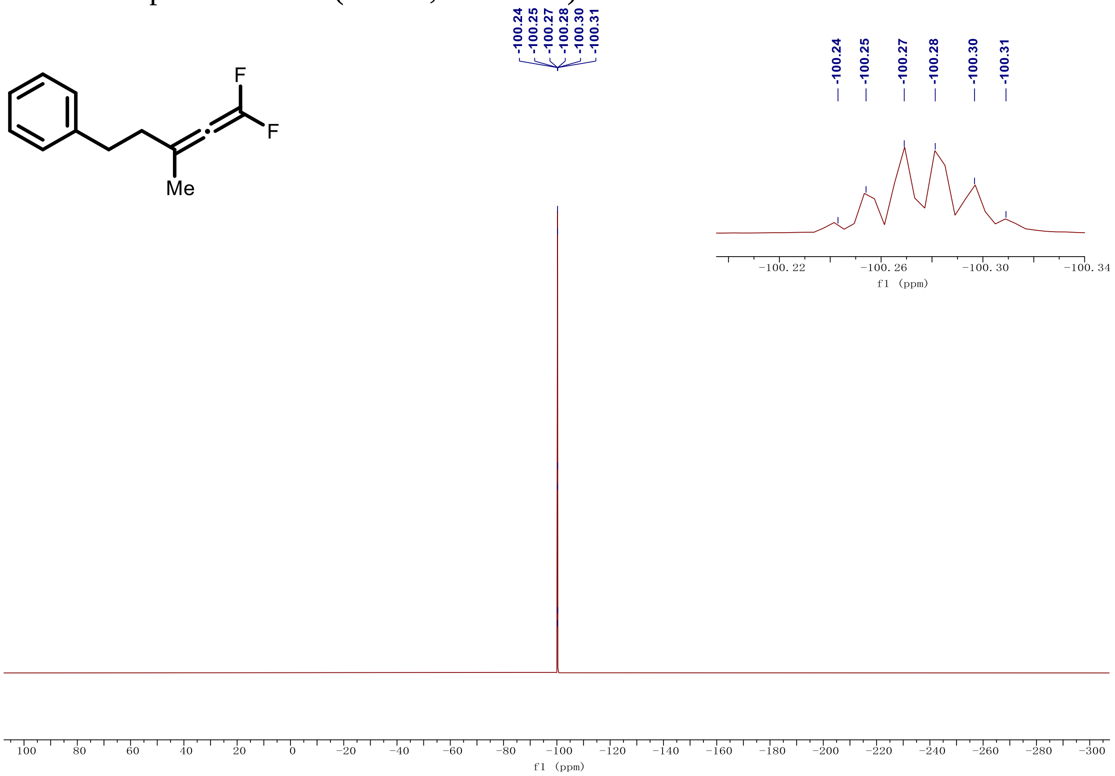

^{13}C NMR Spectrum of **1s** ($CDCl_3$, 101 MHz)

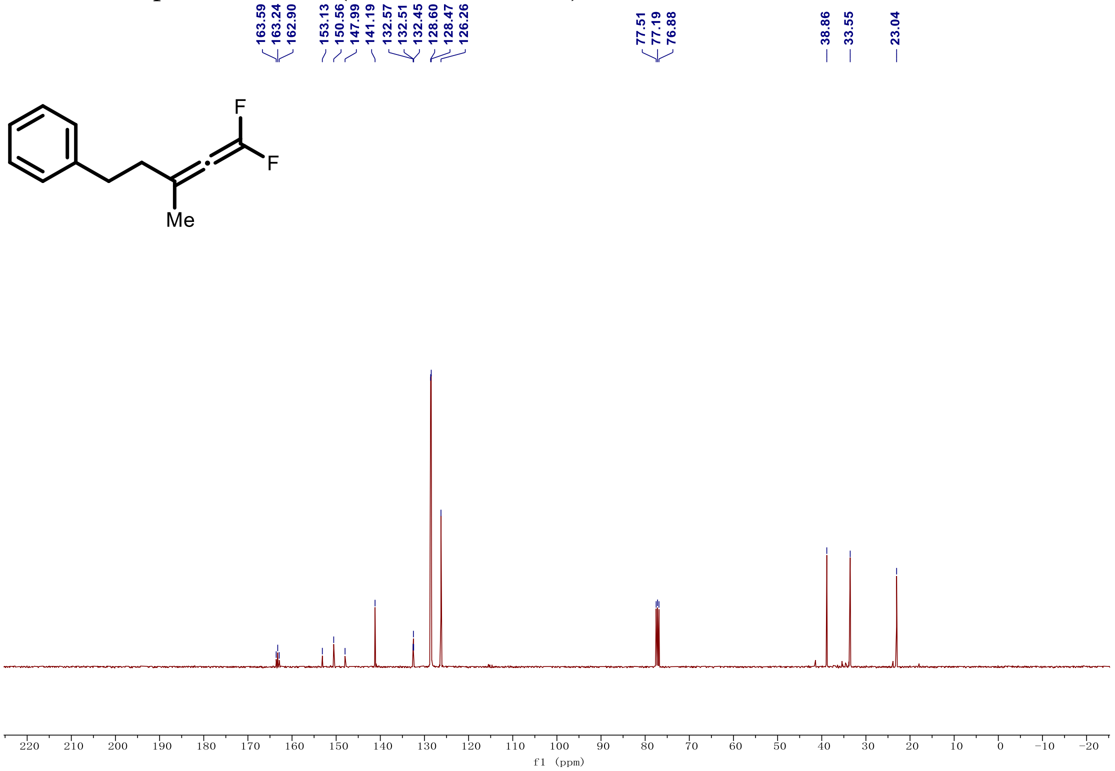

^{1}H NMR Spectrum of **1t** ($CDCl_3$, 400 MHz)

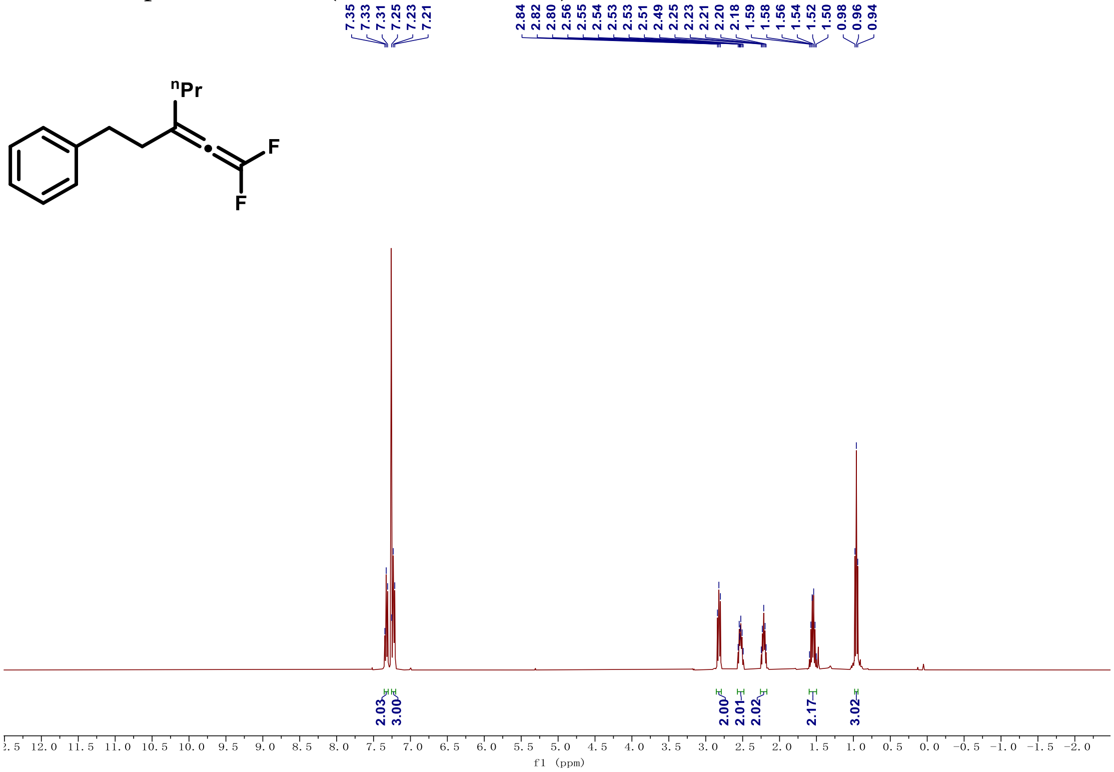

^{19}F NMR Spectrum of **1t** ($CDCl_3$, 376 MHz)

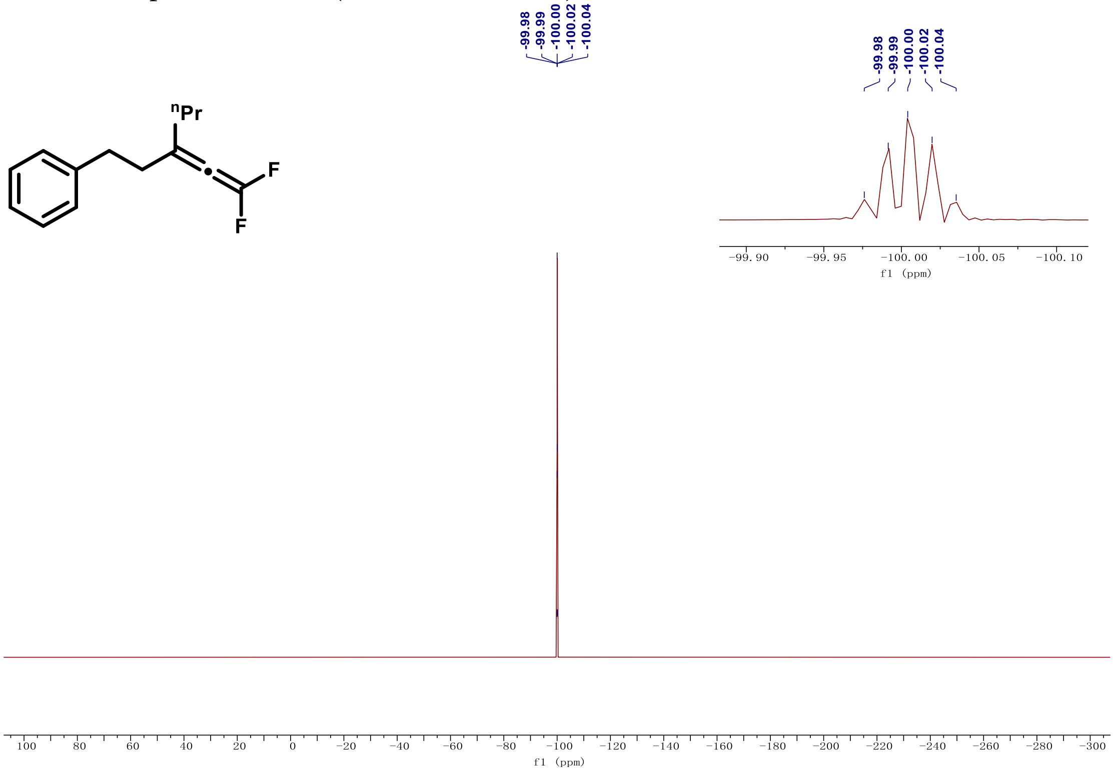

^{13}C NMR Spectrum of **1t** ($CDCl_3$, 101 MHz)

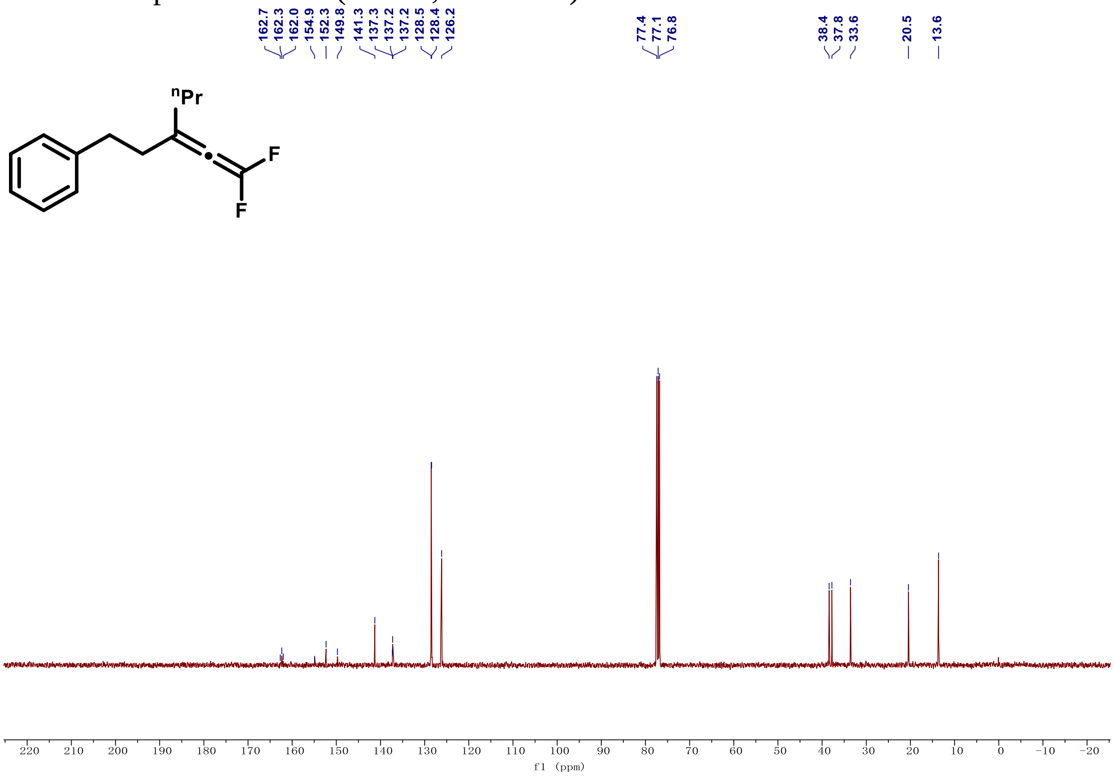

^{1}H NMR Spectrum of **1u** ($CDCl_3$, 400 MHz)

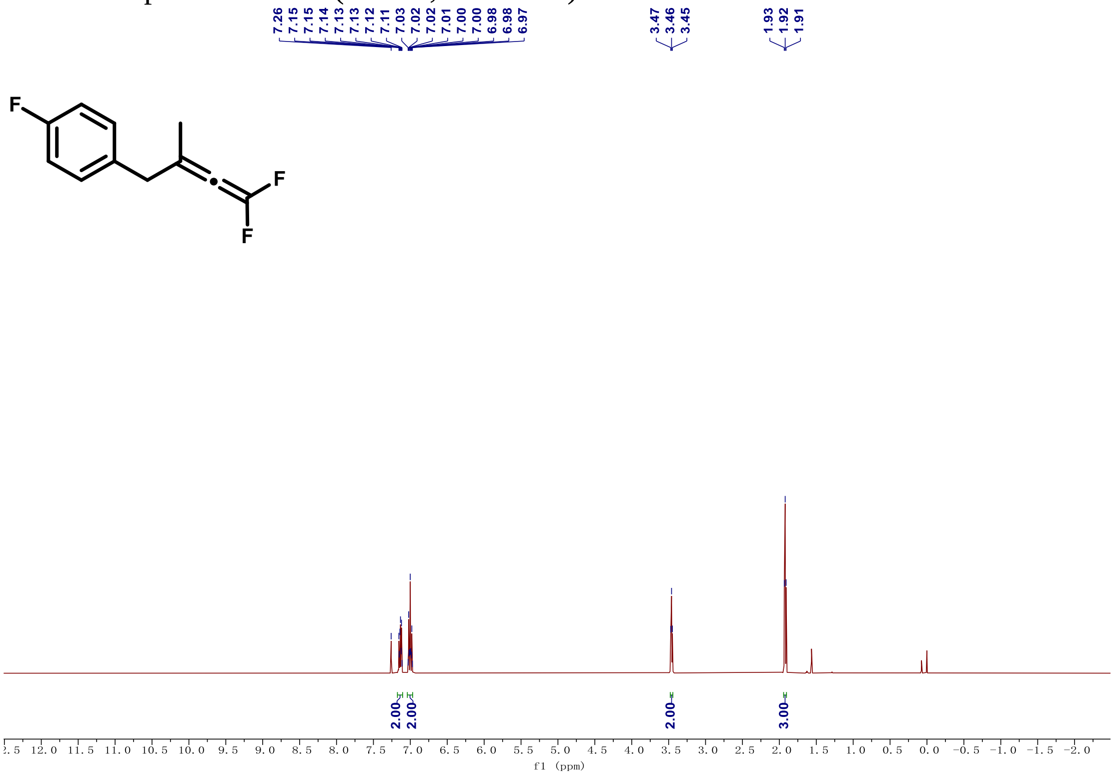

^{19}F NMR Spectrum of **1u** (CDCl$_3$, 376 MHz)

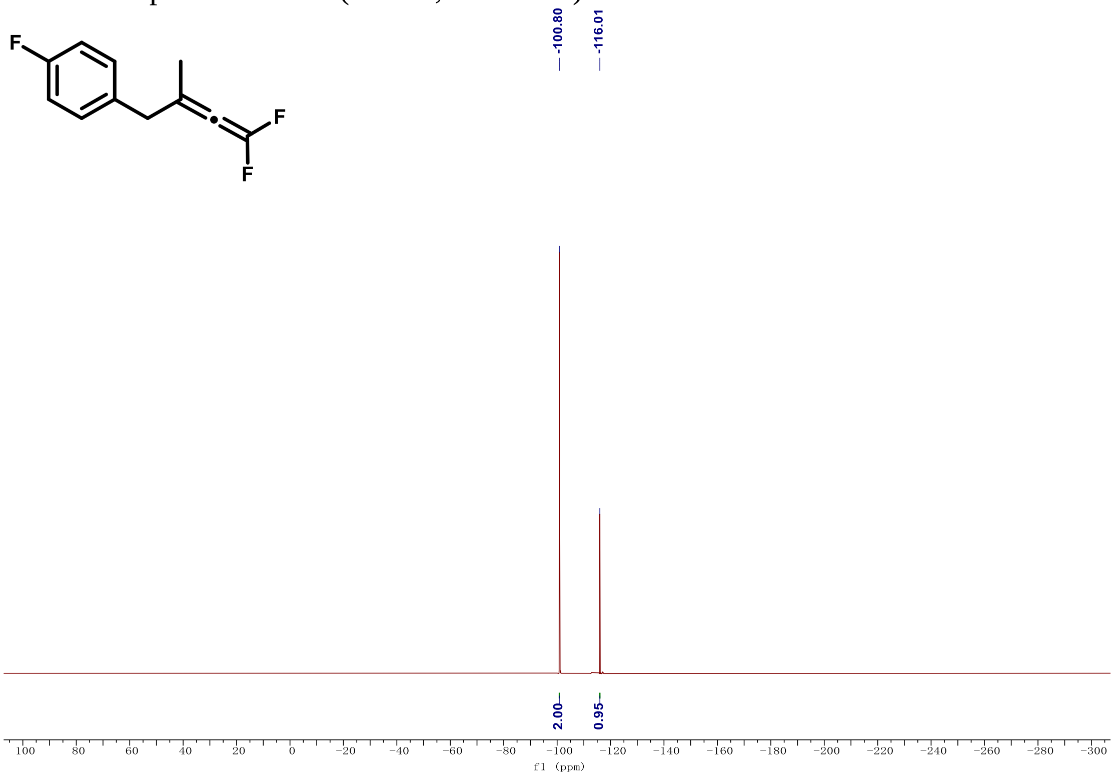

^{13}C NMR Spectrum of **1u**(CDCl$_3$, 101 MHz)

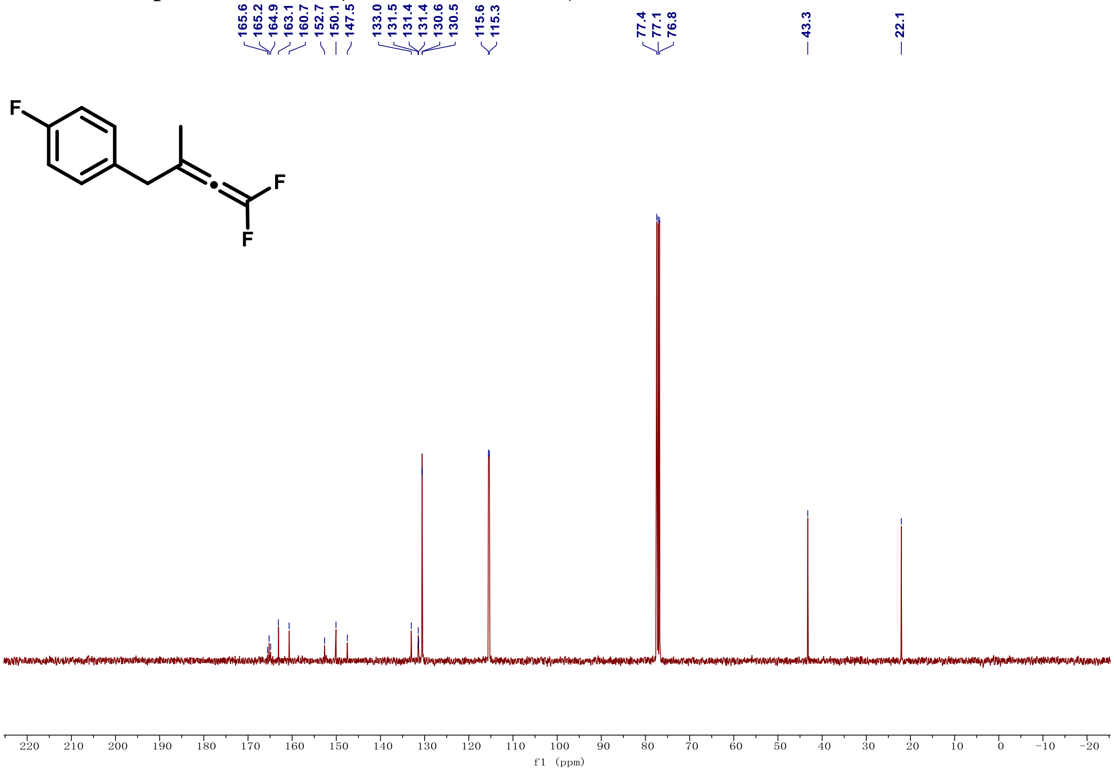

^{1}H NMR Spectrum of **1v** ($CDCl_3$, 400 MHz)

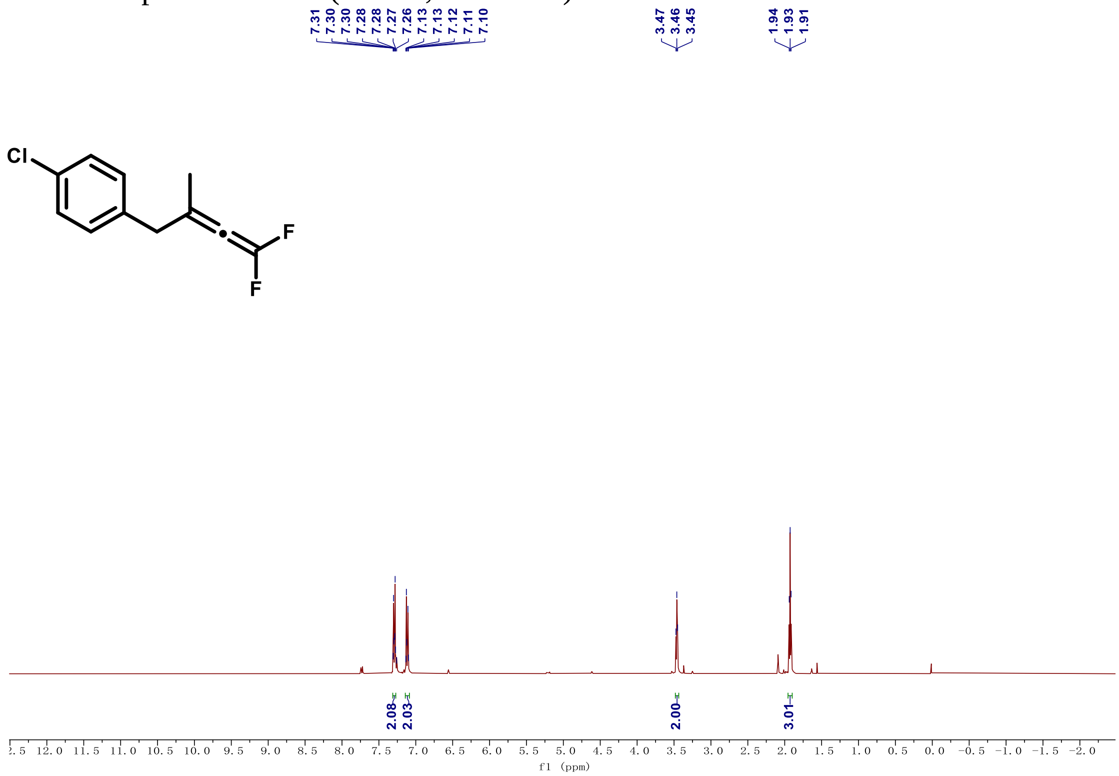

^{19}F NMR Spectrum of **1v** ($CDCl_3$, 376 MHz)

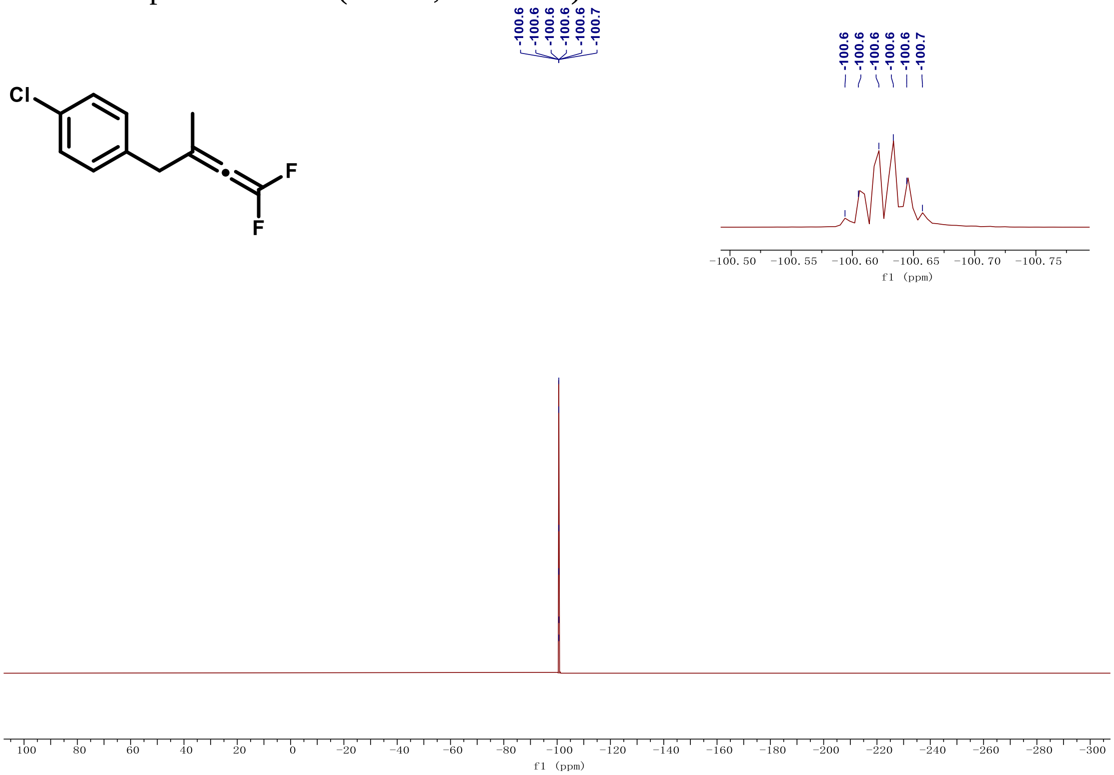

^{13}C NMR Spectrum of **1v** ($CDCl_3$, 101 MHz)

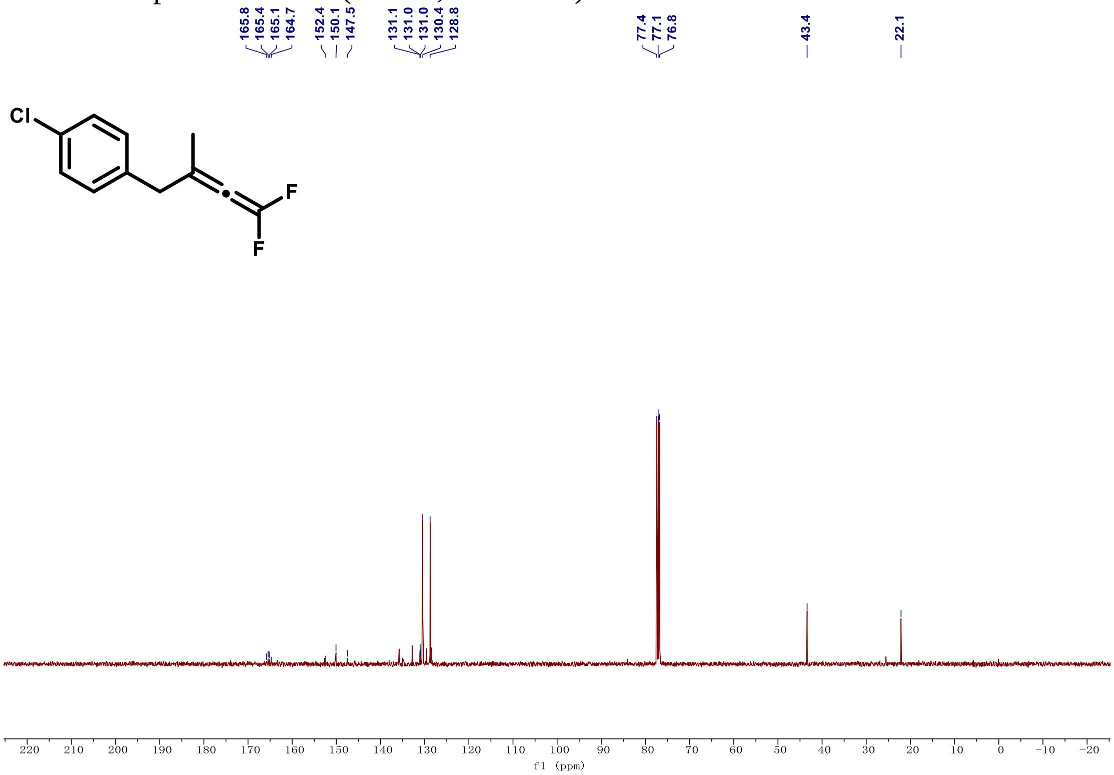

^{1}H NMR Spectrum of **1w** ($CDCl_3$, 400 MHz)

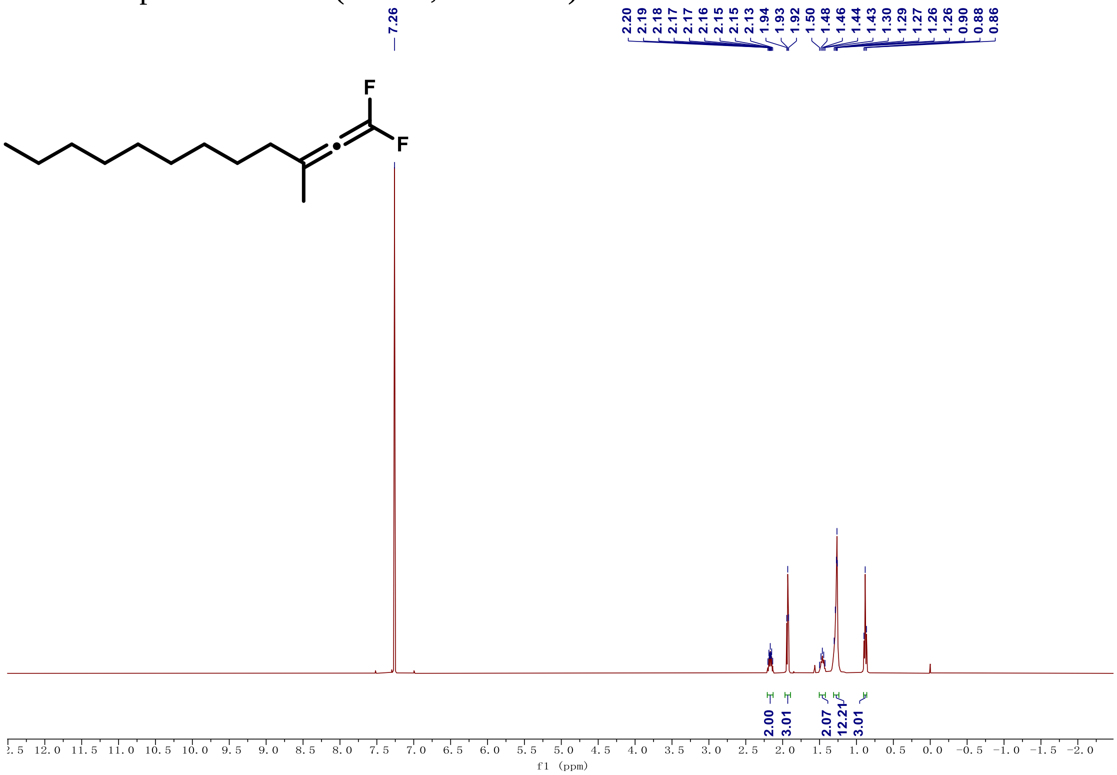

^{19}F NMR Spectrum of **1w** ($CDCl_3$, 376 MHz)

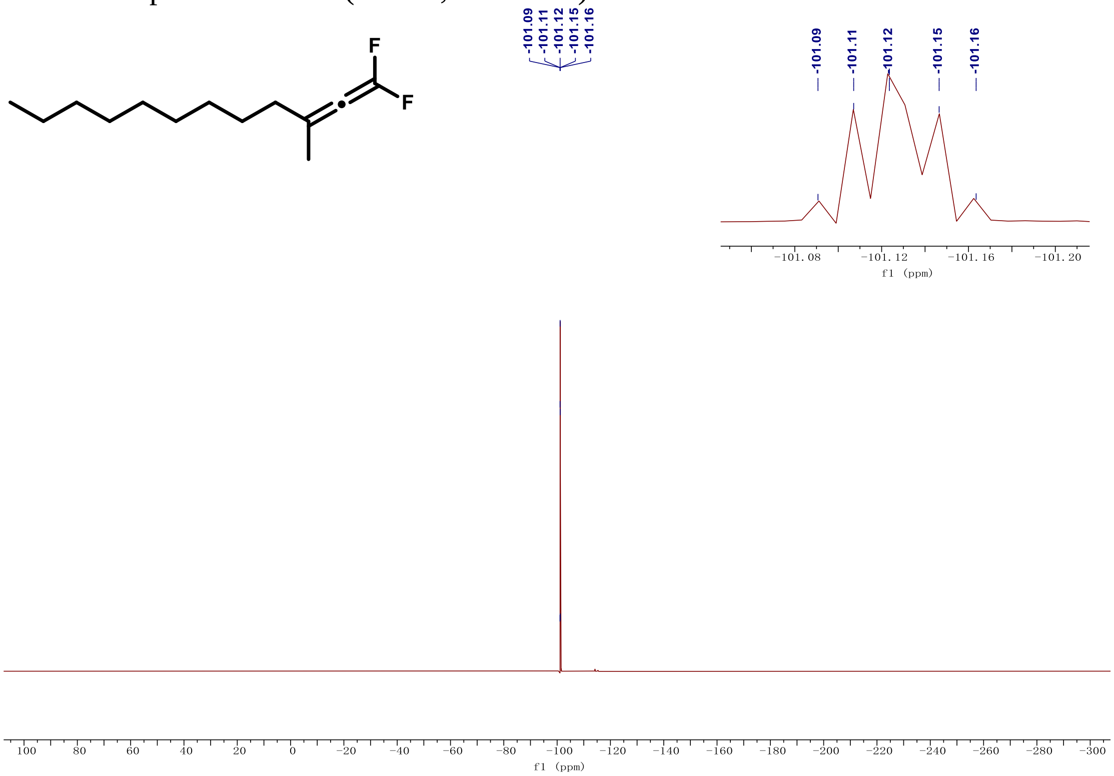

^{13}C NMR Spectrum of **1w** ($CDCl_3$, 101 MHz)

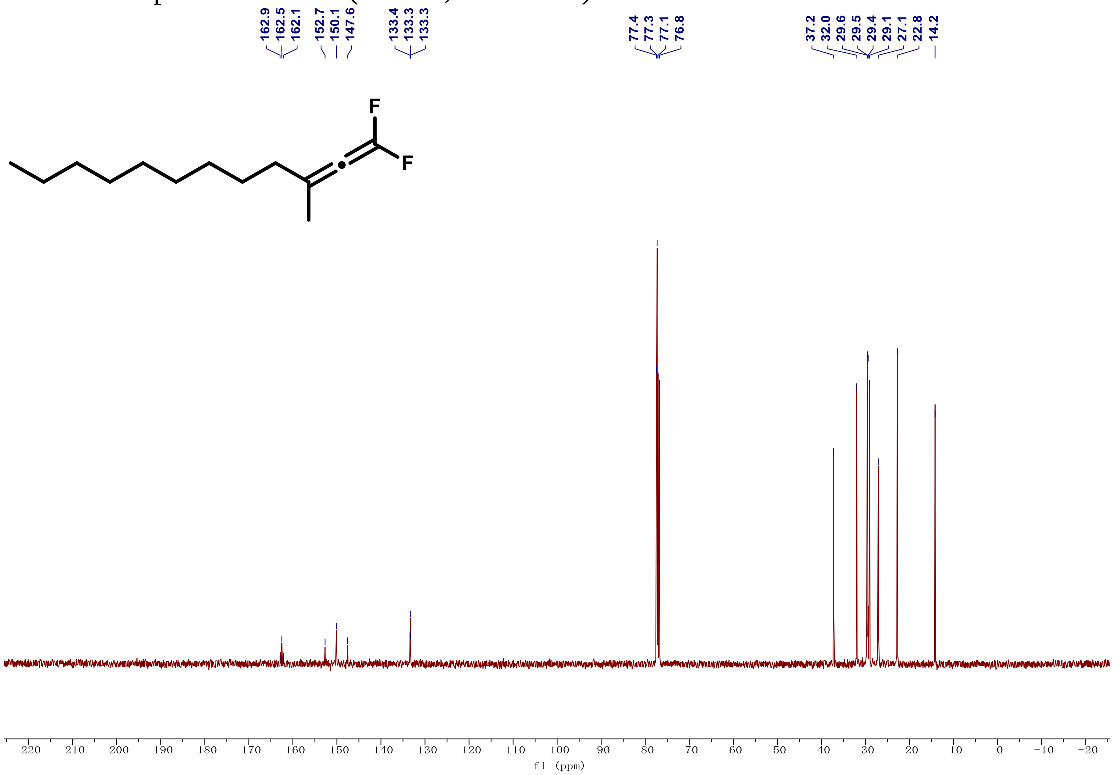

^{1}H NMR Spectrum of **1x** ($CDCl_3$, 400 MHz)

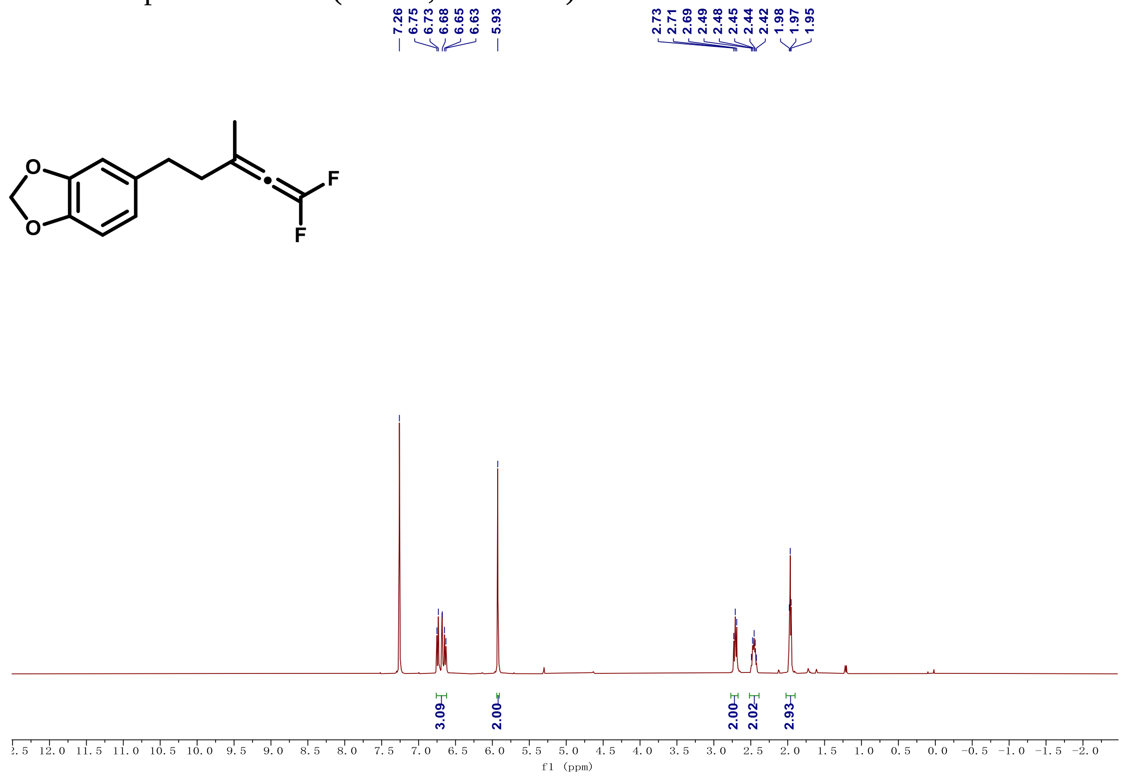

^{19}F NMR Spectrum of **1x** ($CDCl_3$, 376 MHz)

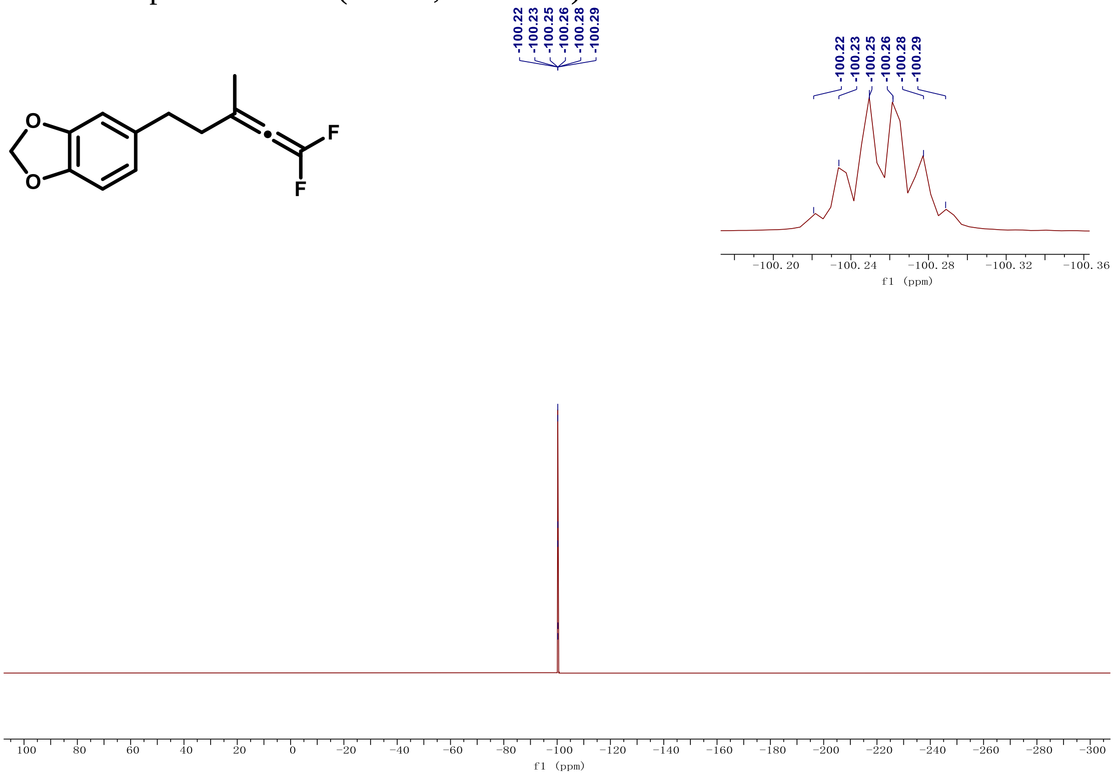

^{13}C NMR Spectrum of **1x** ($CDCl_3$, 101 MHz)

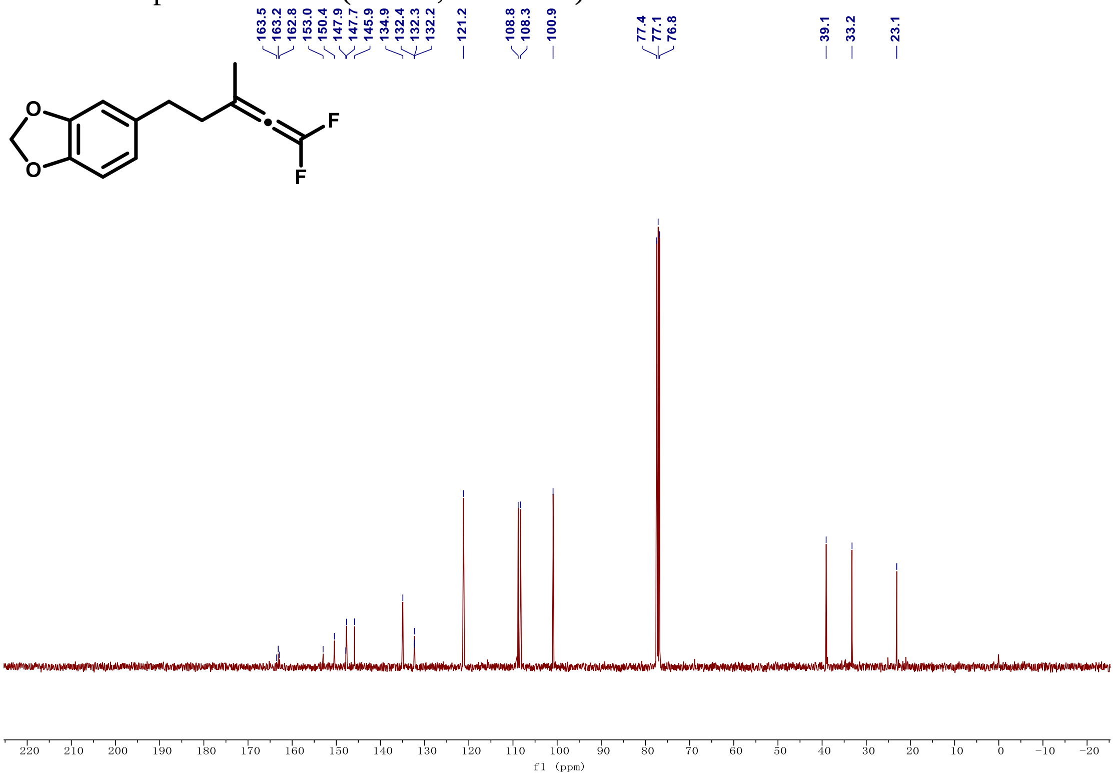

^{1}H NMR Spectrum of **1y** ($CDCl_3$, 400 MHz)

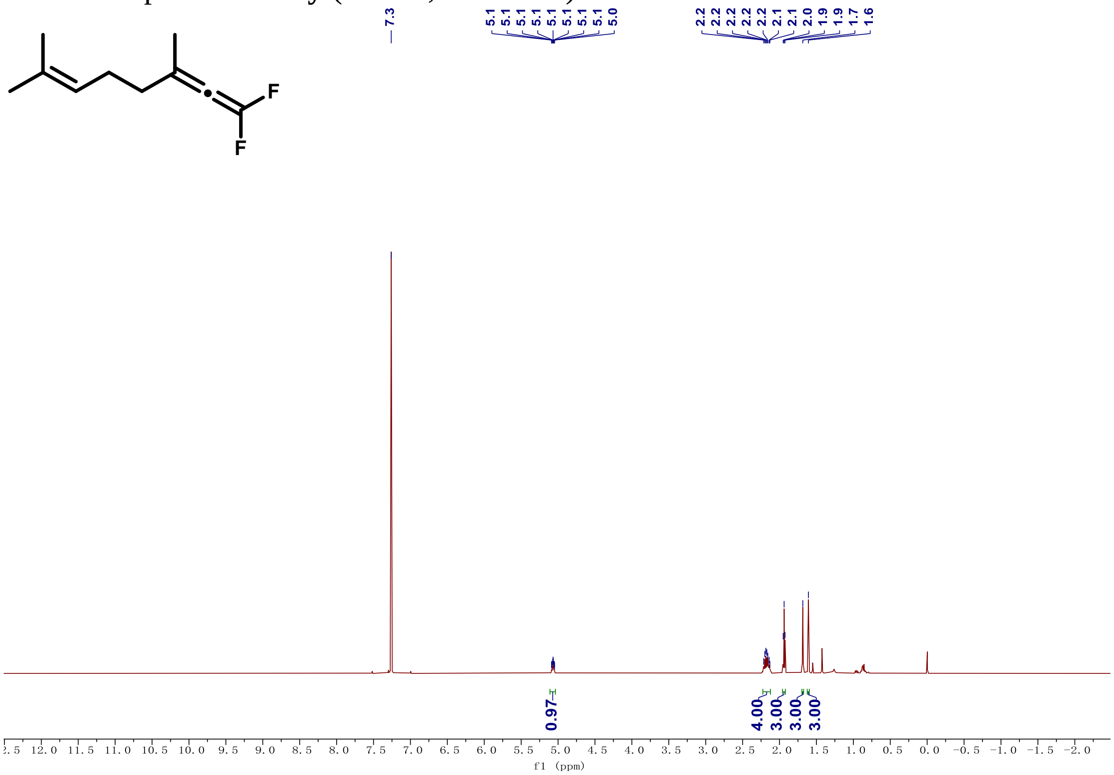

^{19}F NMR Spectrum of **1y** ($CDCl_3$, 376 MHz)

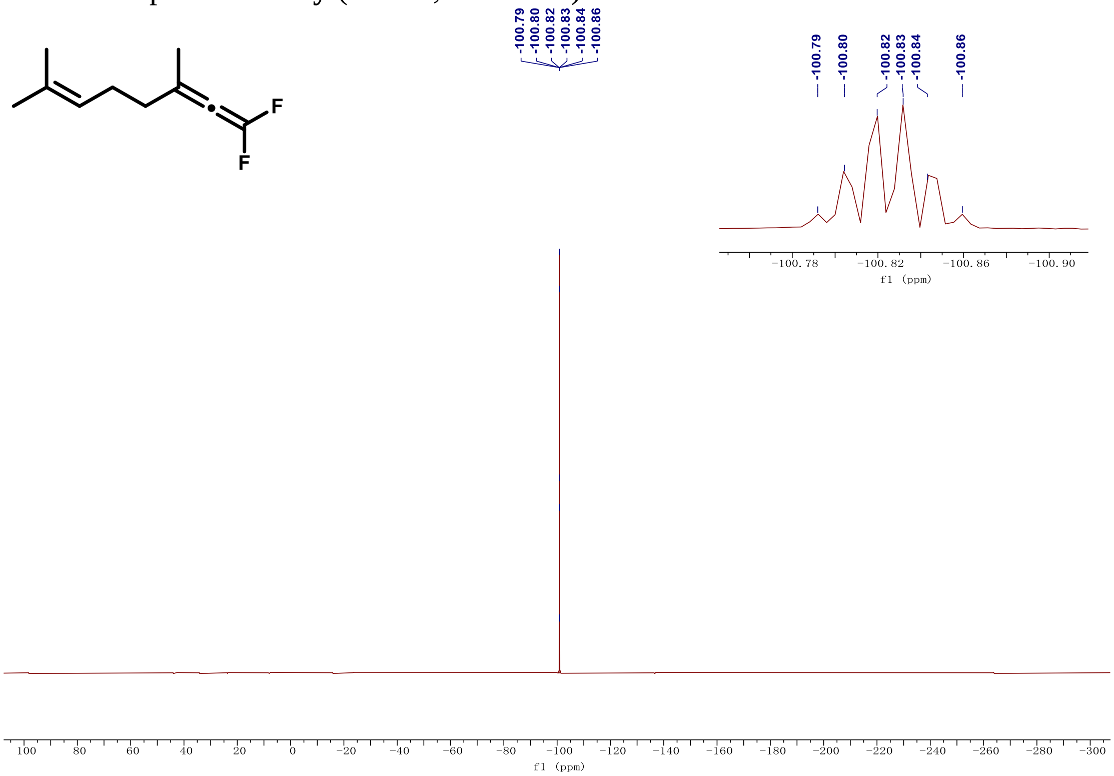

^{13}C NMR Spectrum of **1y** ($CDCl_3$, 101 MHz)

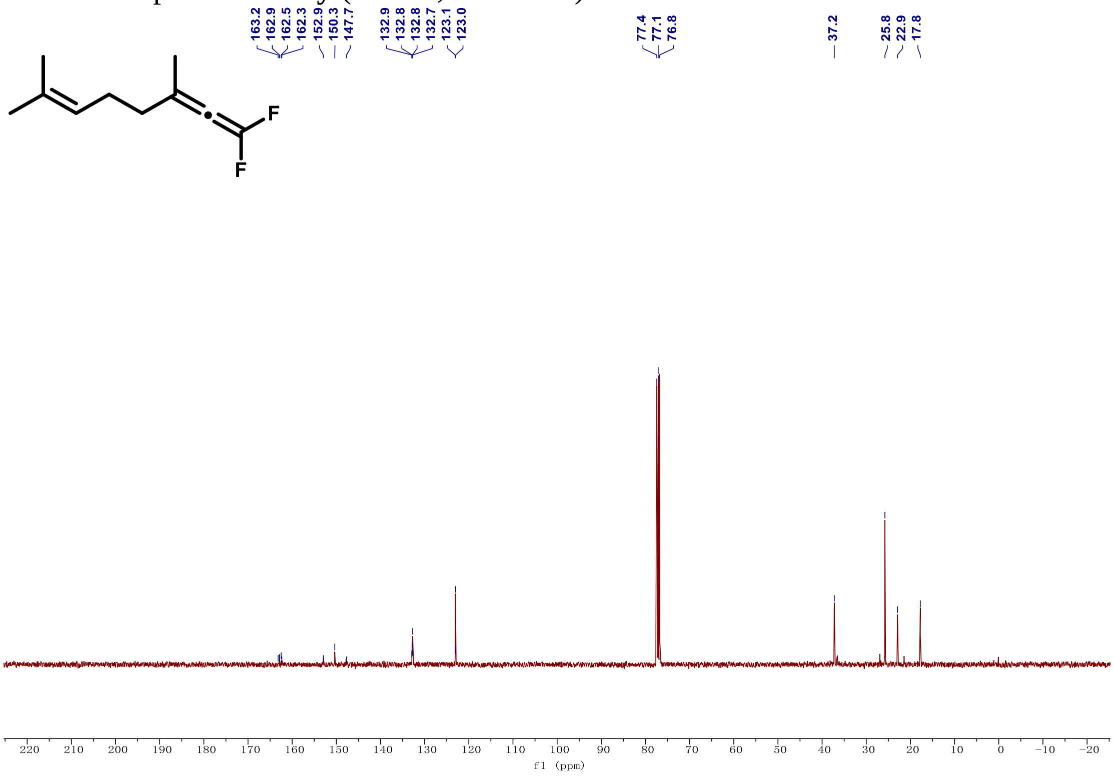

^{1}H NMR Spectrum of **1z** ($CDCl_3$, 400 MHz)

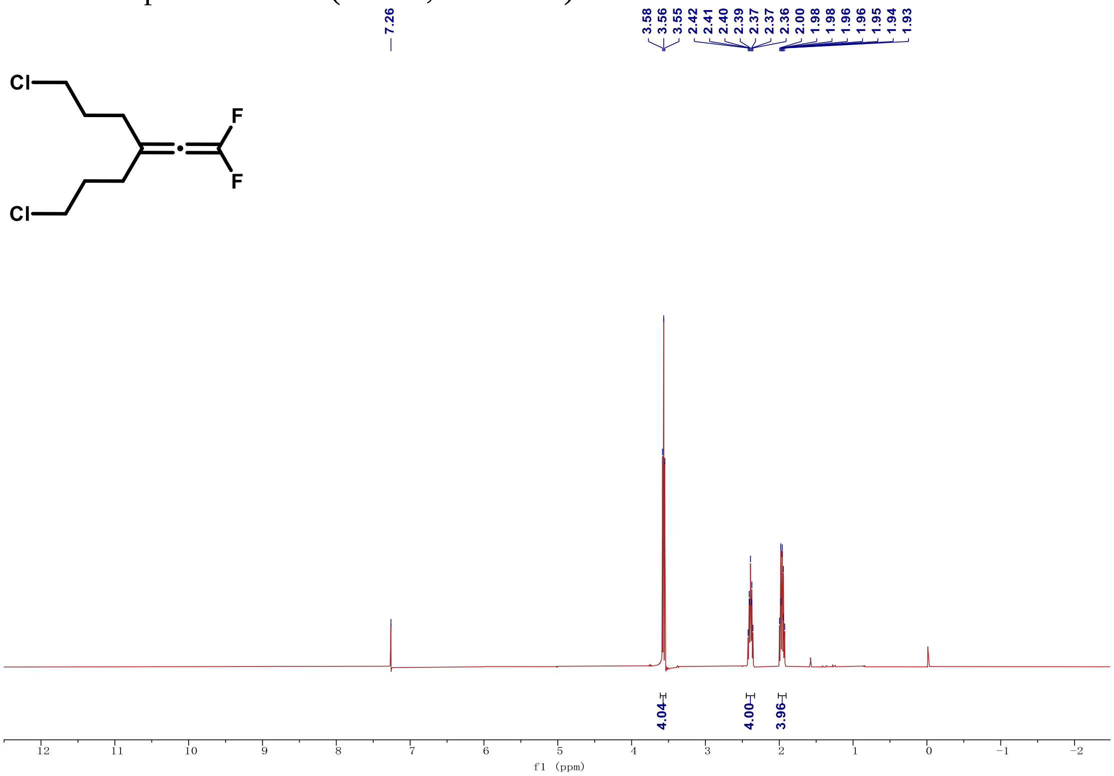

^{19}F NMR Spectrum of **1z** ($CDCl_3$, 376 MHz)

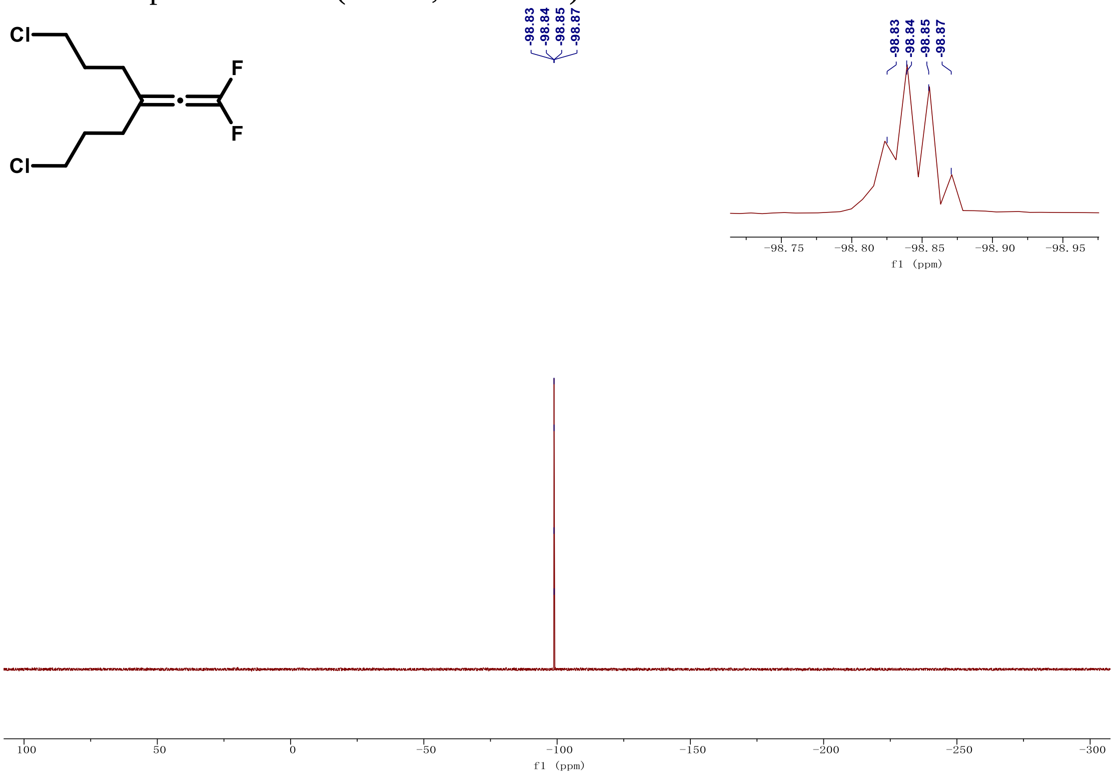

^{13}C NMR Spectrum of **1z** ($CDCl_3$, 101 MHz)

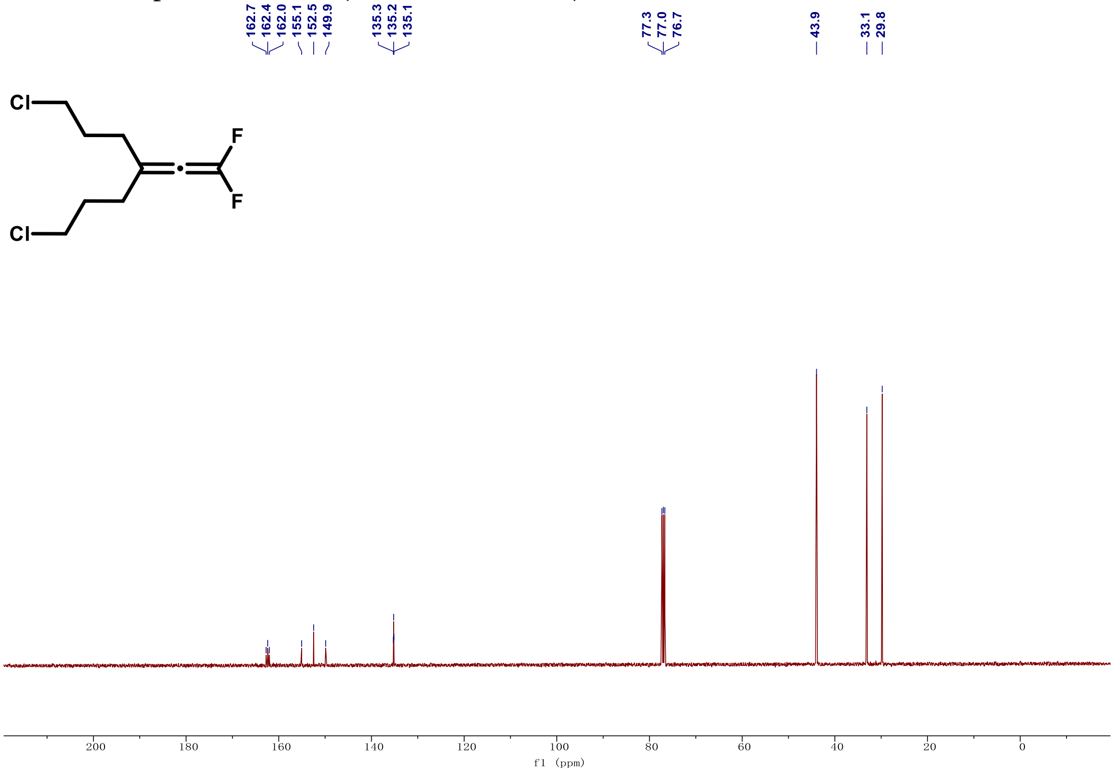

^{1}H NMR Spectrum of **1aa** ($CDCl_3$, 400 MHz)

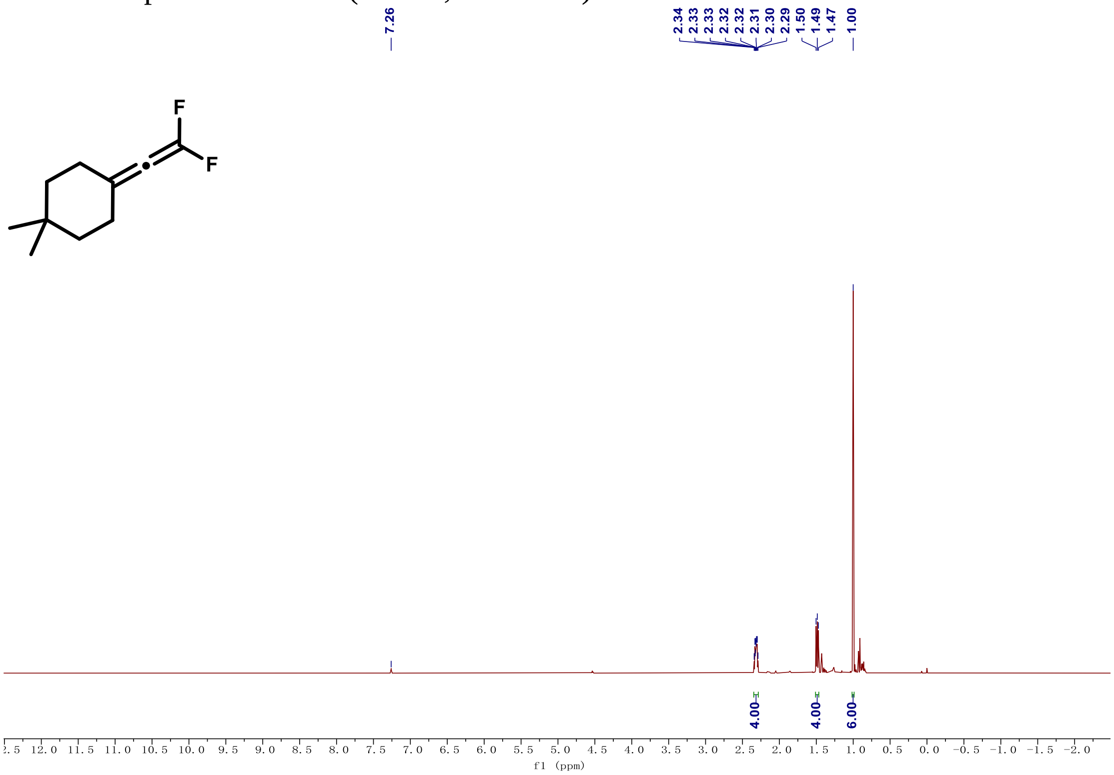

^{19}F NMR Spectrum of **1aa** ($CDCl_3$, 376 MHz)

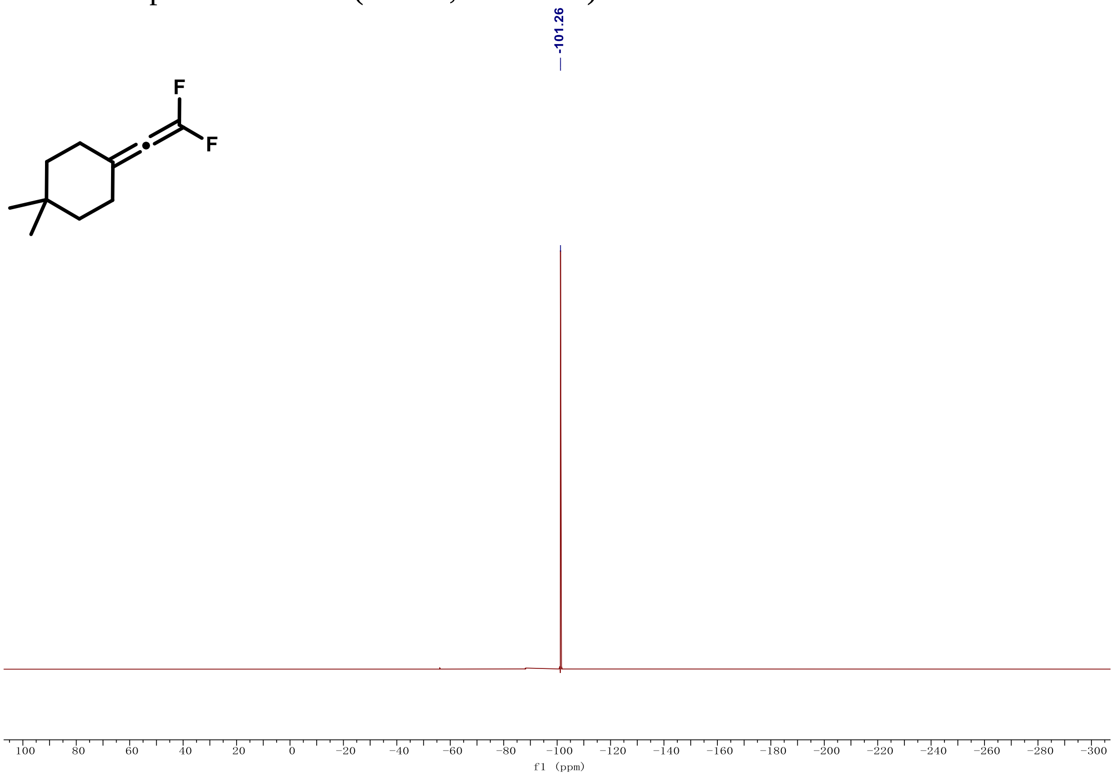

^{13}C NMR Spectrum of **1aa** ($CDCl_3$, 101 MHz)

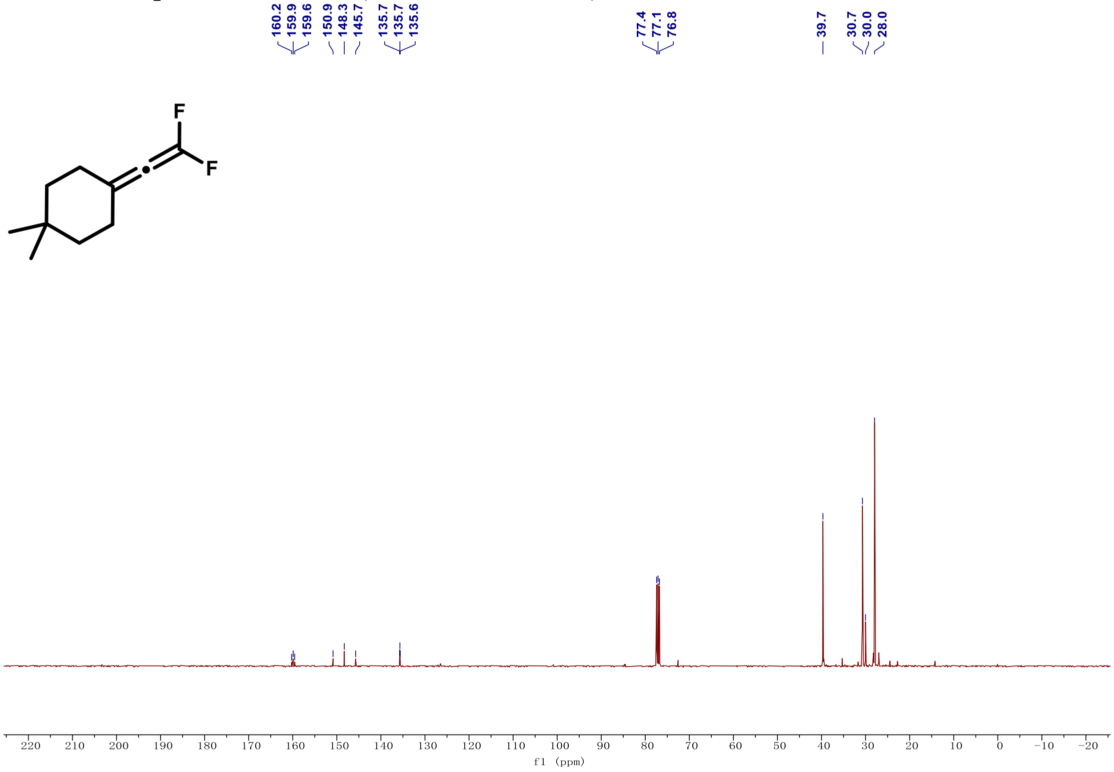

^{1}H NMR Spectrum of **1ab** ($CDCl_3$, 400 MHz)

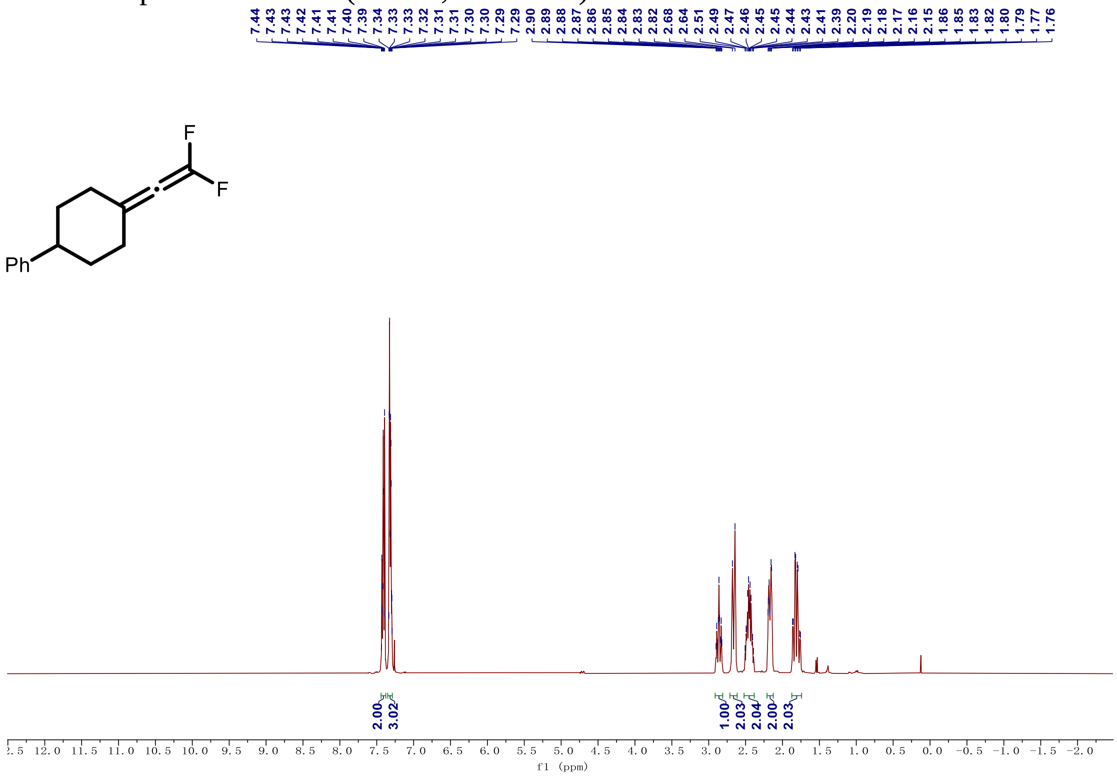

^{19}F NMR Spectrum of **1ab** ($CDCl_3$, 376 MHz)

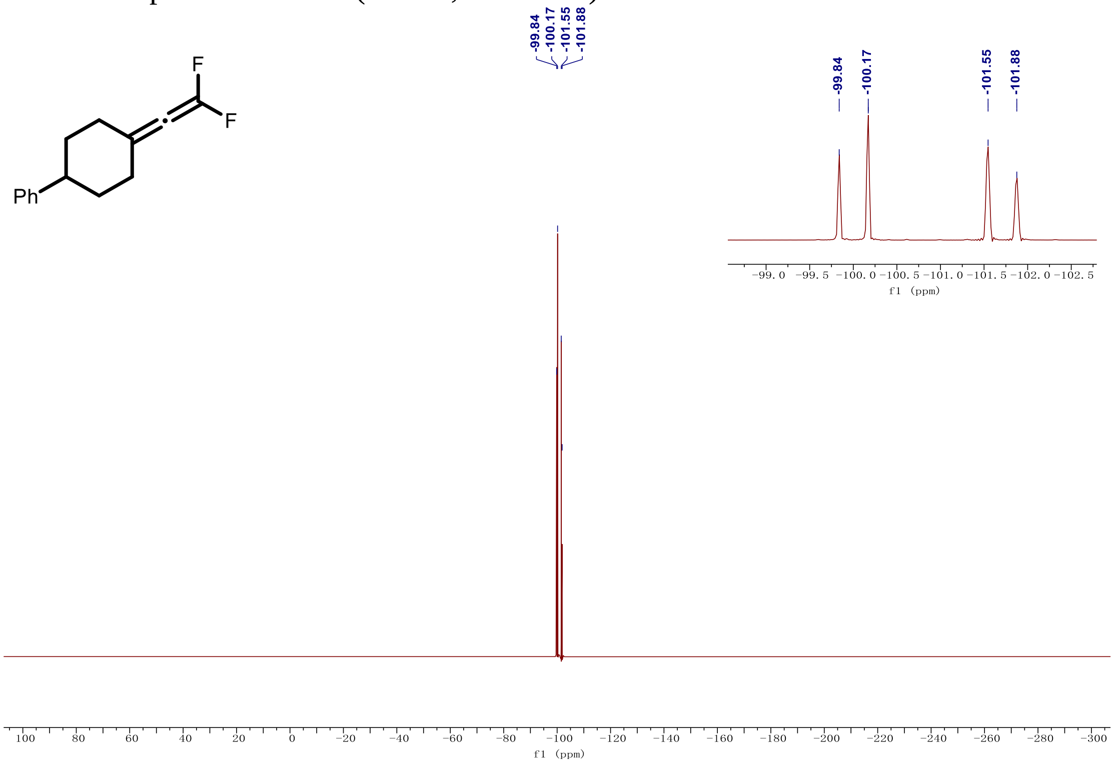

^{13}C NMR Spectrum of **1ab** ($CDCl_3$, 101 MHz)

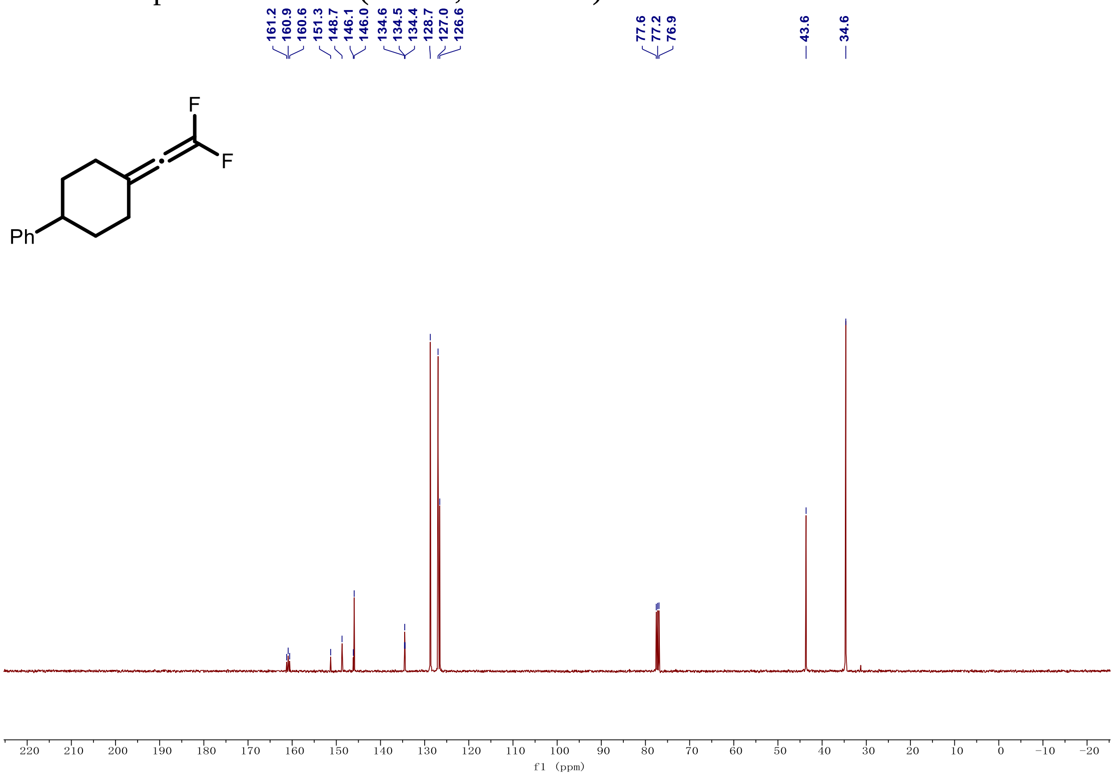

^{1}H NMR Spectrum of **1ac** ($CDCl_3$, 400 MHz)

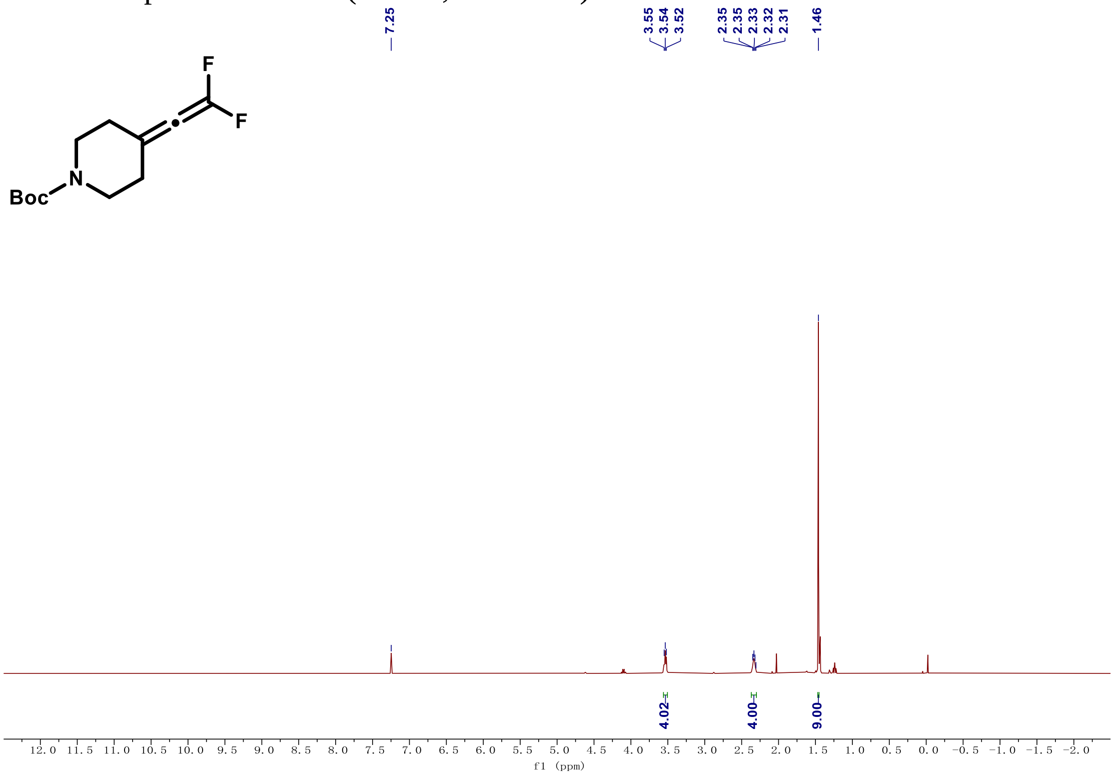

^{19}F NMR Spectrum of **1ac** ($CDCl_3$, 376 MHz)

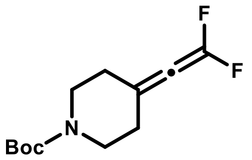

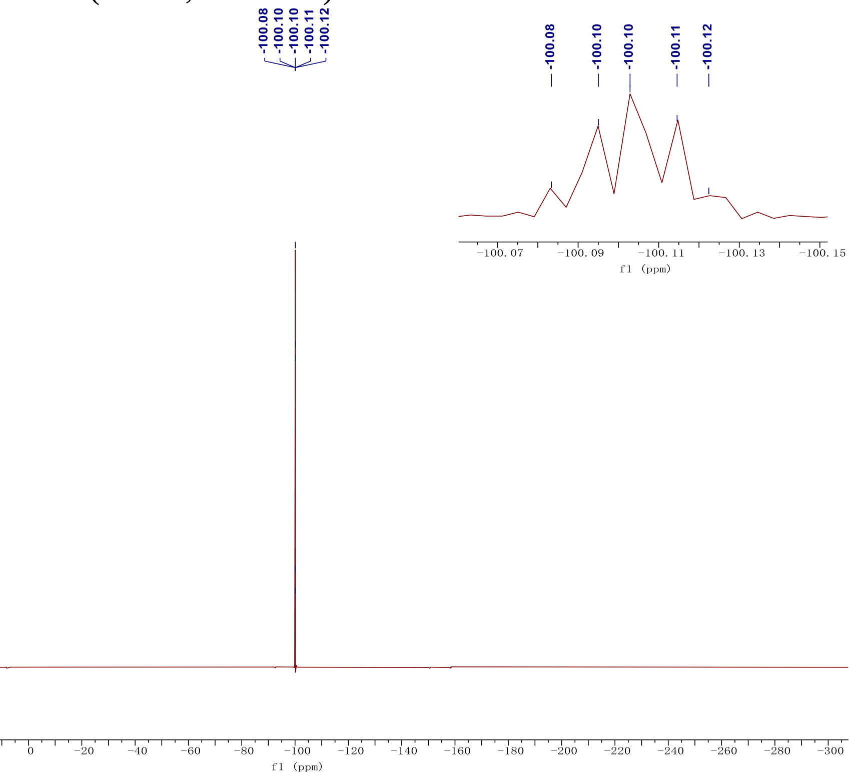

^{13}C NMR Spectrum of **1ac** ($CDCl_3$, 101 MHz)

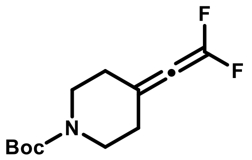

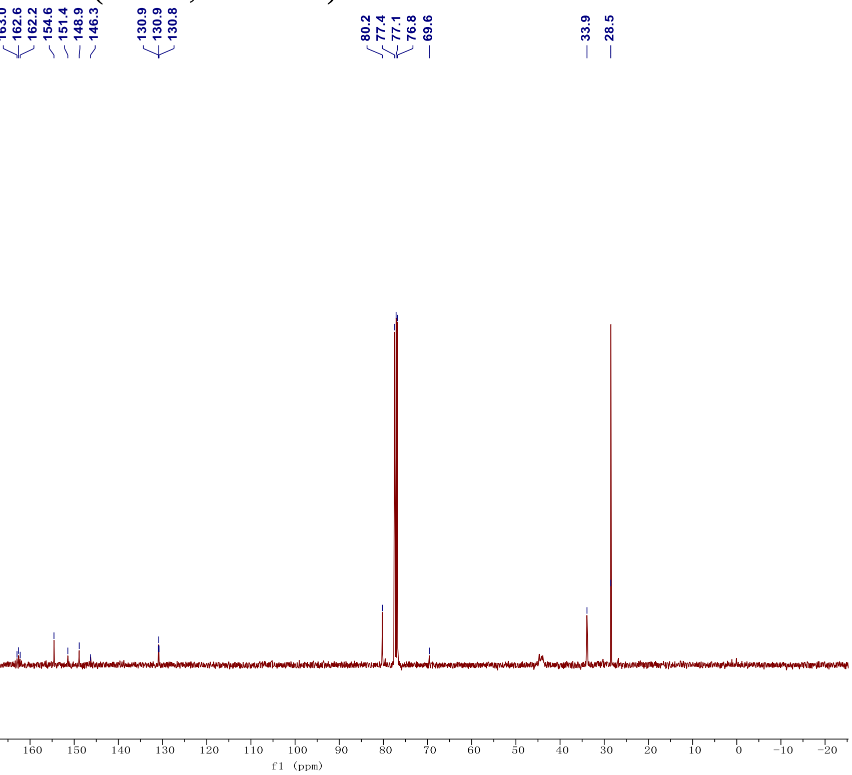

^{1}H NMR Spectrum of **1ad** ($CDCl_3$, 400 MHz)

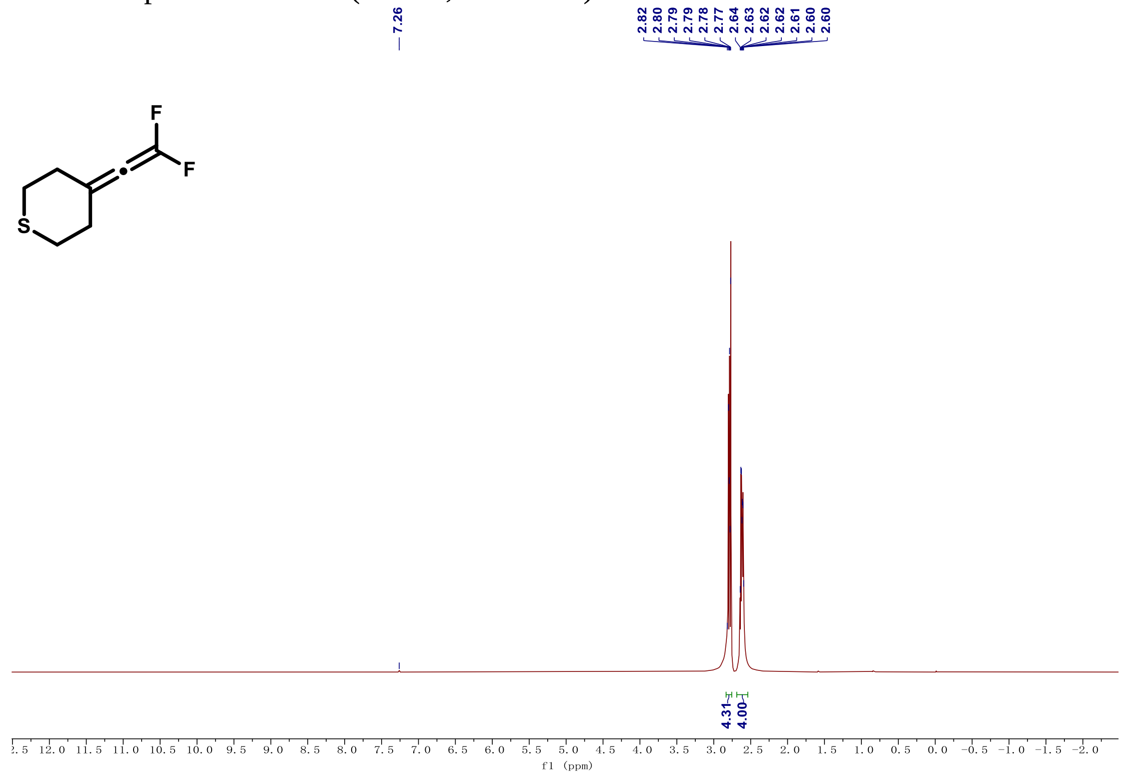

^{19}F NMR Spectrum of **1ad** ($CDCl_3$, 376 MHz)

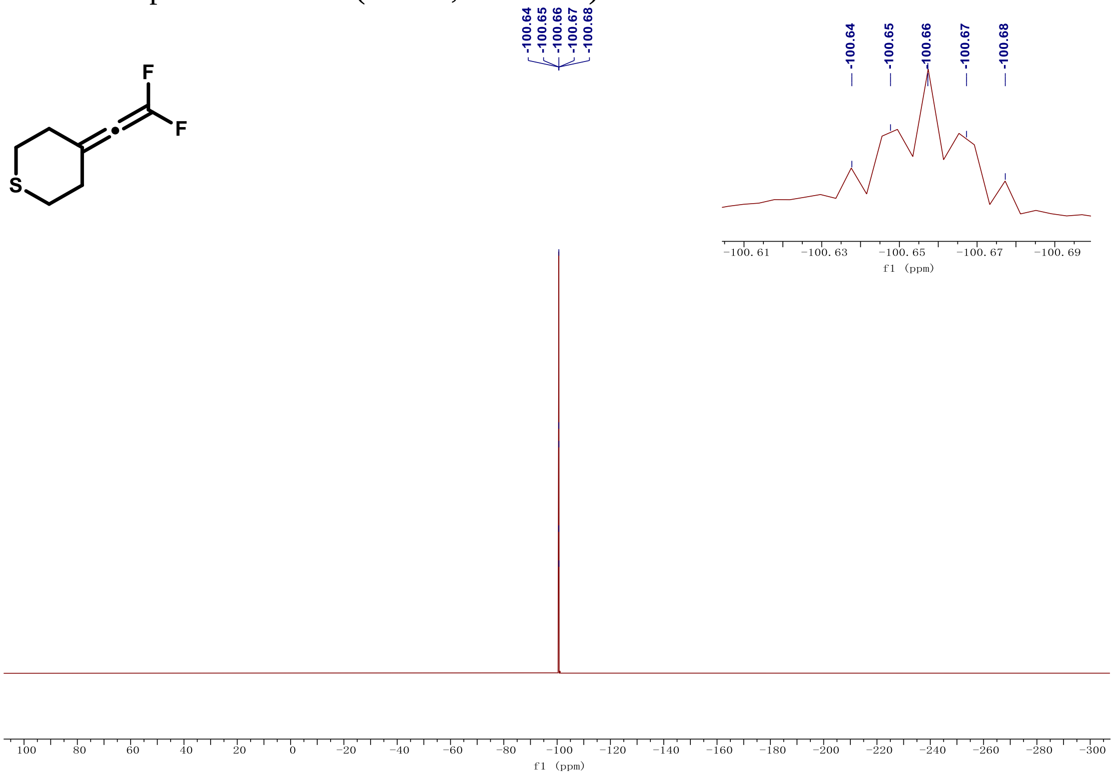

^{13}C NMR Spectrum of **1ad** ($CDCl_3$, 101 MHz)

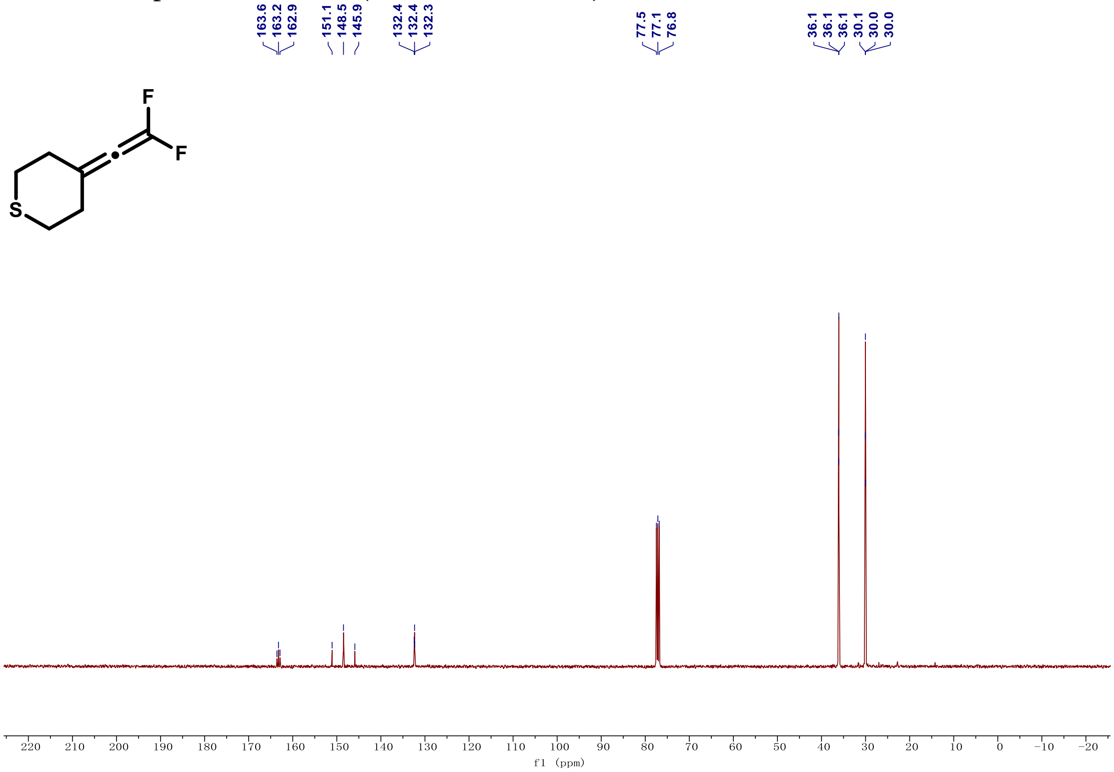

^{1}H NMR Spectrum of **1ae** ($CDCl_3$, 400 MHz)

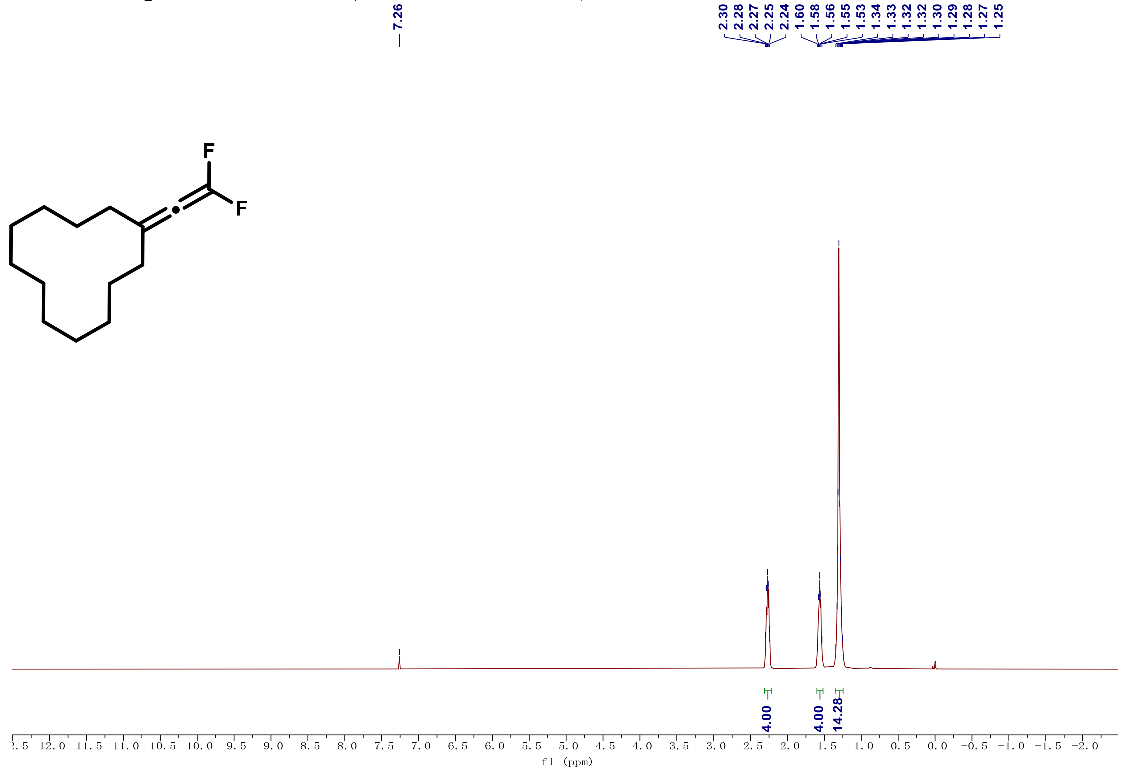

^{19}F NMR Spectrum of **1ae** ($CDCl_3$, 376 MHz)

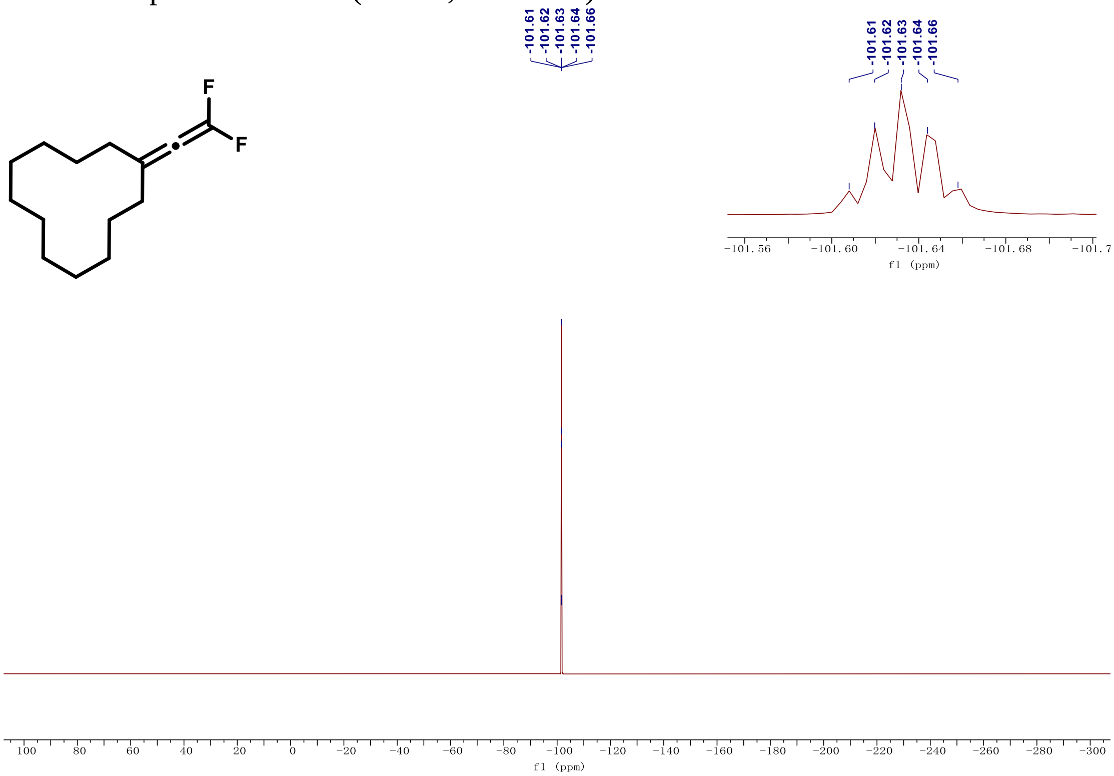

^{13}C NMR Spectrum of **1ae** ($CDCl_3$, 101 MHz)

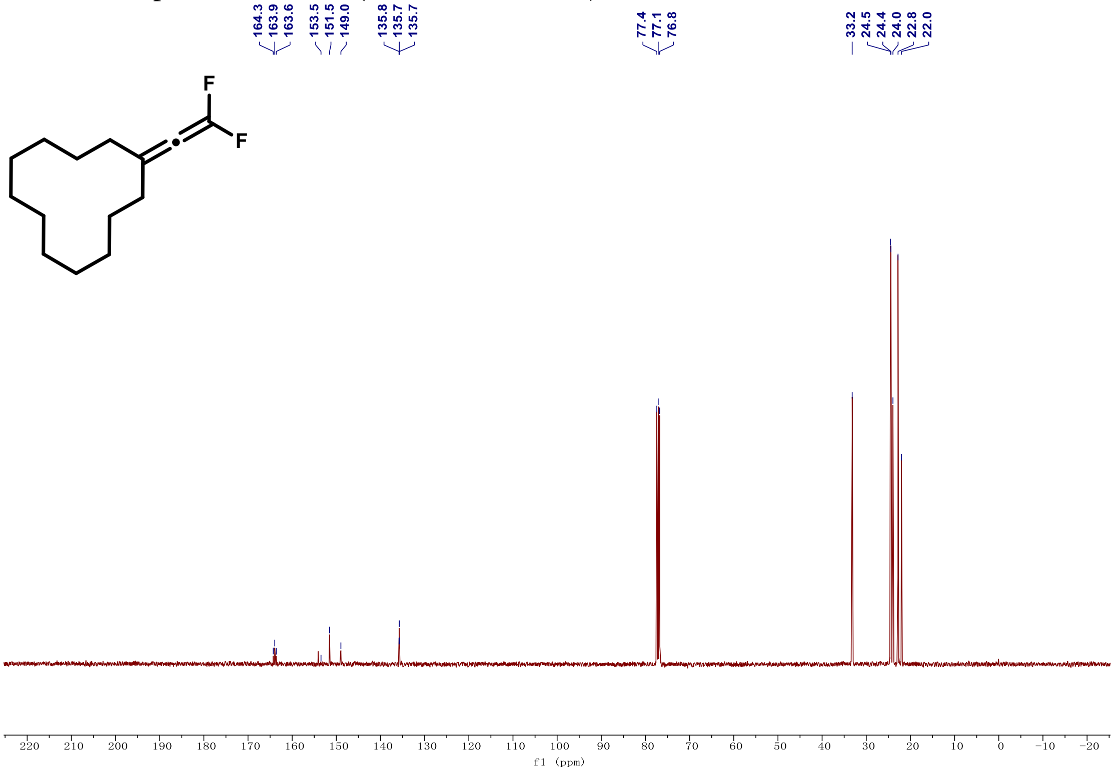

^{1}H NMR Spectrum of **1af** ($CDCl_3$, 400 MHz)

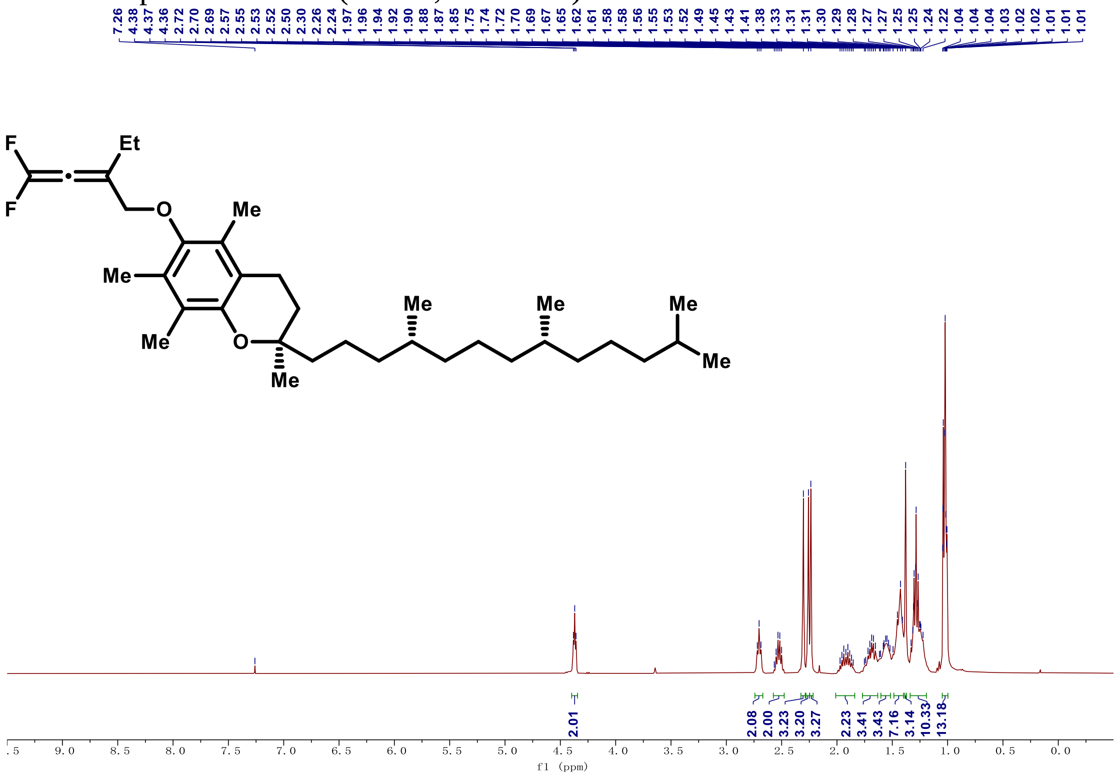

^{19}F NMR Spectrum of **1af** ($CDCl_3$, 376 MHz)

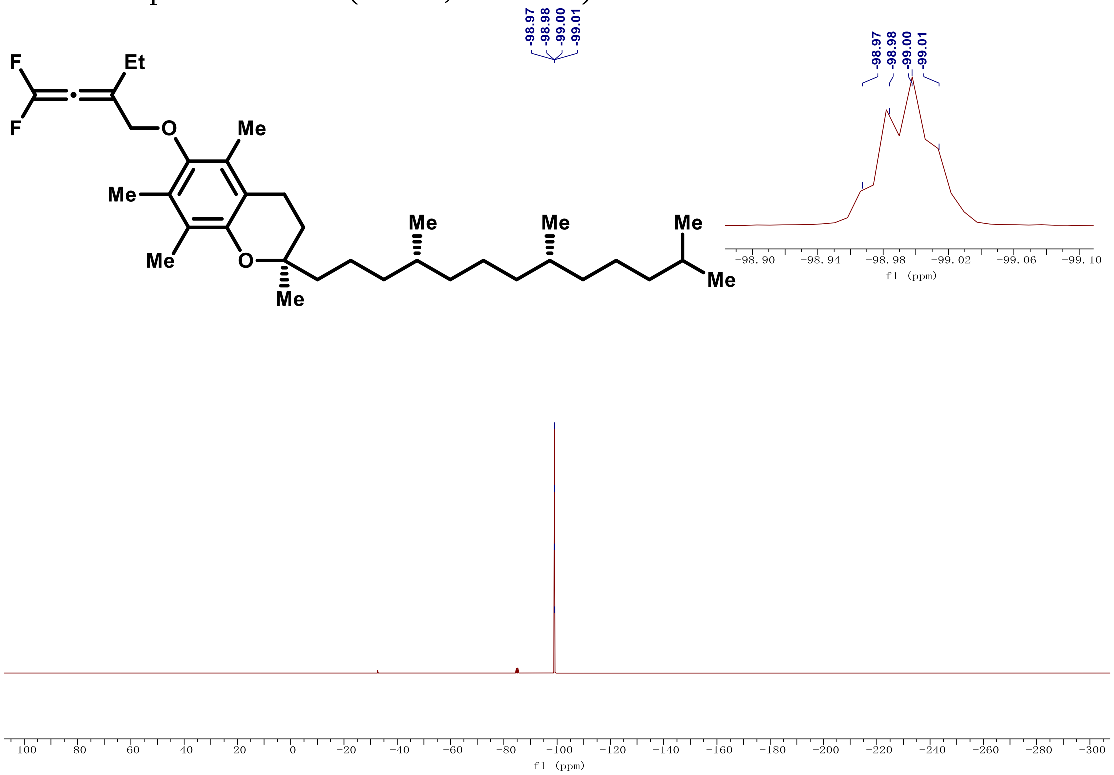

^{13}C NMR Spectrum of **1af** ($CDCl_3$, 101 MHz)

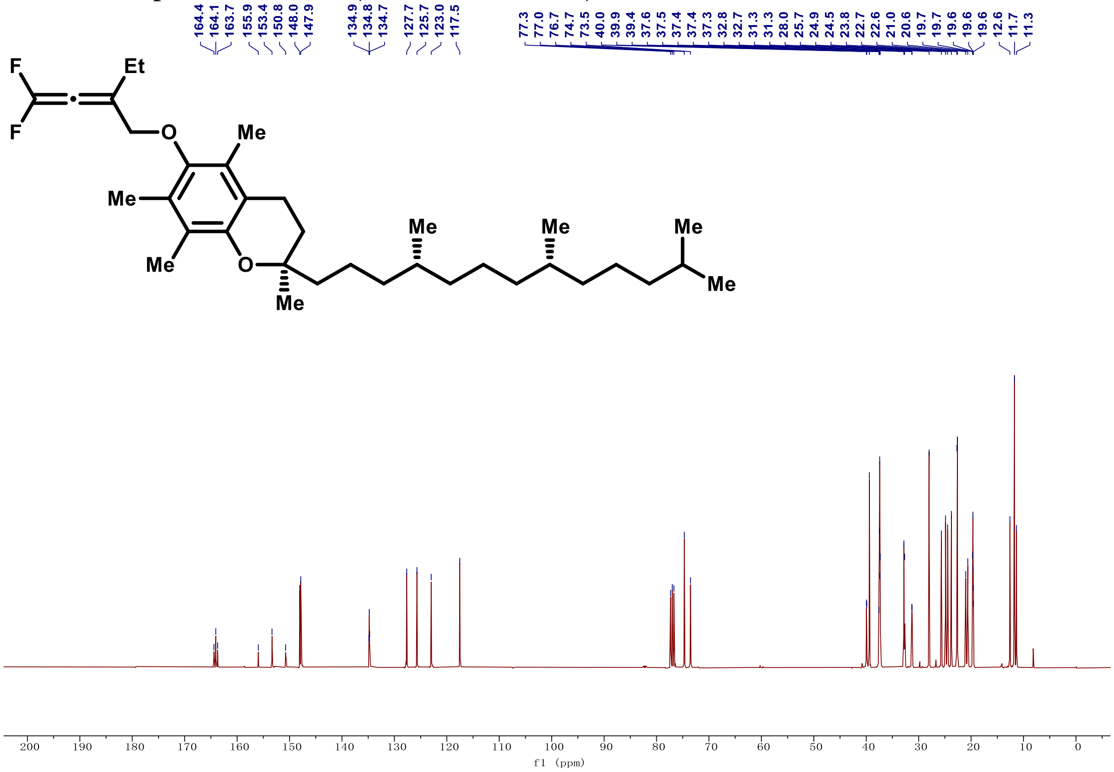

^{1}H NMR Spectrum of **1ag** ($CDCl_3$, 400 MHz)

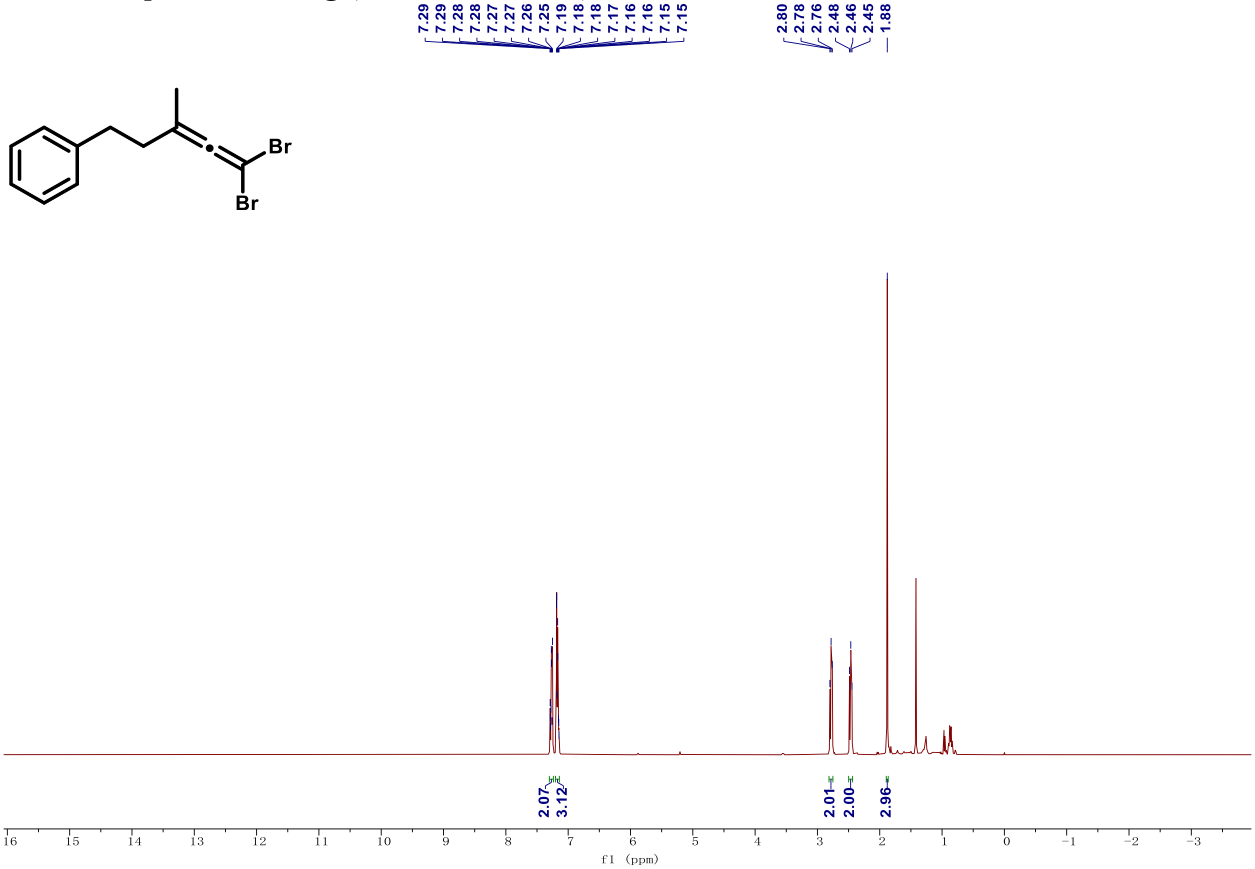

^{13}C NMR Spectrum of **1ag** ($CDCl_3$, 101 MHz)

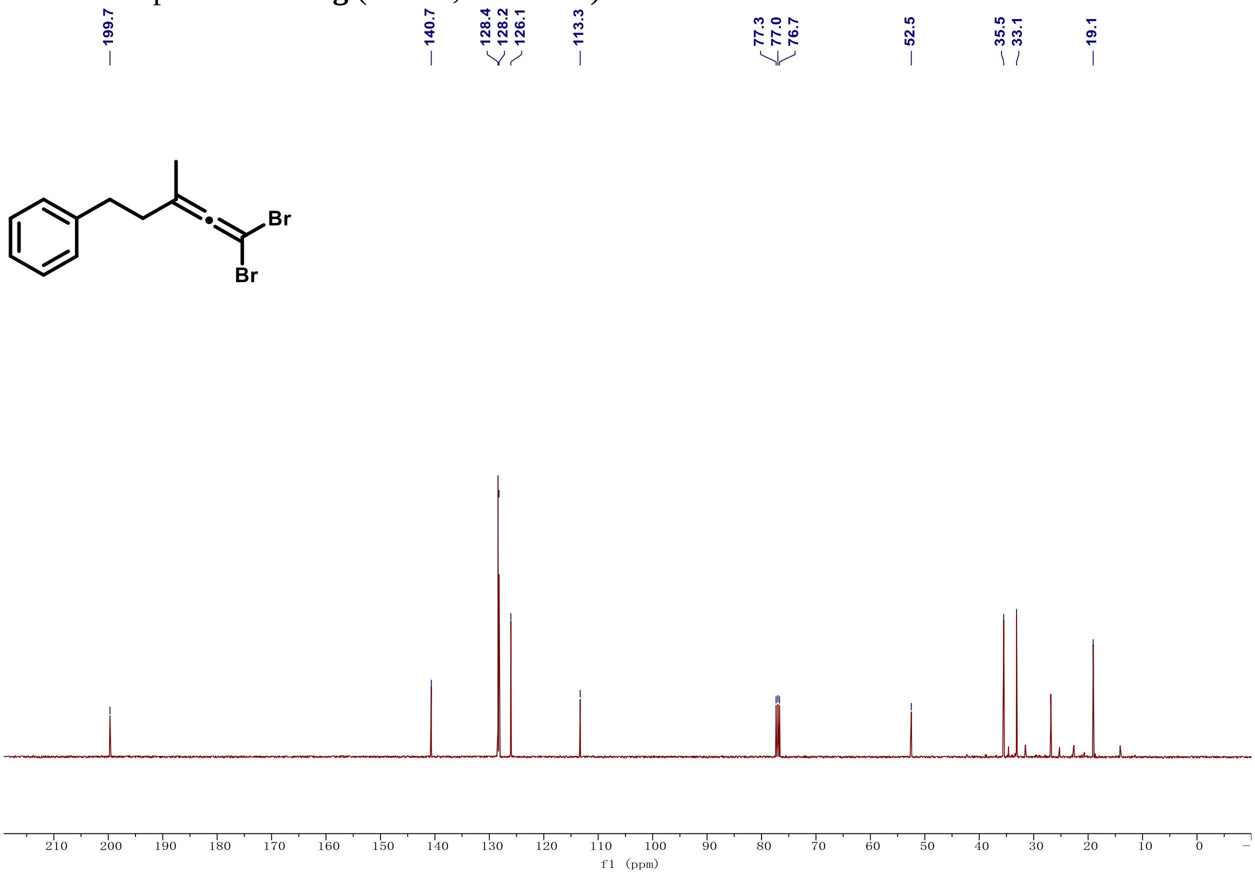

^{1}H NMR Spectrum of **3aa** ($CDCl_3$, 400 MHz)

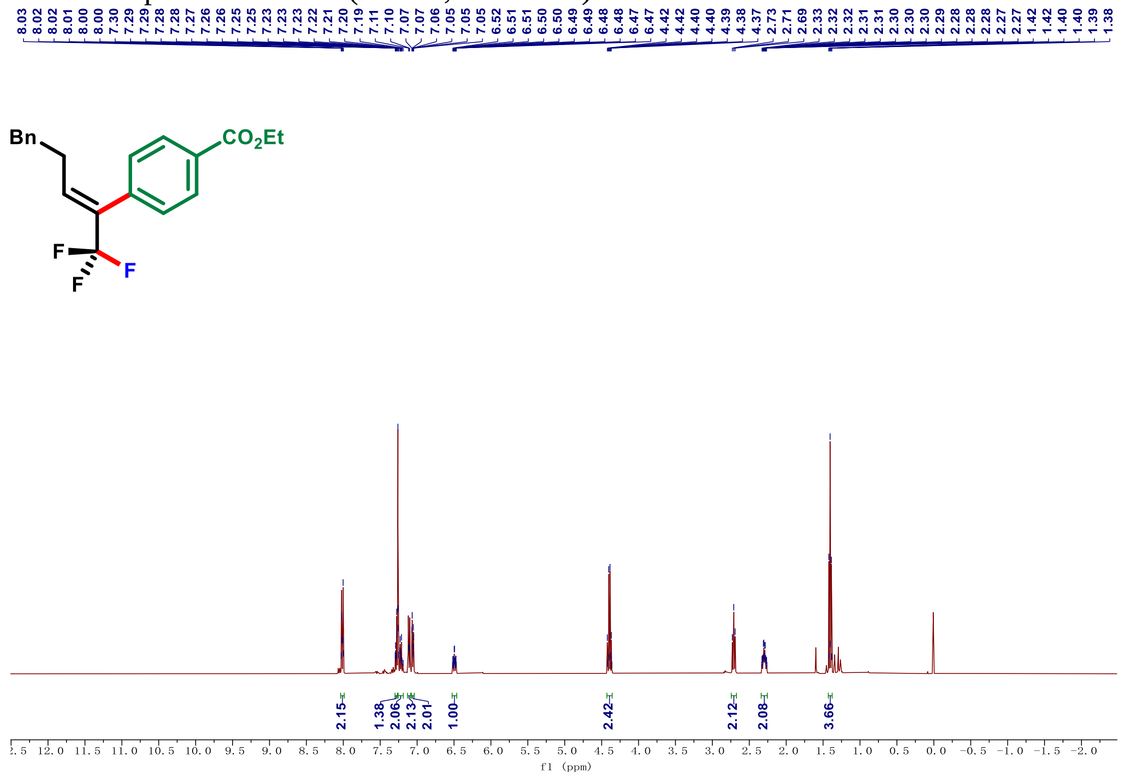

^{19}F NMR Spectrum of **3aa** ($CDCl_3$, 376 MHz)

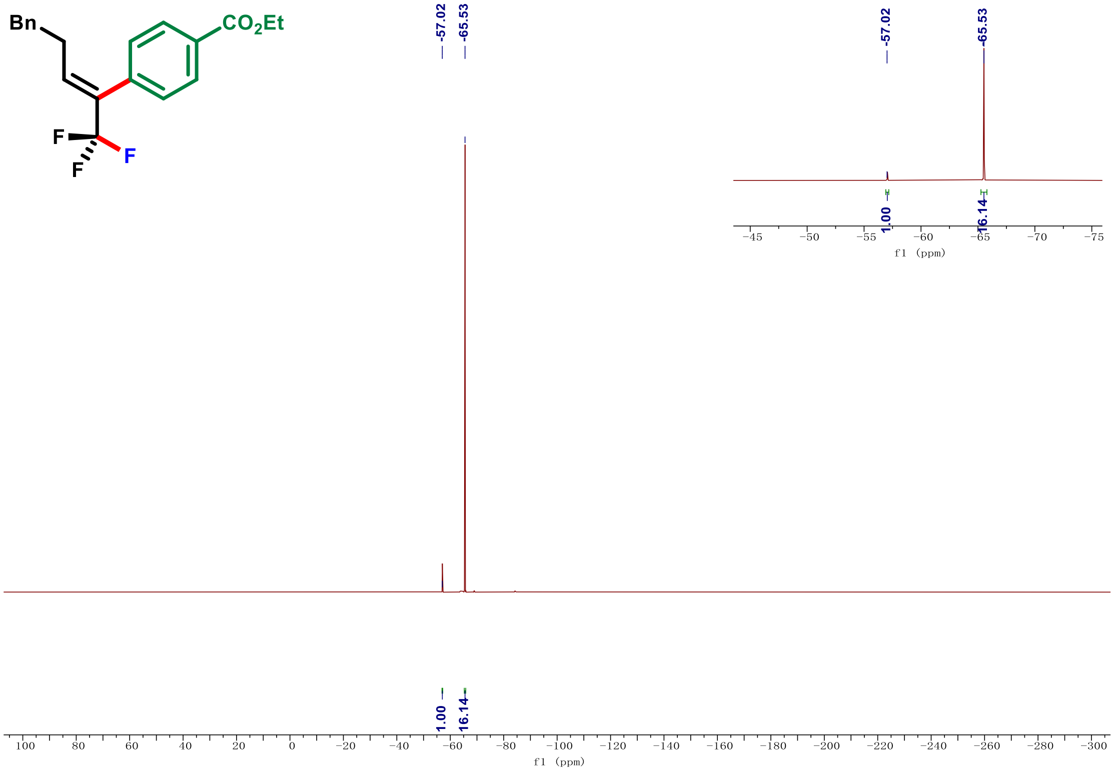

^{13}C NMR Spectrum of **3aa** ($CDCl_3$, 101 MHz)

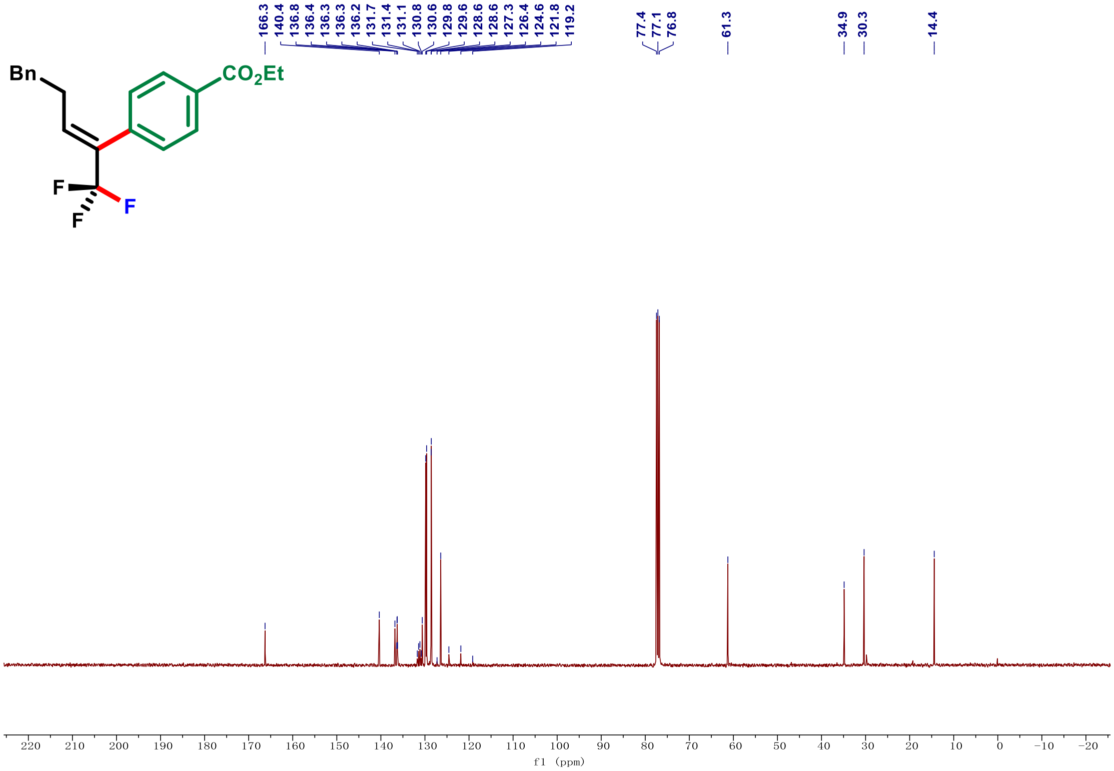

^{1}H NMR Spectrum of **3ba** ($CDCl_3$, 400 MHz)

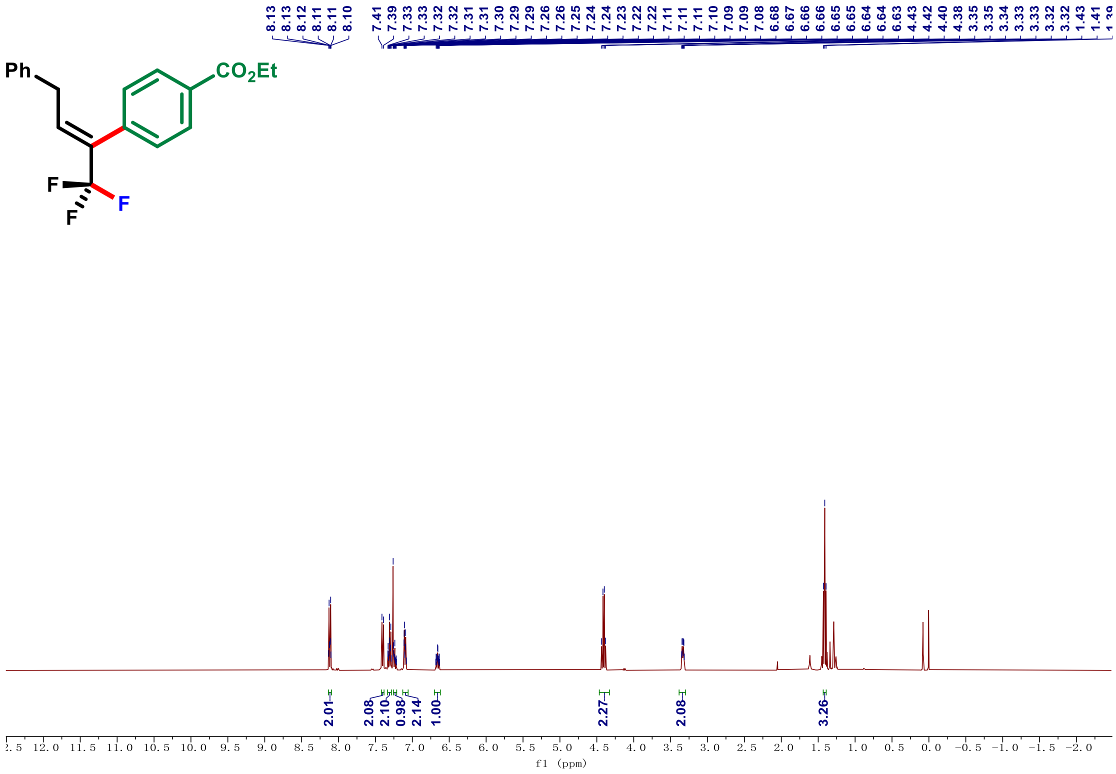

^{19}F NMR Spectrum of **3ba** ($CDCl_3$, 376 MHz)

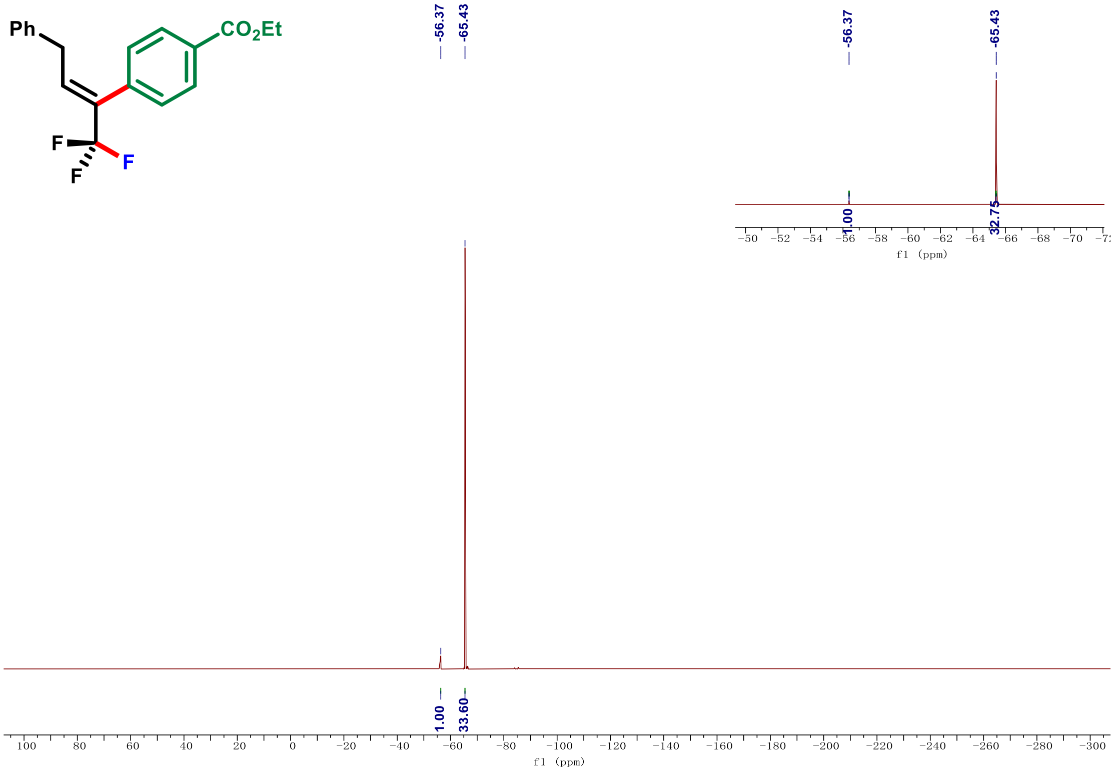

^{13}C NMR Spectrum of **3ba** ($CDCl_3$, 101 MHz)

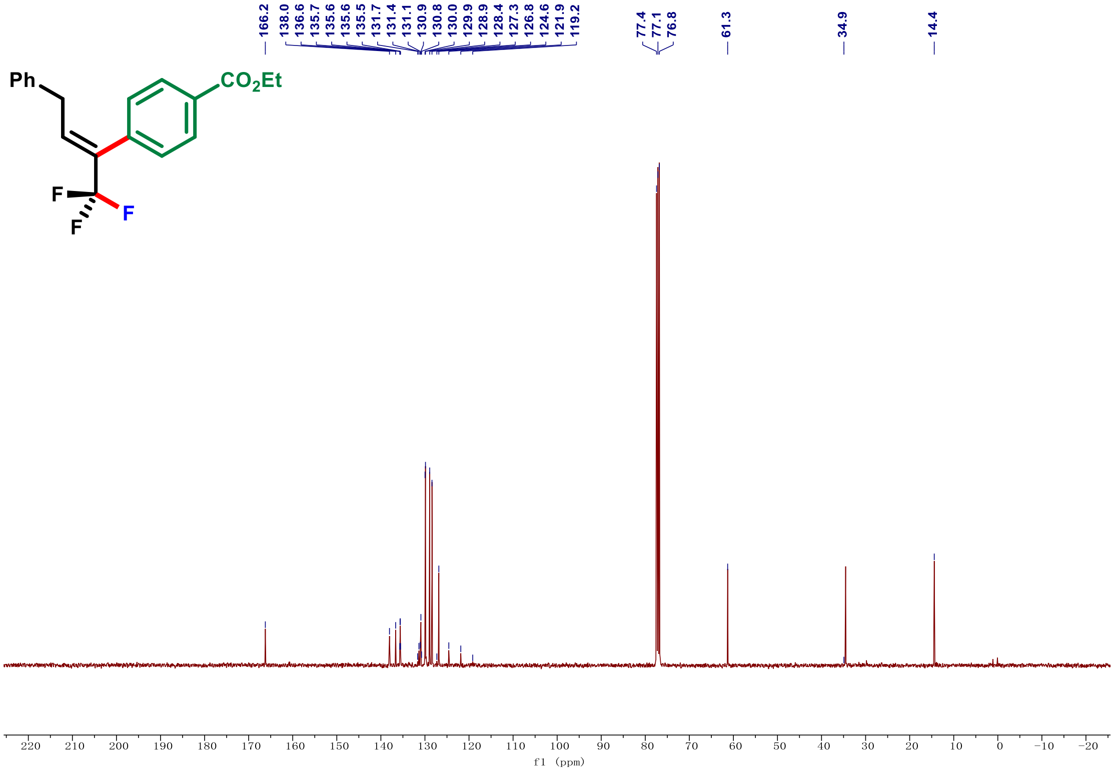

^{1}H NMR Spectrum of **3ca** ($CDCl_3$, 400 MHz)

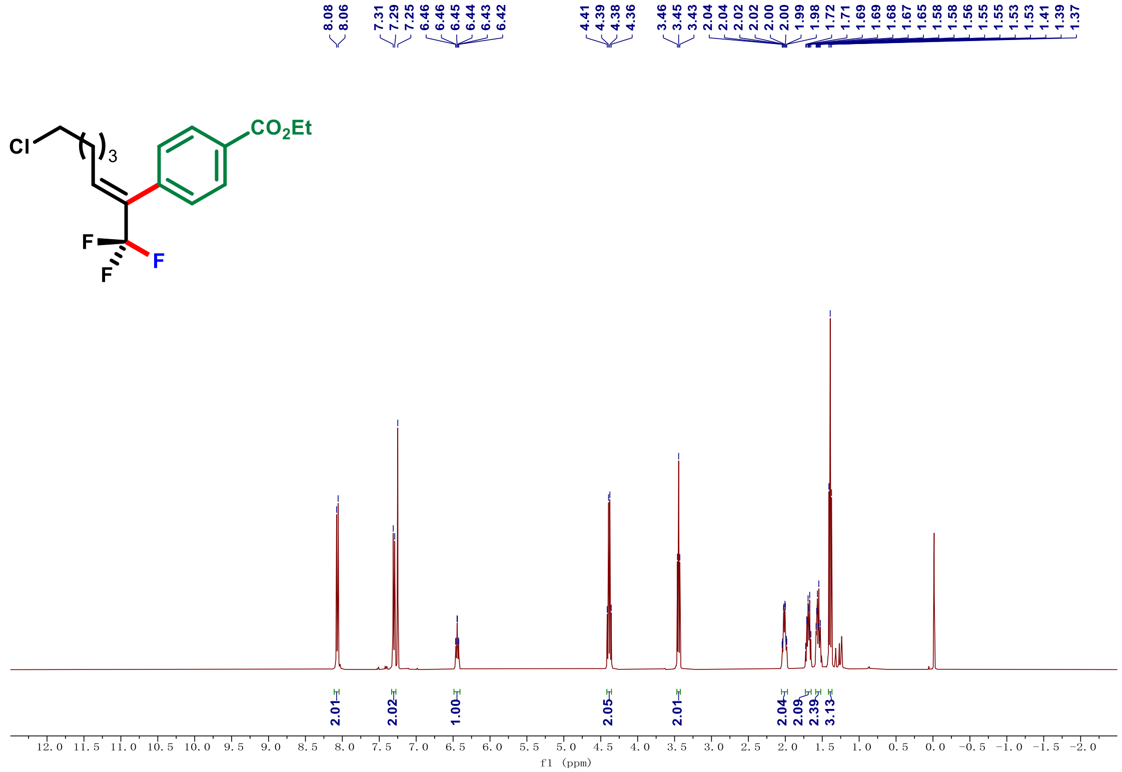

^{19}F NMR Spectrum of **3ca** ($CDCl_3$, 376 MHz)

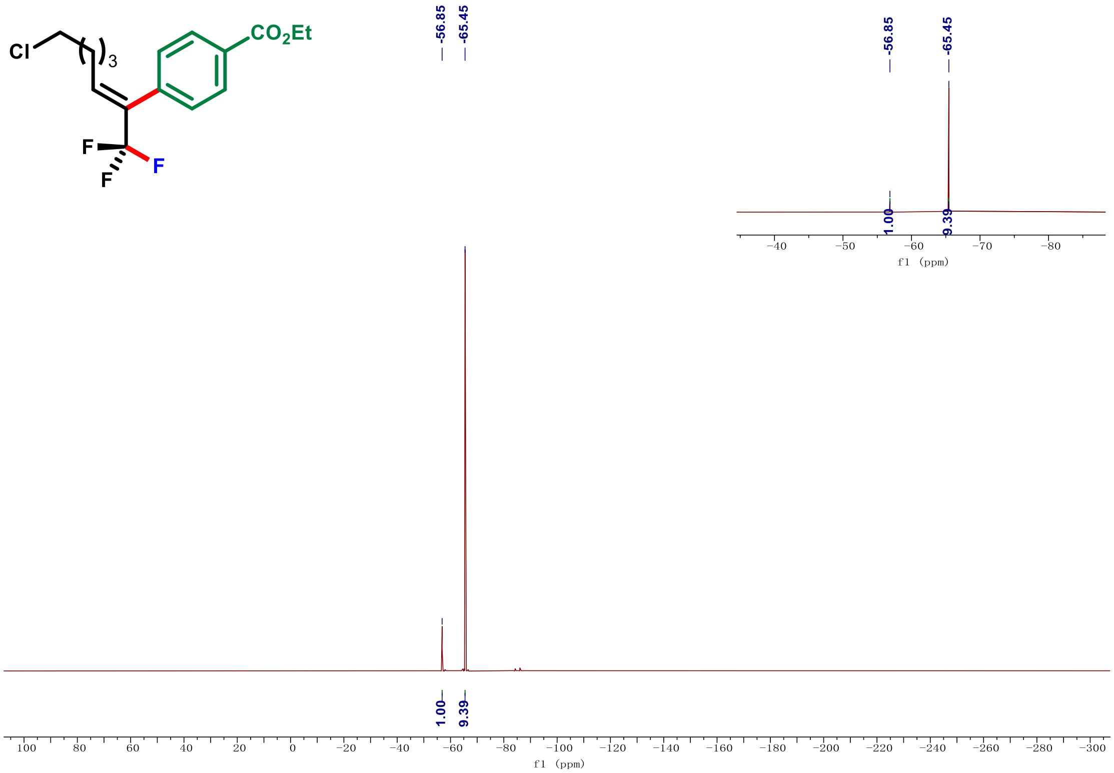

^{13}C NMR Spectrum of **3ca** ($CDCl_3$, 101 MHz)

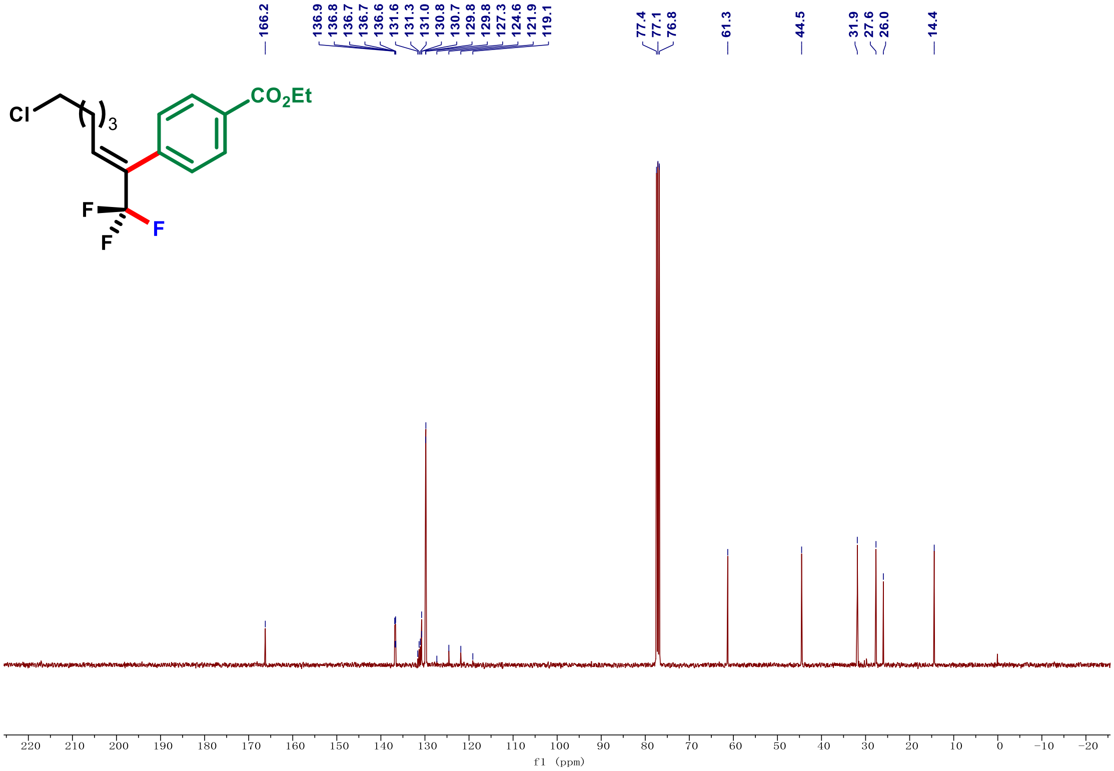

^{1}H NMR Spectrum of **3da** ($CDCl_3$, 400 MHz)

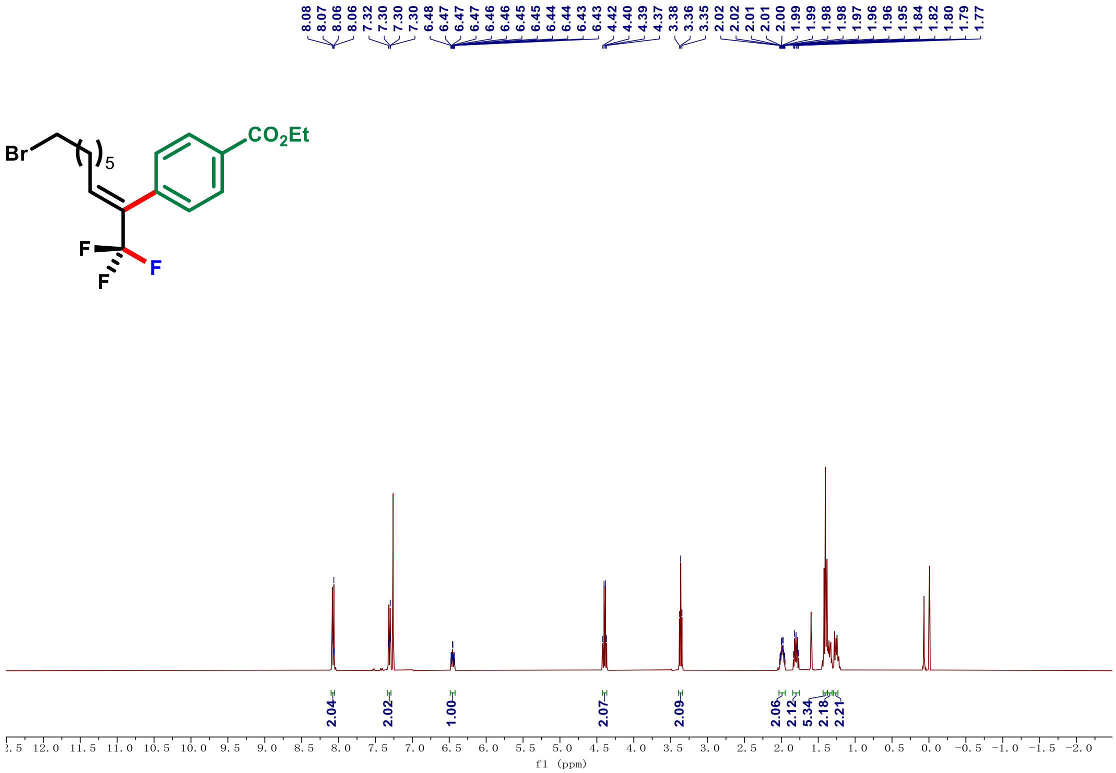

^{19}F NMR Spectrum of **3da** ($CDCl_3$, 376 MHz)

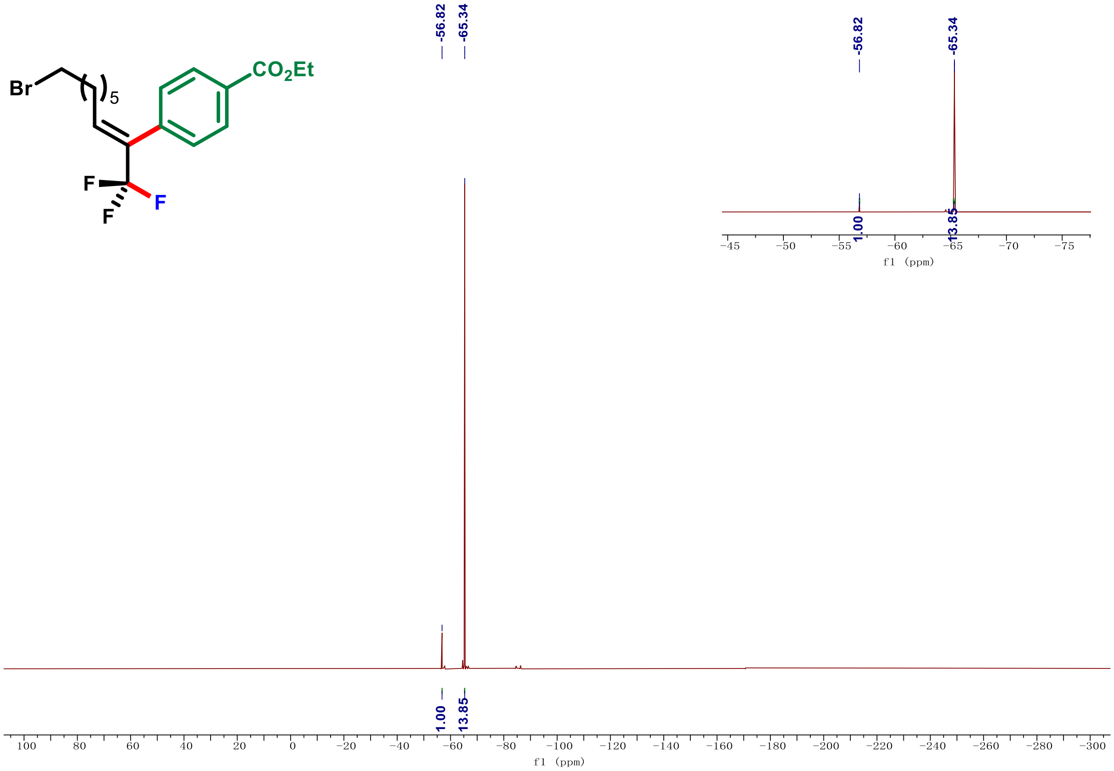

^{13}C NMR Spectrum of **3da** ($CDCl_3$, 101 MHz)

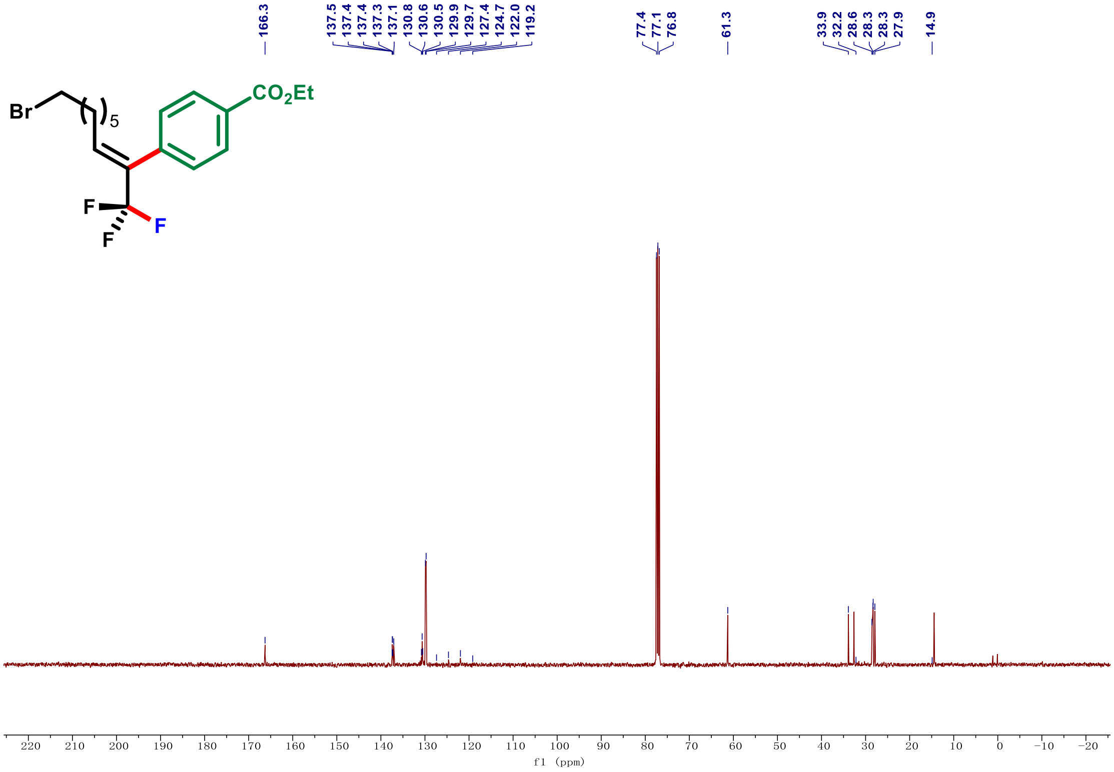

^{1}H NMR Spectrum of **3ea** ($CDCl_3$, 400 MHz)

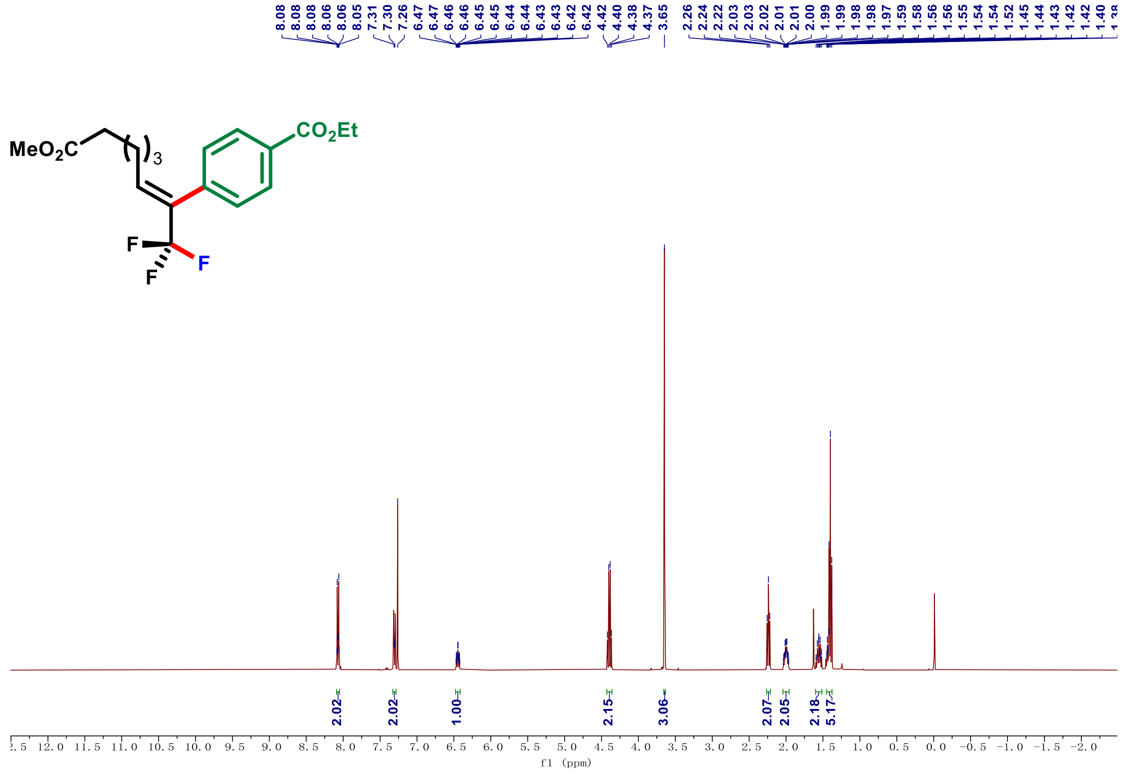

^{19}F NMR Spectrum of **3ea** ($CDCl_3$, 376 MHz)

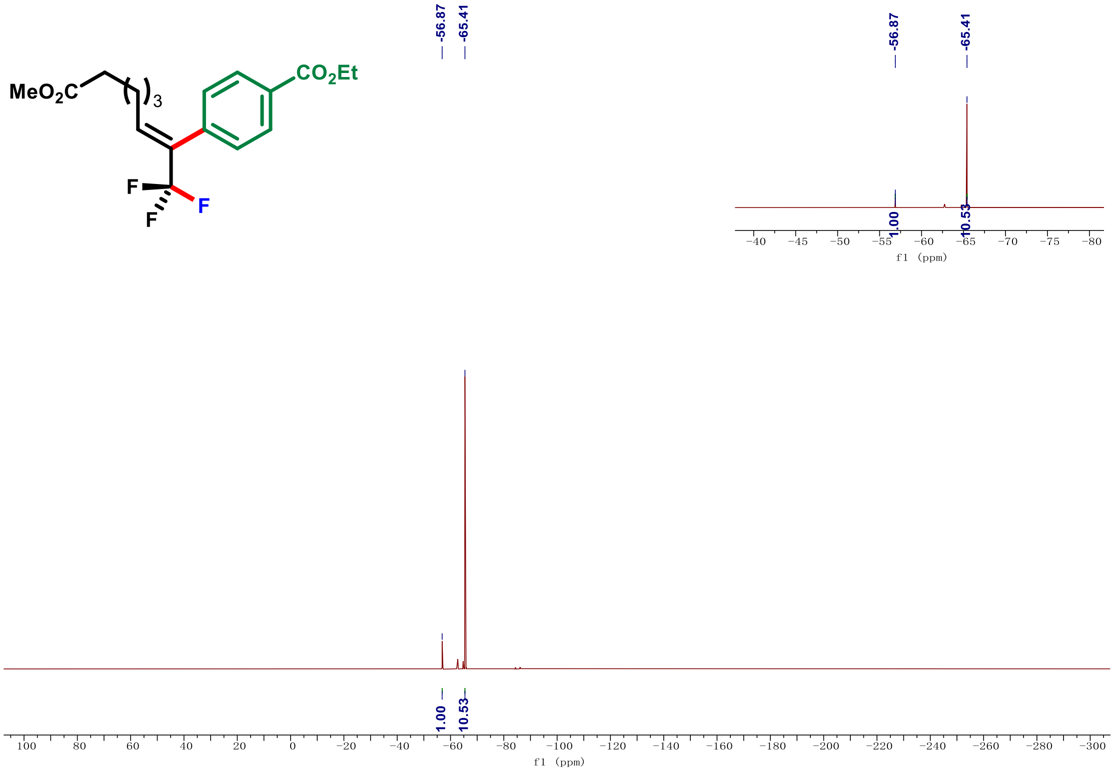

^{13}C NMR Spectrum of **3ea** ($CDCl_3$, 101 MHz)

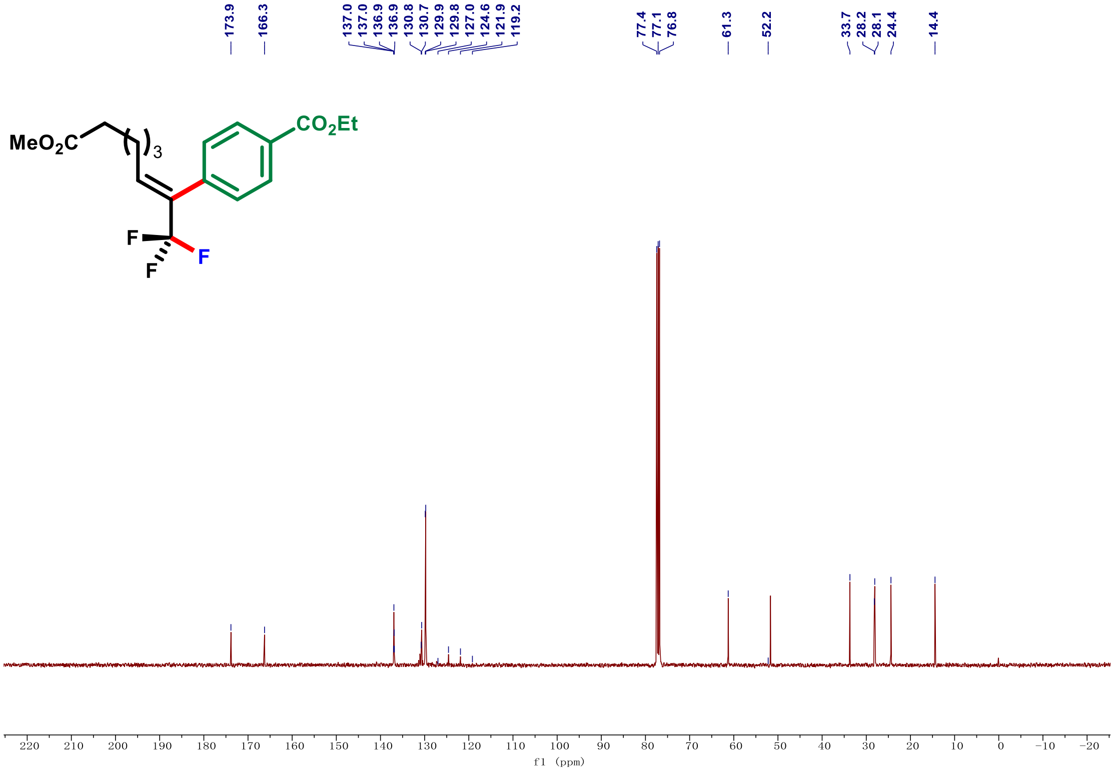

^{1}H NMR Spectrum of **3fa** ($CDCl_3$, 400 MHz)

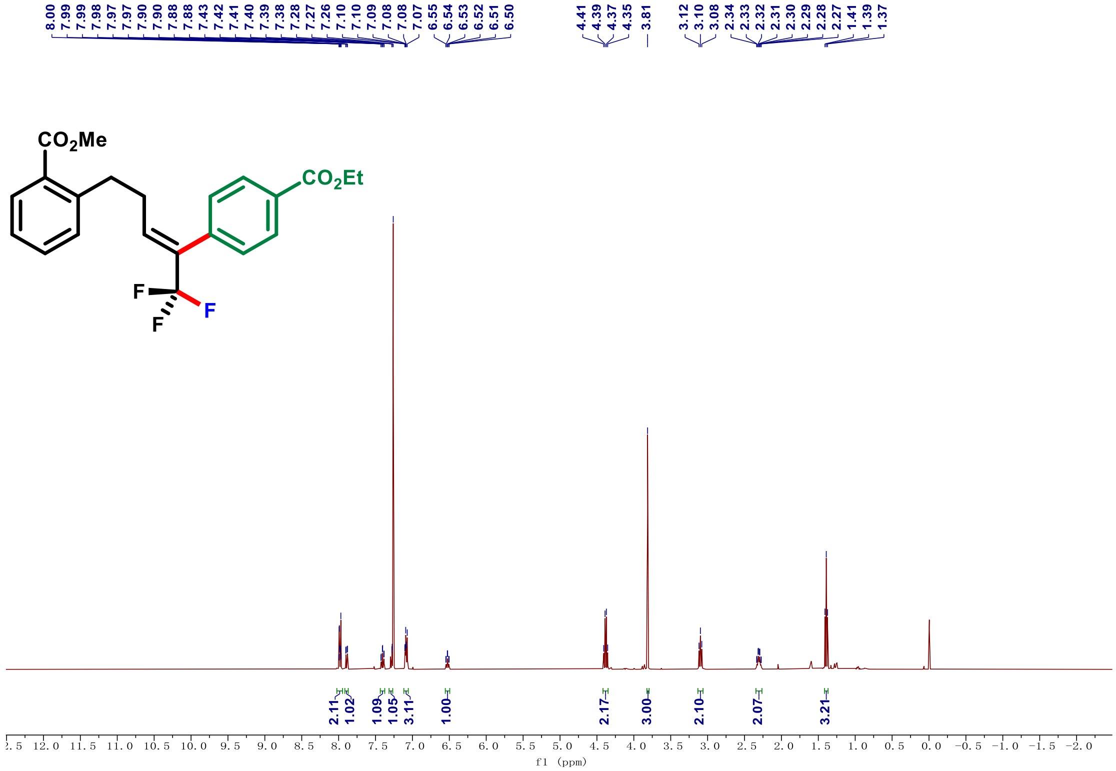

^{19}F NMR Spectrum of **3fa** ($CDCl_3$, 376 MHz)

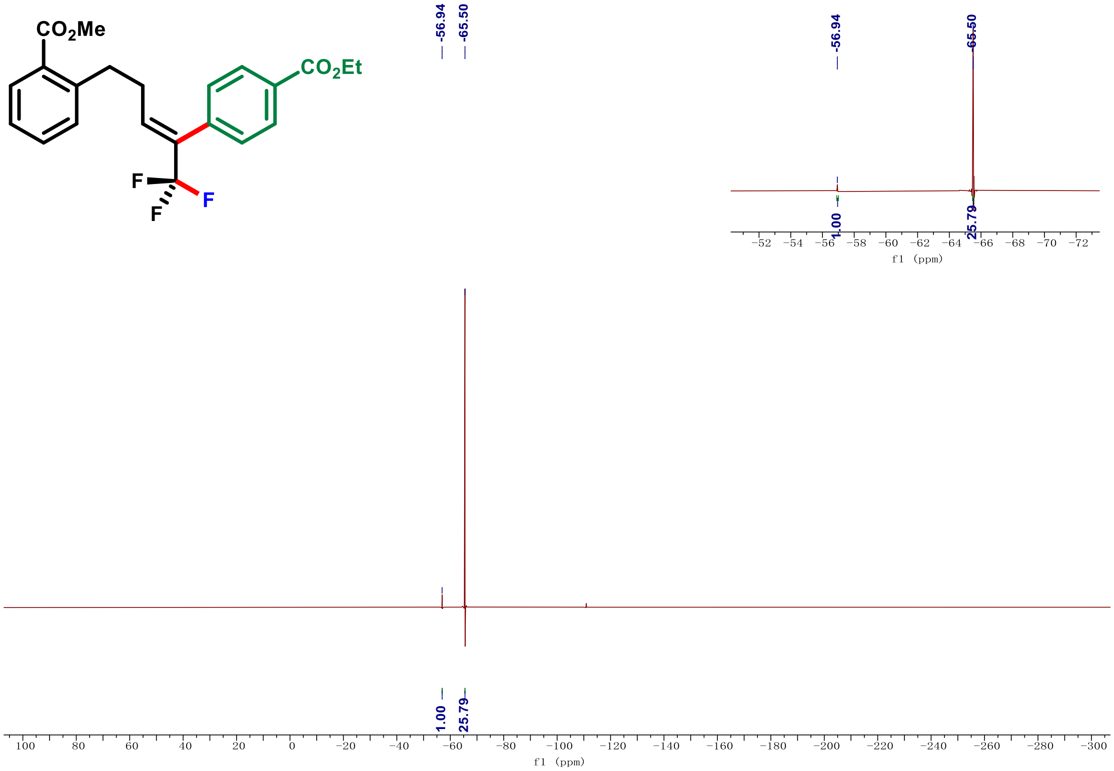

^{13}C NMR Spectrum of **3fa** ($CDCl_3$, 101 MHz)

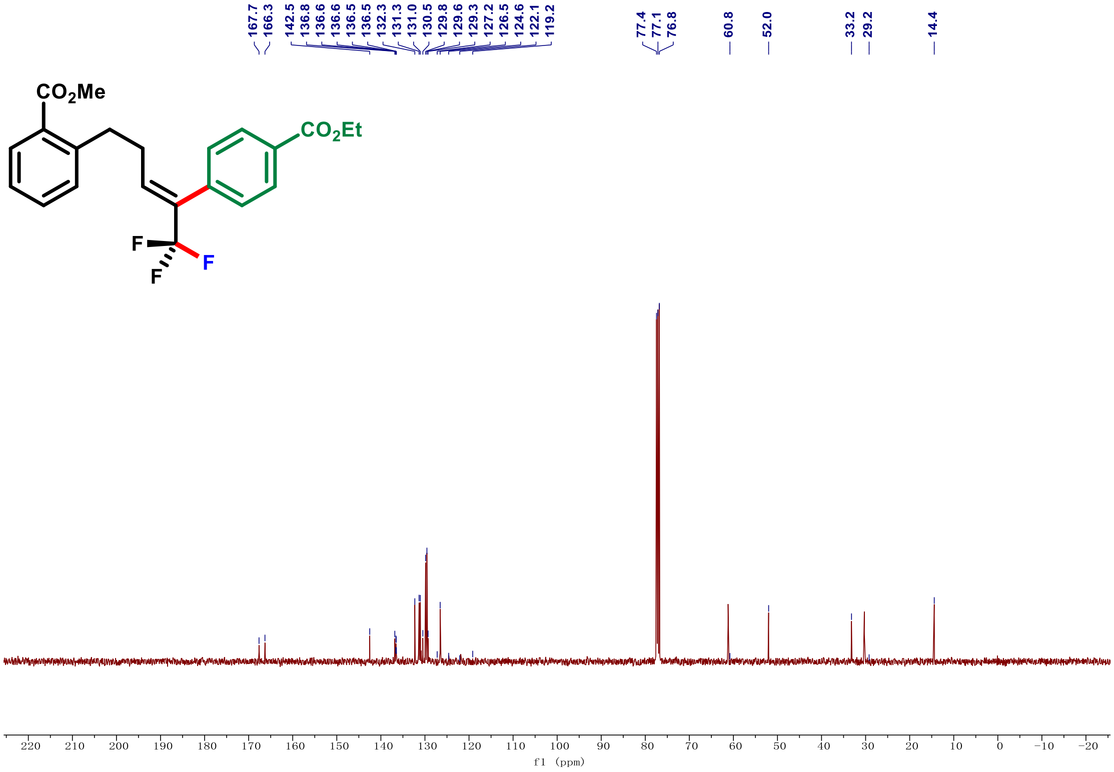

^{1}H NMR Spectrum of **3ga** ($CDCl_3$, 400 MHz)

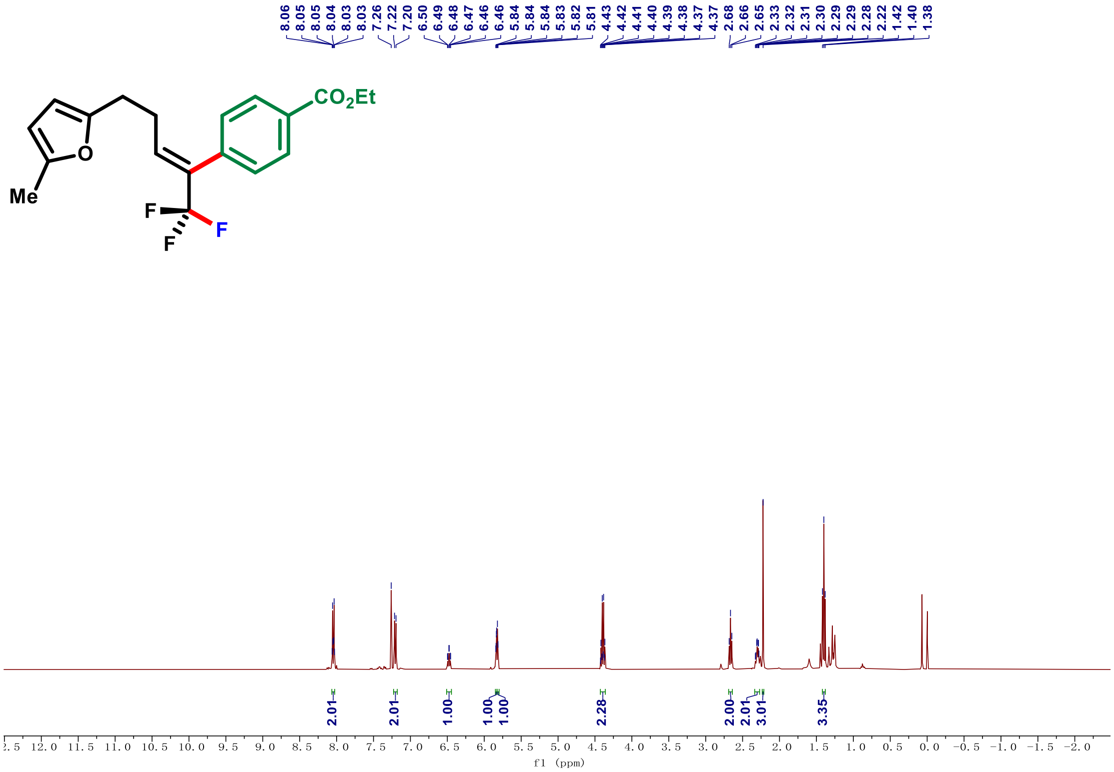

^{19}F NMR Spectrum of **3ga** ($CDCl_3$, 376 MHz)

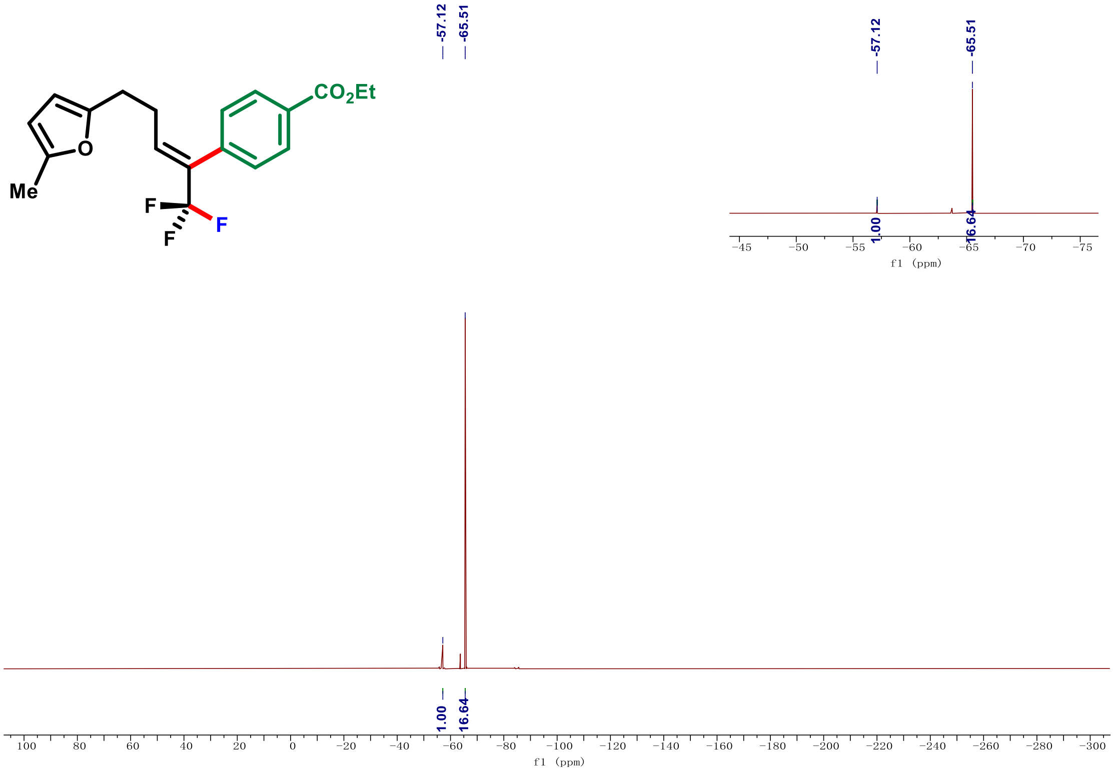

^{13}C NMR Spectrum of **3ga** ($CDCl_3$, 101 MHz)

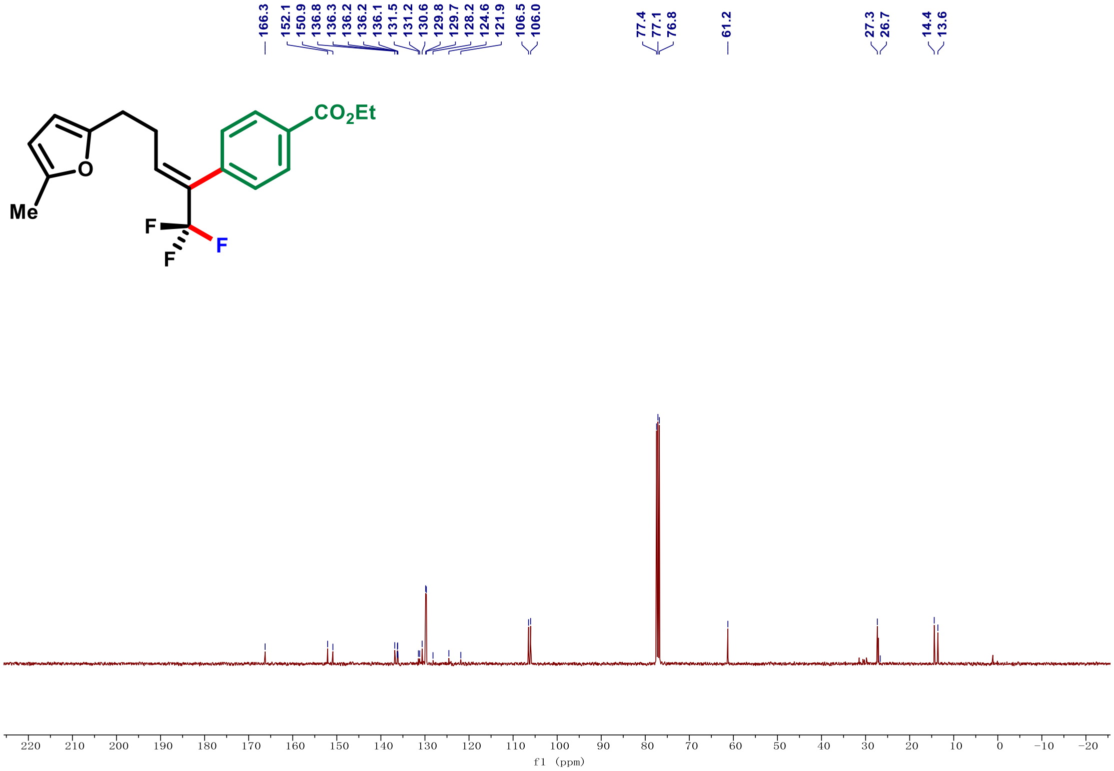

^{1}H NMR Spectrum of **3ha** ($CDCl_3$, 400 MHz)

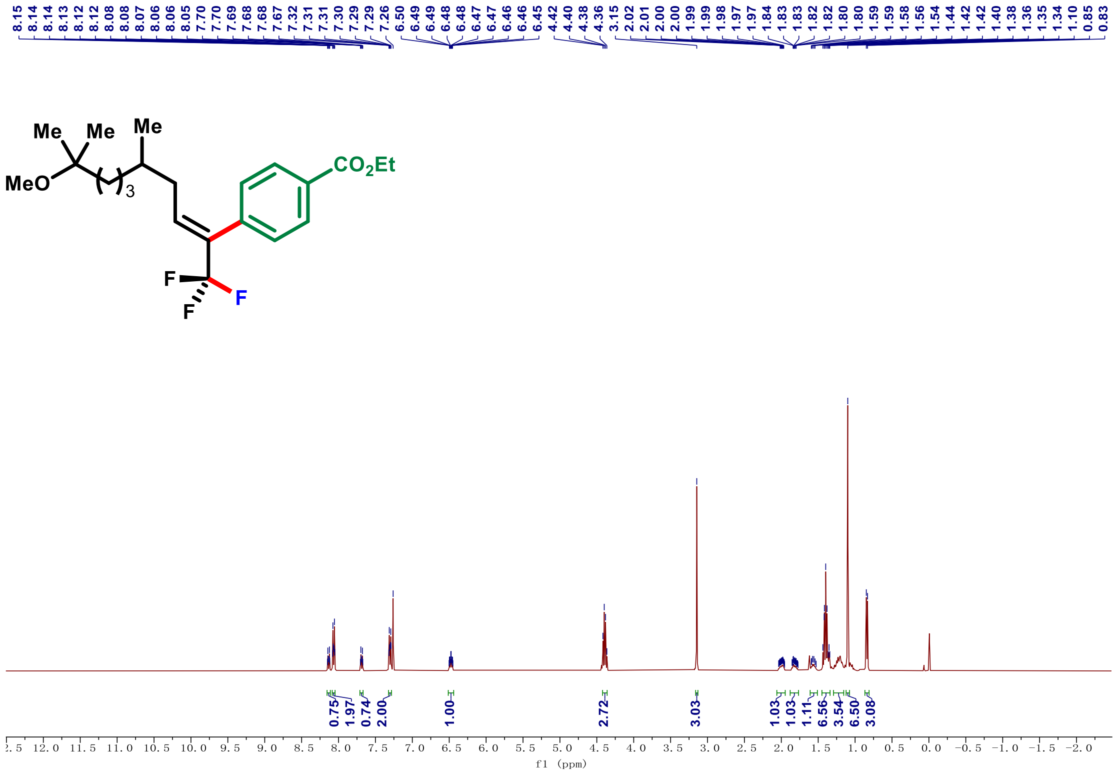

^{19}F NMR Spectrum of **3ha** ($CDCl_3$, 376 MHz)

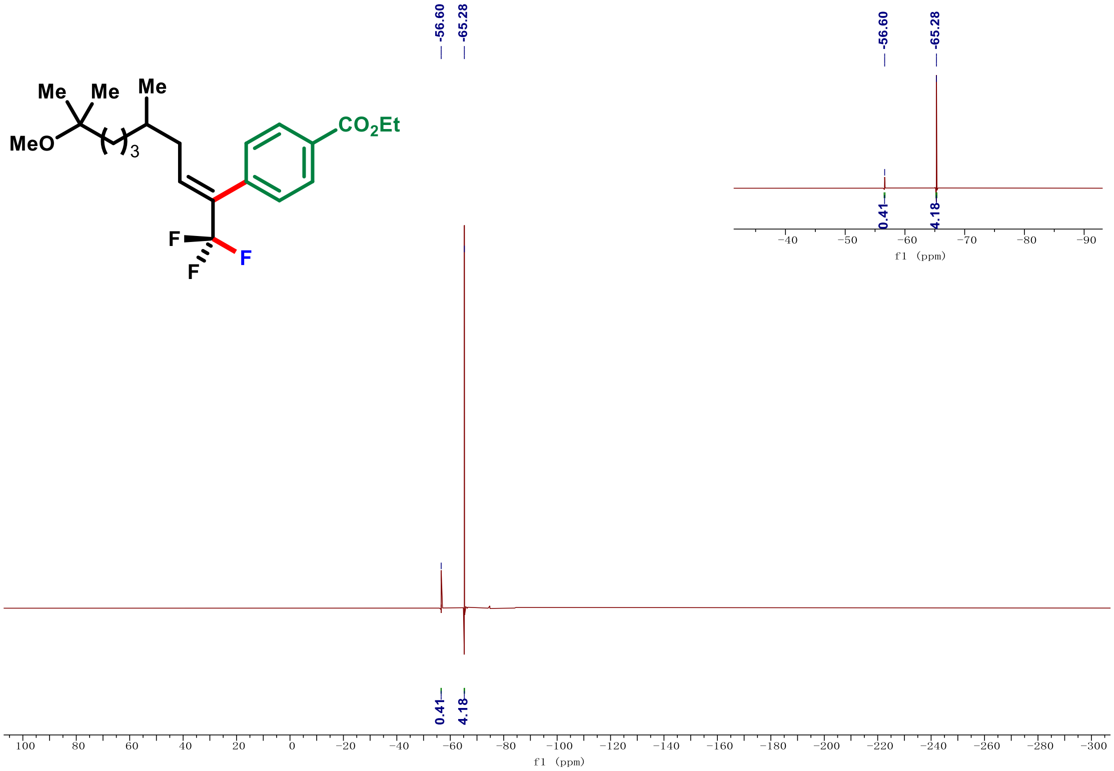

^{13}C NMR Spectrum of **3ha** ($CDCl_3$, 101 MHz)

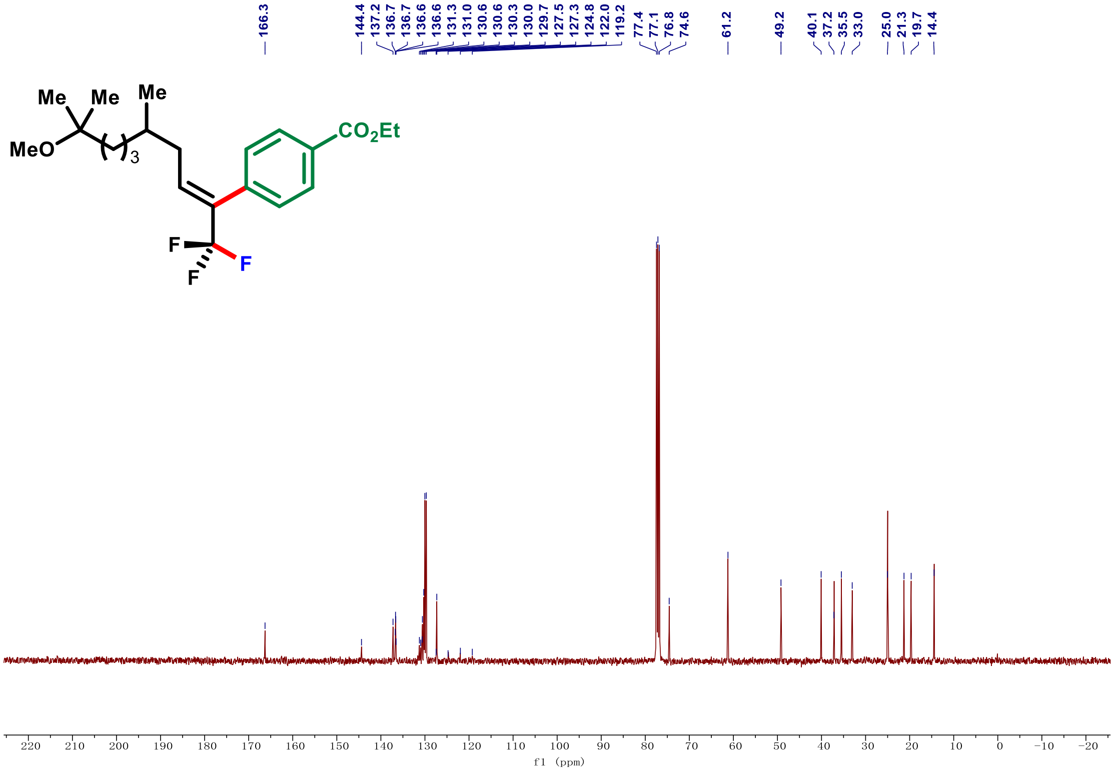

^{1}H NMR Spectrum of **3ia** ($CDCl_3$, 400 MHz)

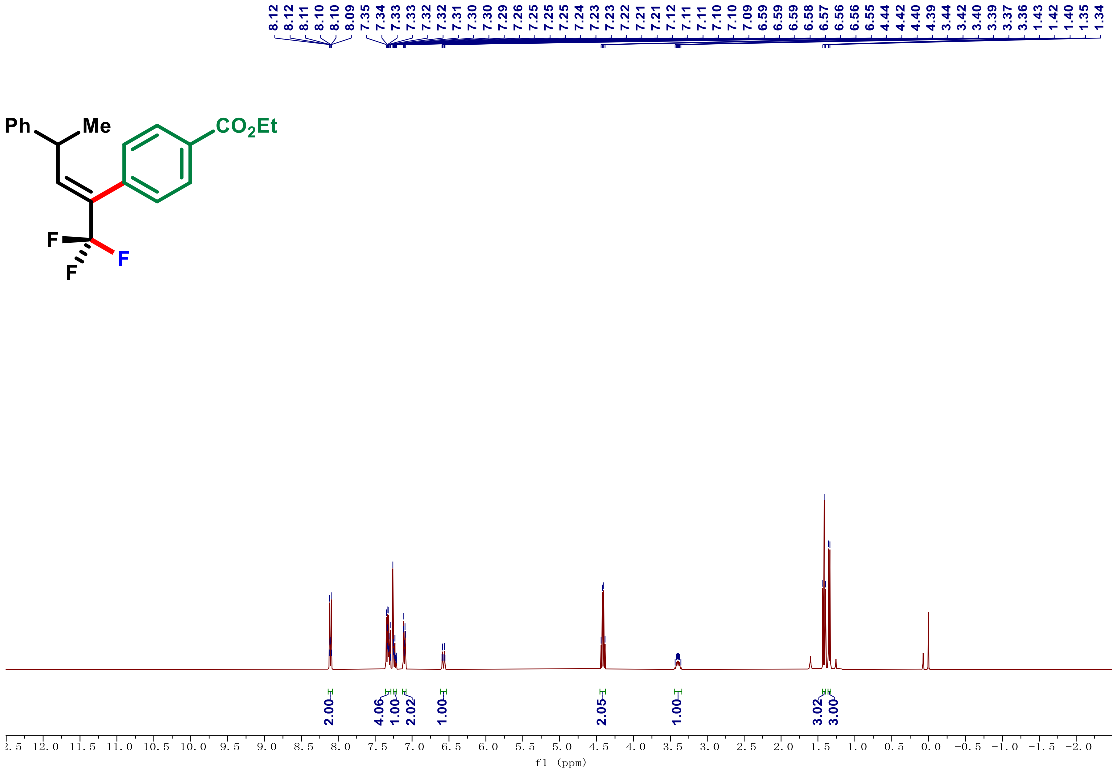

^{19}F NMR Spectrum of **3ia** ($CDCl_3$, 376 MHz)

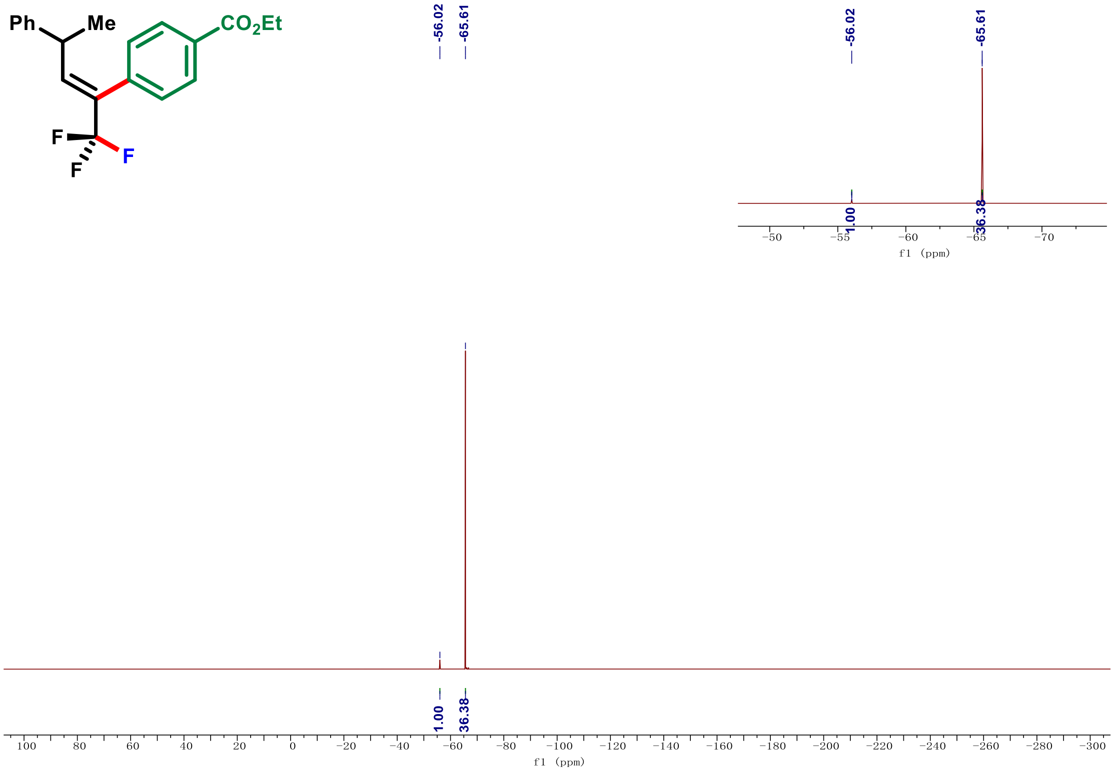

^{13}C NMR Spectrum of **3ia** ($CDCl_3$, 101 MHz)

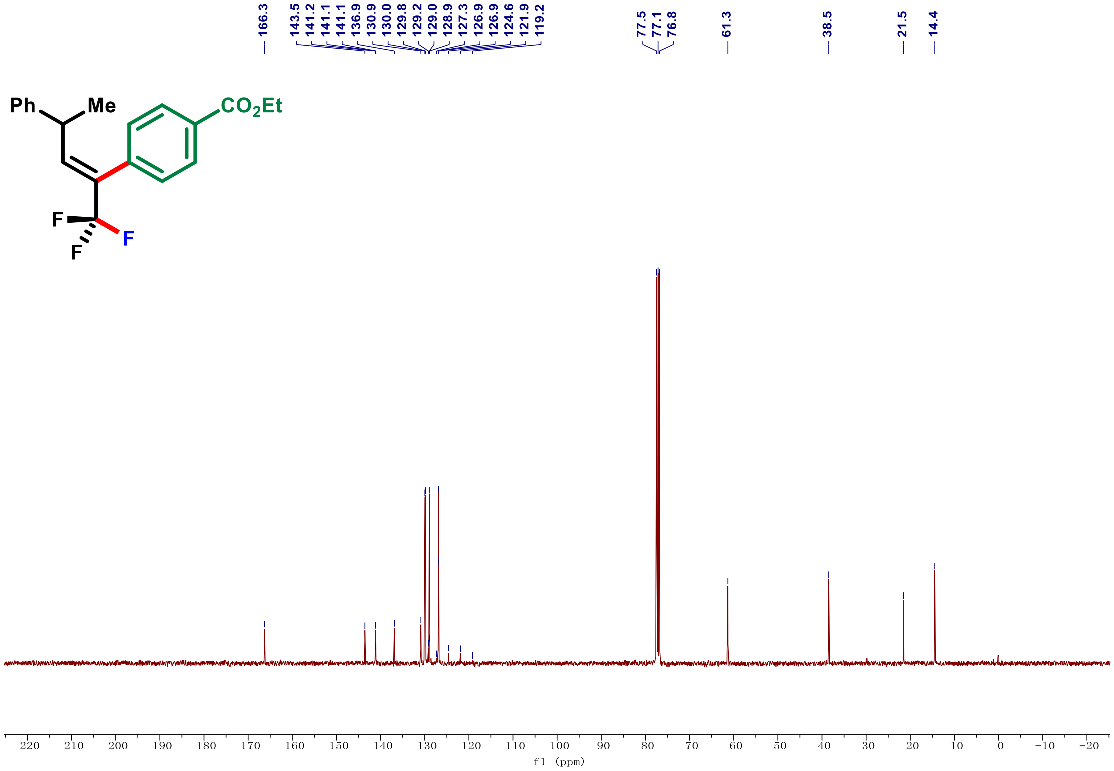

^{1}H NMR Spectrum of **3ja** ($CDCl_3$, 400 MHz)

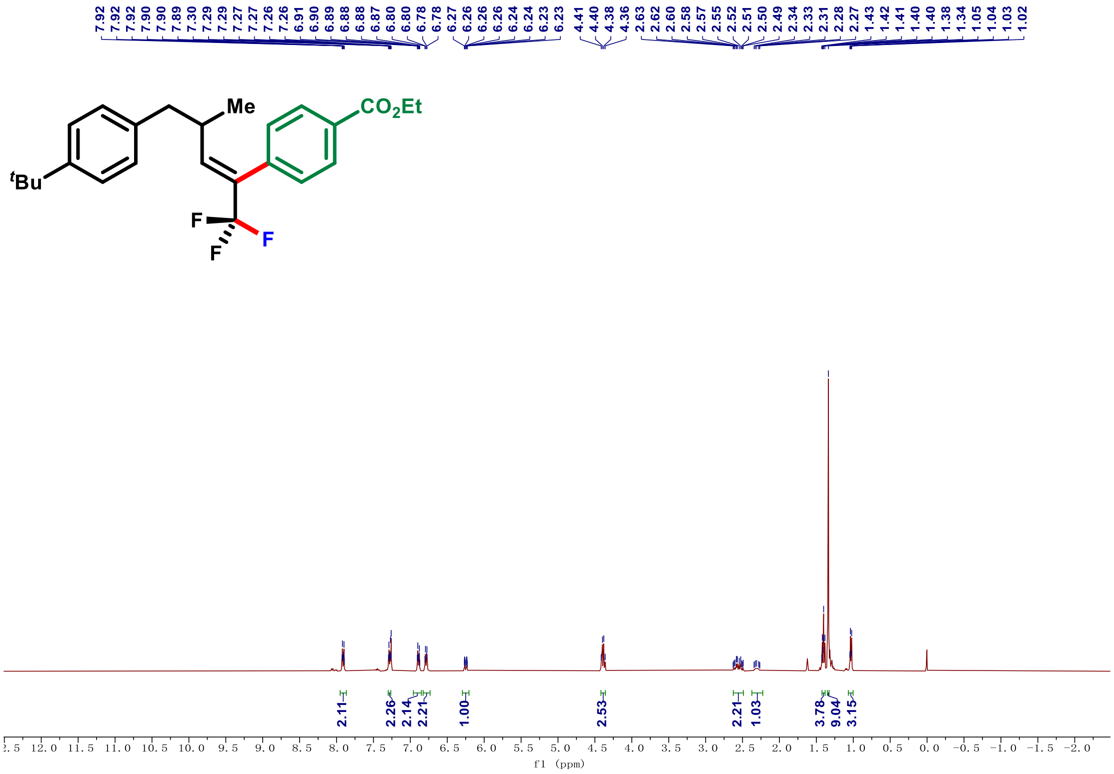

^{19}F NMR Spectrum of **3ja** ($CDCl_3$, 376 MHz)

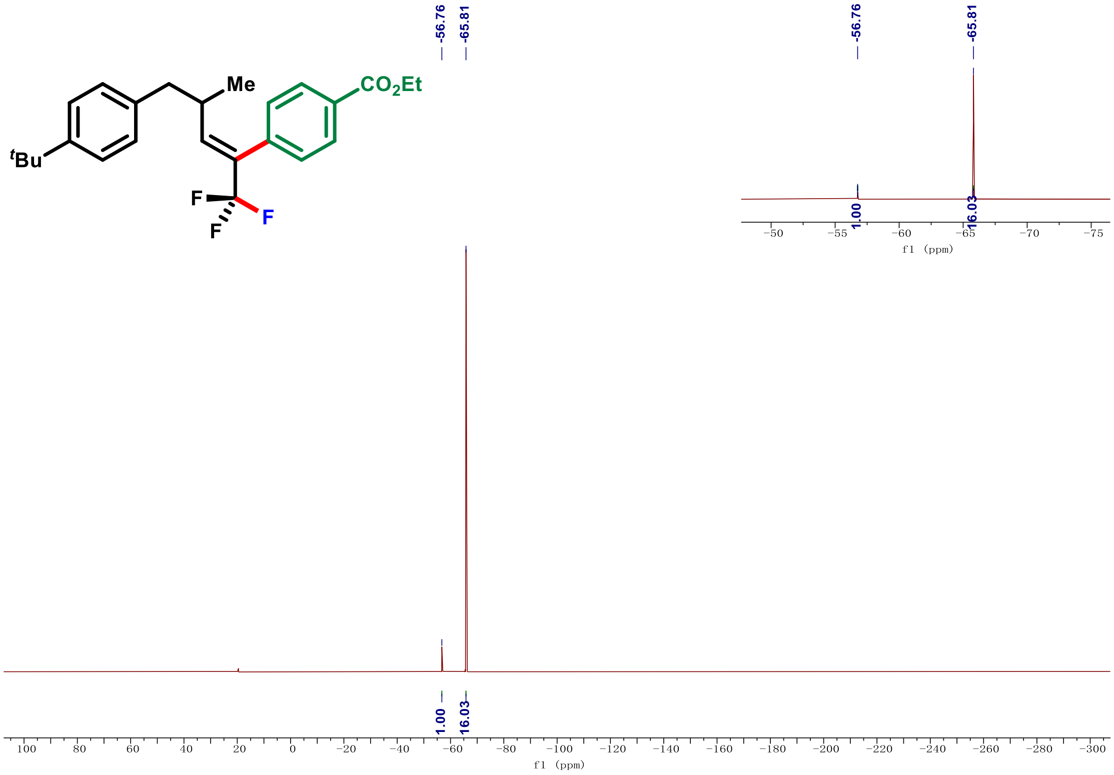

^{13}C NMR Spectrum of **3ja** ($CDCl_3$, 101 MHz)

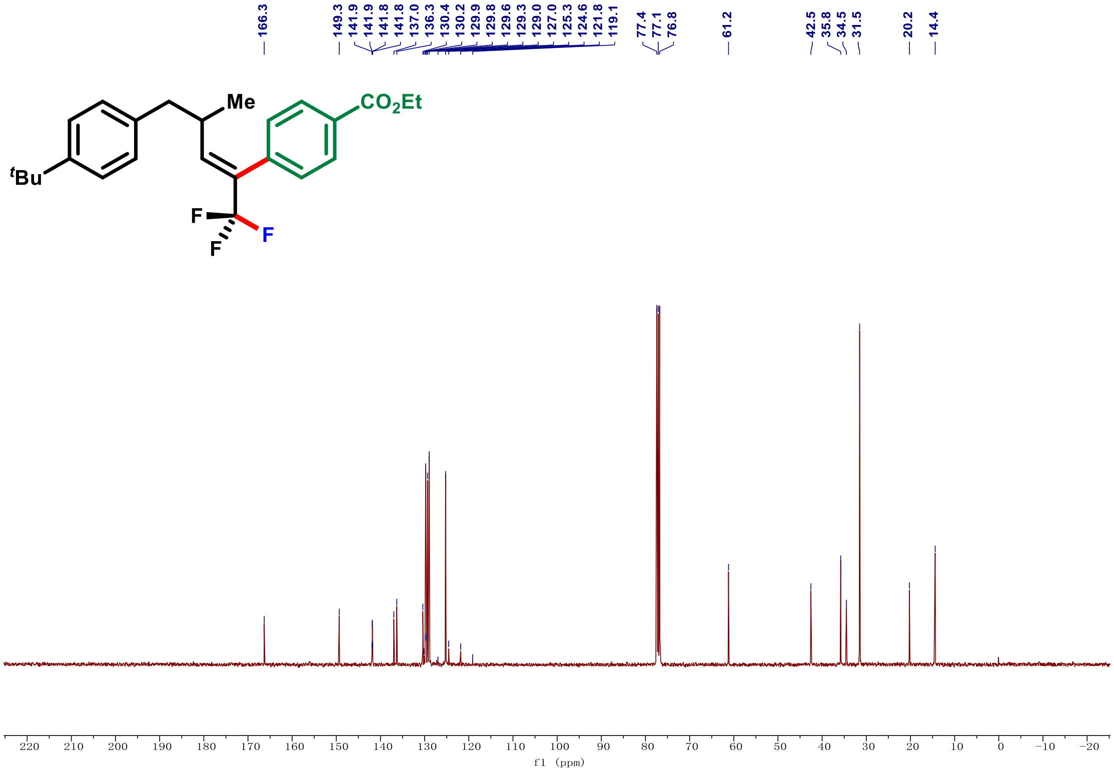

^{1}H NMR Spectrum of **3ka** ($CDCl_3$, 400 MHz)

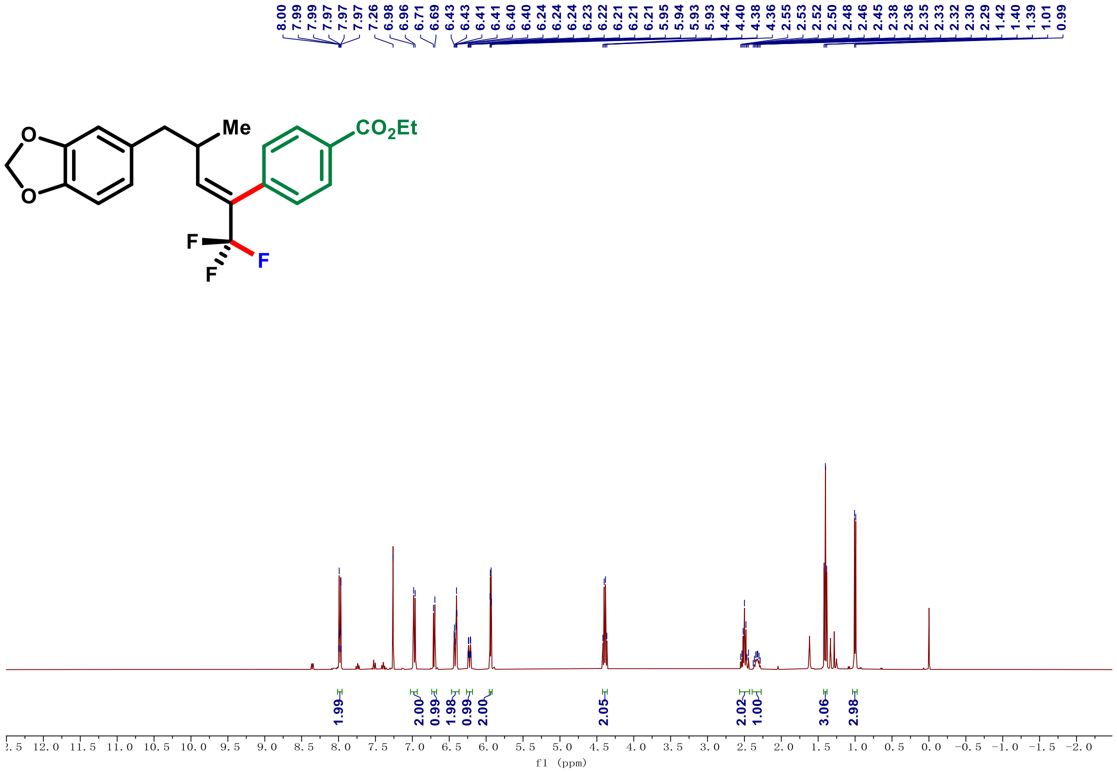

^{19}F NMR Spectrum of **3ka** ($CDCl_3$, 376 MHz)

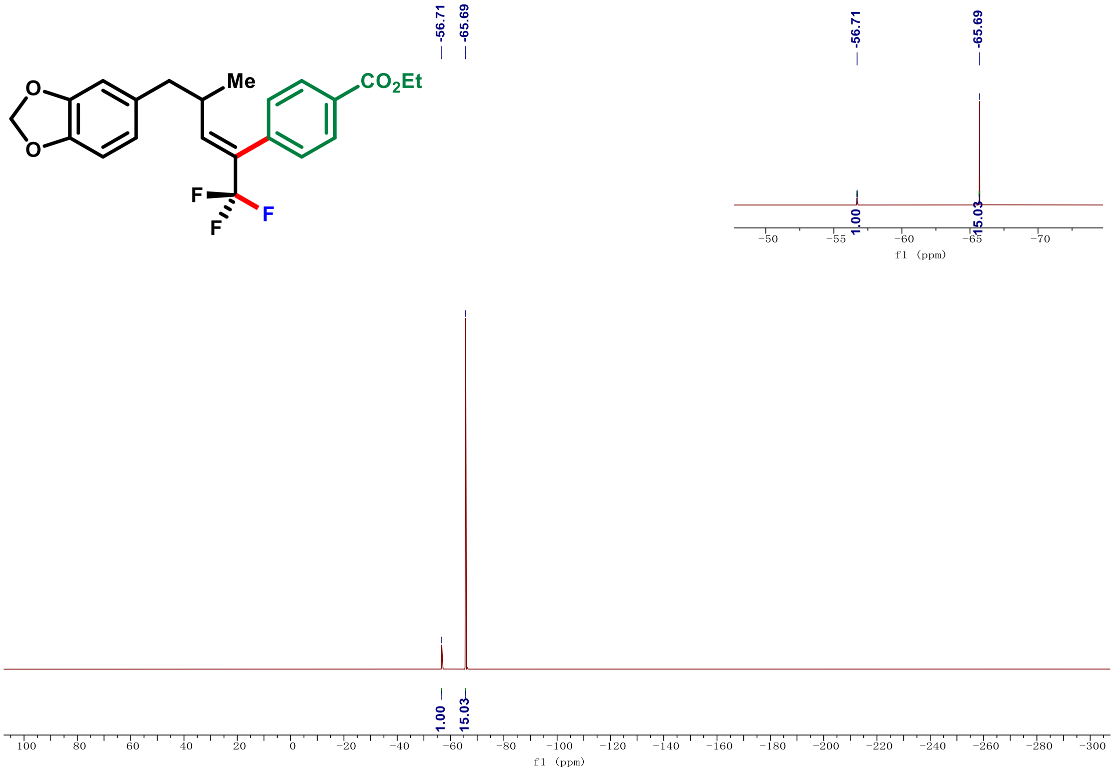

^{13}C NMR Spectrum of **3ka** ($CDCl_3$, 101 MHz)

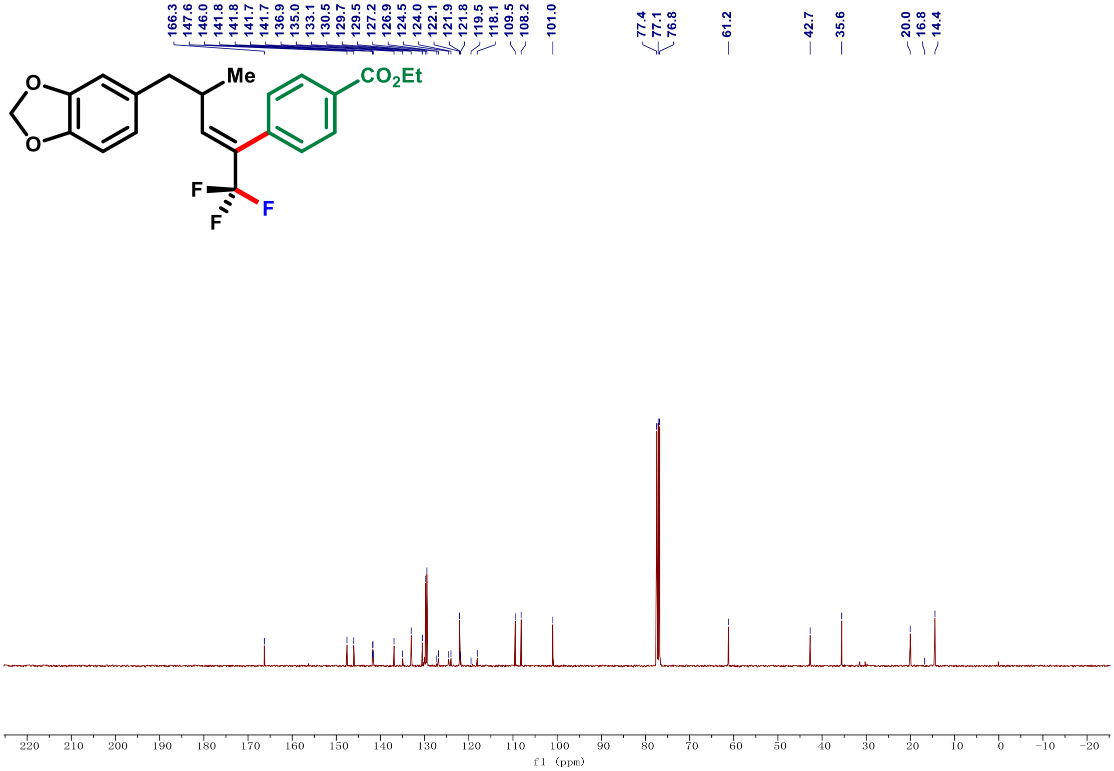

^{1}H NMR Spectrum of **3la** ($CDCl_3$, 400 MHz)

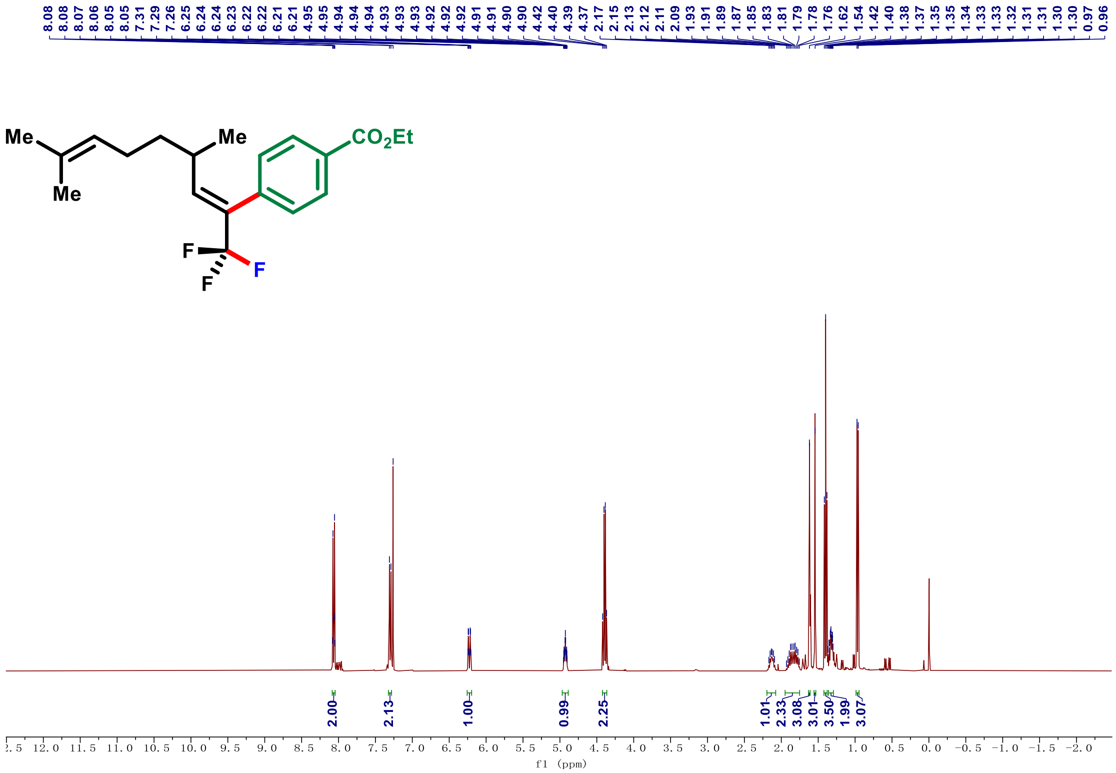

^{19}F NMR Spectrum of **3la** ($CDCl_3$, 376 MHz)

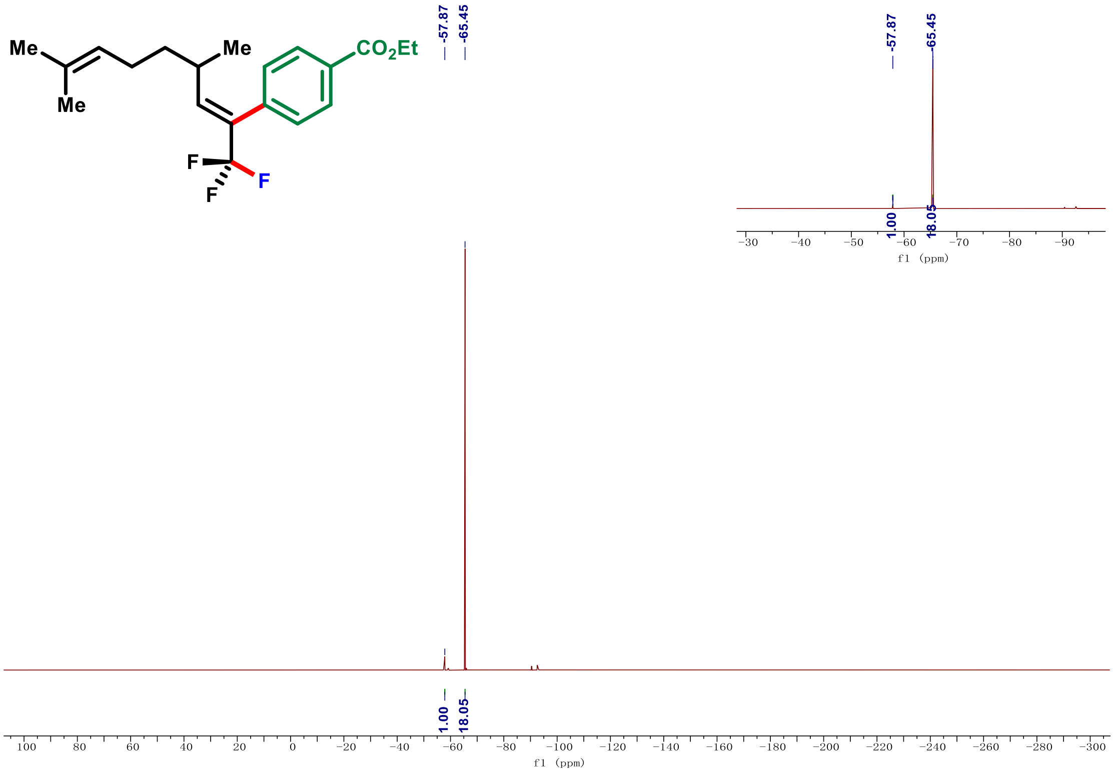

^{13}C NMR Spectrum of **3la** ($CDCl_3$, 101 MHz)

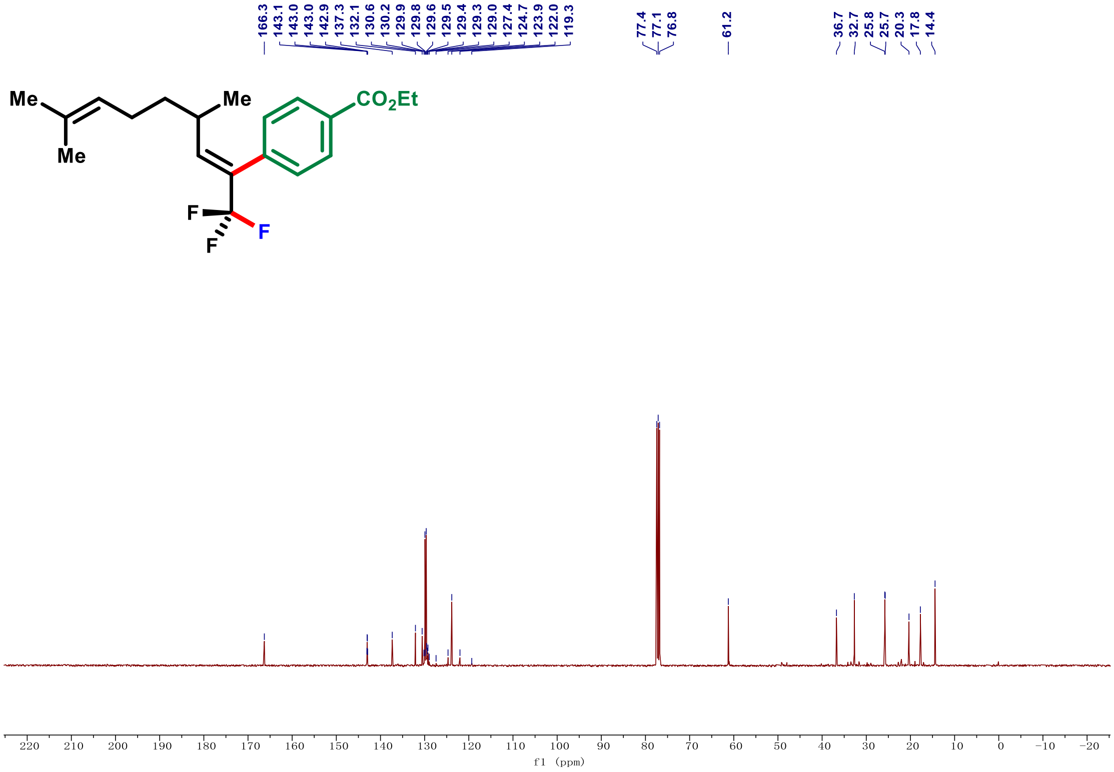

^{1}H NMR Spectrum of **3ma** ($CDCl_3$, 400 MHz)

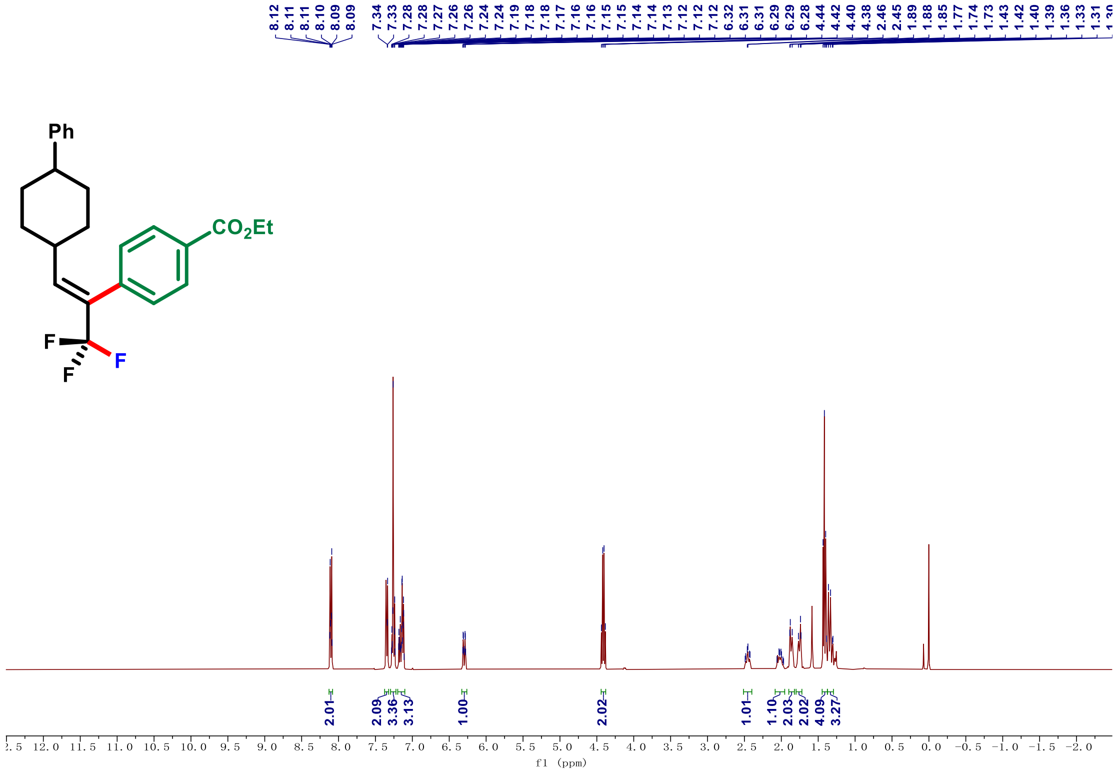

^{19}F NMR Spectrum of **3ma** ($CDCl_3$, 376 MHz)

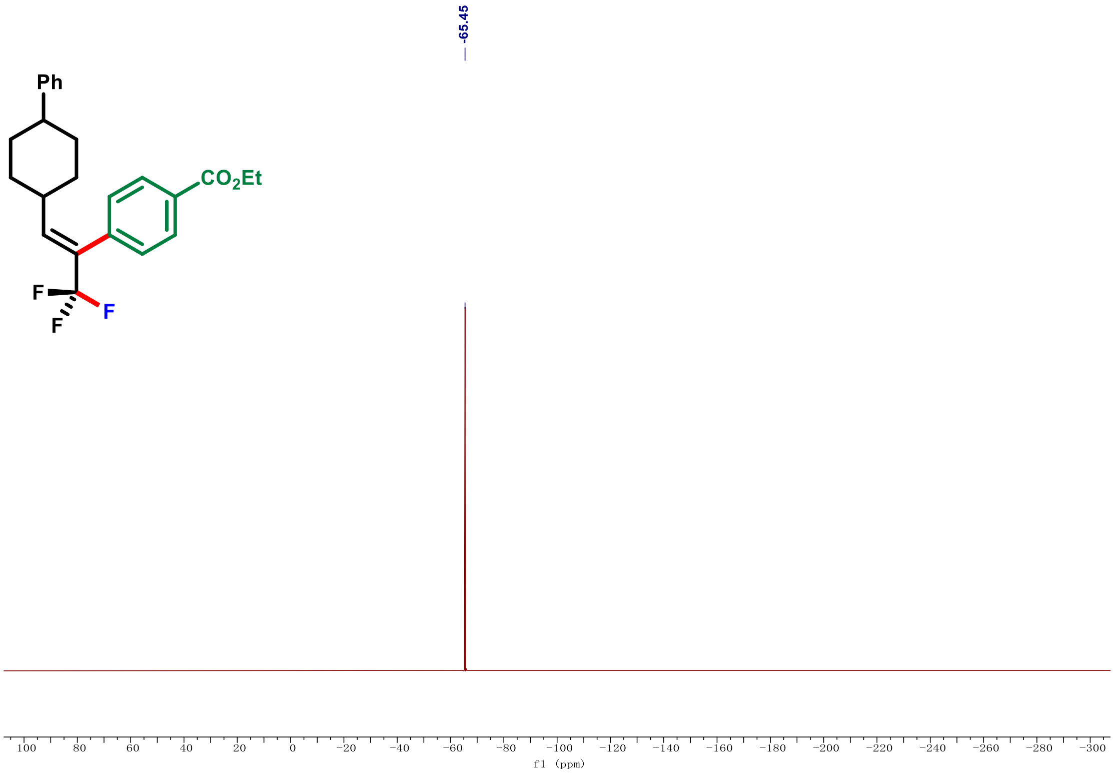

^{13}C NMR Spectrum of **3ma** ($CDCl_3$, 101 MHz)

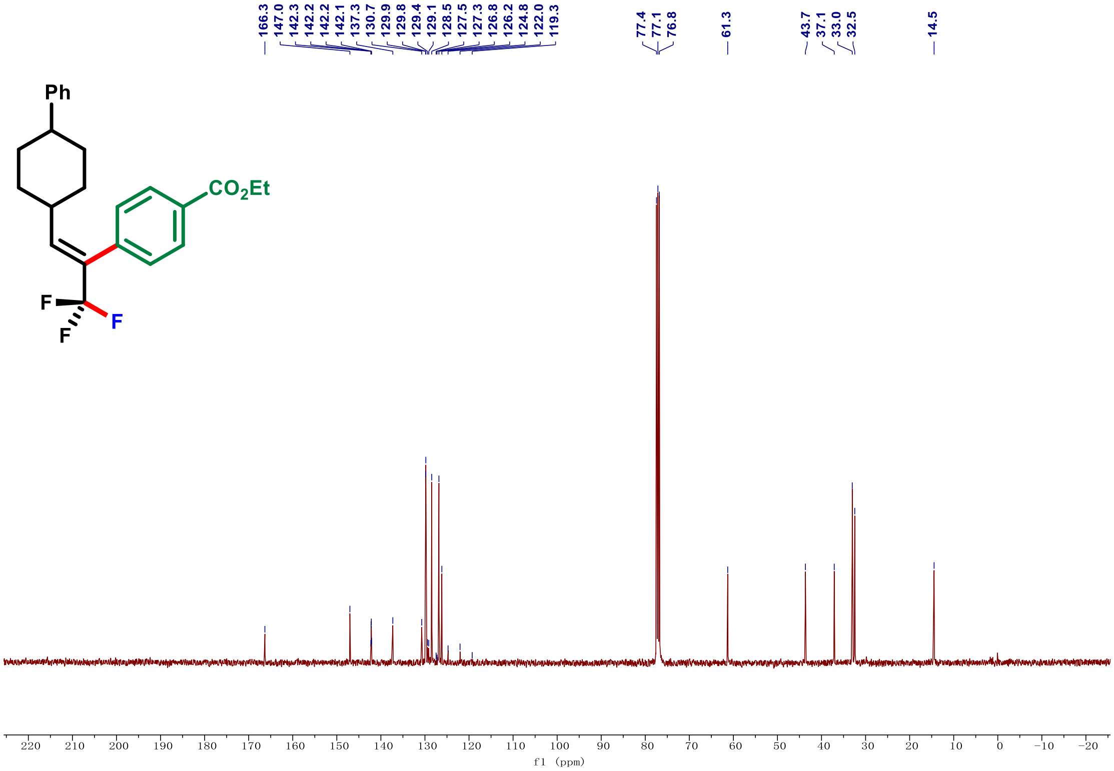

^{1}H NMR Spectrum of **3na** ($CDCl_3$, 400 MHz)

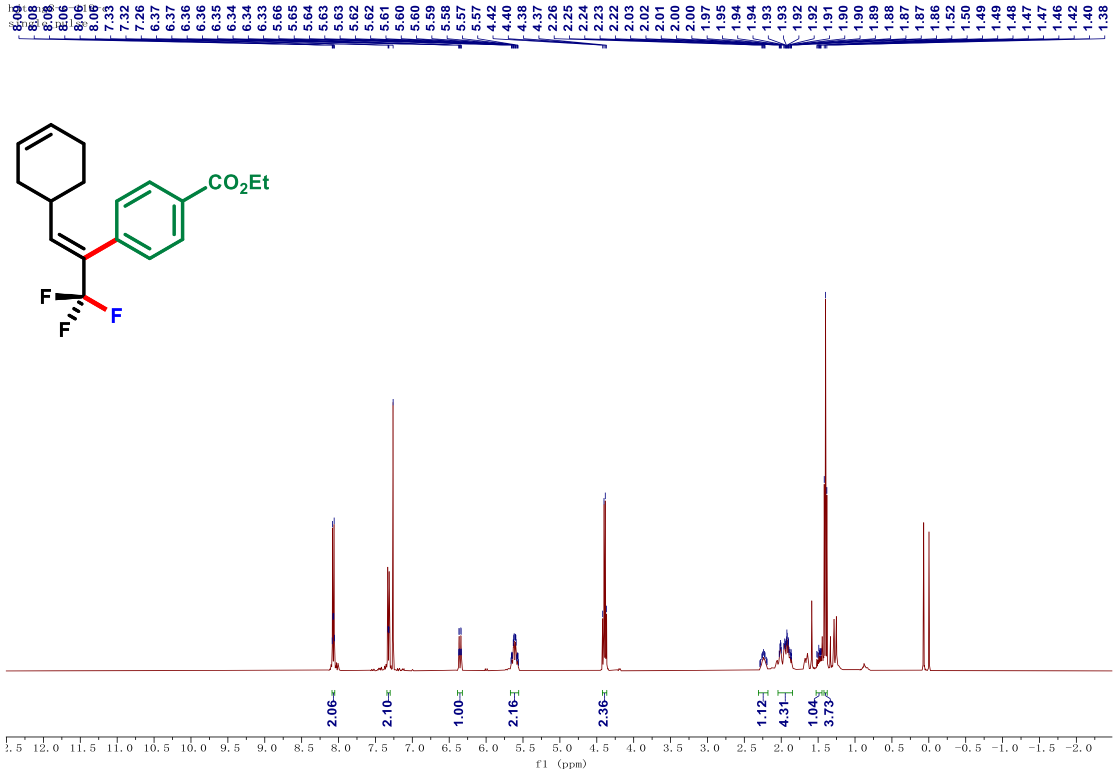

^{19}F NMR Spectrum of **3na** ($CDCl_3$, 376 MHz)

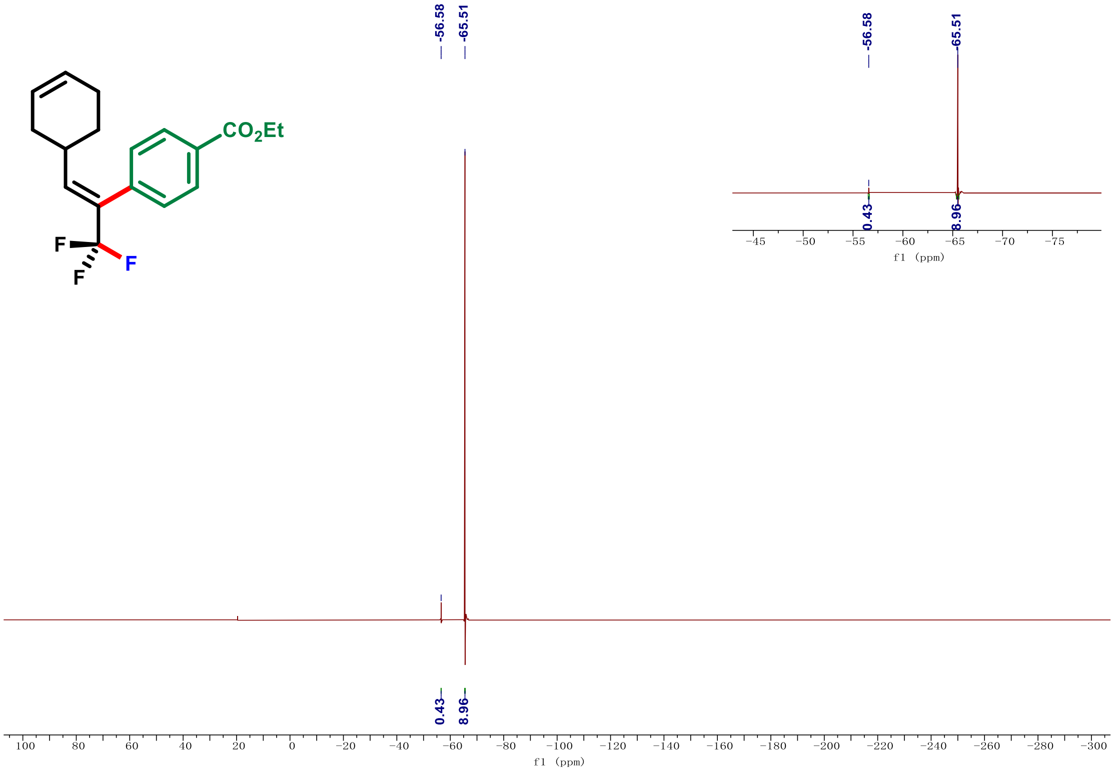

^{13}C NMR Spectrum of **3na** ($CDCl_3$, 101 MHz)

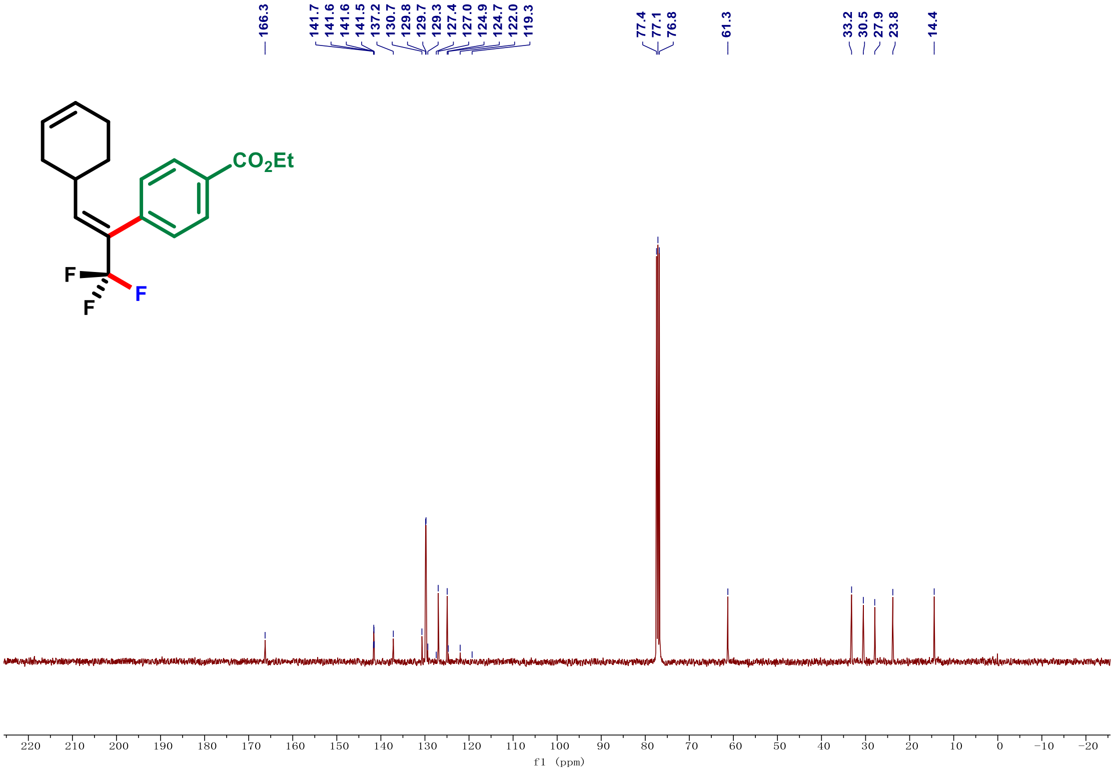

^{1}H NMR Spectrum of **3oa** ($CDCl_3$, 400 MHz)

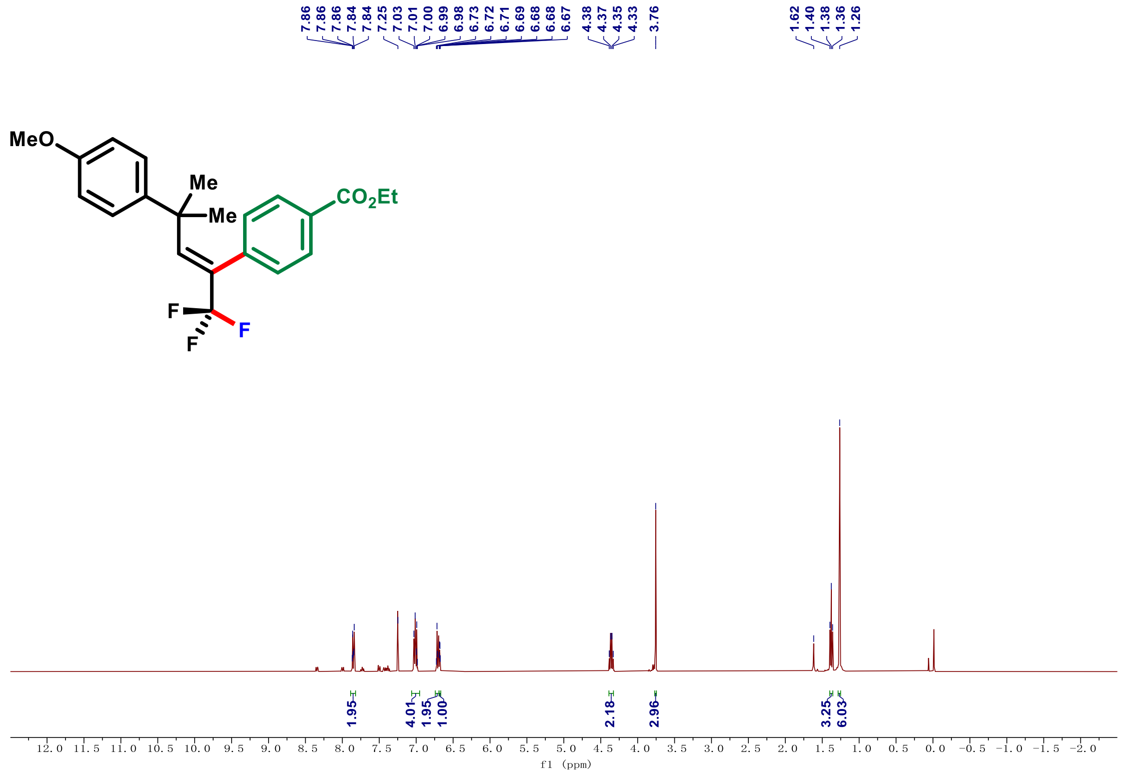

^{19}F NMR Spectrum of **3oa** ($CDCl_3$, 376 MHz)

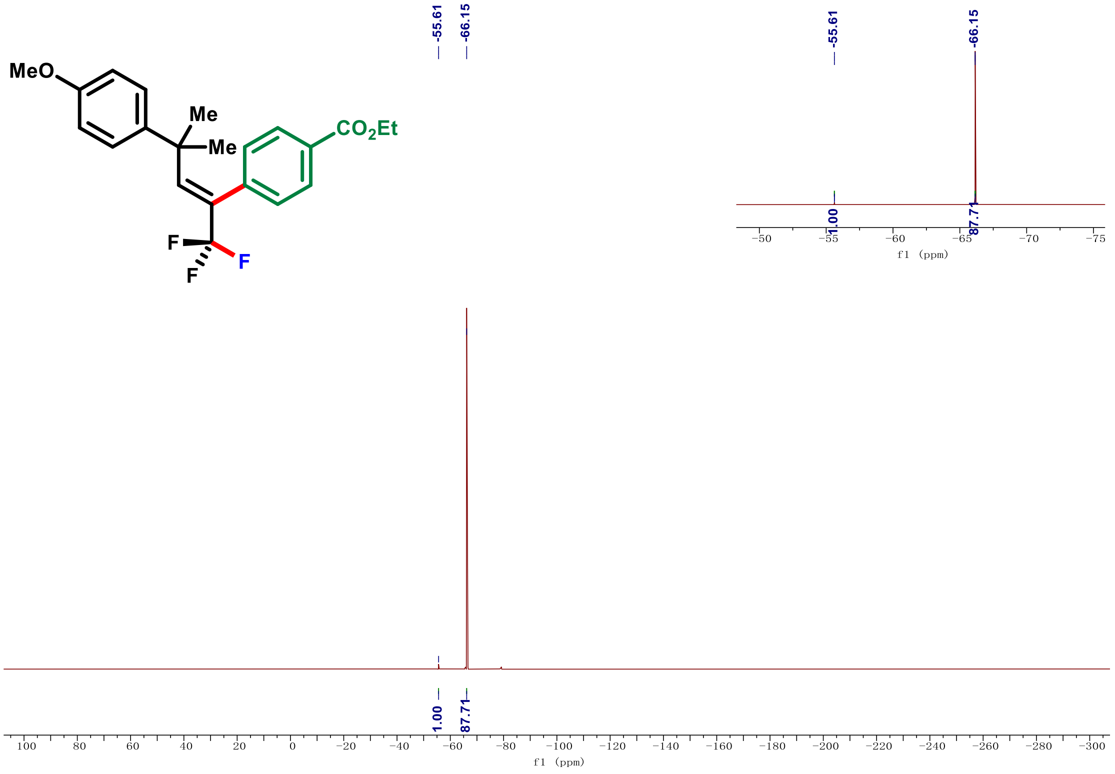

^{13}C NMR Spectrum of **3oa** ($CDCl_3$, 101 MHz)

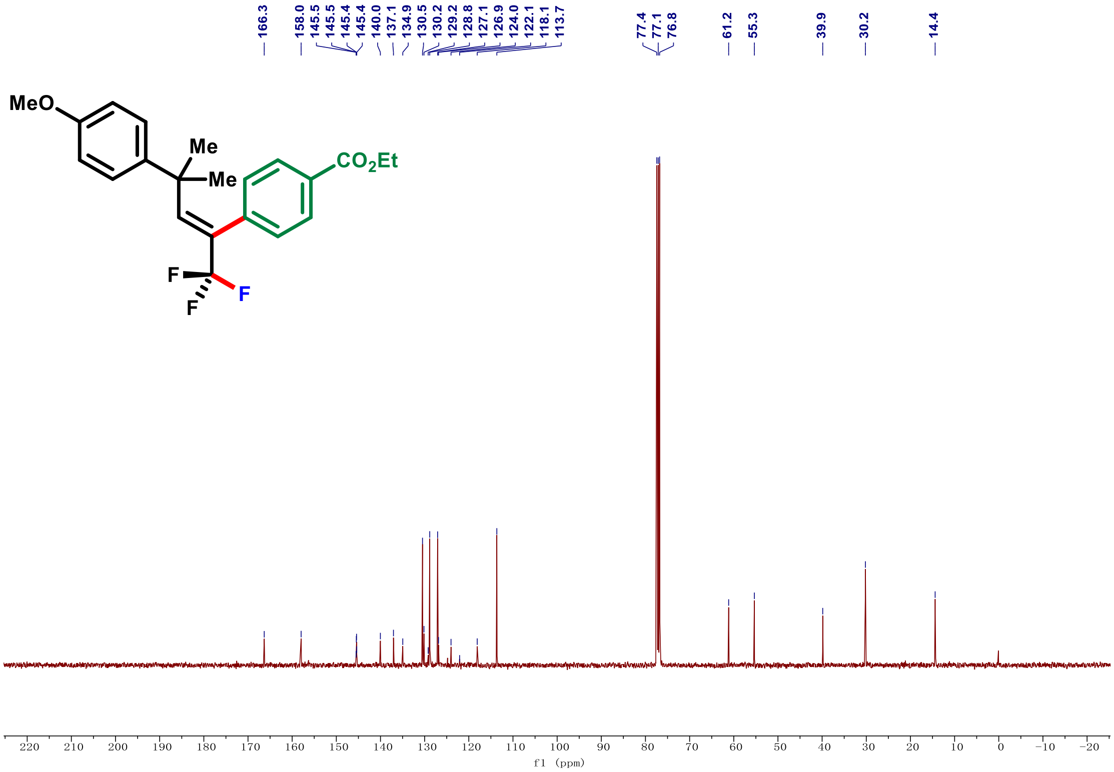

^{1}H NMR Spectrum of **3pa** ($CDCl_3$, 400 MHz)

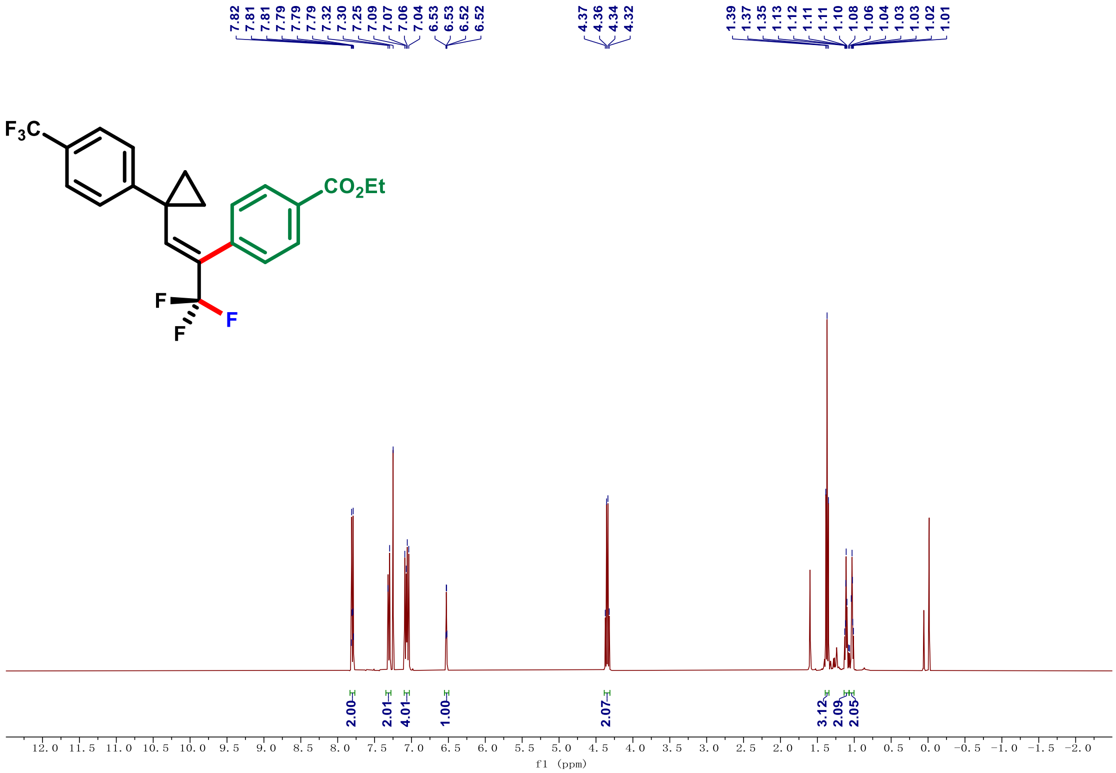

^{19}F NMR Spectrum of **3pa** ($CDCl_3$, 376 MHz)

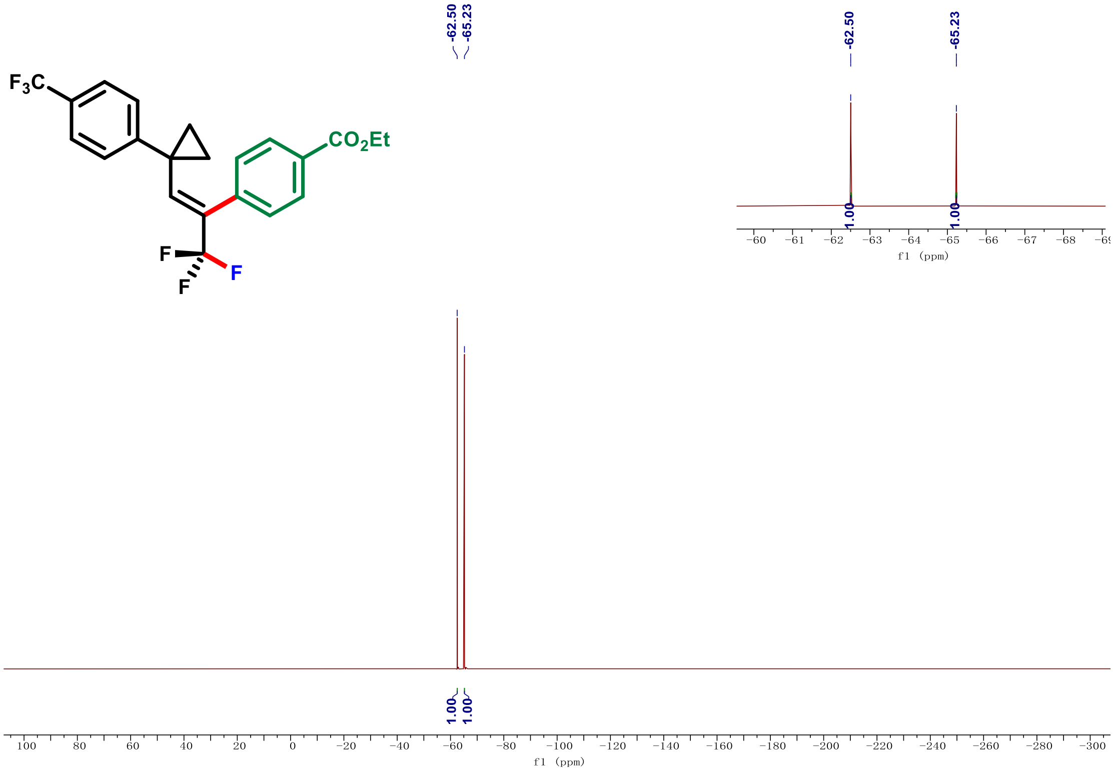

^{13}C NMR Spectrum of **3pa** ($CDCl_3$, 101 MHz)

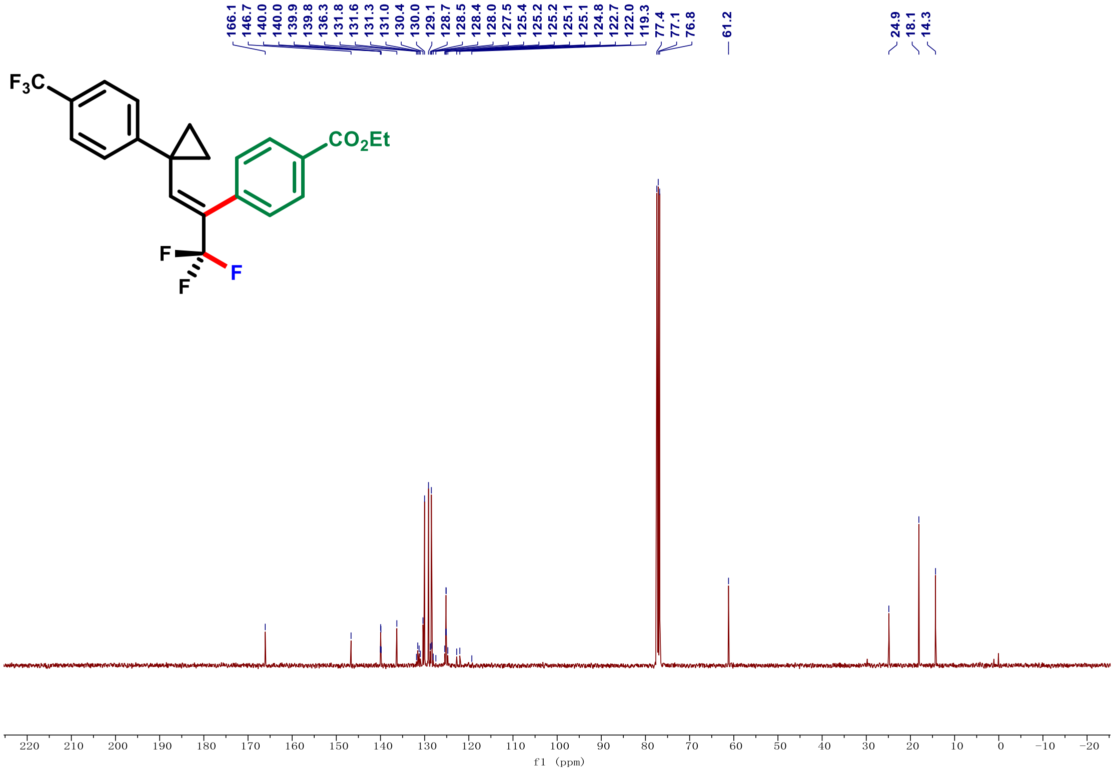

^{1}H NMR Spectrum of **3qa** ($CDCl_3$, 400 MHz)

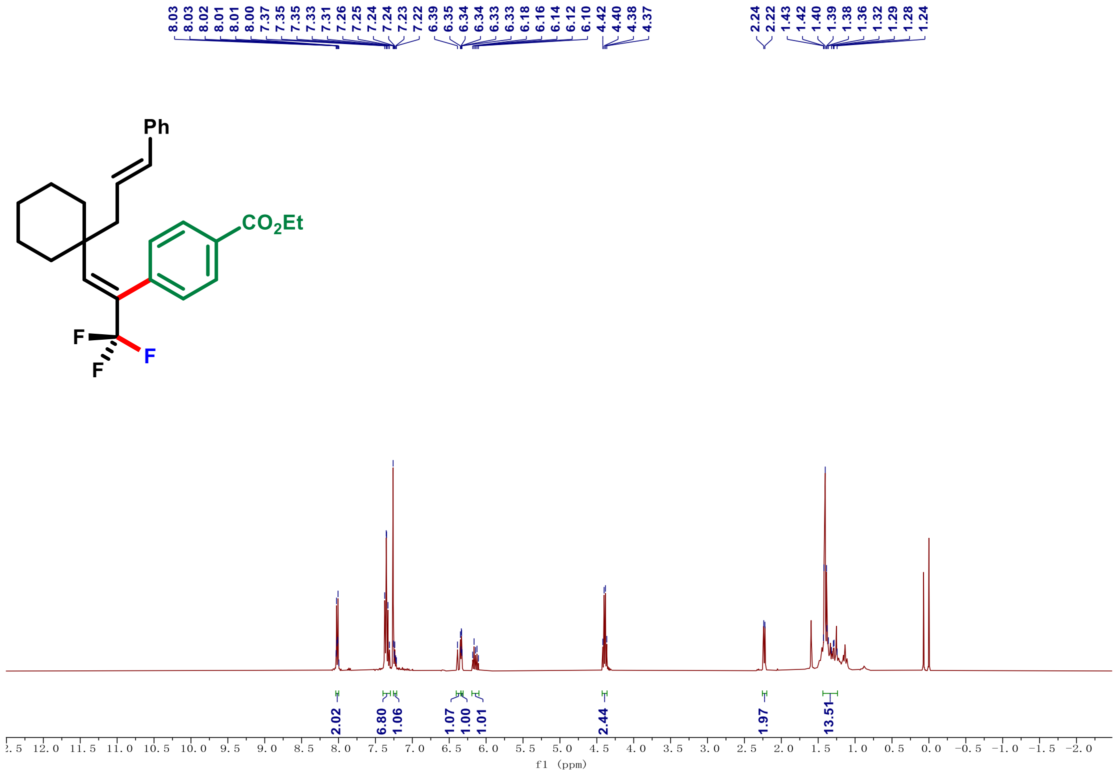

^{19}F NMR Spectrum of **3qa** ($CDCl_3$, 376 MHz)

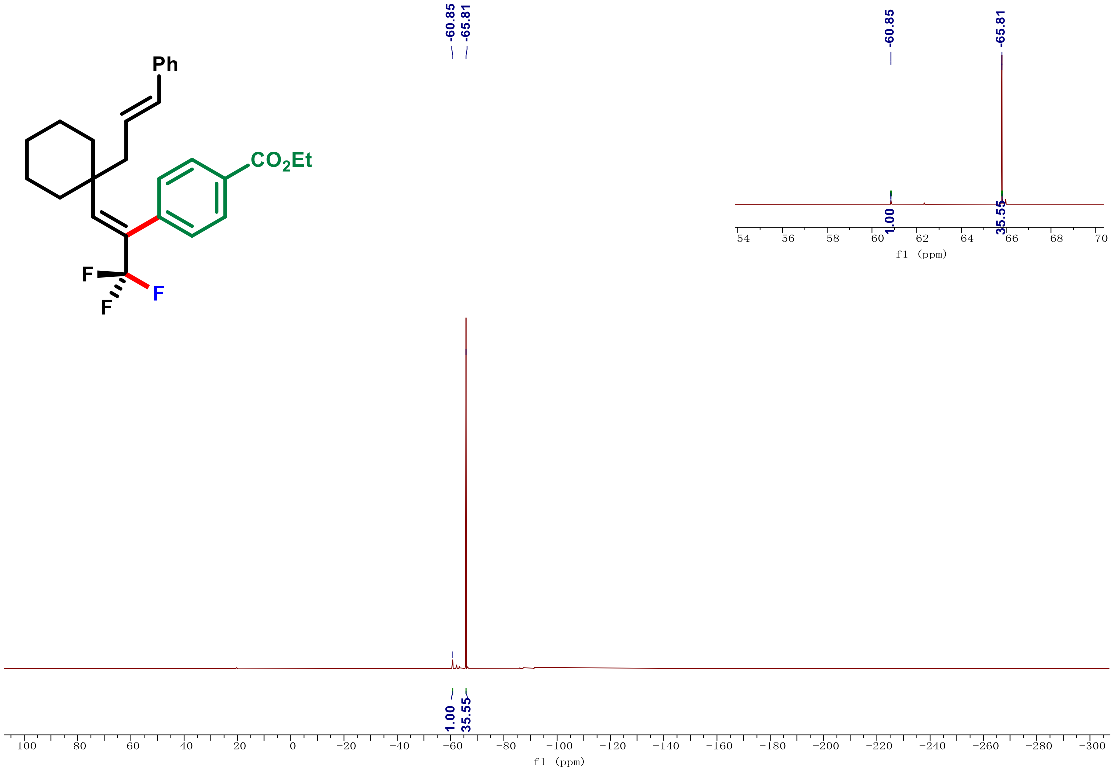

^{13}C NMR Spectrum of **3qa** ($CDCl_3$, 101 MHz)

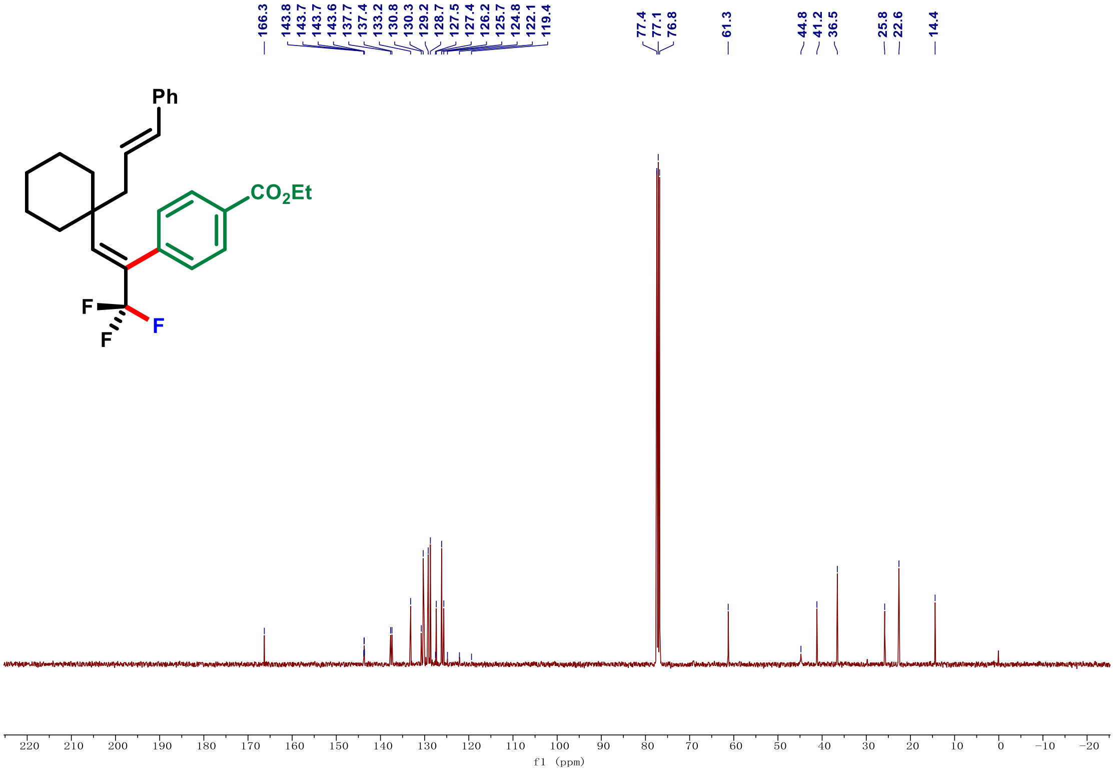

^{1}H NMR Spectrum of **3ra** ($CDCl_3$, 400 MHz)

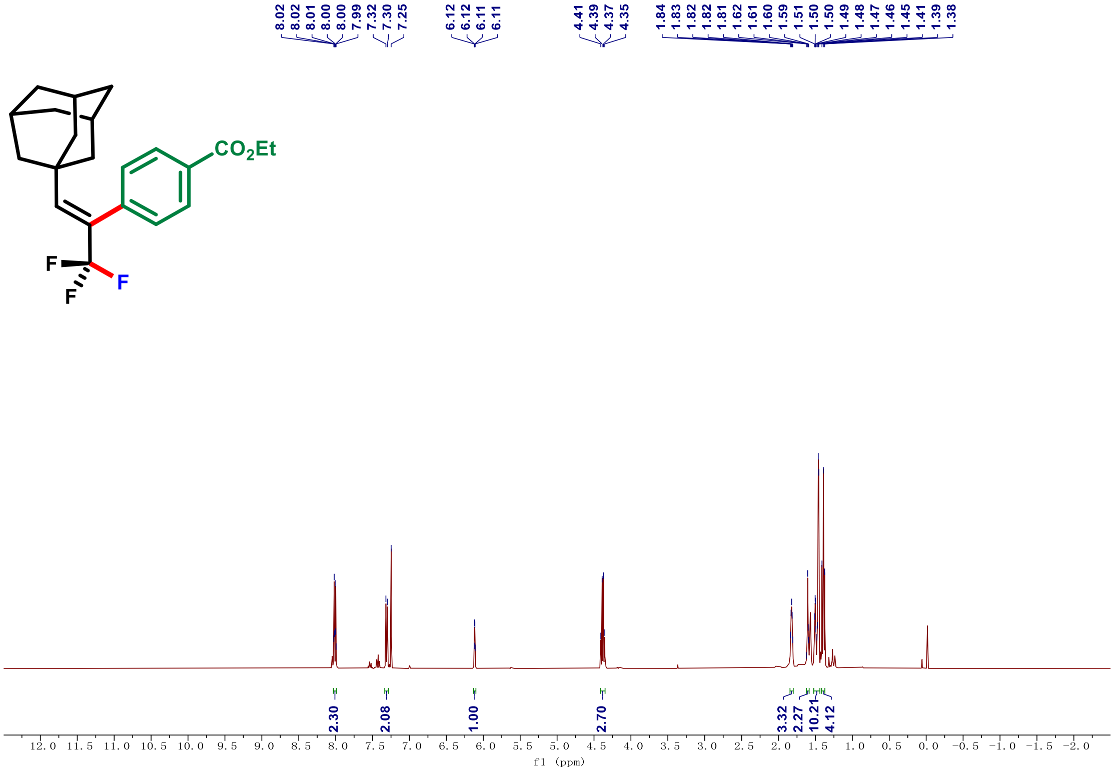

^{19}F NMR Spectrum of **3ra** ($CDCl_3$, 376 MHz)

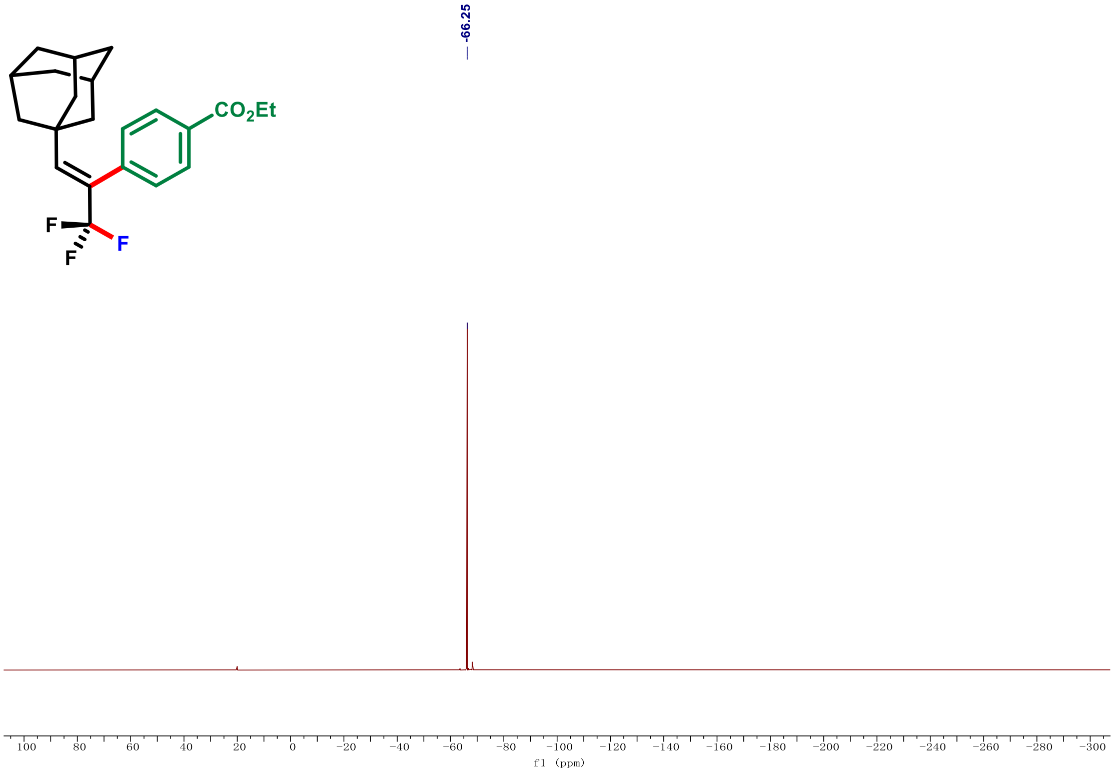

^{13}C NMR Spectrum of **3ra** ($CDCl_3$, 101 MHz)

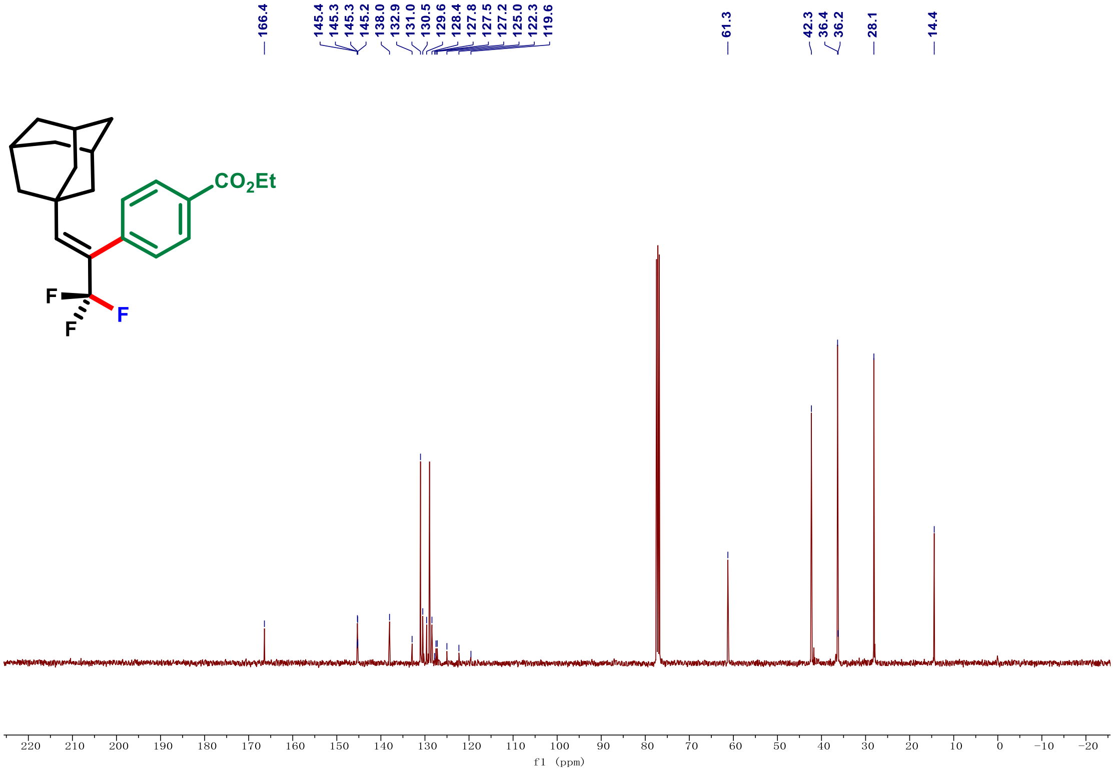

^{1}H NMR Spectrum of **3ab** ($CDCl_3$, 400 MHz)

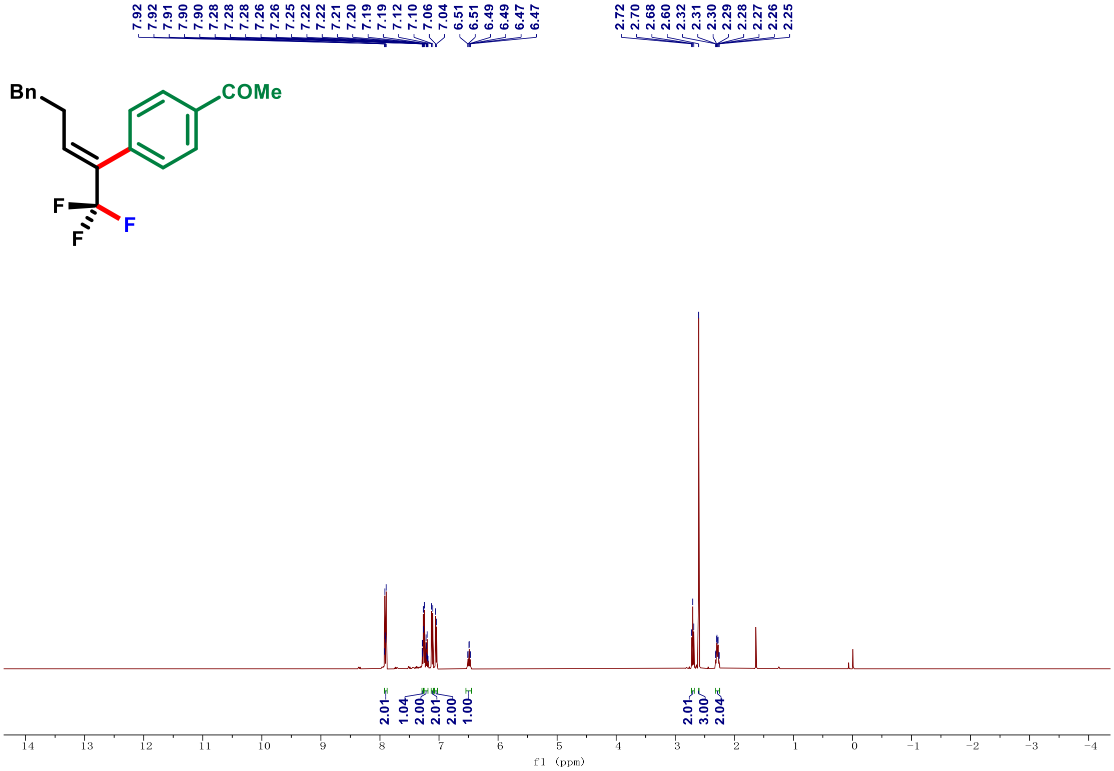

^{19}F NMR Spectrum of **3ab** ($CDCl_3$, 376 MHz)

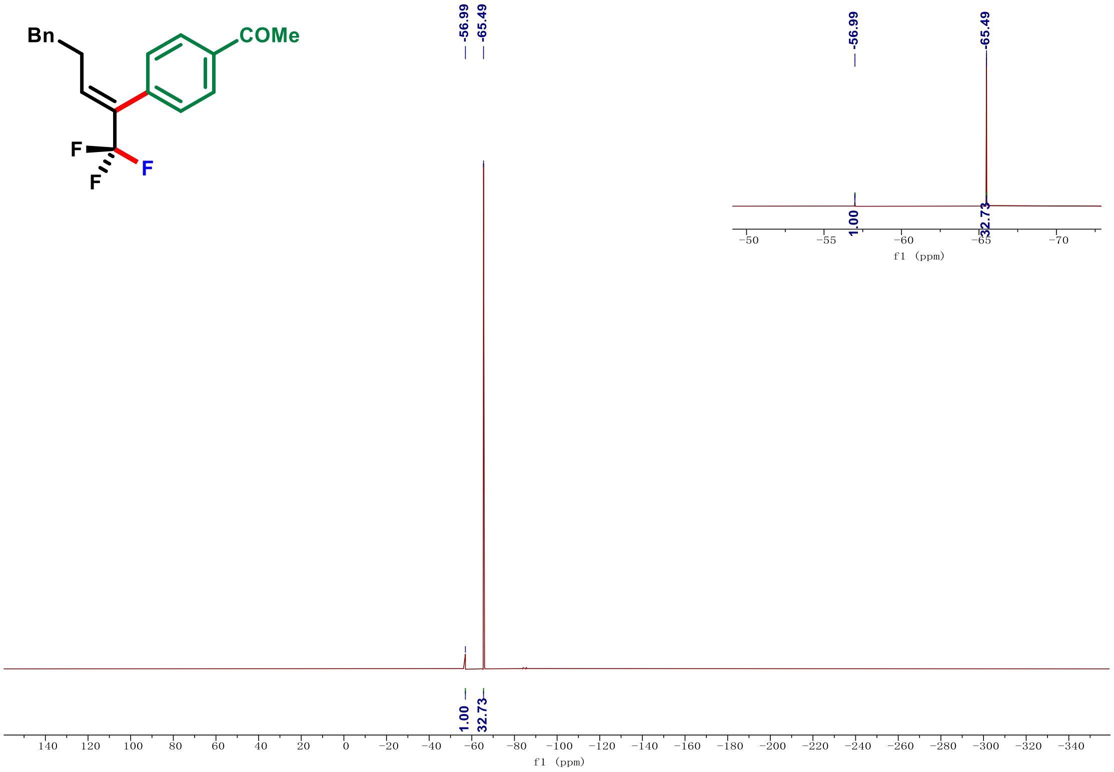

13C NMR Spectrum of **3ab** ($CDCl_3$, 101 MHz)

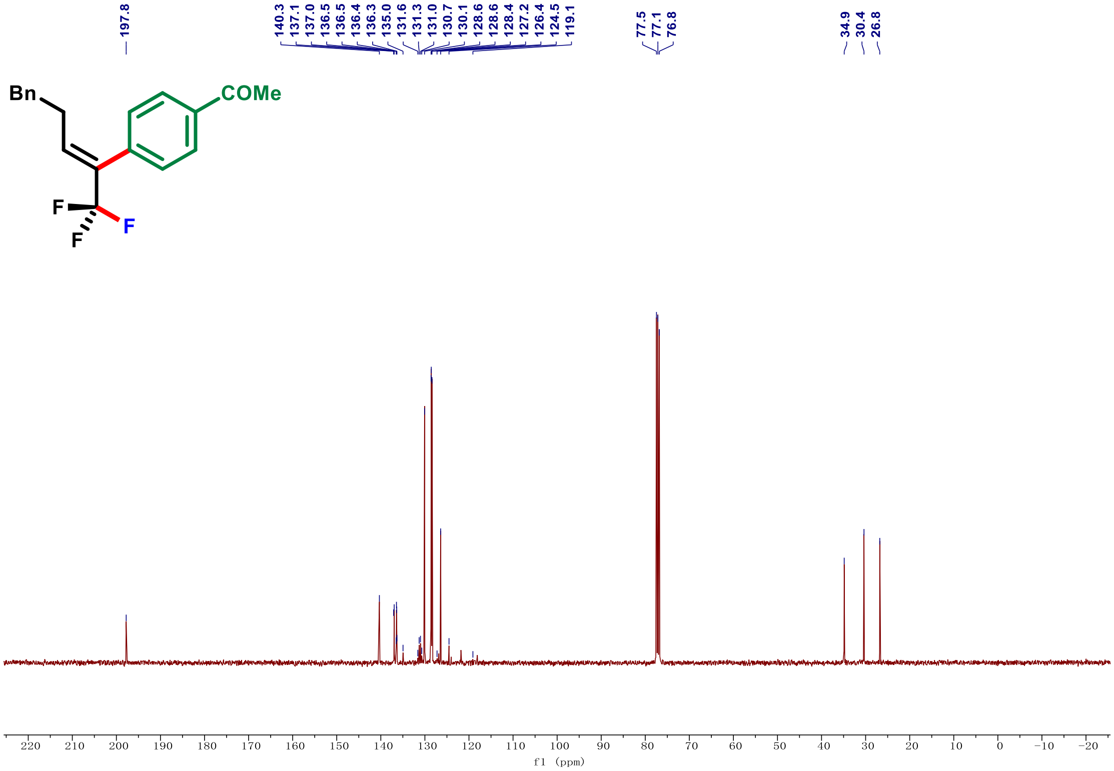

1H NMR Spectrum of **3ac** ($CDCl_3$, 400 MHz)

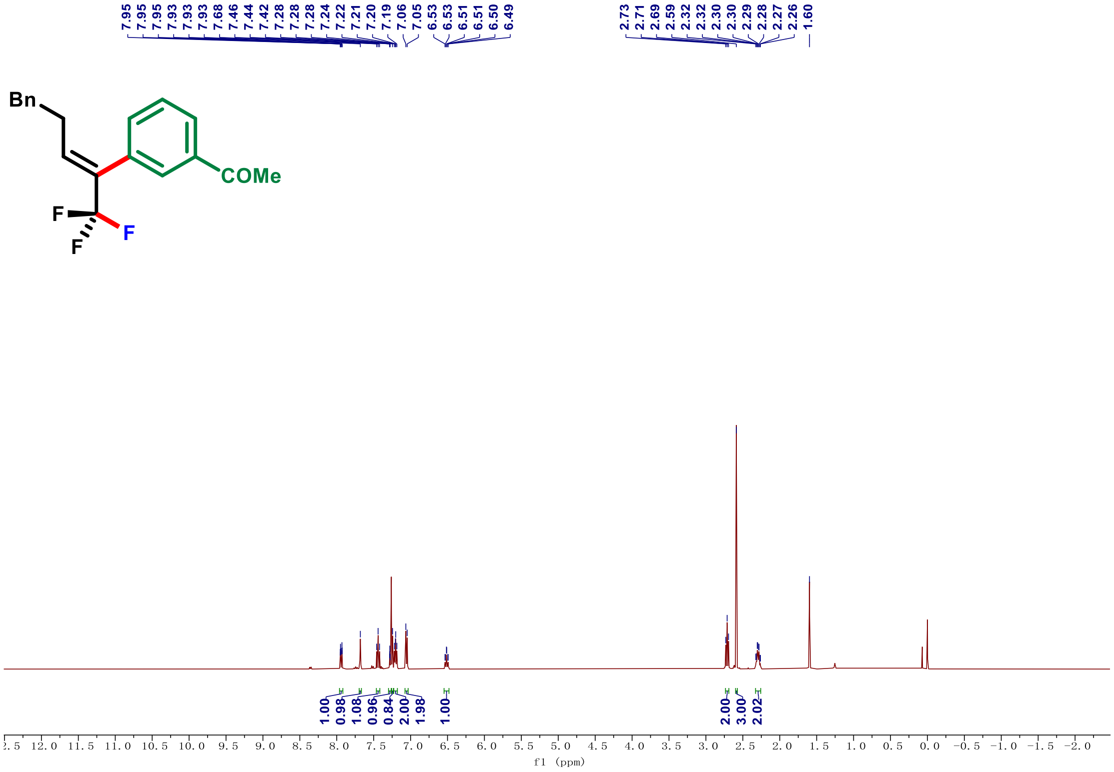

^{19}F NMR Spectrum of **3ac** ($CDCl_3$, 376 MHz)

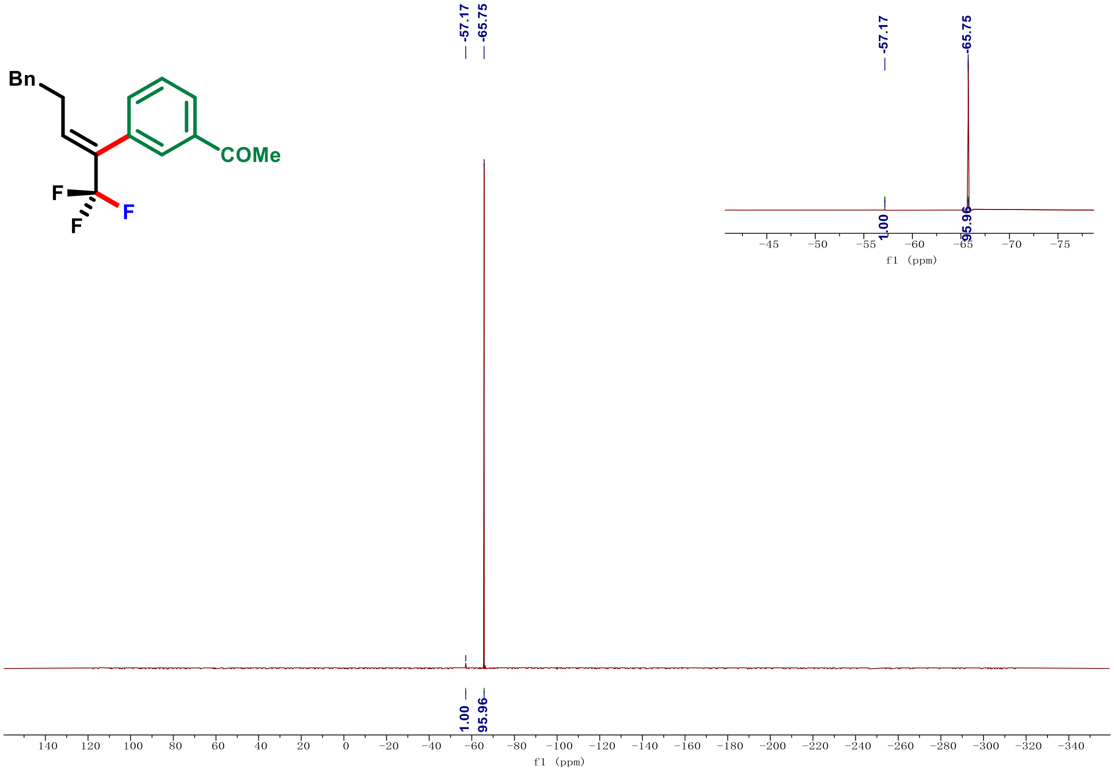

^{13}C NMR Spectrum of **3ac** ($CDCl_3$, 101 MHz)

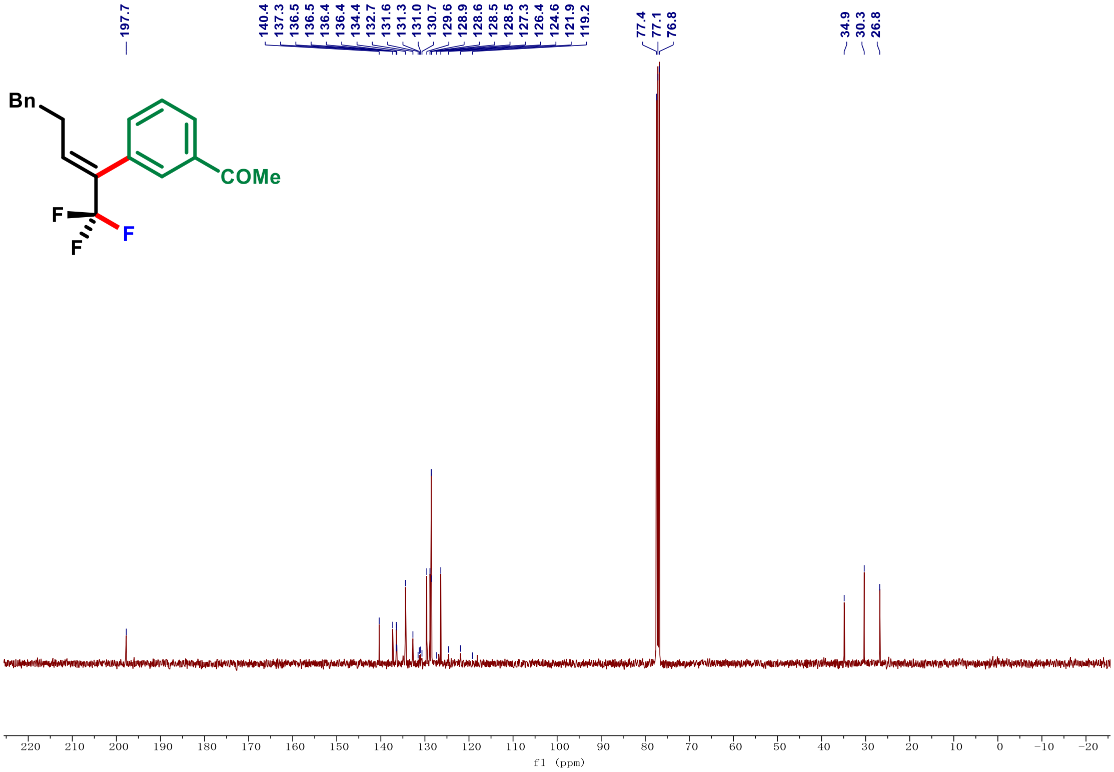

^{1}H NMR Spectrum of **3ad** ($CDCl_3$, 400 MHz)

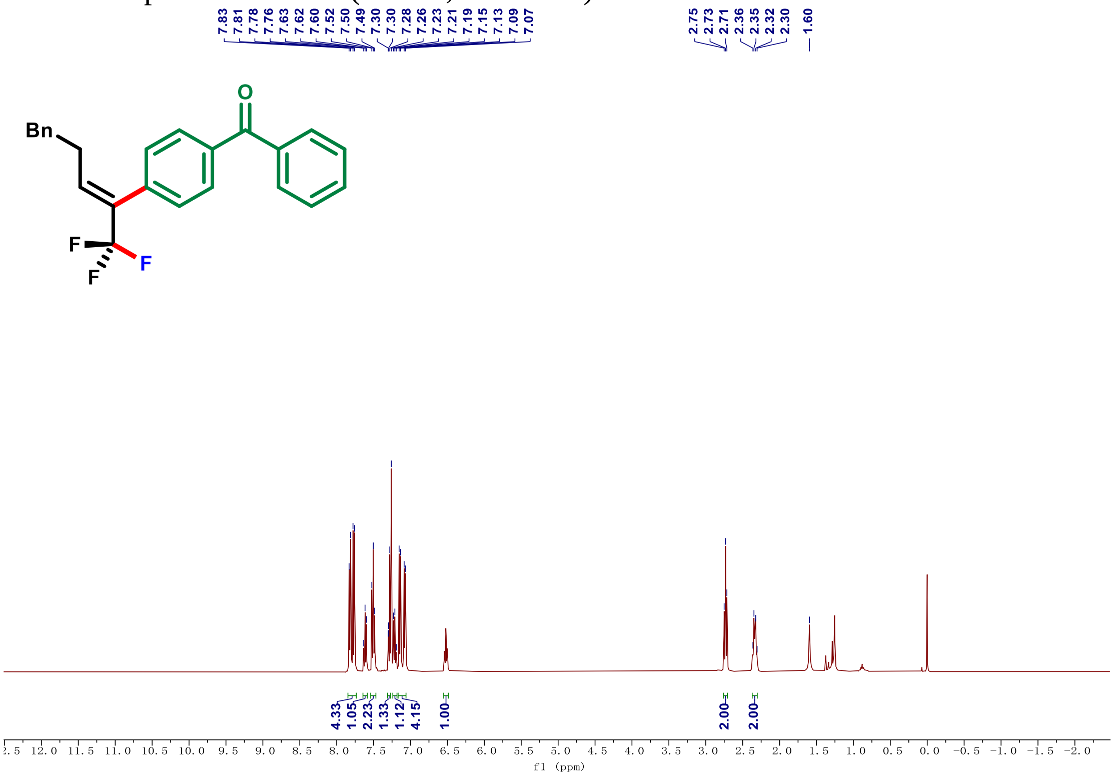

^{19}F NMR Spectrum of **3ad** ($CDCl_3$, 376 MHz)

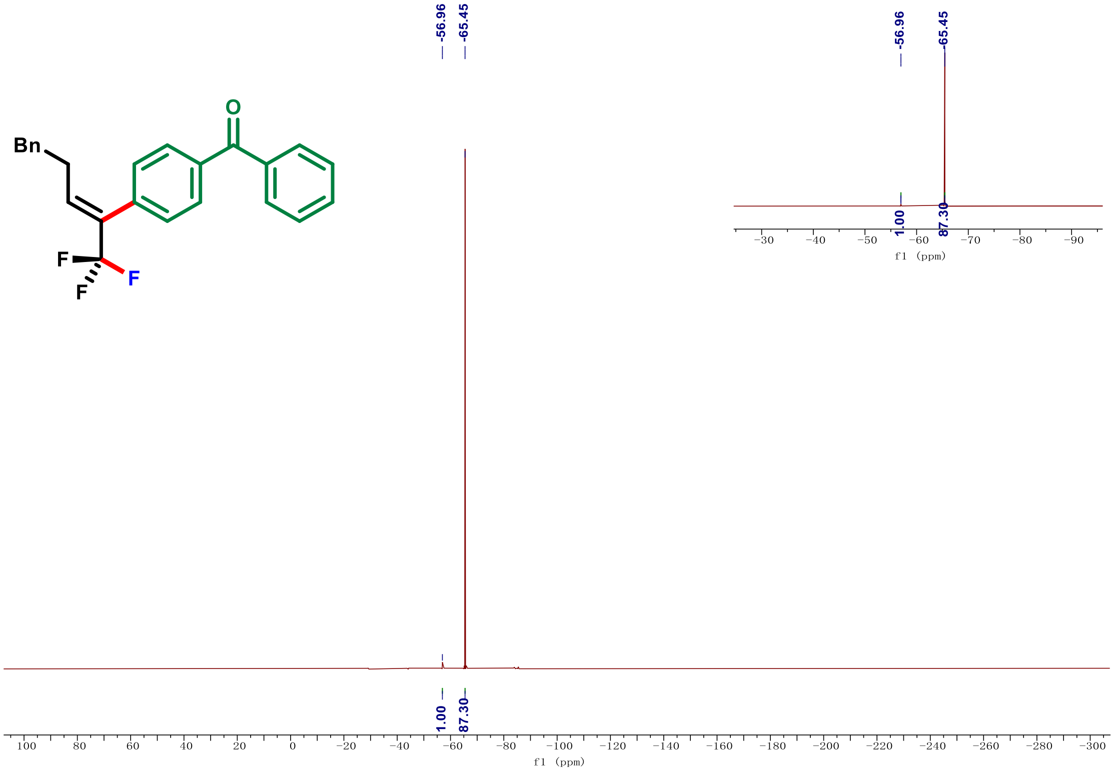

^{13}C NMR Spectrum of **3ad** ($CDCl_3$, 101 MHz)

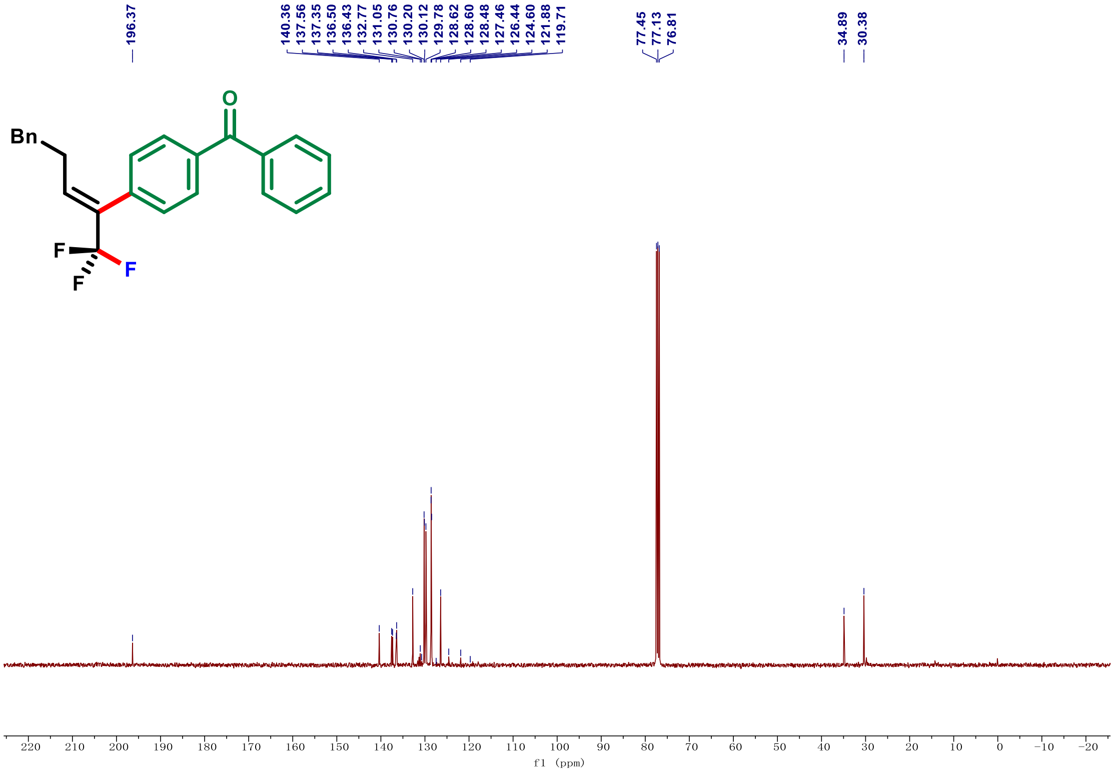

^{1}H NMR Spectrum of **3ae** ($CDCl_3$, 400 MHz)

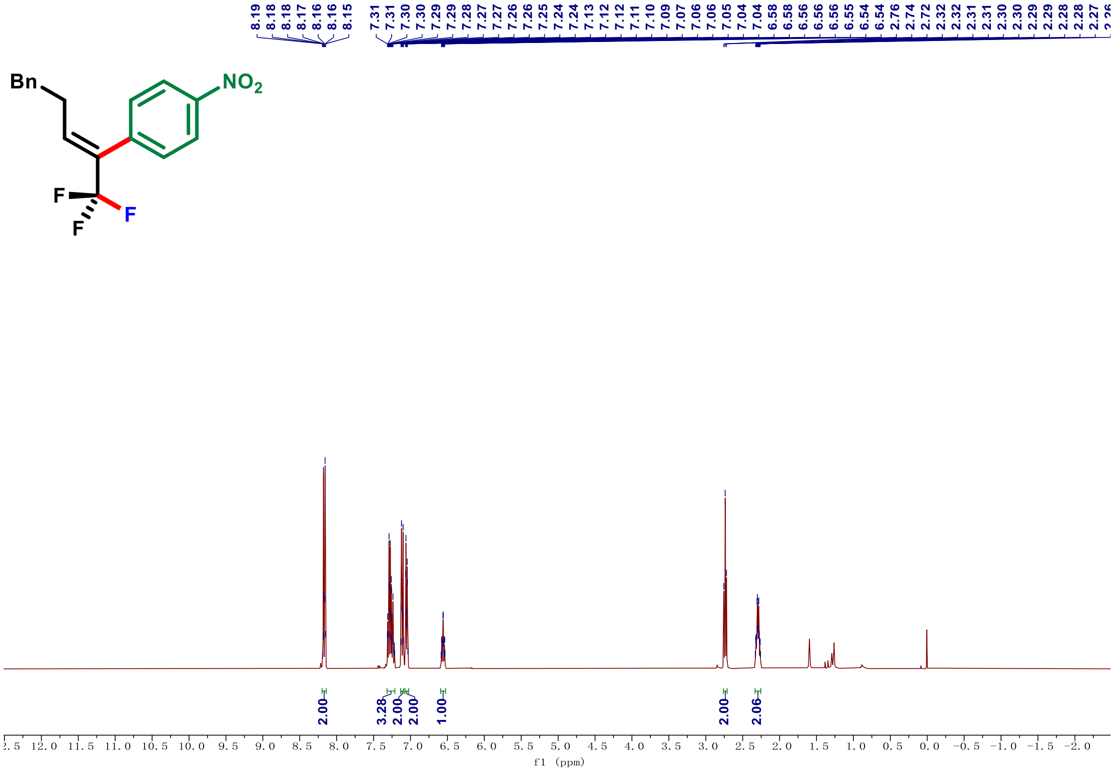

^{19}F NMR Spectrum of **3ae** ($CDCl_3$, 376 MHz)

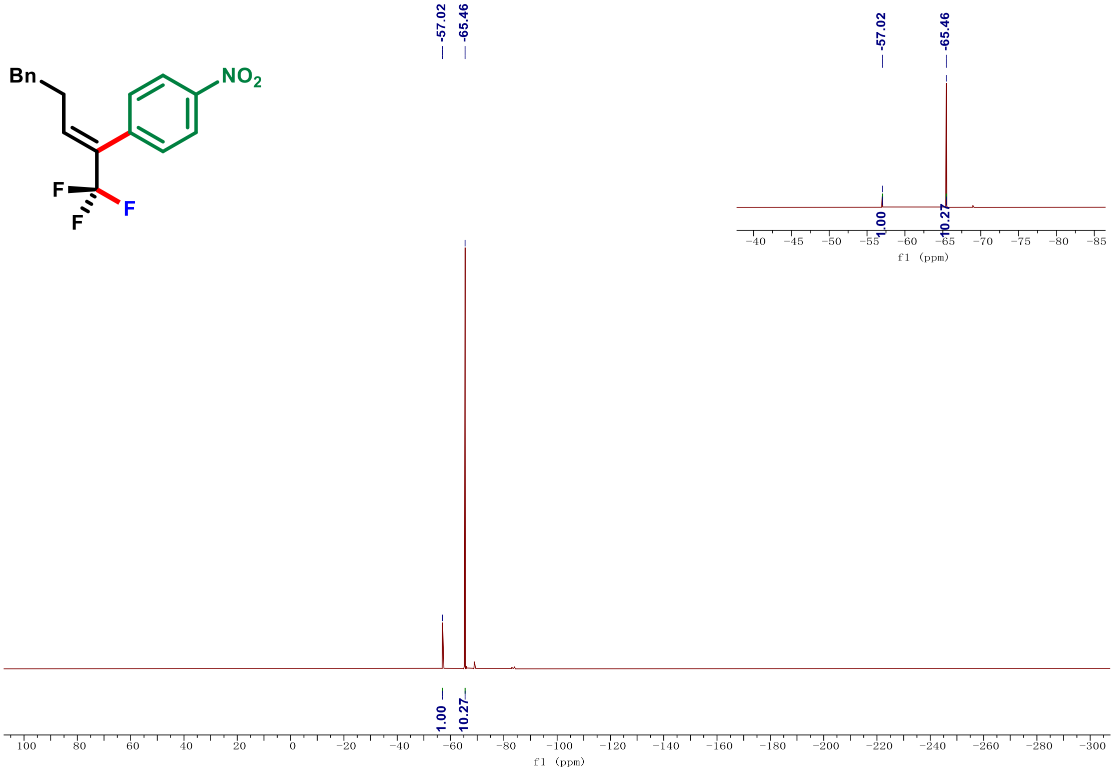

^{13}C NMR Spectrum of **3ae** ($CDCl_3$, 101 MHz)

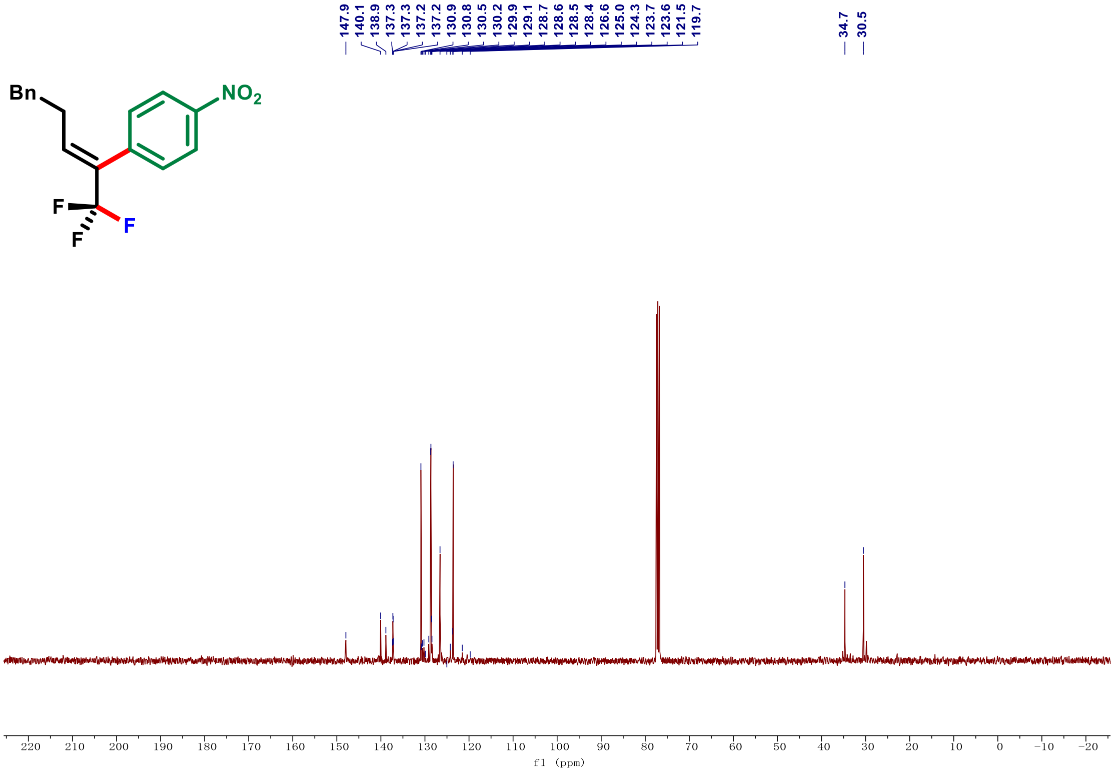

^{1}H NMR Spectrum of **3af** ($CDCl_3$, 400 MHz)

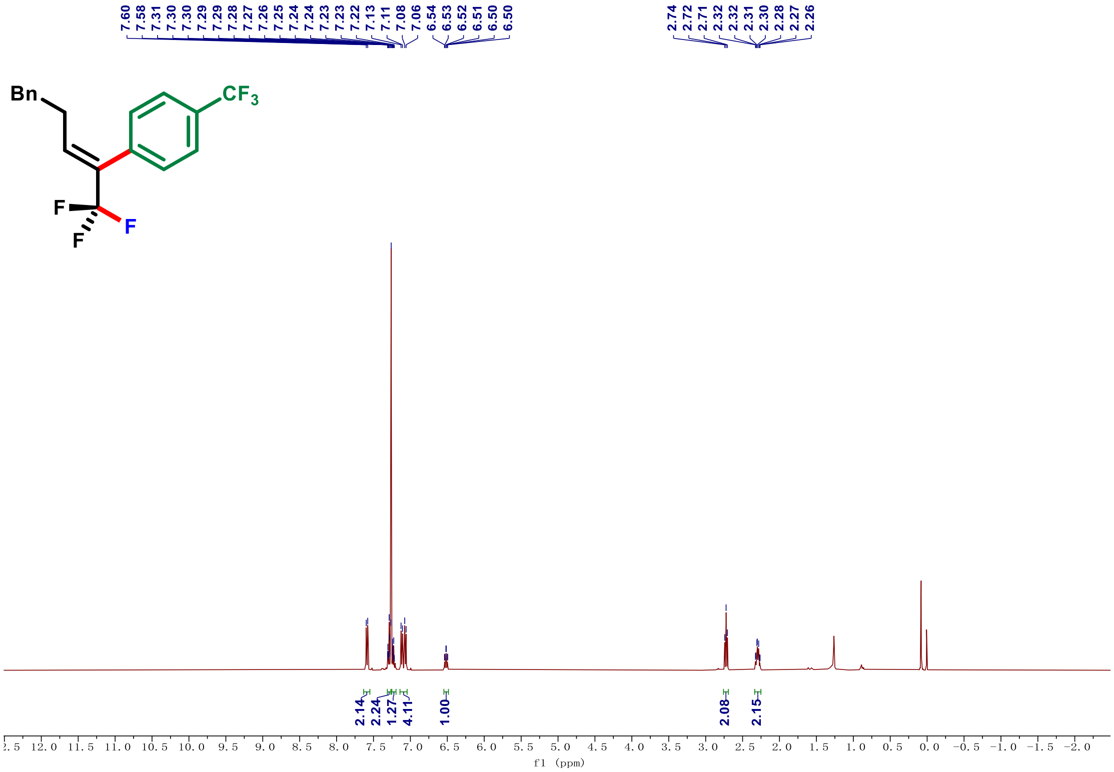

^{19}F NMR Spectrum of **3af** ($CDCl_3$, 376 MHz)

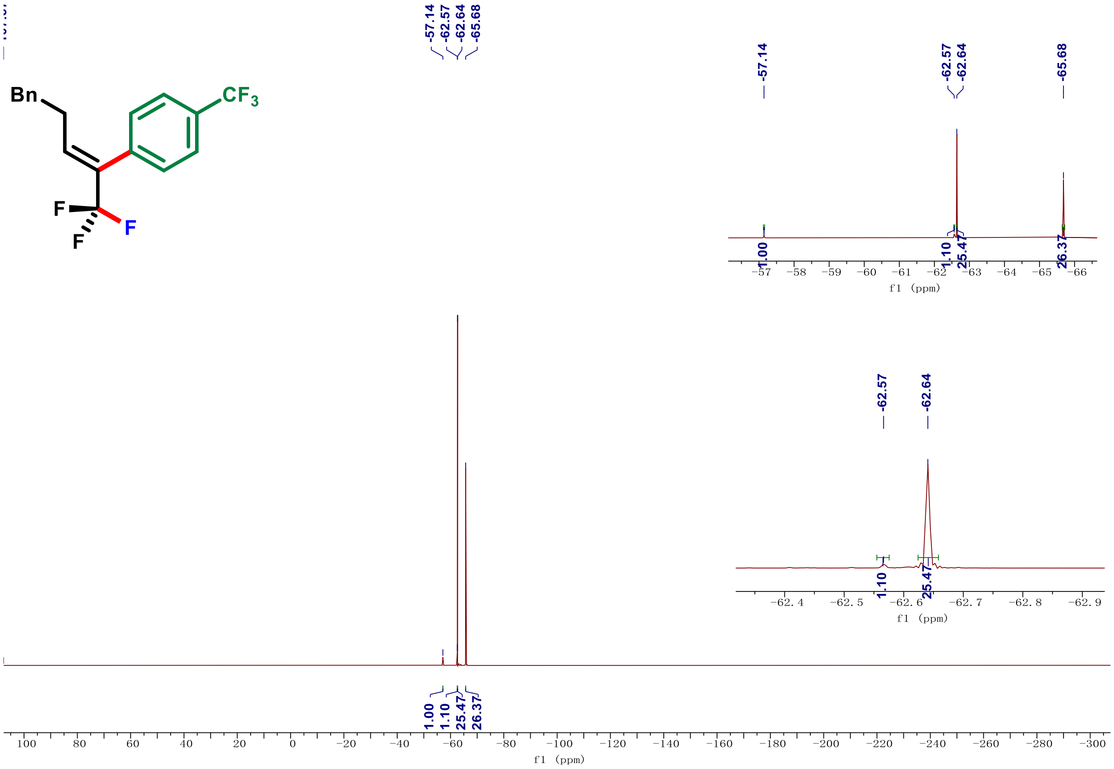

^{13}C NMR Spectrum of **3af** ($CDCl_3$, 101 MHz)

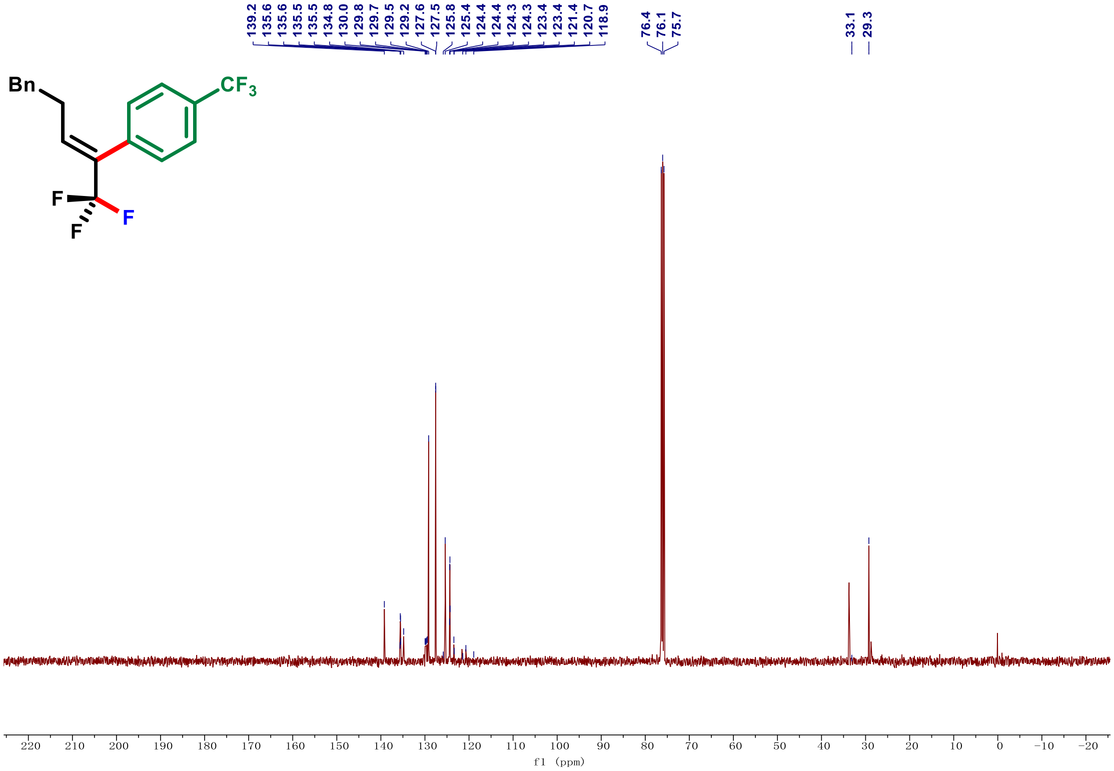

^{1}H NMR Spectrum of **3ag** ($CDCl_3$, 400 MHz)

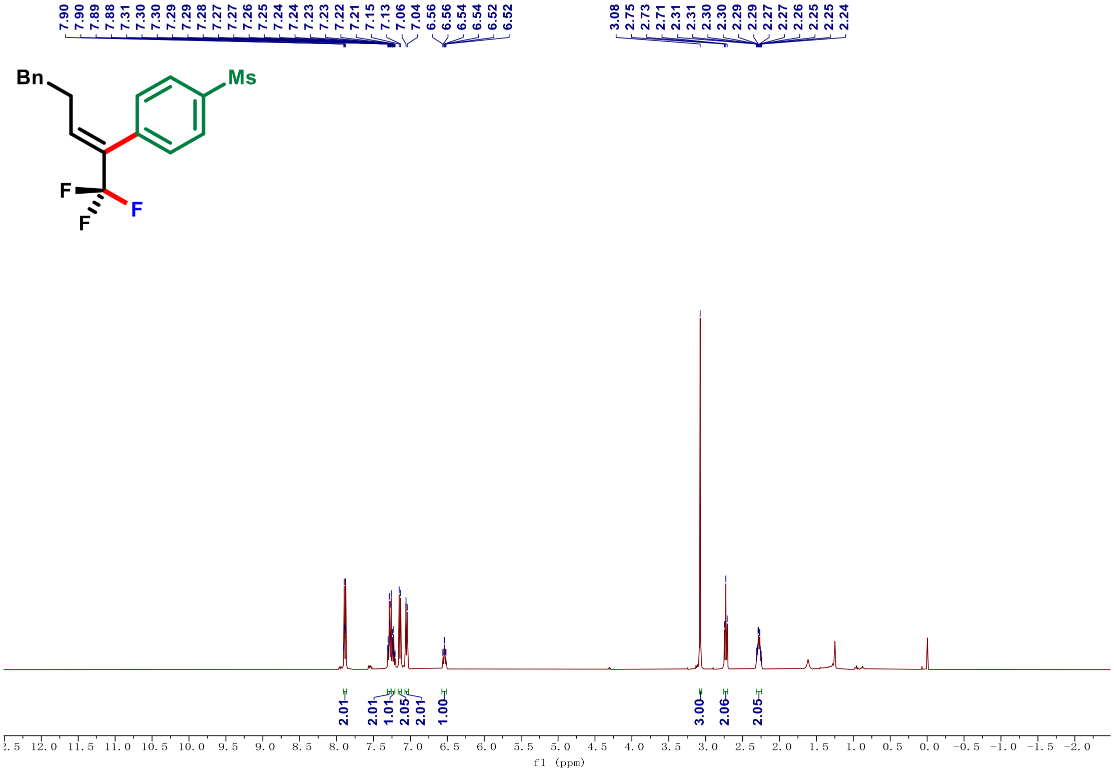

^{19}F NMR Spectrum of **3ag** ($CDCl_3$, 376 MHz)

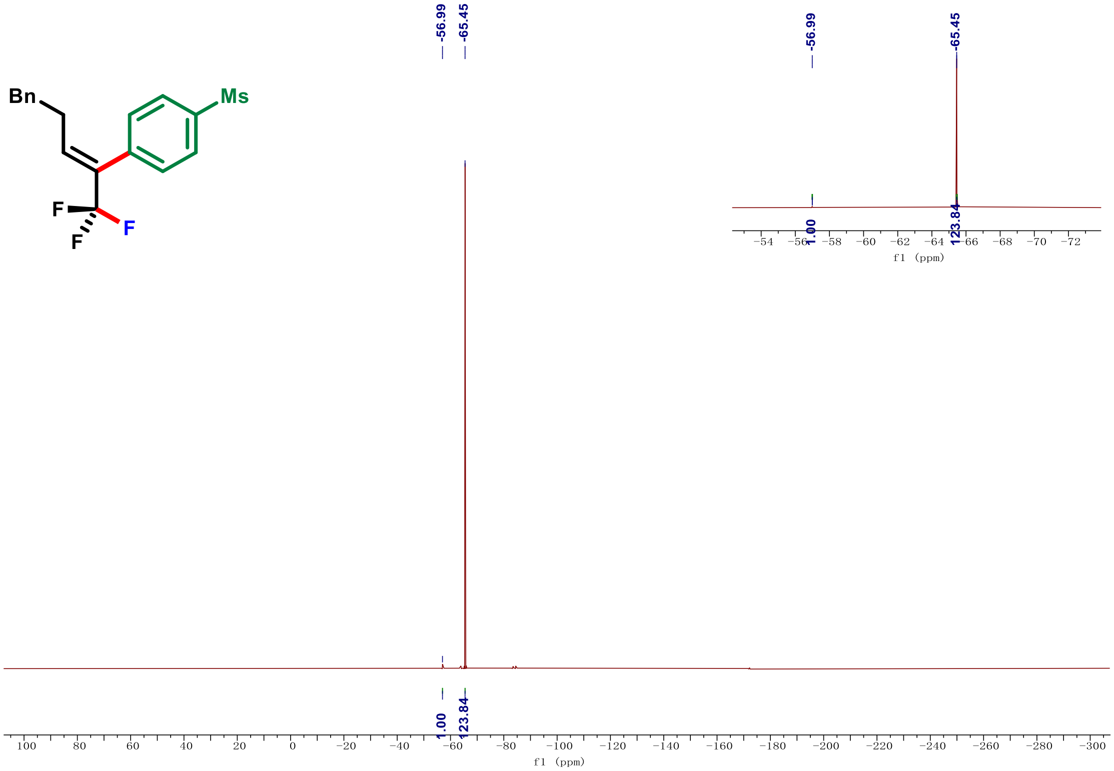

^{13}C NMR Spectrum of **3ag** ($CDCl_3$, 101 MHz)

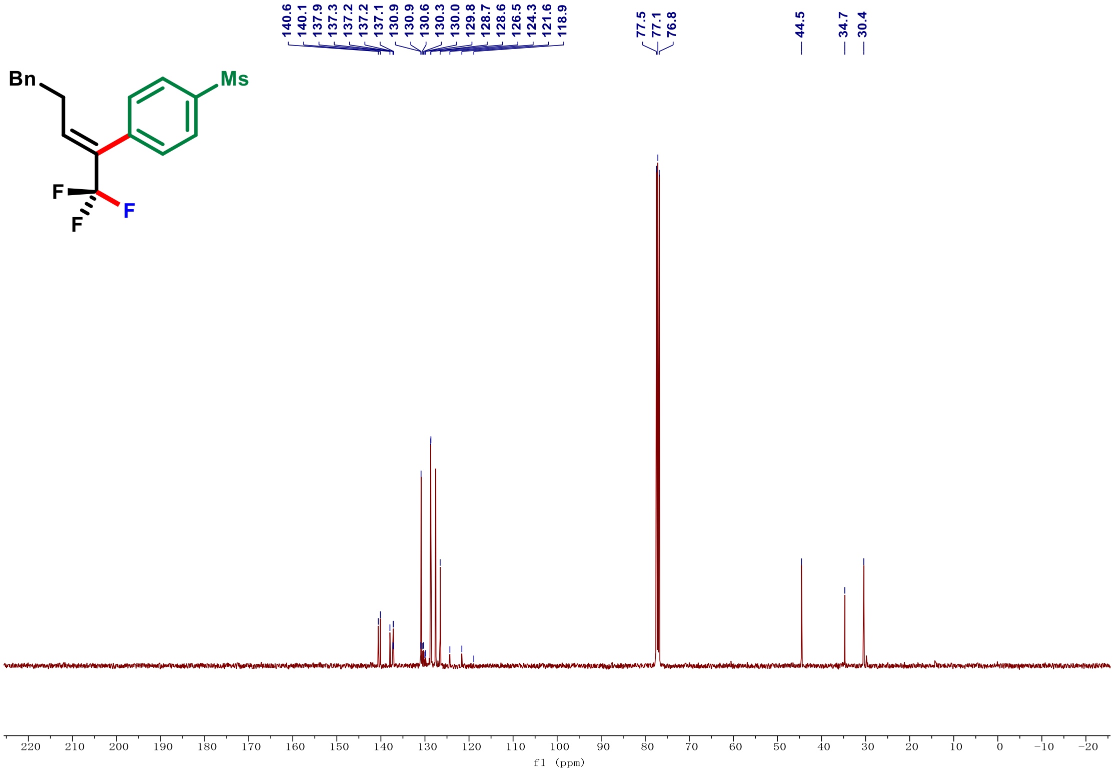

^{1}H NMR Spectrum of **3ah** ($CDCl_3$, 400 MHz)

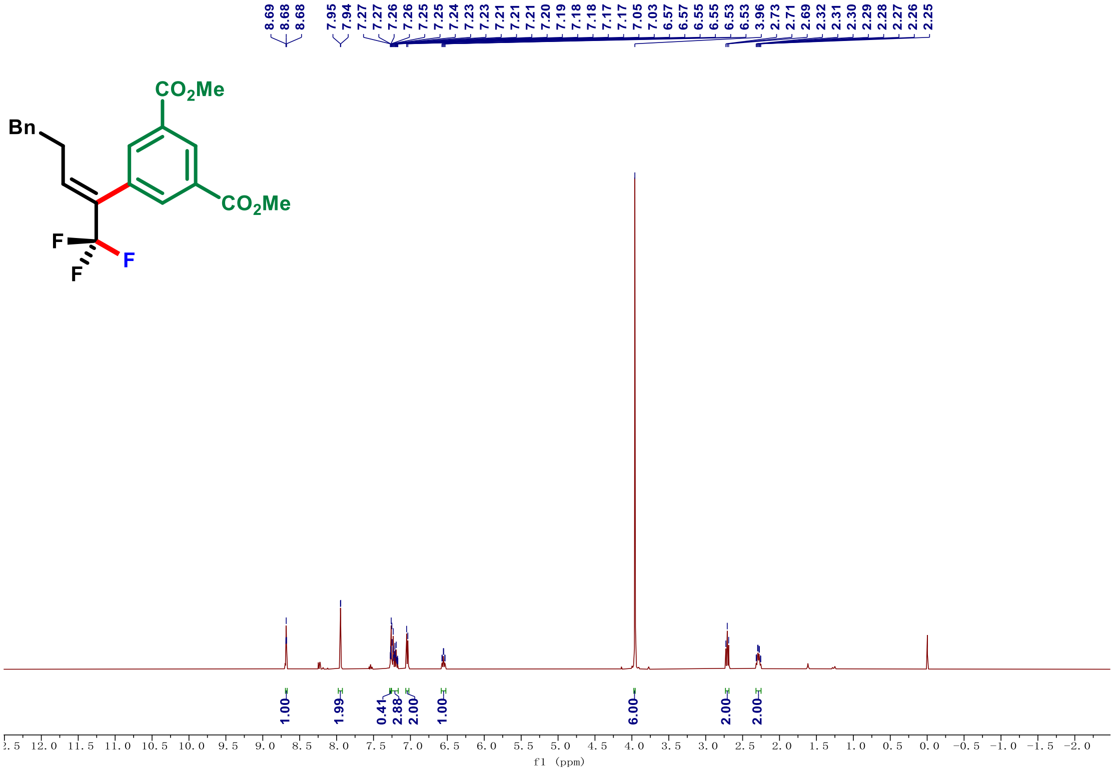

^{19}F NMR Spectrum of **3ah** ($CDCl_3$, 376 MHz)

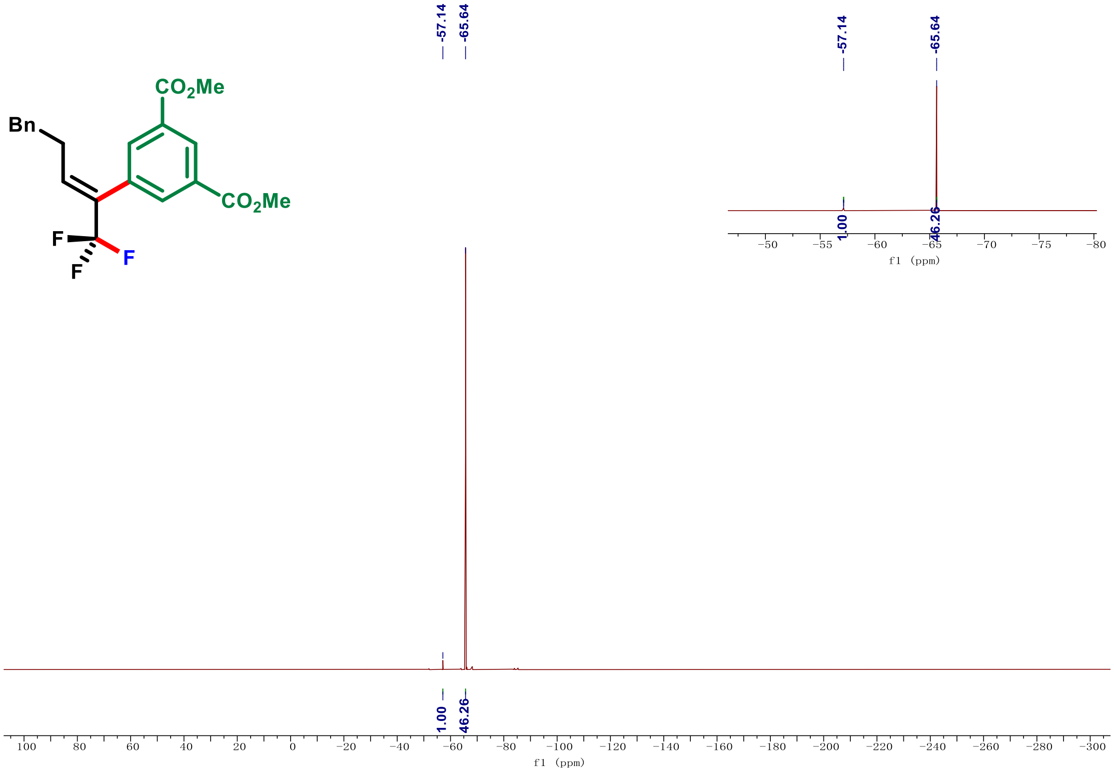

^{13}C NMR Spectrum of **3ah** ($CDCl_3$, 101 MHz)

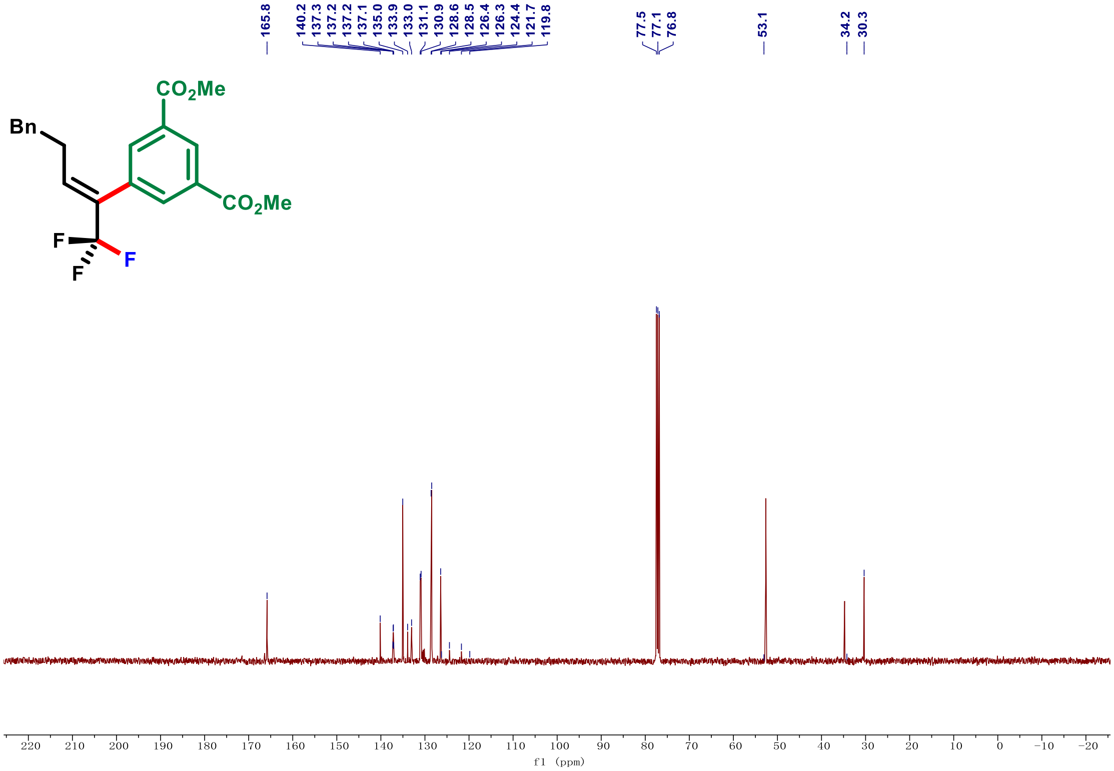

^{1}H NMR Spectrum of **3ai** ($CDCl_3$, 400 MHz)

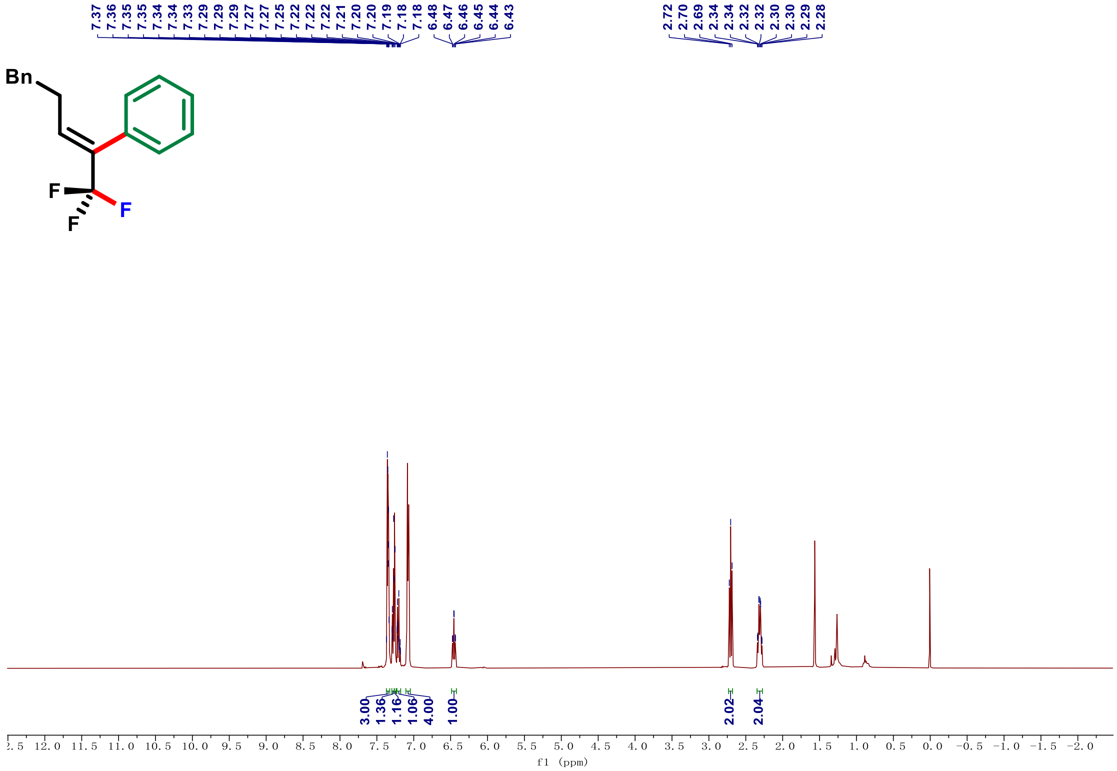

^{19}F NMR Spectrum of **3ai** ($CDCl_3$, 376 MHz)

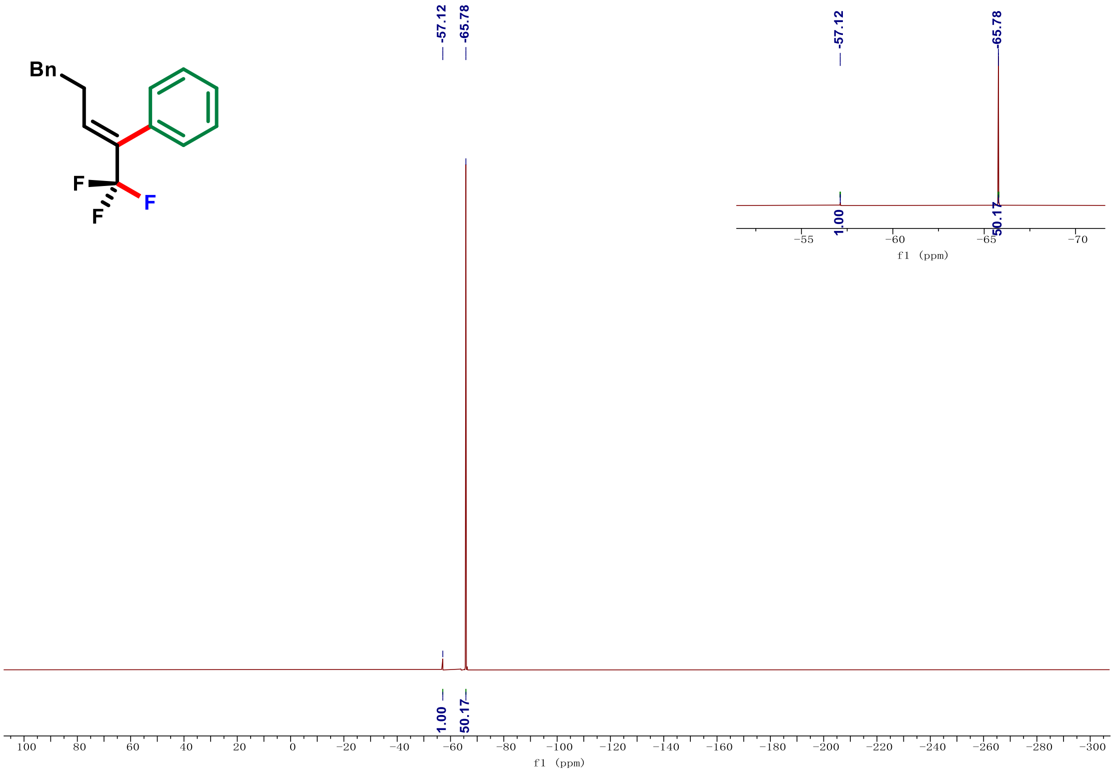

^{13}C NMR Spectrum of **3ai** ($CDCl_3$, 101 MHz)

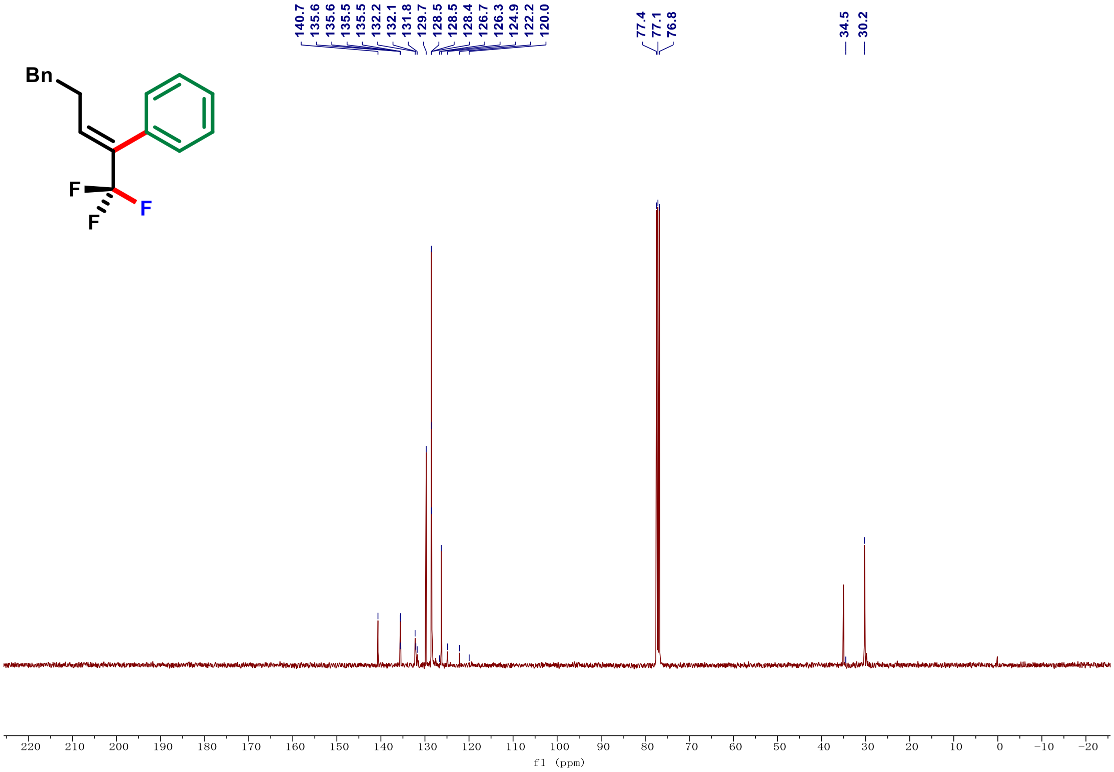

^{1}H NMR Spectrum of **3aj** ($CDCl_3$, 400 MHz)

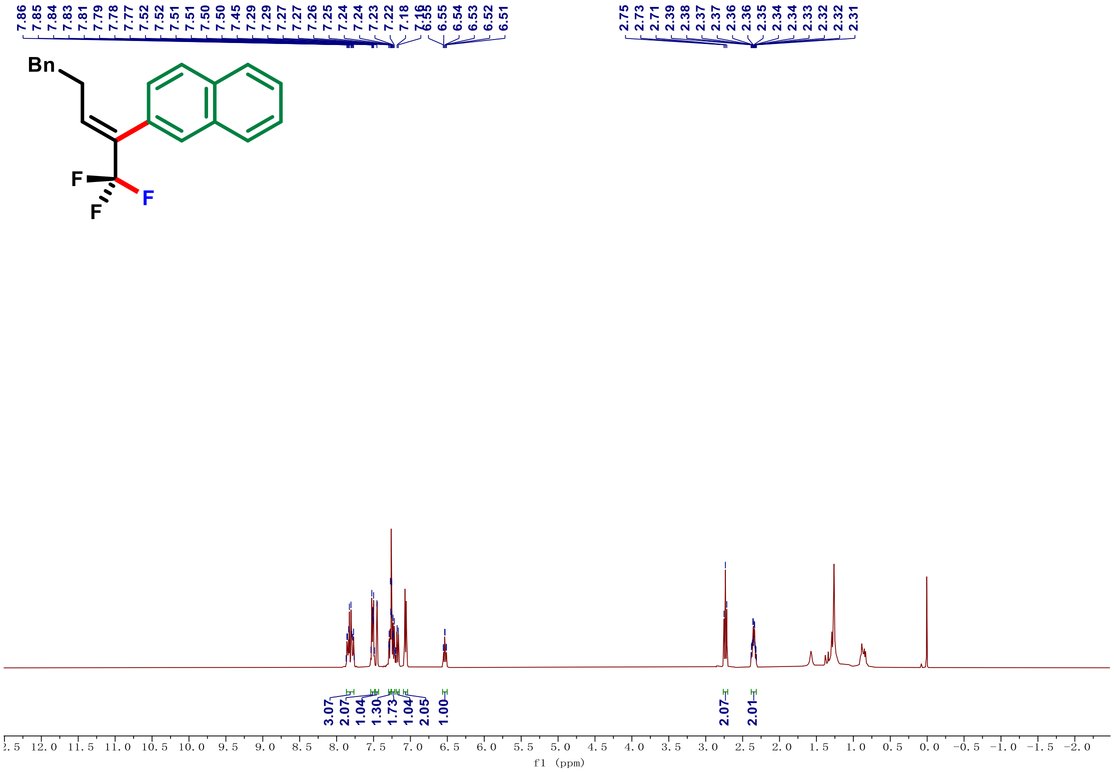

^{19}F NMR Spectrum of **3aj** ($CDCl_3$, 376 MHz)

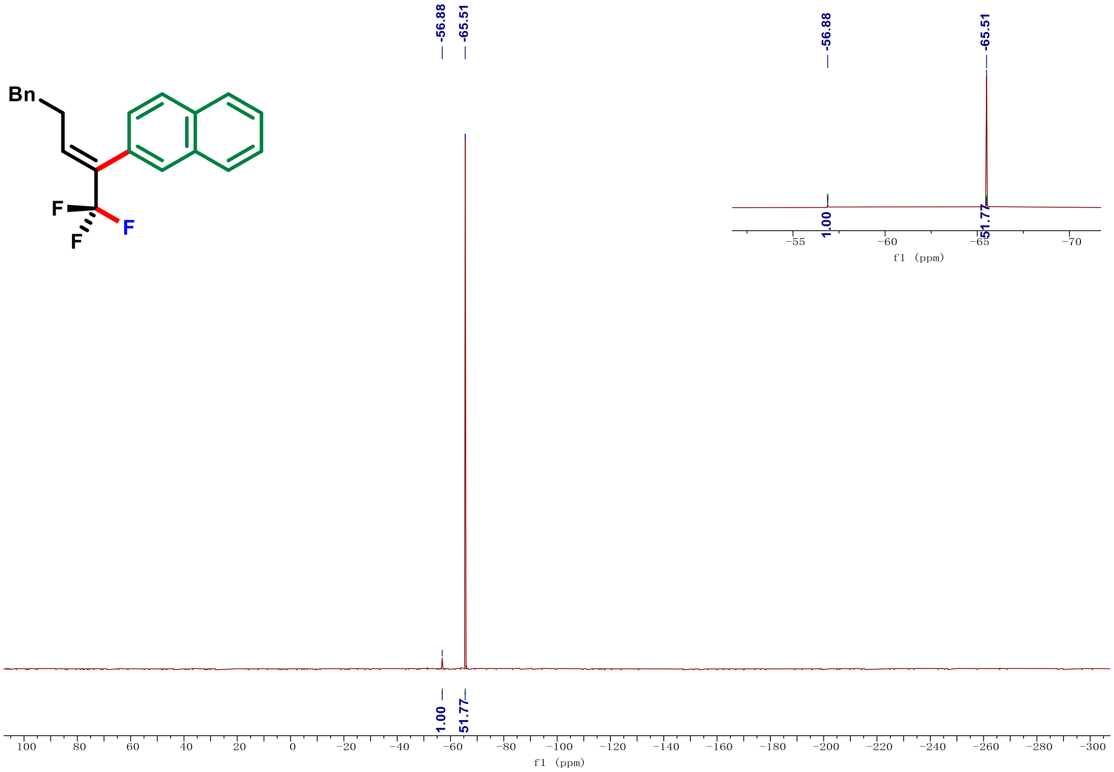

[13] C NMR Spectrum of **3aj** ($CDCl_3$, 101 MHz)

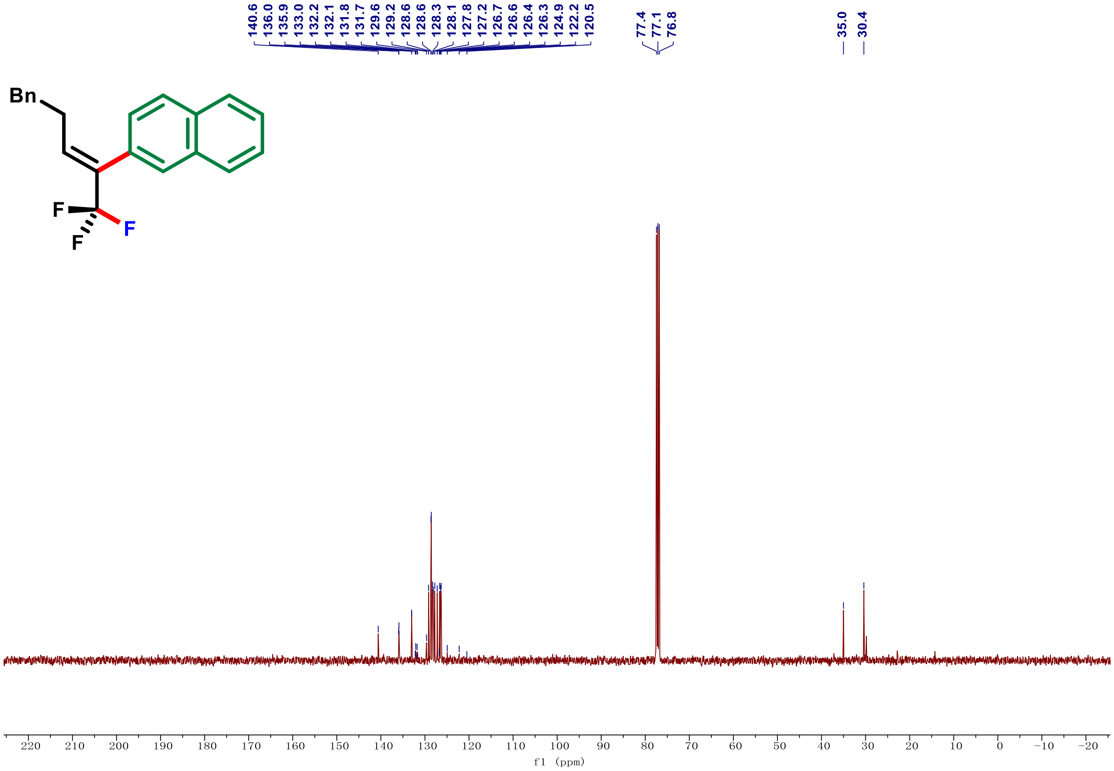

[1] H NMR Spectrum of **3ak** ($CDCl_3$, 400 MHz)

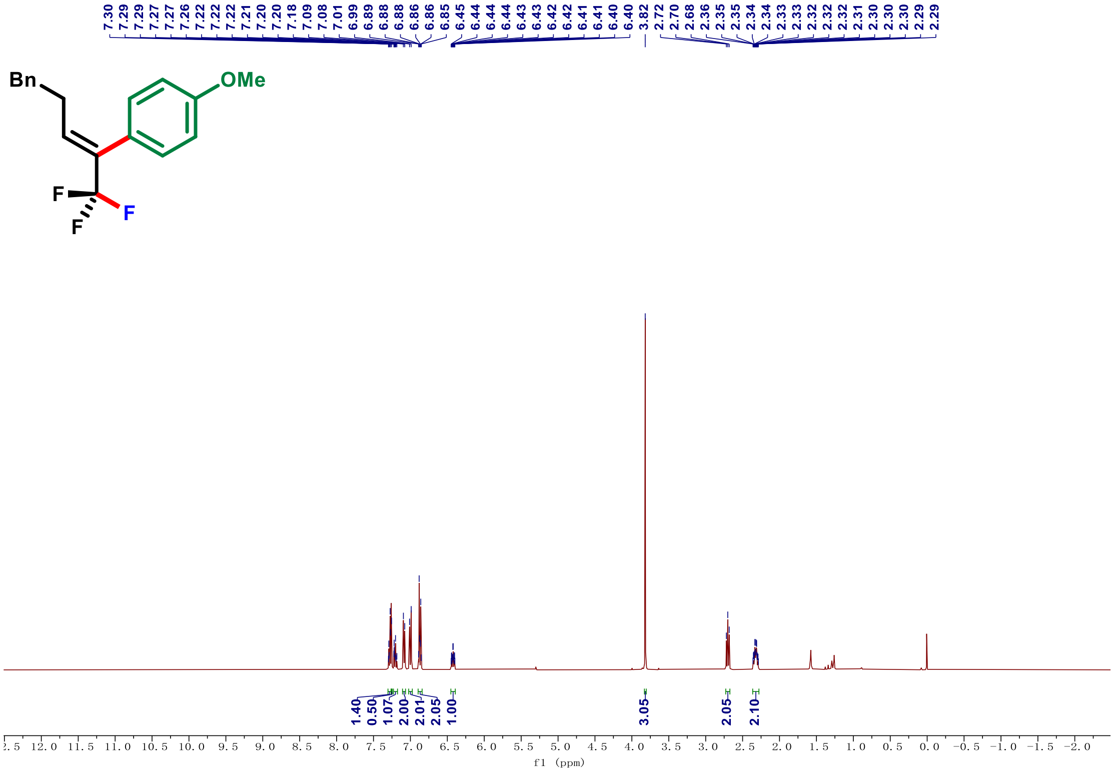

^{19}F NMR Spectrum of **3ak** ($CDCl_3$, 376 MHz)

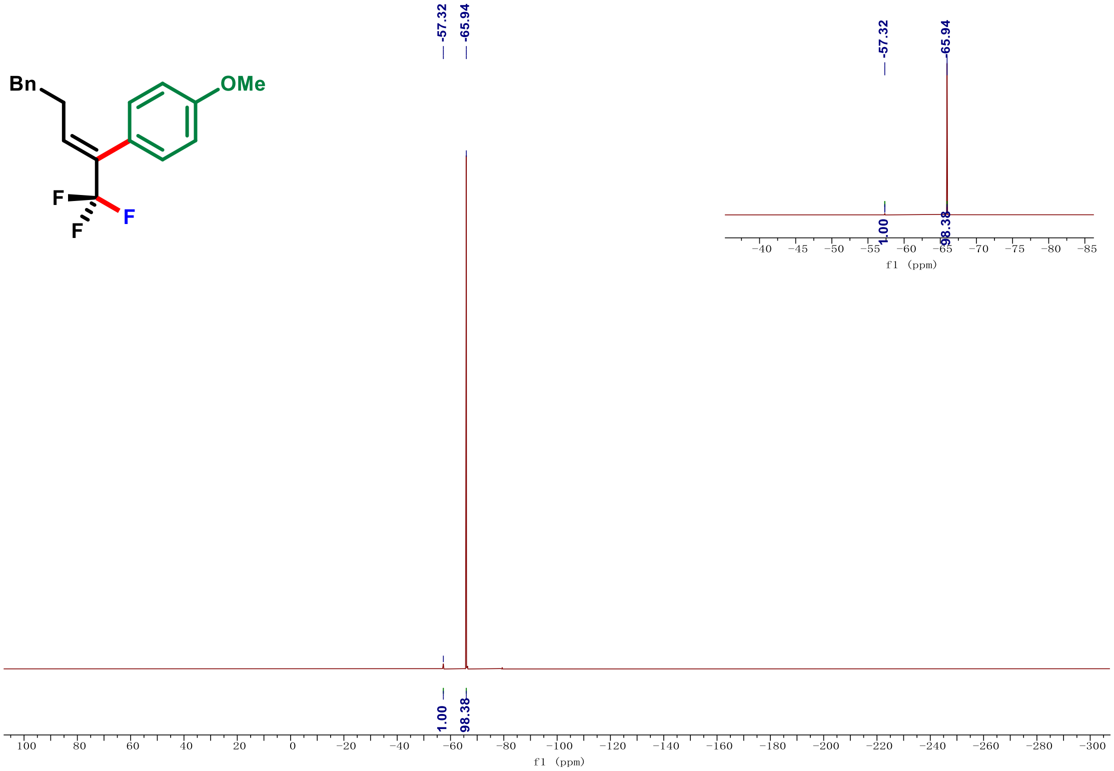

^{13}C NMR Spectrum of **3ak** ($CDCl_3$, 101 MHz)

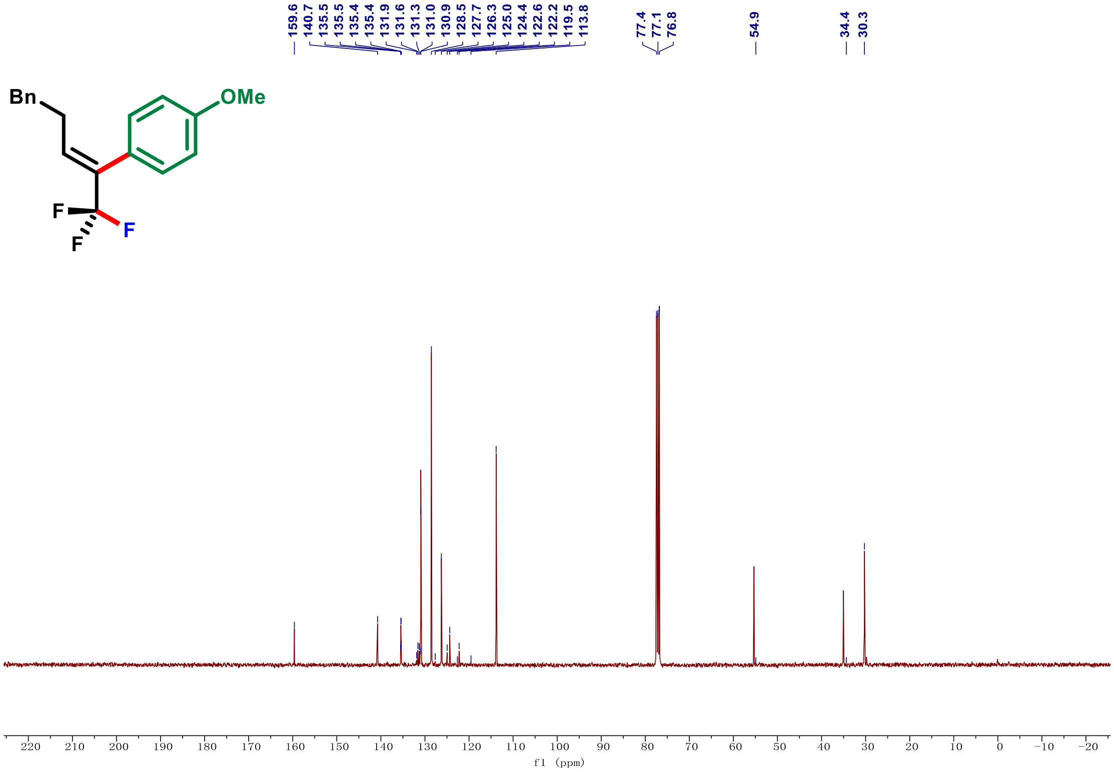

^{1}H NMR Spectrum of **3al** ($CDCl_3$, 400 MHz)

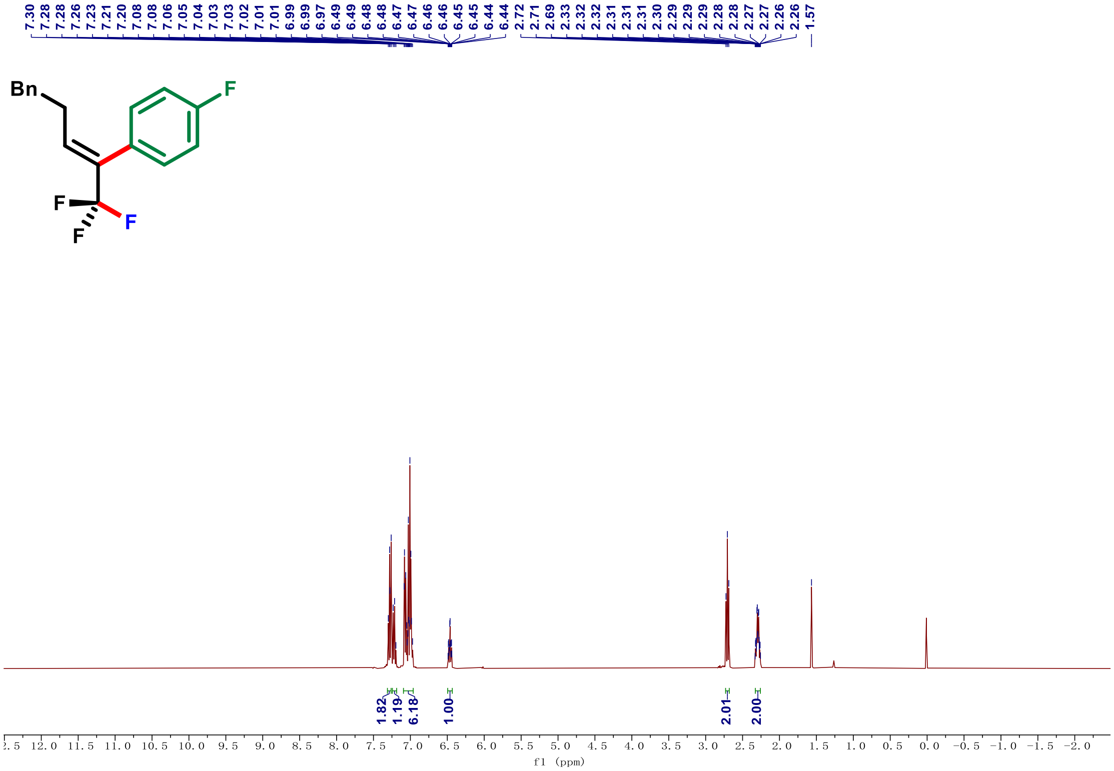

^{19}F NMR Spectrum of **3al** ($CDCl_3$, 376 MHz)

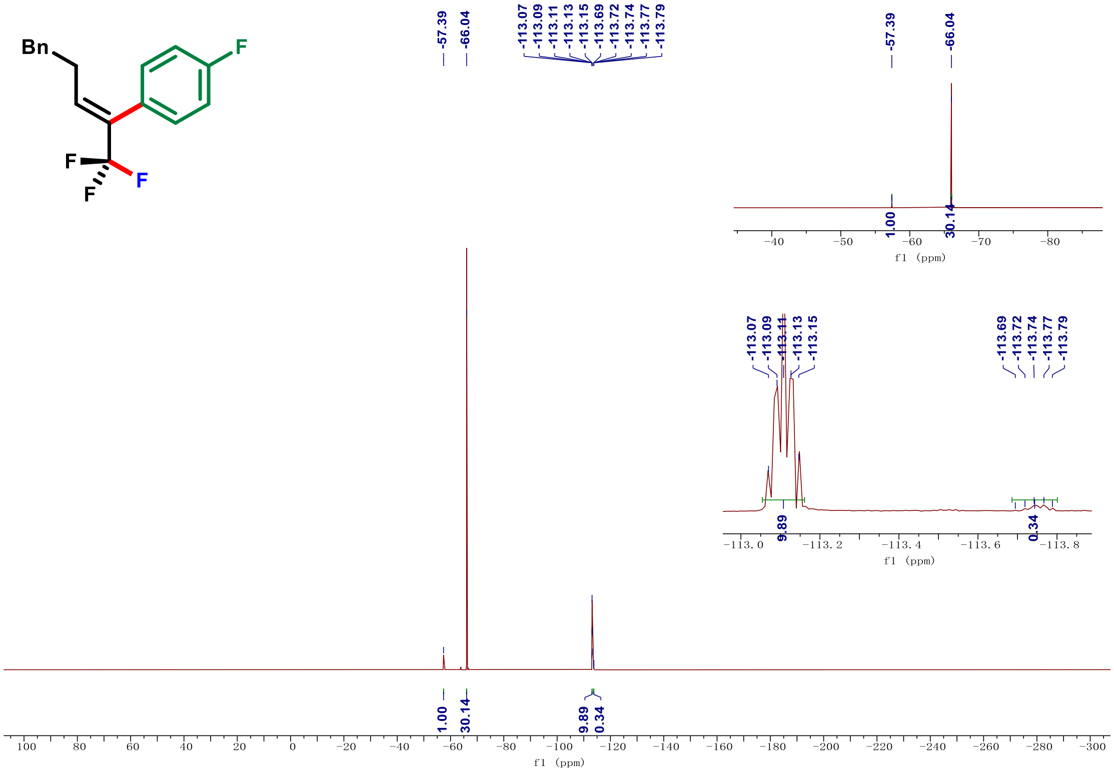

^{13}C NMR Spectrum of **3al** ($CDCl_3$, 101 MHz)

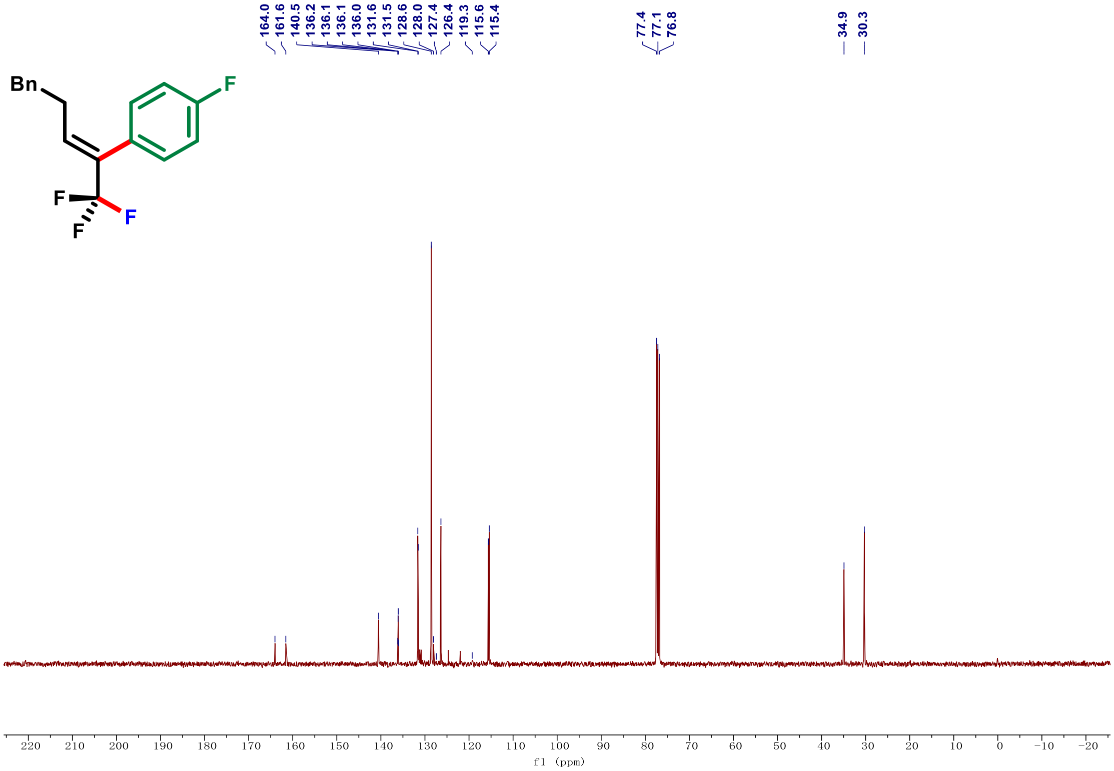

^{1}H NMR Spectrum of **3am** ($CDCl_3$, 400 MHz)

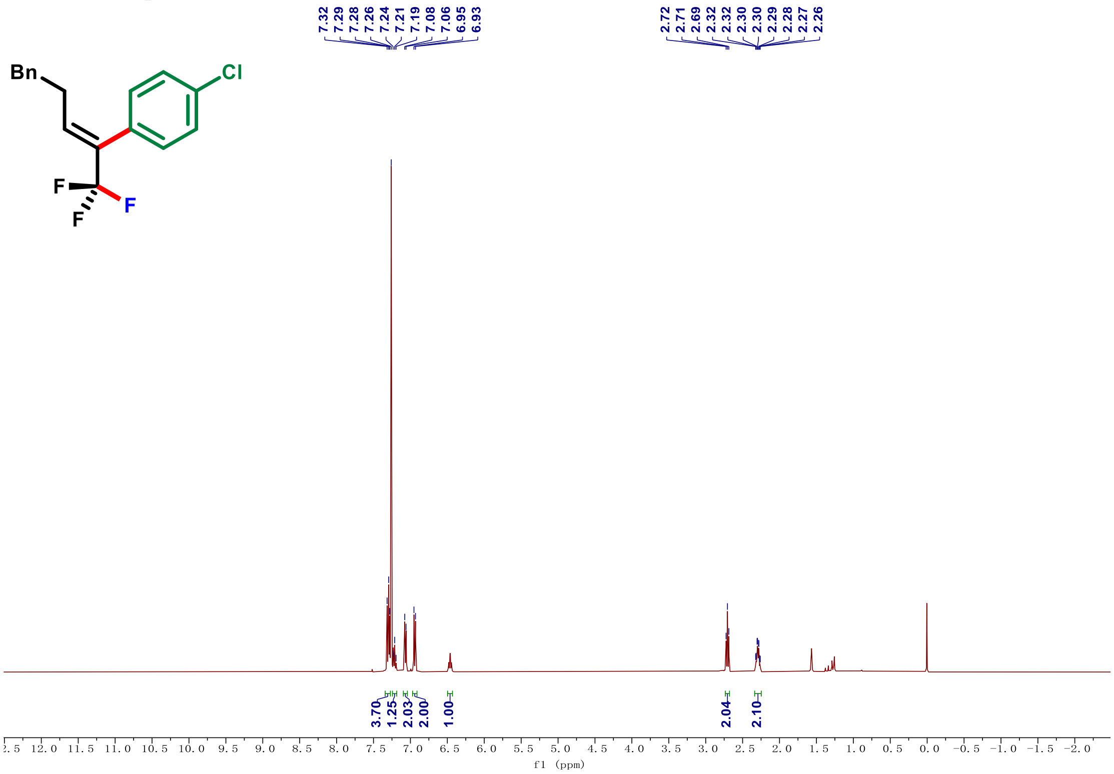

^{19}F NMR Spectrum of **3am** ($CDCl_3$, 376 MHz)

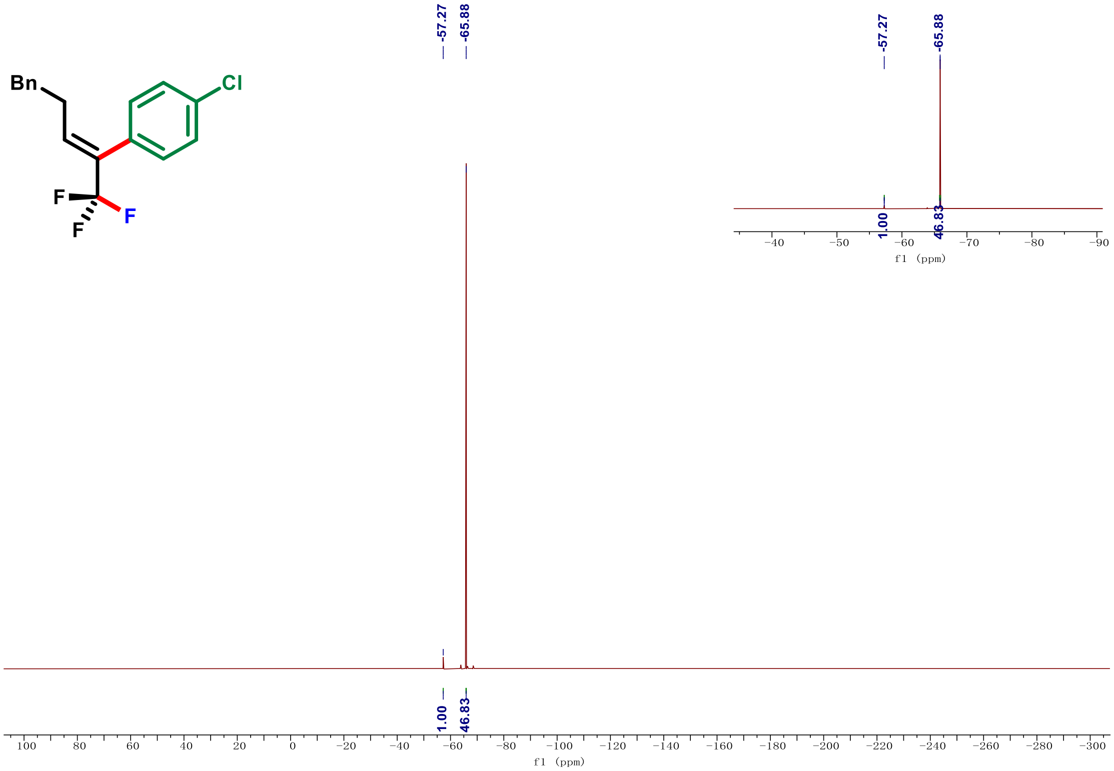

^{13}C NMR Spectrum of **3am** ($CDCl_3$, 101 MHz)

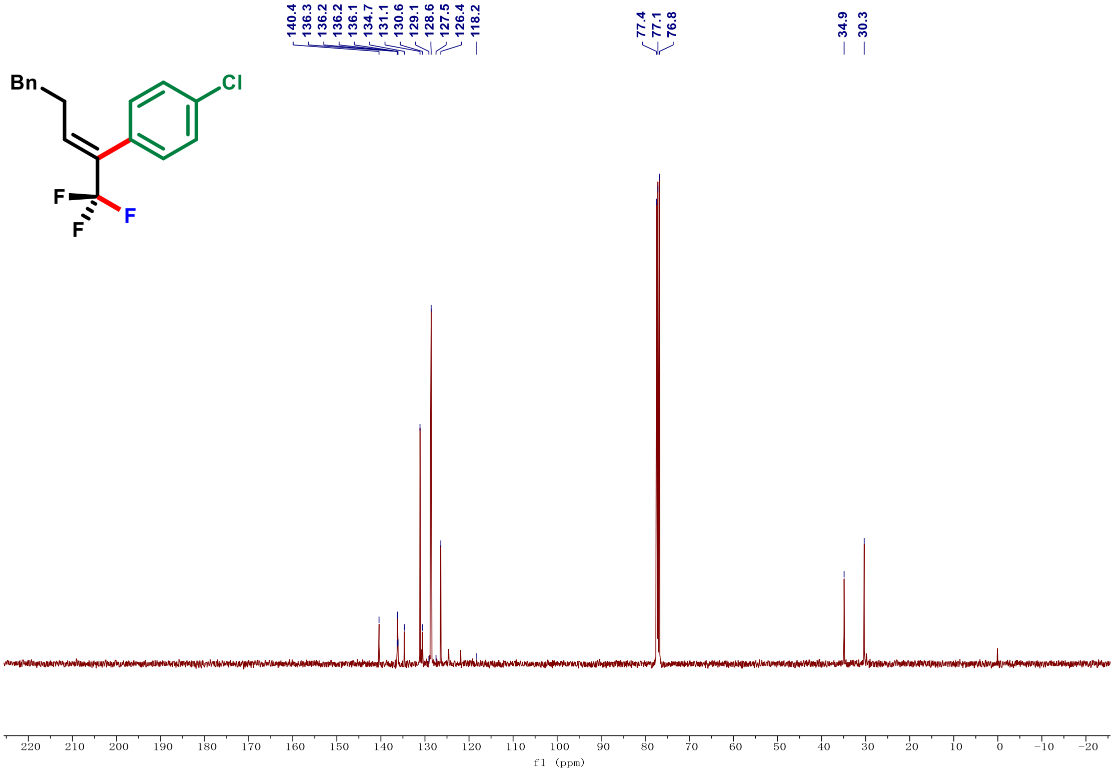

^{1}H NMR Spectrum of **3an** ($CDCl_3$, 400 MHz)

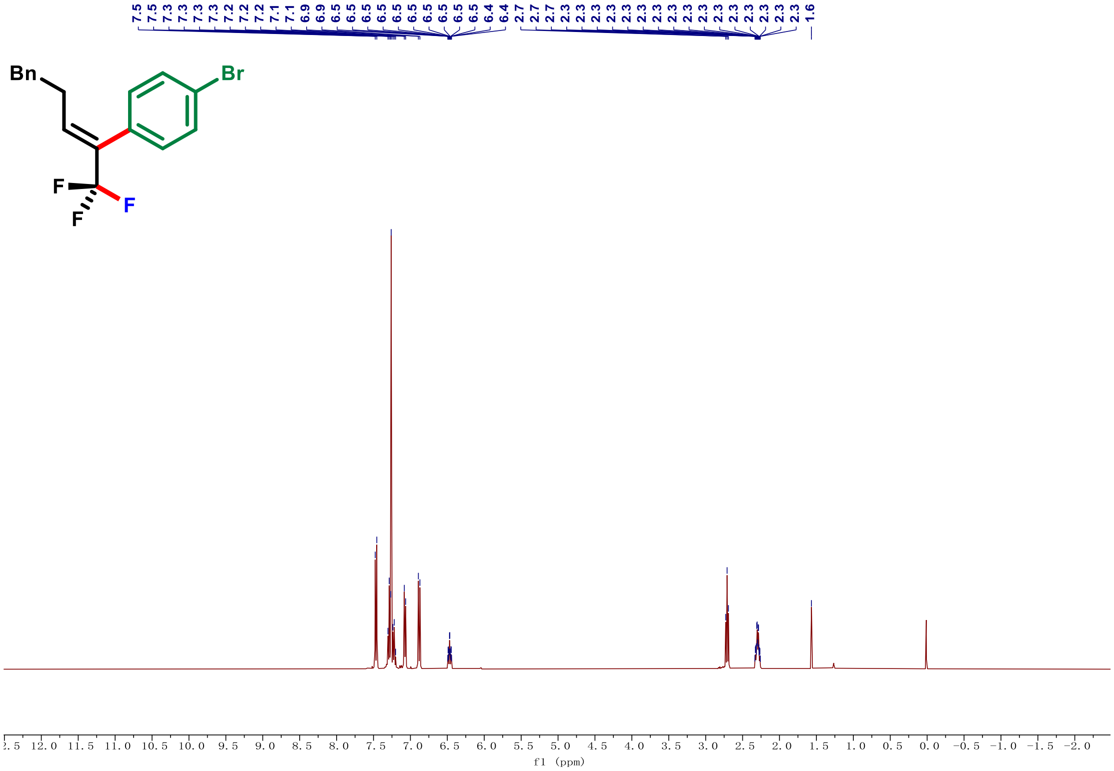

^{19}F NMR Spectrum of **3an** ($CDCl_3$, 376 MHz)

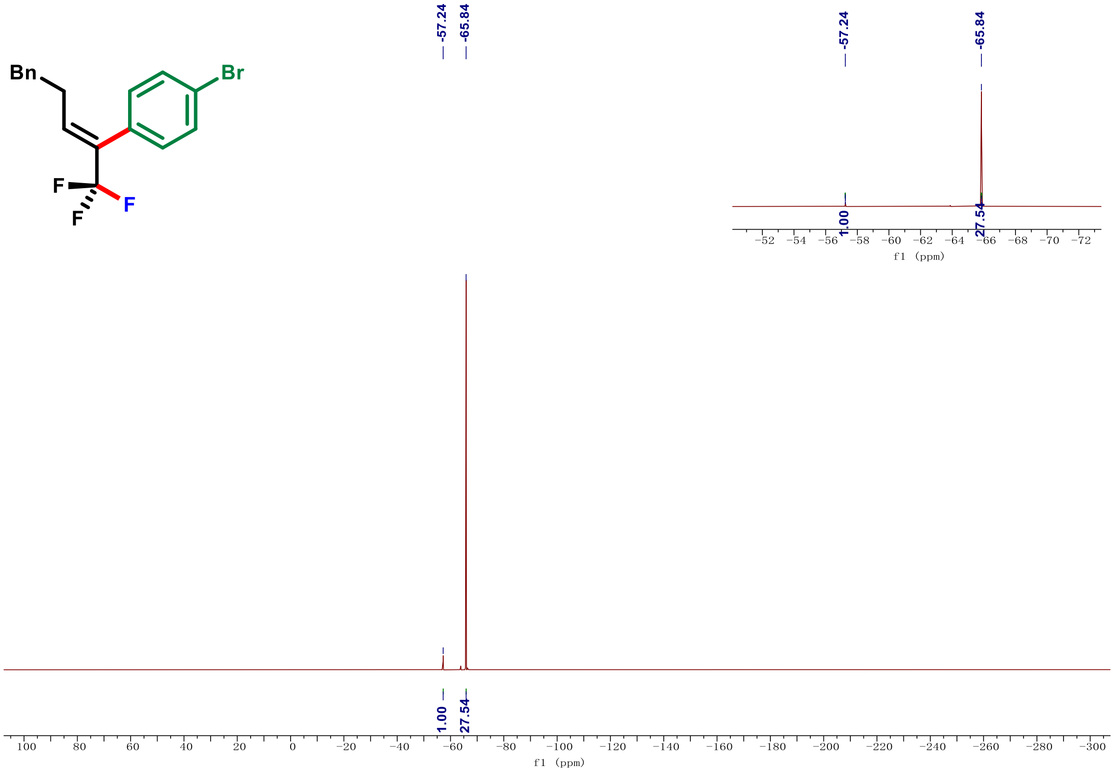

^{13}C NMR Spectrum of **3an** ($CDCl_3$, 101 MHz)

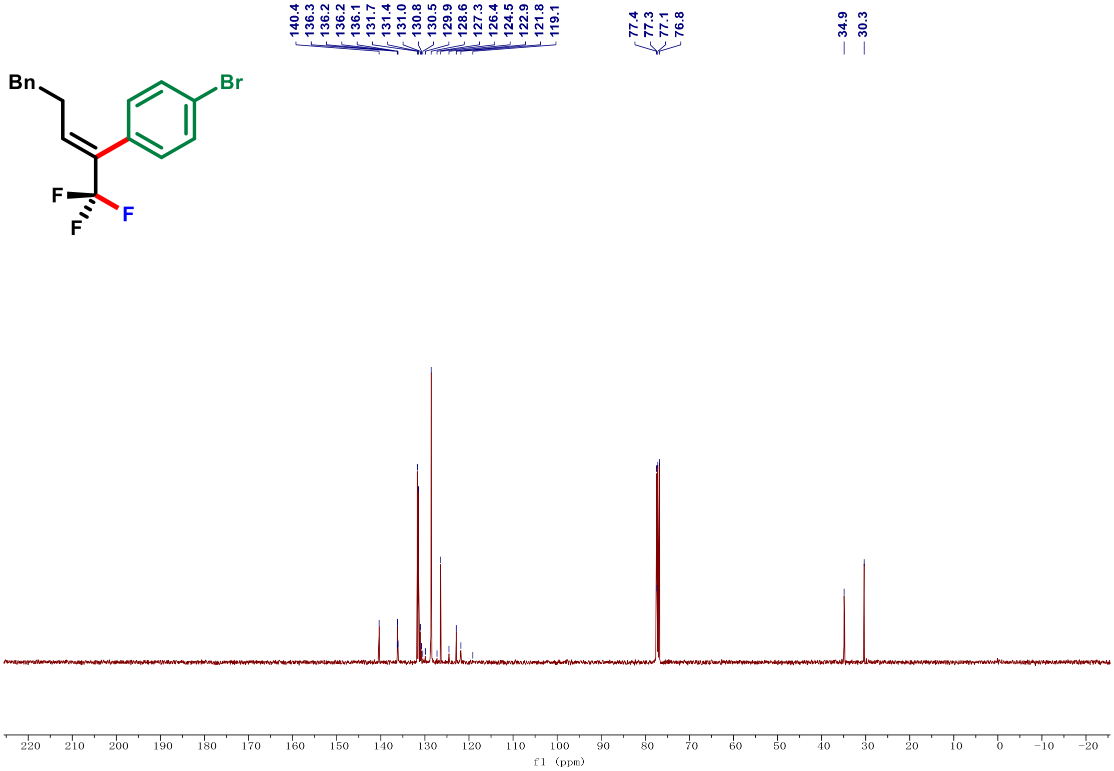

^{1}H NMR Spectrum of **3ao** ($CDCl_3$, 400 MHz)

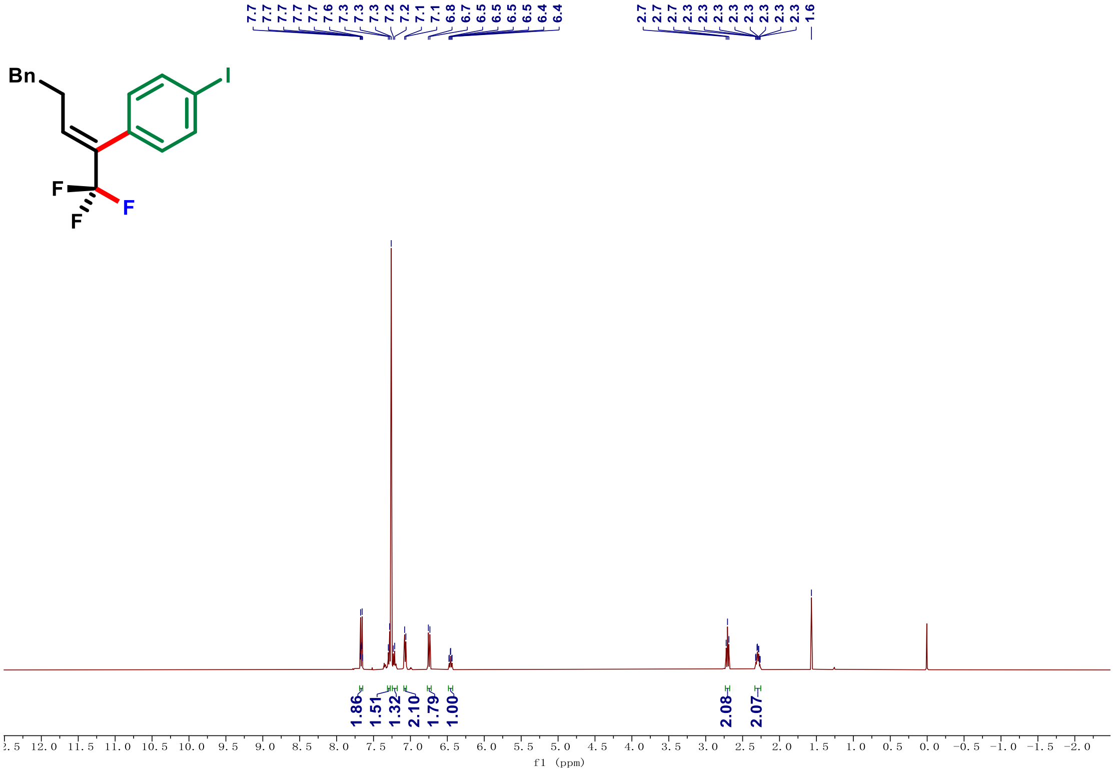

^{19}F NMR Spectrum of **3ao** ($CDCl_3$, 376 MHz)

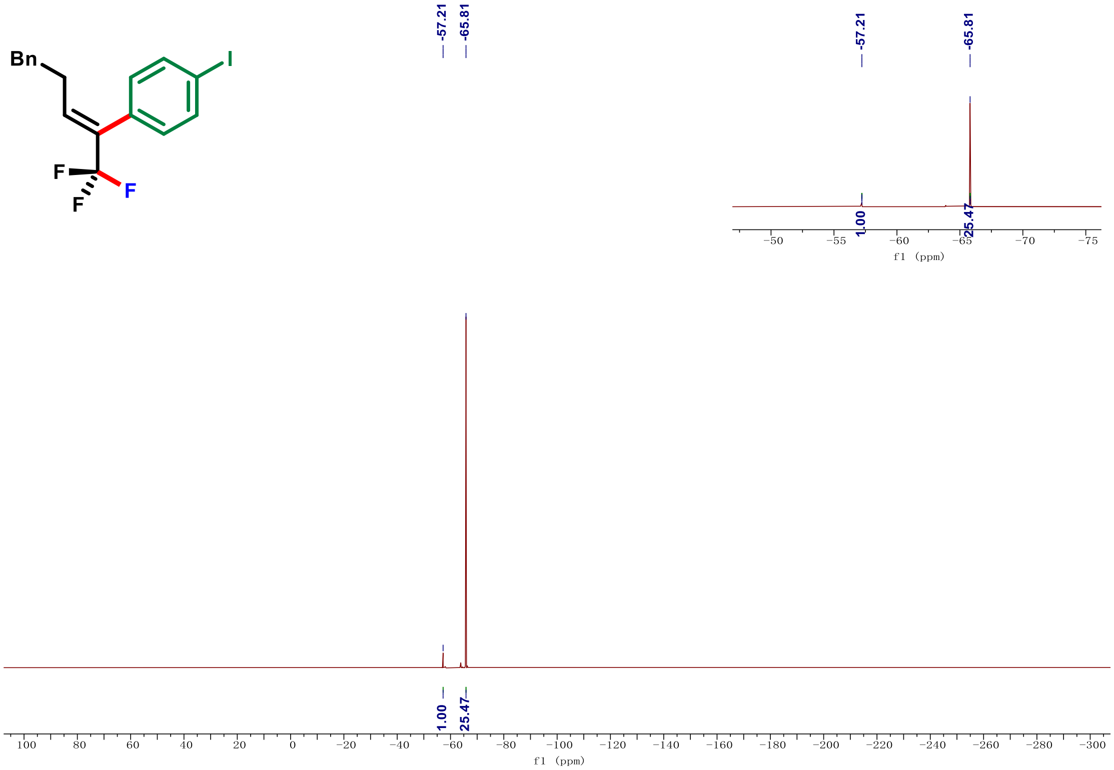

^{13}C NMR Spectrum of **3ao** ($CDCl_3$, 101 MHz)

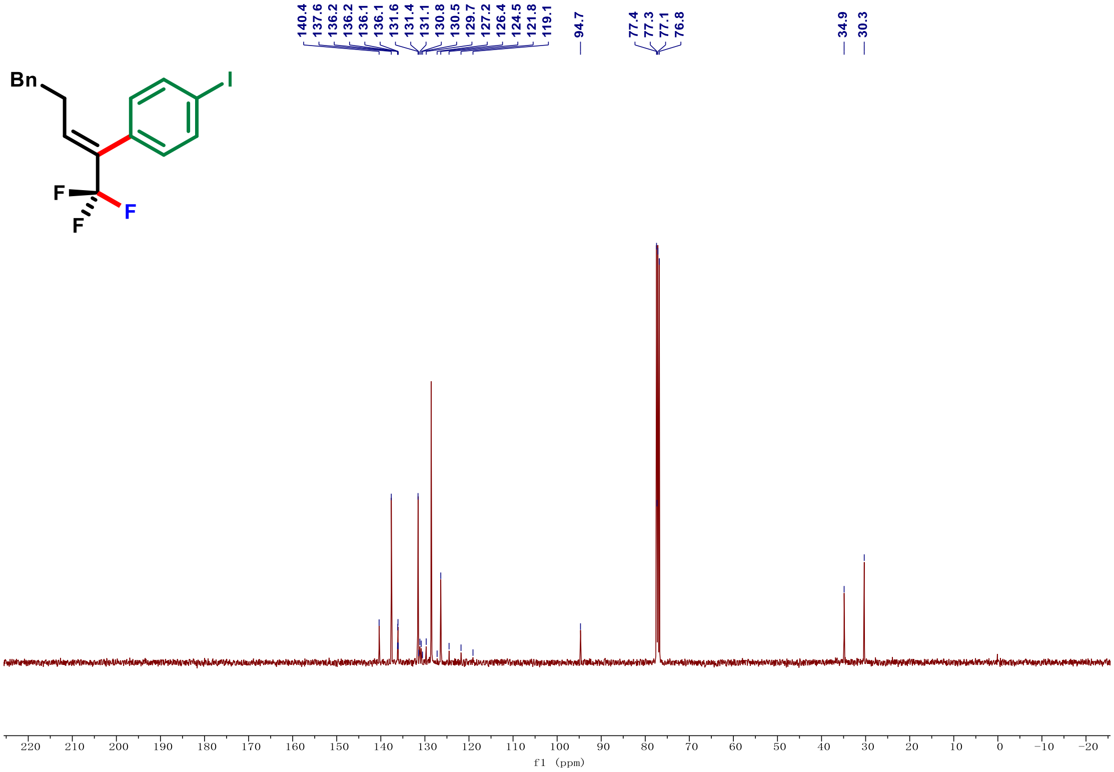

^{1}H NMR Spectrum of **3ap** ($CDCl_3$, 400 MHz)

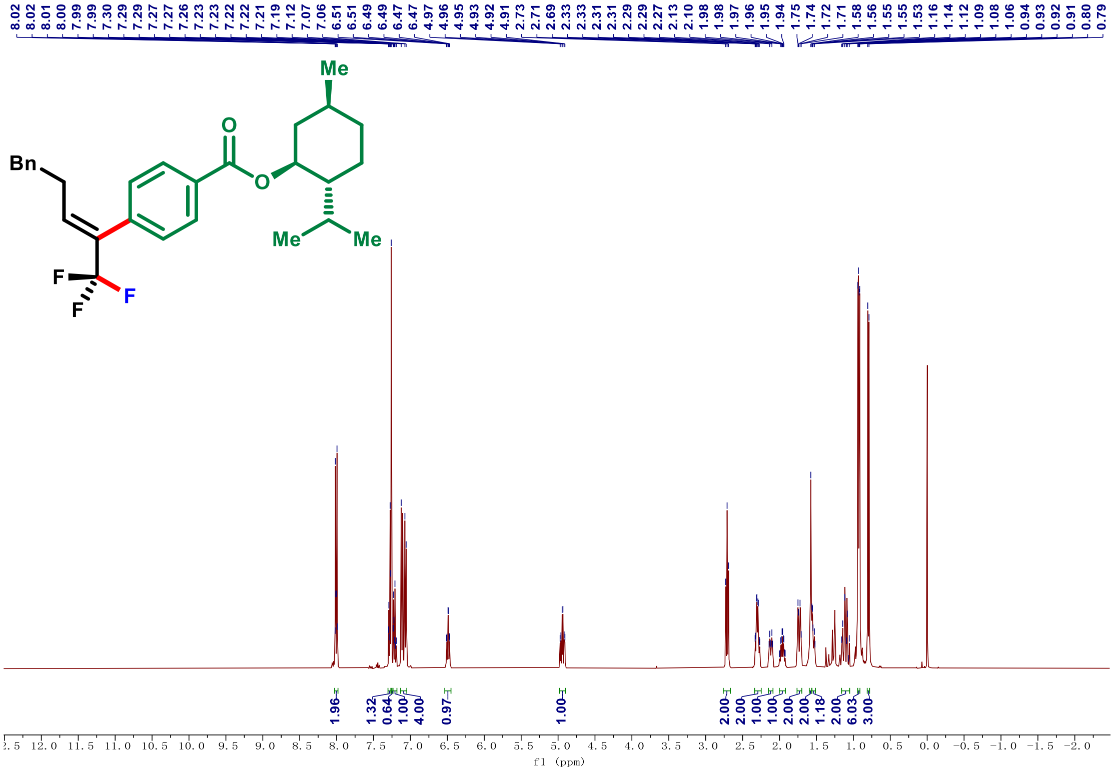

^{19}F NMR Spectrum of **3ap** ($CDCl_3$, 376 MHz)

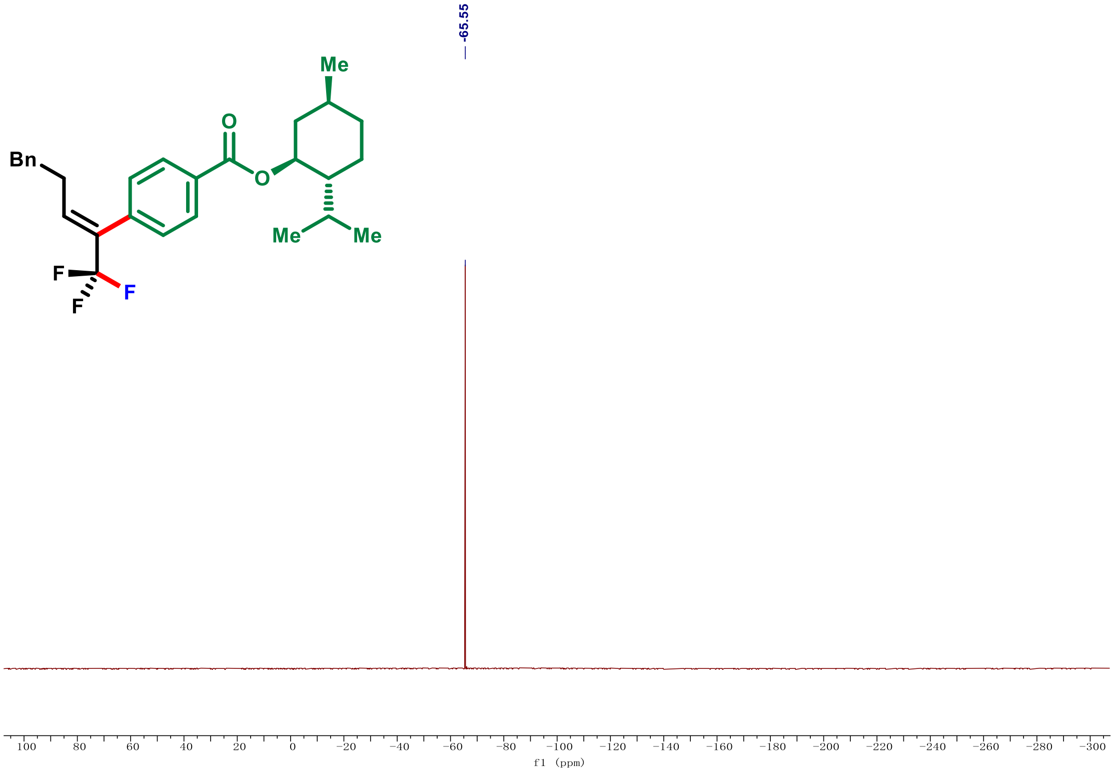

^{13}C NMR Spectrum of **3ap** ($CDCl_3$, 101 MHz)

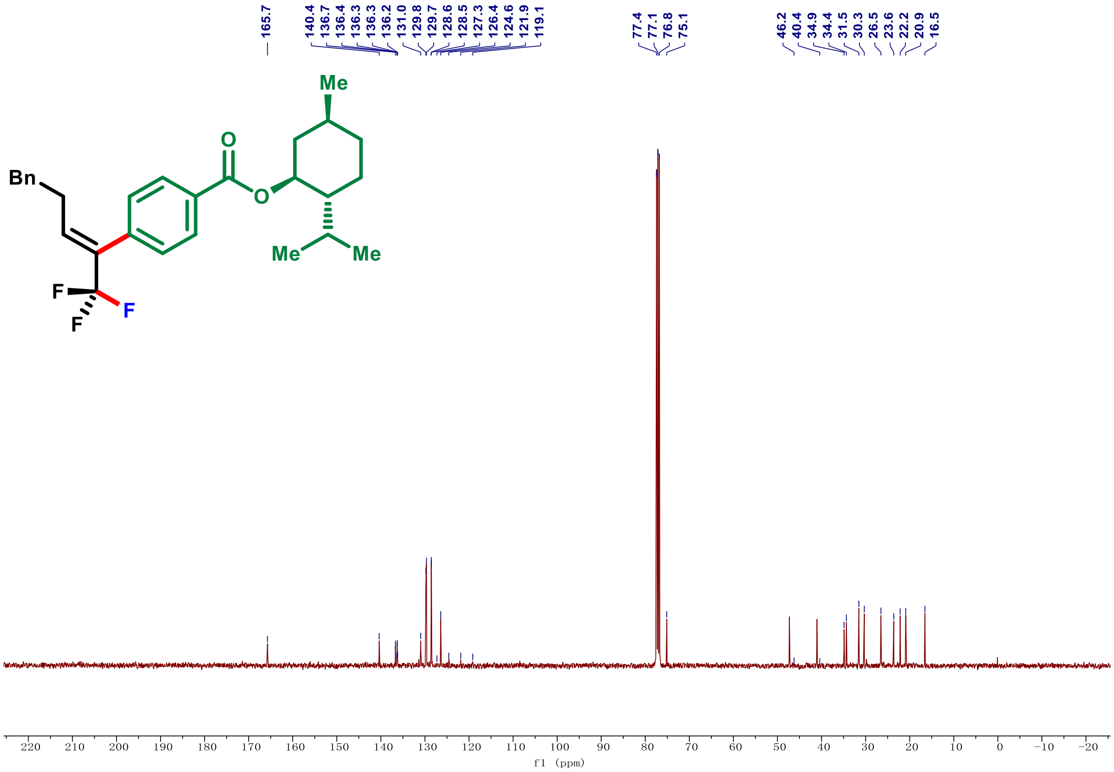

^{1}H NMR Spectrum of **3aq** ($CDCl_3$, 400 MHz)

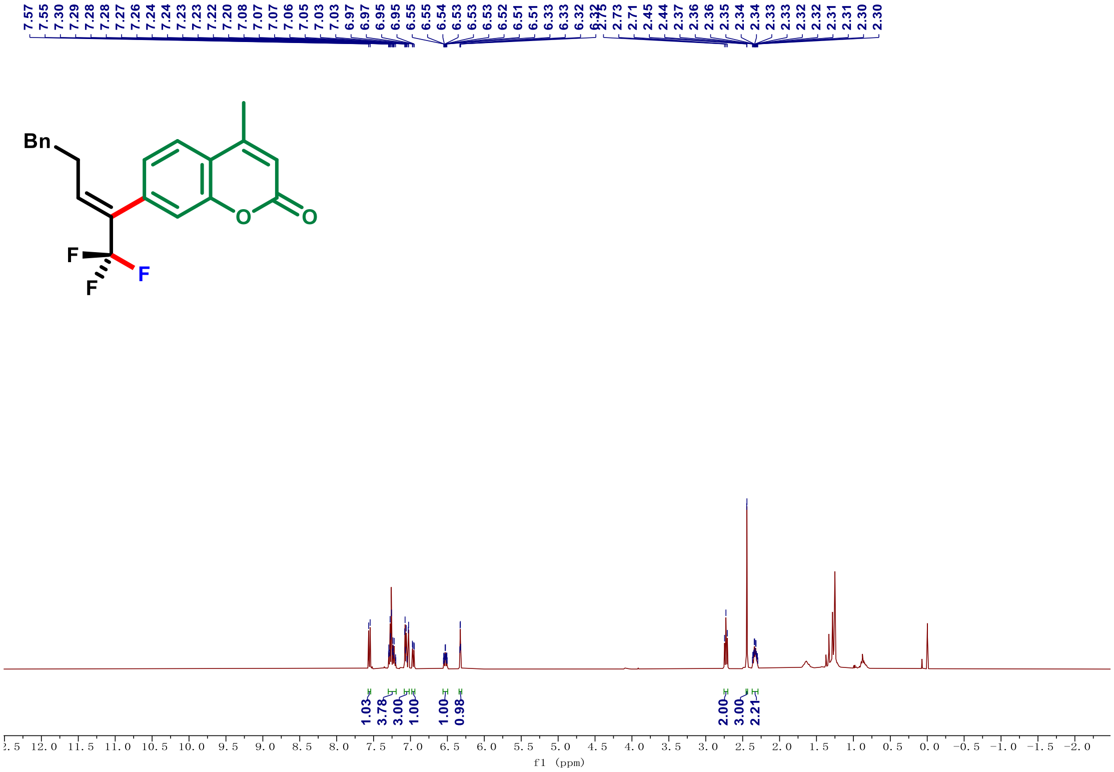

^{19}F NMR Spectrum of **3aq** ($CDCl_3$, 376 MHz)

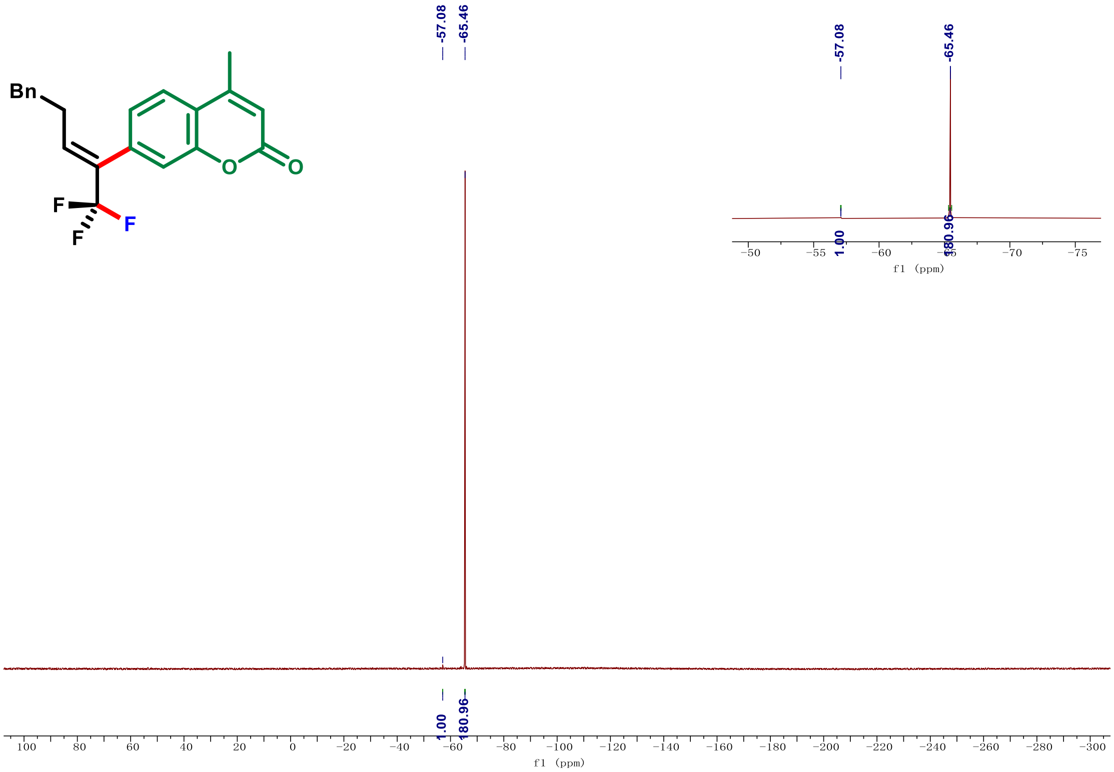

^{13}C NMR Spectrum of **3aq** ($CDCl_3$, 101 MHz)

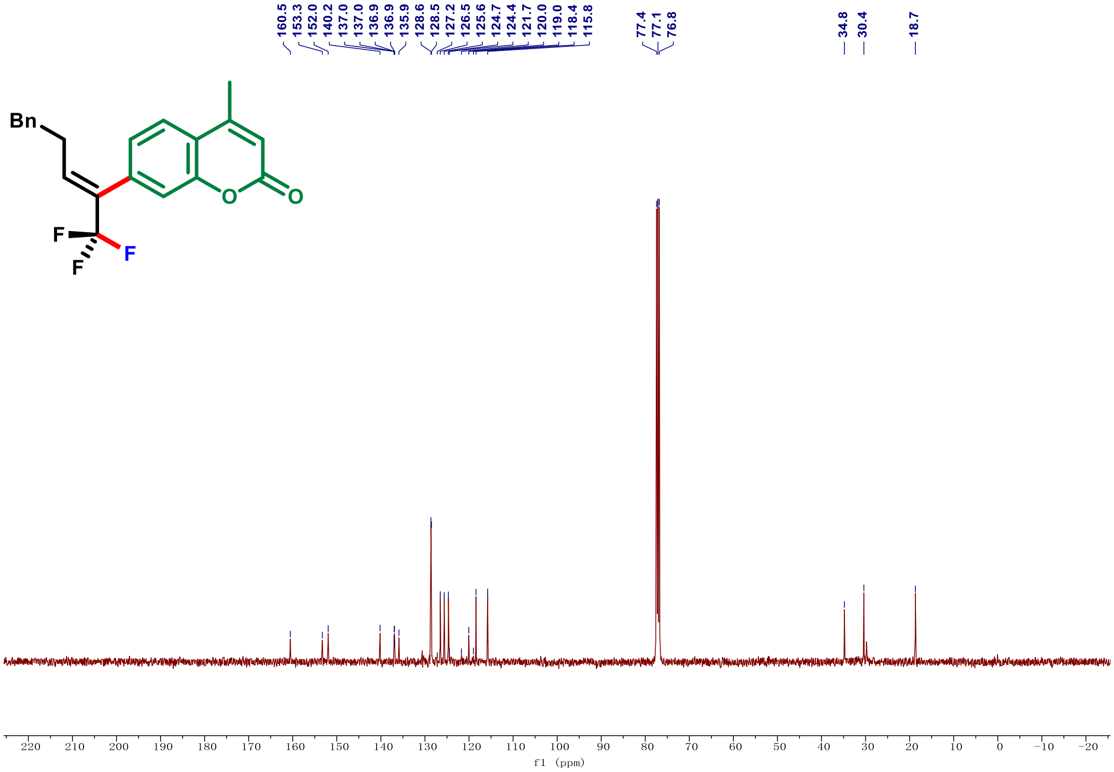

^{1}H NMR Spectrum of **3sa** ($CDCl_3$, 400 MHz)

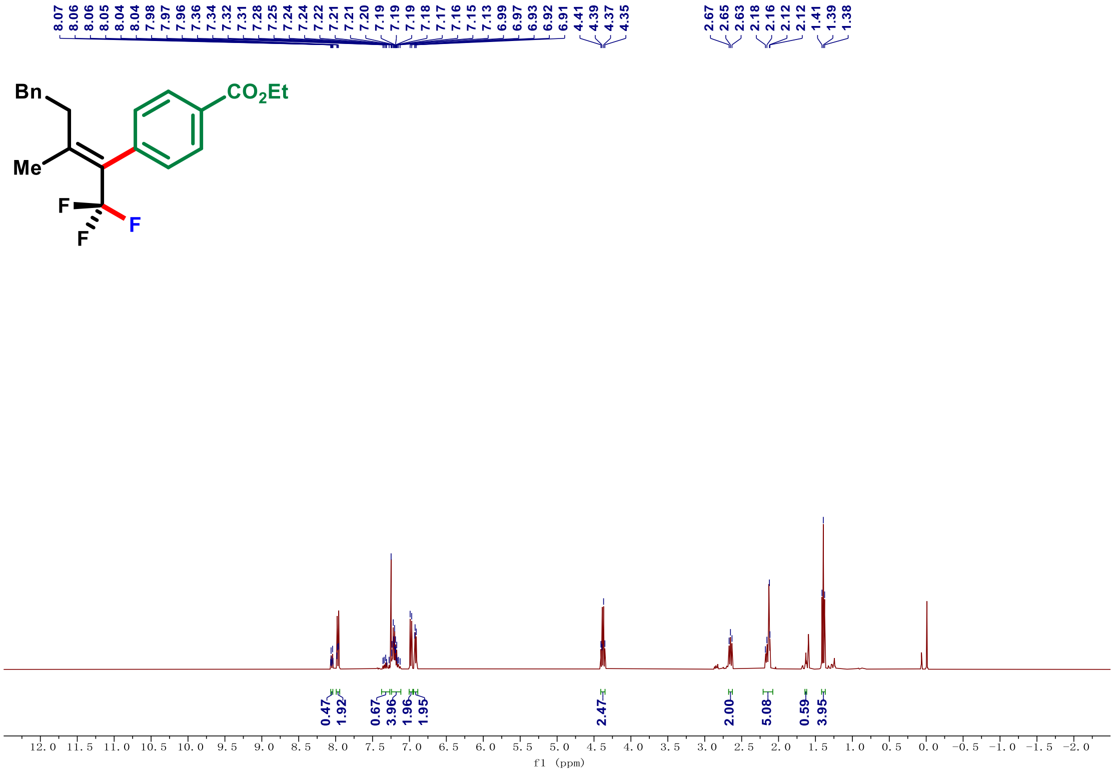

^{19}F NMR Spectrum of **3sa** ($CDCl_3$, 376 MHz)

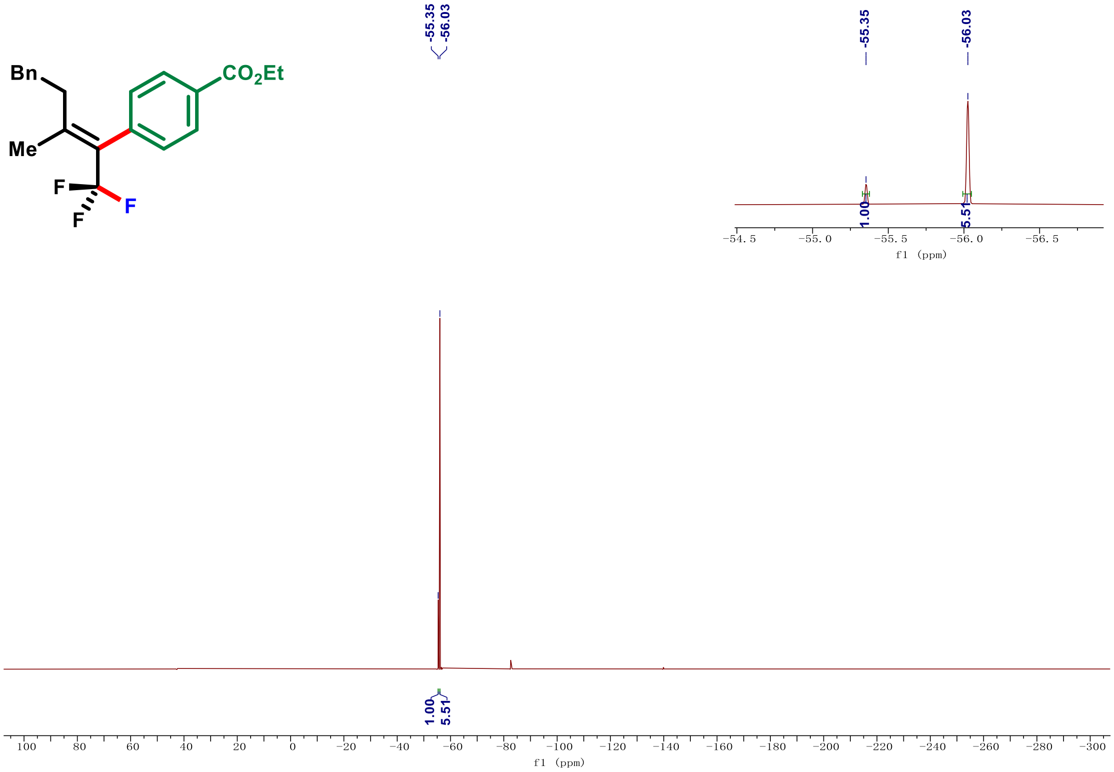

^{13}C NMR Spectrum of **3sa** ($CDCl_3$, 101 MHz)

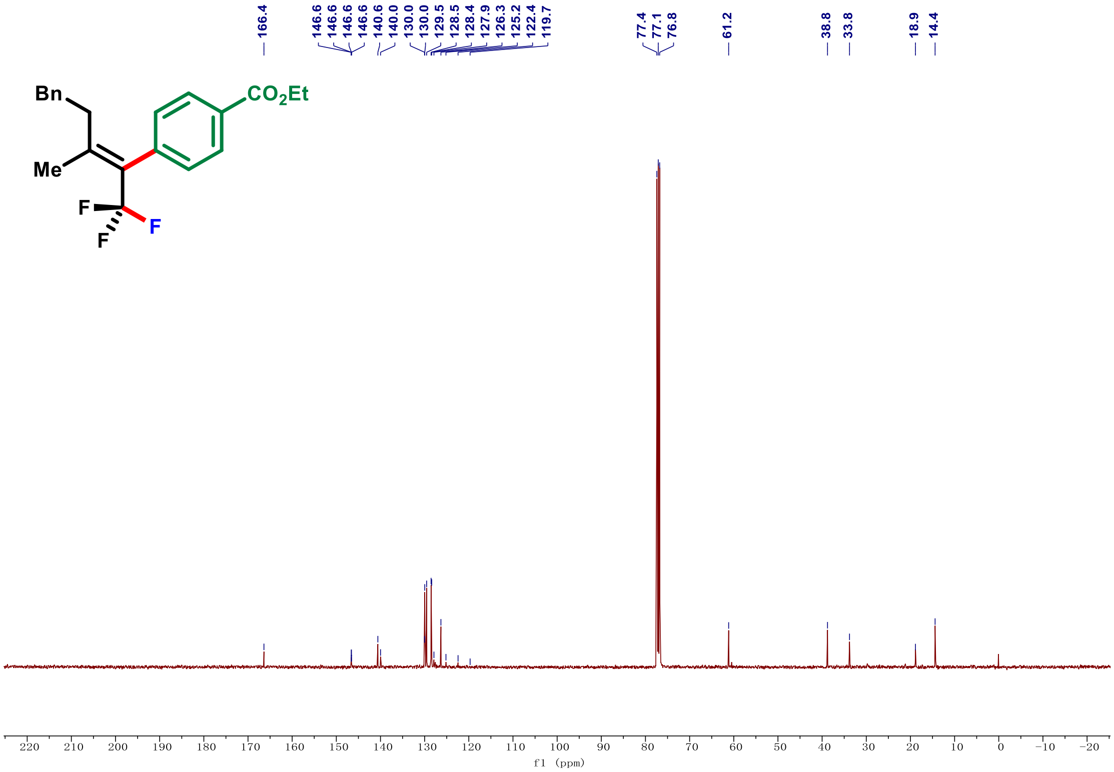

^{1}H NMR Spectrum of **3ta** ($CDCl_3$, 400 MHz)

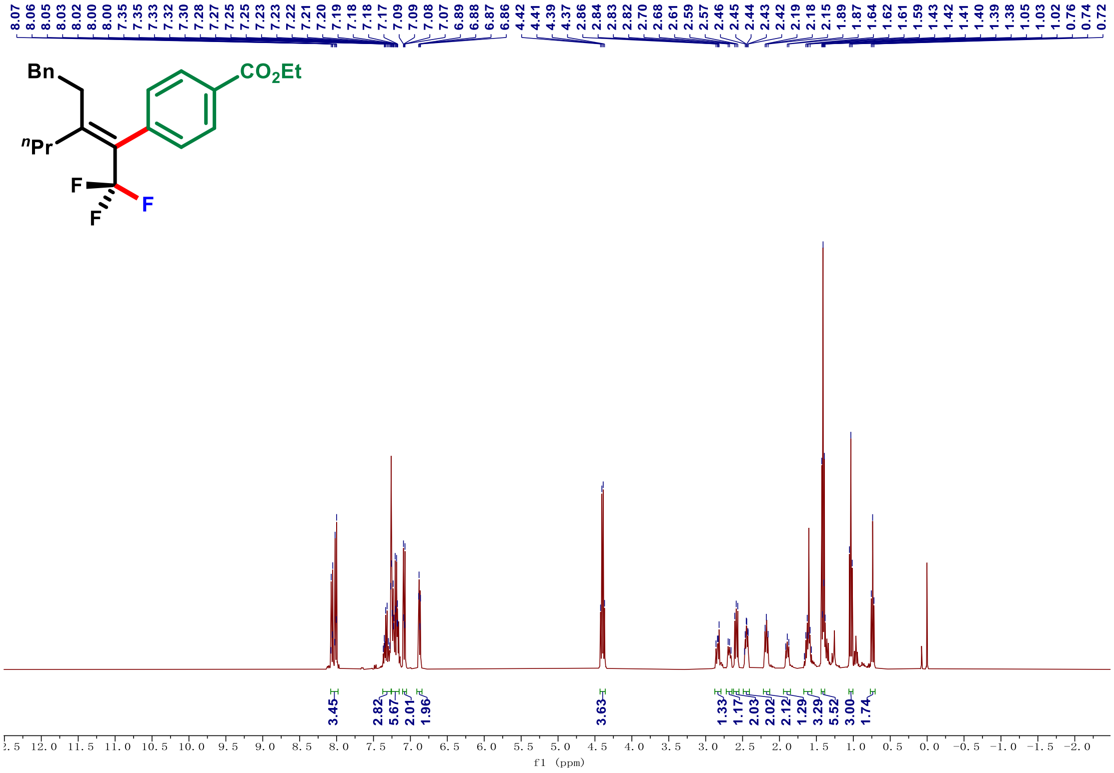

^{19}F NMR Spectrum of **3ta** ($CDCl_3$, 376 MHz)

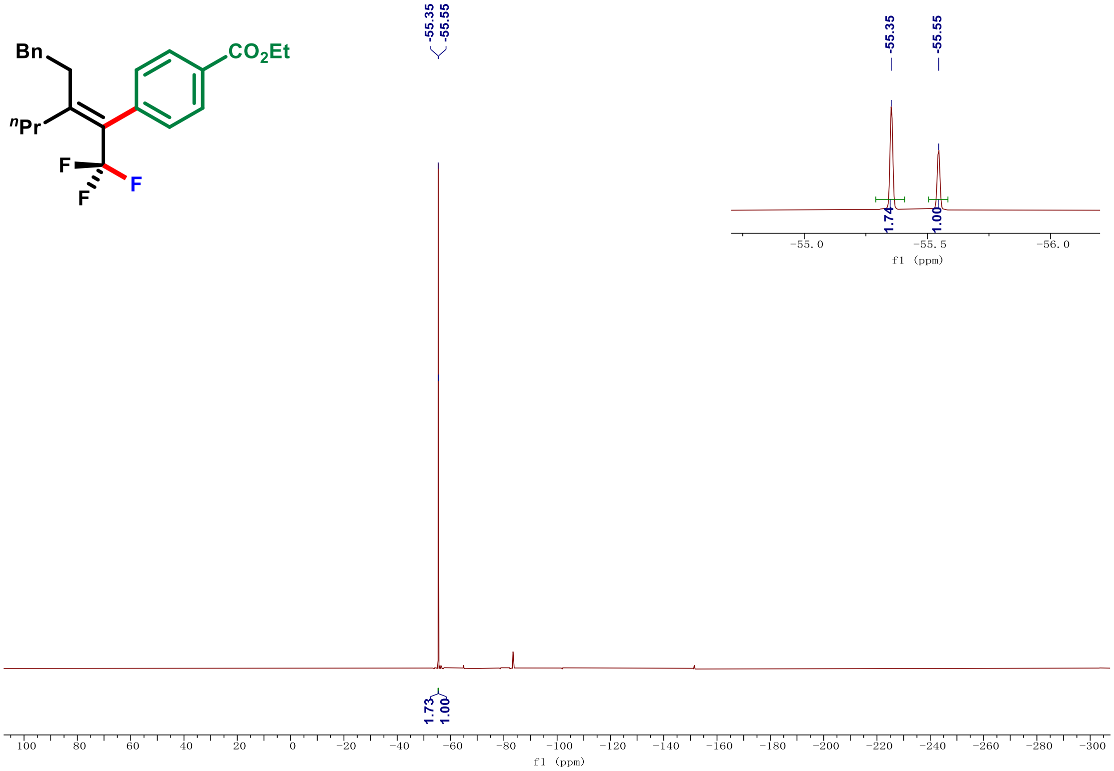

^{13}C NMR Spectrum of **3ta** ($CDCl_3$, 101 MHz)

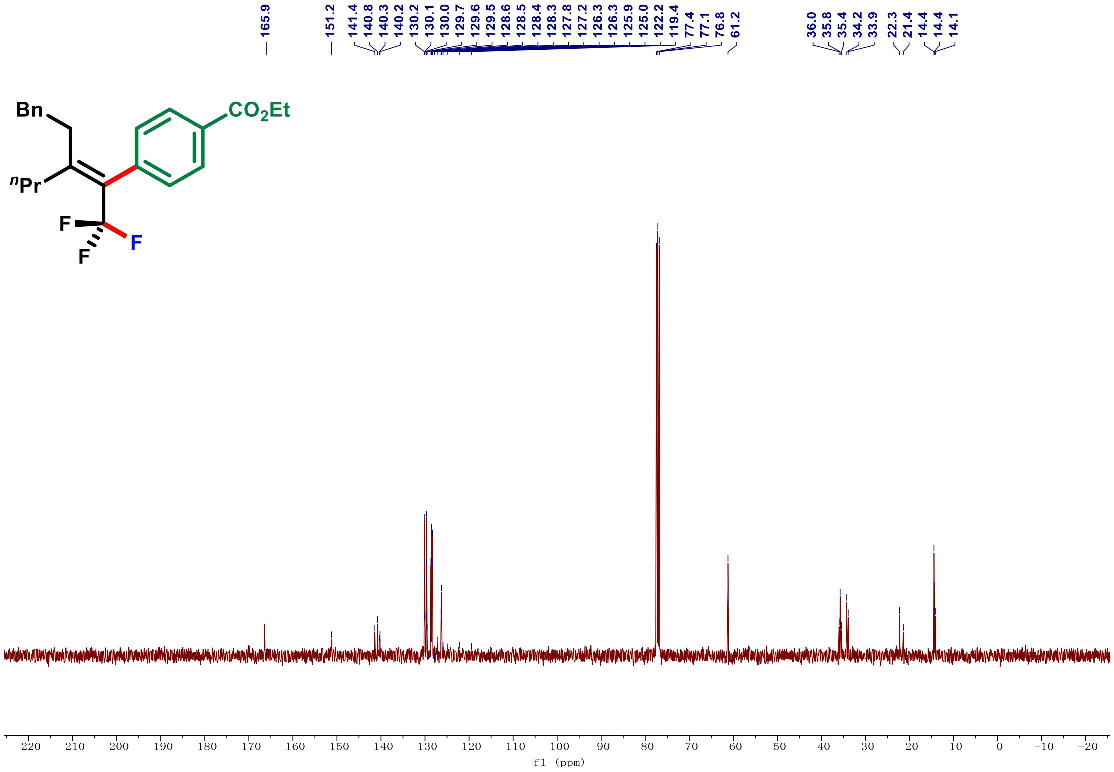

^{1}H NMR Spectrum of **3ua** ($CDCl_3$, 400 MHz)

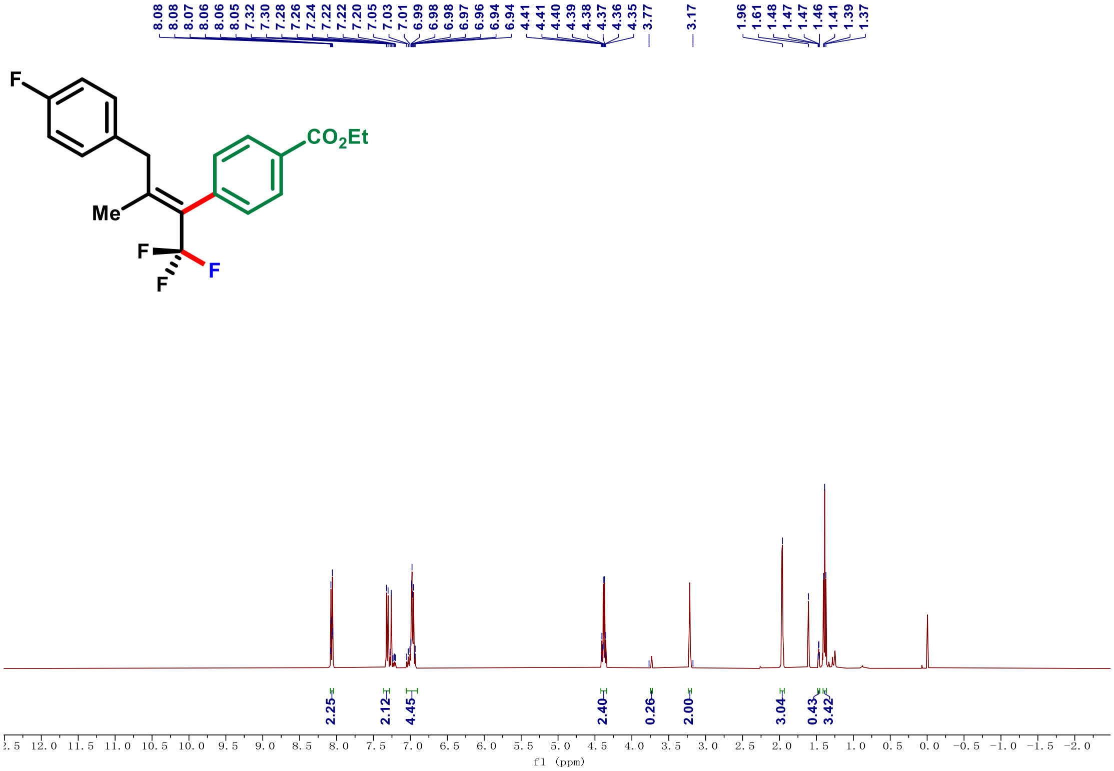

^{19}F NMR Spectrum of **3ua** ($CDCl_3$, 376 MHz)

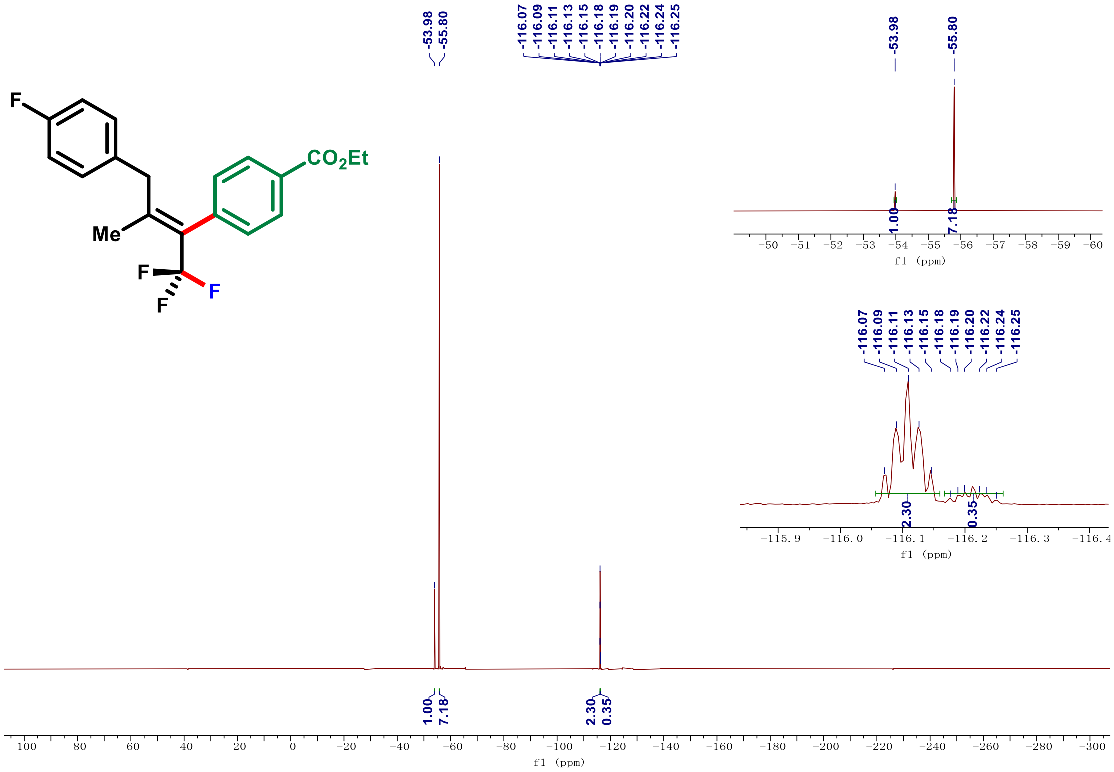

^{13}C NMR Spectrum of **3ua** ($CDCl_3$, 101 MHz)

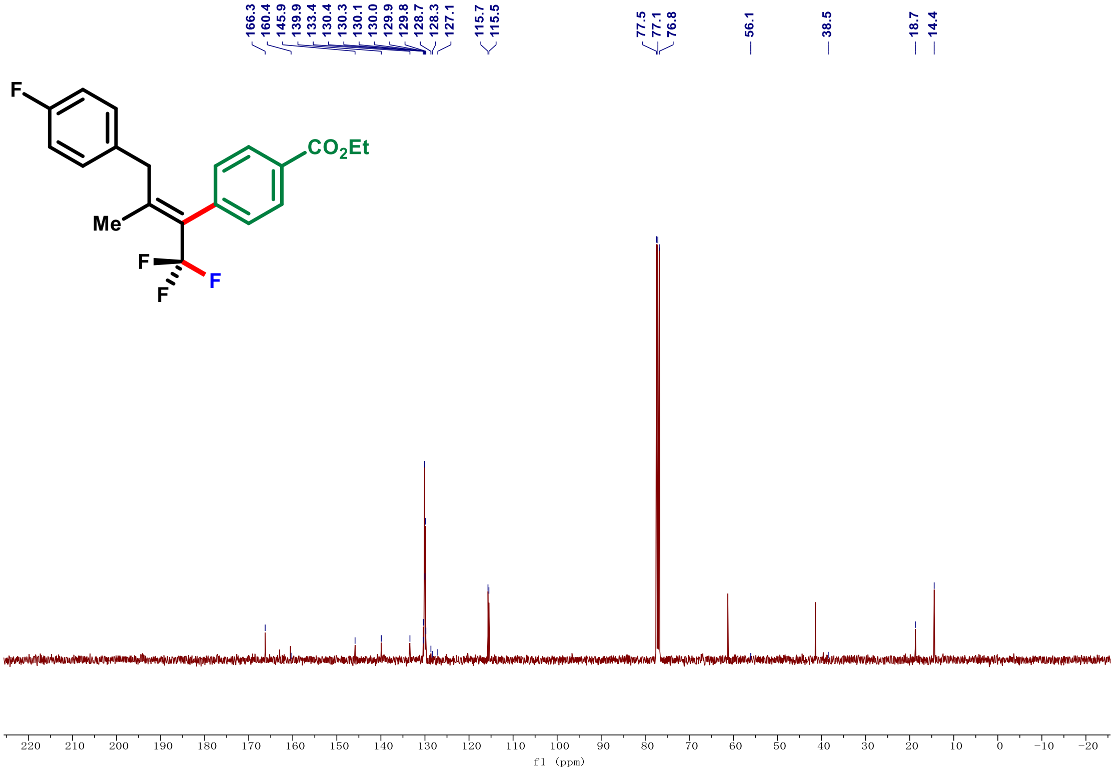

^{1}H NMR Spectrum of **3va** ($CDCl_3$, 400 MHz)

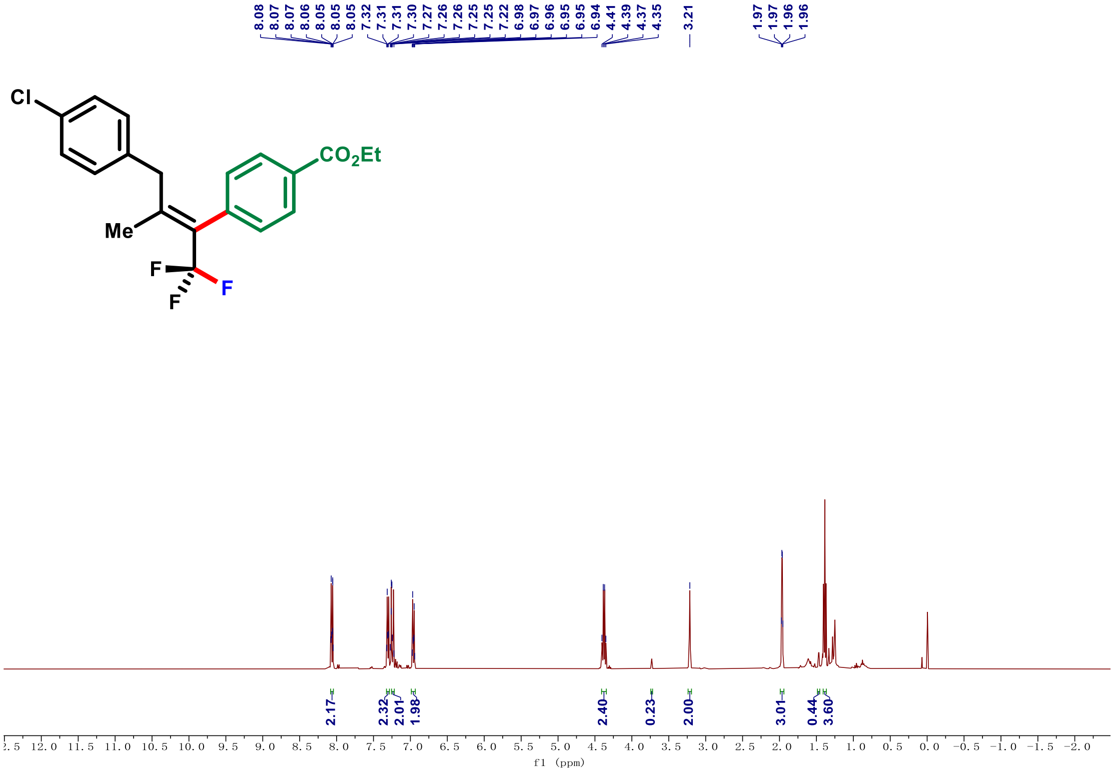

^{19}F NMR Spectrum of **3va** ($CDCl_3$, 376 MHz)

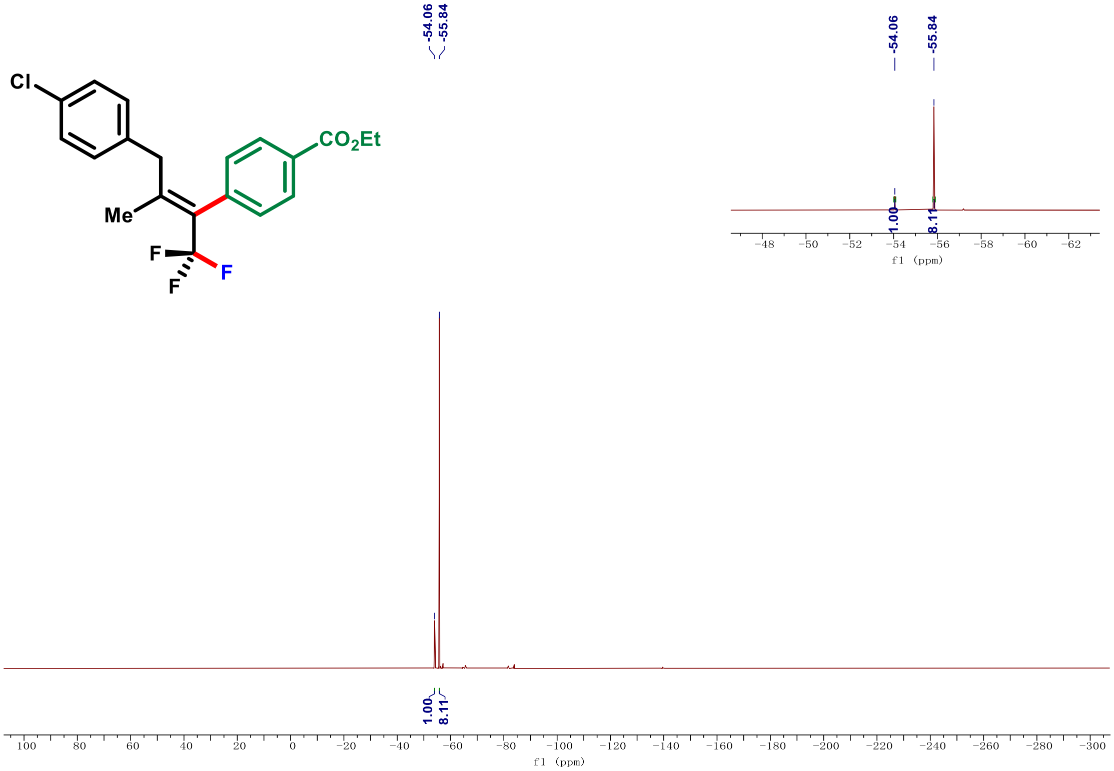

^{13}C NMR Spectrum of **3va** ($CDCl_3$, 101 MHz)

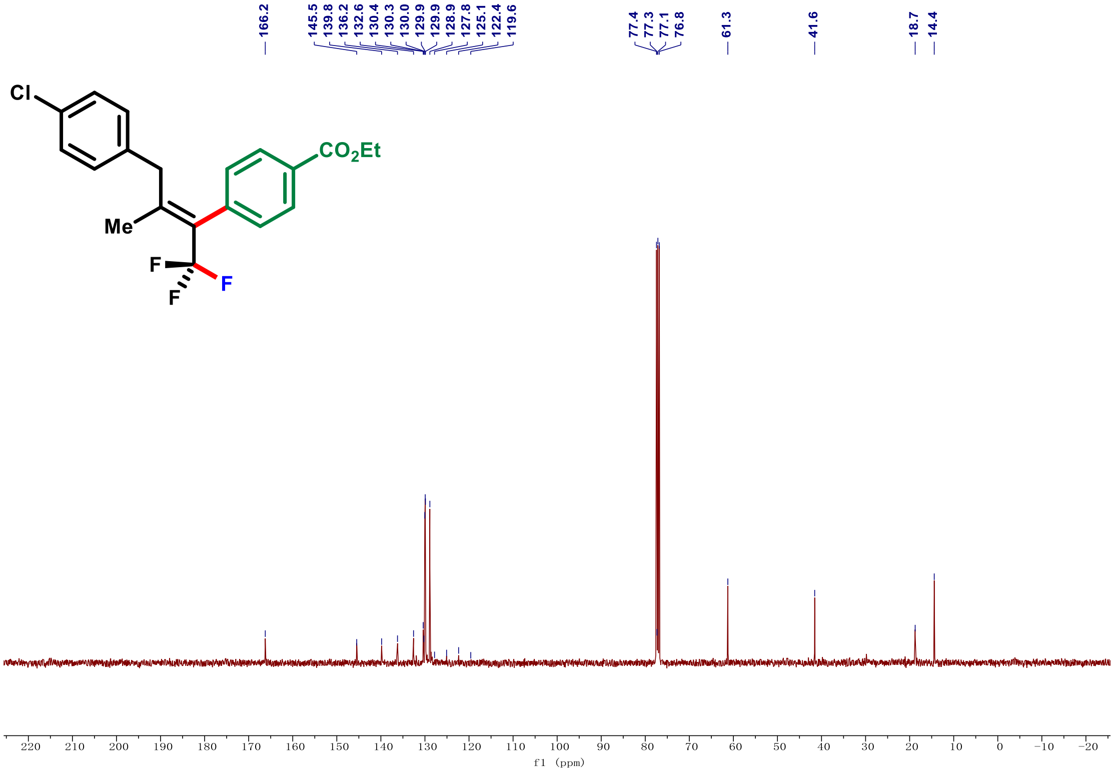

^{1}H NMR Spectrum of **3va-Cosy** ($CDCl_3$, 400 MHz)

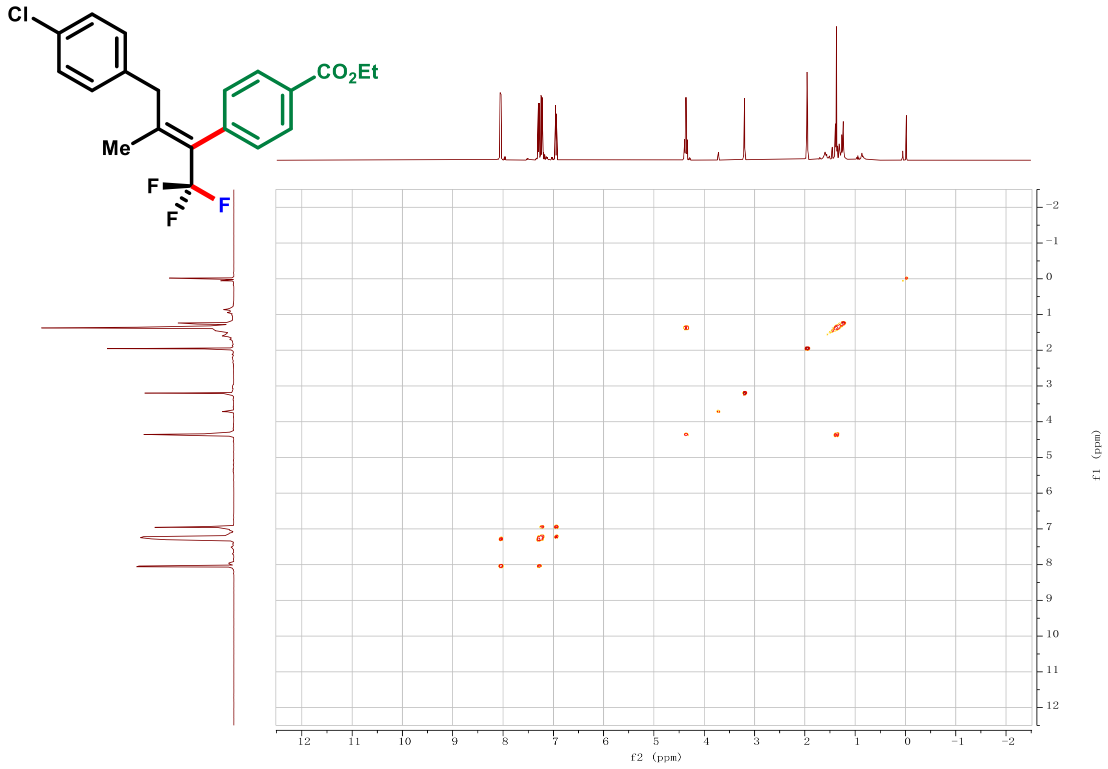

^{1}H NMR Spectrum of **3va-Cosy** ($CDCl_3$, 400 MHz)

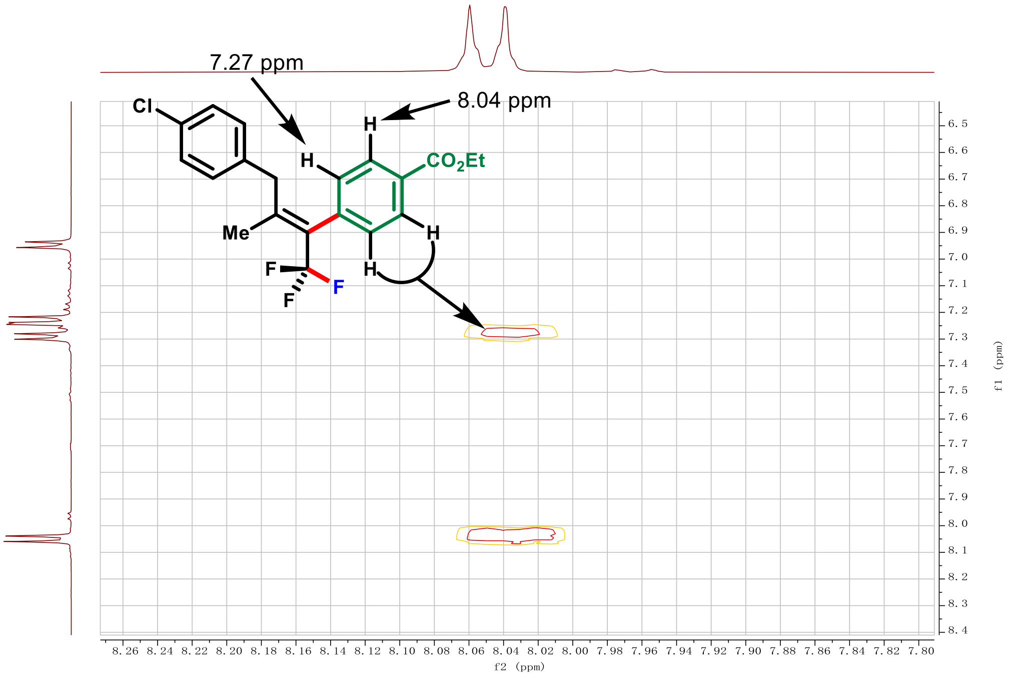

^{1}H NMR Spectrum of **3va-2D-Noesy** ($CDCl_3$, 400 MHz)

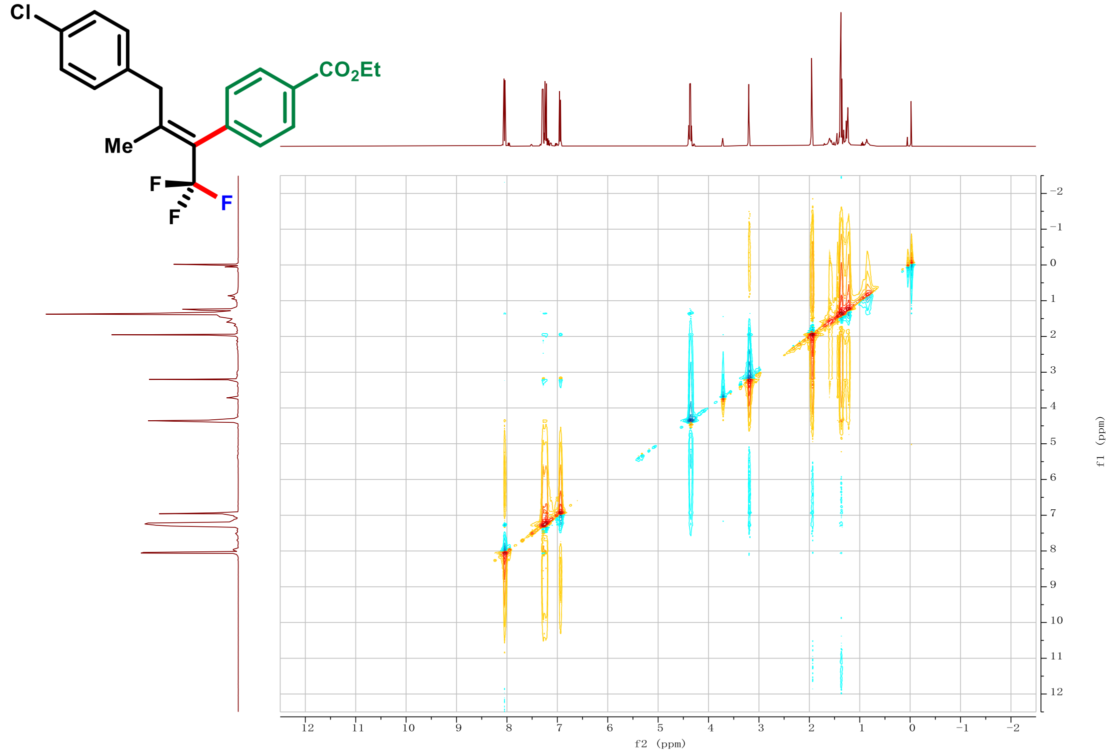

^{1}H NMR Spectrum of **3va-2D-Noesy** ($CDCl_3$, 400 MHz)

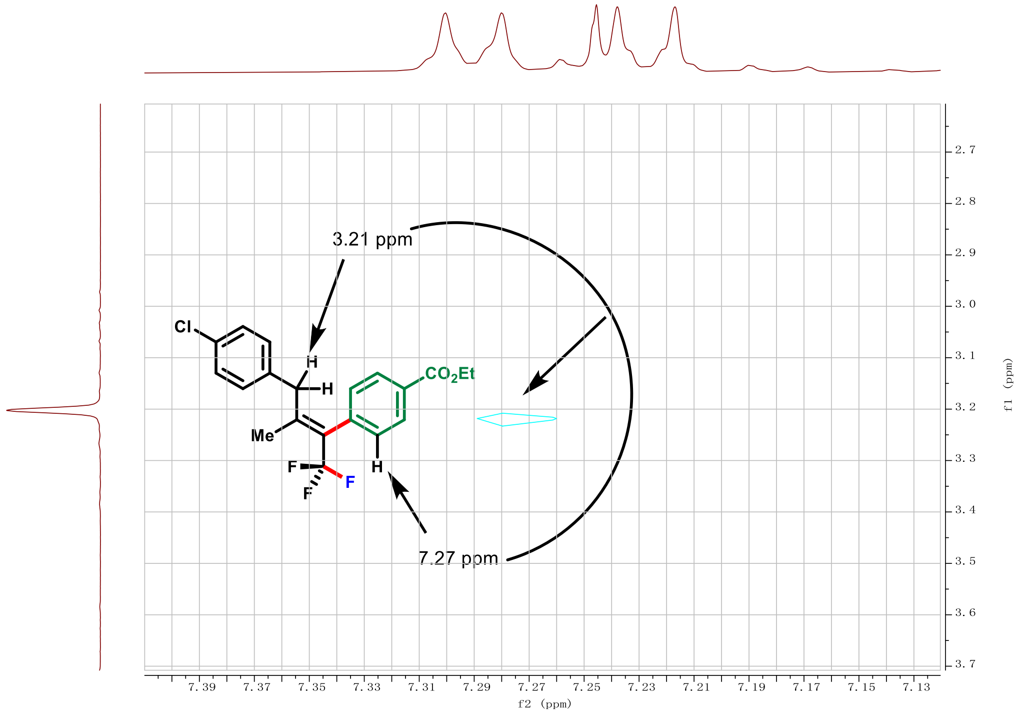

^{1}H NMR Spectrum of **3wa** ($CDCl_3$, 400 MHz)

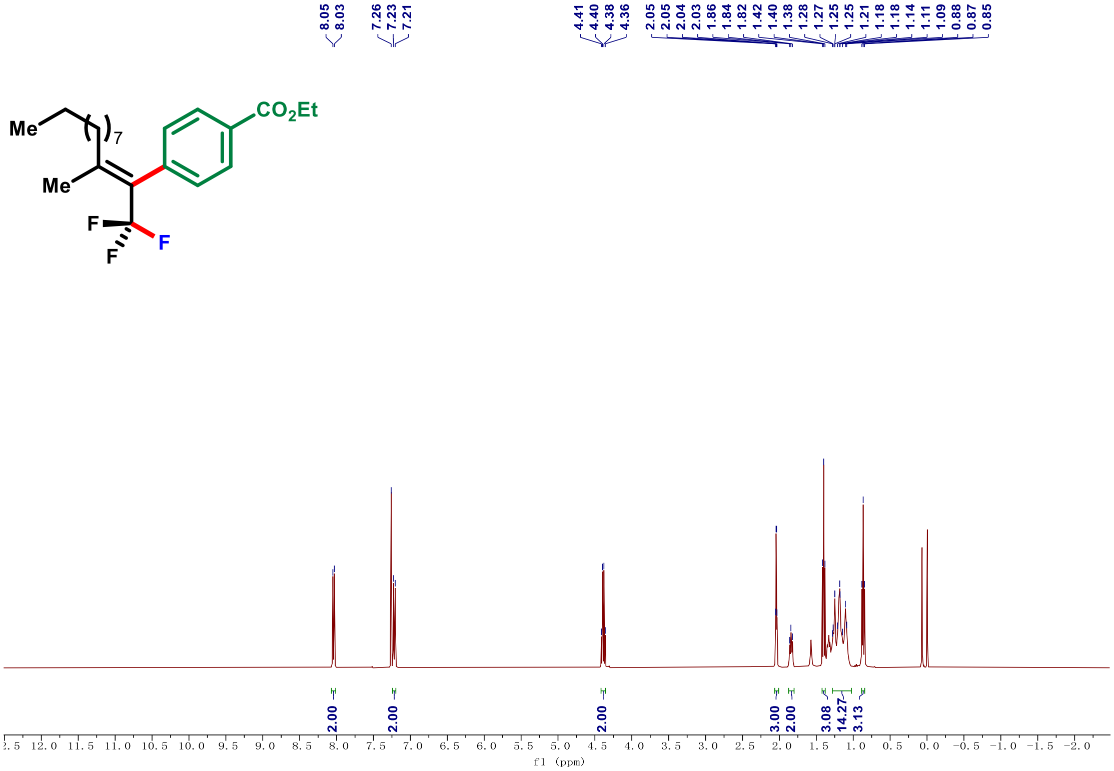

^{19}F NMR Spectrum of **3wa** ($CDCl_3$, 376 MHz)

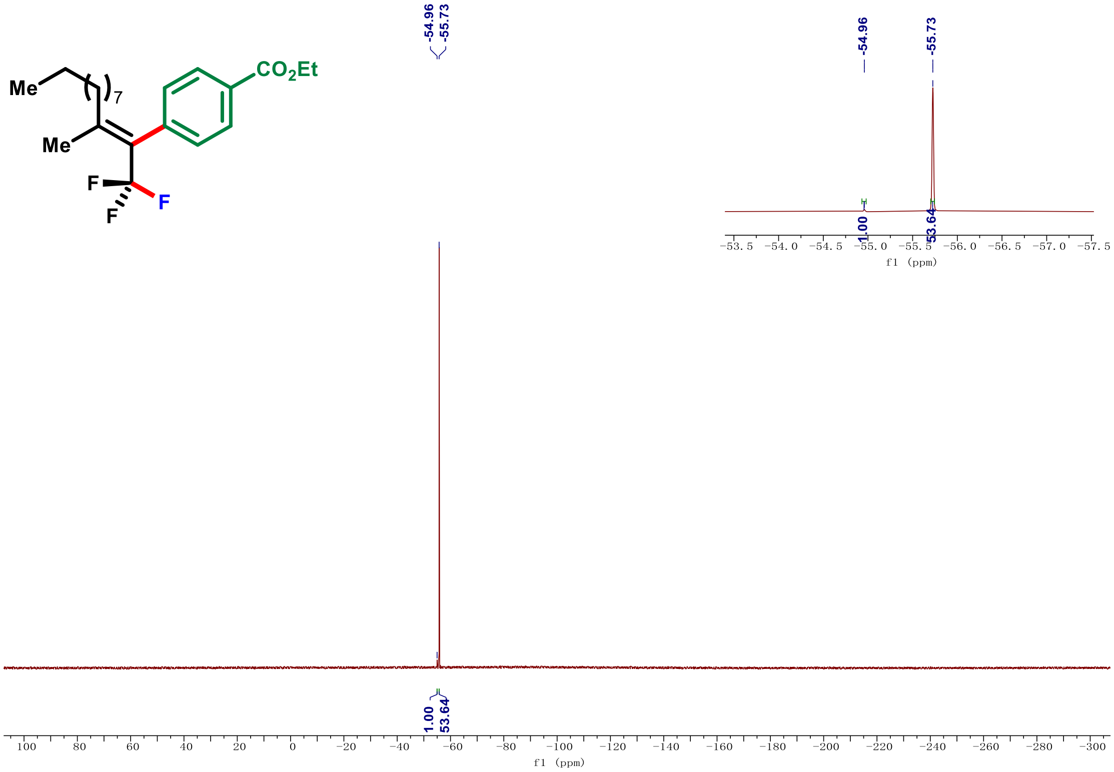

^{13}C NMR Spectrum of **3wa** ($CDCl_3$, 101 MHz)

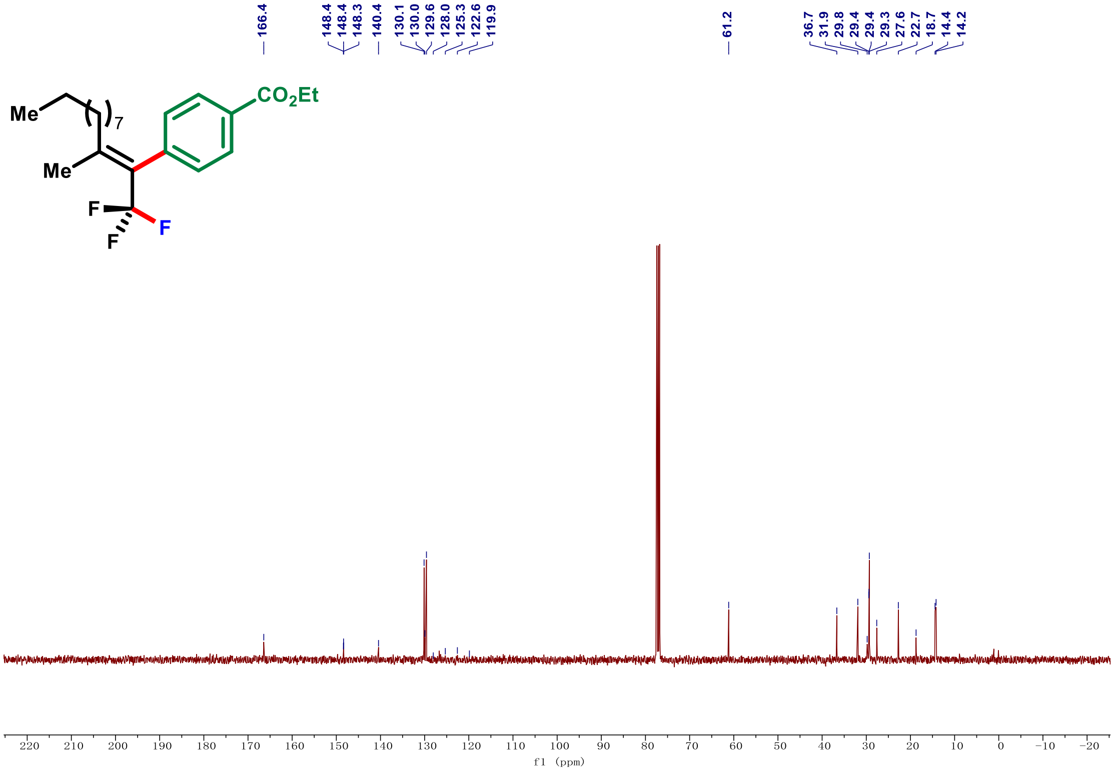

^{1}H NMR Spectrum of **3xa** ($CDCl_3$, 400 MHz)

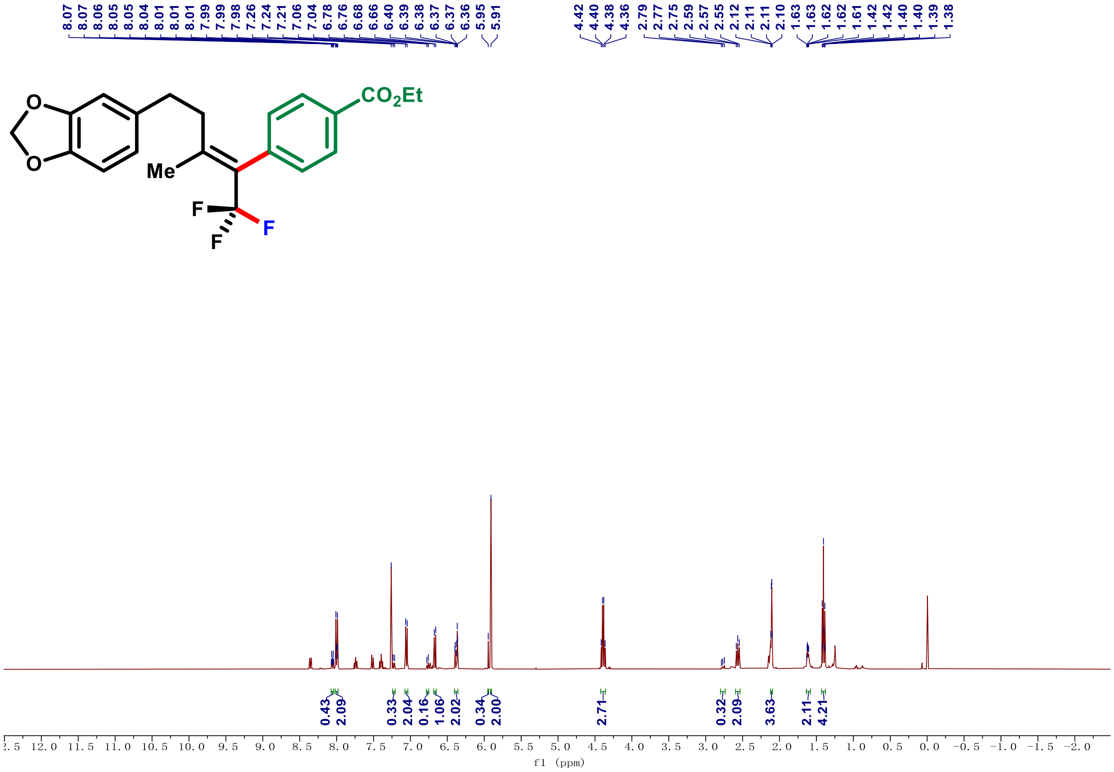

^{19}F NMR Spectrum of **3xa** ($CDCl_3$, 376 MHz)

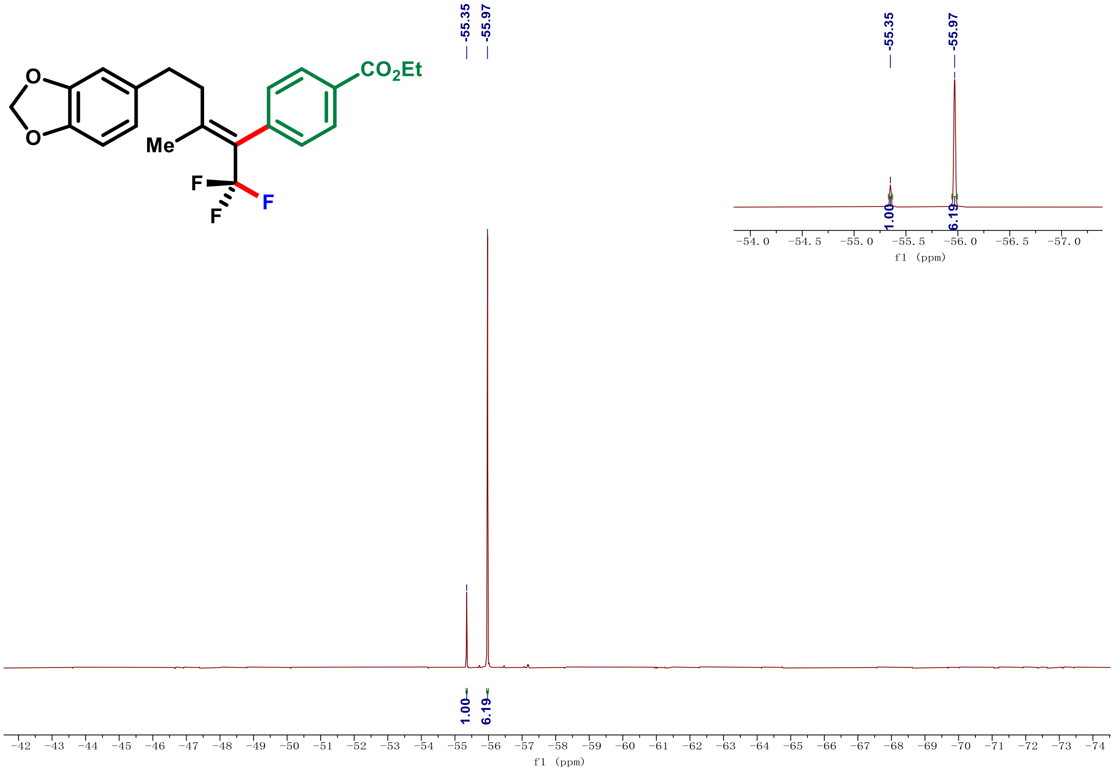

^{13}C NMR Spectrum of **3xa** ($CDCl_3$, 101 MHz)

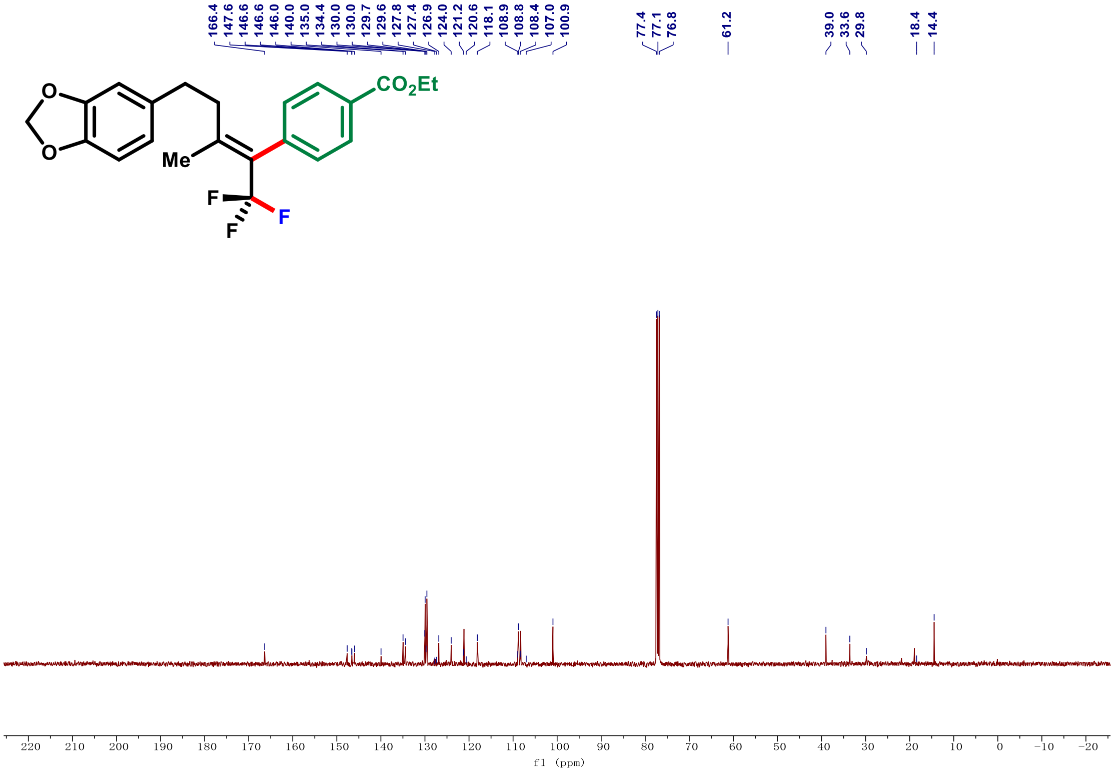

^{1}H NMR Spectrum of **3ya** ($CDCl_3$, 400 MHz)

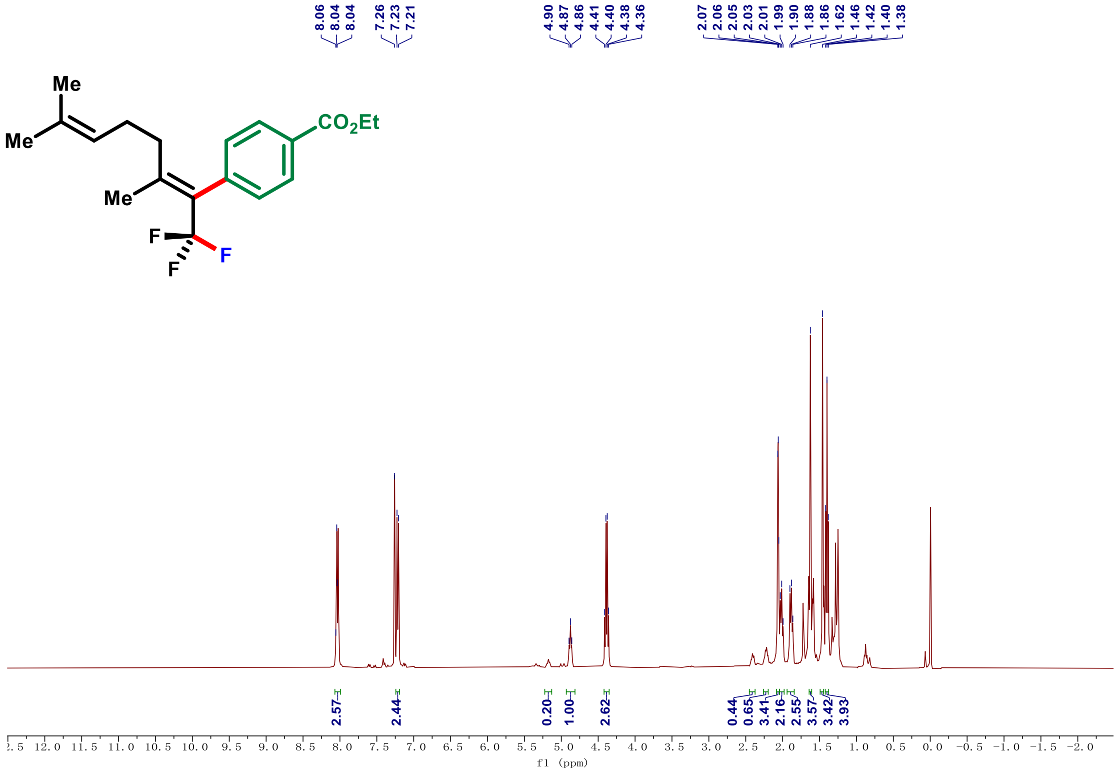

^{19}F NMR Spectrum of **3ya** ($CDCl_3$, 376 MHz)

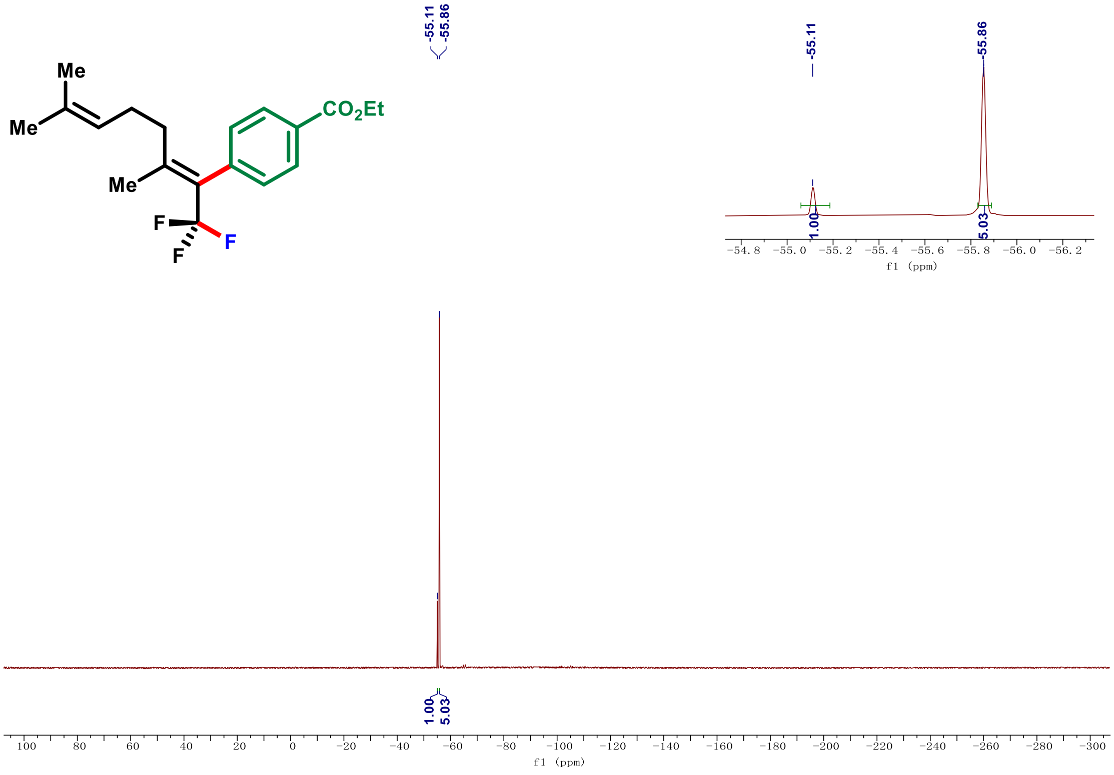

^{13}C NMR Spectrum of **3ya** ($CDCl_3$, 101 MHz)

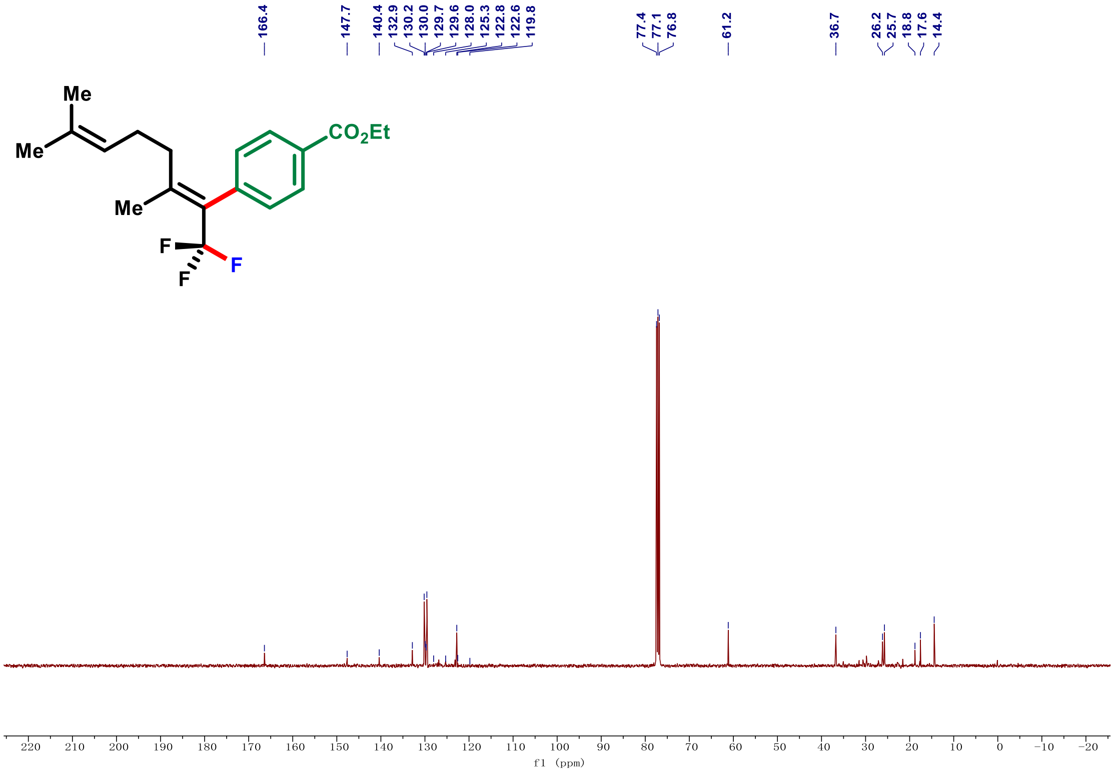

^{1}H NMR Spectrum of **3za** ($CDCl_3$, 400 MHz)

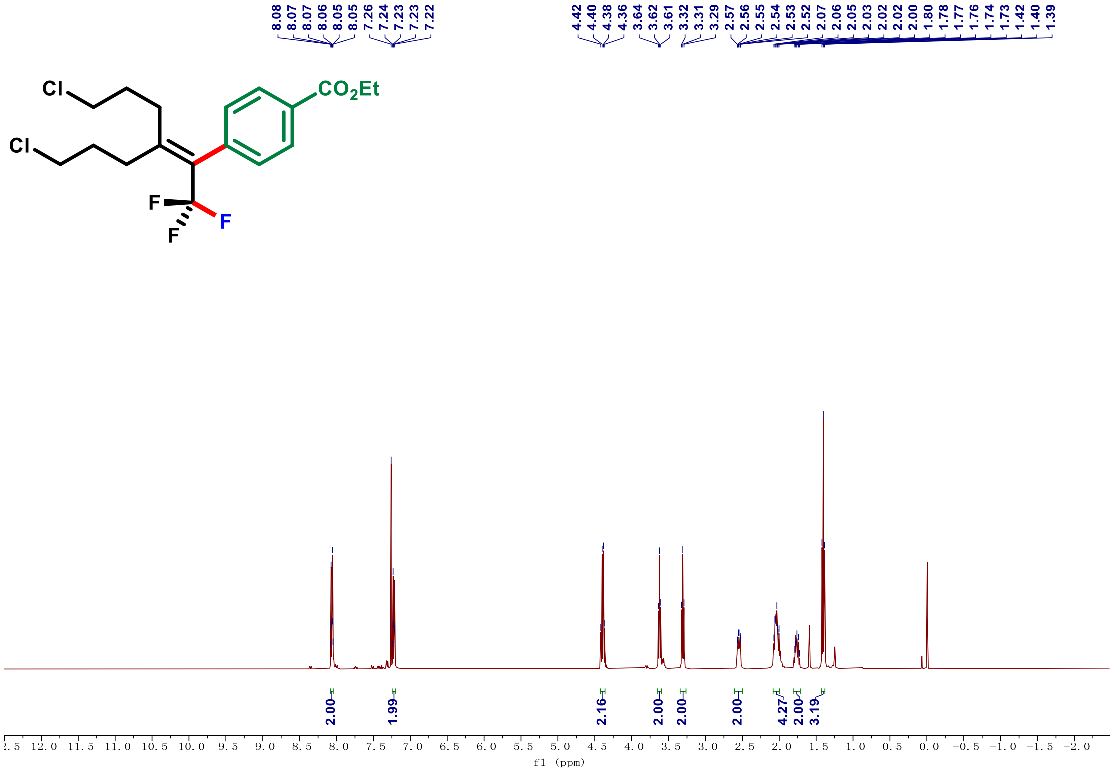

^{19}F NMR Spectrum of **3za** ($CDCl_3$, 376 MHz)

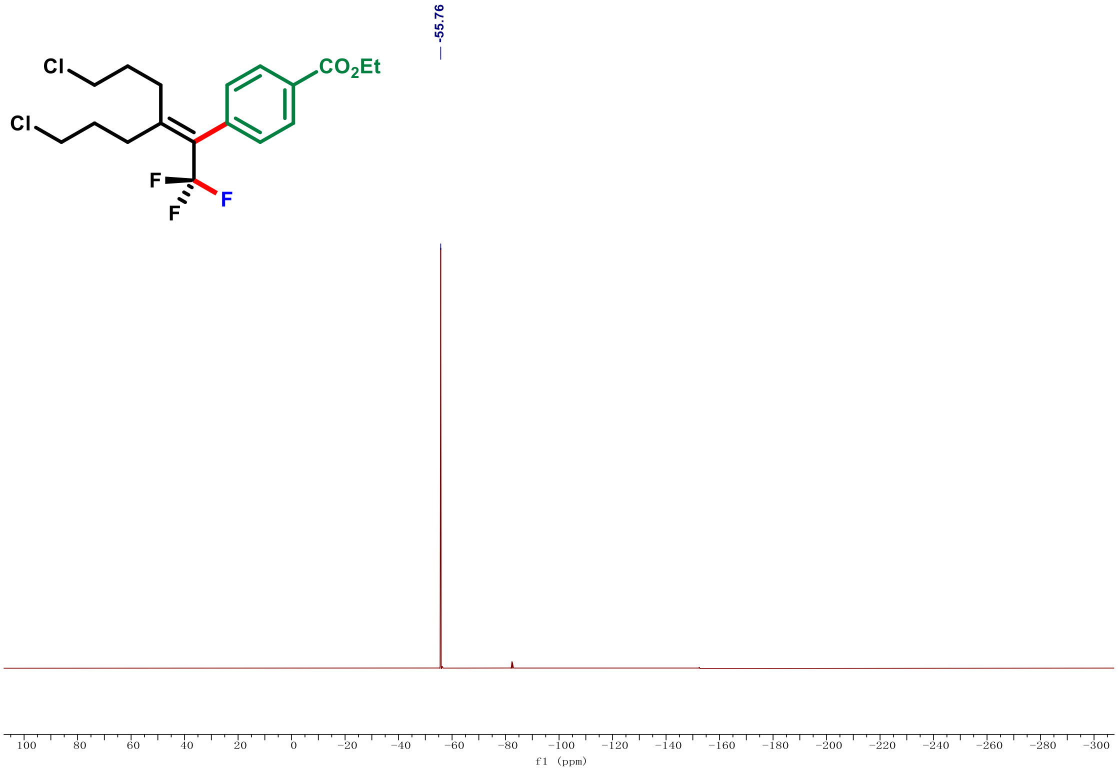

^{13}C NMR Spectrum of **3za** ($CDCl_3$, 101 MHz)

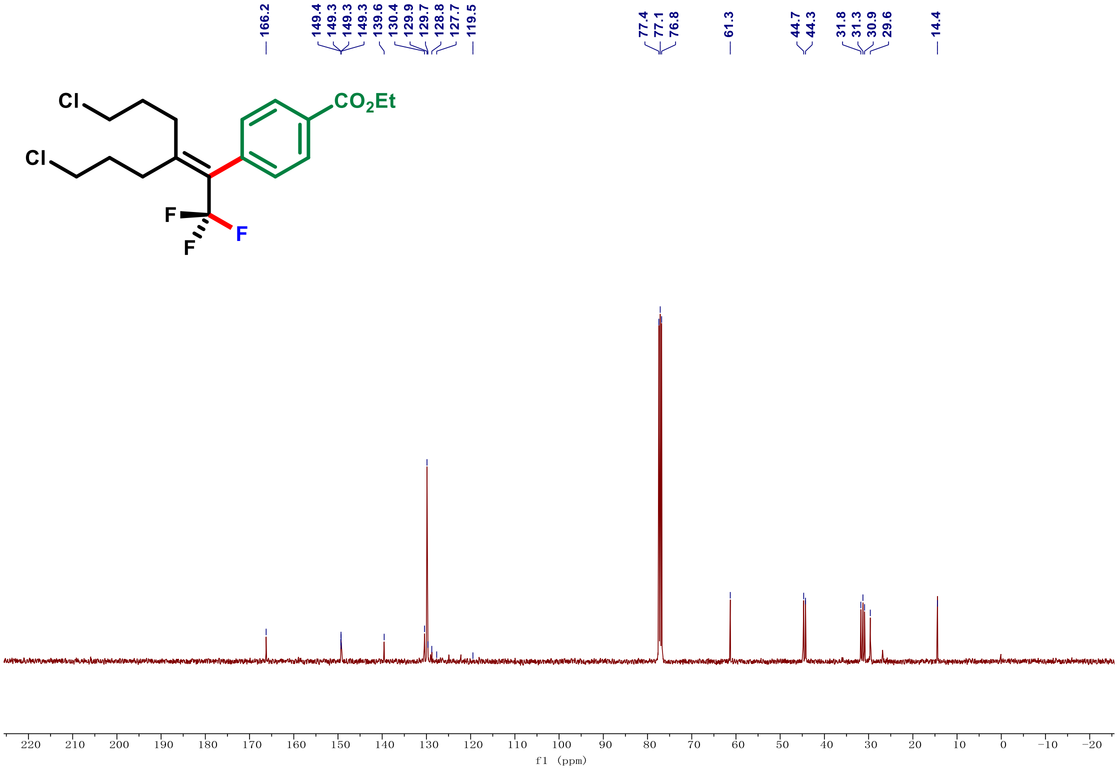

^{1}H NMR Spectrum of **3aaa** ($CDCl_3$, 400 MHz)

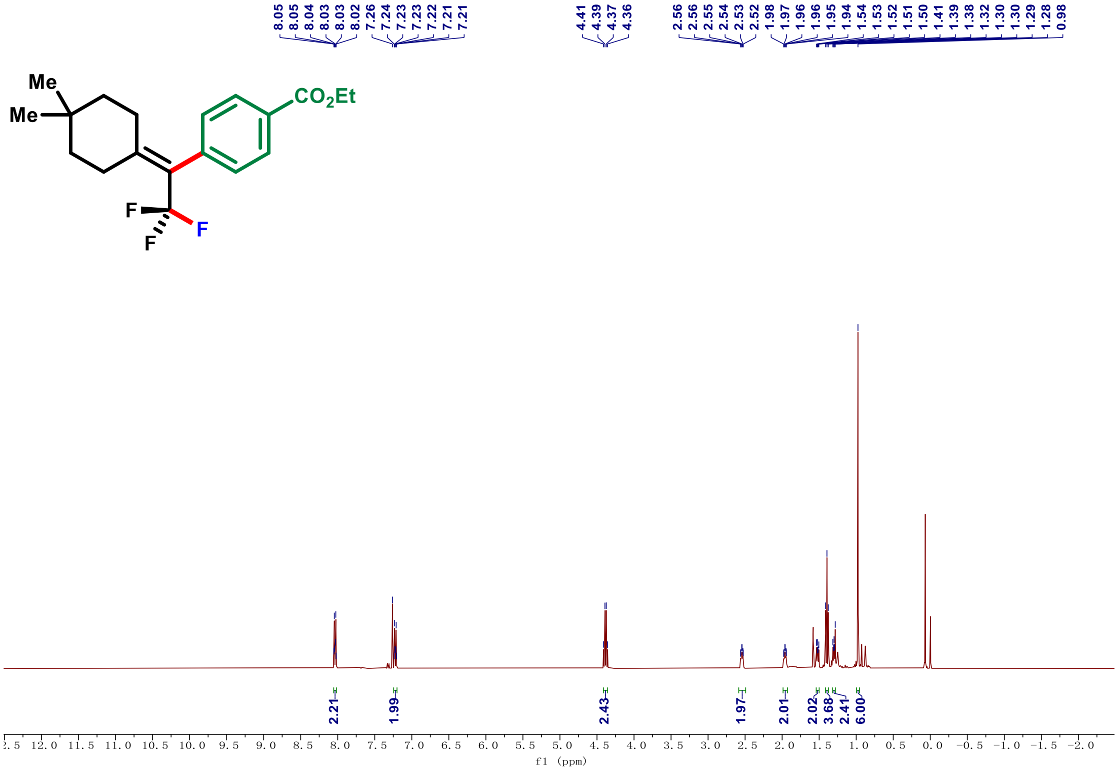

^{19}F NMR Spectrum of **3aaa** ($CDCl_3$, 376 MHz)

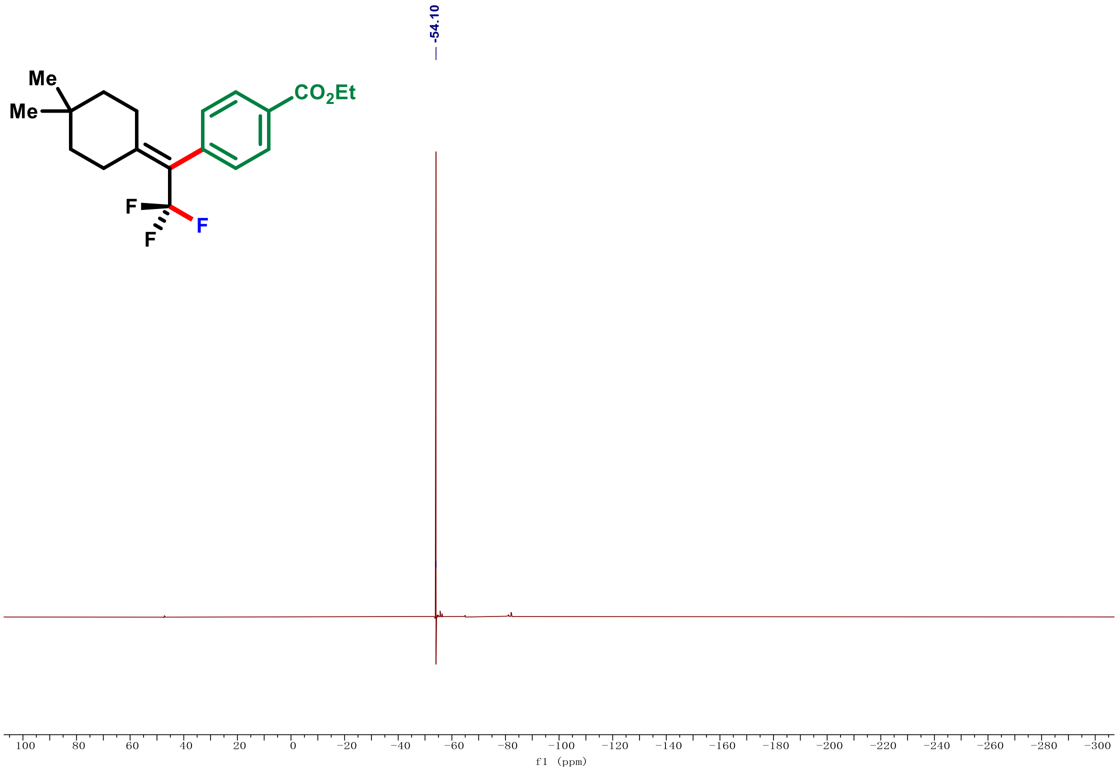

^{13}C NMR Spectrum of **3aaa** ($CDCl_3$, 101 MHz)

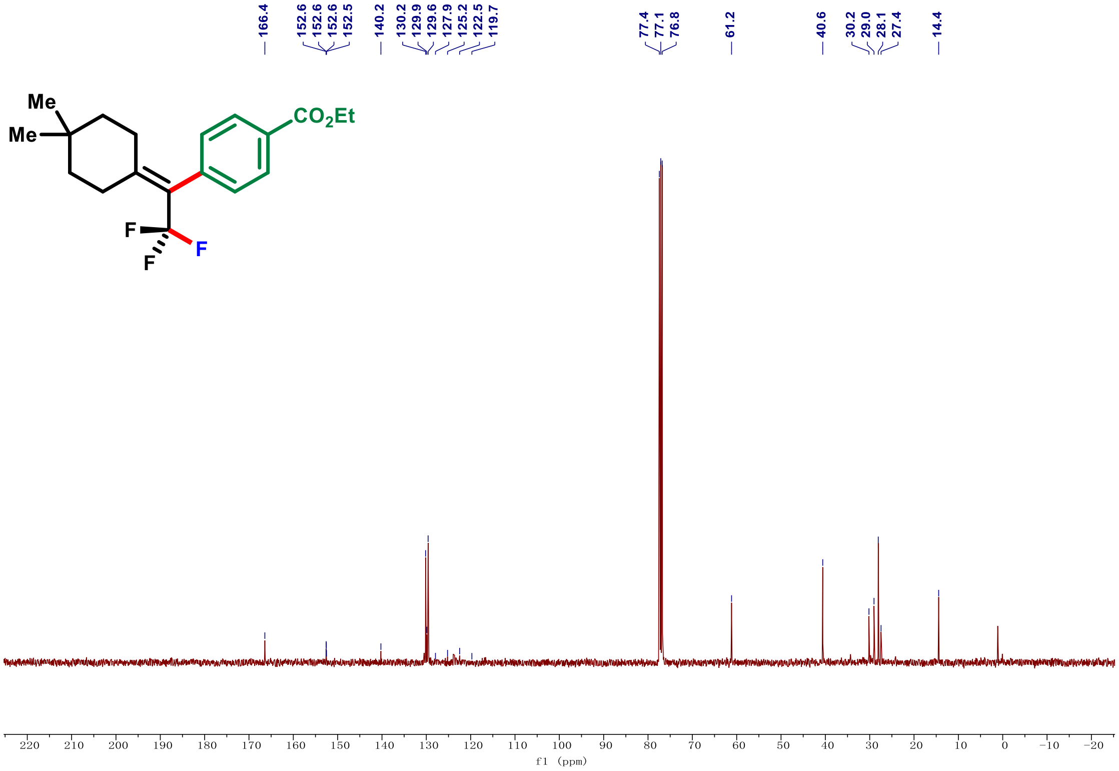

^{1}H NMR Spectrum of **3aba** ($CDCl_3$, 400 MHz)

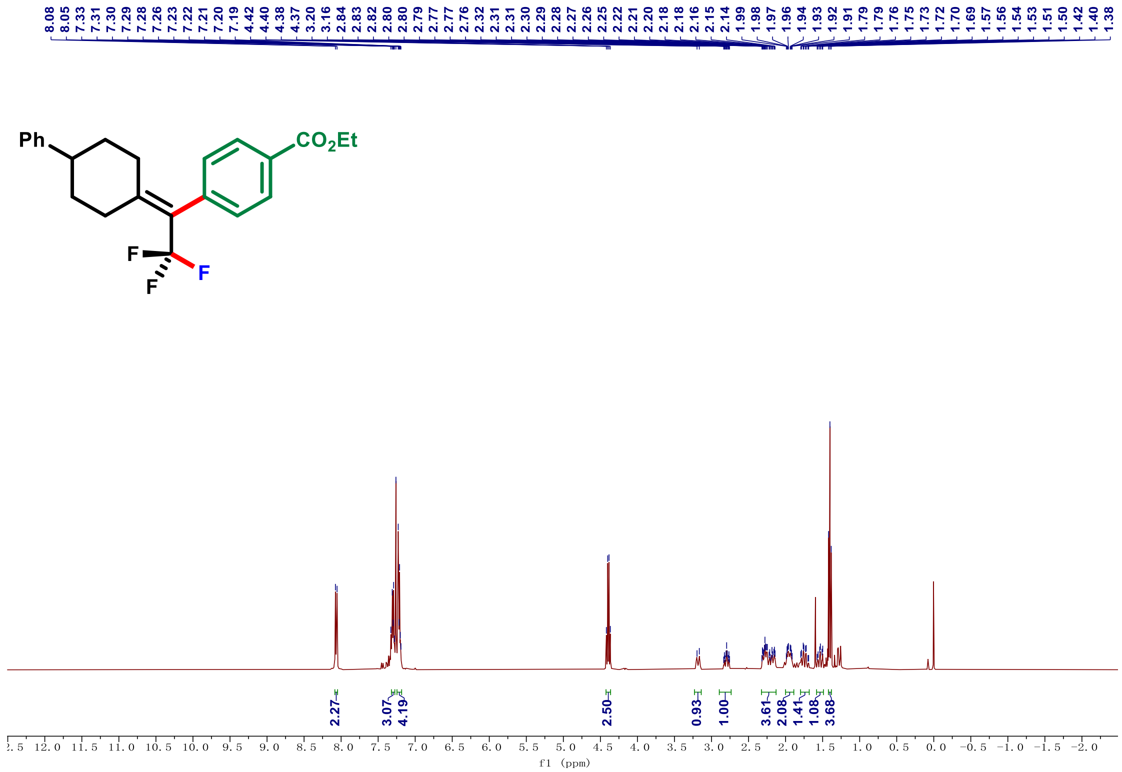

^{19}F NMR Spectrum of **3aba** ($CDCl_3$, 376 MHz)

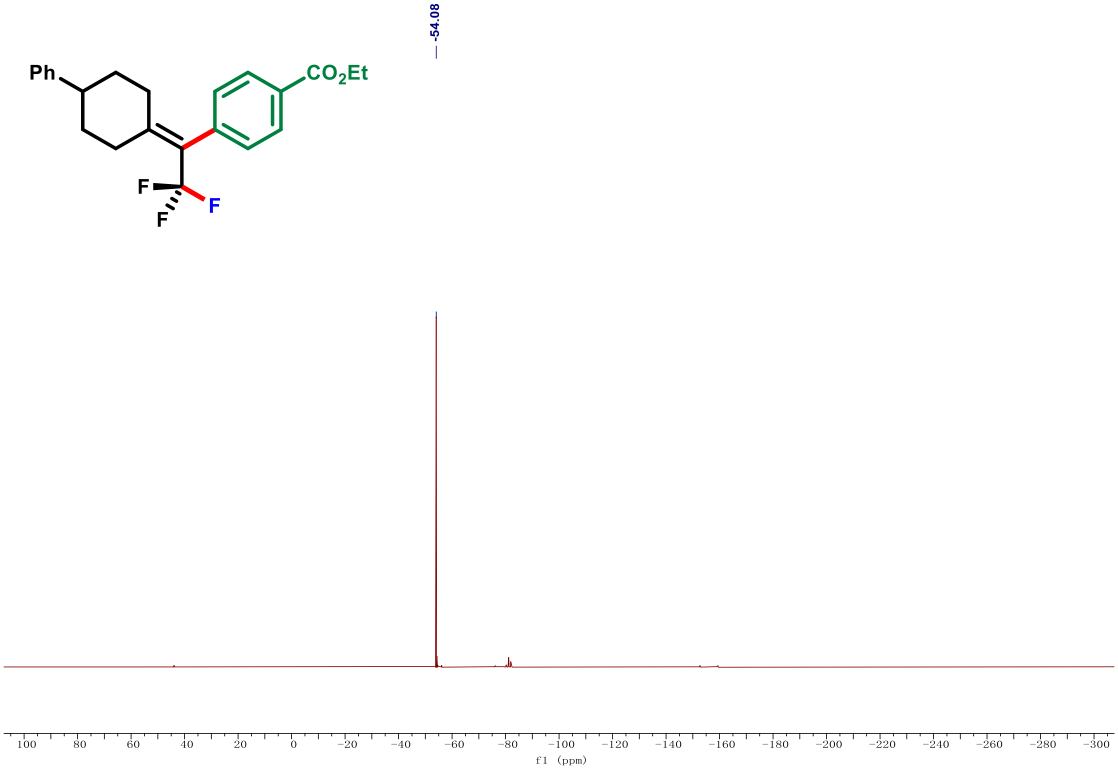

^{13}C NMR Spectrum of **3aba** ($CDCl_3$, 101 MHz)

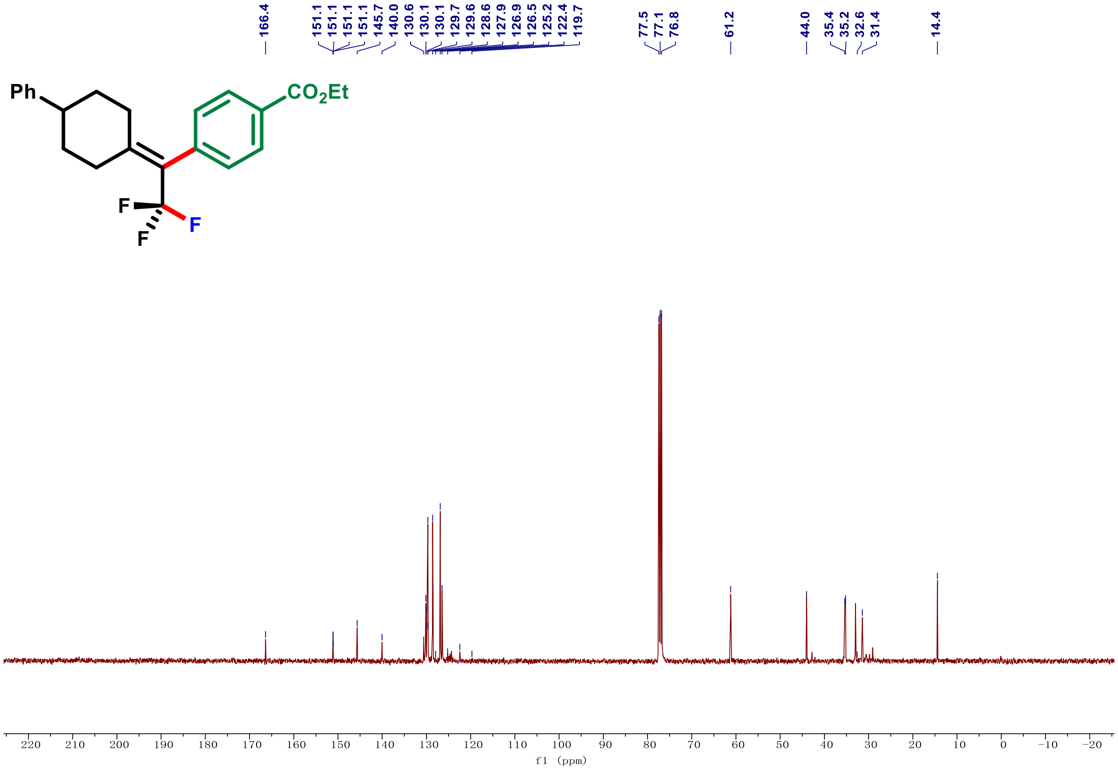

^{1}H NMR Spectrum of **3aca** ($CDCl_3$, 400 MHz)

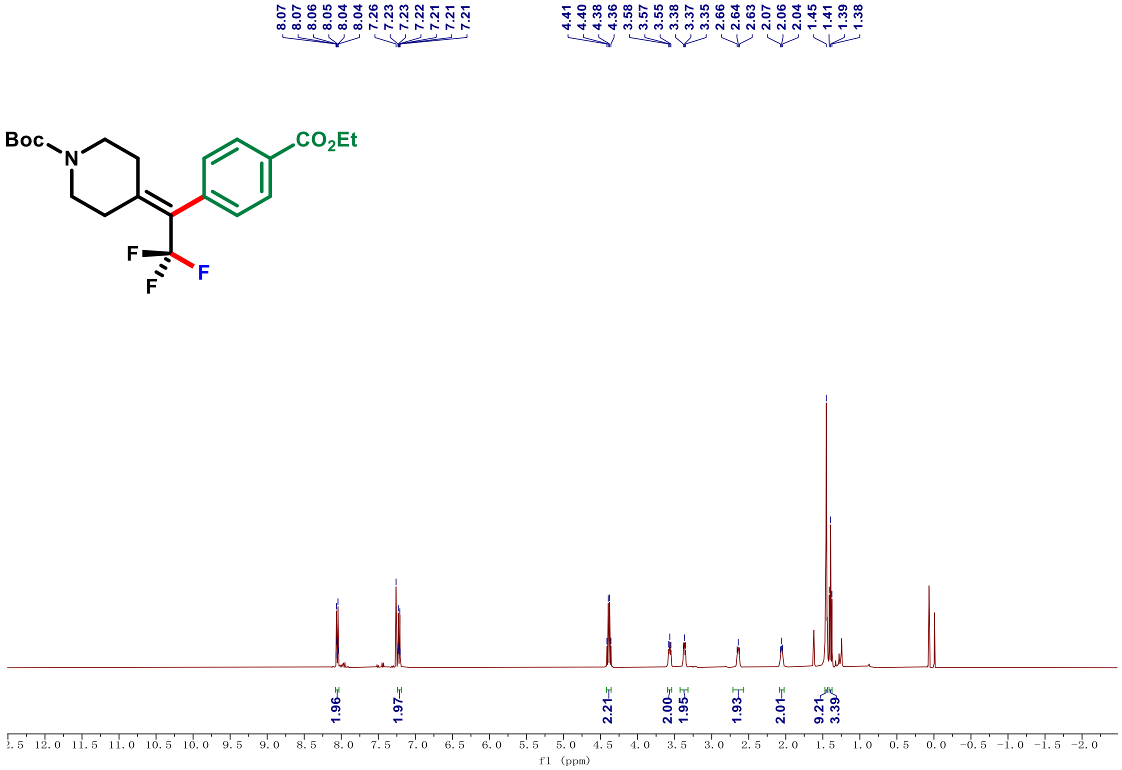

^{19}F NMR Spectrum of **3aca** ($CDCl_3$, 376 MHz)

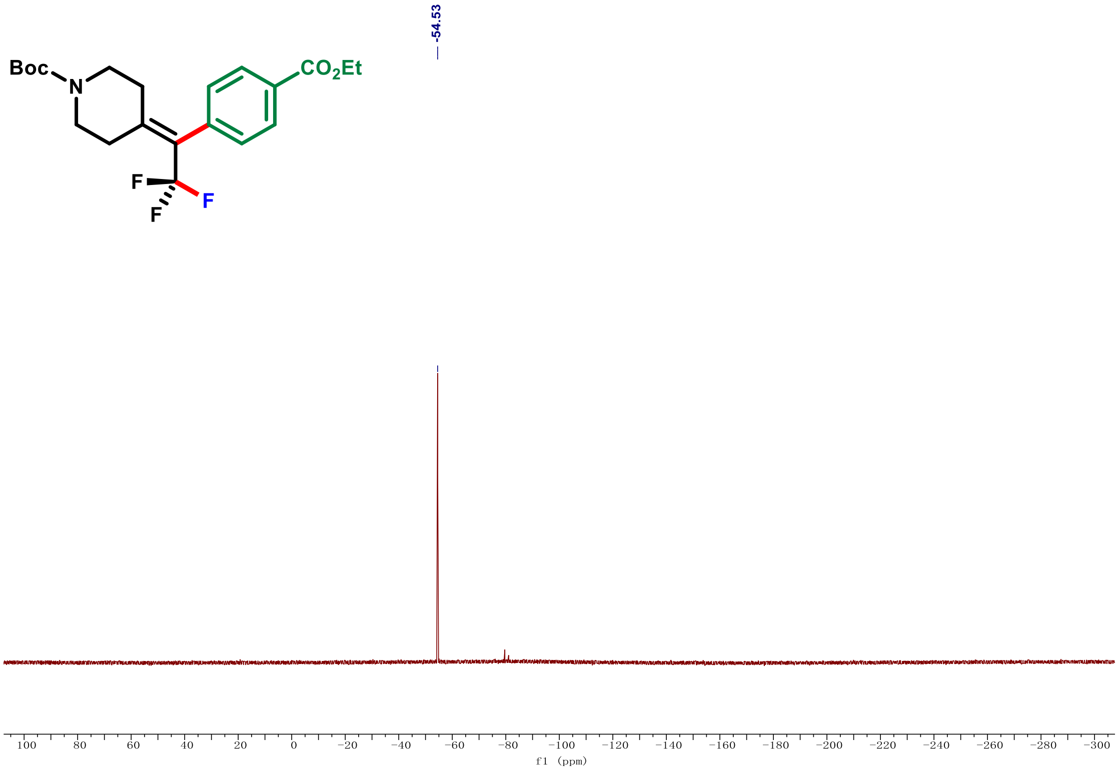

^{13}C NMR Spectrum of **3aca** ($CDCl_3$, 101 MHz)

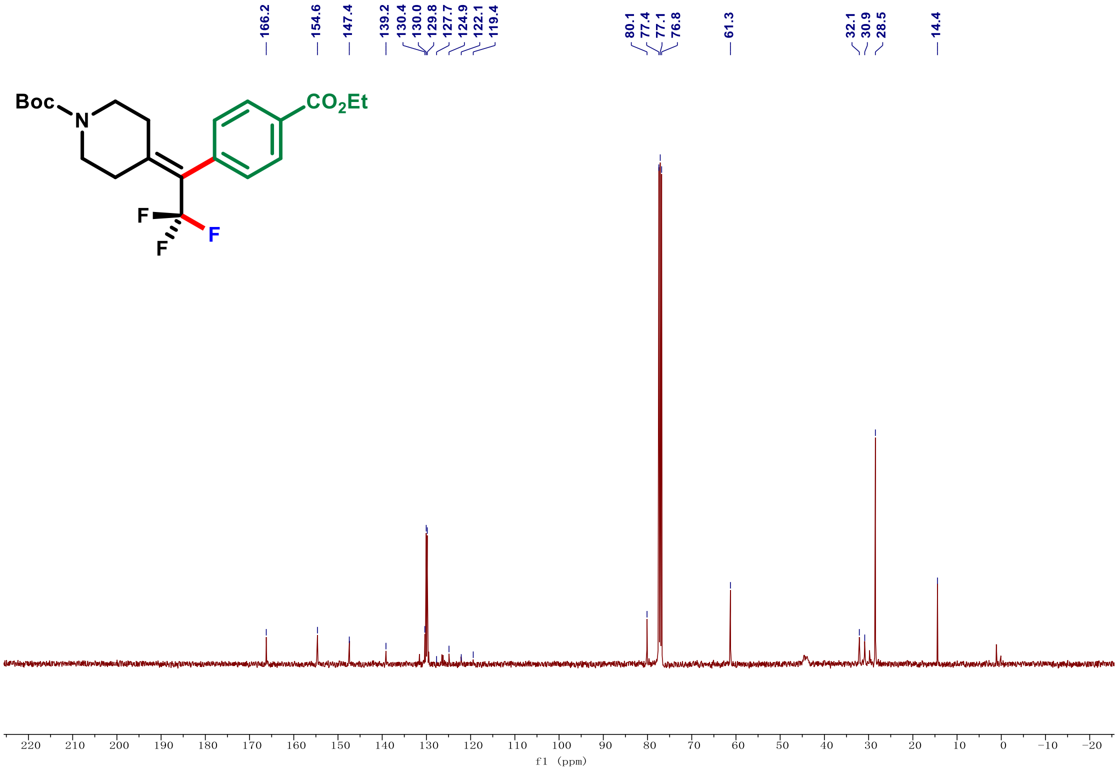

^{1}H NMR Spectrum of **3adk** ($CDCl_3$, 400 MHz)

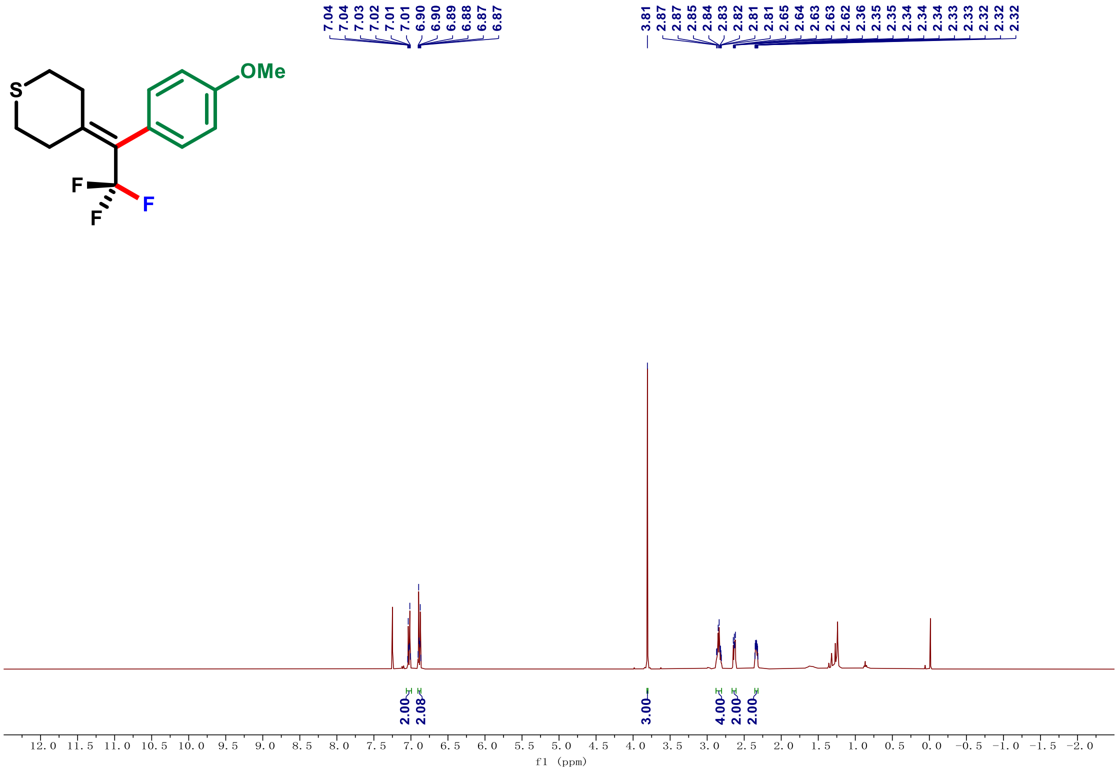

^{19}F NMR Spectrum of **3adk** ($CDCl_3$, 376 MHz)

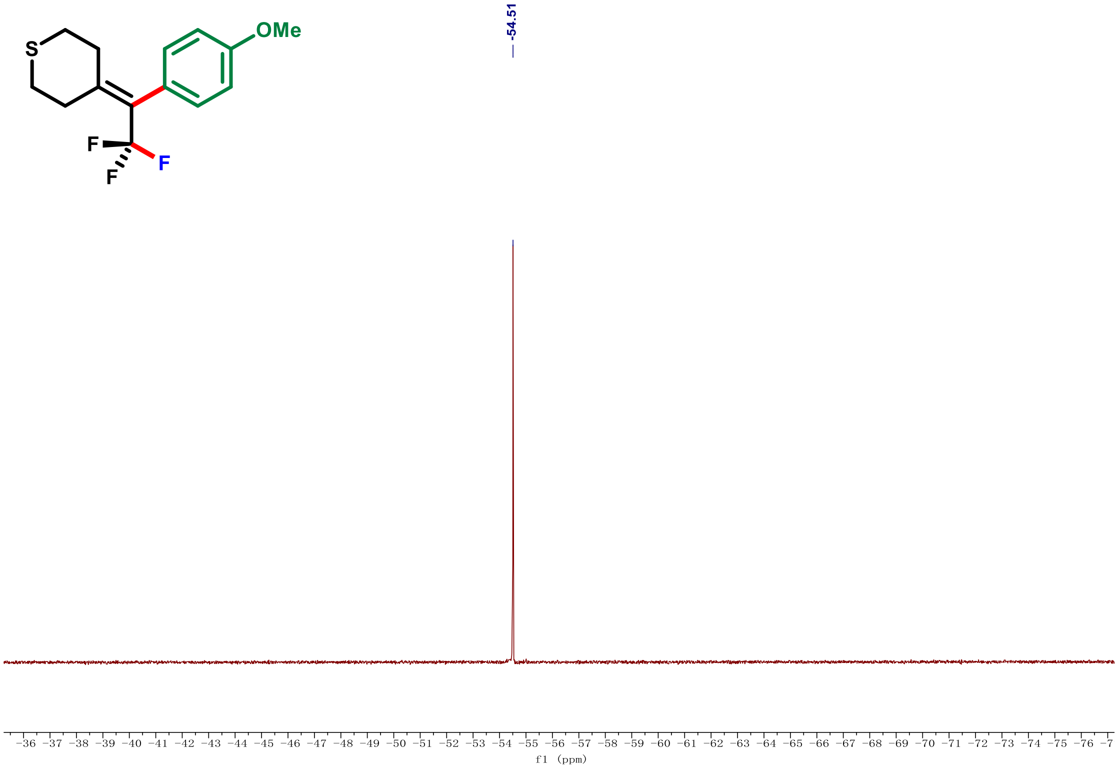

^{13}C NMR Spectrum of **3adk** ($CDCl_3$, 101 MHz)

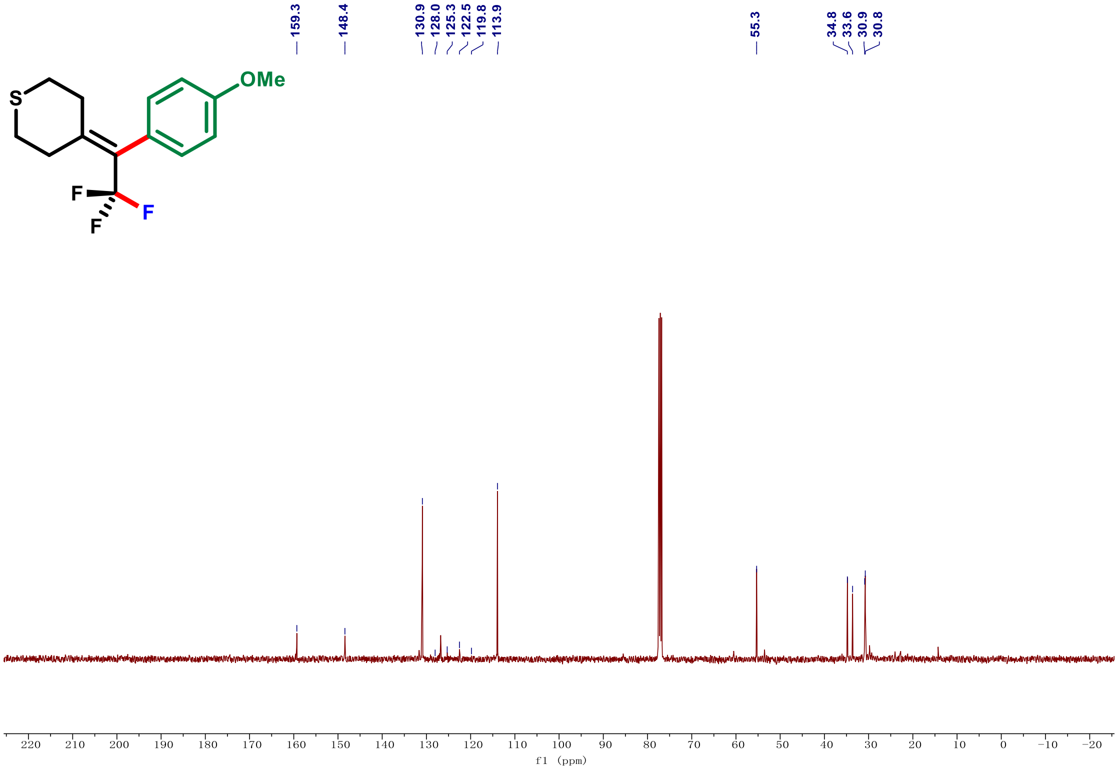

^{1}H NMR Spectrum of **3aea** ($CDCl_3$, 400 MHz)

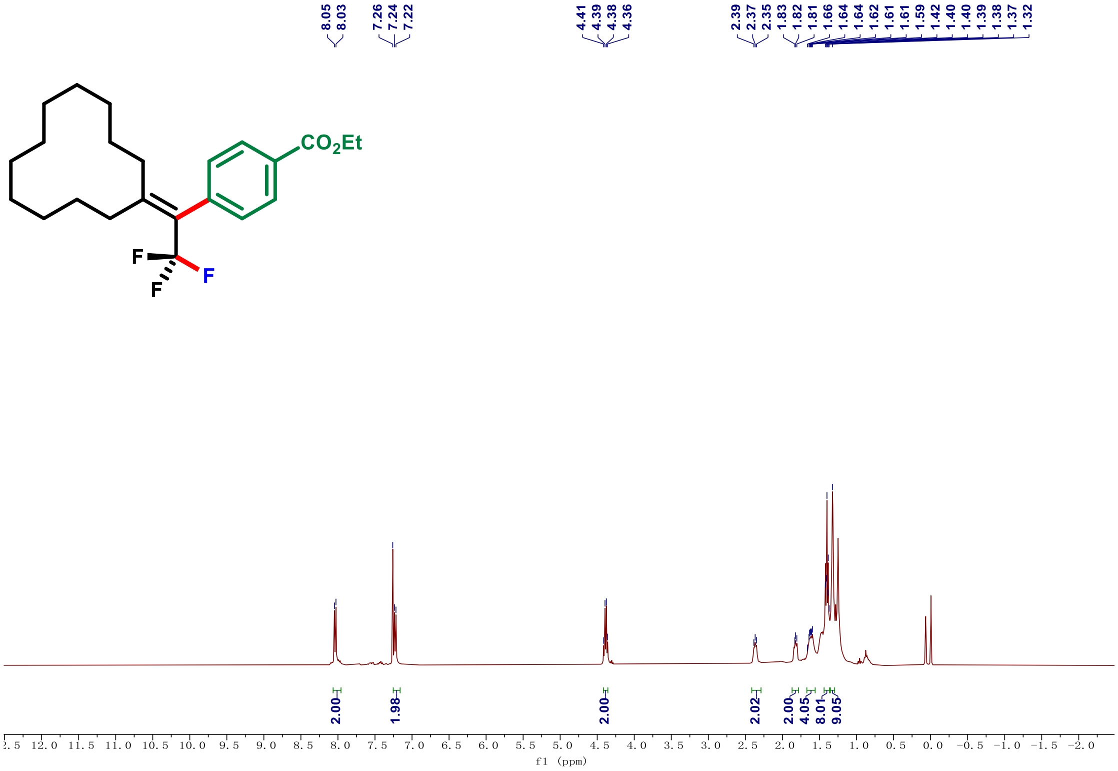

^{19}F NMR Spectrum of **3aea** ($CDCl_3$, 376 MHz)

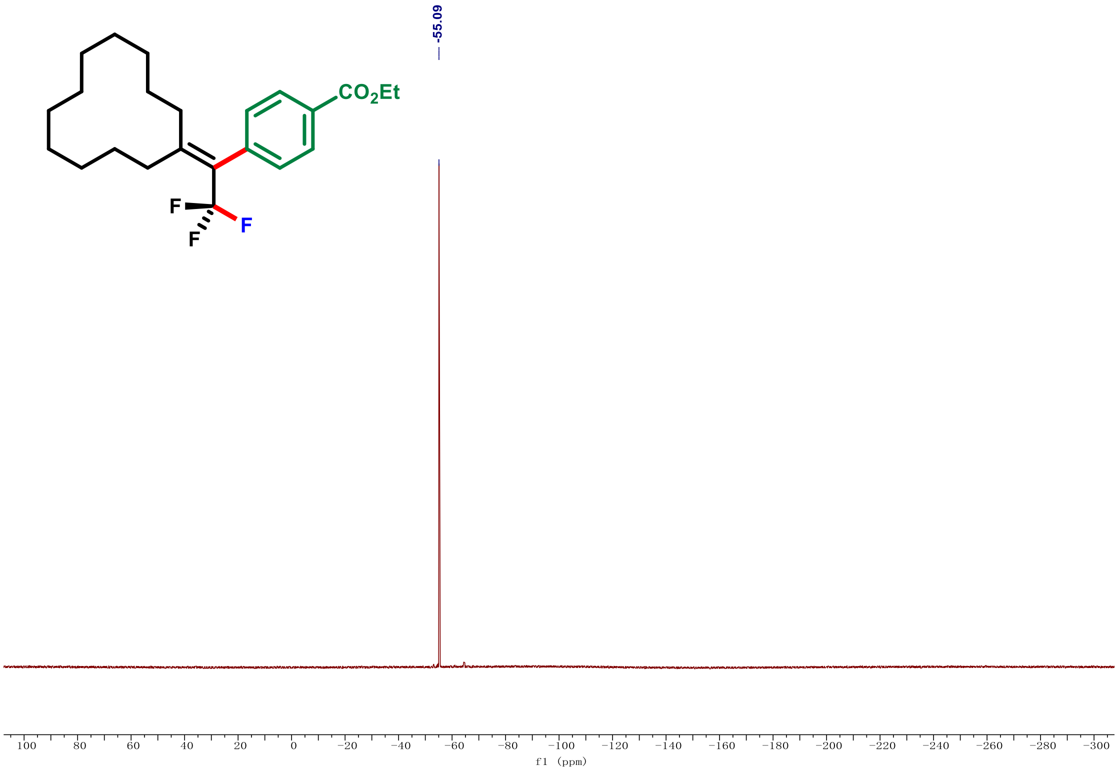

^{13}C NMR Spectrum of **3aea** ($CDCl_3$, 101 MHz)

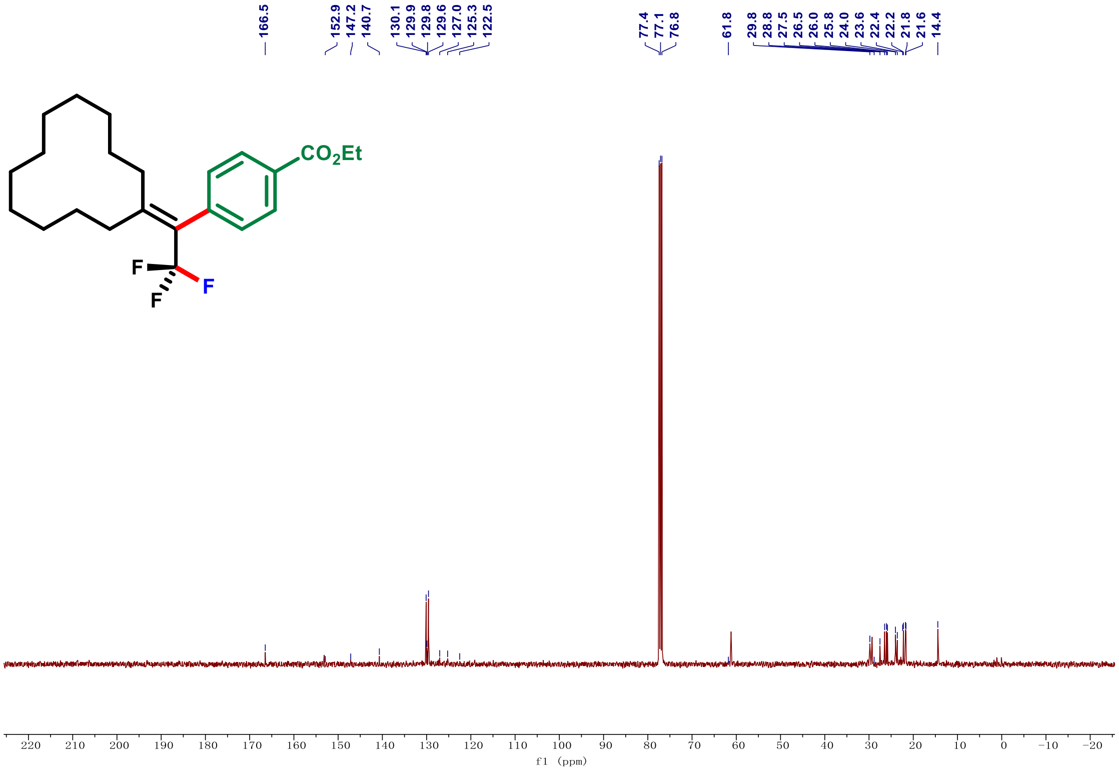

^{1}H NMR Spectrum of **3sf** ($CDCl_3$, 400 MHz)

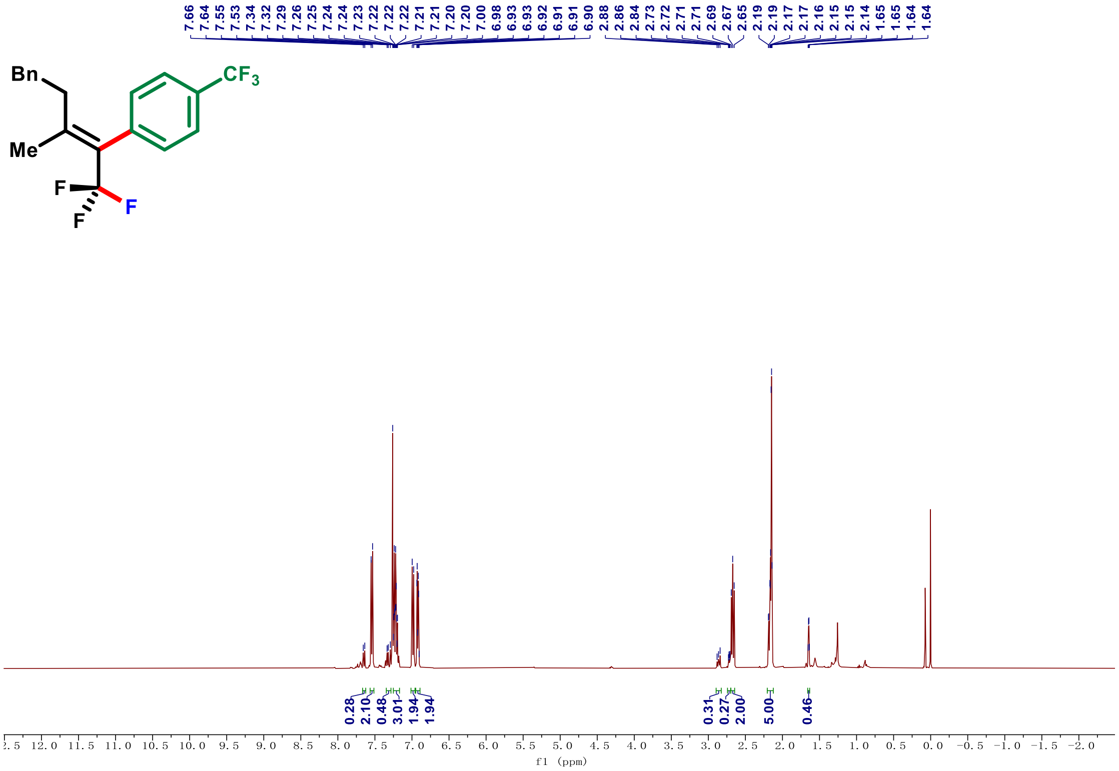

^{19}F NMR Spectrum of **3sf** ($CDCl_3$, 376 MHz)

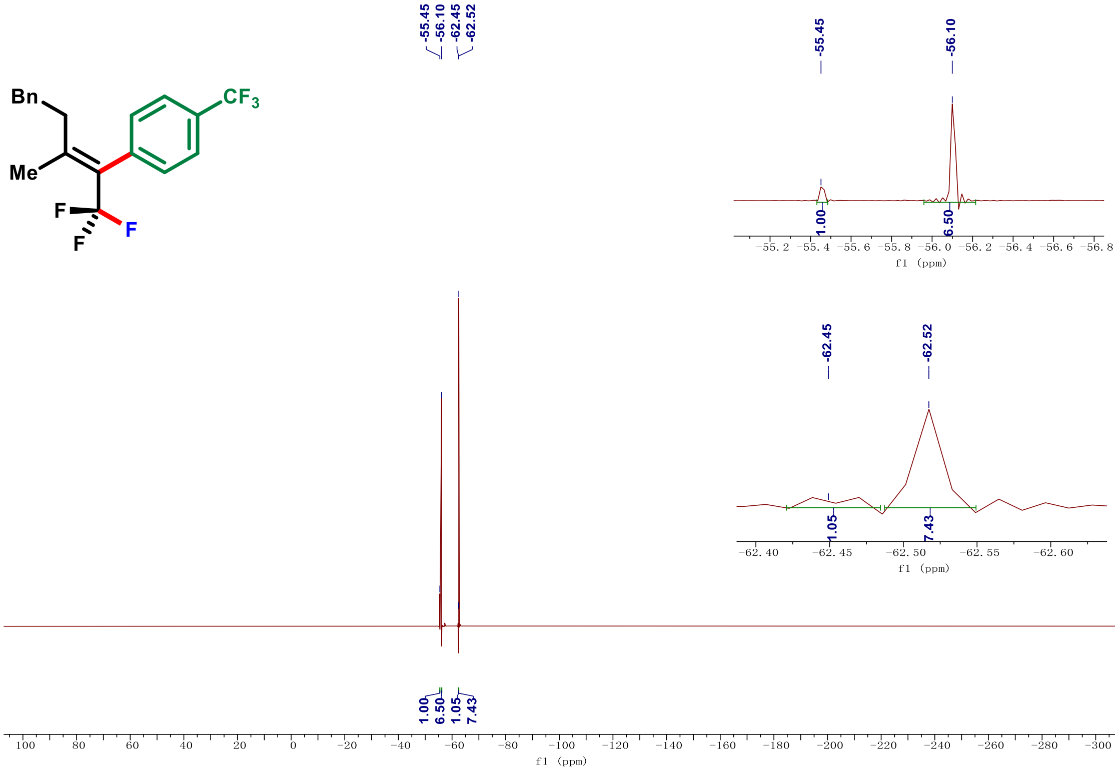

^{13}C NMR Spectrum of **3sf** ($CDCl_3$, 101 MHz)

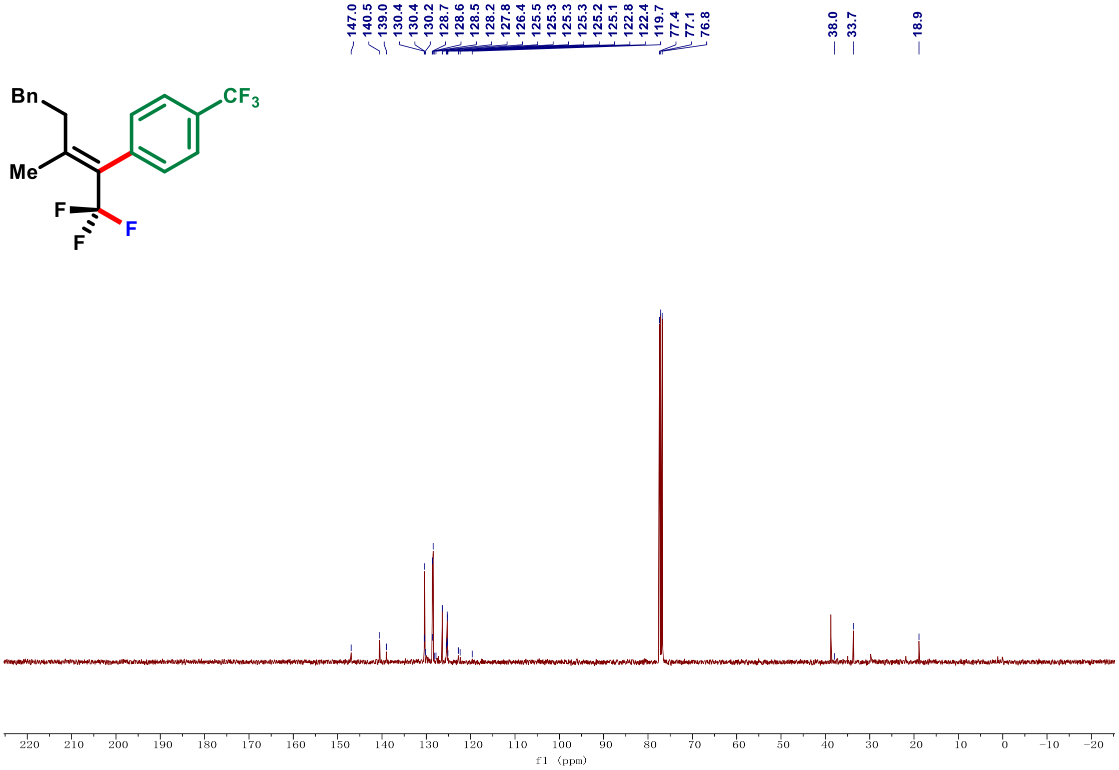

^{1}H NMR Spectrum of **3sp** ($CDCl_3$, 400 MHz)

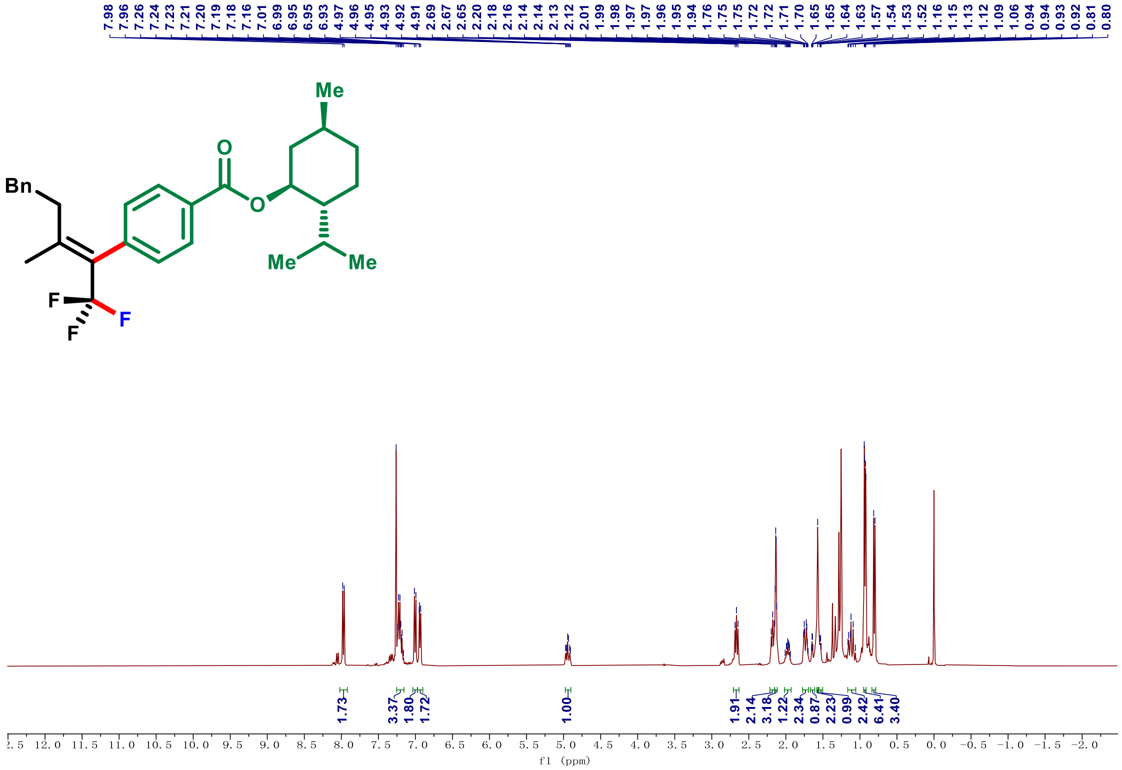

^{19}F NMR Spectrum of **3sp** ($CDCl_3$, 376 MHz)

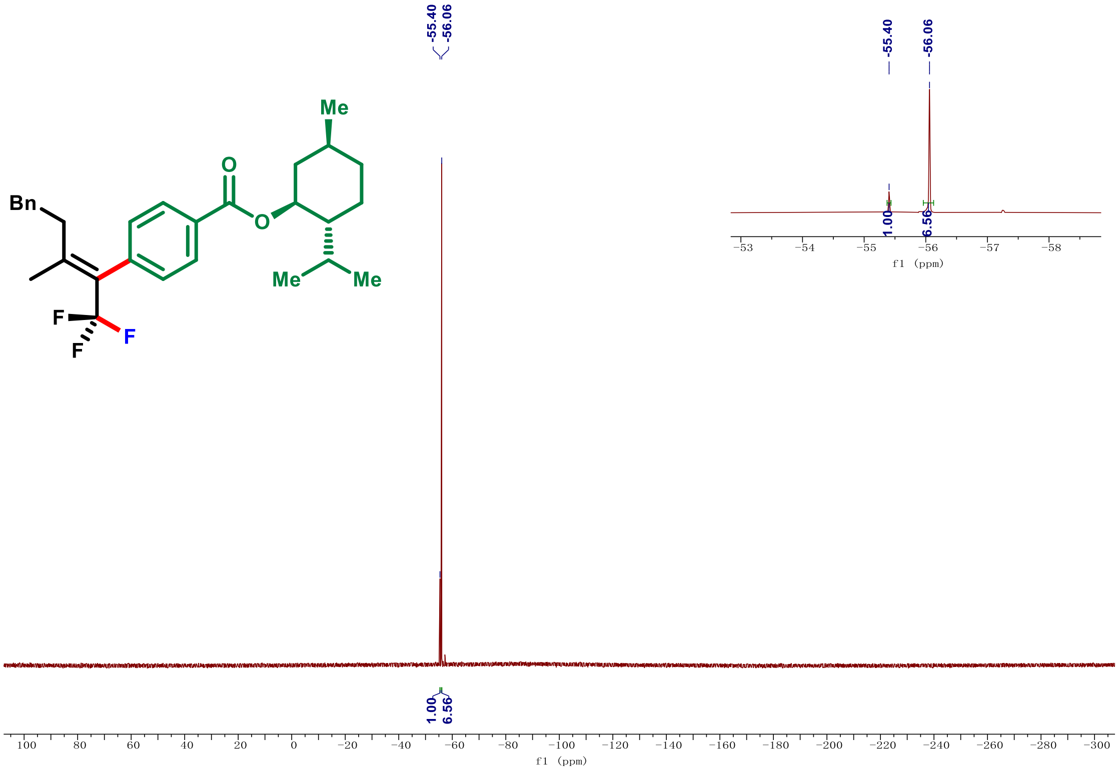

[13] C NMR Spectrum of **3sp** ($CDCl_3$, 101 MHz)

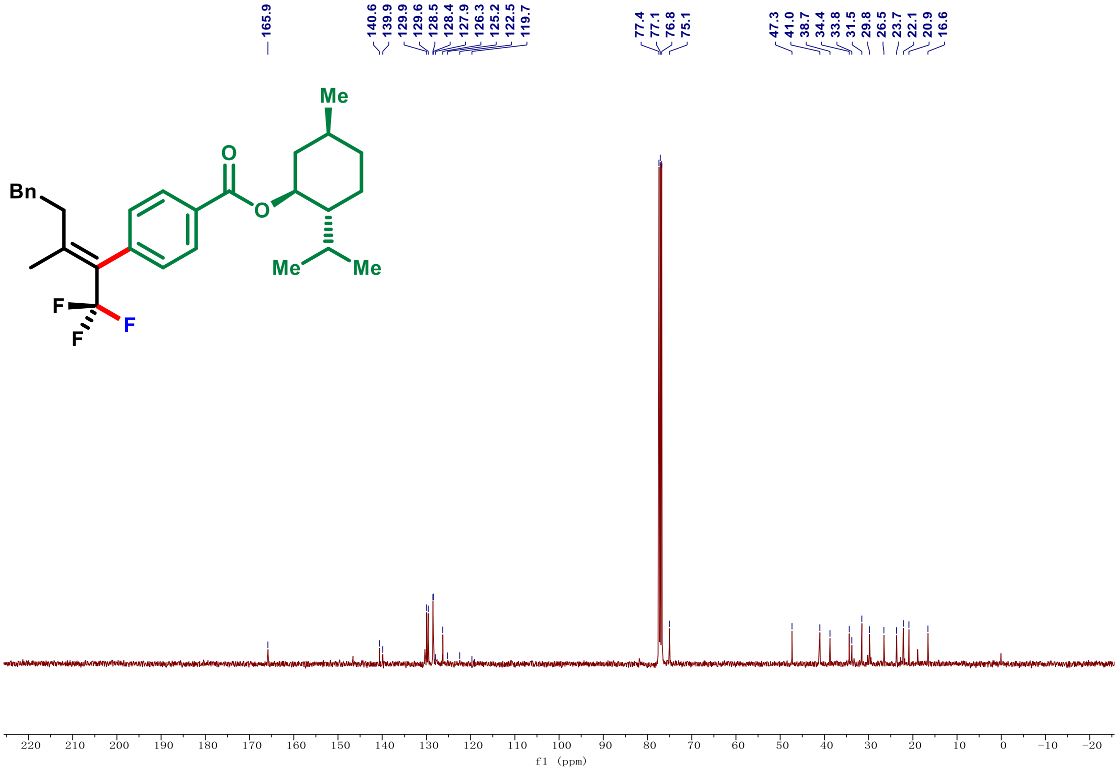

[1] H NMR Spectrum of **3ub** ($CDCl_3$, 400 MHz)

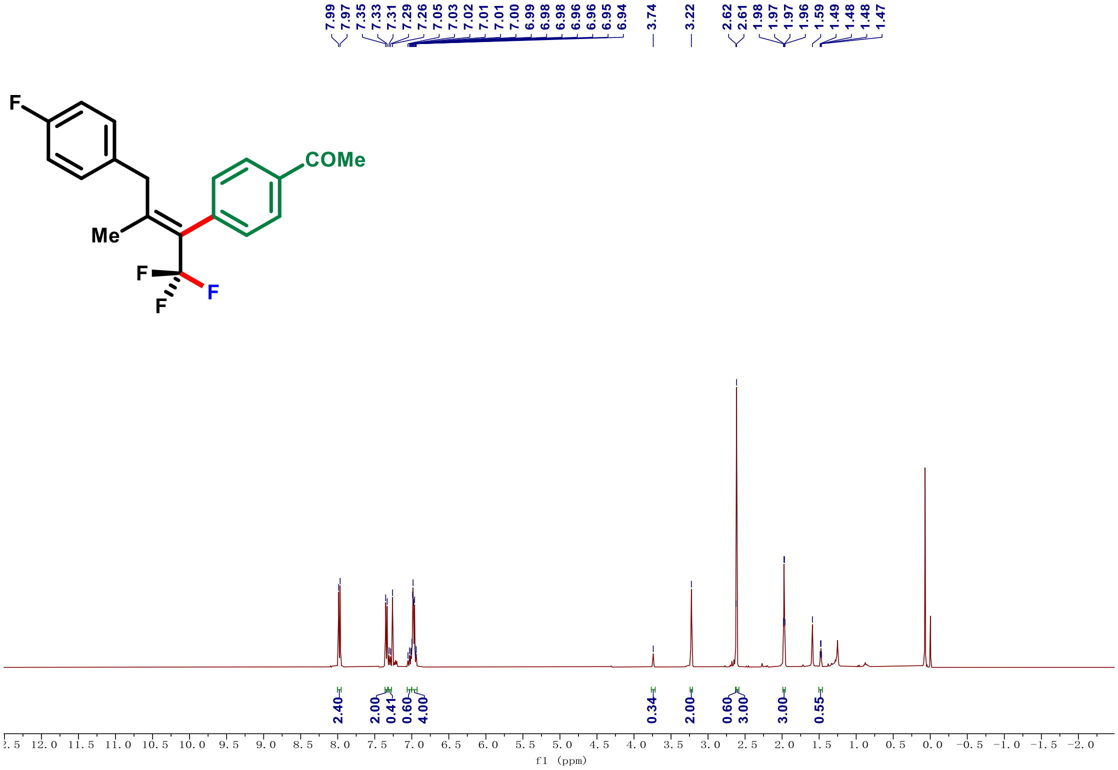

^{19}F NMR Spectrum of **3ub** ($CDCl_3$, 376 MHz)

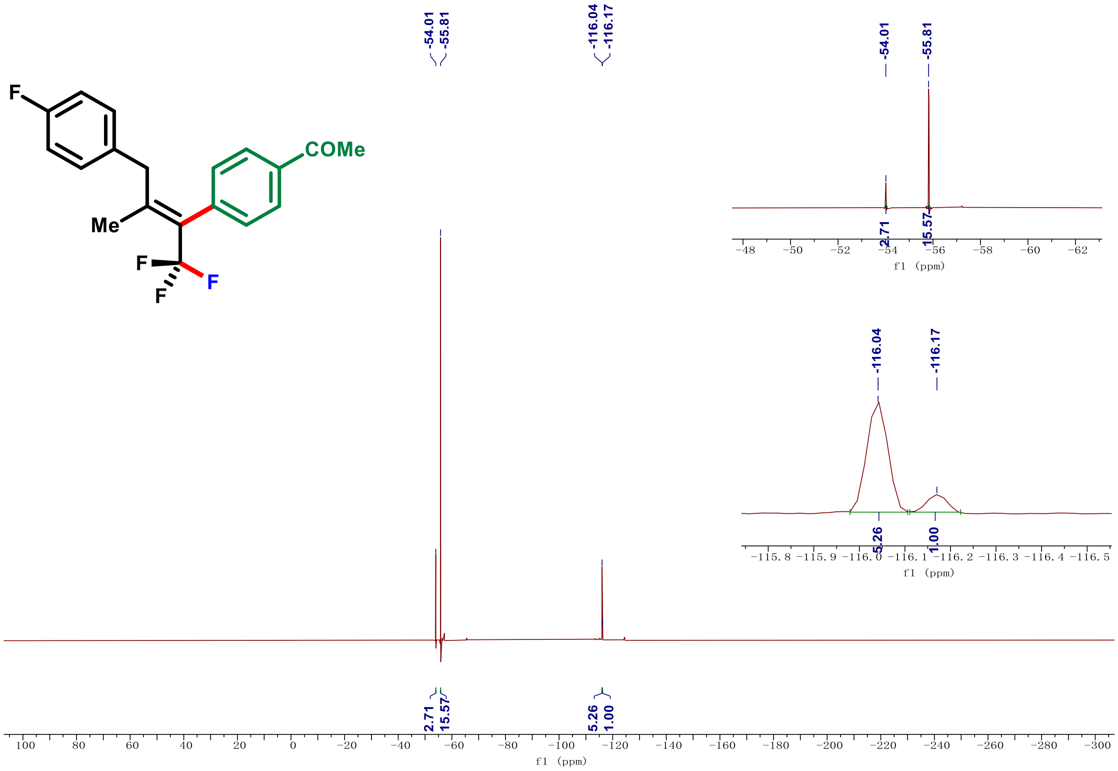

^{13}C NMR Spectrum of **3ub** ($CDCl_3$, 101 MHz)

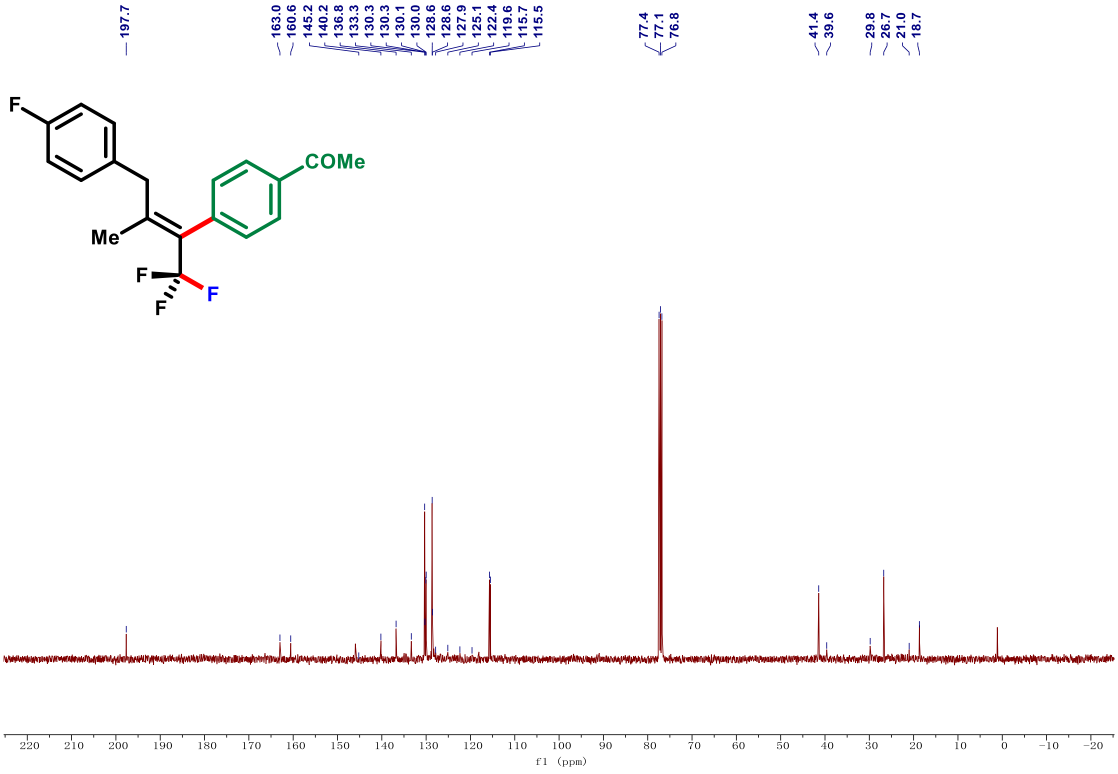

^{1}H NMR Spectrum of **3uh** ($CDCl_3$, 400 MHz)

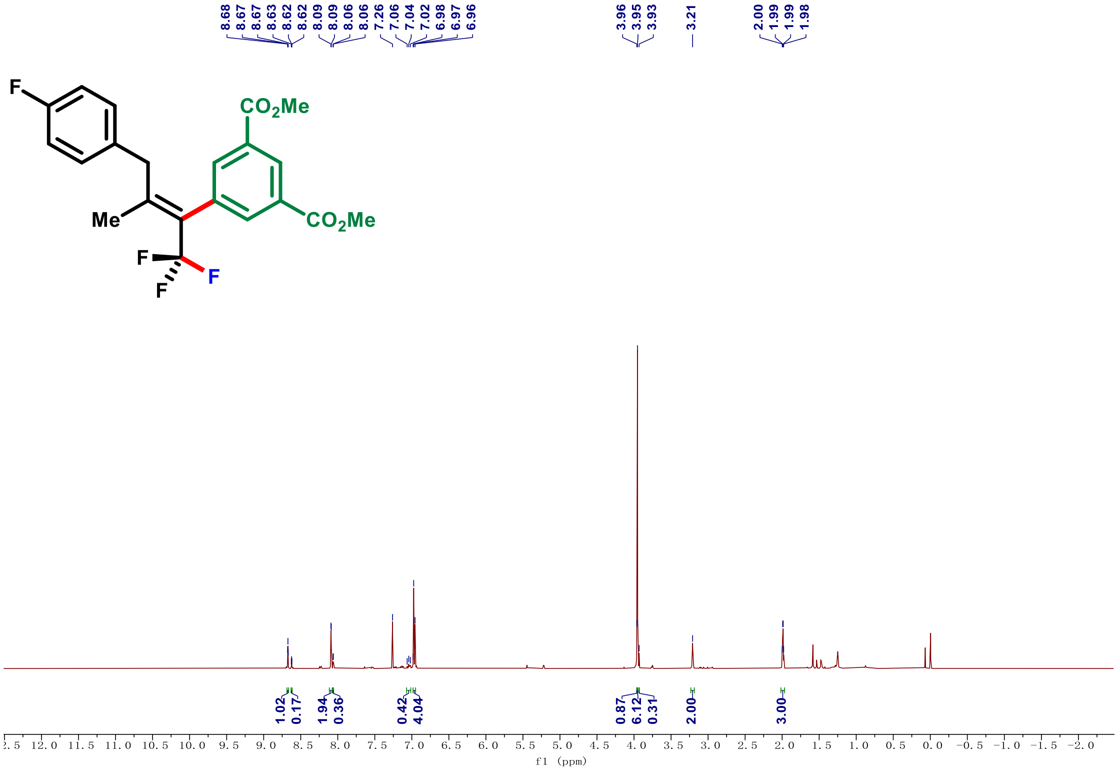

^{19}F NMR Spectrum of **3uh** ($CDCl_3$, 376 MHz)

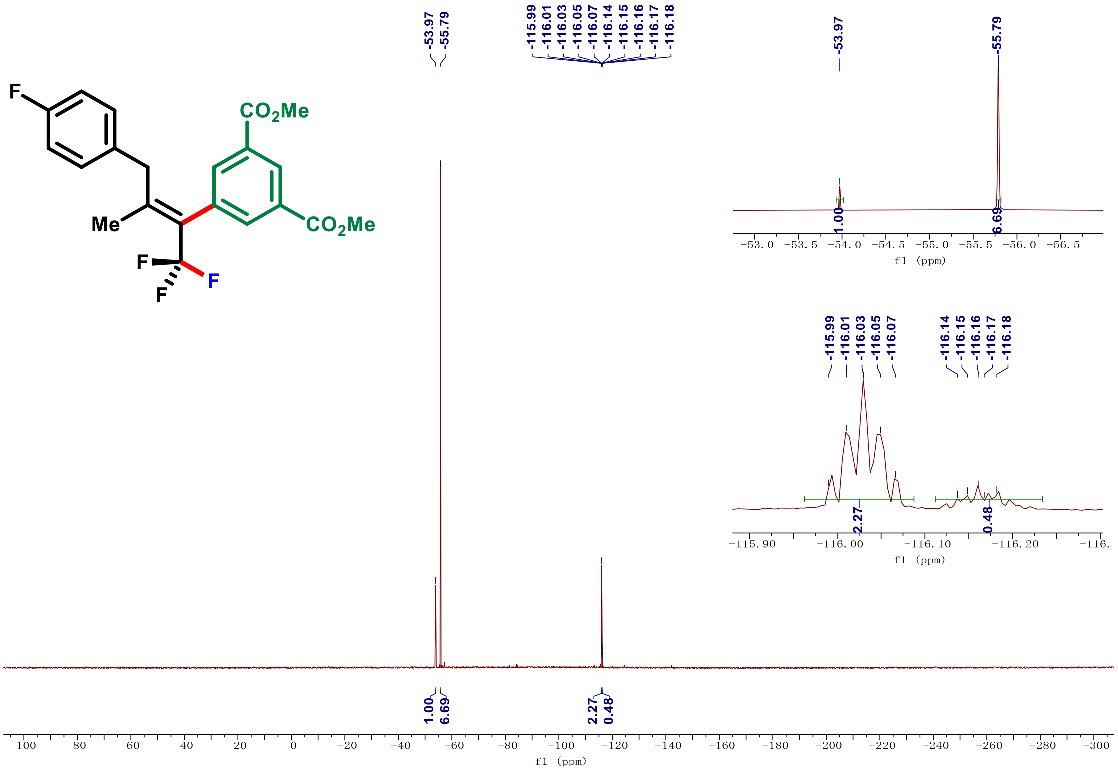

^{13}C NMR Spectrum of **3uh** ($CDCl_3$, 101 MHz)

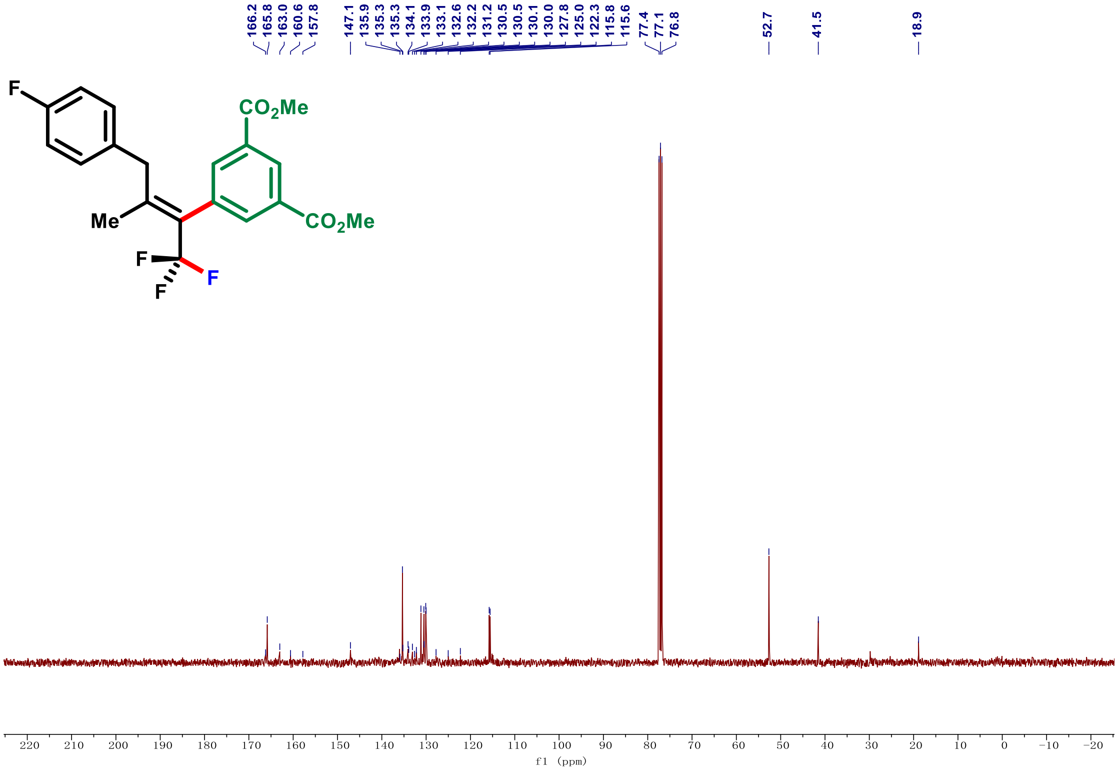

^{1}H NMR Spectrum of **3afa** ($CDCl_3$, 400 MHz)

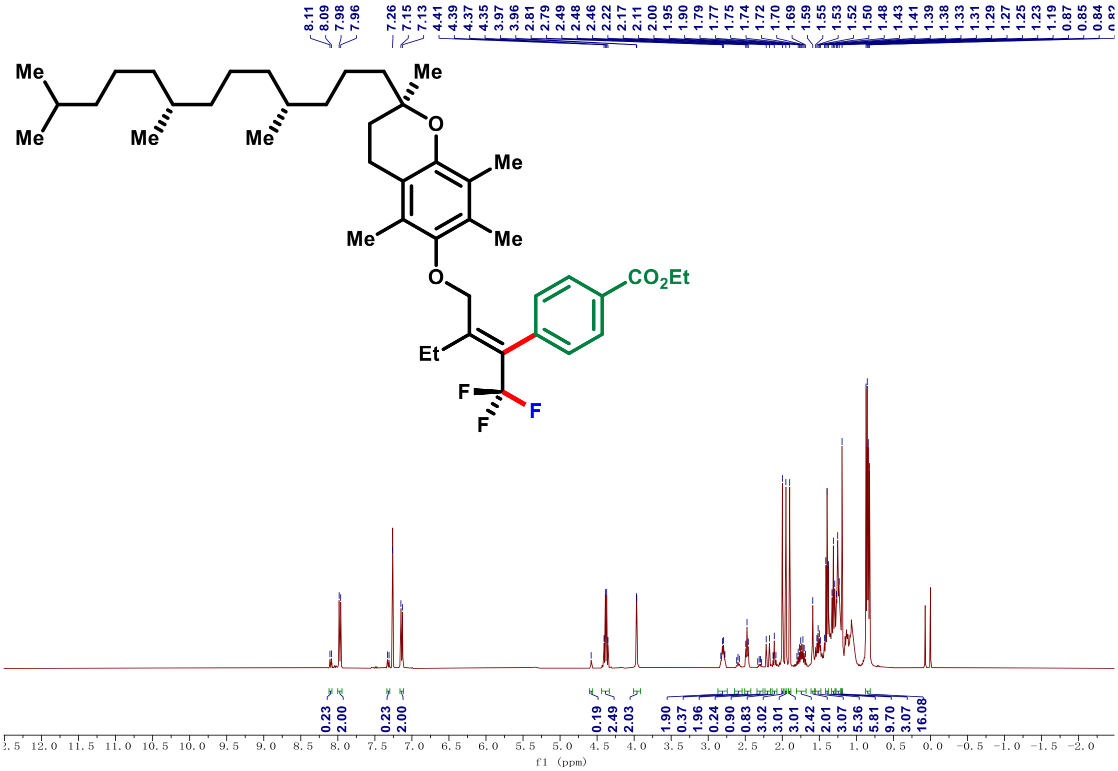

^{19}F NMR Spectrum of **3afa** ($CDCl_3$, 376 MHz)

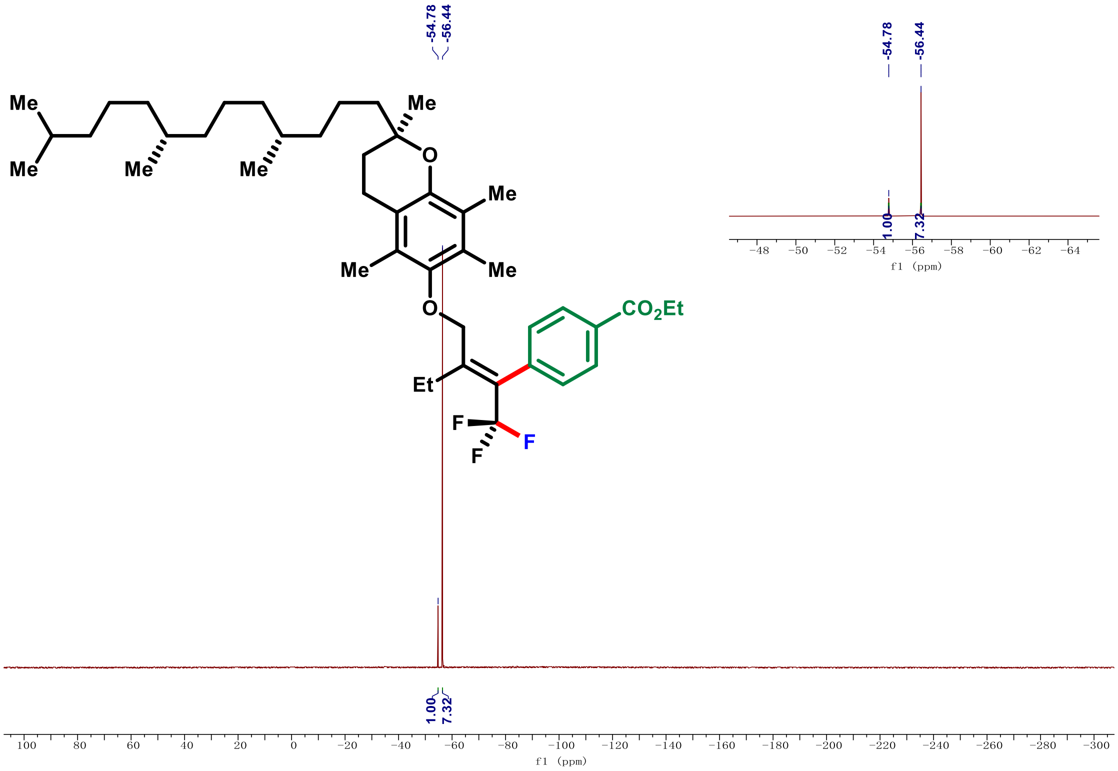

^{13}C NMR Spectrum of **3afa** ($CDCl_3$, 101 MHz)

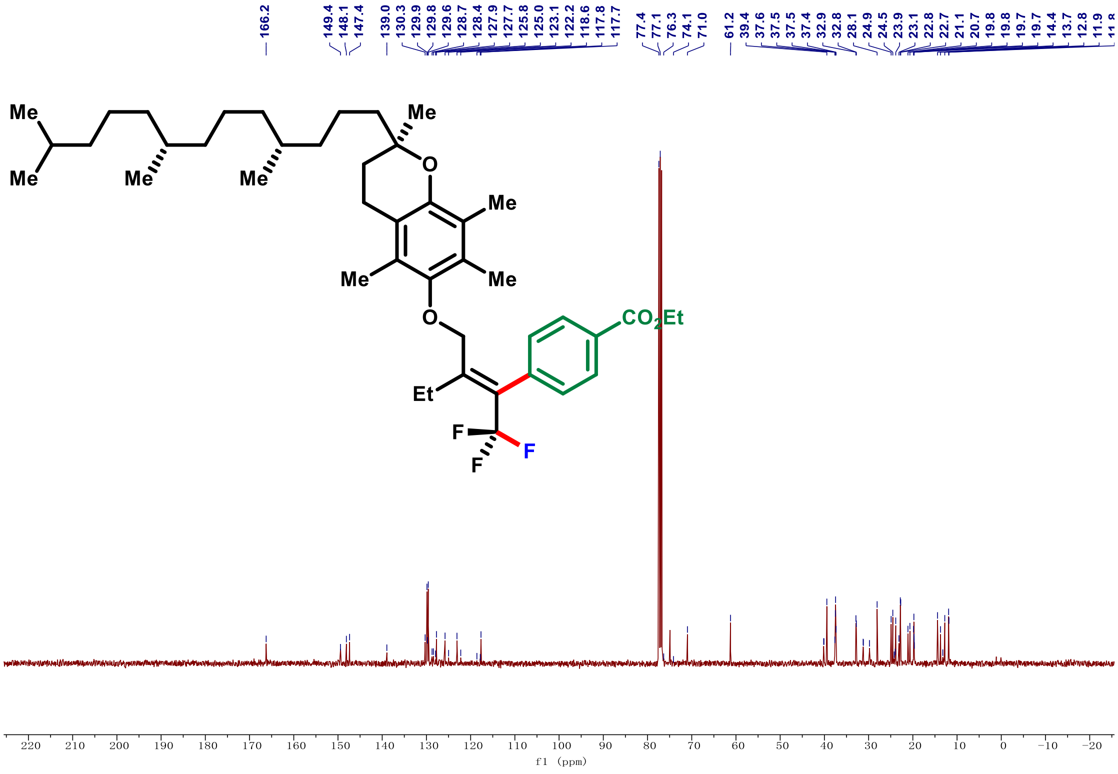

^{1}H NMR Spectrum of **3a** ($CDCl_3$, 400 MHz)

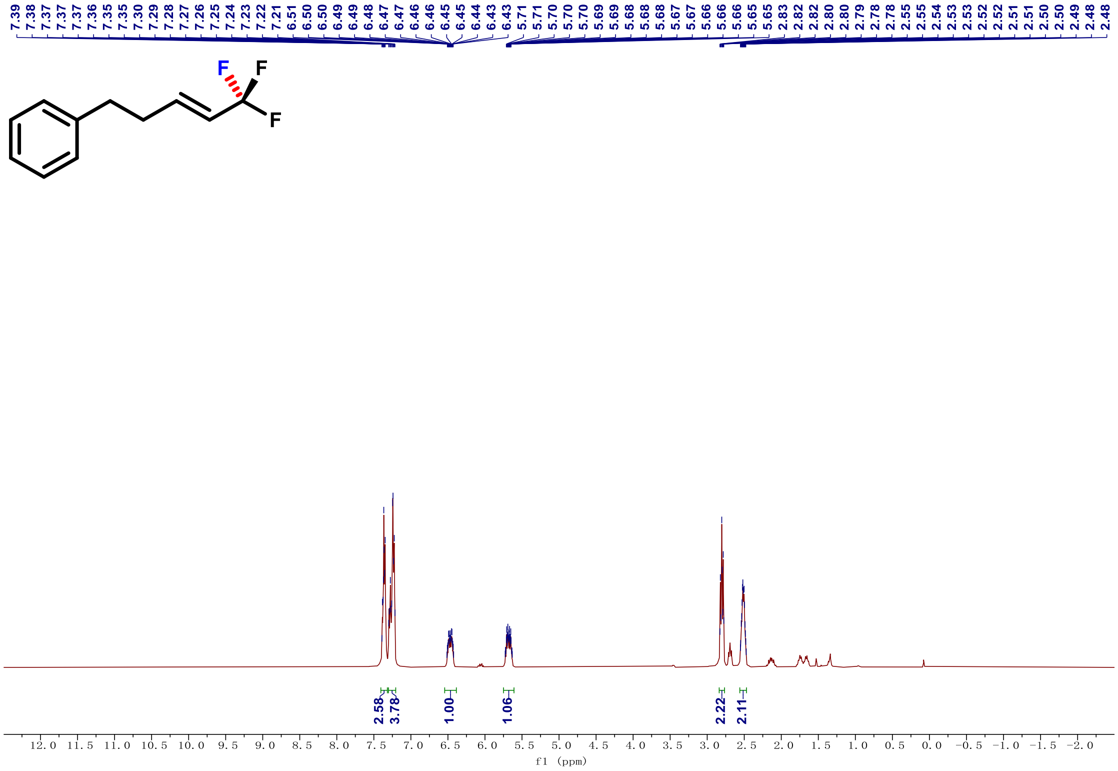

^{19}F NMR Spectrum of **3a** ($CDCl_3$, 376 MHz)

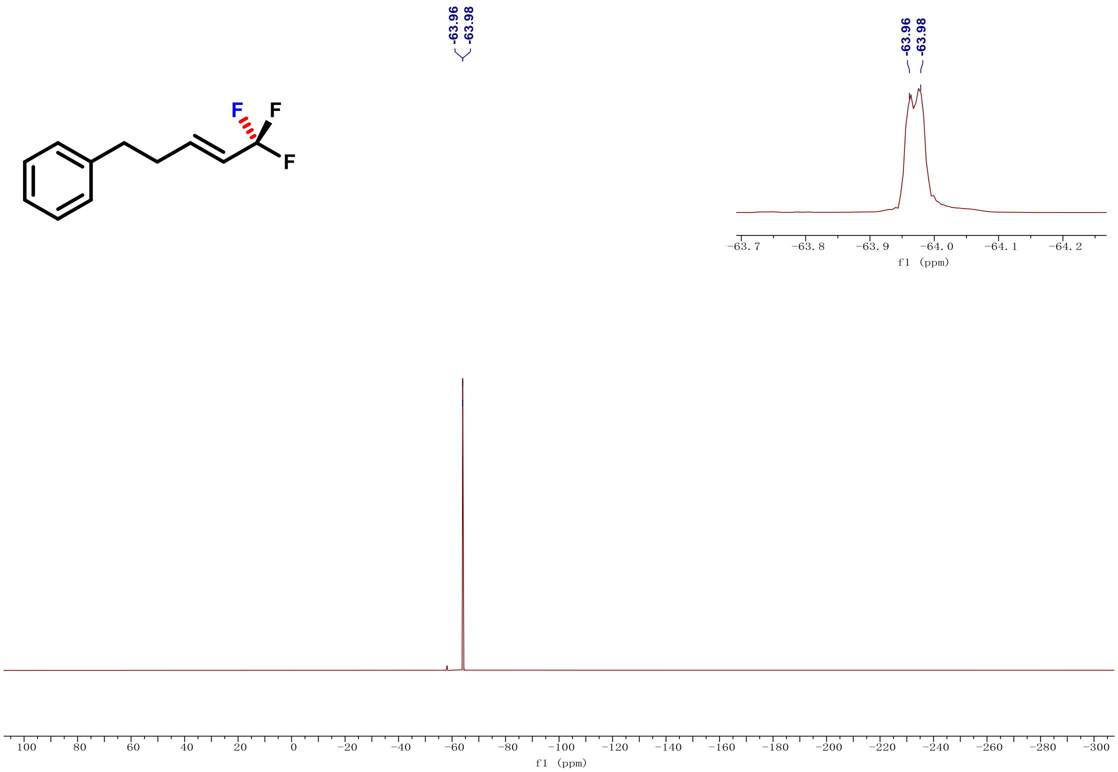

^{13}C NMR Spectrum of **3a** ($CDCl_3$, 101 MHz)

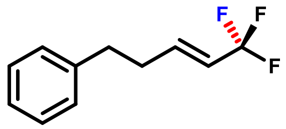

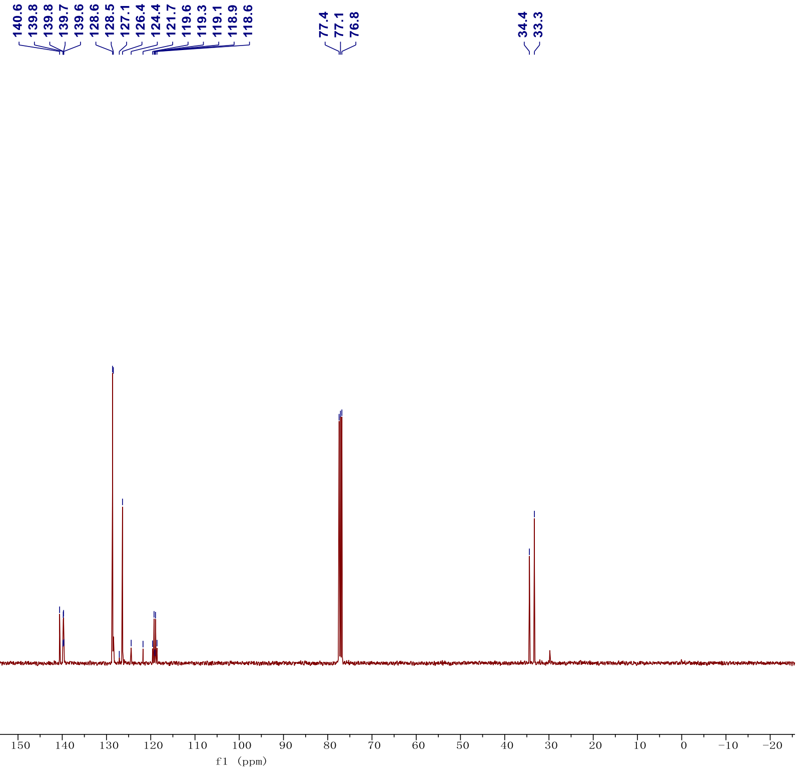

Supplement: SC-015-D3SC06060H-s001 [file SC-015-D3SC06060H-s001.pdf]
